# Supplementary material for: INTERGROWTH-21st Project international INTER-NDA standards for child development at 2 years of age: an international prospective population-based study
Source: BMJ Open. 2020 Jun 7;10(6):e035258. doi: 10.1136/bmjopen-2019-035258 (PMC7282399; doi:10.1136/bmjopen-2019-035258)
Supplement: Supplementary data [file bmjopen-2019-035258supp001.pdf]

**Supplementary Information**

**Supplementary Information S1** Characteristics of normative scales and thresholds used to identify neurodevelopmental impairment in commonly used developmental assessments for two year-old children.

**Supplementary Information S2** Factors associated with poor neurodevelopmental outcomes in young children: Characteristics of the normative sample for the INTERGROWTH-21st Project International INTER-NDA standards, and for other neurodevelopmental tools evaluating two year-old children.

**Supplementary Information S3** The INTERGROWTH-21st Fetal Growth Longitudinal Study (FGLS) individual participant entry criteria for mothers at the time of antenatal booking in the first trimester of pregnancy.

**Supplementary Information S4** The INTERGROWTH-21st Neurodevelopment Assessment (INTER-NDA) data recording form.

**Supplementary Information S5** The INTERGROWTH-21st Neurodevelopment Assessment (INTER-NDA) Protocol Adherence Checklist.

**Supplementary Information S6** Results of the INTERGROWTH-21st Neurodevelopment Assessment (INTER-NDA) standardized evaluations between specialist and non-specialist assessors to (i) score and (ii) administer the INTER-NDA

**Supplementary Information S7** The INTERGROWTH-21st Neurodevelopment Assessment (INTER-NDA) scoring system and interpretation of domain scores.

**Supplementary Information S8** Formulae and tables for the conversion of raw scores to standardised (scaled) scores (range 0-100).

**Supplementary Information S9** INTER-NDA domain scores centiles including and excluding children scoring above the CBCL threshold for clinical (97th centile) problems.

**Supplementary Information S10** Comparison of INTERGROWTH-21st Neurodevelopment Assessment (INTER-NDA) domain scores between two year-old girls and boys

**Supplementary Information S11** INTERGROWTH-21st Project Study Protocol (see separate file)

**Supplementary Information S12** STROBE checklist (see separate file)

**S1** Characteristics of normative scales and thresholds used to identify neurodevelopmental impairment in commonly used developmental assessments for two year-old children.

| Tool | Subscales measured | Characteristics of normative sample | Cut-off score for neurodevelopmental delay | Details on scoring and interpretation of results |
|---|---|---|---|---|
| The Bayley Scales of Infant Development – III edition (BSID III) [a*] | Cognitive, expressive and receptive communication, fine and gross motor, adaptive behaviour, social emotional. | Country: USA<br>Year: January and October 2004<br>Sample Size: 24-month normative sample for cognitive, language and motor scales: 100 children (totally 1700 children aged 16 days through 43 months 15 days divided into 17 age groups of 100 children each). Normative sample for the social-emotional scale was based on 456 children and the adaptive behaviour scale was based on 1,350 children.<br>Sample characteristics: Sample selected to match the 2000 United States census. | Several criteria: **25% delay** in functioning when compared to same age peers; based on SD (**< -1 SD** i.e. cut-off thresholds of 85 for moderate impairment; **< -2 SD** i.e. cut-off thresholds of 70 for severe impairment) or **performance of a certain number of months below** the child's chronological age[1]. | Scoring for every item is either 1 (credit) or 0 (no credit).<br><br>Scaled scores, composite scores, growth scores, centiles, & age-equivalents are obtained from raw scores. |
| The Malawi Developmental Assessment Tool (MDAT)[b*] | Gross motor, fine motor, language and social. | Country: Malawi<br>Year: June 2006 to July 2007<br>Sample size: 1426 normal healthy children aged 0 to 6 years<br>Sample characteristics: Those born preterm at <32 weeks' gestation, or with significant malnutrition using WHO criteria, medical problems or significant neurodisability were excluded. | **<25%** (upper limit of lowest quartile) | Plot children on MDAT normal reference ranges, corresponding to 25%, 50% and 75% of children passing each item. |
| The Griffiths Mental Development Scales[c*] | Locomotor, expressive and receptive language, personal-social, hand and eye coordination, performance, practical reasoning. | Country: UK and Ireland<br>Year: 1960s, revised in 2015<br>Sample size: 1026 children<br>Sample characteristics: National representative sample of children in UK; stratified according to geographical region and proportionate to the 1997 ONS population ratios[1]. | Centiles and z -scores; **z score < -2 or SD< -2** indicates significant developmental delay on that subscale. | Raw scores are converted into z scores, developmental quotients and centile scores – these are used to obtain developmental age equivalents. A general quotient may also be obtained. |
| Pre-school version of Child Behavior Checklist (CBCL)[d*] | Problem and syndrome scales for emotionally reactive; anxious/depressed; somatic complaints; withdrawn; sleep problems; attention problems; aggressive behaviour. | Country: USA<br>Year: 1979, 2000<br>Sample size: Originally normed on 1728 US children.<br>Sample characteristics: -<br><br>Note: Multicultural norms available. | **>93$^{rd}$ centile** is abnormal (norms vary according to societies, and map onto the Diagnostic and Statistical Manual for Mental Disorders).<br><br>Any score that falls below the 93$^{rd}$ centile is considered normal, scores between the 93-97$^{th}$ centile are borderline clinical, and any score above the 97$^{th}$ centile is considered to be in the clinical range. | Different norms for different societies. The CBCL uses software to generate raw score; T score and centile score for each problem score as well as a total problem score, which ranges from 0 to 200. The standard scores are scaled so that 50 is average for the child's age and sex, with a standard deviation of 10 points. Higher scores indicate greater problems. |
| The Rapid Neurodevelopmental Assessment | Primitive reflexes, gross motor, fine motor, vision, | Country: Bangladesh<br>Year: 2010<br>Sample size: 81 children aged ≥3 to 24 months in urban (n = 47) and rural | **< -2 SD**: threshold for severe impairment;<br>**< -1 SD:** threshold for mild | For every item, the severity of functional limitations was determined as mild, moderate or severe limitation. |

| | | | | |
|---|---|---|---|---|
| (RNDA)[e]* | hearing, speech, cognition, behaviour, and seizures. | (n = 34) community-based populations<br>Sample characteristics: 15% did not 'look' properly nourished, parental concerns regarding child development were expressed in 8% and for 50% at least one parent was illiterate. | impairment.<br>If low scores in >1 domain; the child is classified as having 'any' neurodevelopmental impairment. | |
| The Ages and Stages Questionnaire III edition (ASQ III)[f] | Communication, gross motor, fine motor, problem solving and personal-social. | Country: USA<br>Year: January 2004 and June 2008<br>Sample Size: 15,138 children (1,443 aged 24 months).<br>Sample characteristics: 76% of the sample had one or no known risk factor, 19% had 2 risk factors and 4% 3 or more risk factors. Risk factors were defined as extreme poverty, maternal age ≤19 years, maternal education <12$^{th}$ grade; involvement of child protective services with the family for abuse and/or neglect; medical risk, including prematurity; and infant's birth weight less than 3 pounds, 5 ounces. | **< -2SD** | Item scoring: 0 = not true, 1 = somewhat / sometimes true, or 2 = very true or often true of the child. |
| The Parents' Evaluation of Developmental Status (PEDS)[g] | General development. | Country: USA<br>Year: 1997<br>Sample size: 2823 families<br>Sample characteristics: Families from varying backgrounds, including SES and ethnicity. | Table for using scores to identify parental difficulties, non-significant concerns, one significant concern or two or more significant concerns by shading boxes based on scores. These are then used to select associated algorithms for further screening and/or referral. | Eight page booklet used to score the PEDS response form; an algorithm uses these scores to identify associated pathways for further screening and/or referral. |
| Caregiver-reported Early Developmental Instruments (CREDI)[h] | Long Form: Motor, cognitive, language, social-emotional and overall.<br><br>Short form: Overall development. | Countries: Brazil, Cambodia, Chile, Colombia, Ghana, Guatemala, India, Nepal, Philippines and USA<br>Year: 2017-2018<br>Sample size: 7807 children aged 0-35 months<br>Sample characteristics: Children with an "ideal home environment" defined through maternal educational attainment<br>(college or higher), and the number of activities done by adults with the child in the last 3 days (at least 4 out of the 6 MICS home stimulation activities); authors acknowledge that "although the data were representative for local populations in Brazil, Ghana, Tanzania, and Zambia, the overall sample is not representative of any country or a global population of children". | Raw scaled scores, norm referenced standardised scores, and z scores. | Uses the CREDI software package in R. |
| Denver Development Screening Test II (DDST II)[i] | Personal social, fine motor adaptive, language and gross motor. | Country: USA<br>Year: 1980s and 1990<br>Sample size: 2096 children<br>Sample characteristics: Children from Colorado; based on 1988, 1989 and then the 1990 US census population. | **Centile ranks** (25th, 50th, 75th, and 90$^{th}$) are displayed as bar graphs and reflect the ages at which 25%, 50%, 75% and 90% of typically developing children in the standardisation sample completed the task.<br>Overall categories: Normal, and suspect. | Approximates a growth curve in its display of norms over time. The number of scores a child received below the normal expected range classifies the child as within normal, suspect, or delayed. If the child is suspect it is recommended that rescreening occur in 1-2 week. |
| Guide for Monitoring Child | Parental concerns, expressive language | Country: Turkey<br>Year: 1980s and 1990 | <10$^{th}$ centile for any domain; if a child did not demonstrate ≥1 of the age- | Age at which >90% of the study sample performed each milestone was computed. |

| | | | | |
|---|---|---|---|---|
| Development (GMCD)[j] | and communication, receptive language, relationship (social-emotional), play (social-emotional and cognitive) and self-help. | Sample size: 30 children in each age range<br>Sample characteristics: Sample selected as per WHO recommendations for a 'prescriptive sample' i.e. children were born healthy singletons with birth weight ≥2500 g and gestational age ≥37 weeks and had received preventive health care at 2 university-affiliated community well-child care clinics in Ankara from birth. The sample's growth was between the 5th and 95th centiles since birth; they had received and complied with the free iron prophylaxis available to children in Turkey (or had normal haemoglobin screens within 1 month of the study); were healthy, and growing normally, at their paediatric evaluation. They had not had "any health-related problems since birth apart from acute minor illnesses." | appropriate milestones, the GMCD interpretation was classified as "requires follow-up evaluation with or without intervention." | |

[a]©Pearson. Bayley Scales of Infant and Toddler Development, Third Edition (Bayley-III) - Training. Available from: 98 http://www.pearsonclinical.co.uk

[b]Gladstone M, Lancaster GA, Umar E, Nyirenda M, Kayira E, van den Broek NR, Smyth RL. The Malawi Developmental Assessment Tool (MDAT): the creation, validation, and reliability of a tool to assess child development in rural African settings. *PLoS medicine* 2010; **7:** e1000273.

[c]Luiz D, Barnard A, Knoesen N, Kotras N, Horrocks S, McAlinden P, Challis D, O'Connell R. Griffiths Mental Development Scales—Extended Revised: Two to Eight Years: Administration Manual. Hogrefe, Oxford, UK 2006.

[d]Achenbach TM. Manual for the Child Behavior Checklist/4-18 and 1991 profile. University of Vermont, Department of Psychiatry 1991.

[e]Khan NZ, Muslima H, Begum D, Shilpi AB, Akhter S, Bilkis K, Begum N, Parveen M, Ferdous S, Morshed R, Batra M. Validation of rapid neurodevelopmental assessment instrument for under-two-year-old children in Bangladesh. *Pediatrics* 2010; **125:** e755-62.

[f]Squires J, Bricker DD, Twombly E. Ages & stages questionnaires. Baltimore, MD: Paul H. Brookes 2009.

[g]Glascoe FP. Collaborating with parents: Using Parents' Evaluation of Developmental Status to detect and address developmental and behavioral problems. Ellsworth & Vandermeer Press 1998.

[h]McCoy DC, Waldman M, Team CF, Fink G. Measuring early childhood development at a global scale: Evidence from the Caregiver-Reported Early Development Instruments. *Early Childhood Research Quarterly* 2018; **45:** 58-68.

[i]Frankenburg WK, Dodds J, Archer P, Shapiro H, Bresnick B. The Denver II: a major revision and restandardization of the Denver Developmental Screening Test. *Pediatrics* 1992; **89:** 91-7.

[j]Ertem IO, Dogan DG, Gok CG, Kizilates SU, Caliskan A, Atay G, Vatandas N, Karaaslan T, Baskan SG, Cicchetti DV. A guide for monitoring child development in low-and middle-income countries. *Pediatrics* 2008; **121:** e581-9.

[*]Candidate tools contributing to the development of the INTERGROWTH-21st Neurodevelopment Assessment (INTER-NDA).

**S2** Factors associated with poor neurodevelopmental outcomes in young children[§#]: Characteristics of the normative sample for the INTERGROWTH-21st Project international INTER-NDA Standards, and for other neurodevelopmental tools evaluating two year-old children.

| **Factors affecting child development** | **INTERGROWTH-21st Project sample characteristics (N=1209)** | **Characteristics of normative samples, reported and accessible in the public domain, for the following child developmental tools:** | | | | | | | | | |
|---|---|---|---|---|---|---|---|---|---|---|---|
| | | **BSID III[a]** | **MDAT[b]** | **Griff-iths[c]** | **RNDA[d]** | **CBCL[e]** | **ASQ III[f]** | **PEDS[g]** | **CREDI[h]** | **DDST II[i]** | **GMCD[j]** |
| **PRENATAL FACTORS** | | | | | | | | | | | |
| **Genetic Factors:** | | | | | | | | | | | |
| Chromosomal abnormalities | Yes, n=0 (0.0%) | No | Yes^ | No | No | No | Yes^ | No | No | No | Yes^ |
| Genetic syndromes | Yes, n=0 (0.0%) | No | No | No | No | No | No | No | No | No | No |
| Sporadic mutations | Yes, n=0 (0.0%) | No | No | No | No | No | No | No | No | No | No |
| Gene polymorphisms | Yes, n=0 (0.0%) | No | No | No | No | No | No | No | No | No | No |
| Trinucleotide repeat disorders | Yes, n=0 (0.0%) | No | No | No | No | No | No | No | No | No | No |
| Metabolic disorders | Yes, n=0 (0.0%) | No | No | No | No | No | No | No | No | No | No |
| **Intrauterine (materno-feto-placental) factors:** | | | | | | | | | | | |
| Chronic maternal illness | Yes, n=12 (0.9%)[1] | No | No | No | No | No | No | No | No | No | No |
| Maternal infections (including HIV, rubella, syphilis, hepatitis B, CMV, toxoplasmosis, tuberculosis and malaria) | Yes, n=0 (0.0%) | No | No | No | No | No | No | No | No | No | No |
| Maternal anaemia | Yes, mean Hb 12.5 (SD1.0) | No | No | No | No | No | No | No | No | No | No |
| Maternal malignancy | Yes, n=0 (0.0%) | No | No | No | No | No | No | No | No | No | No |
| Poor maternal nutrition (under- and overnutrition) | Yes, mean BMI 23.4 (SD 7.5) | No | No | No | No | No | No | No | No | No | No |
| Maternal substance abuse (including alcohol) and smoking | Yes, n=0 (0.0%) | No | No | No | No | No | No | No | No | No | No |
| Teratogenic drugs | Yes, n=0 (0.0%) | No | No | No | No | No | No | No | No | No | No |
| Toxins (lead, mercury, and arsenic) | No | No | No | No | No | No | No | No | No | No | No |
| Anxiety and depression/mental stress | Yes, n=0 (0.0%) | No | No | No | No | No | No | No | No | No | No |
| Preeclampsia and eclampsia | Yes, n=0 (0.0%) | No | No | No | No | No | No | No | No | No | No |
| Placental structural anomalies | Yes, n=0 (0.0%) | No | No | No | No | No | No | No | No | No | No |
| Liquor volume (oligo and polyhydramnios) | Yes, n=28 (2.3%) | No | No | No | No | No | No | No | No | No | No |
| Intrauterine infections (prolonged rupture of membranes and chorioamnionitis) | Yes, n=145 (11.9%) | No | No | No | No | No | No | No | No | No | No |
| Fetal behaviour | No | No | No | No | No | No | No | No | No | No | No |
| Fetal growth restriction | Yes, n=0 (0.0%) | No | No | No | No | No | No | No | No | No | Yes^ |
| Multiple gestation | Yes, n=0 (0.0%) | No | No | No | No | No | Yes^ | No | No | No | Yes |
| **External factors :** | | | | | | | | | | | |
| Maternal access to health care | Yes, n=1209 | No | No | No | No | No | No | No | No | No | Yes |

| | | | | | | | | | | | |
|---|---|---|---|---|---|---|---|---|---|---|---|
| | (100.0%) | | | | | | | | | | Yes |
| Maternal access to prenatal care | Yes, n=1209 (100.0%) | No | No | No | No | No | No | No | No | No | No |
| Exposure to radiation | No | No | No | No | No | No | No | No | No | No | No |
| Trauma | Yes, n=0 (0.0%) | No | No | No | No | No | No | No | No | No | Yes |
| **Socio-Maternal:** | | | | | | | | | | | |
| SES/Poverty | Yes | Yes | No | No | Yes | No | Yes | No | Yes | No | Yes |
| Hunger due to lack of money to buy food | Yes, n=0 (0.0%) | No | No | No | No | No | Yes | No | Yes | No | Yes^ |
| Maternal education | Yes[2] | Yes | No | No | Yes | No | Yes | No | Yes | No | No |
| Paternal education | No | No | No | No | Yes | No | No | No | No | No | No |
| Maternal age at the time of delivery | Yes; mean=28.9 years (SD 3.8) | No | No | No | No | No | Yes | No | No | No | No |
| Involvement of social services/child protection agencies with family | No | No | No | No | No | No | Yes | No | No | No | No |
| Domestic violence or abuse | No | No | No | No | No | No | Yes | No | No | No | No |
| State of local/regional violence | Yes | No | No | No | No | No | Yes | No | No | No | No |
| Maternal employment in very physically demanding work during pregnancy | Yes, n=0 (0.0%) | No | No | No | No | No | No | No | No | No | No |
| Maternal occupation with risk of exposure to chemicals or toxic substances | Yes, n=0 (0.0%) | No | No | No | No | No | No | No | No | No | No |
| **BIRTH FACTORS** | | | | | | | | | | | |
| Prematurity (born at <37 weeks' gestation) | Yes, n=0 (0.0%) | No | Yes | No | No | No | Yes | No | No | No | Yes |
| Birth weight <2500 g | Yes, n=43 (3.5%) | No | No | No | No | No | Yes | No | No | No | Yes |
| Fetal distress prior to birth as adjudged by fetal heart rate monitoring and/or cord blood sampling | Yes, n=64 (5.3%) | No | No | No | No | No | No | No | No | No | Yes^ |
| Resuscitation of newborn | Yes, n=36 (2.9%) | No | No | No | No | No | No | No | No | No | Yes^ |
| **POSTNATAL FACTORS** | | | | | | | | | | | |
| **Maternal Factors:** | | | | | | | | | | | |
| Maternal mental health/maternal depression | Yes, n=0 (0.0%) | No | No | No | No | No | No | No | No | No | No |
| Maternal exposure to abuse and violence | Yes, n=0 (0.0%) | No | No | No | No | No | Yes | No | No | No | No |
| Parenting style: cognitive stimulation, caregiver sensitivity and responsiveness to the child, and caregiver affect (emotional warmth or rejection of child) | No | No | No | No | No | No | No | No | Yes | No | No |
| **Neonatal Factors:** | | | | | | | | | | | |
| Neonatal sepsis, including congenital and intracranial infections | Yes, n=7 (0.6%) | No | No | No | No | No | Yes^ | No | No | No | Yes^ |
| Neonatal seizures | Yes, n=0 (0.0%) | No | No | No | No | No | Yes^ | No | No | No | Yes^ |

| | | | | | | | | | | | |
|---|---|---|---|---|---|---|---|---|---|---|---|
| Prolonged ventilation | Yes, n=0 (0.0%) | No | No | No | No | No | Yes^ | No | No | No | Yes^ |
| Hypotension requiring inotropic support | Yes, n=0 (0.0%) | No | No | No | No | No | Yes^ | No | No | No | Yes^ |
| Intraventricular haemorrhage grade 2 or greater | Yes, n=0 (0.0%) | No | No | No | No | No | Yes^ | No | No | No | Yes^ |
| Hypoxic ischaemic encephalopathy | Yes, n=1 (0.1%) | No | No | No | No | No | Yes^ | No | No | No | Yes^ |
| Developmental abnormalities | Yes, n=7 (0.6%) | No | No | No | No | No | Yes^ | No | No | No | Yes^ |
| Cardiac diagnosis | Yes, n=1 (0.1%)[3] | No | No | No | No | No | Yes^ | No | No | No | Yes^ |
| Hyperbilirubinaemia, not requiring exchange transfusion | Yes, n=49 (4.0%) | No | No | No | No | No | Yes^ | No | No | No | Yes^ |
| Use of medications, including antibiotics, in neonatal period | Yes, n=141 (11.7%) | No | No | No | No | No | Yes^ | No | No | No | Yes^ |
| Breastfeeding at postnatal discharge from hospital | Yes, n=1123 (92.9%) | No | No | No | No | No | Yes^ | No | No | No | Yes^ |
| **Infant and Toddler Factors:** | | | | | | | | | | | |
| Adequate physical growth measured as per standardised protocols and growth charts | Yes, n=1209 (100.0%) | No | Yes | No | Yes | No | No | No | Yes | No | Yes |
| Breastfeeding and appropriate weaning | Yes[4] | No | No | No | No | No | No | No | No | No | Yes^ |
| Micronutrient deficiencies, including iron, iodine and zinc | Yes, n=1 (0.1%) | No | No | No | No | No | Yes^ | No | No | No | Yes |
| Severe infectious diseases (HIV, malaria, tuberculosis, meningitis) | Yes, n=0 (0.0%) | No | No | No | No | No | Yes | No | No | No | Yes |
| Cerebral palsy | Yes, n=0 (0.0%) | No | No | No | No | No | Yes^ | No | No | No | Yes^ |
| Neurological disorders | Yes, n=0 (0.0%) | No | No | No | No | No | Yes^ | No | No | No | Yes^ |
| Seizures | Yes, n=0 (0.0%) | No | No | No | No | No | Yes^ | No | No | No | Yes^ |
| Long-term health issues including metabolic, endocrinological and surgical conditions | Yes, n=0 (0.0%) | No | No | No | No | No | Yes^ | No | No | No | Yes^ |
| Exposure to environmental toxins such as lead, arsenic, manganese and pesticides | Yes, varies (<10%)[k] | No | No | No | No | No | No | No | No | No | No |
| Exposure to radiation | Yes, varies (<10%)[k] | No | No | No | No | No | No | No | No | No | No |
| Exposure to social adversity (e.g. neighbourhood crime) | Yes, varies (<10%)[k] | No | No | No | No | No | No | No | No | No | No |
| Trauma, including road traffic accidents and non-accidental injury | Yes, n=0 (0.0%) | No | Yes | No | No | No | Yes | No | No | No | No |
| Significant health conditions during the first 2 years of life, requiring prolonged hospitalisation | Yes, n=0 (0.0%) | No | Yes | No | No | No | Yes^ | No | No | No | Yes^ |
| Delayed acquisition of developmental milestones or neurosdisability as reported by parents/healthcare workers | Yes, n=0 (0.0%) | No | No | No | No | Yes | Yes^ | No | Yes^ | No | Yes^ |
| Parental concerns | Yes, n=0 (0.0%) | No | No | No | No | Yes | Yes^ | No | No | No | Yes^ |

BSID III: The Bayley Scales of Infant Development – III edition (BSID III)
MDAT: The Malawi Developmental Assessment Tool
Griffiths: The Griffiths Mental Development Scales
RNDA: The Rapid Neurodevelopmental Assessment

CBCL: Pre-school version of Child Behavior Checklist
ASQ III: The Ages and Stages Questionnaire III edition
PEDS: The Parents' Evaluation of Developmental Status
CREDI: Caregiver-reported Early Developmental Instruments
DDST II: Denver Development Screening Test II
GMCD: Guide for Monitoring Child Development

Yes: Factor in column 1 has been reported in published literature about the tool's normative sample, or has been stated, in published literature, to have been considered in the evaluation of the tool's normative sample
No: Factor in column 1 has not been reported in published literature about the tool's normative sample, or has not been stated, in published literature, to have been considered in the evaluation of the tool's normative sample
^Implied as stated as "healthy" and "low risk", specific descriptions of each predictor not stated.
[§] Walker SP, Wachs TD, Gardner JM, Lozoff B, Wasserman GA, Pollitt E, Carter JA, International Child Development Steering Group. Child development: risk factors for adverse outcomes in developing countries. *The lancet* 2007; **369:** 145-57.
[#] Fernandes M, Srinivasan K, Menezes G, Ramchandani PG. Prenatal depression, fetal neurobehavior, and infant temperament: Novel insights on early neurodevelopment from a socioeconomically disadvantaged Indian cohort. *Development and Psychopathology* 2018; **30:** 725-42.

[1]Chronic respiratory illness, including asthma n=4 (0·3%), endocrinological conditions including hypothyroidism n=7(0·6%), other n=1 (0·08%)
[2]Primary education n=62 (5·1%); Secondary education n=281 (23·2%); Professional/technical training n=198 (16·4%); University n=668 (55·3%)
[3]Patent ductus arteriosus
[4]Duration of exclusive breastfeeding: median 5.6 months (IQR 4.0, 6.0); Age of introduction of formula feeds: median 4.2 months (IQR 2.0, 6.0); age at introduction of first solids; median 6.0 months (IQR 5.5, 6.5)

[a]Pearson. Bayley Scales of Infant and Toddler Development, Third Edition (Bayley-III) - Training. Available from: 98 http://www.pearsonclinical.co.uk
[b]Gladstone M, Lancaster GA, Umar E, Nyirenda M, Kayira E, van den Broek NR, Smyth RL. The Malawi Developmental Assessment Tool (MDAT): the creation, validation, and reliability of a tool to assess child development in rural African settings. *PLoS medicine* 2010; **7:** e1000273.
[c]Luiz D, Barnard A, Knoesen N, Kotras N, Horrocks S, McAlinden P, Challis D, O'Connell R. Griffiths Mental Development Scales—Extended Revised: Two to Eight Years: Administration Manual. Hogrefe, Oxford, UK 2006.
[d]Achenbach TM. Manual for the Child Behavior Checklist/4-18 and 1991 profile. University of Vermont, Department of Psychiatry 1991.
[e]Khan NZ, Muslima H, Begum D, Shilpi AB, Akhter S, Bilkis K, Begum N, Parveen M, Ferdous S, Morshed R, Batra M. Validation of rapid neurodevelopmental assessment instrument for under-two-year-old children in Bangladesh. *Pediatrics* 2010; **125:** e755-62.
[f]Squires J, Bricker DD, Twombly E. Ages & stages questionnaires. Baltimore, MD: Paul H. Brookes 2009.
[g]Glascoe FP. Collaborating with parents: Using Parents' Evaluation of Developmental Status to detect and address developmental and behavioral problems. Ellsworth & Vandermeer Press 1998.
[h]McCoy DC, Waldman M, Team CF, Fink G. Measuring early childhood development at a global scale: Evidence from the Caregiver-Reported Early Development Instruments. *Early Childhood Research Quarterly* 2018; **45:** 58-68.
[i]Frankenburg WK, Dodds J, Archer P, Shapiro H, Bresnick B. The Denver II: a major revision and restandardization of the Denver Developmental Screening Test. *Pediatrics* 1992; **89:** 91-7.
[j]Ertem IO, Dogan DG, Gok CG, Kizilates SU, Caliskan A, Atay G, Vatandas N, Karaaslan T, Baskan SG, Cicchetti DV. A guide for monitoring child development in low-and middle-income countries. *Pediatrics* 2008; **121:** e581-9.
[k]Eskenazi B, Bradman A, Finkton D, Purwar M, Noble JA, Pang R, Burnham O, Cheikh Ismail L, Farhi F, Barros FC, Lambert A. A rapid questionnaire assessment of environmental exposures to pregnant women in the INTERGROWTH-21st Project. *BJOG: An International Journal of Obstetrics & Gynaecology* 2013; **120:** 129-38.

**S3** The INTERGROWTH-21st Fetal Growth Longitudinal Study (FGLS) individual participant entry criteria for mothers at the time of antenatal booking in the first trimester of pregnancy

| **Maternal eligibility criteria at booking (<14 weeks of gestation)** | |
|---|---|
| 1. | Aged ≥18 and <35 years. |
| 2. | Body mass index ≥18.5 and <30 kg/m$^2$. |
| 3. | Height ≥153 cm. |
| 4. | Singleton pregnancy. |
| 5. | A known last menstrual period with regular cycles (defined as 28 ± 4 days) without hormonal contraceptive use, or breastfeeding in the 2 months before pregnancy. |
| 6. | Natural conception. |
| 7. | No relevant past medical history, with no need for long-term medication (including fertility treatment and over-the-counter medicines, but excluding routine iron, folate, calcium, iodine or multivitamin supplements). |
| 8. | No evidence of socio-economic constraints likely to impede fetal growth identified using local definitions of social risk. |
| 9. | No use of tobacco or recreational drugs such as cannabis in the 3 months before or after becoming pregnant. |
| 10. | No heavy alcohol use (defined as >5 units (50 ml pure alcohol) per week) since becoming pregnant. |
| 11. | No more than one miscarriage in the two previous consecutive pregnancies. |
| 12. | No previous baby delivered preterm (<$37^{+0}$ weeks of gestation) or with a birthweight <2500 g or >4500 g. |
| 13. | No previous neonatal or fetal death, previous baby with any congenital malformations, and no evidence in present pregnancy of congenital disease or fetal anomaly. |
| 14. | No previous pregnancy affected by pre-eclampsia/eclampsia, HELLP syndrome or a related pregnancy-associated condition. |
| 15. | No clinically significant atypical red cell alloantibodies. |
| 16. | Negative urinalysis. |
| 17. | Systolic blood pressure <140 mmHg and diastolic blood pressure <90 mmHg. |
| 18. | No diagnosis or treatment for anaemia during this pregnancy (haemoglobin levels will be monitored throughout pregnancy). |
| 19. | No clinical evidence of any other sexually transmitted diseases, including syphilis and clinical trichomoniasis. |
| 20. | Not in an occupation with risk of exposure to chemicals or toxic substances, or very physically demanding activity to be evaluated by local standards. Also women should not be conducting vigorous or contact sports, such as scuba diving or similar activities. |

**S4** The INTERGROWTH-21[st] Neurodevelopment Assessment (INTER-NDA) data recording form

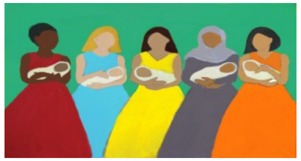

# The INTERGROWTH-21st Neurodevelopment Assessment
# INTER-NDA

Name of child: ______________________ Date of birth: __ /__ / _____ Date of assessment: __ /__ / _____

Name of assessor: ____________________

| No. | Item | Observed Performance | | | | |
|---|---|---|---|---|---|---|
| 1 | **Builds a tower of 5 cubes**<br>*(trials=3, demonstration=3)* | 5 cubes | 3-4 cubes | 2 cubes | No attempt | Unable to assess |
| 2 | **Names 4 colours when asked to do so**<br>*(trials=1, demonstration=0)* | Names 4 colours | Names 3 colours | Names 1 or 2 colours | Does not name any colour | Unable to assess |
| 3 | **Matches 3 cubes of same colours when requested to do so**<br>(trials=1, demonstration=1 of one colour) | Matches 3 colours | Matches 2 colours | Matches 1 colour | Does not match any colour | Unable to assess |
| 4 | **Hands the examiner one cube when asked to do so (Examiner says "Please give me one cube" & keeps palm open for 5 seconds after child has handed over 1 cube)**<br>*(trials=1, demonstration=0)* | Hands only one block within 5 seconds | Hands only one block in more than 5 seconds | Hands two or more blocks | Does not hand any block / No attempt | Unable to assess |
| 5 | **Puts the spoon in the cup when asked to do so**<br>*(trials=5, demonstration=0)* | Puts the spoon in cup in ≤3 trials | Puts the spoon in cup in 4-5 trials | Takes the spoon or the cup but does not complete action | No attempt | Unable to assess |

| | | | | | | |
|---|---|---|---|---|---|---|
| 6 | **Matches shapes on board**<br>*(trials=5, demonstration=partial – removal only)* | All shapes in ≤3 trials | All shapes with repeated demonstration i.e. 4-5 trials | One or two shapes in 4-5 trials | No attempt | Unable to assess |
| 7 | **Matches shapes on rotated board**<br>*(trials=5, demonstration=partial – removal only)* | All shapes in ≤3 trials | All shapes with repeated demonstration i.e. 4-5 trials | One or two shapes in 4-5 trials | No attempt | Unable to assess |
| 8 | **Points correctly when asked "Where is the door/entrance to the room?"**<br>*(trials=5, demonstration=0)* | Identifies door correctly in ≤3 trials | Identifies door correctly in 4-5 trials | Attempts, but does not identify door | No attempt | Unable to assess |
| 9 | **Puts a raisin precisely inside a small opening in a bottle**<br>*(trials=1, demonstration=1, test both hands)* | Precise release of raisin into bottle with each hand | Clumsy release, raisin falls out of bottle with one or more hand | Attempts but unsuccessful release with one or more hand | No attempt | Unable to assess |
| 10 | **Drinks water from cup/bottle/sippy cup when placed in front of child**<br>*(trials=1, demonstration=0; maternal recall if observation not possible)* | Drinks water from cup/sippy cup without spilling | Drinks clumsily & spills | Attempts but unsuccessful | No attempt | Unable to assess |
| 11 | **Looks towards an object located across the room when pointed at by the examiner**<br>(trials=5) | Looks or points at object in ≤3 trials | Looks or points at object in 4-5 trials | Looks at the wrong object, or attempts but cannot identify object | No attempt | Unable to assess |
| 12 | **Pretends to drink from a toy cup when placed in front of him/her**<br>*(trials=2, demonstration=1 if not spontaneous on first attempt)* | Spontaneously | After 1 demonstration | Partial attempt after 1 demonstration | No attempt | Unable to assess |
| 13 | **Able to make a cup of tea with the toy tea set when requested by examiner (Examiner says "Can you make a cup of tea?")**<br>*(trials=2, demonstration=1 if not spontaneous on first attempt)* | Spontaneously, with pouring motion | After 1 demonstration | Partial attempt after 1 demonstration | No attempt | Unable to assess |

| | | | | | | |
|---|---|---|---|---|---|---|
| 14 | **Feeds doll when requested to (Examiner says "Can you give the dolly some tea?")** *(trials=2, demonstration=1 if not spontaneous on first attempt)* | Spontaneously | After 1 demonstration | Partial attempt after 1 demonstration | No attempt | Unable to assess |
| 15 | **Imitates straight horizontal scribble** *(trials=5, demonstration=5)* | ≤3 trials | 4-5 trials; with difficulty | Attempts (hold crayon) | Cannot hold crayon | Unable to assess |
| 16 | **Identifies glitter bracelet under correct washcloth** *(trials=5, demonstration=0, test both sides)* | Finds bracelet correctly in ≤2 trails on both sides | Find bracelet correctly in 3 trials or on one side only | Find bracelet correctly in 4-5 trials or on one side only | Does not find bracelet or no attempt | Unable to assess |
| 17 | **Correctly identifies object groups using plurals** *(concurrent observation)* | Uses 5 plurals | Uses 3-4 plurals | Uses 1-2 plurals | Does not use any plurals | Unable to assess |
| 18 | **Asks for toilet by gesture or verbally** *(maternal recall)* | Always | Occasionally | Partial (only for bowel movement) | Never | Unable to assess |
| 19 | **Runs** *(maternal recall)* | Runs steadily | Attempts | Walks only | Walks with support | Unable to assess |
| 20 | **Throws a ball very near** *(trials=1, demonstration=1; test both hands)* | Good release | Unsteady release | Attempts | No attempt | Unable to assess |
| 21 | **Kicks ball** *(maternal recall)* | Kicks ball with knee flexed | Runs after ball & attempts kicking it | Walks and touches ball with foot | No attempt | Unable to assess |
| 22 | **Climbs upstairs holding rail, 2 feet/stair or in adult fashion** *(maternal recall)* | Climbs stairs alone steadily | Climbs stairs alone unsteadily | Climbs stairs with help (uses railing, holds adult's hand) | No attempt | Unable to assess |
| 23 | **Uses 2-4 syllable babble such as dada, mama but not specifically to anything or any person** *(concurrent observation)* | Spontaneously | Mimics | 1 syllabe babble e.g. ba, ma, da | None | Unable to assess |
| 24 | **Use two words together** *(concurrent observation)* | Two words, appropriate use | Two words, inappropriate use | One word, appropriate use | No attempt | Unable to assess |

| | | | | | | |
|---|---|---|---|---|---|---|
| 25 | **Indicates by gesture to say no**<br>*(concurrent observation or maternal recall)* | Indicates verbally or by definite gesture all the time | Indicates verbally or by definite gesture some of the time | Attempts, but incomplete indication | No attempt | Unable to assess |
| 26 | **Use of a pronoun e.g. me, my, she, he, it, I**<br>*(concurrent observation)* | ≥1 pronoun in correct context | ≥1 pronoun, incorrect use | Use of proper names but not pronouns | No use | Unable to assess |
| 27 | **How many words does the child use during the assessment other than mama/dada**<br>*(concurrent observation)* | ≥8 words | 6-7 words | 4-5 words | ≤3 words | Unable to assess |
| 28 | **How many sentences of 3 words or more does the child use during the assessment?**<br>*(concurrent observation)* | ≥2 | 1 | ≥1 two word utterance | None | Unable to assess |
| 29 | **In how many instances does the child follow on a topic of conversation providing new information?**<br>*(concurrent observation)* | At least one, using ≥ 2 words, proving correct information | At least one, uses single words, provides correct information | Uses any number of words, provides incorrect information | Does not follow up on conversations | Unable to assess |
| 30 | **Combines word and gesture when asked**<br>*(Do not demonstrate, trials=3, use different example if mother says child does not know the one you are asking)* | Combines word and gesture completely and appropriately | Combines word and gesture completely but inappropriately | Combines word and gesture incompletely and inappropriately | Does not combine a word an gesture | Unable to assess |

What is the child's native (first) language? ________________________________________________________________
What is the language in which the assessment is being conducted in? ____________________________________________________
Does the child speak/understand any languages other than his/her native (first) language? ______________________________________

| How often were the following behaviours in the child during the assessment? | | | | |
|---|---|---|---|---|
| 31 | **Positive Affect** | Never or rarely | Some of the time | Most of the time |
| 32 | **Exploration** | Never or rarely | Some of the time | Most of the time |
| 33 | **Ease of engagement** | Never or rarely | Some of the time | Most of the time |
| 34 | **Cooperativeness** | Never or rarely | Some of the time | Most of the time |
| 35 | **Adaptability to change** | Never or rarely | Some of the time | Most of the time |
| 36 | **Distractibility** | Never or rarely | Some of the time | Most of the time |
| 37 | **Negative Affect** | Never or rarely | Some of the time | Most of the time |

**S5** The INTERGROWTH-21st Neurodevelopment Assessment (INTER-NDA) Protocol Adherence Checklist

| | The INTERGROWTH-21st Project<br>Neurodevelopment Assessment Package<br>**Protocol Adherence Checklist** | **NDA-PAC**<br>**Page 2 of 2** |
|---|---|---|

| | | | |
|---|---|---|---|
| **Participant ID No.** | __ __ - __ __ __ __ __ | **Researcher code: Expert** | |
| **Child Date of Birth** | D D M M Y Y | **Researcher code: Assessor** | |
| **Visit Date** | D D M M Y Y | | |

**Please rate the performance of the assessor in adhering to the INTERGROWTH-21st Neurodevelopment Assessment Protocol during the administration of the following items. For the purpose of this exercise, complete adherence is defined as adherence to all aspects of the protocol as stated in the operation manual of the INTER-NDA; partial adherence is defined as adherence to half or more aspects, but not all aspects, of the protocol as stated in the operation manual of the INTER-NDA and limited adherence is defined as adherence to less than half of the aspects of the protocol as stated in the operation manual of the INTER-NDA as adjudged by the expert assessor.**

| **IV. The INTER-NDA** | Complete adherence | Partial adherence | Limited adherence |
|---|---|---|---|
| 1. Item 1 - uses 5 red cubes, 3 trials, demonstration before each trial | | | |
| 2. Item 2 - 4 cubes of different colours placed in a line, assessor does not name colours, 1 trial | | | |
| 3. Item 3 - 1 demonstration, 1 trial, conceptually correct scoring (stacking & pointing accepted) | | | |
| 4. Item 4 - holds hand out for 5 seconds | | | |
| 5. Item 5 - correct placement of objects - handles facing child, cup & spoon not adjacent, 5 trials | | | |
| 6. Item 6 - 5 trials, no demonstration, shapes near child, holds board, puts shapes back discretely | | | |
| 7. Item 7 - 5 trials, no demonstration, shapes near child, rotates board on table, holds board | | | |
| 8. Item 9 - 1 demonstration, 1 trial, tests both hands | | | |
| 9. Item 11 - 5 trials, no demonstration, relatively slow pointing movement so child can follow | | | |
| 10. Item 12 - allows time for self-symbolic play before suggestion & demonstration | | | |
| 11. Item 13 - 2 trials, demonstrate if not spontaneous, involve mother if necessary | | | |
| 12. Item 14 - 2 trials, demonstrate if not spontaneous, involve mother if necessary | | | |
| 13. Item 15 - 5 trials, demonstration before each trial, start at upper part of paper | | | |
| 14. Item 16 - 5 trials, displaces bracelet relatively slowly, tests both sides | | | |
| 15. Item 20 - 1 demonstration, 1 trial, tests both hands | | | |
| 16. Item 30 - uses opportunities during assessment, uses culturally appropriate combinations, informs mothers about purpose & rationale of item | | | |
| 17. Assessor's interaction with the child (good verbal & nonverbal communication, sets child at ease, builds rapport, accomodates needs of the child) | | | |
| 18. Assessor's interaction with the mother/caregiver (explains tests, builds rapport, involves mother in testing providing instructions to prevent compromising test) | | | |

| | |
|---|---|
| Name of expert researcher | |
| Signature | |

**S6** Results of the INTERGROWTH-21st Neurodevelopment Assessment (INTER-NDA) standardized evaluations between specialist and non-specialist assessors to (a) administer and (b) score the INTER-NDA

S6 (a) Comparisons in protocol adherence scores for the INTER-NDA between non-specialist and specialist assessors.

| | Median (95% CI) | | Comparison between groups |
|---|---|---|---|
| | **Non-specialist assessors** (n=4) | **Specialist assessors** (n=3) | |
| **INTER-NDA Protocol Adherence Score (Total Range: 18 – 54)** | 53.0 (43.4 – 53.6) | 52.0 (46.2 – 54.2) | U=27.5, p=0.8 |
| **INTER-NDA Protocol Adherence %** | 98.0 (80.4 – 99.2) | 96.3 (85.5 – 99.3) | U=30.5, p=1.0 |

S6 (b) Comparisons in INTER-NDA domain scores between non-specialist and specialist assessors.

| | | Neurodevelopment scores (n=23) | | | | | |
|---|---|---|---|---|---|---|---|
| | | **Cognition** | **Language** | **Gross motor** | **Fine motor** | **Positive behavior** | **Negative behavior** |
| | | ***Mean (SD)*** | ***Mean (SD)*** | ***Mean (SD)*** | ***Mean (SD)*** | ***Mean (SD)*** | ***Mean (SD)*** |
| **Video/Child 1** | **Field workers** (n=10) | 21.1 (2.0)[a] | 17.7 (3.7) | 4.6 (0.9) | 4.6 (0.9) | 14.9 (0.3) | 2.4 (0.7) |
| | **Healthcare professionals** (n=13) | 24.2 (2.2)[a] | 17.1 (3.1) | 5.1 (0.8) | 5.1 (1.0) | 14.5 (0.9) | 2.3 (0.5) |
| **Video/Child 2** | **Field workers** (n=10) | 19.4 (1.7) | 31.4 (2.7) | 3.2 (0.4) | 4.0[§] | 12.7 (1.3) | 3.8 (0.4) |
| | **Healthcare professionals** (n=13) | 19.5 (1.4) | 30.9 (3.1) | 5.5 (2.6) | 4.5 (1.0) | 12.5 (1.3) | 3.9 (0.5) |
| **Video/Child 3** | **Field workers** (n=10) | 21.9 (2.0) | 25.0 (2.9) | 4.0 (0.9) | 7.6 (0.7) | 12.4 (1.3) | 3.3 (0.5) |
| | **Healthcare professionals** (n=13) | 22.6 (1.9) | 26.8 (5.3) | 3.9 (0.8) | 5.7 (1.4) | 11.0 (1.6) | 3.9 (0.7) |

[a]t=-3.2, p<0.001. [§]As score for this group is identical for all test subjects within this group no SD has been reported

**S7** The INTERGROWTH-21st Neurodevelopment Assessment (INTER-NDA) scoring system and interpretation of domain scores.

| **INTER-NDA domain** | Number of items contributing to domain | Constituent item numbers | Method of domain estimation | Interpretation of score |
|---|---|---|---|---|
| Cognitive | 13 | 1,2,4,5,6,7,8, 11,12,13,14,16,18 | Mean of constituent item scores | Higher score reflects better performance |
| Fine motor | 4 | 9,10,15,20 | Mean of constituent item scores | Higher score reflects better performance |
| Gross motor | 3 | 19,21,22 | Mean of constituent item scores | Higher score reflects better performance |
| Language | 12 | 3,5,8,17,23,24,25,26,27,28,29,30 | Mean of constituent item scores | Higher score reflects better performance |
| Positive behaviour | 5 | 31,32,33,34,35 | Mean of constituent item scores | Higher score reflects better performance |
| Negative behaviour | 2 | 36,37 | Mean of constituent item scores | Lower score reflects better performance |

**S8** Formulae and tables for the conversion of raw scores to standardised (scaled) scores (range 0-100)

| **Domain** | **Min - Max** | **Scaling formula** |
|---|---|---|
| Cognitive, fine motor, gross motor and language | 1 – 4 | ( (x – 1) / 3) ) * 100 |
| Positive and negative behaviour | 1 – 3 | ( (x – 1) / 2) ) * 100 |

| **Domain conversion table (selected values) for cognitive, motor (fine and gross), and language domains** | |
|---|---|
| **Raw mean score** | **Scaled mean score** |
| 1.00 | 0.0 |
| 1.25 | 8.3 |
| 1.50 | 16.7 |
| 1.75 | 25.0 |
| 2.00 | 33.3 |
| 2.25 | 41.7 |
| 2.50 | 50.0 |
| 2.75 | 58.3 |
| 3.00 | 66.7 |
| 3.25 | 75.0 |
| 3.50 | 83.3 |
| 3.75 | 91.7 |
| 4.00 | 100.0 |

| **Domain conversion table (selected values) for positive and negative** | |
|---|---|
| **Raw mean score** | **Scaled mean score** |
| 1.0 | 0.0 |
| 1.2 | 10.0 |
| 1.4 | 20.0 |
| 1.6 | 30.0 |
| 1.8 | 40.0 |

| | |
|---|---|
| 2.0 | 50.0 |
| 2.2 | 60.0 |
| 2.4 | 70.0 |
| 2.6 | 80.0 |
| 2.8 | 90.0 |
| 3.0 | 100.0 |

**Supplementary Information S9** INTER-NDA domain scores centiles including and excluding children scoring above the CBCL threshold for clinical (97th centile) problems.

| INTER-NDA domain | Pooled Centiles for children including children scoring above the 97th CBCL centiles for attentional problems and/or emotional reactivity (n=1209) | | | | | | | Pooled Centiles for children excluding children scoring above the 97th CBCL centiles for attentional problems and/or emotional reactivity (n=1181) | | | | | | |
|---|---|---|---|---|---|---|---|---|---|---|---|---|---|---|
| | **c3** | **c10** | **c25** | **c50** | **c75** | **c90** | **c97** | **c3** | **c10** | **c25** | **c50** | **c75** | **c90** | **c97** |
| **Cognitive[1]** | 27.3 | 38.5 | 60.9 | 79.2 | 88.2 | 92.6 | 99.6 | 27.4 | 38.5 | 62.2 | 79.5 | 88.8 | 92.6 | 99.6 |
| **Fine motor[1]** | 17.4 | 25.2 | 71.5 | 90.4 | 100.0 | 100.0 | 100.0 | 17.5 | 25.7 | 74.2 | 91.4 | 100.0 | 100.0 | 100.0 |
| **Gross motor[1]** | 31.9 | 51.6 | 66.7 | 81.5 | 100.0 | 100.0 | 100.0 | 31.1 | 51.7 | 66.7 | 81.6 | 100.0 | 100.0 | 100.0 |
| **Language[1]** | 12.1 | 17.0 | 44.6 | 70.7 | 88.0 | 94.9 | 100.0 | 12.1 | 17.8 | 45.7 | 71.7 | 88.5 | 95.1 | 100.0 |
| **Positive behaviour[1]** | 32.8 | 50.3 | 69.7 | 90.0 | 100.0 | 100.0 | 100.0 | 37.8 | 51.4 | 70.0 | 90.0 | 100.0 | 100.0 | 100.0 |
| **Negative behaviour[2]** | 0.0 | 0.0 | 0.0 | 25.0 | 25.1 | 50.1 | 83.0 | 0.0 | 0.0 | 0.0 | 25.0 | 25.0 | 50.0 | 76.5 |

INTER-NDA: The INTERGROWTH-21st Neurodevelopment Assessment
[1]For these domains, higher scores reflect better outcomes
[2]For negative behaviour, lower scores reflect better outcomes

**S10** Comparison of INTERGROWTH-21st Neurodevelopment Assessment (INTER-NDA) domain scores between two year-old girls and boys

| | **Centiles for girls (n=617)** | | | | | | | **Centiles for boys (n=564)** | | | | | | | **Girls (n=617)** | **Boys (n=564)** | |
|---|---|---|---|---|---|---|---|---|---|---|---|---|---|---|---|---|---|
| **INTER-NDA Domain** | **c3** | **c10** | **c25** | **c50** | **c75** | **c90** | **c97** | **c3** | **c10** | **c25** | **c50** | **c75** | **c90** | **c97** | **Median (IQR)** | **Median (IQR)** | **p-value*** |
| **Cognitive[1]** | 27.2 | 38.9 | 64.3 | 81.7 | 89.7 | 93.0 | 99.5 | 27.0 | 38.2 | 58.3 | 76.9 | 86.8 | 92.6 | 99.2 | 82.1 (64.1, 89.7) | 76.9 (59.0, 87.2) | 0.001 |
| **Fine motor[1]** | 18.7 | 28.3 | 74.6 | 91.6 | 100.0 | 100.0 | 100.0 | 17.6 | 25.3 | 68.1 | 86.0 | 100.0 | 100.0 | 100.0 | 91.7 (75.0, 100.0) | 83.3 (66.7, 100.0) | 0.062 |
| **Gross motor[1]** | 27.6 | 52.7 | 66.7 | 83.1 | 100.0 | 100.0 | 100.0 | 32.4 | 52.5 | 66.7 | 79.6 | 100.0 | 100.0 | 100.0 | 83.3 (66.7, 100.0) | 77.8 (66.7, 100.0) | 0.318 |
| **Language[1]** | 13.9 | 22.1 | 49.5 | 75.7 | 90.1 | 97.1 | 100.0 | 11.6 | 16.8 | 40.0 | 65.6 | 85.2 | 93.9 | 100.0 | 75.8 (50.0, 90.0) | 66.7 (38.9, 86.1) | <0.001 |
| **Positive behaviour[1]** | 34.1 | 51.7 | 70.4 | 90.0 | 100.0 | 100.0 | 100.0 | 39.5 | 52.4 | 68.2 | 90.0 | 100.0 | 100.0 | 100.0 | 90.0 (70.0, 100.0) | 90.0 (70.0, 100.0) | 0.100 |
| **Negative behaviour[2]** | 0.0 | 0.0 | 0.0 | 16.1 | 25.0 | 50.0 | 75.6 | 0.0 | 0.0 | 0.0 | 25.0 | 29.4 | 50.6 | 83.6 | 25.0 (0.0, 25.0) | 25.0 (0.0, 25.0) | <0.001 |

INTER-NDA: The INTERGROWTH-21st Neurodevelopment Assessment
*p value from Wilcoxon rank-sum test
[1]For these domains, higher scores reflect better outcomes
[2]For negative behaviour, lower scores reflect better outcomes
IQR: inter-quartile range
