# Supplementary material for: INTERGROWTH-21st Project international INTER-NDA standards for child development at 2 years of age: an international prospective population-based study
Source: BMJ Open. 2020 Jun 7;10(6):e035258. doi: 10.1136/bmjopen-2019-035258 (PMC7282399; doi:10.1136/bmjopen-2019-035258)
Supplement: Author's manuscript [file bmjopen-2019-035258.draft_revisions.pdf]

BMJ Open is committed to open peer review. As part of this commitment we make the peer review history of every article we publish publicly available.

When an article is published we post the peer reviewers' comments and the authors' responses online. We also post the versions of the paper that were used during peer review. These are the versions that the peer review comments apply to.

The versions of the paper that follow are the versions that were submitted during the peer review process. They are not the versions of record or the final published versions. They should not be cited or distributed as the published version of this manuscript.

BMJ Open is an open access journal and the full, final, typeset and author-corrected version of record of the manuscript is available on our site with no access controls, subscription charges or pay-per-view fees (http://bmjopen.bmj.com).

If you have any questions on BMJ Open's open peer review process please email info.bmjopen@bmj.com

BMJ Open

The INTERGROWTH-21st Project International INTER-NDA standards for child development at two years of age: An International Prospective Population-based Study

Journal:	*BMJ Open*
Manuscript ID	bmjopen-2019-035258
Article Type:	Original research
Date Submitted by the Author:	26-Oct-2019

Complete List of Authors:	Fernandes, Michelle; University of Oxford, Nuffield Department of Women's & Reproductive Health; University of Southampton, Department of Paediatrics<br>Villar, José; University of Oxford, Nuffield Department of Women's & Reproductive Health; University of Oxford, Perinatal Health Institute, Green Templeton College<br>Stein, Alan; University of Oxford, Department of Psychiatry<br>Staines Urias, Eleonora; University of Oxford, Nuffield Department of Women's & Reproductive Health<br>Garza, Cutberto; Johns Hopkins University, Johns Hopkins Bloomberg School of Public Health<br>Victora, Cesar; Federal University of Pelotas, Postgraduate Program in Epidemiology<br>Barros, Fernando; Catholic University of Pelotas, Post-Graduate Program on Health and Behavior; Universidade Catolica de Pelotas, Post-Graduate Program in Health and Behavior<br>Bertino, Enrico; Universita degli Studi di Torino<br>Purwar, Manorama; Ketkar Hospital, Nagpur INTERGROWTH-21st Research Centre<br>Carvalho, Maria; Aga Khan University - Kenya<br>Giuliani, Francesca ; Ospedale Infantile Regina Margherita, Ospedale Infantile Regina Margherita-Sant'Anna Citta della Salute e della Scienza di Torino<br>Wulff, Katharina; University of Oxford, Nuffield Department of Clinical Neuroscience<br>Abubakar, Amina A.; KEMRI-Wellcome Trust Research Programme, Neurosciences Unit<br>Kihara, Michael; USIU, Department of Psychology<br>Cheikh Ismail, Leila; University of Sharjah, College of Health Sciences<br>Aranzeta, Luis; Centro de Tecnologıa e Innovacion<br>Albernaz, Elaine; Universidade Federal de Pelotas, Faculty of Medicine<br>Kunnawar, Naina; Ketkar Hospital Nagpur, Nagpur INTERGROWTH-21st Research Centre<br>Di Nicola, Paola; Ospedale Infantile Regina Margherita, Ospedale Infantile Regina Margherita-Sant'Anna Citta della Salute e della Scienza di Torino<br>Ochieng, Roseline; Aga Khan University - Kenya, Faculty of Health Sciences<br>Sandells, Tamsin; University of Oxford, Nuffield Department of Women's

	& Reproductive Health<br>Savini, Sandy; University of Oxford, Nuffield Department of Women’s & Reproductive Health<br>Temple, Sophie; University of Oxford, Nuffield Department of Women’s & Reproductive Health<br>Murray, Elizabeth; University of Oxford, Department of Psychiatry, Warneford Hospital<br>Ohuma, Eric O; University of Oxford Medical Sciences Division<br>Gravett, MG; University of Washington, Departments of Obstetrics and Gynecology and of Global Health<br>Pang, Ruyan; Peking University, School of Public Health<br>Jaffer, Yasmine; Ministry of Health, Muscat, Department of Family & Community Health<br>Noble, J. Alison; University of Oxford, Department of Engineering Science<br>Winsey, Adele; University of Oxford, Nuffield Department of Women’s & Reproductive Health<br>Lambert, Ann; University of Oxford, Nuffield Department of Women’s & Reproductive Health<br>Papageorghiou, Aris; University of Oxford, Nuffield Department of Women’s & Reproductive Health; University of Oxford, Oxford Maternal & Perinatal Health Institute, Green Templeton College,<br>Bhutta, Zulfiqar; The Hospital for Sick Children , Centre for Global Child Health<br>Kennedy, Stephen; University of Oxford, Nuffield Department of Women’s & Reproductive Health; University of Oxford, Oxford Maternal & Perinatal Health Institute, Green Templeton College,

Keywords:	Community child health < PAEDIATRICS, Developmental neurology & neurodisability < PAEDIATRICS, PAEDIATRICS, EPIDEMIOLOGY

SCHOLARONE™
Manuscripts

I, the Submitting Author has the right to grant and does grant on behalf of all authors of the Work (as defined in the below author licence), an exclusive licence and/or a non-exclusive licence for contributions from authors who are: i) UK Crown employees; ii) where BMJ has agreed a CC-BY licence shall apply, and/or iii) in accordance with the terms applicable for US Federal Government officers or employees acting as part of their official duties; on a worldwide, perpetual, irrevocable, royalty-free basis to BMJ Publishing Group Ltd ("BMJ") its licensees and where the relevant Journal is co-owned by BMJ to the co-owners of the Journal, to publish the Work in this journal and any other BMJ products and to exploit all rights, as set out in our licence.

The Submitting Author accepts and understands that any supply made under these terms is made by BMJ to the Submitting Author unless you are acting as an employee on behalf of your employer or a postgraduate student of an affiliated institution which is paying any applicable article publishing charge ("APC") for Open Access articles. Where the Submitting Author wishes to make the Work available on an Open Access basis (and intends to pay the relevant APC), the terms of reuse of such Open Access shall be governed by a Creative Commons licence – details of these licences and which Creative Commons licence will apply to this Work are set out in our licence referred to above.

Other than as permitted in any relevant BMJ Author's Self Archiving Policies, I confirm this Work has not been accepted for publication elsewhere, is not being considered for publication elsewhere and does not duplicate material already published. I confirm all authors consent to publication of this Work and authorise the granting of this licence.

For peer review only

The INTERGROWTH-21st Project International INTER-NDA standards for child development at two years of age: An International Prospective Population-based Study

Short title: INTERGROWTH-21st Project INTER-NDA standards

Authors' names	Michelle Fernandes (0000-0002-0051-3389), José Villar, Alan Stein, Eleonora Staines Urias, Cutberto Garza, Cesar G. Victora, Fernando C. Barros, Enrico Bertino, Manorama Purwar, Maria Carvalho, Francesca Giuliani, Katharina Wulff, Amina Abubakar, Michael Kihara, Leila Cheikh Ismail, Luis Aranzeta, Elaine Albernaz, Naina Kunnawar, Paola Di Nicola, Roseline Ochieng, Tamsin Sandells, Sandy Savini, Sophie Temple, Elizabeth Murray, Eric O. Ohuma, Michael G. Gravett, Ruyan Pang, Yasmine A. Jaffer, J. Alison Noble, Adele Winsey, Ann Lambert, Aris T. Papageorghiou, Zulfiqar A. Bhutta, and Stephen H. Kennedy.
Authors' names, positions, affiliations and addresses	Michelle Fernandes, Honorary Research Fellow and NIHR Academic Clinical Fellow in Paediatrics, Nuffield Department of Women's & Reproductive Health, Women's Centre, John Radcliffe Hospital, University of Oxford, Oxford OX3 9DU, UK and Faculty of Medicine, Department of Paediatrics, University Hospitals Southampton, University of Southampton, Southampton SO16 6YD, UK.<br><br>José Villar, Professor, Nuffield Department of Women's & Reproductive Health, University of Oxford, Oxford, OX3 9DU, UK and Oxford Maternal & Perinatal Health Institute, Green Templeton College, University of Oxford, Oxford, OX2 6HG, UK.<br><br>Alan Stein, Professor and Head of Section of Child and Adolescent Psychiatry, Department of Psychiatry, Warneford Hospital, University of Oxford, Oxford, OX3 7JX, UK.<br><br>Eleonora Staines Urias, Senior Statistician, Nuffield Department of Women's & Reproductive Health, University of Oxford, Oxford, OX3 9DU, UK.<br><br>Cutberto Garza, Professor, Johns Hopkins Bloomberg School of Public Health, Baltimore, MD, 21205, USA.<br><br>Cesar G. Victora, Professor, Programa de Pós-Graduação em Epidemiologia, Universidade Federal de Pelotas, Pelotas, 96010-610, Brazil.<br><br>Fernando C. Barros, Professor, Programa de Pós-Graduação em Saúde e Comportamento, Universidade Católica de Pelotas, Pelotas, 96015-560, Brazil.<br><br>Enrico Bertino, Professor, Dipartimento di Scienze Pediatriche e dell' Adolescenza, SCDU Neonatologia, Universita di Torino, Torino, 10126, Italy.

For peer review only - http://bmjopen.bmj.com/site/about/guidelines.xhtml

Manorama Purwar, doctor, Nagpur INTERGROWTH-21st Research Centre, Ketkar Hospital, Nagpur, 440012, Maharashtra, India.

Maria Carvalho, doctor, Faculty of Health Sciences, Aga Khan University, Nairobi, Kenya.

Francesca Giuliani, doctor, Ospedale Infantile Regina Margherita-Sant'Anna Citta della Salute e della Scienza di Torino, Torino, 10126, Italy.

Katharina Wulff, Senior Research Fellow, Nuffield Department of Clinical Neuroscience, University of Oxford, Oxford OX3 9DU.

Amina Abubakar, Neurosciences Unit, KEMRI/Wellcome Trust Research Programme, PO Box 230-80108 Kilifi, Kenya.

Michael Kihara, Professor, Department of Psychology, United States International University, Africa.

Leila Cheikh Ismail, College of Health Sciences, University of Sharjah, Sharjah, United Arab Emirates.

Luis Aranzeta, Software developer, Centro de Tecnologıa e Innovacion, Ciudad de Mexico 11529, Mexico.

Elaine Albernaz, doctor, Faculty of Medicine, Universidade Federal de Pelotas, Pelotas, 96015-560, Brazil.

Naina Kunnawar, doctor, Nagpur INTERGROWTH-21st Research Centre, Ketkar Hospital, Nagpur, 440012, Maharashtra, India.

Paola Di Nicola, doctor, Ospedale Infantile Regina Margherita-Sant'Anna Citta della Salute e della Scienza di Torino, Torino, 10126, Italy.

Roseline Ochieng, doctor, Faculty of Health Sciences, Aga Khan University, Nairobi, Kenya.

Tamsin Sandells, research assistant, Nuffield Department of Women's & Reproductive Health, University of Oxford, Oxford, OX3 9DU, UK.

Sophie Temple, research assistant, Nuffield Department of Women's & Reproductive Health, University of Oxford, Oxford, OX3 9DU, UK.

Sandy Savini, research assistant, Nuffield Department of Women's & Reproductive Health, University of Oxford, Oxford, OX3 9DU, UK.

Elizabeth Murray, PhD student, Department of Psychiatry, Warneford Hospital, University of Oxford, Oxford, OX3 7JX, UK.

For peer review only

	Eric O. Ohuma, Senior Statistician, Nuffield Department of Women's & Reproductive Health, University of Oxford, Oxford, OX3 9DU, UK and Centre for Statistics in Medicine, Nuffield Department of Orthopaedics, Rheumatology & Musculoskeletal Sciences, University of Oxford, Oxford, OX3 7LD, UK.<br><br>Michael G. Gravett, Adjunct Professor, Departments of Obstetrics and Gynecology and of Global Health, University of Washington, Seattle, WA 98195-6460, USA.<br><br>Ruyan Pang, Professor, School of Public Health, Peking University, Beijing, 100191, China.<br><br>Yasmine A. Jaffer, Department of Family & Community Health, Ministry of Health, Muscat, Sultanate of Oman.<br><br>J. Alison Noble, Professor, Department of Engineering Science, University of Oxford, Oxford, OX1 3PJ, UK.<br><br>Adele Winsey, Nuffield Department of Women's & Reproductive Health, University of Oxford, Oxford, OX3 9DU, UK.<br><br>Ann Lambert, Nuffield Department of Women's & Reproductive Health, University of Oxford, Oxford, OX3 9DU, UK.<br><br>Aris T. Papageorghiou, Professor, Nuffield Department of Women's & Reproductive Health, University of Oxford, Oxford, OX3 9DU, UK and Oxford Maternal & Perinatal Health Institute, Green Templeton College, University of Oxford, Oxford, OX2 6HG, UK.<br><br>Zulfiqar A. Bhutta, Professor, Center for Global Child Health, Hospital for Sick Children, Toronto, M5G 2L3, Canada.<br><br>Stephen H. Kennedy, Professor and Head of Department, Nuffield Department of Women's & Reproductive Health, University of Oxford, Oxford, OX3 9DU, UK and Oxford Maternal & Perinatal Health Institute, Green Templeton College, University of Oxford, Oxford, OX2 6HG, UK.
**Corresponding author**	Correspondence to Michelle Fernandes, Nuffield Department of Women's & Reproductive Health, Women's Centre, John Radcliffe Hospital, University of Oxford, Oxford OX3 9DU, UK.<br><br>E: michelle.fernandes@wrh.ox.ac.uk; T:+44(0)1865222122
**Keywords**	Community child health, child development, paediatrics, epidemiology, neurodevelopment.

For peer review only

WORD COUNTS

Abstract: 299

Article Summary: 173

Main manuscript: 5016

For peer review only - http://bmjopen.bmj.com/site/about/guidelines.xhtml

ABSTRACT

Objectives

To describe the construction of the international INTER-NDA standards for child development at two years by reporting the cognitive, language, motor and behaviour outcomes in optimally healthy and nourished children in the INTERGROWTH-21st Project.

Design

Population-based cohort study, the INTERGROWTH-21st Project.

Setting

Brazil, India, Italy, Kenya and the UK.

Participants

1181 children prospectively recruited from early fetal life according to the prescriptive WHO approach, and confirmed to be at low risk of adverse perinatal and postnatal outcomes.

Primary Measures

Scaled INTER-NDA domain scores for cognition, language, fine and gross motor skills and behaviour; vision outcomes measured on the Cardiff tests; attentional problems and emotional reactivity measured on the respective subscales of the preschool Child Behaviour Checklist (CBCL); and the age of acquisition of the WHO gross motor milestones.

Results

Scaled INTER-NDA domain scores are presented as centiles, which were constructed according to the prescriptive WHO approach and excluded children born preterm and those with significant postnatal/neurological morbidity. For all domains, except negative behavior, higher scores reflect better outcomes and the threshold for normality was defined as ≥ 10th centile. For the INTER-NDA's cognitive, fine motor, gross motor, language and positive behaviour domains these are ≥38.5, ≥25.7, ≥51.7, ≥17.8, and ≥51.4, respectively. The threshold for normality for the INTER-NDA's negative behaviour domain is ≤50.0, i.e. ≤90th centile. At 22 to 30 months of age, the cohort overlapped with the WHO motor milestone centiles, showed low postnatal morbidity (<10%), and vision outcomes, attentional problems and emotional reactivity scores within the respective normative ranges.

Conclusions

From this large, healthy and well-nourished, international cohort, we have constructed, using the WHO prescriptive methodology, international INTER-NDA standards for child development at 2 years of age. Standards, rather than references, are recommended for population-level screening and the identification of children at risk of adverse outcomes.

For peer review only - http://bmjopen.bmj.com/site/about/guidelines.xhtml

ARTICLE SUMMARY

- A population-based strategy was implemented to obtain an international, healthy and well-nourished sample of children aged 2 years from Brazil, India, Italy, Kenya and the UK using the prescriptive WHO approach for the construction of biological standards.
- Comprehensive health, growth and neurodevelopmental data were prospectively collected spanning pregnancy, birth, early postpartum and early childhood providing a unique opportunity to confirm the health and nutritional status of the cohort and to control for multiple risk factors for adverse child development.
- The primary outcome, child neurodevelopment at age 2 years, was based on a mixed-methodology, multi-dimensional standardised measure, the INTERGROWTH-21st Project neurodevelopmental assessment (INTER-NDA). The resultant normative centiles represent the fist international INTER-NDA standards for child development at age 2 years, constructed using the WHO MGRS approach.
- The INTER-NDA is a standardized screening assessment and does not provide a clinical diagnosis. Therefore the possibility for misclassification must be considered when interpreting the findings of this analysis.
- The age range of the INTER-NDA is 22 to 30 months and limits its generalizability to other age groups.

For peer review only

For peer review only - http://bmjopen.bmj.com/site/about/guidelines.xhtml

INTRODUCTION

Approximately 250 million children under the age of five worldwide are at risk of not achieving their developmental potential.[1] Effective interventions are available but maximising their benefit at scale depends upon identifying those children at greatest need, preferably using standardised methodology.[2]

At present, a multiplicity of methods are used to measure neurodevelopment during early childhood (Supporting Information S1).[3 4] Many of these are administered by specialist staff and were developed using children from either high-income (HICs) or specific low- and middle-income countries (LMICs), each drawing their normative sample (often country- or region-specific) from the respective settings (Supporting information S1 and S2).[3 4] To our knowledge, none of these tools commonly used to measure neurodevelopment in early childhood, were based on children monitored from fetal life, and have adopted the prescriptive approach recommended by the World Health Organization (WHO) for the development of international biological standards during the construction of their norms.[5] Instead, references have been commonly used to assess the overall achievement of developmental skills and track progress over time in both, groups of children, and individuals. However, while references describe *how* children, in a specific setting and time, have attained certain milestones of interest, they do not describe how children, in all settings, *should* develop. The importance of this fundamental difference between references and standards was elegantly highlighted, in the context of skeletal growth in young children, by the WHO Multicentre Growth Reference Study (MGRS), which resulted in the construction of prescriptive international standards for monitoring child growth.[6] These WHO standards, describing optimal growth from early pregnancy to five years of age,[7 8] are now widely employed in clinical practice and used to make comparisons across disparate populations.

The construction of international, prescriptive standards describing optimal neurodevelopment during early childhood is challenging not least because of the technical and logistical difficulties of implementing comprehensive early child developmental assessments across large international populations. To construct international *standards* of child development, in accordance with the WHO's prescriptive methodology,[5] four fundamental methodological principles must be fulfilled: (1) The normative sample should be selected using a "prescriptive" approach, which includes consideration of key factors known to be associated with poor developmental outcomes during early childhood (Supporting Information S2); (2) the conceptual framework *must* be population-based and international; (3) rigorous data management, standardisation and quality control procedures must be included, and (4) measurements must be complemented by independent assessments of specific functional and developmental domains (e.g. tests of vision) to confirm the prescriptive characteristics of the sample. This rigorous approach is important because the inclusion of inadequately nourished children, or those with mild neurodevelopmental disturbances (NDDs), in normative samples, can affect resultant thresholds. Moreover, the identification of children at risk of (even mild) NDDs is essential because there is evidence to show that very small developmental differences between individuals during early childhood can result in marked discrepancies in mental and physical health, educational attainment, and social and economic outcomes during later life.[9 10]

The INTERGROWTH-21st Project aimed to adopt this WHO prescriptive approach in constructing international standards for child development measured on a standardized, comprehensive assessment tool – the INTERGROWTH-21st Neurodevelopment Assessment (INTER-NDA) - at 2 years of age. Despite this circumscribed age range, by leveraging on the INTERGROWTH-21st Project's international cohort of mothers and children, recruited specifically to be optimally healthy and well-nourished throughout the duration of pregnancy and confirmed, during the infant follow-up component of the project, to be at low risk of adverse birth, health and growth outcomes at birth, 1 and 2 years of age, we were able to adopt the prescriptive approach and methods

For peer review only - http://bmjopen.bmj.com/site/about/guidelines.xhtml

recommended by the WHO MGRS in the construction of the INTER-NDA *standards* of child development at 2 years of age. In the present study, we analyzed cognitive, language, motor and behaviour outcomes at 2 years of age, measured on the INTER-NDA, for healthy and well-nourished children from the INTERGROWTH-21st Project study sites in Brazil, India, Italy, Kenya and the UK. We compared the vision, gross motor, attentional problems and emotional reactivity profiles, as well as growth and health outcomes, in these children to the corresponding norms for these independent measures.

For peer review only

METHODS

Study design and population

The INTERGROWTH-21st Project was a multi-centre, population-based study conducted between 2009 and 2016, in eight delimited geographical areas worldwide: the cities of Pelotas, Brazil; Turin, Italy; Muscat, Oman; Oxford, UK; Seattle, USA; Shunyi County, a suburban district of the Beijing municipality, China; the central area of the city of Nagpur, Maharashtra, India; and the Parklands suburb of Nairobi, Kenya. A geographical area was a complete city, or county, or part of a city with clear political or geographical limits, located at an altitude <1600m, with low-risk health indicators for perinatal morbidity and mortality, in which women receiving antenatal care had plans to give birth within the area, that had to be free or have low levels of major, known, non-microbiological contamination.[11] The primary aim of the INTERGROWTH-21st Project was to study growth, health, and development from early fetal life to two years of age in low-risk populations of mothers and children with optimal health and nutrition so as to produce prescriptive standards of fetal growth, newborn size and early child neurodevelopment to complement the existing WHO Child Growth Standards.

The INTERGROWTH-21st Project recruited pregnant women from the aforementioned populations, who met the individual entry criteria of health, nutrition, education, and socio-economic position (Supporting Information S3).[11] Standardised clinical care and neonatal feeding practices were implemented based on project protocols. The newborn cohort was followed up at birth, one and two years of age and evaluated for growth, nutrition, health, and the WHO gross motor milestones, using standardised methodology and rigorous quality control processes.[12] They constitute the Infant Follow-up Study (IFS) of the INTERGROWTH-21st Project. The baseline characteristics of the full cohort and follow-up methodology have been published elsewhere.[12] The project protocols are available at www.intergrowth21.org.uk.

Data collection and evaluation methods

All eligible children in five of the eight INTERGROWTH-21st Project study sites (the cities of Pelotas (Brazil); Turin (Italy); Oxford (UK); Nagpur (India) and the Parklands suburb of Nairobi (Kenya)), who had contributed data towards the construction of the international Fetal Growth and Newborn Growth Standards,[13 14] were invited to attend a comprehensive neurodevelopmental evaluation at the time of their second birthday. This age was selected as it was found to be the earliest at which: (i) neurodevelopment is not confounded by transient neurological syndromes of prematurity and (ii) conventionally used developmental instruments, such as the Bayley Scales of Infant Development (BSID), have been found to possess an acceptable level of medium and long term predictive validity.[15] The sites in China, Oman, and the USA did not participate because of logistical and administrative reasons, delays in the start of the study and/or staff availability, all unrelated to the IFS' main hypotheses (a comparison in the demographics, and health and growth outcomes between these sites has already been published).[12]

The evaluation consisted of (in order of administration): an assessment of vision (the Cardiff tests) an assessment of cognition, motor skills, language skills and behaviour (the INTER-NDA); caregiver reports of attentional problems and emotional reactivity (the corresponding subscales of the preschool Child Behaviour Checklist; CBCL); measurement of cortical auditory processing (to a novelty odd-ball paradigm on a wireless, gel-free electroencephalography system); measurement of infant sleep (using actigraphy) and an assessment of gross motor milestones (based on the WHO's checklist). Despite measuring cortical auditory processing and sleep in our cohort, a description of the methods and results relating to these technically complex outcomes are

For peer review only - http://bmjopen.bmj.com/site/about/guidelines.xhtml

beyond the scope of this paper. Moreover, as normative values for cortical auditory evoked response potentials and actigraphy data do not exist for children aged 2 years, the added value of these measures in confirming the healthy and well-nourished status of the cohort is uncertain. Information on the child's health and nutritional status, and anthropometric measurements (weight, length and head circumference), were also collected, at the 2-year visit, according to the INTERGROWTH-21st Project protocols.

A specially designed training program for the neurodevelopmental evaluation was implemented at all sites between 2012 and 2013.[16] Staff administering the assessments were aware of the project's general principles but not the specific hypotheses being tested. They were also unaware of individual children's scores from their own and other study sites.

Primary outcome measure: The INTERGROWTH-21st Neurodevelopment Assessment (INTER-NDA)

The INTER-NDA is a comprehensive, rapid assessment of cognition, (fine and gross) motor skills, language, and (positive and negative) behaviour for children aged 22 to 30 months (Supporting Information S4).[16] Its 37 items are administered in approximately 15 minutes using a combination of psychometric techniques (direct administration, concurrent observation and caregiver reports) to minimise risks of reporter and recall bias commonly encountered in caregiver interviews[3] while acknowledging that children might perform differently in artificial testing environments than in familiar settings. Children's performance on the INTER-NDA is scored across a spectrum of abilities, rather than on a pre-defined checklist and, therefore, affords a wider description of a child's faculties.[16] It has demonstrated strong agreement with the BSID, 3rd edition (BSID-III) (interclass correlation coefficients 0·75 to 0·88, p<0·001 for all domains with little to no bias on Bland Altman analysis); satisfactory internal consistency (Cronbach's alpha 0.56 to 0.81) and good unidimensionality across subscales (Comparative Fit Index = 0.90; Tuckler Lewis Index = 0 .94)[17]; and good levels of inter-rater (k=0·70; 95% CI: 0·47–0·88) and test re-test reliability (k=0·79; 95%CI: 0·48–0·96).[16]

The INTER-NDA is designed for use across socio-economic groups and populations. Its operation manual, standardisation protocol and forms are freely available at www.intergrowth21.org.uk. The kit consists of common household items encountered across the world. In all study sites, the INTER-NDA was translated into the local languages of the sites (Brazil: Brazilian Portuguese, India: Marathi; Italy: Italian; Kenya: Kiswahili), using the WHO Mental Health Initiative translation guidelines[18], which included processes of cultural customization, translation and back translation.

Other Outcome Measures of Neurodevelopment

To confirm the developmental normality of our cohort, we assessed specific functional and developmental outcomes of relevance by including three measurements independent of the INTER-NDA: (i) visual acuity and contrast sensitivity, measured on the Cardiff Tests;[19] (ii) attention problems and emotional reactivity measured on the respective subscales of the preschool CBCL,[20] and (iii) the age of achievement of six gross motor milestones measured on the WHO's checklist.[21]

The Cardiff Tests are validated and reliable measures of binocular vision in children that are not influenced by co-existing disturbances in language or cognition, and are independent of cultural biases. Their norms have been applied for clinical purposes.[19] The operational manual for their use in the INTERGROWTH-21st Project is available at https://www.intergrowth21.org.uk. Their administration takes 5 minutes. Visual acuity and contrast

For peer review only - http://bmjopen.bmj.com/site/about/guidelines.xhtml

sensitivity are measured in quick succession and taken together are a more robust measure of the integrity and functioning of the entire visual pathway than either test alone.[22]

The preschool version of the CBCL is a parent-rated questionnaire used worldwide as a diagnostic screen for behavioural and emotional problems in young children (https://aseba.org/translations/).[20] In the IFS of the INTERGROWTH-21st Project, mothers completed questions relating to the attentional problems and emotional reactivity CBCL scales.

The WHO Gross Motor milestones checklist consists of the normative windows of achievement for six gross motor milestones, developed from the WHO MGRS cohort between 4 and 24 months of age.[21] In the INTERGROWTH-21st Project, parents were asked to report the age when they first observed or "never observed" the milestones. The same information was collected from parents at the 1 and 2 year follow-up visits to evaluate the consistency of the reported dates.[12]

Data Management and Statistics

The INTERGROWTH-21st Project neurodevelopmental evaluation was supported by an electronic, tablet-based data collection and management system (the NeuroApp).[16] This contained the INTER-NDA and vision scoring forms, operation manuals, visual cues and integrated data quality checks to facilitate rapid collection of high-quality data and to ensure their secure upload to the project's centralised and site-based data-servers on which rigorous monthly checks were performed.[23]

For the INTER-NDA, two standardisation evaluations were carried out, in accordance with guidelines published in the World Bank's Toolkit for Examining Early Child Development,[3] to assess the ability of assessors to score and administer the INTER-NDA. During the first evaluation, assessors scored children's skills on the INTER-NDA from video recordings of four assessments performed by an expert assessor. Inter-rater and test-re test reliability were compared between assessors. At the second evaluation, an expert observed assessors performing three assessments each, and rated each assessor for their ability to administer the INTER-NDA correctly on a standardised protocol adherence checklist (Supporting Information S5). Protocol adherence scores were compared between assessors. The results of these evaluations are presented in Supporting Information S6.

The sample size considerations for this report have been previously published and depended on pragmatic considerations.[24] In summary, as the present report is the 2-year follow-up of the initial FGLS cohort of pregnant women, the total number of eligible children assessed at 2 years of age was therefore fixed. The initial sample size estimations (approximately 500 fetuses per site) focused on the precision and accuracy of the extreme centiles of the complete population, i.e. the 3rd or 97th centile because they correspond closely to ±2SD, and they are the recommended cut-offs of the WHO Child Growth Standards, which are used internationally to evaluate children of this age; however, in the present study, such estimations do not apply because of the different nature of the hypothesis.[24] In this component of the study, neurodevelopment was evaluated in an average of 261 children per site (1307 children total) at 2 years of age. This sample size was considered adequate to explore the predicted small site-specific differences. Post-hoc power calculations showed that the study was sufficiently powered to observe small differences among study sites (calculations for INTER-NDA domains with power >0.99) and small effect sizes for the between-group variances.[24] For example, for a between-group variance of 10% of the total variance and a two-tail alpha of 0.05, the power is 0.84.

Summary statistics were calculated for birth, neonatal and postnatal characteristics of children completing the neurodevelopmental evaluation and compared to those lost to follow-up. These characteristics include most

For peer review only - http://bmjopen.bmj.com/site/about/guidelines.xhtml

factors associated with poor child neurodevelopmental outcomes during the first two years of life (Supporting Information S2). The analytical and statistical strategy for the construction of the INTER-NDA centiles is presented in Figure 1. For all analyses, Stata 15 software was used (StataCorp. 2017, StataCorp LLC, College Station, TX).

Data from the participating sites were pooled, following the strategy recommended by WHO.[8] We have previously reported striking similarities in the distribution of the INTER-NDA domains among children from the five sites.[24] In summary, similar to the patterns observed in linear growth from fetal life to childhood, the variability in INTER-NDA scores between sites is far less (for most domains <10%) than the total variability between individuals within a study site, justifying pooling the data to construct international standards.[24 25]

Raw mean INTER-NDA domain scores (Supporting Information S7[17]) were calculated and their distributions explored. These showed important skewness and (particularly) kurtosis. As 30 INTER-NDA items were scored on a five-point scale, and six items were scored on a three-point scale, raw domain scores were converted to standardised scaled scores (Supporting Information S8).

To explore the low-risk profile of the cohort, centiles for visual acuity (measured in logMar) and contrast sensitivity (measured in contrast %) were determined and compared to the Cardiff Tests' established norms.[19] Attention problem and emotional reactivity subscale scores were calculated using ASEBA-web software, and compared to the CBCL's norms for Group 1 societies.[20] The proportion of children within the WHO motor development windows of achievement was estimated as previously described.[12]

In addition, after other exclusions (Figure 2), 28 of 1209 eligible children scored above the CBCL's 97th percentile threshold for clinical problems on the attentional problems and emotional reactivity CBCL subscales. We compared INTER-NDA centiles including and excluding this group (Supporting Information S9). As the INTER-NDA centiles were marginally lower on some domains when this group was included, we decided to exclude these children from the normative sample INTER-NDA sample in the construction of the INTER-NDA domain standards.

As no transformation was identified that suited all INTER-NDA domains, the Harrell-Davis distribution-free estimator was used to estimate pooled centiles from the standardised scaled scores.[26] This estimator weights the order statistics by the difference between two incomplete beta functions. INTER-NDA scaled domain scores were compared between boys and girls using the Wilcoxon rank-sum test.

Patient and public involvement

Patients were not involved in developing plans for the design of the study. Parents showed support for the study through high and sustained follow-up rates in all study sites. The INTERGROWTH-21st Project maintains contact with parents in the cohort through newsletters, webinars and blogs on its website, https://intergrowth21.tghn.org/ and through Twitter (@intergrowth21st).

For peer review only - http://bmjopen.bmj.com/site/about/guidelines.xhtml

RESULTS

The INTERGROWTH-21st Project Infant Follow-up Study: INTER-NDA normative cohort characteristics and overall health and nutrition at 2 years of age

Population

Of the 1758 eligible children enrolled in the five participating sites, 1339 (76%) were assessed at two years of age (Figure 2). After exclusions (including 54 children (3·1%) who were born at <37^{+0} weeks' gestation and 28 children who scored at the threshold for clinical problems on the attentional problems and/or emotional reactivity subscales of the CBCL), data from 1181 healthy children (67% of those eligible) were pooled to construct the international INTERGROWTH-21st Neurodevelopmental Standards. The study sites in Brazil, India, Italy, Kenya and the UK respectively contributed 147 (12·2%), 305 (25·2%), 296 (24·5%), 301 (24·9%) and 160 (13·2%) children to the normative INTER-NDA cohort. A detailed description of the prenatal, birth, postnatal morbidity, growth and nutritional characteristics of the cohort during the first two years of life has previously been published and is presented in Tables 1 and 2.[12] The comparison in socio-demographic, birth, health and growth characteristics between the five sites that contributed to the normative INTER-NDA cohort and the three sites that did not has also been previously published - no significant differences were observed between the two groups.[12]

The mean (± SD) age of both girls and boys at assessment was 24·8 (± 1·6) months. Eighty-nine percent of the neurodevelopmental measures were obtained between 22 and 24 months of age, and 99·9% between 22 and 30 months. The baseline prenatal, perinatal and neonatal characteristics were very similar across the five sites,[24] and with those children lost to follow-up (Table 1).

Health, growth and nutritional outcomes from birth to two years

The cohort's mean gestational age and weight at birth were 39·6 (± 1·2) weeks and 3·2 (± 0·4) kg, respectively.[12] The mean birth length and head circumference were 49.2 (±1.8) cms and 34.0(+1.3) cms respectively. Mean age at discharge from hospital, post-birth, was 3 (2-4) days. At hospital discharge, 89% of the cohort was exclusively breast-milk fed.[12] Exclusive breastfeeding was stopped at a median of 5 months (interquartile range, 3-6 months) and (any) breastfeeding stopped entirely at a median of 12 months (interquartile range, 6-18 months). Detailed information on the nutritional status of the cohort has been previously published.[12]

The overall postnatal morbidity of the cohort was low (Table 2): 9·4% of infants were hospitalised during the second year of life with a median hospital stay of 2 days (IQR 1, 3 days). The most frequently morbidities reported in outpatient clinics were exanthema/skin diseases, ≥3 episodes of fever lasting ≥3 days, and otitis media/lower-tract respiratory infections.[12 24] At two years of age, 92%, 90% and 91% of the cohort's length, weight and head circumference measures respectively were within the 3rd and 97th centiles of the WHO Child Growth Standards.[12]

Developmental profile of the cohort on independent measures of vision and development at 2 years

The visual acuity and contrast sensitivity centiles for our cohort are presented in Table 3. The cohort's 50th centile values for visual acuity and contrast sensitivity were 0·20 logMar and 1.5%, respectively. Both are within the Cardiff Tests' normative values for binocular visual acuity in children aged 24 to 30 months.[19] The visual acuity and contrast sensitivity values were identical for boys and girls across all centiles (Table 3) suggesting no biological variability in these outcomes between sexes.

For peer review only - http://bmjopen.bmj.com/site/about/guidelines.xhtml

The cohort's attentional problems and emotional reactivity scores at the 50th centile corresponded to CBCL T-scores of 53 and 50 respectively, i.e. the 62nd and <50th CBCL centiles. These values are below the CBCL's 93rd centile threshold for "borderline clinical problems".[20] For these CBCL subscales, 28 (2.1%) FGLS children scored above the CBCL's cut-off for clinical problems (>97th centile). These children were excluded from the INTER-NDA normative sample.

At two years of age, the cohort overlapped almost perfectly with the WHO motor milestones at the 50th, 3rd and 97th centiles of the range for healthy term infants.[12] For length and head circumference, the mean ± SD z-score was 0.0±1.1 for both measures, and the respective medians were at the 49th and 50th percentiles of the WHO Child Growth Standards.[12] For weight, the mean ± SD z-score was 0.2±1.1, and median was at the 58th percentile. A detailed description of these characteristics are presented elsewhere.[12]

International standards for the cognitive, motor, language and behavior domains of the INTER-NDA

The 3rd, 5th, 10th, 50th, 90th, 95th and 97th centiles for the INTER-NDA standardised (scaled) scores for cognition, language, motor skills, and behaviour domains for healthy, well-nourished two year-old children are presented, in Table 4, for the pooled cohort. For all INTER-NDA domains, except negative behavior, higher scores reflect better outcomes and the threshold for normality was defined as ≥10th centile. For negative behavior, where lower scores reflect better outcomes, the threshold for normality was defined as ≤90th centile. The threshold of normality for the INTER-NDA's cognitive, fine motor, gross motor, language and positive behaviour domains is ≥38.5, ≥25.7, ≥51.7, ≥17.8, and ≥51.4 respectively. The threshold for normality for the INTER-NDA's negative behaviour domain is ≤50.0. To facilitate the easy and rapid implementation of these standards in clinical, community and research settings for the identification of children scoring ≤10th and ≤3rd centile on the INTER-NDA (≥90th and ≥97th centiles for negative behaviour) who would benefit from urgent and routine further assessment and/or specialist referral respectively, we have developed a neurodevelopmental chart that can be printed or downloaded (Figure 3).

INTER-NDA domain scores were similar between the cohort's male and female children (Supporting Information S10). There was a trend towards higher cognitive and language scores among girls, and higher negative behaviour scores among boys (Supporting Information S10); however, the clinical and developmental implications of these differences are unclear.

For peer review only

DISCUSSION

From this international, population-based cohort of optimally healthy and nourished children from Brazil, India, Italy, Kenya and the UK; monitored from early pregnancy to two years of age, we have constructed international prescriptive standards for cognitive, language, motor and behavioural outcomes in two year-old children measured on a rapid, comprehensive assessment - the INTER-NDA. These centiles were constructed after excluding children born at <37 weeks gestation; those with significant/neurological morbidity, those whose mothers were known to have a mental health diagnoses during pregnancy and those who scored above the threshold for clinical attentional and emotional reactivity problems on the CBCL. We have confirmed the prenatal, perinatal, neonatal and postnatal healthy and well-nourished status of the normative INTER-NDA cohort using multiple measures during pregnancy, birth, 1 and 2 years of age; and have confirmed, at 2 years of age, its low-risk profile or adverse neurodevelopmental outcomes.[12] The threshold of normality for the INTER-NDA's cognitive, fine motor, gross motor, language and positive behaviour domains is ≥38.5, ≥25.7, ≥51.7, ≥17.8, and ≥51.4 respectively. The threshold for normality for the INTER-NDA's negative behaviour domain is ≤50.0. These centiles represent, to our knowledge, the first endeavour to construct standards for child developmental outcomes in this age group using the WHO prescriptive methodology *and* an international sampling frame. To facilitate the easy and rapid implementation of these standards in clinical, community and research settings for the identification of children at risk who would benefit from routine and urgent further assessment and specialist referral, respectively, we have developed a neurodevelopmental chart that can be printed or downloaded (Figure 3).

Strengths and limitations of this study

The strengths of the Infant Follow-up Study of the INTERGROWTH-21st Project include the population based cohort design; the use of the WHO recommended "prescriptive" approach; the international sampling frame; the inclusion of rigorous data management, standardization and quality control procedures and the incorporation of independent measurements of specific functional and developmental domains (vision, attentional problems, emotional reactivity and age of acquisition of key gross motor milestones) to confirm the satisfactory growth, health and development of our cohort was confirmed prior to the construction of these standards.[12] In addition, we used the INTER-NDA as the developmental measure of choice to construct these standards (Table 5). In Supporting Information S1 and S2, we present an overview of the normative samples and thresholds for NDDs of ten instruments commonly used to measure neurodevelopmental outcomes in two year-old children. Of these, two tools (the Guide for Monitoring Child Development, GMCD[27], [28] and the Caregiver-reported Early Developmental Instruments, CREDI[29]) fulfill some of the WHO-based methodological criteria for the construction of child developmental standards (GMCD: criteria 1, 3 and 4; and CREDI: criteria 2 and 4). The INTER-NDA fulfills 24 of the 26 criteria. Although a multi-dimensional assessment, is easy to implement and was designed for use across population groups in high-, middle- and low-income settings.[16] Despite an administration time of 15 minutes, it has demonstrated good to acceptable agreement with the BSID-III[17], and can be administered reliably, in the field, by trained non-specialists (Supporting Information S6).

The main limitation of our study was that the INTER-NDA is restricted to the 2-year age group. We selected 22 to 30 months as the time-point for the key developmental assessment of the entire study because developmental markers at this age have been found to be predictive of intelligence, school performance, adult nutrition and human capital in high-, middle- and low-income settings;[30-32] this age also corresponds to the end of Piaget's sensorimotor stage.[33] We acknowledge that, while some authors prefer a wider age range for population-

For peer review only - http://bmjopen.bmj.com/site/about/guidelines.xhtml

based child developmental surveillance,[3] the second birthday remains the earliest time point at which a holistic snapshot of a child's developmental repertoire can be captured reliably and parsimoniously at scale,[16] while still *within* 'the golden window of opportunity for neurodevelopment rescue' - the first 3 years of life - when interventions are evidenced to yield considerable benefits.[34] Conversely, some may argue that the 22 to 30 month age range is too broad in the context of the rapidly developing nervous system. By evaluating the performance of the INTER-NDA against the BSID-III in children aged 22 to 30 months[17], we have provided evidence that the INTER-NDA is a valid and reliable measure of child development in this age group. Nevertheless, the INTER-NDA is a standardized screening assessment and does not provide a clinical diagnosis. Therefore the possibility for misclassification must be considered when interpreting the findings. A further limitation is that three of the INTERGROWTH-21st Project study sites (China, Oman and the USA) did not participate in the neurodevelopmental evaluation. While the inclusion of these sites might have increased our overall sample size; as evidenced by the WHO MGRS study, the representation of every country is not necessary for the construction of biological standards because of the inherent prescriptive nature of the cohort.[35 36] Our findings, published in 2014 and earlier this year, confirmed that the growth and development of children across different ancestries, geographies and cultures are very similar from early pregnancy to 2 years of age, when environmental constraints on their health and nutrition are minimal, and justified the pooling of data across the five populations for the construction of international standards.[24 25] An additional limitation of our study is the exclusion of the detailed auditory and actigraphy data from the analyses.

To address the question as to what limits should be applied to determine thresholds of normal and non-normal development [37], we were guided by other neurodevelopmental tools using centile ranks to stratify NDD risk (Supporting Information S1). While many of these define sub-optimal development as below the 25th centile, we have presented evidence that most children in our cohort were developmentally normal for age. Therefore, we selected a lower threshold (≥10th centile) to define neurodevelopmental normality. Nevertheless, we acknowledge that, in clinical practice, risk-threshold determination may often take into consideration other factors such as parental concerns and resource allocation.[7]

Context of the study

Measuring neurodevelopmental milestones during early childhood at scale and comparing outcomes across populations are essential prerequisites for achieving the United Nations Sustainable Development Goal (UN SDG) 4.2 ("ensure that all girls and boys have access to quality early child development, care and pre-primary education so that they are ready for primary education"). The international INTER-NDA standards presented here contribute an important component to the care of young children: a unique clinical tool for use across all health-care systems (Table 5) to measure neurodevelopmental milestones and associated behaviours in two year-olds uniformly and at scale, and to identify children at risk of NDDs who would benefit from specialist referral and further investigation (Figure 3). It is hoped that these INTER-NDA standards, complementing our published standards for fetal growth and newborn size, and the WHO Child Growth Standards, will (i) contribute to the attainment of the early child development components of the UN SDGs and the WHO survive, thrive, and transform goals of the Global Strategy on Women's, Children's and Adolescents' Health; and (ii) provide a methodological template for the extension of the construction of child developmental standards to younger and older age groups.

For peer review only - http://bmjopen.bmj.com/site/about/guidelines.xhtml

Conclusion

From this international, population-based cohort of healthy and well-nourished children, confirmed to be at low-risk of adverse health, growth and developmental outcomes during the first two years of life, we have constructed the first international standards for cognition, language, motor skills and behavior at two years of age measured on the INTER-NDA. The use of standards to measure early child development is superior to references because of their prescriptive nature and universal applicability, in a manner similar to growth standards.

For peer review only

For peer review only - http://bmjopen.bmj.com/site/about/guidelines.xhtml

Acknowledgements

We gratefully acknowledge the Health Authorities in Pelotas, Brazil; Nagpur, India; Turin, Italy; Nairobi, Kenya, and Oxford, UK who facilitated this Project by permitting the participation of the respective study sites as collaborating centres. We are grateful to MedSciNet, UK, and Centro de Tecnología e Innovación, Mexico, for their support in the development, customisation and maintenance of the Project database and the NeuroApp, respectively. We are extremely grateful to the participating mothers and children in all the study sites, and to all those who contributed to the development of the INTERGROWTH-21st Project protocol.

Contributors

JV and SHK conceptualised and designed the INTERGROWTH-21st Project. MF, JV, SK, AS, LA, AA, MK, FG and KW designed and developed the FGLS neurodevelopmental follow-up and the INTERGROWTH-21st Neurodevelopment Assessment (INTER-NDA). JV, SK, CV, FB, ZB, CG, EB, AP, MG, PR, YJ, LCI and EB were responsible for the implementation all aspects of the INTERGROWTH-21st Project. MF, JC, SHK and LCI coordinated the implementation of the neurodevelopmental follow-up. MF, LCI, FB, EA, PdN, EB, FG, MP, NS, RO, MC, TS, ST, EM, AL and AW were responsible for site-based training and data collection. JV, SK, ESU, EO and MF were responsible for data management. MF, JV, ESU, EO and SHK had access to the Project's data; JV, ESU, MF, SHK and EO were responsible for the statistical analysis. MF and JV wrote the report with input from all the other authors. All authors reviewed and approved the final manuscript.

SHK and JV are responsible for the overall content as guarantors. The guarantors accept full responsibility for the work and/or the conduct of the study, had access to the data, and controlled the decision to publish. The corresponding author attests that all listed authors meet authorship criteria and that no others meeting the criteria have been omitted.

Funding

This work was supported by a grant from the Bill & Melinda Gates Foundation to the University of Oxford, Oxford, UK (Grant ID# 49038). MF is supported by an Academic Clinical Fellowship in Paediatrics from the National Institute for Health Research, UK to the University of Southampton. AP is supported by the National Institute for Health Research Oxford Biomedical Research Centre. The funders had no role in the study design, data collection and analysis, decision to publish or preparation of the manuscript.

Competing interests

None declared.

Ethics Approval

The INTERGROWTH-21st Project was approved by the Oxfordshire Research Ethics Committee "C" (reference: 08/H0606/139), the regional health authorities and the research ethics committees of each participating institution. Parents provided written, informed consent for their children.

Data Sharing Statement

Extra data is available by emailing *intergrowth21st@tghn.org*.

Open access

For peer review only - http://bmjopen.bmj.com/site/about/guidelines.xhtml

This is an Open Access article distributed in accordance with the Creative Commons Attribution Non Commercial (CC BY-NC 4.0) license, which permits others to distribute, remix, adapt, build upon this work non- commercially, and license their derivative works on different terms, provided the original work is properly cited and the use is non-commercial. See: http:// creativecommons.org/licenses/by-nc/4.0/

For peer review only

REFERENCES

1. Richter LM, Daelmans B, Lombardi J, et al. Investing in the foundation of sustainable development: pathways to scale up for early childhood development. The Lancet 2017;389:103-18
2. Ertem IO, OrganizationWHO WH. Developmental difficulties in early childhood: prevention, early identification, assessment and intervention in low- and middle-income countries: a review. Geneva, Switzerland: World Health Organization, 2012:112.
3. Fernald LCH, Kariger P, Engle P, et al. Examining Early Child Development in Low-Income Countries: A Toolkit for the Assessment of Children in the First Five Years of Life. Washington DC: The World Bank, 2009.
4. Fischer VJ, Morris J, Martines J. Developmental screening tools: feasibility of use at primary healthcare level in low-and middle-income settings. Journal of health, population, and nutrition 2014;32:314
5. Garza C, de Onis M. Rationale for developing a new international growth reference. Food & Nutrition Bulletin 2004;25:5S-14S
6. de Onis M, Garza C, Victora CG, et al. The WHO Multicentre Growth Reference Study: planning, study design, and methodology. Food Nutr Bull 2004;25:S15-26
7. Papageorghiou AT, Ohuma EO, Altman DG, et al. International standards for fetal growth based on serial ultrasound measurements: the Fetal Growth Longitudinal Study of the INTERGROWTH-21st Project. The Lancet 2014;384:869-79.
8. WHO Multicentre Growth Reference Study G, de Onis M. WHO Child Growth Standards based on length/height, weight and age. Acta Pædiatrica 2006;95:76-85.
9. Moffitt TE, Arseneault L, Belsky D, et al. A gradient of childhood self-control predicts health, wealth, and public safety. Proceedings of the National Academy of Sciences 2011;108:2693-98.
10. Caspi A, Moffitt TE, Newman DL, et al. Behavioral observations at age 3 years predict adult psychiatric disorders: Longitudinal evidence from a birth cohort. Archives of General Psychiatry 1996;53:1033-39.
11. Villar J, Altman D, Purwar M, et al. The objectives, design and implementation of the INTERGROWTH-21st Project. BJOG: An International Journal of Obstetrics & Gynaecology 2013;120:9-26.
12. Villar J, Cheikh Ismail L, Staines Urias E, et al. The satisfactory growth and development at 2 years of age of the INTERGROWTH-21st Fetal Growth Standards cohort support its appropriateness for constructing international standards. American Journal of Obstetrics and Gynecology 2018;218:S841-S54.e2.
13. Papageorghiou AT, Ohuma EO, Altman DG, et al. International standards for fetal growth based on serial ultrasound measurements: the Fetal Growth Longitudinal Study of the INTERGROWTH-21st Project. Lancet 2014;384:869-79.
14. Villar J, Giuliani F, Bhutta ZA, et al. Postnatal growth standards for preterm infants: the Preterm Postnatal Follow-up Study of the INTERGROWTH-21st Project. Lancet Glob Health 2015;3:e681-91.
15. Marlow N, Abbott J, Field D, et al. Report of a BAPM/RCPCH Working Group: Classification of health status at 2 years as a perinatal outcome. London, UK: British Association of Perinatal Medicine, 2008:23.
16. Fernandes M, Stein A, Newton CRJ, et al. The INTERGROWTH-21st Project Neurodevelopment Package: A novel method for the multi-dimensional assessment of neurodevelopment in pre-school age children PLoS ONE 2014;9: e113360.
17. Murray E, Fernandes M, Newton CR, et al. Evaluation of the INTERGROWTH-21st Neurodevelopment Assessment (INTER-NDA) in 2 year-old children. PloS one 2018;13(2):e0193406.
18. Harkness J, Pennell B, Villar A, et al. Translation procedures and translation assessment in the World Mental Health Survey Initiative. The WHO World Mental Health Surveys: Global Perspectives on the Epidemiology of Mental Disorders 2008:91-113
19. Adoh TO, Woodhouse JM. The Cardiff acuity test used for measuring visual acuity development in toddlers. Vision Research 1994;34:555-60.
20. Achenbach TM, Rescorla LA. *Manual for the ASEBA Preschool Forms & Profiles*. Burlington, USA: University of Vermont, Research Center for Children, Youth, & Families, 2000.
21. WHO Multicentre Reference Study Group, de Onis M. WHO motor development study: Windows of achievement for six gross motor development milestones. Acta Pædiatrica 2006;S450:86-95.
22. Adams RJ, Courage ML. Contrast sensitivity in the 24- and 36-month-olds as assessed with contrast sensitivity card procedure. Optometry & Vision Science 1993;70:97-101.
23. Ohuma EO, Hoch L, Cosgrove C, et al. Managing data for the international, multicentre INTERGROWTH-21st Project. BJOG: An International Journal of Obstetrics & Gynaecology 2013;120:64-70.
24. Villar J, Fernandes M, Purwar M, et al. Neurodevelopmental milestones and associated behaviours are similar among healthy children across diverse geographical locations. Nature Communications 2019;10:511.
25. Villar J, Papageorghiou AT, Pang R, et al. The likeness of fetal growth and newborn size across non-isolated populations in the INTERGROWTH-21st Project: the Fetal Growth Longitudinal Study and Newborn Cross-Sectional Study. The Lancet Diabetes & Endocrinology 2014;2:781-92.
26. Harrell FE, Davis C. A new distribution-free quantile estimator. Biometrika 1982;69:635-40
27. Ertem IO, Dogan DG, Gok CG, et al. A guide for monitoring child development in low-and middle-income countries. Pediatrics 2008;121:e581-e89
28. Ozturk Ertem I, Krishnamurthy V, Mulaudzi MC, et al. Validation of the International Guide for Monitoring Child Development demonstrates good sensitivity and specificity in four diverse countries. Acta Paediatrica 2019;108:1074-86

For peer review only - http://bmjopen.bmj.com/site/about/guidelines.xhtml

29. McCoy DC, Waldman M, Team CF, et al. Measuring early childhood development at a global scale: Evidence from the Caregiver-Reported Early Development Instruments. Early Childhood Research Quarterly 2018;45:58-68
30. Daniels MC, Adair LS. Growth in young Filipino children predicts schooling trajectories through high school. The Journal of nutrition 2004;134:1439-46
31. Feinstein L. Inequality in the early cognitive development of British children in the 1970 cohort. Economica 2003;70:73-97
32. Victora CG, Adair L, Fall C, et al. Maternal and child undernutrition: consequences for adult health and human capital. The lancet 2008;371:340-57
33. Piaget J, Cook M. *The origins of intelligence in children*: International Universities Press New York, 1952.
34. Engle PL, Black MM, Behrman JR, et al. Strategies to avoid the loss of developmental potential in more than 200 million children in the developing world. The Lancet;369:229-42.
35. de Onis M, Grarza C, Victora C, et al. The WHO multicentre growth reference study (MGRS): Rationale, planning, and implementation. Food and Nutrition Bulletin 2004;25(1 supplement 1)
36. De Onis M, Garza C, Victora CG, et al. The WHO Multicentre Growth Reference Study: planning, study design, and methodology. Food and nutrition bulletin 2004;25:S15-S26
37. Council on Children With Disabilities SoDBP, Bright Futures Steering Committee, Medical Home Initiatives for Children With Special Needs Project Advisory Committee. Identifying Infants and Young Children With Developmental Disorders in the Medical Home: An Algorithm for Developmental Surveillance and Screening. Pediatrics 2006;118(1):405.

For peer review only

For peer review only - http://bmjopen.bmj.com/site/about/guidelines.xhtml

Table 1 Prenatal, perinatal and neonatal characteristics of children who completed the INTER-NDA in the INTERGROWTH-21st Project compared to those lost to follow-up.

**Prenatal, perinatal and neonatal characteristics**	**Children contributing to INTERGROWTH-21st international INTER-NDA standards (n=1209)**	**Children lost to follow-up (n=331)**
	Mean (SD) or number (%)	Mean (SD) or number (%)
Maternal age at recruitment, years	28.4 (3.8)	27.4 (4.3)
Maternal body-mass-index, kg/m^2	23.2 (3.0)	23.6 (2.8)
Multiple gestation	n=0 (0·0%)	n=0 (0·0%)
Chronic maternal illness	n=96 (8·1%)	n=26 (7.9)
Maternal infections (including HIV, rubella, syphilis, hepatitis B, CMV, toxoplasmosis, tuberculosis and malaria)	n=0 (0·0%)	n=1 (0.3)
Maternal haemoglobin (g/dL)	12.4 (1.0)	12.4 (1.0)
Maternal malignancy	n=0 (0·0%)	n=0 (0·0%)
Maternal substance abuse (including alcohol) and smoking	n=0 (0·0%)	n=0 (0·0%)
Maternal use of teratogenic drugs during pregnancy	n=628 (53.2)	n=222 (67·1%)
Maternal prenatal anxiety and depression/mental stress	n=0 (0·0%)	n=0 (0·0%)
Maternal preeclampsia and eclampsia	n=10 (0·9%)	n=4 (1·2%)
Placental structural anomalies	n=0 (0·0%)	n=0 (0·0%)
Fetal growth restriction	n=67 (5·7%)	n=14 (4.2)
Gestational age at delivery, weeks[a]	39.6 (1.2)	39.3 (1.5)
Birth weight, kg[a]	3.2 (0.4)	3.2 (0.5)
Birth length, cm[a]	49.2 (1.8)	49.0 (2.1)
Head circumference at birth, cm[a]	34.0 (1.2)	34.0 (1.3)
Apgar at 5 min[a]	9.5 (0.6)	9.6 (0.7)
Age at hospital discharge, days[b]	3.0 (2.0, 4.0)	2.0 (1.0, 3.0)
Boys[a]	n=564 (47.8)	n=160 (48.3)
Hyperbilirubinaemia[a]	n=49 (4.1)	n=18 (5.5)
Respiratory distress syndrome[a]	n=16 (1.4)	n=7 (2.1)
Transient tachypnoea of the newborn[a]	n=11 (0.9)	n=12 (3.6)
Exclusive breastfeeding at hospital discharge[a]	n=1097 (93.0)	n=300 (90.9)

INTER-NDA: The INTERGROWTH-21st Neurodevelopment Assessment
Data are mean (SD) or number (%) unless otherwise specified.
Missing data below 2% for all variables.
[a]Mean (SD)
[b]Median (interquartile range).
NICU: neonatal intensive care unit; ECD: early child development

For peer review only

Table 2 Postnatal morbidity between 1 and 2 years of age of children contributing to INTERGROWTH-21st international INTER-NDA standards.

**Morbidity between 1 and 2 years of life**	**Children contributing to INTERGROWTH-21st international INTER-NDA standards (n=1209)**
Hospitalised at least once	113 (9·4)
Total number of days hospitalised[a]	2 (1, 3)
Any prescription provided by a health care practitioner	712 (59·1)
Antibiotics (≥3 regimens)	142 (11·8)
Iron/folic acid/vitamin B12/other vitamins	194 (16·1)
Up-to-date with local vaccination policies	1136 (94·4)
Otitis media/Pneumonia/Bronchiolitis	88 (7·3)
Parasitosis/Diarrhoea/Vomiting	43 (3·6)
Exanthema/skin disease	150 (12·5)
Urinary tract infection/pyelonephritis	5 (0·4)
Fever ≥3 days (≥3 episodes)	134 (11·1)
Other infections requiring antibiotics	40 (3·3)
Asthma	13 (1·1)
Gastro-oesophageal reflux	3 (0·2)
Cow's milk protein allergy	8 (0·7)
Food allergies	13 (1·1)
Injury or trauma	27 (2·2)
Any condition requiring surgery	9 (0·7)

INTER-NDA: The INTERGROWTH-21st Neurodevelopment Assessment
Data are number (%) unless otherwise specified.
Missing data below 2% for all variables.
[a]Median (interquartile range).
ECD: early child development

For peer review only

Table 3 Visual acuity and contrast sensitivity centiles, measured on the Cardiff Tests, in the normative sample of the international INTER-NDA Standards.

	**Pooled Centiles (n=1209)**	**Girls (n=628)**	**Boys (n=581)**	**p-value**
**Visual Acuity (logMar)**				
**c10**	0·1	0·1	0·1	
**c25**	0·1	0·1	0·1	
**c50**	0·2	0·2	0·2	
**c75**	0·2	0·2	0·2	
**c90**	0·3	0·3	0·3	
Median (IQR)	0·2 (0·1, 0·2)	0·2 (0·1, 0·2)	0·2 (0·1, 0·2)	0·463
**Contrast Sensitivity (%)**				
**c10**	1·0	1·0	1·0	
**c25**	1·0	1·0	1·0	
**c50**	1·5	1·5	1·5	
**c75**	1·8	1·6	1·9	
**c90**	2·0	2·0	2·0	
Median (IQR)	1·5 (1·0, 2·0)	1·5 (1·0, 1·5)	1·5 (1·0, 2·0)	0·303

INTER-NDA: The INTERGROWTH-21st Neurodevelopment Assessment
*p value from Wilcoxon rank-sum test.
IQR: inter-quartile range

For peer review only

Table 4 The INTERGROWTH-21st Project International INTER-NDA standards for child development at two years of age.

INTER-NDA domain	Pooled Centiles (n=1181)						
	**c3**	**c10**	**c25**	**c50**	**c75**	**c90**	**c97**
**Cognitive**[1]	27.4	38.5	62.2	79.5	88.8	92.6	99.6
**Fine motor**[1]	17.5	25.7	74.2	91.4	100.0	100.0	100.0
**Gross motor**[1]	31.1	51.7	66.7	81.6	100.0	100.0	100.0
**Language**[1]	12.1	17.8	45.7	71.7	88.5	95.1	100.0
**Positive behaviour**[1]	37.8	51.4	70.0	90.0	100.0	100.0	100.0
**Negative behaviour**[2]	0.0	0.0	0.0	25.0	25.0	50.0	76.5

INTER-NDA: The INTERGROWTH-21st Neurodevelopment Assessment
[1]For these domains, higher scores reflect better outcomes
[2]For negative behaviour, lower scores reflect better outcomes

For peer review only

Table 5 Evaluation of the INTER-NDA against pre-established feasibility criteria for use of an early child development assessment in a low-middle income setting.

	**Does INTER-NDA fulfil the criteria?**	**Additional details**
**World Bank Toolkit for Examining ECD[1]**		
Psychometrically adequate, valid and reliable	Yes	ICCs 0·74 and 0·88 (p<0·001) between BSID-III and INTER-NDA for cognitive, language and motor domains; internal consistency 0·56-0·80[2]. Inter-rater reliability: k=0·70, 95% CI: 0·47–0·88); test-re test reliability: k=0·79, 95%CI: 0·48–0·96[3].
Balanced in terms of number of items at the lower end to avoid children with low scores	Yes	Age range of items: 6-36 months[3]
Enjoyable for children to take (e.g. interactive, colourful materials)	Yes	
Relatively easy to adapt to various cultures	Yes	Adapted via cultural customisation session during training and currently in use in 12 countries (Brazil, India, Italy, Kenya, Pakistan, Thailand, South Africa, Mexico, Grenada, Finland, Guatemala, Democratic Republic of Congo; www.inter-nda.com)
Easy to use in low-resource settings, e.g. not requiring much material	Yes	See Murray, Fernandes, Newton, et al., 2018 for image of kit[2]; cost <US$121·00; no fee per use; manuals and forms freely available at www.intergrowth21.org.uk
Not too difficult to obtain or too expensive	Yes	See above
Able to be used in a wide age range	Moderately narrow age range	22 to 30 months
**Fischer et al's feasibility criteria for use of developmental screening tools at primary healthcare level in low-middle income settings[4]**		
Results understood by health workers	Yes	Centiles
Reliable	Yes	See above
Valid	Yes	See above
Acceptable to caregivers	Yes	
Provides information that is relevant to primary care providers	Yes	Centiles
Information that can be used for referrals of early intervention	Yes	Centiles
Information that is useful for anticipatory guidance	Unknown	
Results understood by caregivers	Yes	
Staff members have the expertise to answer questions	Yes	Session on maternal questions and responses included in training package.
Access to application	Yes	Freely accessible at www.intergrowth-21.org.uk
Training involved	Yes	Time taken to train assessors in the INTER-NDA: 1 day for ≤3 assessors, 2 days for 3-5 assessors, 3 days for 5-10 assessors
How long it takes to administer the	15 minutes	

For peer review only - http://bmjopen.bmj.com/site/about/guidelines.xhtml

tool		
Cover multiple areas of child development	Yes	Cognition, language, fine and gross motor skills, and behaviour (positive, negative and global)[3]
Cost of the tool	Minimal	Cost of kit <US$121.00; no fee per use; manuals and assessment forms freely available at www.intergrowth21.org.uk. NeuroApp optional.
Minimal adaptation needed	Yes	Sessions on cultural customisation and translation included in training
Educational level of staff members	Secondary education	Results of comparison between field workers and specialists presented in Table 9 and in text
How many staff members to administer the tool	1	
Local norms available	International references available	Normative sample drawn from a prospectively recruited sample of 2 year-olds from Brazil, India, Italy, Kenya and the UK with confirmed optimal nutritional, health and developmental status during the first 1000 days of life.
Space	Minimal	Storage of kit and forms/table. See Murray, Fernandes, Newton, et al., 2018 for image of kit[2]

ICCs = interclass correlations.

[1]Fernald LCH, Kariger P, Engle P, Raikes A. Examining Early Child Development in Low-Income Countries: A Toolkit for the Assessment of Children in the First Five Years of Life. Washington DC: The World Bank, 2009.

[2]Murray E, Fernandes M, Newton CR, et al. Evaluation of the INTERGROWTH-21st Neurodevelopment Assessment (INTER-NDA) in 2 year-old children. *PloS One* 2018; 13: e0193406.

[3]Fernandes M, Stein A, Newton CRJ, et al. The INTERGROWTH-21st Project Neurodevelopment Package: A novel method for the multi-dimensional assessment of neurodevelopment in pre-school age children *PLoS One* 2014; **9**: e113360.

[4]Fischer VJ, Morris J, Martines J. Developmental screening tools: feasibility of use at primary healthcare level in low-and middle-income settings. *J Health Popul Nutr* 2014; **32**: 314.

For peer review only

Figure Titles, Legends and Captions

Figure 1
Title: Analytical and statistical strategy for the construction of the international INTERGROWTH-21st Project INTER-NDA Standards.

Legend: INTER-NDA: INTERGROWTH-21st Project Neurodevelopment Assessment

Figure 2

Title: Participant flow for the INTERGROWTH-21st Project Infant Follow-up Study cohort at two years of age.

Legend: FGLS: Fetal Growth Longitudinal Study; INTER-NDA: INTERGROWTH-21st Project Neurodevelopment Assessment

Figure 3
Title: The INTERGROWTH-21st Project International INTER-NDA standards for child development at two years of age.
Caption: INTER-NDA 3rd to 97th centile ranges for two year-old children are presented. These are based on scaled INTER-NDA standardized domain scores. Scores falling in the yellow zone correspond to scores between the 10th and 3rd centiles; scores in the orange zone correspond to scores <3rd centile. Clinical judgment should determine whether further developmental assessment is warranted for children with scores in the yellow and orange zones, and the urgency of such referrals.

For peer review only

For peer review only

For peer review only - http://bmjopen.bmj.com/site/about/guidelines.xhtml

Figure 1 Analytical and statistical strategy for the construction of the international INTERGROWTH-21st Project INTER-NDA Standards.

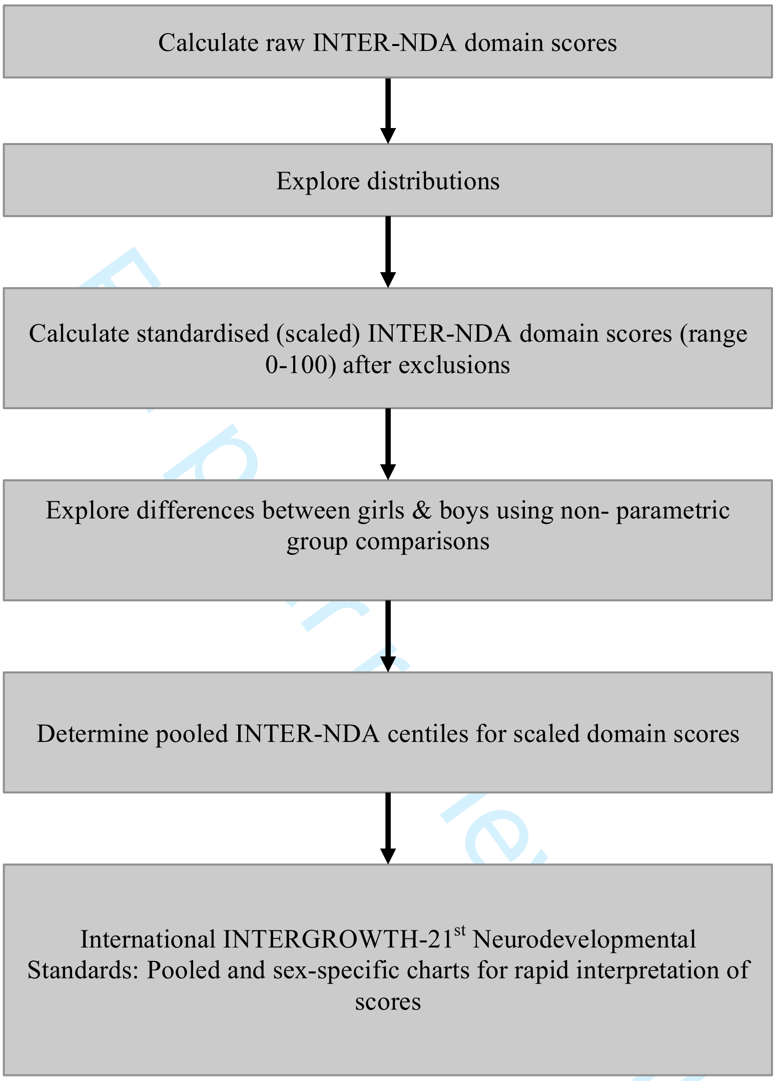

For peer review only - http://bmjopen.bmj.com/site/about/guidelines.xhtml

For peer review only

For peer review only - http://bmjopen.bmj.com/site/about/guidelines.xhtml

Figure 2 Participant flow for the INTERGROWTH-21st Project Infant Follow-up Study cohort at two years of age.

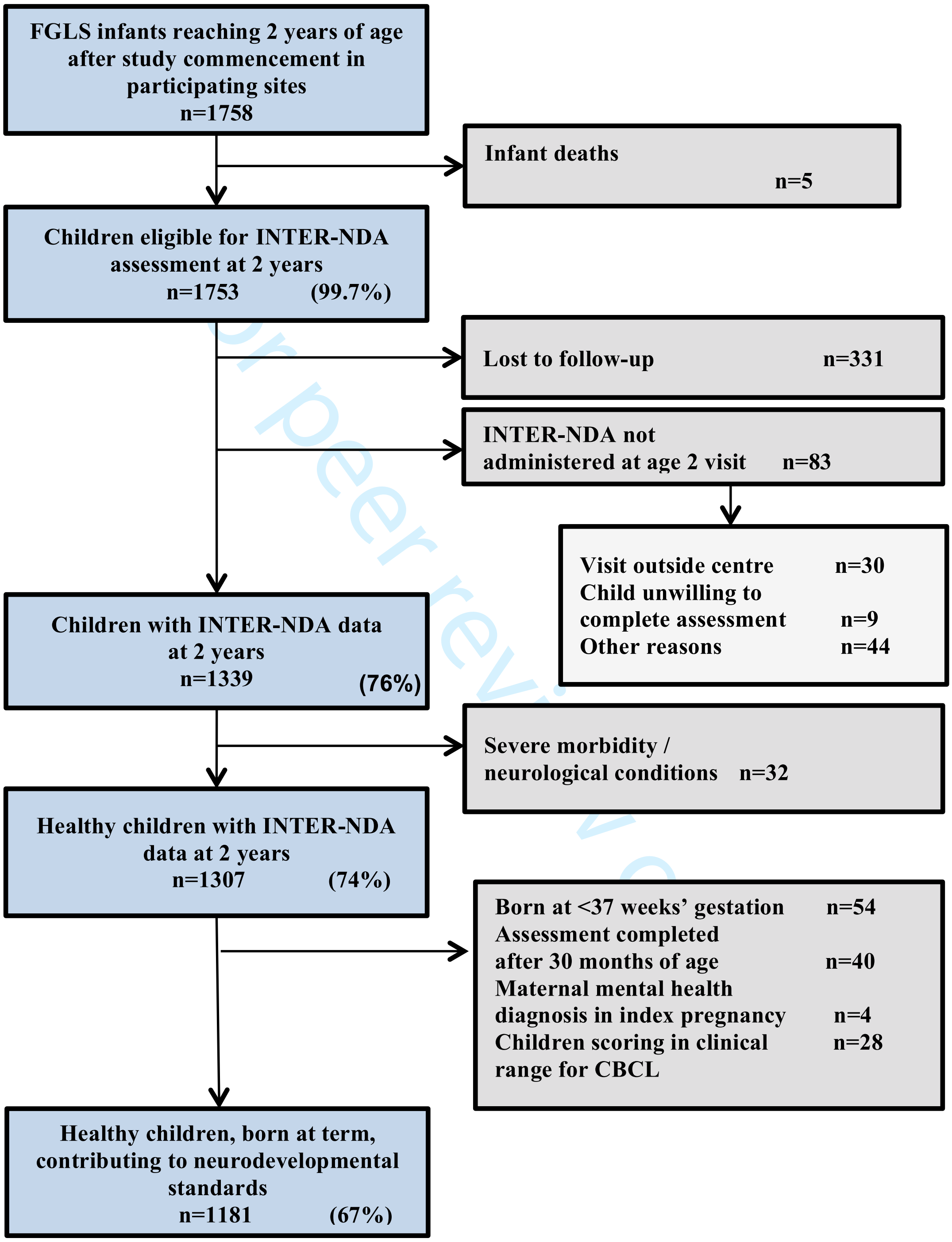

FGLS: Fetal Growth Longitudinal Study; INTER-NDA: INTERGROWTH-21st Project Neurodevelopment Assessment

For peer review only - http://bmjopen.bmj.com/site/about/guidelines.xhtml

The INTERGROWTH-21st Project International INTER-NDA Standards for Child Development at Two Years of Age

INTERGROWTH-21st

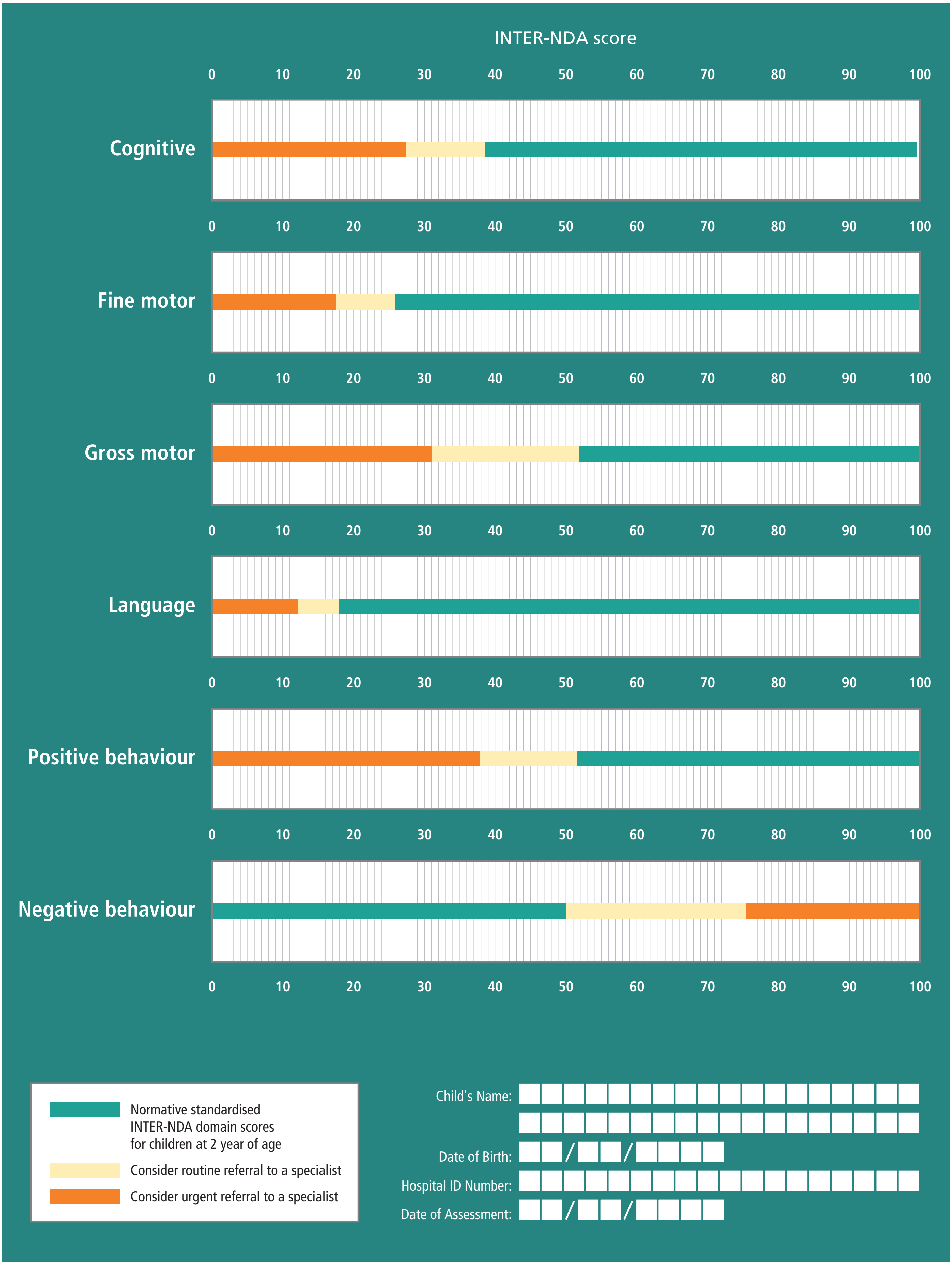

© University of Oxford

Publication reference

Supplementary Information

Supplementary Information S1 Characteristics of normative scales and thresholds used to identify neurodevelopmental impairment in commonly used developmental assessments for two year-old children.

Supplementary Information S2 Factors associated with poor neurodevelopmental outcomes in young children: Characteristics of the normative sample for the INTERGROWTH-21st Project International INTER-NDA standards, and for other neurodevelopmental tools evaluating two year-old children.

Supplementary Information S3 The INTERGROWTH-21st Fetal Growth Longitudinal Study (FGLS) individual participant entry criteria for mothers at the time of antenatal booking in the first trimester of pregnancy.

Supplementary Information S4 The INTERGROWTH-21st Neurodevelopment Assessment (INTER-NDA) data recording form.

Supplementary Information S5 The INTERGROWTH-21st Neurodevelopment Assessment (INTER-NDA) Protocol Adherence Checklist.

Supplementary Information S6 Results of the INTERGROWTH-21st Neurodevelopment Assessment (INTER-NDA) standardized evaluations between specialist and non-specialist assessors to (i) score and (ii) administer the INTER-NDA

Supplementary Information S7 The INTERGROWTH-21st Neurodevelopment Assessment (INTER-NDA) scoring system and interpretation of domain scores.

Supplementary Information S8 Formulae and tables for the conversion of raw scores to standardised (scaled) scores (range 0-100).

Supplementary Information S9 INTER-NDA domain scores centiles including and excluding children scoring above the CBCL threshold for clinical (97th centile) problems.

Supplementary Information S10 Comparison of INTERGROWTH-21st Neurodevelopment Assessment (INTER-NDA) domain scores between two year-old girls and boys

Supplementary Information S11 INTERGROWTH-21st Project Study Protocol (see separate file)

Supplementary Information S12 STROBE checklist (see separate file)

For peer review only

S1 Characteristics of normative scales and thresholds used to identify neurodevelopmental impairment in commonly used developmental assessments for two year-old children.

Tool	Subscales measured	Characteristics of normative sample	Cut-off score for neurodevelopmental delay	Details on scoring and interpretation of results
The Bayley Scales of Infant Development – III edition (BSID III) [a]*	Cognitive, expressive and receptive communication, fine and gross motor, adaptive behaviour, social emotional.	Country: USA<br>Year: January and October 2004<br>Sample Size: 24-month normative sample for cognitive, language and motor scales: 100 children (totally 1700 children aged 16 days through 43 months 15 days divided into 17 age groups of 100 children each). Normative sample for the social-emotional scale was based on 456 children and the adaptive behaviour scale was based on 1,350 children.<br>Sample characteristics: Sample selected to match the 2000 United States census.	Several criteria: **25% delay** in functioning when compared to same age peers; based on SD (**< -1 SD** i.e. cut-off thresholds of 85 for moderate impairment; **< -2 SD** i.e. cut-off thresholds of 70 for severe impairment) or **performance of a certain number of months below** the child's chronological age[1].	Scoring for every item is either 1 (credit) or 0 (no credit).<br><br>Scaled scores, composite scores, growth scores, centiles, & age-equivalents are obtained from raw scores.
The Malawi Developmental Assessment Tool (MDAT)[b]*	Gross motor, fine motor, language and social.	Country: Malawi<br>Year: June 2006 to July 2007<br>Sample size: 1426 normal healthy children aged 0 to 6 years<br>Sample characteristics: Those born preterm at <32 weeks' gestation, or with significant malnutrition using WHO criteria, medical problems or significant neurodisability were excluded.	**<25%** (upper limit of lowest quartile)	Plot children on MDAT normal reference ranges, corresponding to 25%, 50% and 75% of children passing each item.
The Griffiths Mental Development Scales[c]*	Locomotor, expressive and receptive language, personal-social, hand and eye coordination, performance, practical reasoning.	Country: UK and Ireland<br>Year: 1960s, revised in 2015<br>Sample size: 1026 children<br>Sample characteristics: National representative sample of children in UK; stratified according to geographical region and proportionate to the 1997 ONS population ratios[1].	Centiles and z -scores; **z score < -2 or SD< -2** indicates significant developmental delay on that subscale.	Raw scores are converted into z scores, developmental quotients and centile scores – these are used to obtain developmental age equivalents. A general quotient may also be obtained.
Pre-school version of Child Behavior Checklist (CBCL)[d]*	Problem and syndrome scales for emotionally reactive; anxious/depressed; somatic complaints; withdrawn; sleep problems; attention problems; aggressive behaviour.	Country: USA<br>Year: 1979, 2000<br>Sample size: Originally normed on 1728 US children.<br>Sample characteristics: -<br><br>Note: Multicultural norms available.	**>93rd centile** is abnormal (norms vary according to societies, and map onto the Diagnostic and Statistical Manual for Mental Disorders).<br><br>Any score that falls below the 93rd centile is considered normal, scores between the 93-97th centile are borderline clinical, and any score above the 97th centile is considered to be in the clinical range.	Different norms for different societies. The CBCL uses software to generate raw score; T score and centile score for each problem score as well as a total problem score, which ranges from 0 to 200. The standard scores are scaled so that 50 is average for the child's age and sex, with a standard deviation of 10 points. Higher scores indicate greater problems.
The Rapid Neurodevelopmental Assessment	Primitive reflexes, gross motor, fine motor, vision,	Country: Bangladesh<br>Year: 2010<br>Sample size: 81 children aged ≥3 to 24 months in urban (n = 47) and rural	**< -2 SD**: threshold for severe impairment;<br>**< -1 SD:** threshold for mild	For every item, the severity of functional limitations was determined as mild, moderate or severe limitation.

For peer review only - http://bmjopen.bmj.com/site/about/guidelines.xhtml

(RNDA)[e]*	hearing, speech, cognition, behaviour, and seizures.	(n = 34) community-based populations<br>Sample characteristics: 15% did not 'look' properly nourished, parental concerns regarding child development were expressed in 8% and for 50% at least one parent was illiterate.	impairment.<br>If low scores in >1 domain; the child is classified as having 'any' neurodevelopmental impairment.	
The Ages and Stages Questionnaire III edition (ASQ III)[f]	Communication, gross motor, fine motor, problem solving and personal-social.	Country: USA<br>Year: January 2004 and June 2008<br>Sample Size: 15,138 children (1,443 aged 24 months).<br>Sample characteristics: 76% of the sample had one or no known risk factor, 19% had 2 risk factors and 4% 3 or more risk factors. Risk factors were defined as extreme poverty, maternal age ≤19 years, maternal education <12th grade; involvement of child protective services with the family for abuse and/or neglect; medical risk, including prematurity; and infant's birth weight less than 3 pounds, 5 ounces.	**< -2SD**	Item scoring: 0 = not true, 1 = somewhat / sometimes true, or 2 = very true or often true of the child.
The Parents' Evaluation of Developmental Status (PEDS)[g]	General development.	Country: USA<br>Year: 1997<br>Sample size: 2823 families<br>Sample characteristics: Families from varying backgrounds, including SES and ethnicity.	Table for using scores to identify parental difficulties, non-significant concerns, one significant concern or two or more significant concerns by shading boxes based on scores. These are then used to select associated algorithms for further screening and/or referral.	Eight page booklet used to score the PEDS response form; an algorithm uses these scores to identify associated pathways for further screening and/or referral.
Caregiver-reported Early Developmental Instruments (CREDI)[h]	Long Form: Motor, cognitive, language, social-emotional and overall.<br><br>Short form: Overall development.	Countries: Brazil, Cambodia, Chile, Colombia, Ghana, Guatemala, India, Nepal, Philippines and USA<br>Year: 2017-2018<br>Sample size: 7807 children aged 0-35 months<br>Sample characteristics: Children with an "ideal home environment" defined through maternal educational attainment<br>(college or higher), and the number of activities done by adults with the child in the last 3 days (at least 4 out of the 6 MICS home stimulation activities); authors acknowledge that "although the data were representative for local populations in Brazil, Ghana, Tanzania, and Zambia, the overall sample is not representative of any country or a global population of children".	Raw scaled scores, norm referenced standardised scores, and z scores.	Uses the CREDI software package in R.
Denver Development Screening Test II (DDST II)[i]	Personal social, fine motor adaptive, language and gross motor.	Country: USA<br>Year: 1980s and 1990<br>Sample size: 2096 children<br>Sample characteristics: Children from Colorado; based on 1988, 1989 and then the 1990 US census population.	**Centile ranks** (25th, 50th, 75th, and 90th) are displayed as bar graphs and reflect the ages at which 25%, 50%, 75% and 90% of typically developing children in the standardisation sample completed the task.<br>Overall categories: Normal, and suspect.	Approximates a growth curve in its display of norms over time. The number of scores a child received below the normal expected range classifies the child as within normal, suspect, or delayed. If the child is suspect it is recommended that rescreening occur in 1-2 week.
Guide for Monitoring Child	Parental concerns, expressive language	Country: Turkey<br>Year: 1980s and 1990	<10th centile for any domain; if a child did not demonstrate ≥1 of the age-	Age at which >90% of the study sample performed each milestone was computed.

For peer review only

Development (GMCD)[j]	and communication, receptive language, relationship (social-emotional), play (social-emotional and cognitive) and self-help.	Sample size: 30 children in each age range<br>Sample characteristics: Sample selected as per WHO recommendations for a ‘prescriptive sample’ i.e. children were born healthy singletons with birth weight ≥2500 g and gestational age ≥37 weeks and had received preventive health care at 2 university-affiliated community well-child care clinics in Ankara from birth. The sample’s growth was between the 5th and 95th centiles since birth; they had received and complied with the free iron prophylaxis available to children in Turkey (or had normal haemoglobin screens within 1 month of the study); were healthy, and growing normally, at their paediatric evaluation. They had not had “any health-related problems since birth apart from acute minor illnesses.”	appropriate milestones, the GMCD interpretation was classified as “requires follow-up evaluation with or without intervention.”	

[a]©Pearson. Bayley Scales of Infant and Toddler Development, Third Edition (Bayley-III) - Training. Available from: 98 http://www.pearsonclinical.co.uk
[b]Gladstone M, Lancaster GA, Umar E, Nyirenda M, Kayira E, van den Broek NR, Smyth RL. The Malawi Developmental Assessment Tool (MDAT): the creation, validation, and reliability of a tool to assess child development in rural African settings. *PLoS medicine* 2010; **7:** e1000273.
[c]Luiz D, Barnard A, Knoesen N, Kotras N, Horrocks S, McAlinden P, Challis D, O’Connell R. Griffiths Mental Development Scales—Extended Revised: Two to Eight Years: Administration Manual. Hogrefe, Oxford, UK 2006.
[d]Achenbach TM. Manual for the Child Behavior Checklist/4-18 and 1991 profile. University of Vermont, Department of Psychiatry 1991.
[e]Khan NZ, Muslima H, Begum D, Shilpi AB, Akhter S, Bilkis K, Begum N, Parveen M, Ferdous S, Morshed R, Batra M. Validation of rapid neurodevelopmental assessment instrument for under-two-year-old children in Bangladesh. *Pediatrics* 2010; **125:** e755-62.
[f]Squires J, Bricker DD, Twombly E. Ages & stages questionnaires. Baltimore, MD: Paul H. Brookes 2009.
[g]Glascoe FP. Collaborating with parents: Using Parents' Evaluation of Developmental Status to detect and address developmental and behavioral problems. Ellsworth & Vandermeer Press 1998.
[h]McCoy DC, Waldman M, Team CF, Fink G. Measuring early childhood development at a global scale: Evidence from the Caregiver-Reported Early Development Instruments. *Early Childhood Research Quarterly* 2018; **45:** 58-68.
[i]Frankenburg WK, Dodds J, Archer P, Shapiro H, Bresnick B. The Denver II: a major revision and restandardization of the Denver Developmental Screening Test. *Pediatrics* 1992; **89:** 91-7.
[j]Ertem IO, Dogan DG, Gok CG, Kizilates SU, Caliskan A, Atay G, Vatandas N, Karaaslan T, Baskan SG, Cicchetti DV. A guide for monitoring child development in low-and middle-income countries. *Pediatrics* 2008; **121:** e581-9.
*Candidate tools contributing to the development of the INTERGROWTH-21st Neurodevelopment Assessment (INTER-NDA).

For peer review only

S2 Factors associated with poor neurodevelopmental outcomes in young children[§#]: Characteristics of the normative sample for the INTERGROWTH-21st Project international INTER-NDA Standards, and for other neurodevelopmental tools evaluating two year-old children.

**Factors affecting child development**	**INTERGROWTH-21st Project sample characteristics (N=1209)**	**Characteristics of normative samples, reported and accessible in the public domain, for the following child developmental tools:**									
		**BSID III[a]**	**MDAT[b]**	**Griff-iths[c]**	**RNDA[d]**	**CBCL[e]**	**ASQ III[f]**	**PEDS[g]**	**CREDI[h]**	**DDST II[i]**	**GMCD[j]**
**PRENATAL FACTORS**											
**Genetic Factors:**											
Chromosomal abnormalities	Yes, n=0 (0·0%)	No	Yes^	No	No	No	Yes^	No	No	No	Yes^
Genetic syndromes	Yes, n=0 (0·0%)	No	No	No	No	No	No	No	No	No	No
Sporadic mutations	Yes, n=0 (0·0%)	No	No	No	No	No	No	No	No	No	No
Gene polymorphisms	Yes, n=0 (0·0%)	No	No	No	No	No	No	No	No	No	No
Trinucleotide repeat disorders	Yes, n=0 (0·0%)	No	No	No	No	No	No	No	No	No	No
Metabolic disorders	Yes, n=0 (0·0%)	No	No	No	No	No	No	No	No	No	No
**Intrauterine (materno-feto-placental) factors:**											
Chronic maternal illness	Yes, n=12 (0·9%)[1]	No	No	No	No	No	No	No	No	No	No
Maternal infections (including HIV, rubella, syphilis, hepatitis B, CMV, toxoplasmosis, tuberculosis and malaria)	Yes, n=0 (0·0%)	No	No	No	No	No	No	No	No	No	No
Maternal anaemia	Yes, mean Hb 12·5 (SD1·0)	No	No	No	No	No	No	No	No	No	No
Maternal malignancy	Yes, n=0 (0·0%)	No	No	No	No	No	No	No	No	No	No
Poor maternal nutrition (under- and overnutrition)	Yes, mean BMI 23.4 (SD 7·5)	No	No	No	No	No	No	No	No	No	No
Maternal substance abuse (including alcohol) and smoking	Yes, n=0 (0·0%)	No	No	No	No	No	No	No	No	No	No
Teratogenic drugs	Yes, n=0 (0·0%)	No	No	No	No	No	No	No	No	No	No
Toxins (lead, mercury, and arsenic)	No	No	No	No	No	No	No	No	No	No	No
Anxiety and depression/mental stress	Yes, n=0 (0·0%)	No	No	No	No	No	No	No	No	No	No
Preeclampsia and eclampsia	Yes, n=0 (0·0%)	No	No	No	No	No	No	No	No	No	No
Placental structural anomalies	Yes, n=0 (0·0%)	No	No	No	No	No	No	No	No	No	No
Liquor volume (oligo and polyhydramnios)	Yes, n=28 (2·3%)	No	No	No	No	No	No	No	No	No	No
Intrauterine infections (prolonged rupture of membranes and chorioamnionitis)	Yes, n=145 (11·9%)	No	No	No	No	No	No	No	No	No	No
Fetal behaviour	No	No	No	No	No	No	No	No	No	No	No
Fetal growth restriction	Yes, n=0 (0·0%)	No	No	No	No	No	No	No	No	No	Yes^
Multiple gestation	Yes, n=0 (0·0%)	No	No	No	No	No	Yes^	No	No	No	Yes
**External factors :**											
Maternal access to health care	Yes, n=1209	No	No	No	No	No	No	No	No	No	Yes

For peer review only

	(100·0%)										Yes
Maternal access to prenatal care	Yes, n=1209 (100·0%)	No	No	No	No	No	No	No	No	No	No
Exposure to radiation	No	No	No	No	No	No	No	No	No	No	No
Trauma	Yes, n=0 (0·0%)	No	No	No	No	No	No	No	No	No	Yes
**Socio-Maternal:**											
SES/Poverty	Yes	Yes	No	No	Yes	No	Yes	No	Yes	No	Yes
Hunger due to lack of money to buy food	Yes, n=0 (0·0%)	No	No	No	No	No	Yes	No	Yes	No	Yes^
Maternal education	Yes[2]	Yes	No	No	Yes	No	Yes	No	Yes	No	No
Paternal education	No	No	No	No	Yes	No	No	No	No	No	No
Maternal age at the time of delivery	Yes; mean=28.9 years (SD 3.8)	No	No	No	No	No	Yes	No	No	No	No
Involvement of social services/child protection agencies with family	No	No	No	No	No	No	Yes	No	No	No	No
Domestic violence or abuse	No	No	No	No	No	No	Yes	No	No	No	No
State of local/regional violence	Yes	No	No	No	No	No	Yes	No	No	No	No
Maternal employment in very physically demanding work during pregnancy	Yes, n=0 (0·0%)	No	No	No	No	No	No	No	No	No	No
Maternal occupation with risk of exposure to chemicals or toxic substances	Yes, n=0 (0·0%)	No	No	No	No	No	No	No	No	No	No
**BIRTH FACTORS**											
Prematurity (born at <37 weeks' gestation)	Yes, n=0 (0·0%)	No	Yes	No	No	No	Yes	No	No	No	Yes
Birth weight <2500 g	Yes, n=43 (3·5%)	No	No	No	No	No	Yes	No	No	No	Yes
Fetal distress prior to birth as adjudged by fetal heart rate monitoring and/or cord blood sampling	Yes, n=64 (5·3%)	No	No	No	No	No	No	No	No	No	Yes^
Resuscitation of newborn	Yes, n=36 (2·9%)	No	No	No	No	No	No	No	No	No	Yes^
**POSTNATAL FACTORS**											
**Maternal Factors:**											
Maternal mental health/maternal depression	Yes, n=0 (0·0%)	No	No	No	No	No	No	No	No	No	No
Maternal exposure to abuse and violence	Yes, n=0 (0·0%)	No	No	No	No	No	Yes	No	No	No	No
Parenting style: cognitive stimulation, caregiver sensitivity and responsiveness to the child, and caregiver affect (emotional warmth or rejection of child)	No	No	No	No	No	No	No	No	Yes	No	No
**Neonatal Factors:**											
Neonatal sepsis, including congenital and intracranial infections	Yes, n=7 (0·6%)	No	No	No	No	No	Yes^	No	No	No	Yes^
Neonatal seizures	Yes, n=0 (0·0%)	No	No	No	No	No	Yes^	No	No	No	Yes^

Prolonged ventilation	Yes, n=0 (0·0%)	No	No	No	No	No	Yes^	No	No	No	Yes^
Hypotension requiring inotropic support	Yes, n=0 (0·0%)	No	No	No	No	No	Yes^	No	No	No	Yes^
Intraventricular haemorrhage grade 2 or greater	Yes, n=0 (0·0%)	No	No	No	No	No	Yes^	No	No	No	Yes^
Hypoxic ischaemic encephalopathy	Yes, n=1 (0·1%)	No	No	No	No	No	Yes^	No	No	No	Yes^
Developmental abnormalities	Yes, n=7 (0·6%)	No	No	No	No	No	Yes^	No	No	No	Yes^
Cardiac diagnosis	Yes, n=1 (0·1%)[3]	No	No	No	No	No	Yes^	No	No	No	Yes^
Hyperbilirubinaemia, not requiring exchange transfusion	Yes, n=49 (4·0%)	No	No	No	No	No	Yes^	No	No	No	Yes^
Use of medications, including antibiotics, in neonatal period	Yes, n=141 (11·7%)	No	No	No	No	No	Yes^	No	No	No	Yes^
Breastfeeding at postnatal discharge from hospital	Yes, n=1123 (92·9%)	No	No	No	No	No	Yes^	No	No	No	Yes^
**Infant and Toddler Factors:**											
Adequate physical growth measured as per standardised protocols and growth charts	Yes, n=1209 (100·0%)	No	Yes	No	Yes	No	No	No	Yes	No	Yes
Breastfeeding and appropriate weaning	Yes[4]	No	No	No	No	No	No	No	No	No	Yes^
Micronutrient deficiencies, including iron, iodine and zinc	Yes, n=1 (0·1%)	No	No	No	No	No	Yes^	No	No	No	Yes
Severe infectious diseases (HIV, malaria, tuberculosis, meningitis)	Yes, n=0 (0·0%)	No	No	No	No	No	Yes	No	No	No	Yes
Cerebral palsy	Yes, n=0 (0·0%)	No	No	No	No	No	Yes^	No	No	No	Yes^
Neurological disorders	Yes, n=0 (0·0%)	No	No	No	No	No	Yes^	No	No	No	Yes^
Seizures	Yes, n=0 (0·0%)	No	No	No	No	No	Yes^	No	No	No	Yes^
Long-term health issues including metabolic, endocrinological and surgical conditions	Yes, n=0 (0·0%)	No	No	No	No	No	Yes^	No	No	No	Yes^
Exposure to environmental toxins such as lead, arsenic, manganese and pesticides	Yes, varies (<10%)[k]	No	No	No	No	No	No	No	No	No	No
Exposure to radiation	Yes, varies (<10%)[k]	No	No	No	No	No	No	No	No	No	No
Exposure to social adversity (e.g. neighbourhood crime)	Yes, varies (<10%)[k]	No	No	No	No	No	No	No	No	No	No
Trauma, including road traffic accidents and non-accidental injury	Yes, n=0 (0·0%)	No	Yes	No	No	No	Yes	No	No	No	No
Significant health conditions during the first 2 years of life, requiring prolonged hospitalisation	Yes, n=0 (0·0%)	No	Yes	No	No	No	Yes^	No	No	No	Yes^
Delayed acquisition of developmental milestones or neurosdisability as reported by parents/healthcare workers	Yes, n=0 (0·0%)	No	No	No	No	Yes	Yes^	No	Yes^	No	Yes^
Parental concerns	Yes, n=0 (0·0%)	No	No	No	No	Yes	Yes^	No	No	No	Yes^

BSID III: The Bayley Scales of Infant Development – III edition (BSID III)
MDAT: The Malawi Developmental Assessment Tool
Griffiths: The Griffiths Mental Development Scales
RNDA: The Rapid Neurodevelopmental Assessment

For peer review only

CBCL: Pre-school version of Child Behavior Checklist
ASQ III: The Ages and Stages Questionnaire III edition
PEDS: The Parents' Evaluation of Developmental Status
CREDI: Caregiver-reported Early Developmental Instruments
DDST II: Denver Development Screening Test II
GMCD: Guide for Monitoring Child Development

Yes: Factor in column 1 has been reported in published literature about the tool's normative sample, or has been stated, in published literature, to have been considered in the evaluation of the tool's normative sample
No: Factor in column 1 has not been reported in published literature about the tool's normative sample, or has not been stated, in published literature, to have been considered in the evaluation of the tool's normative sample
^Implied as stated as "healthy" and "low risk", specific descriptions of each predictor not stated.
§ Walker SP, Wachs TD, Gardner JM, Lozoff B, Wasserman GA, Pollitt E, Carter JA, International Child Development Steering Group. Child development: risk factors for adverse outcomes in developing countries. *The lancet* 2007; **369:** 145-57.
# Fernandes M, Srinivasan K, Menezes G, Ramchandani PG. Prenatal depression, fetal neurobehavior, and infant temperament: Novel insights on early neurodevelopment from a socioeconomically disadvantaged Indian cohort. *Development and Psychopathology* 2018; **30:** 725-42.

[1]Chronic respiratory illness, including asthma n=4 (0·3%), endocrinological conditions including hypothyroidism n=7(0·6%), other n=1 (0·08%)
[2]Primary education n=62 (5·1%); Secondary education n=281 (23·2%); Professional/technical training n=198 (16·4%); University n=668 (55·3%)
[3]Patent ductus arteriosus
[4]Duration of exclusive breastfeeding: median 5.6 months (IQR 4.0, 6.0); Age of introduction of formula feeds: median 4.2 months (IQR 2.0, 6.0); age at introduction of first solids; median 6.0 months (IQR 5.5, 6.5)

[a]©Pearson. Bayley Scales of Infant and Toddler Development, Third Edition (Bayley-III) - Training. Available from: 98 http://www.pearsonclinical.co.uk
[b]Gladstone M, Lancaster GA, Umar E, Nyirenda M, Kayira E, van den Broek NR, Smyth RL. The Malawi Developmental Assessment Tool (MDAT): the creation, validation, and reliability of a tool to assess child development in rural African settings. *PLoS medicine* 2010; **7:** e1000273.
[c]Luiz D, Barnard A, Knoesen N, Kotras N, Horrocks S, McAlinden P, Challis D, O'Connell R. Griffiths Mental Development Scales—Extended Revised: Two to Eight Years: Administration Manual. Hogrefe, Oxford, UK 2006.
[d]Achenbach TM. Manual for the Child Behavior Checklist/4-18 and 1991 profile. University of Vermont, Department of Psychiatry 1991.
[e]Khan NZ, Muslima H, Begum D, Shilpi AB, Akhter S, Bilkis K, Begum N, Parveen M, Ferdous S, Morshed R, Batra M. Validation of rapid neurodevelopmental assessment instrument for under-two-year-old children in Bangladesh. *Pediatrics* 2010; **125:** e755-62.
[f]Squires J, Bricker DD, Twombly E. Ages & stages questionnaires. Baltimore, MD: Paul H. Brookes 2009.
[g]Glascoe FP. Collaborating with parents: Using Parents' Evaluation of Developmental Status to detect and address developmental and behavioral problems. Ellsworth & Vandermeer Press 1998.
[h]McCoy DC, Waldman M, Team CF, Fink G. Measuring early childhood development at a global scale: Evidence from the Caregiver-Reported Early Development Instruments. *Early Childhood Research Quarterly* 2018; **45:** 58-68.
[i]Frankenburg WK, Dodds J, Archer P, Shapiro H, Bresnick B. The Denver II: a major revision and restandardization of the Denver Developmental Screening Test. *Pediatrics* 1992; **89:** 91-7.
[j]Ertem IO, Dogan DG, Gok CG, Kizilates SU, Caliskan A, Atay G, Vatandas N, Karaaslan T, Baskan SG, Cicchetti DV. A guide for monitoring child development in low-and middle-income countries. *Pediatrics* 2008; **121:** e581-9.
[k]Eskenazi B, Bradman A, Finkton D, Purwar M, Noble JA, Pang R, Burnham O, Cheikh Ismail L, Farhi F, Barros FC, Lambert A. A rapid questionnaire assessment of environmental exposures to pregnant women in the INTERGROWTH-21st Project. *BJOG: An International Journal of Obstetrics & Gynaecology* 2013; **120:** 129-38.

For peer review only

S3 The INTERGROWTH-21st Fetal Growth Longitudinal Study (FGLS) individual participant entry criteria for mothers at the time of antenatal booking in the first trimester of pregnancy

**Maternal eligibility criteria at booking (<14 weeks of gestation)**	
1.	Aged ≥18 and <35 years.
2.	Body mass index ≥18·5 and <30 kg/m^2.
3.	Height ≥153 cm.
4.	Singleton pregnancy.
5.	A known last menstrual period with regular cycles (defined as 28 ± 4 days) without hormonal contraceptive use, or breastfeeding in the 2 months before pregnancy.
6.	Natural conception.
7.	No relevant past medical history, with no need for long-term medication (including fertility treatment and over-the-counter medicines, but excluding routine iron, folate, calcium, iodine or multivitamin supplements).
8.	No evidence of socio-economic constraints likely to impede fetal growth identified using local definitions of social risk.
9.	No use of tobacco or recreational drugs such as cannabis in the 3 months before or after becoming pregnant.
10.	No heavy alcohol use (defined as >5 units (50 ml pure alcohol) per week) since becoming pregnant.
11.	No more than one miscarriage in the two previous consecutive pregnancies.
12.	No previous baby delivered preterm (<37^{+0} weeks of gestation) or with a birthweight <2500 g or >4500 g.
13.	No previous neonatal or fetal death, previous baby with any congenital malformations, and no evidence in present pregnancy of congenital disease or fetal anomaly.
14.	No previous pregnancy affected by pre-eclampsia/eclampsia, HELLP syndrome or a related pregnancy-associated condition.
15.	No clinically significant atypical red cell alloantibodies.
16.	Negative urinalysis.
17.	Systolic blood pressure <140 mmHg and diastolic blood pressure <90 mmHg.
18.	No diagnosis or treatment for anaemia during this pregnancy (haemoglobin levels will be monitored throughout pregnancy).
19.	No clinical evidence of any other sexually transmitted diseases, including syphilis and clinical trichomoniasis.
20.	Not in an occupation with risk of exposure to chemicals or toxic substances, or very physically demanding activity to be evaluated by local standards. Also women should not be conducting vigorous or contact sports, such as scuba diving or similar activities.

For peer review only

S4 The INTERGROWTH-21st Neurodevelopment Assessment (INTER-NDA) data recording form

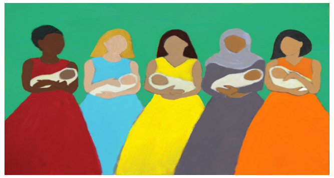

The INTERGROWTH-21st Neurodevelopment Assessment INTER-NDA

Name of child: ______________________ Date of birth: __/__/_____ Date of assessment: __/__/_____

Name of assessor: ____________________

No.	Item	Observed Performance				
1	**Builds a tower of 5 cubes**<br>*(trials=3, demonstration=3)*	5 cubes	3-4 cubes	2 cubes	No attempt	Unable to assess
2	**Names 4 colours when asked to do so**<br>*(trials=1, demonstration=0)*	Names 4 colours	Names 3 colours	Names 1 or 2 colours	Does not name any colour	Unable to assess
3	**Matches 3 cubes of same colours when requested to do so**<br>(trials=1, demonstration=1 of one colour)	Matches 3 colours	Matches 2 colours	Matches 1 colour	Does not match any colour	Unable to assess
4	**Hands the examiner one cube when asked to do so (Examiner says "Please give me one cube" & keeps palm open for 5 seconds after child has handed over 1 cube)**<br>*(trials=1, demonstration=0)*	Hands only one block within 5 seconds	Hands only one block in more than 5 seconds	Hands two or more blocks	Does not hand any block / No attempt	Unable to assess
5	**Puts the spoon in the cup when asked to do so**<br>*(trials=5, demonstration=0)*	Puts the spoon in cup in ≤3 trials	Puts the spoon in cup in 4-5 trials	Takes the spoon or the cup but does not complete action	No attempt	Unable to assess

For peer review only

6	**Matches shapes on board**<br>*(trials=5, demonstration=partial – removal only)*	All shapes in ≤3 trials	All shapes with repeated demonstration i.e. 4-5 trials	One or two shapes in 4-5 trials	No attempt	Unable to assess
7	**Matches shapes on rotated board**<br>*(trials=5, demonstration=partial – removal only)*	All shapes in ≤3 trials	All shapes with repeated demonstration i.e. 4-5 trials	One or two shapes in 4-5 trials	No attempt	Unable to assess
8	**Points correctly when asked "Where is the door/entrance to the room?"**<br>*(trials=5, demonstration=0)*	Identifies door correctly in ≤3 trials	Identifies door correctly in 4-5 trials	Attempts, but does not identify door	No attempt	Unable to assess
9	**Puts a raisin precisely inside a small opening in a bottle**<br>*(trials=1, demonstration=1, test both hands)*	Precise release of raisin into bottle with each hand	Clumsy release, raisin falls out of bottle with one or more hand	Attempts but unsuccessful release with one or more hand	No attempt	Unable to assess
10	**Drinks water from cup/bottle/sippy cup when placed in front of child**<br>*(trials=1, demonstration=0; maternal recall if observation not possible)*	Drinks water from cup/sippy cup without spilling	Drinks clumsily & spills	Attempts but unsuccessful	No attempt	Unable to assess
11	**Looks towards an object located across the room when pointed at by the examiner**<br>(trials=5)	Looks or points at object in ≤3 trials	Looks or points at object in 4-5 trials	Looks at the wrong object, or attempts but cannot identify object	No attempt	Unable to assess
12	**Pretends to drink from a toy cup when placed in front of him/her**<br>*(trials=2, demonstration=1 if not spontaneous on first attempt)*	Spontaneously	After 1 demonstration	Partial attempt after 1 demonstration	No attempt	Unable to assess
13	**Able to make a cup of tea with the toy tea set when requested by examiner (Examiner says "Can you make a cup of tea?")**<br>*(trials=2, demonstration=1 if not spontaneous on first attempt)*	Spontaneously, with pouring motion	After 1 demonstration	Partial attempt after 1 demonstration	No attempt	Unable to assess

14	**Feeds doll when requested to (Examiner says "Can you give the dolly some tea?")** *(trials=2, demonstration=1 if not spontaneous on first attempt)*	Spontaneously	After 1 demonstration	Partial attempt after 1 demonstration	No attempt	Unable to assess
15	**Imitates straight horizontal scribble** *(trials=5, demonstration=5)*	≤3 trials	4-5 trials; with difficulty	Attempts (hold crayon)	Cannot hold crayon	Unable to assess
16	**Identifies glitter bracelet under correct washcloth** *(trials=5, demonstration=0, test both sides)*	Finds bracelet correctly in ≤2 trails on both sides	Find bracelet correctly in 3 trials or on one side only	Find bracelet correctly in 4-5 trials or on one side only	Does not find bracelet or no attempt	Unable to assess
17	**Correctly identifies object groups using plurals** *(concurrent observation)*	Uses 5 plurals	Uses 3-4 plurals	Uses 1-2 plurals	Does not use any plurals	Unable to assess
18	**Asks for toilet by gesture or verbally** *(maternal recall)*	Always	Occasionally	Partial (only for bowel movement)	Never	Unable to assess
19	**Runs** *(maternal recall)*	Runs steadily	Attempts	Walks only	Walks with support	Unable to assess
20	**Throws a ball very near** *(trials=1, demonstration=1; test both hands)*	Good release	Unsteady release	Attempts	No attempt	Unable to assess
21	**Kicks ball** *(maternal recall)*	Kicks ball with knee flexed	Runs after ball & attempts kicking it	Walks and touches ball with foot	No attempt	Unable to assess
22	**Climbs upstairs holding rail, 2 feet/stair or in adult fashion** *(maternal recall)*	Climbs stairs alone steadily	Climbs stairs alone unsteadily	Climbs stairs with help (uses railing, holds adult's hand)	No attempt	Unable to assess
23	**Uses 2-4 syllable babble such as dada, mama but not specifically to anything or any person** *(concurrent observation)*	Spontaneously	Mimics	1 syllabe babble e.g. ba, ma, da	None	Unable to assess
24	**Use two words together** *(concurrent observation)*	Two words, appropriate use	Two words, inappropriate use	One word, appropriate use	No attempt	Unable to assess

25	**Indicates by gesture to say no**<br>*(concurrent observation or maternal recall)*	Indicates verbally or by definite gesture all the time	Indicates verbally or by definite gesture some of the time	Attempts, but incomplete indication	No attempt	Unable to assess
26	**Use of a pronoun e.g. me, my, she, he, it, I**<br>*(concurrent observation)*	≥1 pronoun in correct context	≥1 pronoun, incorrect use	Use of proper names but not pronouns	No use	Unable to assess
27	**How many words does the child use during the assessment other than mama/dada**<br>*(concurrent observation)*	≥8 words	6-7 words	4-5 words	≤3 words	Unable to assess
28	**How many sentences of 3 words or more does the child use during the assessment?**<br>*(concurrent observation)*	≥2	1	≥1 two word utterance	None	Unable to assess
29	**In how many instances does the child follow on a topic of conversation providing new information?**<br>*(concurrent observation)*	At least one, using ≥ 2 words, proving correct information	At least one, uses single words, provides correct information	Uses any number of words, provides incorrect information	Does not follow up on conversations	Unable to assess
30	**Combines word and gesture when asked**<br>*(Do not demonstrate, trials=3, use different example if mother says child does not know the one you are asking)*	Combines word and gesture completely and appropriately	Combines word and gesture completely but inappropriately	Combines word and gesture incompletely and inappropriately	Does not combine a word an gesture	Unable to assess

What is the child's native (first) language? ______________________________

What is the language in which the assessment is being conducted in? ______________________________

Does the child speak/understand any languages other than his/her native (first) language? ______________________________

For peer review only - http://bmjopen.bmj.com/site/about/guidelines.xhtml

How often were the following behaviours in the child during the assessment?				
31	**Positive Affect**	Never or rarely	Some of the time	Most of the time
32	**Exploration**	Never or rarely	Some of the time	Most of the time
33	**Ease of engagement**	Never or rarely	Some of the time	Most of the time
34	**Cooperativeness**	Never or rarely	Some of the time	Most of the time
35	**Adaptability to change**	Never or rarely	Some of the time	Most of the time
36	**Distractibility**	Never or rarely	Some of the time	Most of the time
37	**Negative Affect**	Never or rarely	Some of the time	Most of the time

For peer review only

S5 The INTERGROWTH-21st Neurodevelopment Assessment (INTER-NDA) Protocol Adherence Checklist

	The INTERGROWTH-21st Project<br>Neurodevelopment Assessment Package<br>**Protocol Adherence Checklist**	**NDA-PAC**<br>**Page 2 of 2**

**Participant ID No.**	☐☐ - ☐☐☐☐☐	**Researcher code: Expert**	☐☐
**Child Date of Birth**	D D M M Y Y	**Researcher code: Assessor**	☐☐
**Visit Date**	D D M M Y Y		

Please rate the performance of the assessor in adhering to the INTERGROWTH-21st Neurodevelopment Assessment Protocol during the administration of the following items. For the purpose of this exercise, complete adherence is defined as adherence to all aspects of the protocol as stated in the operation manual of the INTER-NDA; partial adherence is defined as adherence to half or more aspects, but not all aspects, of the protocol as stated in the operation manual of the INTER-NDA and limited adherence is defined as adherence to less than half of the aspects of the protocol as stated in the operation manual of the INTER-NDA as adjudged by the expert assessor.

**IV. The INTER-NDA**	Complete adherence	Partial adherence	Limited adherence
1. Item 1 - uses 5 red cubes, 3 trials, demonstration before each trial			
2. Item 2 - 4 cubes of different colours placed in a line, assessor does not name colours, 1 trial			
3. Item 3 - 1 demonstration, 1 trial, conceptually correct scoring (stacking & pointing accepted)			
4. Item 4 - holds hand out for 5 seconds			
5. Item 5 - correct placement of objects - handles facing child, cup & spoon not adjacent, 5 trials			
6. Item 6 - 5 trials, no demonstration, shapes near child, holds board, puts shapes back discretely			
7. Item 7 - 5 trials, no demonstration, shapes near child, rotates board on table, holds board			
8. Item 9 - 1 demonstration, 1 trial, tests both hands			
9. Item 11 - 5 trials, no demonstration, relatively slow pointing movement so child can follow			
10. Item 12 - allows time for self-symbolic play before suggestion & demonstration			
11. Item 13 - 2 trials, demonstrate if not spontaneous, involve mother if necessary			
12. Item 14 - 2 trials, demonstrate if not spontaneous, involve mother if necessary			
13. Item 15 - 5 trials, demonstration before each trial, start at upper part of paper			
14. Item 16 - 5 trials, displaces bracelet relatively slowly, tests both sides			
15. Item 20 - 1 demonstration, 1 trial, tests both hands			
16. Item 30 - uses opportunities during assessment, uses culturally appropriate combinations, informs mothers about purpose & rationale of item			
17. Assessor's interaction with the child (good verbal & nonverbal communication, sets child at ease, builds rapport, accomodates needs of the child)			
18. Assessor's interaction with the mother/caregiver (explains tests, builds rapport, involves mother in testing providing instructions to prevent compromising test)			

Name of expert researcher	
Signature	

S6 Results of the INTERGROWTH-21st Neurodevelopment Assessment (INTER-NDA) standardized evaluations between specialist and non-specialist assessors to (a) administer and (b) score the INTER-NDA

S6 (a) Comparisons in protocol adherence scores for the INTER-NDA between non-specialist and specialist assessors.

	**Median (95% CI)**		**Comparison between groups**
	**Non-specialist assessors** (n=4)	**Specialist assessors** (n=3)	
**INTER-NDA Protocol Adherence Score**	53.0 (43.4 – 53.6)	52.0 (46.2 – 54.2)	U=27.5, p=0.8
**INTER-NDA Protocol Adherence %**	98.0 (80.4 – 99.2)	96.3 (85.5 – 99.3)	U=30.5, p=1.0

For peer review only

S6 (b) Comparisons in INTER-NDA domain scores between non-specialist and specialist assessors.

		Neurodevelopment scores (n=23)					
		**Cognition**	**Language**	**Gross motor**	**Fine motor**	**Positive behavior**	**Negative behavior**
		Mean (SD)	***Mean (SD)***	***Mean (SD)***	***Mean (SD)***	***Mean (SD)***	***Mean (SD)***
**Video/Child 1**	**Field workers** (n=10)	21.1 (2.0)[a]	17.7 (3.7)	4.6 (0.9)	4.6 (0.9)	14.9 (0.3)	2.4 (0.7)
	**Healthcare professionals** (n=13)	24.2 (2.2)[a]	17.1 (3.1)	5.1 (0.8)	5.1 (1.0)	14.5 (0.9)	2.3 (0.5)
**Video/Child 2**	**Field workers** (n=10)	19.4 (1.7)	31.4 (2.7)	3.2 (0.4)	4.0[§]	12.7 (1.3)	3.8 (0.4)
	**Healthcare professionals** (n=13)	19.5 (1.4)	30.9 (3.1)	5.5 (2.6)	4.5 (1.0)	12.5 (1.3)	3.9 (0.5)
**Video/Child 3**	**Field workers** (n=10)	21.9 (2.0)	25.0 (2.9)	4.0 (0.9)	7.6 (0.7)	12.4 (1.3)	3.3 (0.5)
	**Healthcare professionals** (n=13)	22.6 (1.9)	26.8 (5.3)	3.9 (0.8)	5.7 (1.4)	11.0 (1.6)	3.9 (0.7)

[a]t=-3.2, p<0.001. [§]As score for this group is identical for all test subjects within this group no SD has been reported

S7 The INTERGROWTH-21st Neurodevelopment Assessment (INTER-NDA) scoring system and interpretation of domain scores.

**INTER-NDA domain**	Number of items contributing to domain	Constituent item numbers	Method of domain estimation	Interpretation of score
Cognitive	13	1,2,4,5,6,7,8, 11,12,13,14,16,18	Mean of constituent item scores	Higher score reflects better performance
Fine motor	4	9,10,15,20	Mean of constituent item scores	Higher score reflects better performance
Gross motor	3	19,21,22	Mean of constituent item scores	Higher score reflects better performance
Language	12	3,5,8,17,23,24,25,26,27,28,29,30	Mean of constituent item scores	Higher score reflects better performance
Positive behaviour	5	31,32,33,34,35	Mean of constituent item scores	Higher score reflects better performance
Negative behaviour	2	36,37	Mean of constituent item scores	Lower score reflects better performance

For peer review only

S8 Formulae and tables for the conversion of raw scores to standardised (scaled) scores (range 0-100)

**Domain**	**Min - Max**	**Scaling formula**
Cognitive, fine motor, gross motor and language	1 – 4	((x – 1) / 3)) * 100
Positive and negative behaviour	1 – 3	((x – 1) / 2)) * 100

**Domain conversion table (selected values) for cognitive, motor (fine and gross), and language domains**	
**Raw mean score**	**Scaled mean score**
1·00	0·0
1·25	8·3
1·50	16·7
1·75	25·0
2·00	33·3
2·25	41·7
2·50	50·0
2·75	58·3
3·00	66·7
3·25	75·0
3·50	83·3
3·75	91·7
4·00	100·0

For peer review only

**Domain conversion table (selected values) for positive and negative**	
**Raw mean score**	**Scaled mean score**
1·0	0·0
1·2	10·0
1·4	20·0
1·6	30·0
1·8	40·0

2·0	50·0
2·2	60·0
2·4	70·0
2·6	80·0
2·8	90·0
3·0	100·0

For peer review only

Supplementary Information S9 INTER-NDA domain scores centiles including and excluding children scoring above the CBCL threshold for clinical (97th centile) problems.

INTER-NDA domain	Pooled Centiles for children including children scoring above the 97th CBCL centiles for attentional problems and/or emotional reactivity (n=1209)							Pooled Centiles for children excluding children scoring above the 97th CBCL centiles for attentional problems and/or emotional reactivity (n=1181)						
	**c3**	**c10**	**c25**	**c50**	**c75**	**c90**	**c97**	**c3**	**c10**	**c25**	**c50**	**c75**	**c90**	**c97**
**Cognitive**[1]	27.3	38.5	60.9	79.2	88.2	92.6	99.6	27.4	38.5	62.2	79.5	88.8	92.6	99.6
**Fine motor**[1]	17.4	25.2	71.5	90.4	100.0	100.0	100.0	17.5	25.7	74.2	91.4	100.0	100.0	100.0
**Gross motor**[1]	31.9	51.6	66.7	81.5	100.0	100.0	100.0	31.1	51.7	66.7	81.6	100.0	100.0	100.0
**Language**[1]	12.1	17.0	44.6	70.7	88.0	94.9	100.0	12.1	17.8	45.7	71.7	88.5	95.1	100.0
**Positive behaviour**[1]	32.8	50.3	69.7	90.0	100.0	100.0	100.0	37.8	51.4	70.0	90.0	100.0	100.0	100.0
**Negative behaviour**[2]	0.0	0.0	0.0	25.0	25.1	50.1	83.0	0.0	0.0	0.0	25.0	25.0	50.0	76.5

INTER-NDA: The INTERGROWTH-21st Neurodevelopment Assessment
[1]For these domains, higher scores reflect better outcomes
[2]For negative behaviour, lower scores reflect better outcomes

For peer review only

S10 Comparison of INTERGROWTH-21st Neurodevelopment Assessment (INTER-NDA) domain scores between two year-old girls and boys

	Centiles for girls (n=617)							Centiles for boys (n=564)							Girls (n=617)	Boys (n=564)	
**INTER-NDA Domain**	**c3**	**c10**	**c25**	**c50**	**c75**	**c90**	**c97**	**c3**	**c10**	**c25**	**c50**	**c75**	**c90**	**c97**	**Median (IQR)**	**Median (IQR)**	**p-value***
**Cognitive**[1]	27.2	38.9	64.3	81.7	89.7	93.0	99.5	27.0	38.2	58.3	76.9	86.8	92.6	99.2	82.1 (64.1, 89.7)	76.9 (59.0, 87.2)	0·001
**Fine motor**[1]	18.7	28.3	74.6	91.6	100.0	100.0	100.0	17.6	25.3	68.1	86.0	100.0	100.0	100.0	91.7 (75.0, 100.0)	83.3 (66.7, 100.0)	0·062
**Gross motor**[1]	27.6	52.7	66.7	83.1	100.0	100.0	100.0	32.4	52.5	66.7	79.6	100.0	100.0	100.0	83.3 (66.7, 100.0)	77.8 (66.7, 100.0)	0·318
**Language**[1]	13.9	22.1	49.5	75.7	90.1	97.1	100.0	11.6	16.8	40.0	65.6	85.2	93.9	100.0	75.8 (50.0, 90.0)	66.7 (38.9, 86.1)	<0·001
**Positive behaviour**[1]	34.1	51.7	70.4	90.0	100.0	100.0	100.0	39.5	52.4	68.2	90.0	100.0	100.0	100.0	90.0 (70.0, 100.0)	90.0 (70.0, 100.0)	0·100
**Negative behaviour**[2]	0.0	0.0	0.0	16.1	25.0	50.0	75.6	0.0	0.0	0.0	25.0	29.4	50.6	83.6	25.0 (0.0, 25.0)	25.0 (0.0, 25.0)	<0·001

INTER-NDA: The INTERGROWTH-21st Neurodevelopment Assessment
*p value from Wilcoxon rank-sum test
[1]For these domains, higher scores reflect better outcomes
[2]For negative behaviour, lower scores reflect better outcomes
IQR: inter-quartile range

For peer review only

The International Fetal and Newborn Growth Standards for the 21st Century

INTERGROWTH-21st

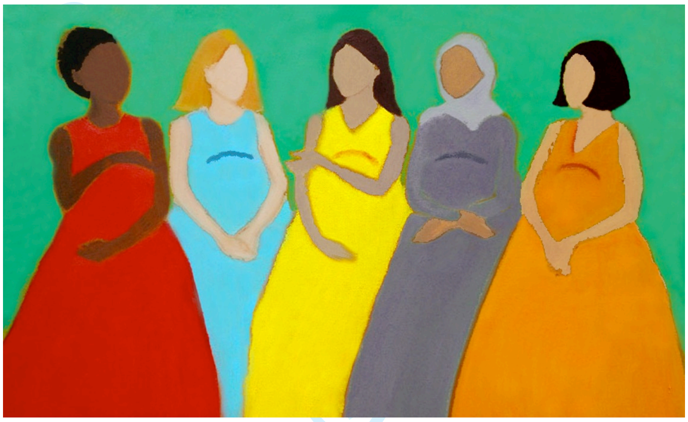

Study Protocol

International Fetal and Newborn Growth Consortium

The Aga Khan University Hospital, Kenya; **Aga Khan University Medical Centre**, Pakistan; **Beijing Obstetrics & Gynaecology Hospital,** China; **Ketkar Nursing Home**, India; **Universidade Federal de Pelotas**, Brazil; **Ministry of Health**, Sultanate of Oman; **University of Oxford**, UK; **Department of Nutrition for Health & Development, WHO**, Switzerland; **Università di Torino**, Italy, **University of Washington**, USA

UNIVERSITY OF OXFORD

v.6 November 2012

For peer review only - http://bmjopen.bmj.com/site/about/guidelines.xhtml

Nuffield Department of Obstetrics & Gynaecology, University of Oxford, UK (Professor José Villar; Professor Stephen Kennedy; Dr Aris Papageorghiou);

Universidade Federal de Pelotas, Pelotas, Brazil (Professor Fernando Barros);

Ketkar Nursing Home, Nagpur, India (Dr. Manorama Purwar);

Department of Paediatrics & Child Health, Aga Khan University Medical Centre, Karachi, Pakistan (Professor Zulfiqar Bhutta);

Beijing Obstetrics & Gynaecology Hospital, Maternal and Child Health Center, Capital Medical University, Beijing, China (Professor Pang Ruyan);

Ministry of Health, Muscat, Sultanate of Oman (Dr. Yasmin Jaffer);

The Aga Khan University Hospital, Nairobi; Kenya (Dr. Maria Carvalho);

Department of Obstetrics & Gynaecology, University of Washington, USA (Professor Mike Gravett)

Department of Paediatrics, Università di Torino, Turin, Italy (Professor Enrico Bertino)

This protocol should be referenced as: International Fetal and Newborn Growth Consortium. The International Fetal and Newborn Growth Standards for the 21st Century (INTERGROWTH-21st) Study Protocol, v.6 2012, www.intergrowth21.org.uk

For peer review only

Acknowledgements

This study is supported by a generous grant from the Bill & Melinda Gates Foundation to the University of Oxford, for which we are very grateful.

We would also like to thank the Health Authorities in Brazil, China, India, Italy, Kenya, Oman, the USA and the University of Oxford, whose funding will facilitate the participation of these study sites as collaborating centres.

For peer review only

Table of Contents

I. Brief Summary 5
II. Governance of Study 6
III. Credits 8
IV. List of Abbreviations Used 9
V. Executive Summary 10
VI. Goals and Objectives 13
VII. Project Design and Implementation 14
VIII. Monitoring, Evaluation, and Dissemination 39
IX. Optimizing Public Health Outcomes and Intellectual Property Plans to Achieve Global Access 40
X. Organizational Capacity and Management Plan 42
XI. Citations 44

XII. Appendices 46

Appendix A: Data Collection Forms 46
Appendix B: Data Flow Models 47
Appendix C: Ultrasound Methods And Quality Control 50
Appendix D: Newborn, Preterm and Maternal Anthropometric Techniques, Equipment and Standardization and Data Quality Assurance and Control Plan. 66
Appendix E: Data Management: the Electronic System 67
Appendix F: Policy and determination of responsibilities for INTERGROWTH-21st 68
Appendix G: Management Structure 73
Appendix H: Selection of Study Sites Flow 74
Appendix I: The Pregnancy Physiology Pattern Prediction (4P) Study: Details of observation measurement 76
Appendix J: The PEA POD Manual of Operations 77
Appendix K: The INTERBIO-21st Study Protocol 89

Michel
Delete

For peer review only

I. Brief Summary

Charitable Purpose:

To develop new "prescriptive" standards describing normal fetal growth and newborn nutritional status in eight geographically diverse populations, and to relate these standards to neonatal health risk. The worldwide use of these tools should improve infants' health and nutritional status.

Project Description:

The project aims to develop scientifically robust clinical tools to assess fetal growth and the nutritional status of newborn infants, as adjuncts to the recently produced WHO charts for children aged 0 to 5. These will be incorporated into national and international maternal and neonatal programs, and be used to monitor and evaluate maternal wellbeing, infant health and nutrition at a population level.

To achieve these objectives, primary data will be collected on a population-based sample of healthy pregnant women. The tools will describe how fetuses and newborns *should* grow in *all* countries rather than the more limited objective of past growth references which describe how they have grown at specific times and locations. They will allow for evidence-based evaluation of nutritional status at birth and measurement of the impact of preventive and treatment interventions in the community.

For peer review only

II. Governance of Study

Steering Committee:
- Zulfiqar Bhutta - Chair (Husein Laljee Dewraj Professor and Chairman, Department of Paediatrics and Child Health, Aga Khan University Medical Centre, Karachi, Pakistan)
- José Villar (Professor of Perinatal Medicine, Nuffield Department of Obstetrics & Gynaecology, University of Oxford) Principal investigator
- Stephen Kennedy (Head of Department & Professor of Reproductive Medicine/Honorary Consultant, Nuffield Department of Obstetrics & Gynaecology, University of Oxford)
- Aris Papageorghiou (Consultant/Honorary Senior Lecturer in Obstetrics and Fetal Medicine, St George's, University of London and Senior Fellow in Fetal Medicine, Nuffield Department of Obstetrics & Gynaecology, University of Oxford)
- Alison Noble (Professor of Engineering Science, Institute of Biomedical Engineering, University of Oxford)
- Doug Altman (Director, Centre for Statistics in Medicine, University of Oxford)
- Cesar G. Victora (Professor of Epidemiology, Federal University of Pelotas, Brazil)
- Cameron Chumlea (Fels Professor Departments of Community Health & Pediatrics, Wright State University School of Medicine, Ohio, USA)
- Juan A Rivera (Director of the Centre for Research in Nutrition and Health, The National Institute of Public Health, Professor of Nutrition in the School of Public Health in Mexico)
- Fiona Burton (Counsellor and Lay Member, Oxford)

Executive Committee:
- Doug Altman
- Zulfiqar Bhutta
- Alison Noble
- Aris Papageorghiou
- Stephen Kennedy
- José Villar

Principal Investigators
- Fernando Barros (Brazil)
- Pang Ruyan (China)
- Alejandro Velasco (Cuba)
- Manorama Purwar/Mangala Ketkar (India)
- Tulia Todros/Enrico Bertino (Italy)
- Maria Cavarlho/William Stones (Kenya)
- Ali Jaffar Mohammed/Dr Yasmin Jaffar (Oman)
- Stephen Kennedy (UK)
- Manjiri Dighe/Michael Gravett (USA)

Scientific Advisory Committee
- M K Bhan (Secretary, Dept of Biotechnology, Government of India, New Delhi, India)
- Cutberto Garza (Provost and Dean of Faculties, Boston College, USA)
- Shahida Zaidi (Professor of Obstetrics & Gynaecology, Vice President of International Federation of Gynaecology & Obstetrics, Geneva)
- Ana Langer (Maternal Health Task Force at the Harvard School of Public Health)
- Michael Katz (ex-March of Dimes Senior Vice President for Research and Global Programs, Carpentier Professor Emeritus of Pediatrics and Professor Emeritus of Public Health, Columbia University, New York, USA).
- Peter Rothwell (Division of Clinical Neurology, University of Oxford)

For peer review only

Coordinating Unit
- José Villar (Principal Investigator)
- Stephen Kennedy (Study Director)
- Leila Cheikh Ismail (Project Leader)
- Ann Lambert (Project Administrator)
- Melissa Shorten (Project Secretary)

Ultrasound Group
- Aris Papageorghiou
- Shaida Zaidi
- Laurant Salomon
- Ippokratis Sarris/Cristos Ioannou (UK)
- Adriane Mitidieri (Brazil)
- Sunita Sohoni (India)
- Zhang Yong/Wu Qingqing (China)
- Megha Venkatraman/Waleeda Al-Zadjali (Oman)
- Jyoti Shah (Kenya)
- Manjire Dighe (USA)
- Manuela Oberto (Italy)

Anthropometry Group
- Leila Cheikh Ismail
- Wm. Cameron Chumlea
- Adelheid Onyango
- Zulfiqar Bhutta
- Manuel Sanchez Luna
- Susan Lloyd (UK)
- Claudia Rossi (Italy)
- Fatima Al Rahbi (Oman)
- Lui Hong/Shen Yingjie (China)
- Naina Kunnawar (India)
- Manjiri Dighe (USA)
- Denise Mota (Brazil)
- Roseline Ochieng (Kenya)

Neonatal Group
- Zulfiqar Bhutta
- Ricardo Uauy
- Enrico Bertino (Italy)
- Elaine Albernaz (Brazil)
- Kenny McCormick (UK)
- Bashir Bhat (Oman)
- Roseline Ochieng (Kenya)
- Vikram Rajan (India)
- Maneesh Batra (USA)
- Pang Ruyan (China)

Data Management and Analysis Group
- Doug Altman
- Laima Juodvirsiene
- Fenella Roseman (UK)
- Marlos Domingues (Brazil)
- Naina Kunnawar (India)
- Paolo Gilli (Italy)
- Wang Jun Hua/ Wu Minghui (China)
- Hamood Al-Jabri (Oman)
- Norah Musee (Kenya)
- Sarah Waller (USA)

For peer review only

III. Credits

This protocol was prepared by José Villar, Stephen Kennedy, Paul Chamberlain, Douglas Altman and Alison Noble (University of Oxford) and Mercedes de Onis (WHO Nutrition). These authors produced a first version, between October 2006 and May 2007, based conceptually on the published WHO Multicentre Growth Reference Study (MGRS) protocol. The following people provided comments that were considered for inclusion in the initial version: Daniel Giordano, Mario Merialdi, Guillermo Carroli, as well as several participants at a WHO meeting held in June 2007 where this first version was presented by José Villar, the Principal Investigator. We thank them for their contributions. José Villar had discussions with Daniel Giordano about data collection forms and models for data management, based on available documents and previous studies; some components have been retained in the present study and his input is much appreciated.

Further important contributions were made by Cesar Victora, Fernando Barros, Michael Kramer and Robert Platt to expanded versions of the initial document that eventually constituted the grant application to the Gates Foundation. They also made important contributions to the responses provided by the applicants to the reviewers' comments. The reviewers and staff at the Gates Foundation made considerable contributions and raised a number of important issues, all of which have been incorporated into this version. We are very grateful to all the contributors and in particular to Dr Ellen Piwoz. Finally, members of the study's Steering Committee and potential investigators contributed during the preparatory phase of the study to the final version of this protocol.

During 2008 until February 2009, Jan van den Broeck made contributions to the anthropometric, ultrasound and anthropometric quality control strategies of the study design after the original protocol was approved by the Gates Foundation. Where these suggestions have been retained, appropriate credits are provided in the text.

Overall, this protocol is intended to extend the concept of the MGRS protocol to cover most aspects of growth during fetal and newborn life. Therefore, this document has been developed to comply as much as possible with the WHO MGRS' rationale and procedures. We thank all the investigators and the members of the WHO MGRS Coordinating Unit for allowing us to consult their study material and to use an electronic version of their document during the early preparation of our protocol.

All the individuals mentioned above, have been invited to join the Study team at various stages and in different capacities. We still welcome contributions from those few who have not yet joined us.

DISCLAIMER: WHO has not formally reviewed the INTERGROWTH-21st study protocol. Therefore, the technical advice being provided to INTERGROWTH-21st by WHO staff does not constitute endorsement of, or support for, the research project by WHO.

For peer review only - http://bmjopen.bmj.com/site/about/guidelines.xhtml

IV. List of Abbreviations Used

**AC**	Advisory Committee
**AC**	Abdominal Circumference
**ADQU**	Anthropometric Quality Control Unit
**APAD**	Antero-posterior abdominal diameter
**BMI**	Body Mass Index
**BPD**	Biparietal Diameter
**CRL**	Crown Rump Length
**CU**	Coordinating Unit
**DMU**	Data Management Unit
**EC**	Executive Committee
**FGLS**	Fetal Growth Longitudinal Study
**FL**	Femur Length
**IDAMIS**	INTERGROWTH-21st Electronic Data Management System
**IUGR**	Intrauterine Growth Restriction
**HC**	Head Circumference
**LBW**	Low Birth Weight
**LMP**	Last Menstrual Period
**MGRS**	WHO Multicentre Growth Reference Study
**NCSS**	Newborn Cross-sectional Study
**NDOG**	Nuffield Department of Obstetrics and Gynaecology
**NICU**	Neonatal Intensive Care Unit
**OFD**	Occipito-Frontal Diameter
**OMPHI**	Oxford Maternal and Perinatal Health Institute
**PPFS**	Preterm Postnatal Follow-up Study
**PRC**	Publications Review Committee
**SC**	Steering Committee
**SLE**	Systemic Lupus Erythematosus
**TAD**	Transverse abdominal diameter

For peer review only

V. Executive Summary

At least 60% of the 4 million neonatal deaths that occur worldwide every year are associated with low birth weight (LBW), caused by intrauterine growth restriction (IUGR), preterm delivery, and genetic/chromosomal abnormalities (1), demonstrating that under-nutrition is already a leading health problem at birth. Accurate assessment of fetal growth and gestational age for timely identification and management of growth restriction, risk assessment of undernourished newborns, and monitoring LBW trends are therefore public health priorities, especially in developing countries where 98% of all neonatal deaths occur. The long-term health implications of these conditions are now well recognized for both developed and developing countries.

Traditionally, fetal nutritional status has been assessed by measurement of uterine height in pregnancy, which can be used in first level screening but needs more precision for suspected IUGR. Birth weight, as the final point of intrauterine growth, is also used but does not adequately describe the fetal growth process. Furthermore, the newborn standards presently recommended by WHO are based on a population of births from California, USA, in the 1970s and are unlikely to be a suitable international reference in the 21st century (2). This is a particular problem for preterm babies, that have different growth patterns to fetuses still *in utero*, and for whom there are no high quality postnatal growth standards, i.e. using the prescriptive approach of the new WHO infant and child growth standards (3).

In the developed world and in urban areas in most developing countries, especially if complications are suspected, fetal growth is assessed by comparing ultrasound measurements of fetal size with reference percentiles obtained from fetuses whose growth was assumed to be normal. Unfortunately, most of the ultrasound charts of size by gestational age were obtained from very small populations of fetuses in the USA or Europe, and may not be appropriate for use internationally.

These limitations have generated concern regarding the value of ultrasound in routine clinical care particularly late in pregnancy, as well as when newborn standards are used to determine the health status of populations or monitor trends and progress in reducing under-nutrition. For example, the WHO Expert Committee on "Physical Status: the use and interpretation of anthropometry" (1995) recommended the development of international standards for both fetal and newborn growth, as well as child growth (4). The latter were recently produced by WHO and are being implemented worldwide; the former are still in the research priority agenda (5). We now propose in this project to extend the WHO work by constructing a new set of international **Fetal and Newborn Growth Standards**. It will be the first, population based, large, multi-ethnic, longitudinal, fetal growth standard based on early assessment of gestational age.

Goals and Objectives

The goal is to develop new "prescriptive" standards describing normal fetal and preterm neonatal growth over time and newborn nutritional status, and to relate these to neonatal health risk.

The primary objective is to produce a set of international **Fetal and Newborn Growth Standards** (fetal growth, birth weight for gestational age and postnatal growth of preterm infants) for practical applications in clinical use and for monitoring trends in populations. We will relate the new newborn standards to neonatal morbidity and mortality to identify levels of perinatal risk.

The secondary objectives are:

a) **Clinical:** to develop a prediction model, based on multiple 2-dimensional (2D) ultrasound measurements, for estimating gestational age during mid-late pregnancy for use in populations of pregnant women without access to early/frequent antenatal care;

b) **Epidemiological:** to investigate in this multi-ethnic, population based sample the determinants of LBW and its components (preterm delivery, impaired fetal growth) under current healthcare conditions, and

For peer review only - http://bmjopen.bmj.com/site/about/guidelines.xhtml

c) **Biological:** to acquire additional 3-dimensional (3D) images to create an anatomical and growth databank of individual fetal organs as a unique source of biological information for future research.

Project Design and Implementation

The primary objective has 3 components (Activities 1-3):

1. **Fetal Growth Longitudinal Study (FGLS)** from <14^{+0} weeks gestational age to birth to monitor and measure fetal growth clinically (Symphyseal-fundal-height) and by ultrasound in a healthy population.
2. **Preterm Postnatal Follow-up Study (PPFS)** of preterm infants (>26^{+0} but <37^{+0} weeks) in the FGLS to describe their postnatal growth pattern.
3. **Newborn Cross-sectional Study (NCSS)** of all newborns at the study centres over 12 months, obtaining anthropometric measures and neonatal morbidity and mortality rates.

Eight geographically diverse populations will participate, covering North and South America, Europe, Africa, Western Asia and the Indian Subcontinent. Each will contribute a population-based sample of healthy pregnant women expected to provide full growth potential to their fetuses, making it possible to produce prescriptive standards, similar to the WHO Child Growth Standards. Participating sites will contribute to all the components of the project.

The study populations obtained from these geographically defined areas (e.g. city or county) should have no socio-economic constraints on growth, low morbidity and perinatal mortality and adequate nutritional status. To be included, women should be non-smokers, with a normal pregnancy history, and without health problems likely to influence fetal growth or indicate a risk for pregnancy-related pathological conditions. In **FGLS**, women will be screened before 14^{+0} weeks at the time of their first antenatal visit and followed-up with standard clinical and 2D ultrasound examinations every five weeks, i.e. up to six times during pregnancy. In **PPFS**, preterm infants (>26^{+0} but <37^{+0} weeks) born from this sample will be followed-up during the first 8 months of life with the same protocol and set of anthropometric measures that were used in the WHO Child Growth Study. Postnatal growth will be evaluated from both delivery and conception for comparison with the corresponding *in utero* measurements. In the **NCSS** study, all newborns at the study centres, born during a fixed (e.g. 12 month) period, will have anthropometric measurements taken immediately after birth. Only babies born to women who meet the same inclusion criteria used in FGLS will be selected to construct the newborn standards. Birth weight and gestational age will also be related to neonatal morbidity and mortality outcomes to construct risk-related newborn weight for gestational age standards. Approximately 5,000 pregnancies will be included in FGLS allowing for ~ 350 preterm infants to be included in PPFS. We expect ~500 women will have to be recruited at each study site to achieve the required number of participants. In NCSS, ~50,000 newborns will be recruited, of whom we expect close to 75% will be eligible for the standards yielding ~1,800 cases with severe newborn outcomes.

Standard quality control measures will be included, as well as a unique system of random evaluation and repetition of ultrasound measurements (from stored images) to monitor validity and reliability, and continuous real time assessment of all data collected. A limited number of experienced obstetric ultrasonographers and neonatal anthropometrists will form the research teams at each study site. Neonatal anthropometric measures will be monitored and standardized centrally. All data will be entered and managed in an on-line data management system specifically developed for the study, including a system for direct transfer of blinded data from the ultrasound equipment to the database. This on-line system allows the initiation of data analysis soon after data collection is completed. Results from all populations will be pooled (if biologically relevant differences are not observed between sites) to construct the curves for international applications using the same analytical strategy as in the WHO Child Growth Standards. We expect that extensive secondary analyses will be conducted from these data that will make major contributions to science and clinical practice in the near future (Objectives II and III) and for many years to come. This study involves minimal risk to participants, no greater than at routine antenatal or clinical

For peer review only - http://bmjopen.bmj.com/site/about/guidelines.xhtml

examinations. Ethical approval will be sought at the appropriate levels, informed consent obtained and confidentiality guaranteed.

Monitoring, Evaluation and Dissemination

The project will run from July 2008 to December 2014. The project's implementation will be monitored following standardized practices implemented in previous large multi-centre studies by WHO and the Perinatal Research Network, according to the set of milestones described in the proposal. It is expected that by 2014: a) The newly developed fetal growth standards will be incorporated into all obstetric ultrasound equipment produced worldwide; b) Preterm infants' growth will be evaluated using the new postnatal growth curve, and c) Newborns will be assessed using the new, international birth weight for gestational age standards. Intermediate milestones are: a) Start recruitment by April 2009; b) Complete fetal sample by March 2011; c) Complete follow-up by February 2012; d) Have data set ready for analysis by July of 2012, and e) Present final standards by the end of 2013. The dissemination of the fetal, postnatal and newborn growth standards will utilize the same channels as the roll-out of the WHO Child Growth Standards and will be coordinated by the Maternal Health Task Force at the Harvard School of Public Health.

Optimizing Public Health Outcomes

The new fetal and newborn standards when incorporated into health services will be unique tools for evidence-based estimation of under-nutrition at birth, maternal and perinatal programme monitoring and the clinical care of newborns worldwide, specifically in developing countries where most of these health problem exist. When fully implemented, the growth charts we propose to develop should benefit all pregnancies throughout the world because fetal growth is currently evaluated using clearly limited tools. This should provide major clinical and economic benefits. The preterm and newborn charts should be of greatest benefit in developing countries where most morbidity and mortality amongst newborns (especially growth restricted and preterm babies) occur.

For peer review only

VI. Goals and Objectives

A. Goals and Attributable Benefits

The **goal** is to develop new "prescriptive" standards describing normal fetal and preterm neonatal growth and newborn nutritional status, and relate these to perinatal health risk.

The anticipated **attributable benefits** of this project are that by 2014:

a) The newly developed fetal growth standards will be incorporated into all obstetric ultrasound equipment produced worldwide.
b) Preterm infants' growth will be evaluated using the new postnatal growth curves
c) All newborns worldwide will be assessed using the new, risk related, international birth weight for gestational age standards.

B. Objectives

Primary objective:

I. To produce a set of international **Fetal and Newborn Growth Standards** (fetal growth, birth weight for gestational age and postnatal growth of preterm infants) for practical applications in clinical use and for monitoring trends in populations. The newborn standards will then be related to perinatal morbidity and mortality to identify levels of risk and target interventions.

Secondary objectives:

II. To develop a prediction model, based on multiple 2D ultrasound measurements, for estimating gestational age during mid-late pregnancy for use in populations of pregnant women without access to early/frequent antenatal care.
III. To determine in this multi-ethnic sample the determinants of LBW, as well as associated conditions, e.g. preterm delivery and impaired fetal growth and their subgroups, under current healthcare conditions.
IV. To acquire additional 3D images to create an anatomical and growth databank of individual fetal organs as a unique source of biological information for future research.

To the best of our knowledge, this project is unique because of its comprehensiveness and scale (never attempted before). It has all the characteristics recommended in the literature for constructing ideal standards for international use (6, 7): it is prescriptive, longitudinal, population-based, and gestational age will be established by ultrasound early in pregnancy. We will be able to explore associated research questions, such as possible ethnic differences in fetal/newborn growth, risk factors for LBW and its subgroups. We will also be able to predict gestational age in women with poor antenatal care and simultaneously provide a set of practical tools, which can be introduced into maternal and newborn care worldwide in a short space of time.

There is considerable demand from maternal and neonatal services to WHO and professional organizations for the standards that this study will produce (5). Hence, we have designed this project in consultation with experts in the field, as well as other research groups and institutions, in such a way that it could easily complement other yet to be developed studies, thereby reducing the risk for duplication.

For peer review only

VII. Project Design and Implementation

Background

In April 2006, WHO released the WHO Child Growth Standards for children aged 0 and 5 which were generated by the WHO Multicentre Growth Reference Study (MGRS) (8). Two characteristics made the MGRS unique and unprecedented as a study in its field: 1) The study included populations from several countries (Brazil, Ghana, India, Norway, Oman and the USA) and 2) A prescriptive approach was used to select the study populations, i.e. only children from populations with minimal environmental constraints on growth were included. This was achieved by recruiting children of affluent and educated parents, because high education and family income have been identified as the environmental variables most likely to be associated with optimal child growth. In addition, chronic illness, failure to adhere to MGRS feeding recommendations and maternal smoking were used as exclusion criteria.

By virtue of these characteristics, the MGRS provided the strong scientific foundations for developing standards that indicate how children should grow, as opposed to previous studies that simply described actual patterns of growth at a particular time and place. Consequently, the WHO Child Growth Standards (8) are now being used worldwide to judge children's growth because they demonstrate how healthy children grow in an environment which allows them to achieve their full growth potential.

The proposed study extends the MGRS into fetal and neonatal life. It will be based on the same prescriptive approach and international representation will be ensured by including populations from several countries. The design, implementation and conduct of the study, and dissemination of the results, as well as their incorporation into clinical practice guidelines and health care policy, will build on what has been achieved with the WHO Child Growth Standards, ensuring continuity between the development and implementation at country level of pre- and postnatal growth standards.

Conceptual Issues

There are important conceptual differences between growth references and standards. References, traditionally regarded as descriptive, are used for comparing different populations, while standards are prescriptive, implying a value judgment of optimal growth to be followed by individual pregnancies. A number of developed, as well as some developing countries have their own national standards for obstetric and neonatal use. In general, it is accepted that there is some variation in growth patterns among children of different racial or ethnic groups; however, these variations are relatively small compared to the large, worldwide variation in growth related to health, nutrition and socio-economic status. Therefore, the WHO Expert Committee on Physical Status (4) argued that "for this reason, a common reference has the advantage of uniform application allowing international comparisons without losing the usefulness for local application". Such advantages outweigh the disadvantage of not taking into account racial and ethnic variations, if actually observed. There are also practical reasons for not developing local growth standards for each country: 1) Many populations in less developed areas experience growth deficits as a result of poor health and nutrition, so that a local reference will have less screening value for the detection of health and growth disorders; 2) Significant secular changes in growth status within a relatively short period of time may render local standards less useful for clinical screening, and 3) Proper reference development is not a task that can be done easily or repeatedly, and it is very expensive to develop local standards.

In addition, as this is a longitudinal study, we will be able to study both fetal size over time (most commonly referred to as growth charts, i.e. centiles of *size* conditional on age) and actual fetal growth, expressed by the rate of growth between time periods (velocity charts).

For peer review only - http://bmjopen.bmj.com/site/about/guidelines.xhtml

Rationale for International Population Based Standards

We believe that the current use of multiple local charts presents sufficient problems to warrant preparing simple, international standards. However, measuring fetuses and newborns from a single, ethnically homogeneous community would be inappropriate, as it would fail to reflect any worldwide variation in growth. Using multiple populations from several countries would enhance the diversity in the biological characteristics - such as parental size and maternal weight gain during pregnancy - as well as the socio-cultural factors influencing fetal growth. This approach would also be politically more acceptable than constructing the standards based on data obtained from a single country.

Furthermore, there have been suggestions that the so called "customized" birth weight standards including ultrasound-derived curves of intrauterine weight (based on estimated fetal weight) are better predictors of perinatal mortality than population-based birth weight standards similar to those we plan to produce in the newborn component of the study. We do not agree with this concept as the maternal variables included in "customized" charts, such as ethnicity, parity and maternal weight are not physiological characteristics, they are intermediate variables. There is now strong evidence, using perinatal mortality as the substantive outcome, that the benefit in prediction is not a consequence of the "customization" by maternal characteristics but rather an effect of the higher birth weight distribution obtained from ultrasound derived fetal weight charts. Therefore, these adjusted charts will identify more preterm infants as growth restricted and therefore predict mortality better because of a higher cut-off and not because they fit fetal growth patterns better (9). The proposed population-based newborn standards from a well nourished, healthy population should produce results similar to those obtained by the "customized" charts, and in addition will be related to perinatal morbidity and mortality in the same study population.

Introducing the New Standards to Pregnancy and Newborn Care

Antenatal care consists mostly of a series of screening tests of varied complexity, implemented at different levels of care that, as a group, contribute to evaluating the overall status of each pregnancy. Objective assessments of fetal growth deviations can play a major role in prenatal care, neonatal care and outcome-based research. We are not proposing to recommend routine serial ultrasound examinations for all pregnant women in either developed or developing countries (see (10) and NICE Recommendations 2008); rather that, when they have to be used because of a clinical condition, they are interpreted correctly. Our study will produce three, new, scientifically valid tools for use at different levels of care to complement other tools already in use. The new standards will facilitate the correct interpretation of ultrasound scanning at levels of care where it is already widely used e.g. referral and most urban hospitals in developing countries. It is anticipated therefore that the new charts will have a major impact on overall care: for example, they are likely to result in fewer unnecessary interventions, such as Caesarean sections. The newborn standards will be used at all levels of delivery including rural areas; the preterm standards will be used in NICUs and similar levels up to primary health care. As an additional contribution to primary health care, we will produce new, multi-ethnic, uterine fundal height charts. We believe that such a comprehensive approach is the most effective format of care for pregnancy and newborns in developing countries.

Overall Study Strategy

Our overall strategy has been to adopt a very pragmatic approach to the study. In other words, we have tried to incorporate the study procedures into routine clinical care, so as to inconvenience the mothers as little as possible and avoid disrupting service delivery. An additional advantage of this strategy is that it increases the likelihood that the study's recommendations will be implemented and introduced into real life settings.

Nevertheless, during the preparation and implementation of the study, there will be many instances when a clear-cut answer to a clinical problem will not exist. We plan, therefore, to adopt a policy of asking the Principal Investigators and other members of the Steering Committee to make decisions in such cases. This policy will broaden our knowledge base and create a genuine feeling of team membership, which is so important in carrying out complex, multi-centre studies. The decision-making process will be supported by an

For peer review only - http://bmjopen.bmj.com/site/about/guidelines.xhtml

independent Advisory Group, which will be asked to resolve strategic conflicts. Ultimately, however, responsibility for finalizing the Study Protocol and the Operations Manual rests with the Executive Committee.

Selection of Study Sites and Population Framework:

There are two levels in the selection process of the study population: a) the cluster level, i.e. geographical region (e.g. city or part of a city) and within this geographical area, the selection of health institutions where women attend for antenatal and delivery care and b) the individual level, i.e. women or newborns that have the characteristics to be eligible for each of the sub- studies. We will discuss here the latter strategy, as it is common to all sub-studies and leave the former for each section on the specific sub-studies.

It is not necessary and would be unrealistic, especially in developing countries, for all institutions in a study area to fulfill all selection criteria. The basic characteristic for an institution to be considered is to serve a low risk population for impaired fetal growth. The concept is that such target populations, from which the individual participants will be drawn, should have no or be at very low risk of health, environmental or socio-economic constrains on fetal and newborn growth. Definitions will be locally adapted, for identifying socioeconomic characteristics associated with unconstrained growth in these populations. In principle, these will include markers of household income, housing tenure, education, occupation and employment status using locally selected cut-off points. These variables have been recently identified as explanatory factors in the relationship between birth weight and ethnicity (11).

Specifically, from each study site we will identify health institutions that serve populations with the above characteristics. We will conduct first a census of all hospitals where deliveries take place that are classified under local definitions as "private" or "corporation" hospitals or serving the upper socio-economic sector of the selected region. This is very important for developing countries sites. In developed countries, potential centres should also serve a general population, which will mostly have low-risk characteristics. We should concentrate on large institutions (>1000 deliveries a year), as it is important to involve only a small number of hospitals. Thus, from this census we will select those hospitals that cover at least 80% of all deliveries in the target population. However, it is recognized that some sites will not have large private hospitals and so local adaptations will have to be made.

The selected hospitals will need to agree to collaborate with the University of Oxford (Study Coordinating Unit) and provide evidence of being able to implement the study protocol; in particular, being able to coordinate referrals for ultrasound scans and having a policy of confirming gestational age by early ultrasound examination in all pregnant women. They will also need to show, using the latest data available, that the population they serve is actually of low risk as defined by: a) LBW rate <10% and mean birth weight >3100g; b) located at an altitude below 1,600m; c) perinatal mortality <20 per 1000 live births; d) mothers attending antenatal care in these institutions should plan to deliver in that or a similar hospital located in the same region; e) >75% of mothers have attained an educational level greater than the locally defined cut-off point; and f) lack of known non-microbiological contamination such as pollution, radiation or any other toxic substances (this will be evaluated in collaboration with the WHO Department of Protection of the Human Environment).

From this pool of eligible institutions that have the potential to provide the required sample size for ALL components of the study (target population), the Executive Committee and its advisors, in collaboration with the local study team, will identify the final list of units that will contribute to all the different sub-studies according to their capacity to provide the sample size needed, logistics, availability of equipment and population distribution. Individual participants will be selected from such a target population following the individual criteria listed below (see Section 1.1). Sampling for the fetal growth study will consist of all women that agree to participate and meet the selection criteria from the given date for initiation of the study until the sample is reached (500 pregnancies). For the preterm study we will take all preterm babies, with the inclusion criteria, from this cohort and for the newborn study we will take all newborns delivered at these institutions during the 12 month study period.

For peer review only - http://bmjopen.bmj.com/site/about/guidelines.xhtml

A. Objective I (Activities 1 – 3)

The primary objective of the international, multi-centre study will be achieved by three Activities:

1. Fetal Growth Longitudinal Study (FGLS) from <14^{+0} weeks gestation to birth, to develop the Fetal Growth Standards;
2. Preterm Postnatal Follow-up Study (PPFS) of preterm infants (>26^{+0} but <37^{+0} weeks) in the FGLS, to develop the Preterm Postnatal Growth Standards;
3. Newborn Cross-Sectional Study (NCSS) of all neonates born over 12 months in the participating centres including anthropometric measures and indicators of perinatal morbidity/mortality to develop the Newborn Birth Weight for Gestational Age Standards.

Both longitudinal and cross-sectional data will be collected at eight geographically diverse study sites, covering North and South America, Europe, Africa, Eastern, Western Asia and the Indian subcontinent, providing subpopulations from 5 major ethnic groups worldwide. The study sites have been selected from a pool of approximately 15 institutions already identified on the basis of a proven record of submitting high-quality data to clinical studies (10, 12-14). They also a) serve populations with a socio-economic status that does not restrict fetal growth (ensuring that the curves reflect the true growth potential of fetuses, without constraints related to maternal nutrition or any other social or environmental factors); b) are located at an altitude below 1,600m, and c) have a perinatal mortality rate <20/1000, a preterm delivery rate less than 15% of the target population.

1. FGLS: to develop the Fetal Growth Standards (Activity 1)

Limitations of existing intrauterine growth charts: There are three important issues relating to the construction of fetal growth standards that, in general, were not considered when the ones presently in use were produced (4). The issues are a) **longitudinal approach**: To create a standard that reflects patterns of healthy fetal growth, serial size measurements at different stages of pregnancy are required. This provides estimates of both the amount of variability in measures among fetuses in a population, as well as the amount of variability within individuals from their own average growth trajectory (15). Some early charts were built using a single measurement from each fetus based on the assumption that cross-sectional measurements from different fetuses can be used to estimate longitudinal patterns of growth. Other authors collected multiple measurements per fetus but analyzed the data in a cross-sectional manner, resulting in an underestimation of the true variance. The present study will collect and analyze data longitudinally. b) **Small sample size**: Most intrauterine standards are based on small sample sizes, which may lead to unstable estimates of percentiles, in particular at the extremes of the distribution. The extreme centiles are those that matter for the identification of growth deviations. c) **Lack of a prescriptive approach**: In the past, references have often been built based on existing practices or from specific research units' their final aim was to have population representation in particular place and time (population representativeness). This is a descriptive approach. We believe this strategy is incorrect as the underlying population often does not possess the best health and/or nutrition. For example, the observed secular increase in birth weight could reflect the tendency towards an increase in maternal body mass index (BMI) and obesity observed in most countries and not necessarily better health status of the pregnant population. Similarly, the very high Caesarean section rates in some populations (12) can affect birth weight patterns as reflected by the increase in preterm deliveries in these populations. Therefore, the proposed new standards will be built on the basis of recommended health practices (i.e. prescriptive approach): that is, defining the target population as well-nourished, healthy (both before and during pregnancy), non-smoking, no extreme ages, adequate education and socio-economic status. The sample to be used for the new standards should be representative of this "prescriptive" population, rather than the general population.

1.1 FGLS Individual Entry Criteria

Introduction: How to select a "healthy population" or its counterpart, the risk factors associated with fetal growth restriction, is open to considerable debate. The most important

For peer review only - http://bmjopen.bmj.com/site/about/guidelines.xhtml

factors to consider when selecting a healthy population with no obvious risk factors for IUGR or over-growth, at the first antenatal care visit early in pregnancy, are: a) achieving a balance between strict criteria for risk and external validity of the study population and b) the logistics of screening for factors that are not part of routine care or for which consensus is lacking about their effect on fetal growth.

We have reviewed the extensive literature on this topic which has tried to separate out the risk factors for preterm delivery and IUGR (16) particularly in low and middle income countries, as well as data from our own large-scale studies which have systematically explored the associations between risk factors and perinatal outcomes. In defining the eligibility criteria for the FGLS, one could exclude women with every possible risk factor for poor pregnancy outcome. However, we believe it would be preferable, in the initial screening process, to identify factors that are proven to be related to pregnancy outcomes, specifically those that are recommended as ways of identifying women who would benefit from low-risk antenatal care.

The approach is based upon our overview of the evidence of the effectiveness of antenatal care (17), which was recently up-dated (Abalos, *WHO* 2008). Similar patterns of care are recommended in the NICE/UK Clinical Guideline (2003) and by WHO for the antenatal booking visit. We will try to ensure that the approach is implemented in the participating centres in a standardized way.

All women attending the first antenatal care visit in the selected institutions with an initial evaluation of gestational age $<14^{+0}$ weeks will be screened at study entry based upon the criteria listed below. These women define a population that is likely to need only routine antenatal care in pregnancy, i.e. it is a group of clinically healthy women who can follow basic antenatal care models. Some variables have clear thresholds (e.g. urinary culture positive or negative); for others with less clear values (e.g. maternal height), we have selected a cut-off of 153 cm from the WHO Global Survey, a very large, global, multi-ethnic Maternal & Perinatal Health Study - for a detailed description of one of the 3 regions studied, see (18). This cut-off is supported by corresponding results from a number of other international studies (19, 20)

For peer review only - http://bmjopen.bmj.com/site/about/guidelines.xhtml

Individual Characteristics:

Recruited women must have the following characteristics at booking:

a) aged ≥18 and <35 years;
b) BMI ≥18.5 and <30 kg/m^2;
c) height ≥ 153 cm;
d) singleton pregnancy;
e) a known LMP with regular cycles (defined as 28 days ±4 days) without hormonal contraceptive use, or breastfeeding in the 2 months before pregnancy;
f) natural conception
g) no relevant past medical history (refer to screening form), with no need for long-term medication (including fertility treatment and over-the-counter medicines, but excluding routine iron, folate, calcium, iodine or multivitamin supplements);
h) no evidence of socio-economic constraints likely to impede fetal growth identified using local definitions of social risk;
i) no use of tobacco or recreational drugs such as cannabis in the 3 months before or after becoming pregnant;
j) no heavy alcohol use (defined as > 5 units (50ml pure alcohol) per week) since becoming pregnant;
k) no more than one miscarriage in the 2 previous consecutive pregnancies;
l) no previous baby delivered pre-term (<37 weeks) or with a birth weight <2500g or >4500g;
m) no previous neonatal or fetal death, previous baby with any congenital malformations, and no evidence in present pregnancy of congenital disease or fetal anomaly;
n) no previous pregnancy affected by pre-eclampsia/eclampsia, HELLP syndrome or a related pregnancy-associated condition;
o) no clinically significant atypical red cell alloantibodies;
p) negative urinalysis;
q) systolic blood pressure <140 mmHg and diastolic blood pressure < 90 mmHg;
r) no diagnosis or treatment for anaemia during this pregnancy (Hb levels wil be monitored throughout pregnancy)
s) no clinical evidence of any other sexually transmitted diseases, including syphilis and clinical Trichomoniasis;
t) not in an occupation with risk of exposure to chemicals or toxic substances, or very physically demanding activity to be evaluated by local standards. Also women should not be conducting vigorous or contact sports, as well as scuba diving or similar activities

Estimation of gestational age at study entry: Clearly, establishing a very precise determination of gestational age is vitally important for constructing these growth standards (in addition to clinical management) and we are prepared to screen large numbers of women, if necessary, to obtain the ideal population.

There are three possibilities for gestational age estimation early in pregnancy: dating by a) LMP alone; b) early ultrasound alone or c) LMP and ultrasound. The implications of these different methods on research findings have recently been discussed (22). Dating by LMP and ultrasound is clearly an assessment of different parameters. Although it has been suggested that before 14^{+0} weeks of gestation ultrasound may be better by an average of 2-3 days in predicting the date of delivery, in clinical practice, both are often used in combination. Based on the 2004 Birth Cohort from the Brazilian Centre, we anticipate that 7% of women will not have a reliable LMP. In other studies in this socio-economic group, the figure may be as high as 20%.

For peer review only - http://bmjopen.bmj.com/site/about/guidelines.xhtml

Taking all these factors into consideration, we have decided not to base gestational age solely on LMP; rather we shall use a standard gynecological definition of LMP as the first level in calculating the gestational age. Among women with a certain LMP and regular periods who are 9^{+0} to 14^{+0} weeks pregnant, we will corroborate the gestational age with an ultrasound CRL measurement, using an internationally recognized and validated chart (23). If the difference in gestational age estimation by CRL and LMP is ≤ 7 days we will consider the LMP to be reliable and take it as the true biological date. It is acknowledged that we may exclude a number of women who might otherwise have been eligible if only ultrasound were used, but the aim is to define a population with as exact a gestational age at entry as possible.

1.2 Nutritional adequacy during pregnancy

In the WHO Child Growth Study, it was assumed from the outset that children recruited from affluent populations consumed adequate complementary foods. This proved to be the case when we analyzed their complementary diets (24). In the present study, we shall adopt the same concept but in addition will develop general nutritional guidelines for pregnant/lactating women, suitable for local use, based on the best available evidence, e.g. NICE recommended, UK Food Standards Agency advice http://www.eatwell.gov.uk/agesand Stages/pregnancy/whenyrpregnanct/ for promotion amongst the participating mothers and care providers.

Routine nutritional supplements, e.g. protein or energy, will not be given because a) it is not a component of the recommended antenatal care package (10) and b) we are only planning to recruit women with adequate nutritional status. Iron-folate supplementation, will be prescribed if necessary for anemia during/after pregnancy but given routinely only if such a policy is in place in the institution. A similar position will be taken with calcium supplementation for the prevention of pre-eclampsia and preterm delivery if such a policy is in place in the hospital.

Women will be asked to indicate if they are taking nutritional supplements and this information will be recorded in the data collection form. It is not practical in a study of this size to measure adherence in any other way or to obtain individual intakes, e.g. 24 hr recall, considering the poor reliability of such instruments for individual assessment.

1.3 Pregnancy follow-up

Women in FGLS will receive standardized antenatal care (with some local variations) based on the recommended WHO package, part of which involves screening for conditions that emerge during pregnancy.

All women recruited will be followed throughout pregnancy from the time of the first visit, irrespective of the pregnancy outcome. As a general principle, the number of exclusions will be as small as possible. They are likely to be confined to fetuses with congenital abnormalities (based on a final evaluation at birth); multiple pregnancies that were not identified at recruitment; mothers diagnosed with catastrophic or very severe medical conditions which were not present at recruitment (e.g. cancer, HIV), and those with pregnancy related conditions requiring hospital admission (e.g. eclampsia or severe pre-eclampsia). Hospital admission *per se* is not a reason for exclusion: women admitted simply for “observation” will still contribute data to the fetal growth standards unless they develop one of the conditions listed above. Most sites are malaria free ecozones; thus, intermittent preventive treatment is not routinely recommended although insecticide-treated mosquito nets may be used. In other potential sites malaria is prevalent. We will adhere to local protocols and exclude from incorporation in the fetal growth standards any woman with evidence of malaria infection during the pregnancy.

For the sake of clarity, we will produce during the preparatory phase of the study, in collaboration with the Steering Committee and experts in the field, a very detailed list of those conditions, which would result in a pregnancy being excluded from the preparation of the fetal growth standards. In any case, if any exclusion is needed it will be done only during the data analysis period and local investigators will complete the follow-up of all enrolled women irrespective or any follow-up experience. Data from preterm deliveries, even in the absence of one of the exclusion criteria, will not contribute to the fetal growth standards.

For peer review only - http://bmjopen.bmj.com/site/about/guidelines.xhtml

We also expect that INTERGROWTH-21st will have fewer follow-up problems than randomized trials as it does not involve an intervention; a well-educated population will be recruited, and women and their families will like some components of the study such as ultrasound pictures or close monitoring of preterm infants. Nevertheless, to ensure that the loss to follow-up remains below our predicted <5% level, we will maintain very close contact with participants; remind them about imminent visits and ultimately conduct home visits if necessary.

1.4 FGLS 2D Ultrasound Measurements

Justification: In preparing the protocol it became clear that more than 6 ultrasound examinations after the dating scan would present unwanted logistical problems and inconvenience to mothers. It was also evident that a minimum growth change has to occur between visits, to be reliably measured by ultrasound, considering the errors in the equipment and observers. Thus, even if velocity growth by unit of time (e.g. one week) for certain parameters is high during some gestational periods, the actual change may not be reliably measured. We have considered alternative spacing between measurements such as longer intervals early in pregnancy (e.g. 8 weeks) and shorter ones in later pregnancy (e.g. 4 weeks). There is evidence, however, that measures such as BPD and femur length continue to increase fairly linearly until 34 weeks without a clear period of very high velocity growth. The logistics of coordinating multiple antenatal visits at different time intervals in a large sample mitigate against adopting variable timings. Finally, as women will be recruited more or less randomly at gestational ages between 9^{+0} to 14^{+0} weeks, we will have a spread of visits throughout pregnancy, which is also advantageous in logistical terms. In a previous report, for example the Guatemalan study, 21% of participants had one scan, 50% had two scans, 24% had 3 scans, and 5% had 4 or 5 scans at varying gestational ages (25). In the Western Australia study, participants were recruited at 16-18 weeks and scanned again at approximately 24, 28, 34 and 38 weeks (26) which we believe is less satisfactory than our schedule. We have explored further this question in a large Scandinavian data set. For 5 measures, in a fixed interval of 7 weeks between measures, they did not detect any problems in modeling growth. Therefore, after the first scan between 9^{+0} to 14^{+0} weeks, we will perform scans at ~5 weekly (±1 week) intervals, i.e. 14-18, 19-23, 24-28, 29-33, 34-38 and 39-42 weeks. Thus, a woman could have scans at 10, 15, 20, 25, 30, 35 and 40 weeks or 11, 16, 21, 26, 31, 36 and 41 weeks depending upon the gestational age at recruitment and duration of pregnancy. This has the advantage of providing greater coverage of the complete gestational period and easier scheduling of the scans.

Schedule: After the dating scan, 6 further visits (for fetal biometry) will be scheduled at ~5 weekly (± 1 week) intervals (i.e. 14-18, 19-23, 24-28, 29-33, 34-38 and 39-42 weeks). Seven measurements will be taken at each visit from 14 weeks onwards: Biparietal Diameter (BPD); Occipito-Frontal Diameter (OFD); Head Circumference (HC); Transverse abdominal diameter (TAD); Anterio-posterior abdominal diameter (APAD); Abdominal Circumference (AC) and Femur Length (FL)

At each visit, the measurements will be obtained 3 times from 3 separately generated ultrasound images in a "blinded" fashion, and submitted electronically (with the associated images) to the Coordinating Unit. The BPD, OFD, HC, TAD, APAD, AC and FL images should fill at least 30% of the monitor screen. The last recorded value of each fetal measurement will be revealed after submission for clinical purposes, as per local protocols. Standardized images will be obtained for all measurements based on internationally accepted protocols (27).

For example, the BPD will be measured from the outer-outer edges of the parietal bones in a cross-sectional view of the fetal head at the level of the ventricles. The measurement should be obtained from an image with the midline echo as close as possible to the horizontal plane with the angle of insonation of the ultrasound beam at 90°. The image should be oval containing a centrally positioned midline echo interrupted in its anterior third by the cavum septum pellucidum. The anterior walls of the lateral ventricles should be centrally placed around the midline echo and the choroid should be visible in the posterior horn in the distal hemisphere.

For peer review only - http://bmjopen.bmj.com/site/about/guidelines.xhtml

The CRL to gestational age conversion will be performed using the charts developed by Robinson and Fleming (23), which remain the most widely accepted reference for early gestational age estimation. This chart is, in any case, based in a cohort of women with known gestational estimation by LMP. This is a circular argument that limits the use of ultrasound alone for the estimation of gestational age. We are aware that an ultrasound expert group in the UK is reviewing the use of these charts. We are, therefore, prepared to consider changing to a new reference if one becomes available.

We also prefer not to include participants in whom fetal size is discrepant from LMP. If we use ultrasound alone to estimate gestational age and the measurement is erroneous, that error becomes built into the growth chart. This seems another circular argument that we must try to avoid. Furthermore, the ultrasound estimation of gestational age has the limitation that all fetuses with a given CRL value will have the same gestational age estimation. This lack of biological variability is a major limitation for evaluating growth. A very interesting discussion on this point can be seen in (28).

In short, we will only consider for recruitment women with a certain LMP and regular periods; from these, we will only select those women with a CRL that is equivalent to their LMP, based on the Robinson and Fleming charts. This population is at the lowest risk of negative pregnancy outcomes (29).

Additional measurements: There is a unique opportunity to complement the study by taking additional measurements, e.g. BPD using the scanner's ellipse facility; trans-cerebellar diameter; humerus, radius/ulna, tibia/fibula and fetal foot length, and Doppler measurements of the umbilical cord artery, middle cerebral artery and maternal uterine artery. However, we are concerned that introducing extra tasks could reduce the overall quality of the seven main measurements and inconvenience the participants. Therefore, the feasibility of taking additional measurements will be evaluated in consultation with each centre before a final decision is made. In general, only some centres are expected to participate in these additional studies, which are mostly descriptive and exploratory with smaller sample sizes than in the main study.

Variability of measurements: We intend to explore several factors that might explain variability, e.g. parity, BMI, gestational age, position/presentation of the fetus, liquor volume, gender and number of measurements. We shall also introduce a comprehensive system for evaluating intra- and inter-observer variability using the 2D and 3D measures, as well as a random sample of retaken measures by an external ultrasonographer in each centre.

Equipment: All the study centres will use equipment with the same characteristics (determined by an independent group), including a state-of-the-art scanner. The staff will be appropriately trained following standardized procedures. The study will provide the equipment free to all the centres in developing countries; centres in developed countries will be expected to provide equivalent equipment themselves, as their contribution to the study. It is very important that ultrasound probes provide an extremely high quality grey scale image. We like to stress that the results of the study will be in the public domain and all manufacturers will be able to use the results free of charge. A detailed description of the methodology for ultrasound measures is presented in Appendix C.

For peer review only - http://bmjopen.bmj.com/site/about/guidelines.xhtml

1.5 FGLS Quality Control Measures

Quality control measures will include a unique system of random evaluation and repetition of ultrasound measurements and continuous real time assessment of all data collected. A limited number of experienced obstetric ultrasonographers and neonatal anthropometrists will form the research teams at each study site. They will be provided with standardized equipment and an operations manual that will describe all measurement techniques, protocols and procedures for training and clinical use in all the centres. The ultrasonographers will receive specific training under the supervision of qualified instructors. Intra-observer and inter-observer measurement errors will be assessed during the training course before initiating the study.

Both the examiner and the instructor will scan each fetus briefly. Each examiner will obtain two images of each fetal anatomical parameter under study. Differences between these measurements will be expressed as the percentage of the measurement obtained from the technically better image. Percentage differences will be used to take into account the increase in the dimensions of the fetal anatomical parameters with advancing gestational age. Percentage differences for each examiner will be averaged and the mean values compared to zero and to the instructor's measurements by t-test. In addition, measurement error will be evaluated by defining a maximum acceptable discrepancy, e.g. 5%, and requiring that 95% of differences lie within that value. This will provide information on the accuracy of individual measurements. The same model of ultrasound machine will be used at all sites, and the equipment will be serviced periodically by specialized technicians as per the manufacturer's recommendations.

1.6 FGLS 3-Dimensional Ultrasound Volumes

3-Dimensional (3D) ultrasound technology, a relatively new scanning technique in obstetrics, offers exciting possibilities to assess the fetus. The technique is based upon volume acquisition using specially designed probes with later, off-line review and "3D reconstruction" on a computer. This allows examination of multiple planes of the imaged structure from 3 different right angles (top-to-bottom; front-to-back and side-to-side). Additionally, a variety of techniques for measuring fetal organ and limb volumes have been described. The multiplanar display allows a clearer and more comprehensive assessment of fetal anatomy, and volume calculation produces more accurate estimates of fetal size and the impact of growth restriction on specific fetal organs (30).

The primary purpose of the 3D component in this study is quality control, i.e. to provide volumetric data to assess the quality of the 2D measures (BPD, OFD, HC, TAD, APAD, AC and FL). Hence, the 3D volumes will be the head (at the level of the BPD), the abdomen (at the level of the AC) and the femur. The task of controlling quality with these data will carried out by an independent group. The technique of 3D volume acquisition is similar for all structures. Initially, the structure to be imaged is identified during the last of the three 2D scans taken. Once imaged correctly, the volume acquisition facility is engaged and the volume obtained over a brief (10-15 seconds) interval and stored in digital form on computer for later analysis, which will be performed in Oxford by the Noble research group after the stored images have been transferred there electronically.

1.7 Criteria for excluding participants from preparation of fetal growth standards

Women in FGLS will receive standardized antenatal care (with some local variations) based on the recommended packages, part of which involves screening for conditions that emerge during pregnancy and is presently used in the hospitals. All women recruited will be followed throughout pregnancy from the time of the first visit irrespective of the pregnancy outcome, except for those with a late diagnosis of multiple pregnancy. However, we do not expect to include all pregnancies in the preparation of the fetal growth standards as some of them may develop conditions that can affect fetal growth. As a general principle, the number of exclusions will be as small as possible. They are likely to be confined to fetuses with congenital abnormalities (based on a final evaluation at birth); severe IUGR requiring early delivery; multiple pregnancies that were not identified at recruitment; mothers diagnosed with catastrophic or very severe medical conditions which were not present at recruitment (e.g. cancer, HIV), those with pregnancy related conditions requiring hospital admission (e.g. eclampsia, placental abruption or severe pre-eclampsia) or those with acute conditions such

For peer review only - http://bmjopen.bmj.com/site/about/guidelines.xhtml

as an episode of malaria infection. Hospital admission *per se* is not a reason for exclusion: women admitted simply for observation will still contribute data to the fetal growth standards unless they develop one of the conditions listed above.

For the sake of clarity, we will produce during the preparatory phase of the study, in collaboration with the Steering Committee and experts in the field, a very detailed list of those conditions, which would result in a pregnancy being excluded from the preparation of the fetal growth standards. Data from preterm deliveries, in the absence of one of the exclusion criteria, will contribute to the fetal growth standards until the time of delivery.

1.8 Symphyseal-fundal height charts

We will produce, in addition to the ultrasound charts, new data on symphyseal-fundal height measurements to update the charts we first produced 30 years ago based on a single sample (31). To produce one, standardized, well-validated, multi-ethnic chart using this selected population to replace several used in primary health care units and hospitals around the world would be extremely useful. We expect that these measures be taken at the same time that the ultrasound 2D measures following the same schedule by specifically trained staff.

1.9 PEA POD measurements

For the Oxford site only, we will include an additional anthropometric measurement of body composition from FGLS pregnancies. Body weight alone can be very misleading; the weight scale cannot tell the difference between an ounce of fat and an ounce of muscle. Body composition estimation provides a measure of the amount of fat mass and the fat-free mass in the body and is now recognized as the most accurate method of measuring infant growth and nutritional status. It is now widely recognised that the accurate assessment and tracking of body composition in the critical period immediately following birth can provide key information in both clinical and research settings. Body composition information can be used to help monitor and evaluate infant growth patterns, optimise nutritional interventions, obtain important feedback during drug treatments, and optimise discharge criteria. Previously, obtaining reliable infant body composition data has been difficult, with available methods limited by problems with accuracy, practicality, invasiveness, and safety. Air Displacement Plethysmography has solved all of these issues by offering accurate and precise measurements of infant body fat and fat-free mass quickly, safely, and comfortably. This technique has now become a vital tool in evaluating infant growth and nutritional status and it has been widely applied in a large number of studies of newborns and infants throughout the world as it is quick and entirely safe.

Over many thousands of applications in infants the use of the PEA POD has not proven to be distressing at all to infants or their parents. In the unlikely event that a child were to be distressed after being placed in the PEA POD (or if for any reason a parent became distressed) then the procedure would be immediately terminated. A further attempt would be made only when appropriate i.e. the child was calm and with consent from the parent.

We will use the PEA POD, which is very similar to a standard neonatal incubator for determining percent fat and fat-free mass in infants. The simple, 7-minute test consists of measuring the subject's mass (weight) using a very precise electronic scale, and volume, which is determined by air displacement plethysmography while the infant lies inside the PEA POD chamber. From these two measurements, the infant’s body composition is calculated. For the PEA POD manual of operations see Appendix J.

1.10 1 and 2 year follow-up

All infants born to mothers in FGLS will be followed up at the ages of 1 and 2 (± 2 weeks) to evaluate their postnatal growth. The same methods used to measure the head circumference, weight and length at birth will be employed in the follow-up of infants in this cohort. These will allow us to monitor the growth and health of all infants for whom we have accurate, ultrasound-derived, intrauterine growth patterns and detailed information about pregnancy and perinatal events.

Four additional, non-invasive and painless measures of development will also be taken at the 2 year follow-up using standard protocols as defined by the authors of the respective tests: 1) Assessments of motor skills, cognition, speech and behaviour using a tool

For peer review only - http://bmjopen.bmj.com/site/about/guidelines.xhtml

consisting of the Rapid Neurodevelopmental Assessment of Children (32) and components from the Malawi Development Assessment Tool and the Bayley Scales of Infant Development; 2) Visual acuity and contrast sensitivity measurements using the Cardiff Tests (33); 3) Cortical auditory processing assessments to an auditory novelty, odd-ball paradigm using wireless and gel-free electroencephalography (34), and 4) sleep-wake measures using actigraphy (35).

These measurements will allow us to monitor the growth, health and development of all infants for whom we have detailed ultrasound-derived, intrauterine growth patterns and detailed information about pregnancy and perinatal events.

The additional developmental measurements at the 2 year follow-up will be as follows:

**Functional Domain**	**Functional construct assessed**	**Measure**
Development	Motor skills, cognition, speech and behaviour	Modification of the Rapid Neurodevelopmental Assessment of Children
Vision	Visual acuity and contrast sensitivity	Cardiff tests
Hearing	Auditory processing to a novelty, odd-ball paradigm	Cortical evoked response potentials using electroencephalography
Sleep	Sleep efficiency	Actigraphy

At the 1 and 2 year follow-up appointments of the infants, we will measure maternal weight. We will contact women and their GPs by letter about these appointments and invite them to attend. If they are unable to attend, we will ask them if we can instead arrange a home visit, a telephone interview or if they can complete the questionnaire by post or email.

1.11 Biological samples

Oxford FGLS extension (INTERBIO-21st Fetal & Infant Growth Study)

From 2011 to 2014, we will collect and store maternal blood (12ml) at booking, a urine sample (5ml) at each follow-up ultrasound appointment, as well as maternal faeces (5ml, only if the mother has opened her bowels) and cord blood (12ml)/placental (9x 5mg) samples at delivery (in addition to the pregnancy and fetal growth data) from a total of 1,000 pregnancies. The samples will be used for nutritional and epigenetic studies into fetal growth. The INTERBIO-21st Fetal & Infant Growth Study will continue to monitor fetal growth and the growth and development of the infants using the same protocols as the FGLS component of INTERGROWTH-21st http://www.medscinet.net/intergrowth/protocol.aspx http://www.medscinet.net/Interbio/protocol.aspx?lang=1 (See Appendix K).

Oxford NCSS extension (INTERBIO-21st Neonatal & Infant Growth Study)

From 2011 to 2014, we will collect and store maternal blood (12ml) at delivery, as well as maternal faeces (5ml, only if the mother has opened her bowels) and cord blood (12ml)/placental (9x 5mg) samples at delivery (in addition to the pregnancy data) from a total of 10,000 pregnancies. The INTERBIO-21st Neonatal & Infant Growth Study will continue to monitor the growth and development of the infants using the same protocols as the NCSS component of INTERGROWTH-21st http://www.medscinet.net/intergrowth/protocol.aspx. http://www.medscinet.net/Interbio/protocol.aspx?lang=1 (See Appendix K).

Epigenetic studies in INTERGROWTH-21st participants

Use of Guthrie cards: We will perform the same analyses (i.e. nutritional and epigenetic) on DNA extracted from routinely collected blood spots (stored as Guthrie cards and no longer required for clinical purposes) obtained from the cohort of 5,941 babies born at the John Radcliffe Hospital who participated in the original INTERGROWTH-21st Study. The Guthrie cards will be barcoded, linked anonymously to the clinical data collected from the INTERGROWTH-21st participants and then unlinked.

For peer review only - http://bmjopen.bmj.com/site/about/guidelines.xhtml

Discarded 1st trimester Down's screening samples

From 2011 to 2014, we will collect and store all discarded 1st trimester samples that were used to screen Oxford patients for Down's Syndrome (approximately 7,000/year). The results of the analysis of these samples will be linked to INTERBIO-21st Fetal and Neonatal & Infant Growth Study data, as well as anonymised, routinely collected pregnancy outcome data from all other women. As serum samples from other hospital trusts are also routinely sent to the John Radcliffe Hospital for Down's screening, we will: a) store these samples; b) link them to anonymised, routinely collected pregnancy outcome data from all women delivering at the Royal Berkshire Hospital, Reading; Milton Keynes Hospital, and Buckinghamshire Healthcare NHS Trust (approximately 21,000/year), and c) similarly use them in future studies to test novel biomarkers predictive of adverse pregnancy outcomes.

Sample use

All the samples described above will be used for a range of biochemical, nutritional, genetic and epigenetic studies into placental function and fetal growth. Any unused samples in the future may be donated to the Oxford Radcliffe Biobank for studies into the causes of pregnancy-related problems http://wyvern.ndcls.ox.ac.uk/orb/.

1.11 The Pregnancy Physiology Pattern Prediction (4P) Study

An urgent need to develop an evidence-based, national, Modified Obstetric Early Warning Score (MEOWS) was highlighted in the two most recent Confidential Enquiries into Maternal Deaths in the UK. An essential prerequisite to developing such an early warning system is knowledge of the normal distributions of physiological data (blood pressure, temperature, respiratory rate, heart rate and oxygen saturation) in "low-risk" pregnant women. The 4P Study aims to obtain these longitudinal data, from <14 weeks' gestation to 2 weeks after delivery, from women participating in the Oxford arm of the INTERBIO-21st Fetal & Infant Growth Study. For details of observation measurement in the 4P Study, see Appendix I.

Outcomes

- A national early warning system will be developed for all gestational ages and the immediate post-partum period.
- An NHS web-based programme will be developed, allowing age, parity and gestational-age specific MEOWS chart to be created for individual mothers on admission to hospital.
- An automated, multi-parameter, age, parity and gestational-age specific alerting system will be created for use in monitored areas and hospitals with an EPR system.
- Fetal size will be correlated with maternal physiological values.

2. PPFS: to develop the Preterm Postnatal Growth Standards (Activity 2)

2.1 Follow-up of preterm babies in FGLS and INTERBIO-21st Fetal & Infant Growth Study

All preterm newborns (>26^{+0}<37^{+0} weeks) from the FGLS and INTERBIO-21st Fetal & Infant Growth Study cohorts will be followed-up for 8 months after delivery and at 15, 18 and 21 months to evaluate postnatal growth. The same anthropometric measurement techniques to measure the head circumference, weight and length will be used to follow-up infants in these cohorts. At 15, 18 and 21 months, we will use the same data collection forms that are used to follow-up these infants at 1 year. These measurements will allow us to monitor the growth and health of all infants for whom we have detailed ultrasound-derived, intrauterine growth patterns and detailed information about pregnancy and perinatal events.

In addition, we will assess the motor development of the preterm cohort every month from 4 months of age to 8 months of age using the motor development assessment questionnaire, which is part of the 1 year assessment. This consists of two parts: 1) a questionnaire for the parents about the age their child achieved some gross motor milestones, and 2) a questionnaire completed by the Measuring Team reporting whether or not the child demonstrated those milestones during the visit.

At 2 years of age, the same anthropometric measurements (head circumference, weight and length) will be taken. In addition, we will also measure developmental outcomes in the

For peer review only - http://bmjopen.bmj.com/site/about/guidelines.xhtml

children. These measurements will include a test of hearing (cortical auditory processing); a test of visual acuity and contrast sensitivity; an assessment of the child's cognitive, language, motor and behavioural development, and an assessment of sleep. Section 1.9 details the techniques used to measure these developmental outcomes.

These measurements will allow us to monitor the growth, health and development of all infants for whom we have detailed ultrasound-derived, intrauterine growth patterns and detailed information about pregnancy and perinatal events.

We plan not to include preterm newborns ≤26 weeks (only ~3% of all preterm newborns) because of the very high morbidity/mortality in this group and need for very special intensive care. A fixed follow-up period was chosen to simplify organization of the study and reduce loss to follow-up. Nevertheless, an analysis based on the time from conception (corrected age) will be performed to compare preterm babies with their *in utero* counterparts conceived around the same time. This length of follow-up should minimize a) inconvenience to the mothers; b) the need for home visits, and c) the study's cost and complexity. An analysis based on the time from conception (corrected age) will be performed to compare preterm babies with their *in utero* counterparts conceived around the same time. Although we want to produce growth charts for the first 6 months of life, we have extended the follow-up period to 8 months for these infants to avoid the so-called "right-edge" effect in the construction of the growth standards.

Exploratory sub-group analyses will also be conducted, e.g. very preterm infants <32 weeks (~20% of all preterm newborns) and induced for medical reasons vs. spontaneous preterm deliveries. However, to be included in the PPFS growth charts, newborns have to meet the criteria decided a priori. A detailed protocol describing clinical conditions for inclusion in the final growth curves has been prepared in consultation with expert neonatologists before any data analysis is undertaken.

This protocol will focus on definitions that can be made operational across institutions. Newborns will be excluded retrospectively if they have severe medical morbidities likely to impact upon growth and congenital malformations. (See also section 2.4).

Clearly, for some subgroups of very preterm infants this will represent only an exploratory analysis; however, it will still be done because of the unique opportunity to study continuous fetal-preterm growth. It is expected that many babies, especially those born <32 weeks, will be managed in some form of neonatal intensive care unit. Measurements of these babies will be taken using the study protocol, but in accordance with the clinical status of the infant and the unit's protocols. It is clear that to ensure that "clinically stable" babies are comparable across different NICUs, standardization and continuous monitoring are needed. Ultimately, in such an acute clinical care setting, the final judgment will depend on the attending doctor; realistically, this is how the standards will be used in practice anyway.

2.2 PPFS Anthropometric Measurements

The anthropometric measurements will include weight, length and head circumference. The three measurements (and a standard clinical evaluation) will be taken every 2 weeks during the first 8 weeks, and then every 4 weeks until 8 postnatal months, using essentially the same methodology and operating manual employed in the WHO Child Growth Study (6). The only difference will be that in the proposed study all measures, interviews and clinical evaluations will be conducted at a special follow-up clinic in the corresponding hospital. Routine home visits are not planned, except for those mothers who do not comply with the protocol's scheduled visits. It is expected that the preterm babies will have a maximum of 11 follow-up visits over 8 months, but this number may be reduced for some as complications, including death, are expected especially in the very preterm subgroup. Abdominal circumference will not be included as it is not used in routine neonatal practice and respiratory movements in these tiny newborns make the measurements unreliable.

Standardized, electronic, digital, newborn weighing scales will be used and serviced regularly; they will be replaced if they are faulty and cannot be repaired. All anthropometrists in the study will be trained centrally; they in turn will train the nurses/midwives in how to use the scales. We will follow the same procedures used in MGRS to certify all staff responsible for taking the measurements, as was the case in our own previous randomized trials. During routine measurement sessions, two anthropometrists will independently take duplicate

For peer review only - http://bmjopen.bmj.com/site/about/guidelines.xhtml

measurements. Maximum allowable differences between duplicate measurement values will be checked to detect outliers and trigger immediate re-measurement if necessary.

2.3 PPFS Quality Control Measures

An Anthropometric Data Quality Unit, organized on similar lines to the Ultrasound Quality Control Unit, will be created. Its role will be to conduct, following the WHO MGRS's training procedures, the standardization, monitoring, site visits, and retraining when necessary of all staff responsible for anthropometric measurements (MGRS Manual of Operation, WHO Geneva). Using the on-line data management system, samples of measurements will be obtained to evaluate variability and consistency within each site and across sites. The same protocol will be followed for measures at birth for all newborns in the fetal and newborn studies. For the preterm follow-up we will use the MGRS protocol but include only weight, length and head circumference. We will assign 2 staff at each centre to take all newborn (and postnatal follow-up) anthropometric measurements. This will permit replication of the WHO MGRS measurement standardization protocol (6).

As stated above, the standardization of all anthropometric measurements is a central element and challenge of this protocol. The measurers (or lead measurer from each site) will be brought together at a central location for two initial training meetings to be conducted by experienced staff from the WHO-MGRS team with the assistance of the MGRS anthropometry video. This video will be used for local training as well as for on-going standardization activities. The study will provide all sites with a new infantometer(s) with digital reading for infant length measures. For birth weight, hospitals will use the balances they are currently using but they will be calibrated daily. Other equipment will be calibrated at least once a week during data collection. The overall sample will be also be monitored daily, using the on-line data entry system, for unexpected values and outliers so that measurements can then be repeated within a few days, if appropriate.

2.4 Infant Morbidity During Follow-Up

For the Preterm Follow-up Study, we will use an indicator of morbidity at entry and an extended version for the follow-up itself. The protocol states (see section 2.1) that the study will exclude newborns ≤26^{+0} weeks (only ~3% of all preterm newborns) because of the very high morbidity/mortality in this group and need for very special intensive care. A detailed protocol describing clinical conditions, "minimum criteria", for inclusion in the final growth curves will be prepared a priori before any data analysis is undertaken. Babies that do not meet the “minimum criteria” will nevertheless be followed up to form a subgroup for comparative analysis with the “healthy” preterm infants. Efforts will therefore be made to standardize the major components of infant care throughout the participating institutions. The protocol will focus on definitions that can be made operational across institutions. Criteria are likely to include any morbidity severe enough to have a negative impact on growth, feeding or the feasibility of measuring the infants at the required times.

2.5 Infant Feeding Practices

We expect newborn feeding to be protocol driven and based on a) the recent WHO recommendations (Edmond and Bahl "Optimal feeding of low-birth-weight infants" WHO, 2006) and b) standard NICU practice for clinical status. Descriptive data, collected in an adapted version of the WHO MGRS form, will serve to document that the centres conformed to recommended feeding patterns and clinical practice. The comparison with intrauterine charts is an important, biological secondary objective, but the main objective of the PPFS is to describe the postpartum growth that can be expected, or aspired, to by "healthy preterm newborns".

It is anticipated that all the sites will adhere to internationally recommended standards for feeding preterm infants; see review (36). The recommended first choice for LBW infants is breastfeeding or mother's own expressed milk. This is associated with the lowest incidence of infection and necrotizing enterocolitis, and best outcomes in terms of neurodevelopment. Where that is not possible, the next best choice is donor human milk and, in third place, preterm infant formula (Edmond and Bahl "Optimal feeding of low-birth-weight infants" WHO, 2006).

For peer review only - http://bmjopen.bmj.com/site/about/guidelines.xhtml

3 NCSS: to develop Newborn Birth Weight for Gestational Age Standards (Activity 3)

3.1 NCSS Individual Entry Criteria

The Newborn Cross-Sectional Study will include a) all babies born to mothers in FGLS and b) all other babies born at the study centres during a fixed 12 month period. The vast majority of babies will have had their gestational age confirmed by an early ultrasound because all the hospitals included in INTERGROWTH-21st sites should already have a policy of checking gestational age with an early dating scan. Measures will be taken during the study period to reinforce this policy to ensure that all newborns have their gestational age at birth corroborated by ultrasound scan estimation early in pregnancy. The strategy of including the complete newborn population will allow us to study a large number of LBW infants and subgroups under a wide range of conditions that can affect these outcomes. We will also be able to compare the total populations across centres in terms of socio-demographics and pregnancy outcomes to confirm that the populations contributing to the new standards have the required characteristics.

3.1.2 Constructing the Standards

For construction of the birth weight for gestational age standards, babies born to mothers without health problems likely to influence fetal growth will be selected from the total number of births at each centre. However, the total population of newborns during the same study period will be used to explore Objective III (determinants of LBW and its components, preterm delivery and impaired fetal growth, under current healthcare conditions). In addition, we plan to use an outcome-based approach, based on recent recommendations.[a]

3.2 NCSS Anthropometric Measurements

All babies born during the study period will have these anthropometric measures taken within 24 hours of delivery: weight, length and head circumference. To select those newborns eligible for the NCSS (newborn standards), we will use the same criteria as for the fetal growth study.

Standardized, electronic, digital, newborn weighing scales with a precision of 10 grams will be used and their calibration status will be checked daily; they will be replaced if they are faulty and cannot be repaired. We shall also provide all clinics with standardized infantometers for length (precision 0.1 cm) and tape measures for head circumference (precision 0.1 cm); these will be similarly calibrated and maintained. All anthropometrists will be trained centrally and monitored during the study following standard procedures by the Anthropometric Standardization Unit; they in turn will train the nurses/midwives in how to apply the study's measurement protocol. We will follow the same procedures used in MGRS to certify before and during the study all staff responsible for taking the measurements, as was the case in our own previous randomized trials. As in MGRS, routine data collection will comprise duplicate measurements of weight, length and head circumference taken independently by two anthropometrists. They will check maximum allowable differences between their replicate measurement values immediately the measurements. These maximum allowable differences are approximately twice the Technical Error of Measurement of a well-trained observer (e.g. 0.7 cm for length and 0.5 cm for head circumference). If the maximum allowable difference is exceeded, the anthropometrists will immediately go on taking other duplicate measurements until the difference is acceptable. Replicate measurement values will be averaged before analysis to increase accuracy further.

3.3 NCSS Quality Control Measures

The same quality control measures and standardization strategy (adapted from the WHO Child Growth Study protocol) (7) which are described above for PPFS will be employed for the three measurements to be taken for the whole study population in NCSS. A special Anthropometric Data Quality Control Unit, independent of the Study Coordinating Unit, will

---

[a] Joseph KS, Fahey J, Platt R et al. (2009) An outcome based approach for the creation of fetal growth standards: do singletons and twins need separate standards? Am J Epidemiol 169:5:616-624

For peer review only - http://bmjopen.bmj.com/site/about/guidelines.xhtml

be responsible for the monitoring and quality control of these measurements during the entire study.

3.4 Follow-up of the NCSS

All newborns during the study period, including those on NICU or special care, will be followed on a daily basis until hospital discharge to detect neonatal death and document severe morbidity. We will make strenuous efforts to coordinate and promote evidence-based care for the preterm neonates using materials developed as part of the WHO best practice programme, by liaising with the lead neonatologist in each NICU before and during the study. We recognize that differences in practice will persist despite our best efforts. However, we believe this is unavoidable in a very pragmatic study such as this, which is trying to reflect what happens on a daily basis in clinical practice. Furthermore, we will similarly make strenuous efforts to standardize the main protocols for feeding practices in each NICU before the study starts. During the routine site-visits by members of the Study Coordinating Unit and the Anthropometric Team we will monitor the implementation of the protocols.

3.5 Biological Samples

We have debated the issue of anonymized genetic/epigenetic data at great length. In principle, we would not wish to miss an opportunity to put together what would be a unique databank. However, we are very concerned about a) the effect that a request to collect DNA might have on recruitment rates; b) ethical questions that might be raised by IRBs; c) the additional time required to obtain informed consent, and d) the extra cost of processing the samples. Having said this, it nonetheless remains an important issue. Therefore, we shall bring together a group of international experts and potential academic partners, to discuss the advantages and disadvantages of collecting biological samples for a specific hypothesis relating to fetal growth and/or preterm delivery. The final decision will depend largely on whether the study has sufficient power to answer a specific question, relating for example to the effect of a genetic polymorphism on a pregnancy outcome. After much discussion, it was finally decided to collect biological samples from FGLS and NCSS pregnancies from 2011 onwards. For details of biological sample collection refer to Section 1.11 and Appendix K.

3.6 Severe Perinatal Morbidity and Mortality Outcomes

Birth weight for gestational age standards will be related to indicators of perinatal outcome to establish risk levels associated with different growth patterns. The 'ideal' outcome is perinatal mortality, but its anticipated infrequent occurrence in this low risk population makes it unrealistic to have a sample large enough for the necessary number of events across the gestational age distribution (perinatal mortality is expected to be close to 1% in these populations). We have therefore decided to use an un-weighted composite outcome including at least one of the following conditions: stillbirth, neonatal death until hospital discharge of the newborn, newborn stay in NICU for ≥7 days or other severe neonatal complications. We have used such an outcome recently (12, 13); it requires limited standardization of clinical diagnoses across hospitals and is well accepted as a marker in large, international, population based studies of newborns that are severely ill.[b] It could be argued, however, that intrapartum stillbirth may not be related to fetal growth and should not be included in this index. We believe this is a valid point but as it will not be possible to separate those intrapartum deaths that are related to IUGR from those that are unrelated, we suggest keeping the index as it is. As we expect a very low fetal death rate, it should not have a major impact on the observed rates.

We believe this is a good proxy for adverse perinatal outcomes across countries. We have used it as a primary neonatal outcome in recent publications and it has been well accepted. Its only disadvantage is that it risks excluding from the total number of early neonatal deaths some cases amongst healthy, mostly term babies delivered vaginally who, after hospital discharge at 48 hours, develop severe complications or death up to 7 days

[b] Others have also used these composite indices of neonatal morbidity (Hannah ME, Hannah WJ Kewson SA et al (2000); Wapner RJ, Sorokin Y, Thom EA (2006); Joseph KS, Fahey J, Platt R (2009))

For peer review only - http://bmjopen.bmj.com/site/about/guidelines.xhtml

post-natally without returning to the same hospital. However, missing these isolated cases is preferable to performing thousands of unnecessary home visits.

Data from our population-based studies in some of the sites indicate that the incidence of this outcome is close to 5%. We will confirm this estimate during the preparatory phase of the study in the study centres. We will also reach consensus with the investigators and consultants on the few very severe clinical conditions to be included in the composite outcome in addition to perinatal death and ≥7 days NICU stay.

4. On-line Data Management and Statistical Analysis

All data will be entered into an on-line data management system specifically developed for the study; it will include a system for direct transfer of blinded data from the ultrasound machines to the database. This on-line system has the practical benefit of allowing on-going quality control, correction of errors or missing values and the initiation of data analysis soon after data collection is completed. We have previously used such a system in very large multi-centre studies (12) and randomized clinical trials in developing countries, and we are confident that there will be few problems in adapting this concept to the proposed study. The system will be field tested during 2008 using the data flow model shown in Appendix B.

This system will be used for data management and monitoring all sub-studies, including patient recruitment and follow-up, and is based on the INTERGROWTH-21st Electronic Data Management System (IDAMIS). The system permits all participants' data to be incorporated contemporaneously into the data files via the Internet. Included within the system is a review process to ensure that all data are complete, and that the research team is notified about imminent patient visits and expected delivery dates. At an institutional level, each centre will have its own progress enrolment charts and weekly recruitment targets. The system will also provide the Data Coordinating Unit with a detailed daily record of patient enrolment and data entry, at both individual and institutional levels to monitor progress against the milestones listed in the protocol. Corresponding actions, such as telephone calls, web conferences or site visits will take place within a week of detecting a problem in a centre to ensure that appropriate corrective measures are introduced. Lastly, the system will allow the database to be analyzed as soon the data collection period has ended.

Data from all sites will be pooled to construct the curves for international applications using the WHO Child Growth Study analytical strategy (8). Data analysis will be conducted following the same strategy used in the construction of the WHO MGRS curves during Years 3-5. It will be coordinated by the Statistical Director (D. Altman), consultant statistician/epidemiologist and the team's senior investigators. Extensive secondary analyses of these data will be conducted including the development of velocity standards, thereby contributing in a major way to answering questions relating to clinical practice and public health now and for years to come.

The appropriateness of pooling data from all sites will be assessed by comparisons of site means, standard deviations and the 3rd and 97th centiles to the pooled values of primary data. Consistent differences ≥ 0.5 SD between the mean values of individual sites and the pooled sample will be used as a pre-set trigger for considering whether to adjust by site for purposes of pooling data. This has to be supported by site-specific consistent differences across primary measures. For the WHO-MGRS it was concluded that data from all sites were remarkably similar and thus should be pooled (8). This is the strongest biological argument to date and will be the basis for the conceptualization of our analysis.

The planned approach will be based on the experience of the WHO-MGRS adapted to the fetal growth parameters (37); we shall follow the same exploratory strategy used in this study. We are aware that these analyses may not be automatically applicable, but we feel comfortable that the considerable experience of this team of statisticians in carrying out a similar study will enable them to select a final model that is fit for purpose. The methods do not require similar intervals. We are not making any assumptions about the shapes of the individual curves, except that they will be smooth.

For the physical growth standards, a class growth distribution that can fit the data for all measurements will be explored with an appropriate smoothing technique to generate centiles. Candidate distributions (e.g. Box-Cox t, modulus-exponential-normal, Box-Cox-power-exponential, Johnson's S_U or Johnson's S_B) will be compared on the basis of goodness of fit for each age group using the following diagnostic tools: (a) Log-likelihood

For peer review only - http://bmjopen.bmj.com/site/about/guidelines.xhtml

comparisons, (b) Q-Q plots (i.e. comparing empirical and distributional centiles), and (c) Comparisons of observed percentages that occur below estimated centiles against expected values. Once the most appropriate distribution(s) for our data is (are) identified, smoothing techniques will be tested against the same diagnostic criteria applied to the selection of distributions such as polynomial smoothing, the Box-Cox t distribution that smoothes using natural splines and a variation of it with the Box-Cox-power-exponential distribution. Sir David Cox (University of Oxford Statistics Department) who advised us on another pregnancy-based study, will be invited to contribute to the selection process.

It could be argued that only parous women should be included in the construction of the standards. Furthermore, it would reduce the incidence of LBW babies amongst the mothers recruited as nulliparous women are known to be at higher risk of LBW babies. However, it is also the case that long-term outcomes in newborns born to parous versus nulliparous women have similar overall morbidity and mortality rates. Overall, we believe that the advantages of recruiting solely parous women are outweighed by the loss in external validity and credibility in doing so. Therefore, we intend including all women regardless of parity but we will try to recruit similar proportions across the study sites. We will also explore in the analysis if, in this sample of healthy women, parity is an important effect modifier for fetal growth, but we do not expect to produce parity-specific fetal growth standards. In addition, based on the same biological principles, sex-specific charts should be constructed for fetuses as they have been for newborns. However, we do not plan to separate fetuses by sex as this is not a routine practice and it is unlikely that it will became one in the future. Conversely, separate sex-specific charts will be developed to monitor the postnatal growth of preterm babies.

5. Sample Sizes for Objective I (Activities 1 - 3)

General principles: The precision of growth chart centiles is determined by several factors, of which the most important is sample size. Other factors are also relevant, including study design (cross-sectional vs. longitudinal), the timing of measurements, and the method of curve-fitting. Many criteria can be used to estimate sample size, but three are considered critical here, namely the precision of: a) a given centile at a particular gestational age; b) the logistics of selecting and following-up a large cohort, and c) the rate of the expected perinatal outcome for Activity 3, in Objective I. (The outcomes to be used in Objectives II-III are either continuous variables or far more prevalent than severe neonatal morbidity/mortality, i.e. a preterm delivery rate of 9-10%, and therefore sample size limitations are of less concern for these parts of the study). Sample sizes have been estimated taking each of these criteria into account. The numbers quoted below are combined figures for all study sites.

It is possible that the WHO-MGRS findings of similar infant growth patterns amongst different ethnic groups may not apply to fetal growth, even though recent data for US white/black newborns support the concept of pathological rather than physiological factors being responsible for observed variations (16). We question why fetuses should grow differently to infants or be affected differently by environmental influences, or why biologically different growth patterns should exist across ethnic groups. Nevertheless, we will make all practical efforts to obtain the largest possible sample size per ethnic group to allow separate evaluation in case the alternative hypothesis (different fetal growth by ethnic group) is observed. Even if this is the case for some groups it is very unlikely that all ethnic groups will have biologically relevant different growth patterns.

FGLS: To obtain complete data from 4,000 pregnancies at 8 study sites, 500 mothers will have to be enrolled at each centre. Data from previous years will be used at each study site to estimate the expected number of eligible women available and the recruitment rates. We expect that ~75% of the total pregnant population at the sites selected will be eligible for this cohort, although local investigators will be strongly advised to be conservative in their estimates of compliance rates and to allow for attrition due to other reasons. The 4,000 women should provide close to 800 cases of each of the 5 general ethnic groups. Sample size calculations (15), based on a width of the 95% CI of the 5th birth weight percentile of 2.5% at term (2,700g), demonstrate that 600 women per ethnic group should be adequate, after excluding complicated pregnancies and women lost to follow-up. This sample size is larger than most previous studies and adequate, we believe, to produce reliable curves.

For peer review only - http://bmjopen.bmj.com/site/about/guidelines.xhtml

However, we will not produce ethnic-specific standards because such pregnant populations are rarely representative of a unique ethnic group, particularly in urban areas in developing and developed countries. It would therefore be impractical to alternate between standards based on each patient's ethnic classification, even if this could be done. We will not be able to estimate outcomes across groups with any degree of power but that is not an objective of this study. These numbers should also fulfill the requirements for PPFS of ~360 preterm infants (assuming a preterm rate of 9% based on our previous study involving similar populations (12)).

We estimated that fewer than 5% of women will be lost to follow up (the figure is usually ~ 3% in our large trials). We also adjusted for the fact that 10-15% of women will be excluded from the preparation of the fetal growth standards because they will have developed problems severe enough to have affected fetal growth.

PPFS: A cohort of ~360 preterm babies will be recruited from FGLS for this study. It is expected that the gestational age distribution will provide 310 babies >30 weeks gestation. Only the subgroup of babies free of major clinical problems will be used to create the standards. We recognize that the sample size calculation here is influenced by logistic issues and the availability of newborns from FGLS rather than statistical calculations. However, it is still large by preterm study standards and we shall have very detailed follow-up data. We consider that the possibility of having a full set of fetal and newborn growth patterns from a cohort of preterm newborns is a biological priority even if we shall not have the power to explore other subgroup analyses such as gestational age sub-groups or early postnatal morbidity. Postnatal growth from healthy populations has been shown to be similar among ethnic groups (38, 39).

NCSS: The sample size calculations for this component of the study depend mostly on the number of preterm newborns needed at the lower end of the gestational age distribution and the perinatal outcome selected. A fixed data collection period (12 months) is planned for all hospitals but it is clear that some variability will have to be accepted considering the size of each institution and the population characteristics. From the pool of all newborns, we will select the ones born to eligible women using the FGLS criteria listed above. We expect the refusal rate to be minimal. We will aim for a total of 50,000 newborns with very detailed information which will provide, based on data from similar institution (40), a sample of ~75% eligible babies for the newborn charts, including ~1,800 with a severe outcome using our established criteria. Of these 1,800, we expect that 70% (about 1,200) will be between 26 and 36 completed weeks of gestation.

Missing values: We consider that women without newborn data cannot contribute to any of the three studies. Fetal deaths and newborns with congenital malformation will be excluded. All women should have at least 2 follow-up values after the dating scan to be included in the fetal growth study.

B. Objective II: Activities 4 - 6

Objective II will be achieved by the following three activities:

1) Preparation of computer files of 2D ultrasound measures for 3-week gestational windows between 20-34 weeks;

2) Development of analytical strategies for the construction of predictive models to estimate gestational age during mid-late pregnancy and

3) Completion of prediction model to estimate gestational age during mid-late pregnancy based on several ultrasound measures obtained at a single visit. Ultrasound-based gestational age estimation in infrequent attendees or women with limited access to care is usually based on a single ultrasound measure (e.g. BPD), but the estimates have large errors and wide confidence intervals. We are planning to develop equations that use several ultrasound measures obtained at a single visit. If we achieve this, it will be a major contribution to the care of high-risk women attending hospitals on only one or two antenatal visits late in pregnancy.

Statistical strategies for the production of the prediction models include multiple regression analysis using gestational age at delivery as the gold standard (dependent variable), then recalculating the expected gestational age at different time points. The independent variables or predictors will be the basic ultrasound measures at a given

gestational age window, as well as other clinical parameters that are available to the staff during the antenatal care.

C. Objective III: Determinants of LBW and its components, preterm delivery and impaired fetal growth, under current healthcare conditions (Activity 7)

The complex interactions between risk factors, clinical presentations and underlying biological processes are poorly understood in relation to adverse perinatal outcomes, especially LBW which is such a heterogeneous entity. This has been a major limitation in preventing preterm delivery and impaired fetal growth. We therefore aim to explore risk factors and perinatal outcomes for LBW (<2,500g), preterm delivery and impaired fetal growth in the entire NCSS study population of 50,000 newborns. For the identification of risk factors, data collected during routine and specialist antenatal care from ALL women delivering in the institutions over a fixed period will be included in the study. A summary antenatal and delivery form will be introduced into these institutions, which will conform with a) the new WHO model of antenatal care for basic routine care (10) and b) local protocols for special cases, standardized by us from previous trials in pre-eclampsia, hypertension, urinary tract conditions, and intra- and post-partum care. An important conceptual issue is that we do not aim to detect any new, unexplored risk factors. Rather, we plan to determine how risk factors, that are routinely recorded during standard antenatal care, are distributed in the preterm/IUGR cases and their subgroups across these populations, in view of the considerable heterogeneity in risks and outcomes within these two conditions.

We plan to investigate the determinants of preterm delivery and IUGR in sub-groups from clinical, routine laboratory, demographic and socio-economic variables obtained from all women attending the study centres without any exclusion (as opposed to FGLS which aims to produce standards from a sample of selected, healthy women). It would, of course, be very interesting to collect more detailed information about other variables or test biomarkers of, for example, infection. The question, as always, is when to stop adding more variables to an already complex study. One alternative is to add ancillary studies to selected centres that are interested in collaborative research with other groups. It would certainly not be impossible for us to coordinate the collection, storage and testing of such samples (as our group has experience of collecting thousands of samples for similar studies) but funding is not available for such an activity at this point.

We will apply standard statistical strategies (e.g. logistic regression analysis) using the ~4,500 preterm newborns or IUGRs from the total NCSS study population to study these relationships compared to term babies, and we shall explore some recently proposed analytical strategies as well (41). We will consider subgroups of preterm deliveries, e.g. induced vs. spontaneous deliveries; premature rupture of membranes; pre-eclampsia related vs. unexplained impaired fetal growth, as well other pathological and physiological conditions (42, 43). The adjusted ORs and confidence intervals for these risk factors as related to the different subgroups will be estimated and biologically plausible interactions will be explored. The association between subgroups of newborns and perinatal outcomes will be also examined. We also intend to explore several factors that might explain variability in fetal growth, e.g. parity, BMI, gestational age, position/presentation of the fetus, liquor volume, gender and number of measurements. We shall also introduce a comprehensive system for evaluating intra- and inter-observer variability using the 2D and 3D measures, as well as a random sample of retaken measures by an external ultrasonographer in each centre.

D. Objective IV: Acquisition of additional 3D images to create an anatomical and growth databank of individual fetal organs (Activities 8 - 9)

Activity 8: 3D Data Collection during FGLS

Data collection for this activity will be conducted immediately after the 2D measurements have been taken for FGLS. The 3D volumetric images of the head, abdomen, and femur will be acquired using the methods described in Section 1.6. We expect that the provider of the equipment will provide technical assistance to optimize data collection.

The primary purpose of the 3D component is to provide volumetric data to assess the quality of the 2D measures (BPD, OFD, HC, TAD, APAD, AC and FL). The secondary objectives are a) to evaluate currently used ultrasound measures of fetal volume with the

For peer review only - http://bmjopen.bmj.com/site/about/guidelines.xhtml

expectation that some of them can contribute to the improvement of the presently used estimation of fetal weight and b) to establish a unique "biological databank" for future research. Additional 3D volumes of the chest (at the level of the 4-chamber view of the heart) and the humerus will be taken specifically for these purposes in some centres.

The secondary objective a) is very interesting from a clinical point of view as most of the ultrasound-derived equations for estimation of fetal weight have low predictability and were developed using data collected from small studies with less sophisticated ultrasound equipment than is now available. Thus, the feasibility of combining 2D and 3D measures will be explored including the possibility of doing so across gestational ages. We expect to evaluate the additional benefits that could be obtained by adding 3D values in a sub-sample of the study.

Other uses of the 3D data are planned: for example, the accuracy of fetal weight estimation based on 2D measures is known to be poor in clinical practice. It is expected that 3D data will improve the formulae that are still in use despite being produced over 20 years ago. Furthermore, the likelihood of low-cost, portable 3D equipment being available in the future raises the possibility of managing high-risk pregnancies in rural or distant regions by sending the images to a referral centre rather than the mother herself. We plan to take the final decision regarding the amount of 3D data to be collected and the number of participating sites after conducting a pilot study assessing the human resources available at the centres, the need for additional training, the time that 3D scanning adds to each visit, equipment considerations and the feasibility of transferring the very large datasets to the Coordinating Unit in Oxford. Issues we shall explore in the pilot study include: a) difficulties obtaining individual organ volumes (44); b) the need to obtain at least two good volume measures from most of the areas of interest which may require 20-30 minutes additional scanning; c) the need for the audit staff to know the values of the calculated volumes and the way the operator arrived at each volume which could introduce considerable bias, and d) how to save and transmit the volumetric data reliably given the risks of a hard drive crashing or being corrupted. In any case, it is unlikely that we shall record more than the 5 volumes listed above at each visit, and it is also worth mentioning that the Gates Foundation has not funded the 3D component of the study.

We have debated the issue of adding more ultrasound measures (i.e. we have a unique opportunity that may not be repeated again) with the Ultrasound Advisory Group and other experts in the field, as we are concerned that the potential list of additional 2D/3D measures is long. As is the case in selecting which data to collect in any large study, one has to balance the enthusiasm of the researchers against pragmatism. We have chosen to concentrate on taking a few measures well (and more than once) in the time available during each scanning session rather than risk the possibility that additional measurements (including some not used in routine clinical practice) will be taken hurriedly. Of course, it will be possible to take further 2D measurements retrospectively for research purposes, if needed, from the acquired 3D volumes.

Activity 9: Preparation of 3D Files and Exploratory Analyses

There are two goals of the 3D component of this project. The first is to understand better the clinical advantages of making manual and automated fetal measurements on 3D v. 2D data. This should lead to a clearer understanding of IUGR and the relationship between different growth patterns and fetal/neonatal outcomes, as well as the impact of delayed growth in individual fetal organs and health care problems in adult life (e.g. an increased risk of osteoporosis). The second goal is to derive statistical models of the fetus or parts of the fetus to provide informative visualizations of nominal shapes and growth data at different gestational ages.

One possible approach will be to build parts model of the fetus, and to deform it to different instances of data acquired at the same gestational age. This will not be a simple task and will require theoretical and technical advances in biomedical image analysis. The research will be led by A.Noble, Professor of Engineering Science, University of Oxford, and forms the basis of a separate grant application to a UK agency, the UK Engineering and Physical Sciences Research Council. The aims of Activity 9 are simply therefore to prepare the 3D images and commence these exploratory analyses. Examples of what is currently possible include studying the relationship between a) brain growth and neonatal/infant

For peer review only - http://bmjopen.bmj.com/site/about/guidelines.xhtml

neurodevelopment (45) and b) fetal bone growth and neonatal/infant bone density to assess the origins of osteoporosis (46).

Ethical Issues

The study will comply with the International Ethical Guidelines for Biomedical Research Involving Human Subjects. Ethical safeguards will include:

Ethical approval at international, national and local levels: The study protocol will be submitted to the relevant UK ethics committee in keeping with the University of Oxford's responsibilities as the study sponsor, as well as the appropriate national bodies of the countries included in the study (if required) and all local ethics committees. Ultrasound and anthropometric research involves minimal, if any, risk to the mother and fetus and is part of routine care in these institutions.

Individual informed consent: Written informed consent will be obtained from all women enrolled in the study after the study objectives and procedures have been described in detail. The information will be provided in written form (translated into all the required languages) and orally by the researchers themselves based on a standard template, which can be referred to whenever needed. Women will only be recruited if it is clear they understand what the research entails and once they have had sufficient time (at least 48 hours) to consider whether to participate or not. All information collected in these studies will be made available to the clinicians responsible for the women's care; however, the 2D ultrasound measurements will only be made available after the data have been submitted on-line (see 1.2 above).

Discontinuation: Women who have agreed to participate in FGLS may decide to leave the study at any time without adversely affecting their care in any way.

Confidentiality: All phenotypic and imaging data will be available for clinical use at each centre. For research purposes, privacy rules will be maintained in line with the *Health Insurance Portability and Accountability Act of 1996* (HIPAA). All individuals will be de-identified to ensure confidentiality. At the Coordinating Unit, all phenotypic and imaging data will therefore be stored, transmitted and analyzed anonymously.

Data sharing for clinical care: All the clinical data we plan to collect are being obtained anyway as part of routine clinical practice in these institutions; the data will therefore will be available to the providers at all times. Our proposed policy with regard to the ultrasound measures is as follows: a) the gestational age estimate will be incorporated into the medical records; b) an ultrasound examination for structural congenital malformations will be performed at the time of the 3rd scan, if this conforms with local practice, and the results will be incorporated into the medical records; c) as far as 2D ultrasound measurements are concerned, after the blinded values have been submitted electronically to the dataset, the last measurement will be provided for clinical use, and d) no 3D data will be provided for clinical use.

Finally, there are concerns about the misuse of ultrasound for sex selection in places where it may be used (especially if the practice is illegal). We shall therefore be very vigilant in regions where this practice is known to occur and monitor sex ratios in the sample continuously.

E. Challenges

Implementing this project presents major challenges for the research team. However, our experience in conducting large-scale studies in pregnancy means that we are well prepared to face the challenges, some of which may be difficult to resolve:

Recruitment: We do not anticipate capacity problems in recruiting a large number of women (4,000) from 8 centres, but it remains unclear what proportion of women in each centre will actually be eligible for FGLS given the inclusion criteria. We anticipate an average eligibility of 75%, and expect the centres to devise local strategies to maximize that number.

Follow-up: We do not anticipate problems retaining pregnant women during follow-up but we do recognize that such a large follow-up study requires a commitment from participants and staff. In addition, the planning and coordination of the ultrasound scans with only one ultrasound machine available per centre will require considerable organization in the hospitals that should not be underestimated. Follow-up of the newborns presents the following challenges: a) difficulties measuring very small infants, especially those in an

For peer review only - http://bmjopen.bmj.com/site/about/guidelines.xhtml

incubator; b) morbidities associated with prematurity, which may modify the measures to be taken; c) mortality affecting the final sample size, which explains why we intend focusing on healthy preterms, and d) follow-up after discharge is a problem in any such study, but we expect that adherence to the protocol will be very good for the preterm babies given the parents' concerns for their health.

Data quality: Standardization of a large number of health professionals is always a major challenge for which we are prepared. Coordinating travel arrangements between centres to monitor data quality will need to be well organized.

Data analysis: Constructing growth curves is a sophisticated task for which several statistical methods are available; the experience gained in the WHO study is a major asset to overcome this challenge.

Sample size: Selecting an adequately powered sample size for studies like this one is always a compromise between statisticians' estimates, logistics and cost. We are concerned that we should not over-extend our study by attempting to answer too many questions. Ideally, we should have a sample size to create preterm standards with stable outer centiles and birth weight for gestational age standards with sufficient newborns with severe outcomes across the gestational age distribution. We would also like to explore ethnic-specific growth, in the event that multiple ethnic differences emerge from the data. However, based on the findings of the WHO Child Growth Study, we consider it unlikely that each ethnic group will have its own distinct growth pattern. Nevertheless, we shall explore this question by including women from each of the 5 ethnic groups: Whites, Latin Americans, African blacks, Orientals and Indians.

Motivation: The final challenge, but perhaps the most important, is to maintain motivation of the local investigators and data collection teams throughout the project, as they are the people who can guarantee the quality of the study.

Resources: Finally, we appreciate that advocating the introduction of new fetal growth standards into clinical practice in developing countries could be criticized on the grounds that it will require already under resourced institutions to purchase relatively expensive ultrasound equipment. We accept the possibility that some institutions will purchase ultrasound equipment based on the results of this study, despite our recommendation that serial ultrasound measurements are not necessary for the evaluation of fetal growth during routine antenatal care (10). However, many district level and referral hospitals in developing countries already have ultrasound machines and when they are used to evaluate high risk pregnancies and/or women with uncertain gestational age, the results may be misinterpreted or misused as the charts available are often inadequate. Therefore, much of this project is orientated towards more effective use of equipment that is already in such institutions.

Interestingly, the expensive and very sophisticated 3D imaging system being investigated in this study, for the first time on this scale, has potential to benefit high-risk pregnancies in rural areas and isolated communities. The rationale is that it will become feasible to transfer images from peripheral clinics to specialized centres for reconstruction and interpretation as the cost of the equipment falls and it becomes more portable. Such a strategy would reduce the transfer of large numbers of women unnecessarily as occurs at present and contribute to the decentralization of antenatal care services worldwide.

F: Regional distribution of Study Sites:

We have recruited study sites in the following regions of the world: Europe (UK); North America (USA); Central and South America (Brazil); Africa (Kenya); Arab World (Oman); Asia (China) and the India subcontinent (India).

G. Intervention Effectiveness for Impaired Fetal Growth

We ourselves have explored the question of what to do after IUGR is diagnosed and we have published a series of review papers critically evaluating RCT interventions for IUGR (47-50) and another group's recent update (51). There are not many interventions for treating (as opposed to preventing) IUGR, other than planned elective delivery, to which use of ultrasound contributes greatly in developing countries. This explains why referral to an adequate level of perinatal care is so important an option. Our study therefore focuses on helping clinicians to detect impaired fetal growth accurately, which should avoid incorrect diagnoses and, thereby, iatrogenic preterm births. The misuse of this technology is one of

For peer review only - http://bmjopen.bmj.com/site/about/guidelines.xhtml

the factors responsible for unnecessary medical interventions and why some treatments or interventions are shown to be ineffective, i.e. treatment of fetuses that may not have required it in the first place. Furthermore, in the light of information contained in the Lancet 2008 Nutrition Series and other similar recent reports, we will consider the possibility of implementing nested case-control studies to obtain information related to maternal body composition, metabolism and micronutrient status, or growth factors and birth outcomes. We will explore them in detail as this is a unique opportunity for such studies.

We think that interventions can only be effective if they focus on the factor responsible for the growth restriction. IUGR is a very heterogeneous condition, as we have shown, and it is unlikely that a "silver bullet" will resolve all IUGR (or preterm deliveries), even if correctly detected. Overall, we believe that accurate early identification, avoiding false positive cases, and appropriate referral to adequate levels of obstetric and newborn care will have a great impact on neonatal mortality and resource allocation in developing countries.

For peer review only

VIII. Monitoring, Evaluation, and Dissemination

A. Monitoring and Evaluation

Data collection will be monitored using routine procedures and protocols employed by our network in several similarly sized, multi-centre studies. The production and evaluation of specific standards will undergo a methodical and rigorous peer review process through the expert consultation mechanisms used in previous projects: the WHO Child Growth Study and the perinatal multi-centre studies conducted by our network. Monitoring the implementation process at the study sites will follow principles established in implementing previous, similarly complex, studies carried out by Consortium members. This will include site visits and on-going quality control measures (see Section IV).

B. Dissemination of Results

The generous participation of hundreds of scientists in all aspects of dissemination was the key to the success of the WHO Child Growth Study and we will therefore rely on their already established system. So, involving the Maternal Health Task Force at the Harvard School of Public Health in disseminating the results locally will be our principal strategy; this includes local and regional meetings and symposia. We will then focus on the local and global professional societies that are an integral part of the application, as well as health authorities in the participating countries and regional health institutions. Finally, a formal dissemination strategy similar to the one used by the WHO Child Growth Study will be implemented, including partnerships with other key players in the international field such as UNICEF, other UN agencies, NGOs and government institutions.

For peer review only

IX. Optimizing Public Health Outcomes and Intellectual Property Plans to Achieve Global Access

It is expected that these international standards will be used to assess the growth of fetuses and newborns throughout the world. The basic assumption behind the proposed standards is that fetal growth is optimal in healthy populations. Therefore, the proposed curves will constitute “optimal” standards. Widespread uptake of these fetal curves will be ensured by ultrasound equipment manufacturers incorporating them into their systems. The potential uses are:

1) Population assessment to a) provide a reference for comparison of the means (or medians), standard deviations, and trajectories of the population means (or medians) for any given sample, b) estimate the proportions of fetuses-newborns below a given cut-off in a sample, and c) standardize for gestational age and sex differences between samples, thereby allowing comparisons of prevalence rates below a given cut-off across samples.

2) Individual assessment to a) screen for fetal growth on a single occasion to identify fetuses with excess or deficient growth leading to appropriate diagnostic and/or therapeutic interventions (including delivery), and b) provide a clinical tool for assessing the efficacy of treatments or helping to choose time of delivery, particularly in severely growth restricted fetuses. Hence, the primary use of the curves at an individual level will be to identify unacceptable deviations as early as possible to prevent severe deficits and excesses, although they will need to be introduced into clinical practice with appropriate care and auditing. We expect to link with, and build upon, WHO’s global efforts to disseminate the child growth charts. The large network of proposed partners and consortium members should prove, with the help of industry, to be a very effective method for disseminating the results at both clinical and public health levels.

A. Intellectual Property (IP) Plan.

	Yes	*No*
1. Is the proposed research likely to lead to any patentable or commercially exploitable results?		X
The study will provide each centre with a sophisticated (commercially available) 3D ultrasound machine, specially adapted for the needs of the study, as well as technical expertise for data transfer and storage of 2D and 3D data. The proposed research is unlikely to lead to patentable results. Ultrasound machine manufacturers throughout the world will be encouraged as part of the Dissemination and Implementation Plan to incorporate the new standards into their machines from which they may derive commercial benefit.		

2. Will the proposed project, either at its inception or at a foreseeable future point, depend on the use of technologies, materials, or other inventions that may conflict with goals of global access in terms of either cost or availability in the developing world?		X

3. Is the proposed project and related IP subject to any agreements (e.g., licenses, collaborations, research or funding agreements or any other form of agreement) with commercial, academic, or other organizations, including other funding entities, subgrantees or subcontractors?		X

4. Does your organization plan to assume responsibility for maturation, production, and dissemination of the innovation itself?	X	

For peer review only - http://bmjopen.bmj.com/site/about/guidelines.xhtml

B. Commitment to Sharing Data and Materials

It is highly unlikely that patentable IP will be generated by this project. The growth standard and related analyses represent the new evidence-based knowledge that will be generated. These will be widely disseminated, especially in open access publications, and shared with research and clinical communities worldwide, as well as the appropriate national and international agencies.

The Fetal Growth Standards charts will be made available to all manufacturers of ultrasound machines. Data from each centre will be provided to the local investigators with the understanding that the primary and secondary objectives of this study can only be explored with the pooled data set and can not be replicated using local data. Other locally relevant issues can be analyzed by individual centres.

The study Steering Committee (SC) and Executive Committee (EC) will also, after the main tasks are completed, engage in negotiations with other *bona fide* researchers for access to the data set to allow scientific and public health relevant questions to be explored. The SC and the EC have developed a set of rules and procedures for the evaluation of such requests in keeping with the commitment of the present investigators to ensure the widest possible utilization of the data (see Appendix F).

C. How will we bridge the new data to the existing data?

We aim to produce the following new standards:

1. **Fetal growth by ultrasound:** Based on discussions with ultrasound manufacturers and the known limitations of existing charts, we anticipate that the newly developed fetal growth charts will replace all charts that are currently incorporated into ultrasound machines.
2. **Postnatal growth for preterm babies:** Based on discussions with a number of pediatricians in different countries and WHO information, we anticipate that the postnatal growth charts for preterm babies will be incorporated into routine clinical practice without much difficulty because there is a lack of well-developed charts for this specific purpose. We believe that the international community is looking forward to such charts being developed.
3. **Birth weight for gestational age standards:** It is likely that the new, international, risk-related, birth weight for gestational age standards will be widely adopted following implementation of the new fetal growth and preterm postnatal growth standards. However, we acknowledge that this process may take longer because other standards are already being used (even though their limitations are recognized). We shall therefore work closely with the Maternal Health Task Force at the Harvard School of Public Health to follow the strategy they are using for the infant growth standards.

We do not anticipate any conceptual limitations to the future use of the standards, particularly in the light of the statement in the recently published Lancet "Maternal and Child Undernutrition" series that international fetal and newborn growth standards need to be developed (5). Nevertheless, it is clear that whenever efforts are being made to change clinical practice (even if the need to do so is recognized), common barriers and resistance to change will be encountered. We feel that our experience in introducing the MGRS standards will be invaluable for this task although, in theory at least, introducing the ultrasound standards should present fewer problems as they will be built into the equipment and we hope they will be endorsed by the International Society of Ultrasound in Obstetrics & Gynecology and other professional associations responsible for recommending new technology into clinical practice.

Rolling out the fundal-height charts and the newborn and preterm growth standards will use the same procedures as WHO did when it rolled out its Child Growth Standards. We shall coordinate these processes from the start of INTERGROWTH-21st by liaising closely with both the Maternal Health Task Force at the Harvard School of Public Health and the leading manufacturers of ultrasound machines. The network already developed by the team will serve as a means of rolling out the standards we propose developing in INTERGROWTH-21st.

For peer review only - http://bmjopen.bmj.com/site/about/guidelines.xhtml

X. Organizational Capacity and Management Plan

A. Organizational Capacity and Facilities

History: The University of Oxford enjoys an international reputation as a world-class centre of excellence in research and teaching. It employs over 7,800 academic, research and support staff across a wide range of academic disciplines. The Medical Sciences Division, within which the Nuffield Department of Obstetrics & Gynaecology (NDOG) is located, is one of the major centres for clinical and basic biomedical research in Europe, with more than 2,200 staff and 800 postgraduate students. It achieved top scores in both the 1996 and 2001 UK HEFCE research assessment exercises, and fosters the highest possible standards in research, teaching and patient care. The annual grant income from external sources for the Medical Sciences Division is approximately $200M.

Experience: The Consortium members (University of Oxford and collaborating centres) have extensive experience in conducting multi-centre studies and constitute perhaps the largest research network in the world in the field of women's and perinatal health. NDOG has coordinated large-scale genetic epidemiology studies across multiple international sites, involving data collection from thousands of individuals in the ENDOGENE Study. Office space within its existing facilities at the John Radcliffe Hospital, Oxford, will be provided to house the Coordinating Unit (CU) staff, as well as the necessary IT support and administrative assistance. Sites have been chosen on the understanding that they have the necessary infrastructure to conduct the study (e.g. IT and capacity for ultrasound research). Finally, the professional societies recruited to help coordinate implementation and dissemination of the results are among the leading groups in the world in the field of obstetrics.

B. Management and Staffing Plan

Management: The study will be coordinated and managed by NDOG (University of Oxford), where the CU will be located (see Appendix F). Four units responsible for day-to-day monitoring of quality control and data collection (2D Ultrasound Data Quality Unit; Anthropometric Data Quality Control Unit; Data Management Unit and 3D Ultrasound Data Quality Unit) will report directly to the CU. All data, except the 3D data, will be centralized at the Data Management Unit.

The Steering Committee (SC) consists of representatives of Consortium members and the lead investigator from each of the study centres. It has also external members to provide support in areas related to the study. The SC will meet twice a year to review progress, ensure uniformity of data collection from the study sites, and discuss any substantive issues that arise. Any adaptations to the protocol or technical variations required at individual sites because of local needs will be reviewed and approved by the SC.

The Executive Committee (EC) will monitor the progress of the study on a regular basis and resolve substantive issues arising from implementation of the study. The Study Coordinator will be ex-officio member of the EC. The EC will make the final selection of study sites; exclude a site if data collection standards are not sufficiently high, and make final decisions about sample selection and analytical issues related to construction of the standards.

The AC, consisting of internationally recognized experts in ultrasound, obstetrics, statistics, nutrition, fetal development, anthropometrics, epidemiology and biology, will provide technical advice to the CU, EC and SC. The study will be indemnified by the University of Oxford that will carry liability for any adverse outcomes arising from breaches of research protocol.

Study coordination:

The **Principal Investigator** (José Villar) will be responsible for all scientific and technical components of the project. The **Project Director** (Stephen Kennedy) will be the focal person in NDOG responsible for overall study management. The **Project Leader** (Leila Cheikh Ismail), will be responsible for day-to-day implementation of the study, coordinating and monitoring of all activities as well as training, monitoring and evaluation. A **Statistical**

For peer review only - http://bmjopen.bmj.com/site/about/guidelines.xhtml

Director (Doug Altman) will advise on sampling strategies, sample sizes and all statistical methods, in particular those for the construction of the new standards. He will be assisted in Years 1-5 by a **Data Management Supervisor** (Stephen Ash), who will coordinate all aspects of data management between the Data Management Unit (DMU), the CU in Oxford and the other data quality units. This will include the preparation of data sets, quality control strategies and data files for use by the team of statisticians. This strategy for data management and analysis has been extremely efficient in previous, large multi-centre studies conducted by us as it allows for direct contact between the data collection centres and the coordinating unit. In Years 3-5, Dr Altman will also be assisted by a **Senior Statistician** to develop, test and implement computer programs to construct the new growth standards.

There will be a **Senior Technical Coordinator** (Aris Papageorghiou, St George's, London) of the ultrasound component of the study, who will have responsibility for overall coordination of the ultrasound measurements, including quality control and standardization. He will be assisted by an **External Ultrasound Expert** (Laurant Salomon), acting as a consultant and a **Clinical Research Fellow** (Caroline Knight) to oversee the quality of a random sample of ultrasound measurements during the data collection period. The CU team will be completed by a **Secretary** (Melissa Shorten) responsible for secretarial support to the CU, a **Project Administrator** (Ann Lambert) in charge of all aspects of transferring funds to the study centres, equipment, meetings, travel and coordination between the CU, DMU and study centres, and a **Research Associate** (Isabelle Wilson) responsible for piloting protocol and monitoring its implementation.

For peer review only

XI. Citations

1. Lawn JE, Cousens S, Zupan J. 4 million neonatal deaths: when? Where? Why? Lancet 2005;365:891-900.
2. Williams RL, Creasy RK, Cunningham GC, Hawes WE, Norris FD, Tashiro M. Fetal growth and perinatal viability in California. Obstet Gynecol 1982;59:624-32.
3. Rao SC, Tompkins J. Growth curves for preterm infants. Early Hum Dev 2007;83:643-51.
4. de Onis M, Habicht JP. Anthropometric reference data for international use: recommendations from a World Health Organization Expert Committee. Am J Clin Nutr 1996;64:650-8.
5. Black RE, Allen LH, Bhutta ZA, Caulfield LE, de Onis M, Ezzati M et al. Maternal and child undernutrition: global and regional exposures and health consequences. Lancet 2008;371:243-60.
6. de Onis M, Onyango AW, Van den Broeck J, Chumlea WC, Martorell R. Measurement and standardization protocols for anthropometry used in the construction of a new international growth reference. Food Nutr Bull 2004;25:S27-36.
7. Kramer MS, Platt RW, Wen SW, Joseph KS, Allen A, Abrahamowicz M et al. A new and improved population-based Canadian reference for birth weight for gestational age. Pediatrics 2001;108:E35.
8. de Onis M, Garza C, Onyango AW, Martorell R. WHO Child Growth Standards. Acta Paediatr 2006;450:1-101.
9. Zhang X, Platt RW, Cnattingius S, Joseph KS, Kramer MS. The use of customised versus population-based birthweight standards in predicting perinatal mortality. BJOG 2007;114:474-7.
10. Villar J, Ba'aqeel H, Piaggio G, Lumbiganon P, Miguel Belizan J, Farnot U et al. WHO antenatal care randomised trial for the evaluation of a new model of routine antenatal care. Lancet 2001;357:1551-64.
11. Kelly Y, Panico L, Bartley M, Marmot M, Nazroo J, Sacker A. Why does birthweight vary among ethnic groups in the UK? Findings from the Millennium Cohort Study. J Public Health 2008;epub Jul 21.
12. Villar J, Valladares E, Wojdyla D, Zavaleta N, Carroli G, Velazco A et al. Caesarean delivery rates and pregnancy outcomes: the 2005 WHO global survey on maternal and perinatal health in Latin America. Lancet 2006;367:1819-29.
13. Villar J, Abdel-Aleem H, Merialdi M, Mathai M, Ali MM, Zavaleta N et al. World Health Organization randomized trial of calcium supplementation among low calcium intake pregnant women. Am J Obstet Gynecol 2006;194:639-49.
14. Gulmezoglu AM, Villar J, Ngoc NT, Piaggio G, Carroli G, Adetoro L et al. WHO multicentre randomised trial of misoprostol in the management of the third stage of labour. Lancet 2001;358:689-95.
15. Royston P. Constructing time-specific reference ranges. Stat Med 1991;10:675-90.
16. Kramer M, Victora CG. Low birth weight and perinatal mortality. In: Nutrition and Health in Developing Countries. Totowa, NJ: Humana Press, 2001:57-69.
17. Carroli G, Rooney C, Villar J. How effective is antenatal care in preventing maternal mortality and serious morbidity? An overview of the evidence. Paediatr Perinat Epidemiol 2001;15 Suppl 1:1-42.
18. Villar J, Carroli G, Zavaleta N, Donner A, Wojdyla D, Faundes A et al. Maternal and neonatal individual risks and benefits associated with caesarean delivery: multicentre prospective study. BMJ 2007;335:1025.
19. Merchant KM, Villar J. Maternal height and newborn size relative to risk of intrapartum caesarean delivery and perinatal distress. BJOG 2001;108:689-96
20. Goedhart G, van Eijsden M, van der Wal M, Bonsel G. Ethnic differences in term birthweight: the role of constitutional and environmental factors. Paediat Perinat Epidemiol 22:4:360-368
21. Mortensen LH, Diderichsen F, Arntzen A, Gissler M, Cnattingius S, Schnor O et al. Social inequality in fetal growth: a comparative study of Denmark, Finland, Norway and Sweden in the period 1981-2000. J Epidemiol Community Health 2008;62:325-31.

For peer review only - http://bmjopen.bmj.com/site/about/guidelines.xhtml

22. Lynch CD, Zhang J. The research implications of the selection of a gestational age estimation method. Paediatr Perinat Epidemiol 2007;21 Suppl 2:86-96.
23. Robinson HP, Fleming JE. A critical evaluation of sonar "crown-rump length" measurements. Br J Obstet Gynaecol 1975;82:702-10.
24. Group MGRS, de Onis M. Complementary feeding in the WHO Multicentre Growth Reference Study. Acta Paediatr Suppl 2006;450:27-37.
25. Neufeld LM, Haas JD, Grajeda R, Martorell R. Ultrasound measurement of fetal size in rural Guatemala. Int J Gynaecol Obstet 2004;84:220-8.
26. Blake KV, Gurrin LC, Beilin LJ, Stanley FJ, Kendall GE, Landau LI et al. Prenatal ultrasound biometry related to subsequent blood pressure in childhood. J Epidemiol Community Health 2002;56:713-8.
27. British Ultrasoud Society. Fetal Measurements Document, 2008.
28. Verburg BO, Steegers EAP, De Ridder M, Snijders RJM, Smith E, Hofman A, Moll, HA, Jaddoe VWV, Wittema JCM. New charts for ultrasound dating of pregnancy and assessment of fetal growth: longitudinal data from a population-based cohort study. Ultrasound Obstet Gynae:31:388
29. Thorsell M, Kaijser M, Almstrom H, Andolf E. Expected day of delivery from ultrasound dating versus last menstrual period--obstetric outcome when dates mismatch. BJOG 2008;115:585-9.
30. Platt LD. Three-dimensional ultrasound, 2000. Ultrasound Obstet Gynecol 2000;16:295-8.
31. Belizan JM, Villar J, Nardin JC, Malamud J, De Vicurna LS. Diagnosis of intrauterine growth retardation by a simple clinical method: measurement of uterine height. Am J Obstet Gynecol 1978;131:643-6.
32. Khan NZ, Muslima H, Begum D, Shilpi AB, Akhter S, Bilkis K, et al. Validation of Rapid Neurodevelopmental Assessment Instrument for Under-Two-Year-Old Children in Bangladesh. Pediatrics. 2010; **125**(4): e755-e62.
33. Adoh TO, Woodhouse JM. The Cardiff acuity test used for measuring visual acuity development in toddlers. Vision Research. 1994; **34**(4): 555-60.
34. Kihara M, Hogan AM, Newton CR, Garrashi HH, Neville BR, de Haan M. Auditory and visual novelty processing in normally-developing Kenyan children. Clinical Neurophysiology. 2010; **121**(4): 564-76.
35. So K, Michael Adamson T, Horne RSC. The use of actigraphy for assessment of the development of sleep/wake patterns in infants during the first 12 months of life. Journal of Sleep Research. 2007; **16**(2): 181-7.
36. McGuire W, Henderson G, Fowlie PW. Feeding the preterm infant. BMJ 2004;329:1227
37. Borghi E, de Onis M, Garza C, Van den Broeck J, Frongillo EA, Grummer-Strawn L et al. Construction of the World Health Organization child growth standards: selection of methods for attained growth curves. Stat Med 2006;25:247-65.
38. Bhandari N, Bahl R, Taneja S, de Onis M, Bhan MK. Growth performance of affluent Indian children is similar to that in developed countries. Bull World Health Organ 2002;80:189-95.
39. Owusu WB, Lartey A, de Onis M, Onyango AW, Frongillo EA. Factors associated with unconstrained growth among affluent Ghanaian children. Acta Paed 2004;93:1115-9.
40. Villar J, Abalos E, Carroli G, Giordano D, Wojdyla D, Piaggio G et al. Heterogeneity of perinatal outcomes in the preterm delivery syndrome. Obstet Gynecol 2004;104:78-87.
41. Kramer MS, Ananth CV, Platt RW, Joseph KS. US Black vs White disparities in foetal growth: physiological or pathological? Int J Epidemiol 2006;35:1187-95.
42. Zhang J, Villar J, Sun W, Merialdi M, Abdel-Aleem H, Mathai M et al. Blood pressure dynamics during pregnancy and spontaneous preterm birth. Am J Obstet Gynecol 2007;197:162 e1-6.
43. Villar J, Carroli G, Wojdyla D, Abalos E, Giordano D, Ba'aqeel H et al. Preeclampsia, gestational hypertension and intrauterine growth restriction, related or independent conditions? Am J Obstet Gynecol 2006;194:921-31.
44. Chang CH, Yu CH, Ko HC, Chen CL, Chang FM. Predicting fetal growth restriction with liver volume by three-dimensional ultrasound: efficacy evaluation. Ultrasound Med Biol 2006;32:13-7.

For peer review only - http://bmjopen.bmj.com/site/about/guidelines.xhtml

45. Huang H, Zhang J, Wakana S, Zhang W, Ren T, Richards LJ et al. White and gray matter development in human fetal, newborn and pediatric brains. Neuroimage 2006;33:27-
46. Cooper C. Epidemiology of osteoporotic fracture: looking to the future. Rheumatology (Oxford) 2005;44 Suppl 4:iv36-iv40.
47. Villar J, Gulmezoglu AM, de Onis M. Nutritional and antimicrobial interventions to prevent preterm birth: an overview of randomized controlled trials. Obstet Gynecol Surv 1998;53:575-85.
48. Gulmezoglu M, de Onis M, Villar J. Effectiveness of interventions to prevent or treat impaired fetal growth. Obstet Gynecol Surv 1997;52:139-49.
49. de Onis M, Villar J, Gulmezoglu M. Nutritional interventions to prevent intrauterine growth retardation: evidence from randomized controlled trials. Eur J Clin Nutr 1998;52 Suppl 1:S83-93.
50. Kulier R, de Onis M, Gulmezoglu AM, Villar J. Nutritional interventions for the prevention of maternal morbidity. Int J Gynaecol Obstet 1998;63:231-46.
51. Iams JD, Romero R, Culhane JF, Goldenberg RL. Primary, secondary, and tertiary interventions to reduce the morbidity and mortality of preterm birth. Lancet 2008;371:164-75.

For peer review only

XII. Appendices

Appendix A: Data Collection Forms

Three different types of data recording forms will be used:

FGLS:
1) Screening: Interview
2) Screening: Ultrasound Dating
3) Maternal Study Entry
4) Ultrasound Follow-up
5) Pregnancy Follow-up
6) Pregnancy and Delivery (including newborn anthropometrics)
7) Referral/Admission
8) Adverse Events
9) Fetal and Neonatal Abnormality

PPFS:
1) Preterm Follow-up
2) Preterm End of Study
3) Preterm Referral/Admission

NCSS: 1) Pregnancy and Delivery (including newborn anthropometrics) - same as FGLS

It should be emphasized that all forms are as short as possible to improve compliance. Therefore, all questions have been carefully evaluated to ensure that they serve at least one of the following purposes: a) eligibility (e.g. socio-economic status); b) sample description (demographic and environmental variables, etc.); c) exclusion criteria (major illnesses, etc.); d) standardization of results across centres; e) future use of references (vitamin/mineral supplements), or f) to assess possible selection biases.

Appendix B: Data Flow Models
FETAL GROWTH LONGITUDINAL STUDY DATA FLOW

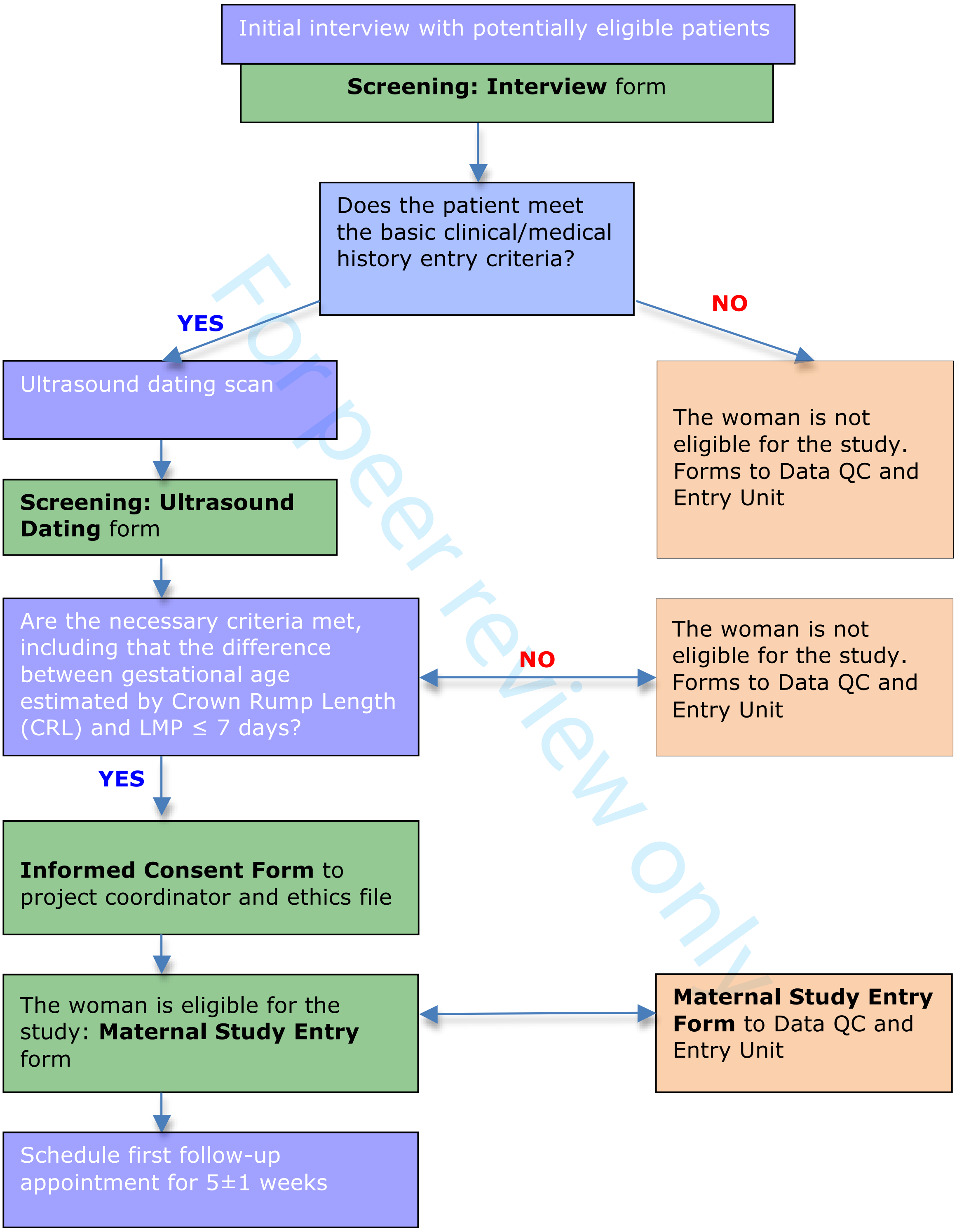

For peer review only

FETAL GROWTH LONGITUDINAL STUDY DATA FLOW (CONT.)

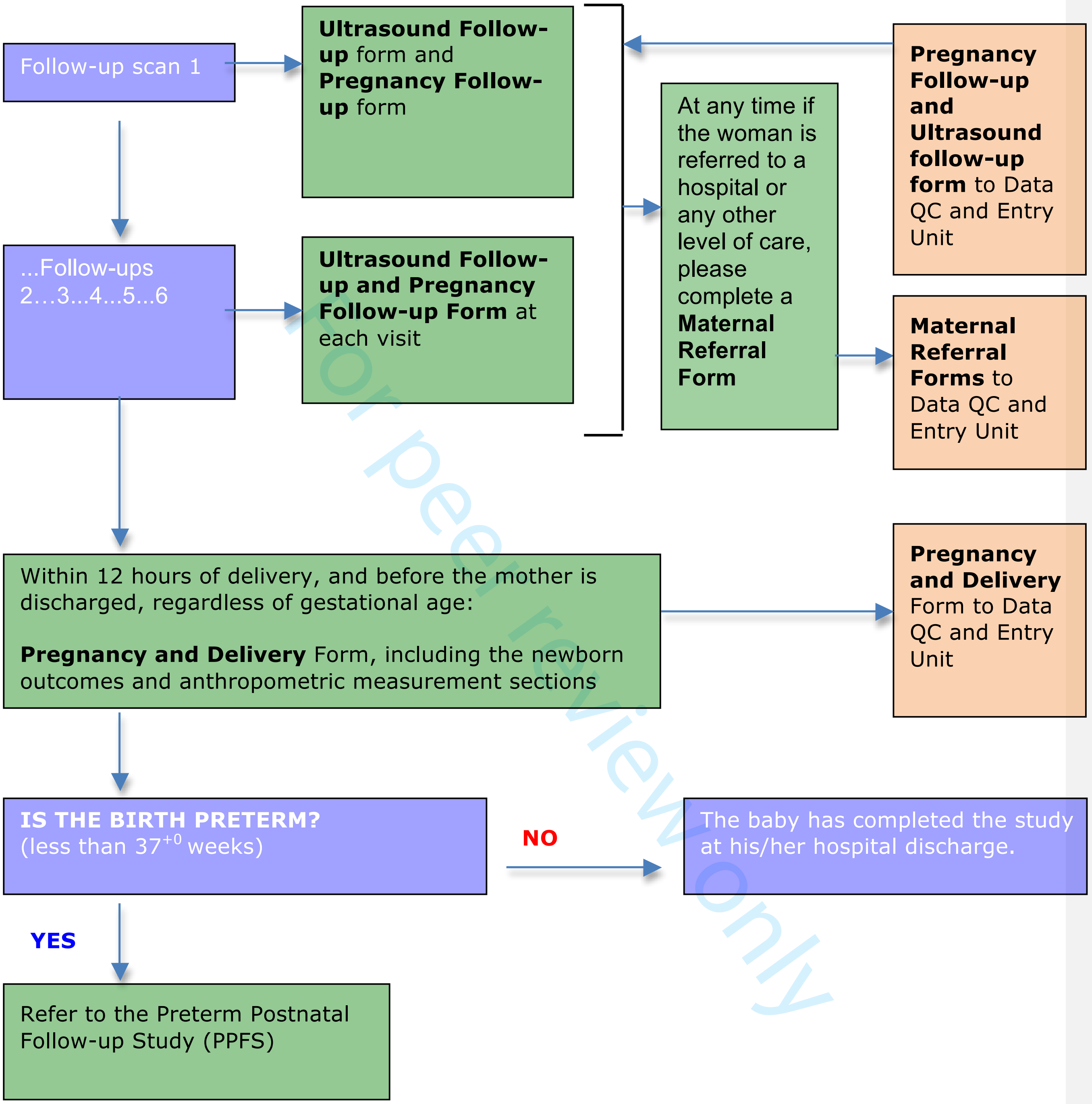

For peer review only

PRETERM POSTNATAL FOLLOW-UP STUDY DATA FLOW

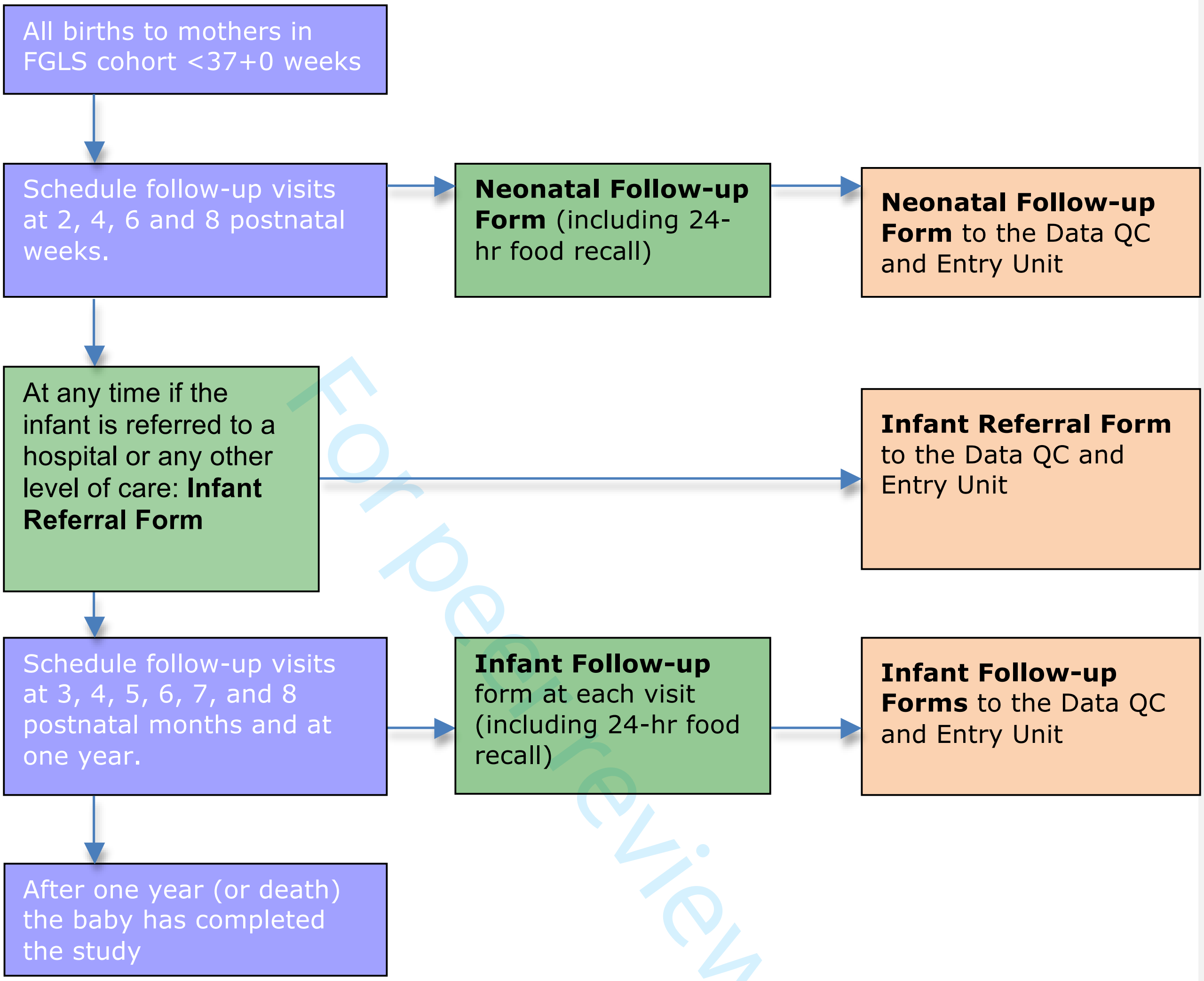

For peer review only

Appendix C: Ultrasound Methods And Quality Control

Acknowledgements: The first version of the ultrasound protocol was prepared between 18th June and 10th July 2008 by Aris Papageorghiou and Laurent Salomon, with valuable input from Jan van den Broeck. Aris Papageorghiou revised the protocol based on feedback from the INTERGROWTH-21st Executive Committee and Paul Chamberlain. Further amendments were then made based on a series of exchanges between Aris Papageorghiou and Laurent Salomon, Jan van den Broeck (quality control), Paul Chamberlain, José Villar and Stephen Kennedy. The protocol was finalized after extensive discussion at the initial meeting of the Steering Committee, which took place in Oxford on 15-17th September 2008, and finally approved in the Steering Committee Meeting in Oxford, 25-27th March 2009.

1. Background

A central plank of INTERGROWTH-21st is the creation of fetal growth charts during the FGLS part of the study. The correct use of ultrasound is essential to ensure that accurate, reproducible and applicable results are obtained. This document sets out all the ultrasound requirements for the study, including measurements, training, equipment and quality control.

2.1 Initial ultrasound examination and dating:

All mothers will have a trans-abdominal ultrasound scan between 9^{+0} to 14^{+0} weeks. This will be performed to rule out ectopic pregnancy, missed abortion and multiple gestations and to estimate gestational age (GA).

For the purposes of the study, gestational age will be calculated by LMP and confirmed by fetal crown-rump length (CRL). In order to allow accurate assessment of gestation the inclusion criteria used were:

- Certain LMP
- Regular 26-30 day menstrual cycles in the last 3 months;
- No hormonal contraception use, pregnancy or breastfeeding in the 3 months preceding the LMP.
- As embryonic growth may be different in pregnancies from assisted reproductive techniques[2,3] only those with spontaneous conception were included
- The CRL will be plotted on well-established charts [4,5]. If the difference in gestational age estimation by CRL and LMP is ≤ 7 days we will consider the LMP to be reliable and take it as the true biological date. Women where the difference in gestational age between LMP and CRL is greater than 7 days will not be eligible.

2.2 Subsequent ultrasound examinations

Following the initial scan, women will have up to six scheduled scans at ~5 weekly (±1 week) intervals until delivery. When rescanning does not occur within the allotted time, the women will be asked to attend at their next scheduled study appointment.

Essential documentation

At all examinations after dating the following will be documented:

1. Fetal presentation (cephalic, Breech, Transverse, Oblique)
2. Placental localization (fundal, high anterior, high posterior, high right lateral, high left lateral, low anterior, low posterior, low right lateral, low left lateral).
3. Amniotic fluid volume (polyhydramnios, increased, normal, reduced, oligohydramnios, anhydramnios).

For peer review only - http://bmjopen.bmj.com/site/about/guidelines.xhtml

Essential measurements (see* 3. Methodology of ultrasound and definitions*)

At all examinations after dating the following measurements will be taken:

1. Biparietal diameter (BPD)
2. Occipito-Frontal Diameter (OFD)
3. Head circumference (HC) using the ellipse facility
4. Transverse abdominal diameter (TAD)
5. Antero-posterior abdominal diameter (APAD)
6. Abdominal circumference (AC) using the ellipse facility
7. Femur length (FL)

Optional measurements

This study presents a unique opportunity to obtain other measurements of fetal biometry. However, the introduction of extra tasks could reduce the overall quality of the seven main measurements and inconvenience the participants. Therefore, the feasibility of taking additional measurements will be evaluated in consultation with each centre before a final decision is made and only some centres are expected to participate in these additional studies. In descending order of importance:

1. Humerus
2. Radius / Ulna
3. Tibia / Fibula

2.5 3-Dimensional Ultrasound Volumes (3D ultrasound)

Essential 3D measurements

The objective of the 3D component is to provide volumetric data to assess the quality of the 2D measurements (BPD, OFD, HC, AC, FL). Hence, the 3D volumes that we obtain will be of the:

(1) head (capture 3D volume with 2D view at the level of the BPD)
(2) abdomen (volume at the level of the AC)
(3) femur (volume in the same view as measuring the FL)

Optional 3D measurements

In order to establish a unique “biological databank” for future research additional 3D volumes of other organs, e.g. the heart at the level of the 4-chamber view of the heart; placental volumes, can be taken specifically for these purposes. These will be considered ancillary studies and will be dependent upon local capacity and the approval of the Steering Committee.

We plan to take the final decision regarding the amount of 3D data to be collected and the number of participating sites for these ancillary studies after conducting a pilot study assessing the human resources available at the centres, the need for additional training, time that 3D scanning adds to each visit, equipment considerations and the feasibility of transferring the very large datasets to the Coordinating Unit in Oxford.

Issues that we will explore in the pilot study include:
a) difficulties obtaining liver, brain and fetal cerebellar volumes
b) the need to obtain at least two good volume measures from the areas of interest, which may require 20-30 mins additional scanning
c) the temptation to divert attention away from standard measurements to more “exciting new technology” which will be popular with women. For these purposes the surface rendering function will be disabled.
d) how to save and transmit the volumetric data reliably given the risks of a hard drive crashing or being corrupted.
e) With advancing gestation (especially in the third trimester, it is likely that we will not be able to record many of the 5 volumes listed above accurately (i.e. brain and abdomen).

For peer review only - http://bmjopen.bmj.com/site/about/guidelines.xhtml

3. Methodology of ultrasound and definitions

The first visit (dating scan) will be between 9^{+0} and 14^{+0} completed weeks. The aim of this scan is to confirm fetal viability, and to exclude multiple pregnancy or major fetal abnormality. Fetal CRL is measured at this visit. In women who meet the inclusion criteria and where the discrepancy between gestational age by LMP and CRL is ≤7 days, subsequent visits (for fetal biometry) will be scheduled at approximately 5 weekly (± 1 week) intervals (i.e. 14-18, 19-23, 24-28, 29-33, and 34-38 weeks and 39-42 weeks).

A full morphological evaluation will be conducted at 19-23 weeks following standard practice at each centre. Fetuses diagnosed with any minor abnormalities will be managed as per local clinical guidelines. If the clinical decision is to continue with the pregnancy the mother will remain in the study. Study follow-up for these cases will be identical, but eventual exclusion at the time of data analysis will be carried out.

Fetuses with major abnormalities that may affect morphometric measurements will be excluded from further study. All infants will receive anthropometrical assessment after delivery

3.1 Basic characteristics

1. Transabdominal ultrasound
2. Lateral recumbent position.
3. Essential ultrasound measurements are obtained at all visits
    - Biparietal diameter (BPD)
    - Occipito-Frontal Diameter (OFD)
    - Calculated Head circumference (HC) using the ellipse facility
    - Transverse abdominal diameter (TAD)
    - Anterio-posterior abdominal diameter (APAD)
    - Calculated abdominal circumference (AC) using the ellipse facility
    - Femur length (FL)
4. Measurements are obtained 3 times from 3 separately generated ultrasound images of each structure in a blinded fashion (no measurement visible) and submitted electronically.
5. Following this the final set of measurements are revealed for clinical management purposes as per local protocols.
6. In each view a single 3D volume is stored for quality control purposes.
7. Ultrasound equipment provides measurements to tenth of a millimetre.
8. Data are submitted electronically to the web-based data set
9. Depending on the availability of appropriate technology, associated images are also submitted electronically to the Coordinating Unit. If real time submission of images is not available these will be down-loaded onto a memory card and submitted via email.
10. Ultrasound images of CRL, BPD, OFD, HC, AC and FL must fill at least 30% of the monitor screen.

3.2 Measurement techniques for compulsory measurements [4-10]

Crown – Rump Length (CRL) – only at the dating scan (9^{+0} to 14^{+0} weeks)

Obtaining the image

- A mid-sagittal section of the fetus should be obtained
- The fetus should be horizontal (at 90° to the angle of insonation)
- The fetus should be in a neutral position (not hyperextended or flexed)

Magnification

- The image must fill at least 30% of the monitor screen.

For peer review only - http://bmjopen.bmj.com/site/about/guidelines.xhtml

Caliper placement

- The intersection of the calipers should be placed on the outer borders of the head and rump.

Biparietal Diameter

Obtaining the image

- A cross-sectional view of the fetal head at the level of the thalami
- As close as possible to the horizontal with the angle of insonation as close as possible to 90°
- Oval shape
- Symmetrical
- Centrally positioned, continuous midline echo (falx cerebri) broken anteriorly at one third of its length by the cavum septum pellucidum
- The thalami should be located symmetrically on each side of the midline falx.

Magnification

- The cross section of the fetal head must fill at least 30% of the monitor.

Caliper placement

- The intersection of the calipers should be placed on the outer border of the parietal bones ('outer to outer') at the widest part of the skull.

Occipito-frontal diameter (OFD) and Head Circumference (HC)

Obtaining the image and magnification: obtained from the same still image as the BPD.

Caliper placement

The intersection of the calipers should be placed on the outer border of the occipital and frontal edges of the skull at the point of the midline ('outer to outer') across the longest part of the skull. The HC will be calculated from the BPD and OFD measurements using the ellipse facility (using the formula HC = π(BPD + OFD)/2).

Abdominal Circumference

Obtaining the image

- Transverse section of the fetal abdomen as close as possible to circular
- Umbilical vein in its anterior third
- Stomach bubble visible
- Kidneys and bladder not visible

Magnification

- The cross section of the fetal abdomen must fill at least 30% of the monitor screen.

Caliper placement

- The anterior-posterior abdominal diameter (APAD) and transverse abdominal diameters (TAD) are measured.
- To measure the APAD the intersection of the calipers is placed on the outer borders of the body outline from the posterior aspect (skin covering the spine) to the anterior abdominal wall.
- To measure the TAD the intersection of the calipers is placed on the outer borders of the body outline at 90° to the APAD, across the abdomen at the widest point.
- The AC will be calculated from the APAD and TAD measurements using the ellipse facility (based on the formula AC = π(APAD + TAD)/2).

Femur Length Technique

Obtaining the image

- To be imaged as close as possible to the horizontal plane.
- Angle of insonation of the ultrasound beam is 90°.

For peer review only - http://bmjopen.bmj.com/site/about/guidelines.xhtml

- The full length of the bone is visualised
- Not obscured by shadowing from adjacent bony parts.

Magnification

- The cross section of the fetal abdomen must fill at least 30% of the monitor.

Caliper placement

- The intersection of the calipers is placed on the outer borders of the edges of the femoral bone 'outer to outer').
- The trochanter is not to be measured.

3.3 Techniques for optional measurements:
The humerus, radius, ulna, tibia and fibula can all be measured from 14^{+0} weeks onwards. Similar to the guidelines of femur length measurement, the longest length of each bone is measured with the bone at as close as possible to 90° to the ultrasound beam. The humerus is measured from upper to lower lateral margins. In the forearm the ulna is distinguished from the radius by its longer proximal length (both radius and ulna end at the same level distally). In the lower leg the lateral bone is the fibula with the tibia lying medially to it. All long bones are to be measured along their longest lengths from upper to lower lateral margins. The bone measured should fill at least 30% of the width of the screen. **Only one side (the anterior or most easily accessible limb) should be measured.**

3.4 Inability to take measurements
The fetal position may on occasion be so persistently unfavourable as not to allow measurements according to the guidelines above.

CRL: Accurate measurement of CRL is of obvious importance in ensuring that a woman is eligible to take part in the study. If despite repeated attempts it is not possible to obtain a CRL measurement that allows confirmation of gestational age the woman is not eligible for the study (in the same way she will not be eligible for the study if the gestation calculated from CRL and LMP is discrepant by more than 7 days).

Essential measurements: With the exception of CRL, every effort should be made to obtain the best possible measurements taking into account the definitions above. This may require allowing the woman to go and return for the scan at a later date/time (within one week). Measurements should not be taken if it remains impossible to obtain a good quality image to allow accurate measurement this should not be taken. The next appointment should be kept as scheduled.

Optional measurements: The same principle applies to the optional measurements: these should not be taken if it is impossible to obtain a good quality image.

4. Equipment (based on RCR recommendations)[13]:
In all applications of ultrasound, three things are of prime importance: image clarity, resolution and the ability to differentiate tissue structures. Hence, although it is accepted that the best possible images may not be obtained from all patients at all times, the equipment chosen must be capable of visualizing tissue structures in the overwhelming majority of patients with different body sizes and shapes to a level sufficient to meet the exacting needs of the study.

The specifications below are the minimum requirements for the study, but it is important to recognize that image clarity and resolution may differ between manufacturers despite machines having similar specifications. It is therefore essential that candidate machines are tested before a final choice is made to assess their quality and to determine operator preferences. It is envisaged that this will be done at the Study Coordinating Unit (Oxford) or the Ultrasound Coordinating Unit (St George¹s, London).

For peer review only - http://bmjopen.bmj.com/site/about/guidelines.xhtml

We realize that there is a potential trade-off between quality and cost and, therefore, both these factors need to be considered in a balanced way in making a decision.

Study specific considerations

1. All ultrasound equipment to be used will be standardized
   - Uniform probes
   - Uniform factory presets ("study preset")
2. Commercially available high quality real-time ultrasound scanner.
3. Less than 2 years old.
4. Transabdominal probes suitable for scanning throughout pregnancy.
5. Facility for on-line transfer of measurements and associated images.
6. Facility to "blind" measurements from examiner until after data transfer.
7. Facility to "unblind" the final measurement to allow clinical use.
8. Equipment will be serviced periodically by specialized technicians as per the manufacturer's recommendations.

For peer review only

Operation/ Display

General requirements:

- Operating from a single phase standard domestic 240V, 13A, 50 Hz mains supply (110V in Cuba)
- Large single high resolution non-interlaced monitor (min.15")
- Display examination details
  Basic patient ID
  date & time
  examination centre
  selected probe
  acoustic power setting
  mechanical and thermal indices and other image processing and Doppler information to be shown in all display modes on all video/disk/hardcopy outputs
- Floating keyboard and monitor with easy to move console
- Single and dual display of the modes should be available simultaneously
- High definition variable size display magnification
- Digital Processing Channels Beamformer
- Digital display memory
- Large Dynamic Range
- Automated 'Tissue Specific' pre-sets
- Automated 'Tissue Specific' signal processing
- Ability to store reasonable number of images
  The number of images that can be stored must be stated.
- Large Cineloop
- Extended signal processing facilities
- Customizable pre-sets and calculations, for individual users and for different types of applications, for all modes
- Automated 'INTERGROWTH-21st Study" pre-sets
- Full DICOM-3 image and Cineloop transfer activated
- Hard Disk storage
- CD rewriter or DVD rewriter for backup of presets, configuration and archiving of studies in DICOM format
- DVD ±R(W) Drive
- > 100GB on board archive
- S video or composite video out (ideally all of the following: RGB, composite, S video, VGA and DVI)
- Ability to save images / cineloops in jpeg / avi format to external media for teaching purposes and ideally the ability to store images / cine loops to USB key
- On screen measurement of distance, area and circumference

OPTIONAL **3D / 4D capability**

(using ellipse or tracing methods), where a minimum of four simultaneous distance measurements or one area/ circumference measurement is required

- High definition B-mode
  - Controls of transmit power, receive gain (including TGC) and dynamic compression
  - Dynamic transmit and receive focusing with user selection of focal zones
  - Configurable spatial/temporal averaging
  - Measurement tools such as distance, area, circumference and 2D-area (ellipse or tracing)

Scanheads

- The scanner should have, at least 2 active transducer ports from which one can be selected from the control panel.
- Broad band 2D probe which must be suitable for examinations at 9-14 weeks, 21-23 week and third trimester growth scans and with a penetration suitable for a wide variety of patients.
  E.g. Broadband 3-6 MHz and broadband 5-9 MHz probes or a single broadband probe of 3-9 MHz

Accessories

- Thermal printer capable of printing onto continuous standard thermal paper
- DVD player/recorder

General Configuration

- The unit should be compact and provide reasonable portability.

Safety

- The unit should meet or exceed performance and safety requirements of UL 544, CSA C22.2 and IEC 60601-1 and be CE marked

Maintenance

- In order to ensure the equipment is maintained in proper working order, the basic maintenance requirements are listed below. Suppliers are required to specify the services they will provide and their costs:
  - Clear instructions on the regular maintenance that should be carried out by the user
  - If the Unit needs regular maintenance, the frequency with which any parts need replacing
  - Engineer call-out response time of 48 hours following initial assessment/advice from the hospital's maintenance team
  - Options available if the equipment is out of action for an extended period
  - Details of any maintenance contract(s) available to cover all normal device deterioration and emergency repairs
- The maintenance support should be such that there is automatic supply upgrade of new firmware

For peer review only

- 3D Broadband 3-6 MHz or 3D broadband 5-9 MHz probes
- Spatial Temporal Image Correlation (STIC) – option – capture full fetal heart cycle in real time using volumetric transducer
- Surface rendering **disabled**
- Volume Contrast Imaging
- Specific software for post-processing stored 3D volumes.

HD Flow imaging (bidirectional Power Doppler), Tissue Doppler, Spectral Doppler, Colour velocity mapping and Power Doppler mapping

- Controls of power, gain, velocity range, baseline shift, low pass filter and compression
- Control for display time base
- Manual and automatic spectral analysis and waveform index estimator
- Volume flow measurement, User defined calculations
- Good sensitivity, temporal and velocity resolutions
- Acceptable Range gate registration accuracy, Gate duration, Beam width, Penetration depth, Velocity, Direction indication, Directional discrimination, Velocity estimation accuracy, High-pass filter, Waveform index estimation accuracy, Volume flow estimation accuracy
- Acceptable Colour direction indication, Lowest detectable velocity, Highest detectable velocity, Image spatial resolution (axial, lateral and slice thickness), Temporal resolution, Velocity resolution, Tissue colour suppression, Angle dependence of colour, Registration colour/B-scan image, Penetration depth

Further Notes

- Any non-compliance with the specification must be clearly identified together with any alternative or additional features.
- A decision may be taken to lease the selected device. If so, a tendering exercise will be undertaken to choose an appropriate leasing company.
- The availability of any training including courses offered / recommended by the manufacturer / supplier should be stated, together with any associated costs that may be incurred. Preference will be given to manufacturers / suppliers offering free fault finding, preventative maintenance, and first line maintenance training courses to two members of each participating centre's maintenance team.
- The cost of any contracts available for the regular service and/or maintenance of the devices should be separately stated.
- Prices should include any discounts or special offers available, including those of any ex-demonstration units that may be available, but these should be separately detailed.
- The terms of guarantee must be stated.

5. Quality Control

Quality assurance and control of the Ultrasound component of INTERGROWTH-21st will be the remit of an Ultrasound Quality Unit (USQU) (see section 6 below).

In order to ensure ongoing quality control we will employ rigorous tools for training, assessment and certification under the supervision of qualified instructors. Intra-observer and inter-observer measurement errors will be assessed during the training course before initiating the study.

Ongoing quality control and adherence to protocols will be performed. This will include quality assessment of images, random evaluation and repetition of ultrasound measurements, and assessment of collected data. It is envisaged that a limited number of

For peer review only - http://bmjopen.bmj.com/site/about/guidelines.xhtml

experienced obstetric ultrasonographers will form the research teams at each study site. They will be provided with standardized equipment and the ultrasound protocol describing all measurement techniques, protocols and procedures for training and clinical use.

5.1 Initial training and standardization of all study 2D-ultrasonographers against an international lead ultrasonographer, and assessment of local conditions

5.1.1 Initial training, assessment and standardization of ultrasonographers

It is recognized that the local ultrasonographers will already have a high standard of training. Therefore, the goals of initial training are:

- To ensure all ultrasonographers are familiar with equipment to be used in the study, including the ultrasound machine, the SOPs, the automated recording of ultrasound measurement values, software and entry of data.
- Ensuring standardization by training, assessment and certification. The schedule for this is as follows:
  1. Individual and group theoretical training (Coordinating Unit)
  2. Hands-on ultrasound training (Coordinating Unit)
  3. Submission of a log of 10 measurements of each parameter. These measurements will be repeated three times from three different images (to assess intra-observer variability), and repeated offline by the external sonographer (to assess inter-observer variability):
  4. Measurements should, on average, score >75% of the max score (i.e. 4 and 3 respectively, see below (5.2.1)
  5. No more that one out of the ten repeated measurement by the same operator should vary of more than two standard deviations (SD) of the measurement error of each given parameter at the given GA.
  6. No more than two out of the ten repeated measurements by a different operator should vary by more than two SD (proxy for random error) and there should not be a systematic bias of more than 0.5 SD.
  7. If these criteria are not fulfilled, certification will not be given.
  8. If the criteria are fulfilled certification of competence will be given prior to the start of the study

5.1.2 Pilot test-retest study

Similar to the anthropometry component of the study, quality control of ultrasound measurements will be primarily based on the comparison of repeat measurements by the same or different observers. Routine data collection will be guided by a system of maximum allowable differences between replicates. However, in contrast to anthropometry, for ultrasound measurements not much is known about what the maximum allowable differences should be for the different fetal measures at different stages of pregnancy. It is not known how exactly fetal size and GA influence measurement error in ultrasound. In addition, it is not clear how much measurement error in ultrasound is influenced by expertise and how expert-level and non-expert-level measurement errors compare. A pilot test-test study is therefore proposed to determine the Technical Errors of Measurement at both levels of expertise and trends to bias (in comparison with expert), specifically for:

- CRL between 9^{+0}-14^{+0} weeks
- BPD, HC, FL, AC in the different GA intervals that will be used in the study:14-18, 19-23, 24-28, 29-33, 34-38 and 39-42 weeks

This pilot study will be carried out in the unit at Oxford. It will involve one or several other obstetricians who regularly do ultrasound measurements in the same hospital. All observers should be familiar with the measurement protocol of the study and should have received at least one training session by the LU prior to the start of the pilot study. The same equipment must be used as the one that will be used in the main study. For each of the seven GA

For peer review only - http://bmjopen.bmj.com/site/about/guidelines.xhtml

intervals (from 9^{+0}-14^{+0} to 39-42 weeks) a number of 10 or 15 women will be enrolled over a 2-month period (November – December 2008).

It is proposed that each woman undergoes 4 independent assessments in a random order, two assessments by the LU and two by another ultrasonographer in the hospital. Each assessment will consist of an ultrasound session during which all the measurements needed for the GA interval will be performed in a random sequence. For each measurement a number of metadata (factors potentially modifying measurement error) could be collected so that the pilot study of itself becomes more publishable. All observers should ideally be blinded to the measurement values they obtain by automatic transfer of values to the online system.

This pilot study will yield GA-specific Technical Errors of Measurement and other statistics of inter- and intra-observer reliability and bias. These, in turn, will be used to define the maximum allowable differences for each fetal measure at each age that will be used to: (1) evaluate success of initial training (2) identify the need for re-measurement in the routine data collection, and (3) interpret data quality statistics from the standardization exercises and the random re-measurements.

5.1.3 Assessing local factors: the initial site visit

Prior to the start of the study, an initial site visit will be performed by a member of the USQU. The aims are to ensure:

- Preparation of the local data quality control activities, which will involve
    - Identification of a local supervisor of ultrasound data quality control activities. This will often be the ultrasonographer with the best data quality statistics and closest in performance to the LU during the initial LU visit. The person will have good organizational and team working skills and be competent in using the required software. The local supervisor should be instructed and trained to do the following tasks:
        - Conduct refresher sessions every two months, and document observer performances
        - Conduct data quality control re-measurements on a random sample of routine measurements
        - Observe as many routine measurements as possible performed by all site ultrasonographers
        - Extract and analyze, in collaboration with a LU, data from routine standardization sessions and from random re-measurements done at the site.
        - Liaise with LU about problems with ultrasonographers, SOPs and data quality statistics
    - Identification of a local data management coordinator who will liaise with the overall study coordinator.

5.2 Quality control of measurements in the FGLS: Monitoring and feedback of performances of ultrasonographers and identification of needs for retraining

5.2.1 Qualitative quality control

All measurements taken by ultrasonographers will be qualitatively controlled and scored by the Research Fellow.

Scores will be given according to the described scheme by *Salomon et al*[11]. Each specific criterion scores one point; thus, the maximum score is 6 points for BPD/HC; 6 points for AC; and 4 points for FL.
Any ultrasonographer with more that 10% of images rejected in a given period will have his/her certification withdrawn and will undergo re-training.

Objective scoring system for still images (modified from Salomon et al[11]*)*

BPD/ OFD/ HC	*AC*	*FL*
Symmetrical plane	Circular plane	Both ends of the bone clearly visible
Plane showing thalami	Image shows the stomach bubble	<45◦ angle to the horizontal
Cavum septum pellucidum 1/3 along midline echo	Image shows umbilical vein along 1/3 of the abdomen	Femoral plane occupying at least 30% of the total image size
Cerebellum not visible	Kidneys not visible	Calipers placed correctly
Fetal head occupies at least 30% of the total image size	Abdomen occupies at least 30% of the total image size	
Calipers and dotted ellipse placed correctly	Calipers and dotted ellipse placed correctly	

In case of clearly incorrect caliper placement, images will be rejected and measurement excluded. If the image scores half or less of the maximum score (i.e. 3 points or 2 points, respectively), measurements will also be excluded.

5.2.2 Quantitative quality control: Intra-observer reliability

Intra-observer reliability will be prospectively assessed based on the three concealed measurements taken routinely. No more that **one out of the ten** repeated measurement by the same operator should vary by more than two SD of the given parameter at the given GA.

If this is not fulfilled, certification is cancelled and the sonographer identified as requiring re-training. Feedback and discussion between USQU and site supervisors will follow in order to highlight the need for re-training and to carry out re-training.

5.2.3 Quantitative quality control: Random re-measurement on 2D images

In order to assess correct caliper placement, a random sample of 10% of all measurements will be re-measured on still images by the Clinical Research Fellow / LU. This will allow production and evaluation of inter-observer reliability and bias (against the LU) for each observer based on the random re-measurements. No more than **one out of the ten** repeated measurement by a different operator should vary by more than two SD and there should not be a systematic bias of more than 0.5 SD.

If this is not fulfilled, certification is cancelled and the sonographer identified as requiring re-training. Feedback and discussion between USQU and site supervisors will follow in order to highlight the need for re-training and to carry out re-training.

5.2.4 Quantitative quality control: Random use of 3D images to re-measure planes

In order to assess correct image acquisition, a random sample of 5% of all 3D volumes will be used to acquire the ideal measurement plane by the Clinical Research Fellow / LU. This will allow evaluation of inter-observer reliability and bias (against the LU) for each observer based on the random re-measurements. No more than **two out of the ten** repeated measurement by a different operator should vary by more than two SD (proxy for random error) and there should not be a systematic bias of more than 0.5 SD.

For peer review only - http://bmjopen.bmj.com/site/about/guidelines.xhtml

If this is not fulfilled, certification is cancelled and the sonographer identified as requiring re-training. Feedback and discussion between USQU and site supervisors will follow in order to highlight the need for re-training and to carry out re-training.

5.2.5 Site standardization exercises.

Local test-retest exercises involving all site observers are an opportunity for refreshing the understanding of the protocol, better focus on the protocol, re-training and better comparison of observers as each observer measures the same subjects, which is not the case for routine measurements and for the random quality control re-measurements.

- Organize periodic production and evaluation of data quality statistics for each observer based on the measurements taken during the standardization sessions
- Feedback and discussion between USQU and site supervisors about data quality statistics from the standardization sessions; identification of need for re-training; organize and carry out re-training
- These exercises will be done on 10 patients every 6 months, or more frequently depending on UQDU recommendations

5.3 Optimizing the data systems to accommodate the needs for quality control

The data entry system should allow incorporating measurement values and data from quality control and standardization exercises and be able to identify these as such.

- The data system could select a random sample of non-supervisor routine measurements for QC re-measurement. Ultrasonographers will be unaware which measurements will be re-measured when they do their measurements.
- Data extraction routine for monitoring of data quality statistics of entire study, sites, and individual ultrasonographers
- Establishment of a research dataset on determinants of ultrasound data quality, to be extracted from the main database.
    - Possible factors causing variability in ultrasound measurements with a given type of instrument may include, among others:
        - Parity
        - BMI
        - GA, size
        - Presentation / position of fetus
        - Sex
        - Liquor volume
        - Order of measurements
        - Observer factors
        - Measurement setting factors

For peer review only - http://bmjopen.bmj.com/site/about/guidelines.xhtml

5.4 Reporting, analysis and publication on ultrasound data quality

5.4.1 Central reporting

The USQU will produce 3-monthly data quality statistics based on standardization sessions, QC re-measurements and routine replicates. This may include, as appropriate the production of statistics and trend plots of:

- Intra-observer reliability of individual ultrasonographers
- Inter-observer reliability of individual ultrasonographers against LU
- Site bias against LU
- Site intra-observer reliability
- Site inter-observer reliability against supervisor
- Comparison among sites
- Proportion of failed checks on maximum allowable differences and on range checks

B. Publication plan

- Publications on ultrasonographic training and data quality will be integrated into the study's overall publication policy
- Possible topics include:
  - Paper on ultrasound data quality in INTERGROWTH 21st
  - Research questions using the research dataset on ultrasound data quality
    - Determinants of observer bias in ultrasonography
    - Determinants of observer reliability in ultrasonography
    - Differences between concealed and revealed measurements
    - Difference between 2D and 3D measurements
    - Differences in data quality statistics obtained from training sessions, routine data collection, quality control, and standardization sessions

For peer review only

Figure: Summary of quality control

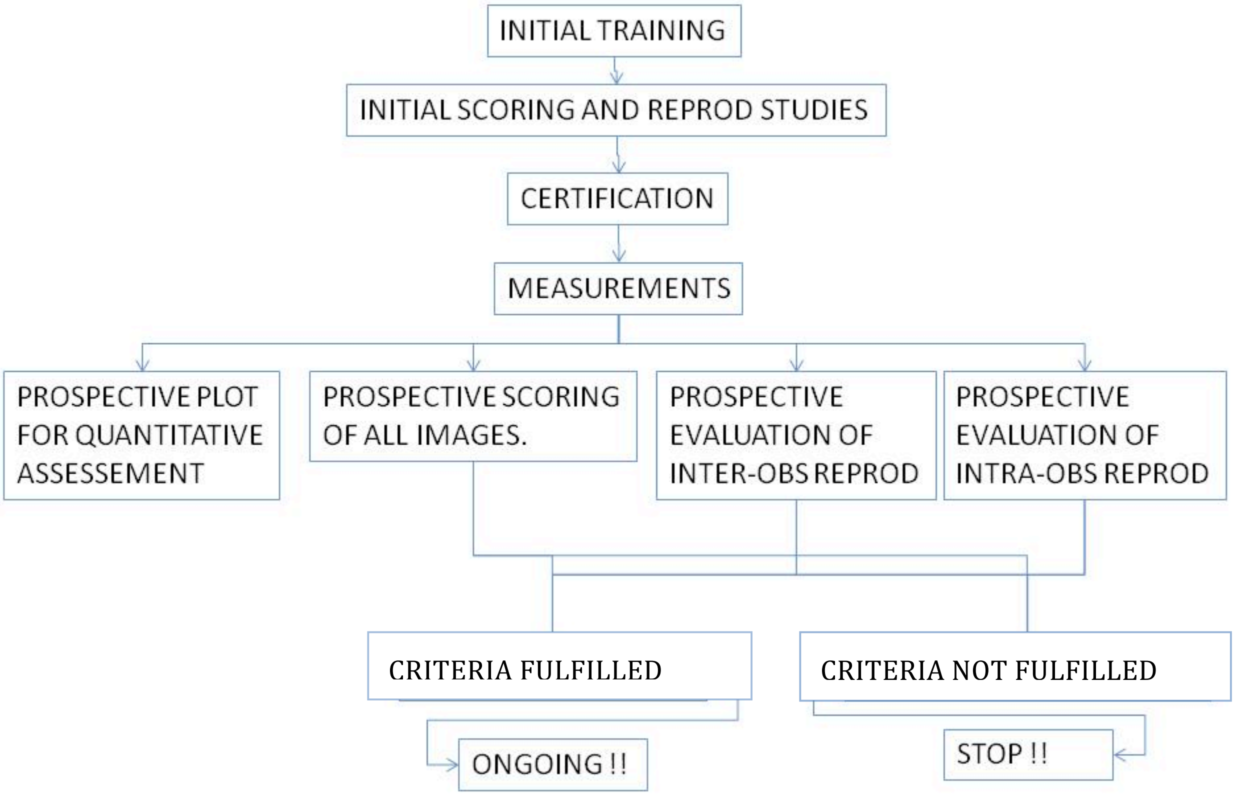

6. Ultrasound Data Quality Unit

Quality assurance and control of the Ultrasound component of INTERGROWTH-21st will be the remit of a Ultrasound Quality Unit (USQU). USQU will be coordinated by Aris Papageorghiou who will be assisted by External Ultrasound Experts (Laurent J Salomon and Shaida Zaidi) and a Clinical Research Fellow (Study protocol p.34). This unit will coordinate with the anthropometric quality control unit and both will follow similar protocols.

6.1 Proposed activities of the USQU

The USQU will be responsible for:

1. Development of standard operating procedures (SOP)
2. Initial training, assessment and certification of ultrasonographers
3. Standardizing all study 2D-ultrasonographers against a lead ultrasonographer (LU)
4. Monitoring and feedback of on-site standardization levels and performances of individual ultrasonographers; identification of needs for retraining
    a. Quality control of routine measurements
    b. Random sample of replicate measurements
    c. Site standardization exercises
5. Optimizing the data system to accommodate the needs for quality control
6. Reporting, analysis and publication on ultrasound data quality
    a. Central reporting
    b. Establishment of an analysis dataset on ultrasound data quality, to be extracted from the main database
    c. Publication plan

6.2 Ultrasound protocol and standard operating procedures (SOP).

This SOP includes procedural guidelines on:

- Patient selection
- Preparation of subject for measurement
- Sequence of measures and replicates; blinding to previous measurement values
- Measurement technique
    - Positioning of subject
    - Handling of caliper, identification of planes and landmarks, marking distances, recording and transfer of results
    - Entry of measurement metadata
- How to deal with special fetal positions and other unusual circumstances; circumstances under which one should delay or forego the assessment or part of the assessment;
- Release of measurement values and GA calculations for clinical purposes
- Preparation of measurement setting and instruments; linkage with computer and database
- Ultrasound device and accessories: maintenance, transport, storage, calibration checks

6.3 Unanticipated changes to this protocol / SOP

Despite all efforts, it is likely to be impossible to anticipate all possible eventualities in a study of this size. Therefore, changes to this protocol will be possible. These will be proposed to the Steering Committee by the USQU, and incorporated if approved.

For peer review only

References

1. The International Fetal and Newborn Growth Consortium for the 21st Century INTERGROWTH-21st Study Protocol. Oxford University, 2008

2. Cetin I, Cozzi V, Antonazzo P. Fetal development after assisted reproduction - a review. Placenta. 2003 Oct;24 Suppl B:S104-13

3. Jacob S, Moley KH. Gametes and embryo epigenetic reprogramming affect developmental outcome: implication for assisted reproductive technologies. Pediatr Res. 2005 Sep;58(3):437-46

4. Robinson HP. Sonar measurement of fetal crown-rump length as means of assessing maturity in first trimester of pregnancy. Br Med J. 1973 Oct 6;4(5883):28-31

5. Robinson HP, Fleming JE. A critical evaluation of sonar "crown-rump length" measurements. Br J Obstet Gynaecol 1975;82:702-10.

6. British Medical Ultrasound Society. Fetal size and dating: Charts recommended for clinical obstetric practice. February 2007 (Revised: January 2008)

7. Chitty LS, Altman DG, Henderson A, Campbell S. Charts of fetal size: 2. Head measurements. Br J Obstet Gynaecol 1994; 101: 35–43.

8. Chitty LS, Altman DG, Henderson A, Campbell S. Charts of fetal size: 3. Abdominal measurements. Br J Obstet Gynaecol 1994; 101: 125–131.

9. Chitty LS, Altman DG, Henderson A, Campbell S. Charts of fetal size: 4. Femur length. Br J Obstet Gynaecol 1994; 101:132–135.

10. Snijders RJM, Nicolaides KH. Fetal biometry at 14–40 weeks' gestation. Ultrasound Obstet Gynecol 1994; 4: 34–48.

11. Salomon LJ,. Bernard JP,. Duyme M, Doris B, Mas N, Ville Y. Feasibility and reproducibility of an image-scoring method for quality control of fetal biometry in the second trimester. Ultrasound Obstet Gynecol 2006; 27: 34–40

12. International Society of Ultrasound in Obstetrics & Gynecology Education Committee. Sonographic examination of the fetal central nervous system: guidelines for performing the 'basic examination' and the 'fetal neurosonogram'. Ultrasound Obstet Gynecol. 2007 Jan;29(1):109-16. Links

13. Royal College of Radiologists: Standards for ultrasound Equipment (2005)

For peer review only - http://bmjopen.bmj.com/site/about/guidelines.xhtml

Appendix D: Newborn, Preterm and Maternal Anthropometric Techniques, Equipment and Standardization and Data Quality Assurance and Control Plan.

Please see the Anthropometric Handbook.

For peer review only

Appendix E: Data Management: the Electronic System

The data management process will be performed electronically, simultaneously with data collection, at each study site. Data entry will be implemented on a customized web-based system developed for the study. The system automatically carries out range and consistency checks for immediate correction and reviews percentages of missing/unknown values for each variable. This technology also allows a real-time audit of each site by a set of pre-defined reports included in the system (recruitment, data inconsistencies, etc) run periodically by the Project Data Manager. Additional modules for internal/external messenger, hosting of study documentation (protocol, guidelines, etc) and user administrator make up an integral data management solution for multicentre studies.

The INTERGROWTH-21st website homepage will consist of an open part and a part protected by username and password. The open part will be used for publication of information, news etc. The protected part will contain the data entry application, a reporting module, an alert module, a descriptive statistics module, and an administration module. We are looking into the possibility of linking the application to the external ultrasound data sources.

Ultrasound measurements obtained during the follow-up visits will be electronically transferred from the ultrasound equipment to the web-based system to avoid transcription errors using a database format, agreed between ultrasound equipment manufacturer and system development group. Routine analyses will be carried out regularly for each variable to check digit preference and unusual frequencies of answers that may reflect poor understanding of the instructions. After the information is electronically transferred, the last measured will be made available to the attending staff for clinical use. The application includes an automated user and event logging system.

Data collected will be evaluated by the Data Management Unit for further quality control analyses and compliance with the study protocol, including the analysis of eligibility criteria, the timing of visits and drop-out rates. Measurements for the study taken from the equipment will have a facility to blind all fetal measurement values until they are submitted for the study. Following submission the last value will be printed for use in clinical care.

All other clinical and anthropometric data will be collected initially in a paper form entered onto the on-line system at each local institution by specially trained data management staff. Data should be entered into the system within one week of being collected facilitating the retrieval of possible missing data or other inconsistencies.

For peer review only

Appendix F: Policy and determination of responsibilities for INTERGROWTH-21st

1. Study Coordination and Management

The study's coordination and management, including data management and analyses, are the primary responsibility of the Oxford Maternal & Perinatal Health Institute (OMPHI) within the Nuffield Department of Obstetrics & Gynaecology, University of Oxford, which will act as the study Coordinating Unit (CU). The Maternal Health Task Force at the Harvard School of Public Health will collaborate with OMPHI in the study's overall implementation.

The study coordination and management will include the following activities:

1.1 Study Preparation

Coordination activities:

- Develop protocol with collaborators
- Conduct site visits for selection of study sites
- Selection of committee members and assign tasks to study committees
- Organize collaborators' meetings
- Establish communication procedures between sites and CU
- Administer funding base for coordination activities and study implementation
- Keep donor agencies informed about progress of study

Study materials:

- Prepare data collection forms and supporting documents
- Prepare operation manuals
- Prepare measurement and standardization protocols including Quality Assurance
- Select and purchase equipment
- Prepare data collection forms and other study materials as required

Data processing and management systems:

- Conduct feasibility study
- Negotiate and implement an electronic data management system
- Set-up system for on-line data entry and validation
- Define electronic monitoring reports to be produced
- Pilot data collection systems and monitoring reports

Statistical analysis:

- Develop statistical analysis plan

1.2 Study Implementation

Coordination activities:

- Assist study sites in local adaptation of study materials
- Submit protocol to Ethics Committees
- Distribute data collection forms and other study materials to centres
- Develop standard set(s) of slides for presentations about the study
- Establish training procedures for research staff

1.3 Study Conduct

Coordination activities:

- Coordinate study's day-to-day activities
- Monitor study progress through site visits and on-going review of data entry
- Communicate with local investigators; supporting units; Steering Committee (SC) and Advisory Committee; and other collaborators (e.g. UN agencies, donors)
- Prepare electronic study newsletter

For peer review only - http://bmjopen.bmj.com/site/about/guidelines.xhtml

- Convene regular meetings of staff at CU
- Organize coordination meetings of local Principal Investigators; Steering Committee; site visits; exchange of relevant staff between sites, etc.

Data management and statistical analysis:

- Monitor data collection procedures at study sites and regularly advise SC about all data received and processed
- Validate data and produce queries about inconsistent and/or apparently incorrect data
- Send data queries to study sites so that errors are corrected at collection phase
- Update master file using corrections from validation checks and/or answers to queries
- Evaluate electronic monitoring reports by study site based on recruitment rates, drop-outs, data completeness, quality assurance checks, etc.
- Maintain communication channels between study sites and supporting units relating to queries
- Inform SC whenever a study site requires retraining in data collection or is failing to respond to queries and implement corresponding actions
- Conduct interim statistical analyses of data in accordance with analysis plan approved by SC
- Conduct final statistical analyses of data in accordance with plan approved by SC
- In collaboration with SC, coordinate preparation of articles for publication in peer-reviewed, scientific journals etc.

2. Study Committees

The following Committees will oversee the implementation of the study:

2.1 Steering Committee (SC)

The SC will consist of the following:

- Professional staff from CU and its supporting units
- Principal Investigators (one per site)
- Selected senior scientists
- Representatives from Department of Nutrition and Department of Environmental Health, and other related Institutions as considered appropriate

The SC will meet regularly by conference call or face-to-face meetings to review the study's progress and discuss substantive issues that arise from the study's implementation and conduct. Its role is to make managerial decisions that affect the conduct of the study; to ensure the comparability of data from the different sites, and to resolve problems that arise in running this complex project.

The SC will have an **Executive Committee (EC)** that will meet frequently to review study progress and problems, protocol divergences, and other substantive issues that may arise from the study's implementation. The EC will have overall administrative responsibility for the study's implementation. All changes or alterations to the protocol or issues related to the technical, financial or administrative conduct of the study must be approved by the EC. This Committee will also select the study sites; approve the continuing participation of the sites selected, and determine which data are to be included in the pooled, international data set. Every effort will be made to achieve consensus for all decisions but where this is not possible resolution will be by simple majority with a quorum of at least 5 members. The EC membership will be JV, SK, AN, DA, MdO, ZB, AP, the SC Chair and a Principal Investigator selected by his/her peers. The Project Manager will be an ex-officio member.

For peer review only - http://bmjopen.bmj.com/site/about/guidelines.xhtml

2.2 Advisory Committee (AC)

- The AC will consist of senior scientists in the areas of Medicine, Epidemiology and Statistics, Obstetrics and Gynaecology, Paediatrics and Human Biology. The task of the AC will be to provide overall and strategic advice to the SC.

3. Data Management

Electronic data management will be contracted to Medscinet, a private company that has provided similar services in previous multicentre studies we have conducted. It has extensive experience of conducting large multicentre trials and observational studies, and it is recognized as a leading research organization in reproductive health. Although based in Sweden, the company has a UK base in the Department of Women's Health, St Thomas' Hospital, London. It will provide a full on-line data management system, as well as supervision and training support to the participating sites for the purposes of data entry, cleaning, file preparation, and study monitoring. All participating sites will be required to adhere to the study's data management system, which will be available on the study web page. We have successfully used this system in two previous multicentre studies involving sites included in INTERGROWTH-21st.

4. Ancillary Studies

Proposals for ancillary studies from one or more local Principal Investigators will be considered as long as a) they do not result in alterations to the main protocol and b) they do not impact adversely on subject recruitment and/or participation in the main study. All ancillary studies and/or additional data collection must be submitted to the EC for written approval before implementation. A letter of intent must be submitted to the CU at the conceptual stage of the ancillary study. If approved by the EC, the local Principal Investigator(s) will be invited to submit a detailed proposal that should include the study's rationale, objective, methodology, expected outcomes and budget.

5. Site Monitoring

Study progress will be monitored by:

a) Monthly reports for each study site produced by the CU using data from the electronic data bank, which will be shared with the study sites and EC members.

b) Visits (the frequency as required by the progress of the study) to all study sites to ensure that the study is uniformly implemented (the timing to be decided by the CU in coordination with the EC and Anthropometric Quality Control Unit (ADQU). The activities to be performed during each visit include, but are not restricted to:

- Meeting site Principal Investigator and members of study staff
- Review of study procedures, use of data forms and related documents
- Observation of study personnel carrying out specific procedures
- Particular attention will be paid to standardization of data collection (e.g., adherence to study protocol, and measurement/standardization protocols)
- Review of coverage and participation rates, including characteristics of participants and non-participants, and reasons for loss to follow-up
- Observation of data validation procedures
- Physical walk-through of certain procedures (e.g., screening to determine subject eligibility or taking of informed consent)
- Conversations with key support personnel to assess their practice with regard to data collection
- Inspection of study facilities and subject study files

For peer review only

c) Exchange visits by field supervisors, regional and global meetings of field directors to facilitate standardization of procedures across sites.

6. Dissemination of Results, Data ownership, Publications and Authorship

6.1 Dissemination of Results

The results of the study will be disseminated as widely as possible, in publications and the mass media. Activities will include:

- Publication in peer-reviewed journals of all papers reporting primary and secondary outcomes based on the pooled international data set
- Publication of papers and editorials in regional journals that are published in local languages, such as Bulletin of PAHO (Spanish), Chinese, French or African Journals
- Electronic study newsletter
- Publication in periodicals from other collaborating institutions.
- Presentations at local, regional and international meetings
- Publication of a book including all collected information (e.g., growth curves) and detailed methodological issues unlikely to be included in reports to peer-reviewed journals. The Executive Committee in coordination with Local investigators will take responsibility for this task.

6.2 Data Ownership

All data derived from the INTERGROWTH-21st multicentre project will be the property of the Oxford Maternal and Perinatal Health Institute (OMPHI) at the University of Oxford. Individual investigators will share the ownership of their site-specific data sets with OMPHI. After publication of the growth curves and the main papers related to the secondary objectives, the pooled data set will enter the public domain at a time to be determined by the SC. The anonymity of all study participants will be ensured.

6.3 Publications and Authorship

All papers reporting the new growth charts (Primary Objective, components 1-3), based on the pooled international data set (the growth curves) will be published under corporate authorship (International Fetal and Newborn Growth Consortium) The address for correspondence will be the Oxford Maternal & Perinatal Health Institute (OMPHI), Nuffield Department of Obstetrics & Gynaecology, University of Oxford, John Radcliffe Hospital, Oxford OX3 9DU, UK.

For these publications, the names of the members of all committees and study units will be listed at the end of the paper (see Lancet 2006;367:1819 for an example of the format) plus their individual contributions to the study (e.g., protocol development, study coordination, data management, data analysis, manuscript preparation, etc) as required by the individual journal.

Sites participating in the multicentre project will be listed alphabetically by country; the local Principal Investigator will be responsible for selecting the authors within each country. It is understood that authorship, within each site, will be offered to those who have made a substantial contribution to the study. Within each site, the order of the author's names will be the responsibility of the local Principal Investigator. The SC will provide a list of people to be mentioned in the standard Acknowledgments, once it has been agreed what type of support will be acknowledged. All papers arising from the study should include such a list of acknowledgements. The University of Oxford will give certificates of collaboration to doctors, nurses and local staff who contributed to the study but whose names cannot appear in publications.

For peer review only - http://bmjopen.bmj.com/site/about/guidelines.xhtml

For all other papers arising from the study (except the main growth charts described above), including those relating to secondary objectives, a modified system of corporative authorship will be used, in which SC members will be listed by name according to an agreed rotation based on personal interests and leadership in the preparation of the paper. These names would be followed by "for the International Fetal and Newborn Growth Consortium" or similar statement as agreed by the SC. The authorship order will rotate based on a system agreed by the SC, i.e. initial lottery and rotation thereafter or some other system.

The study will have a **Publications Review Committee (PRC)** consisting of the EC members, a Local Principal Investigator and one or two *ad hoc* Senior Scientists invited according to specific needs. The PRC will be responsible for:

- Preparation of a list of expected publications on the primary and secondary outcomes of the study, with the authorship strategy for each set according to the rules above which are to be approved by the SC.
- Setting-up writing committees for the primary outcome, corporate authorship papers and a review mechanism by the SC of the final drafts that are submitted for publication.
- Reviewing and approving all papers sent for publication with the goal of maintaining internal consistency of material and methods, as well as authorship policies.
- Reviewing and approving all site-specific analyses proposed by local Principal Investigators.

The following more general rules will apply:

- Individual sites will not be allowed to publish analyses of country-specific data or subsets of sites that, in the EC's assessment, have the potential to be misconstrued as standard data for particular nations or population groups.

- However, individual sites and groups of sites are encouraged to publish other analyses of data based on their site-specific data. These analyses must be cleared by the EC before preparation. The order of author's names in publications based on site-specific analyses will be the responsibility of the local Principal Investigator. The EC will provide the Acknowledgments and funding sources, in a standard format.

- The CU is encouraged to publish methodological and conceptual papers that describe the methods and procedures used in the study. The order of authors' names for these publications will follow the modified corporative authorship model including the SC as well as the CU members that lead the preparation of the papers. The CU will provide the Acknowledgments in a standard format.

- Scientists are encouraged to present the study at scientific conferences and meetings. When scientists are invited to international or regional meetings to present the study, the CU should be informed and prior written approval is required with details of the type, venue and organizers of the meeting. The CU will keep an archive of all materials presented at meetings and make them available at the study's web page.

Press enquiries will be honoured unless there are some operational or scientific reasons for withholding information. Requests for interim results or other details arising during the study which if honoured are likely to have an adverse effect on the study, will be denied. Only one individual will be authorized to interact with the press (the local Principal Investigator or public relations officer of his/her institution) at every site in coordination with the CU. In the multicentre study context, the EC will respond to queries concerning the overall study design or results. The SC will define the type of queries that may be answered locally and those that must be referred for response to the EC. Publicity concerning study results in preparation will be avoided.

For peer review only - http://bmjopen.bmj.com/site/about/guidelines.xhtml

Appendix G: Management Structure

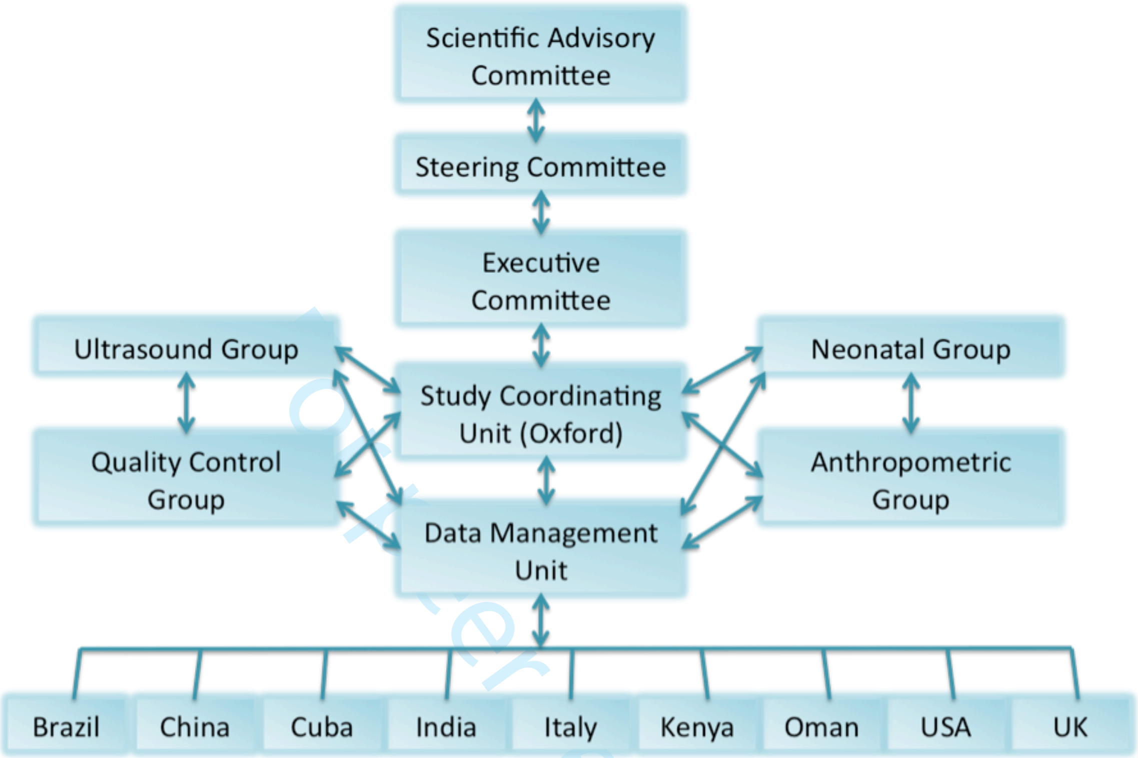

For peer review only

Appendix H: Selection of Study Sites Flow

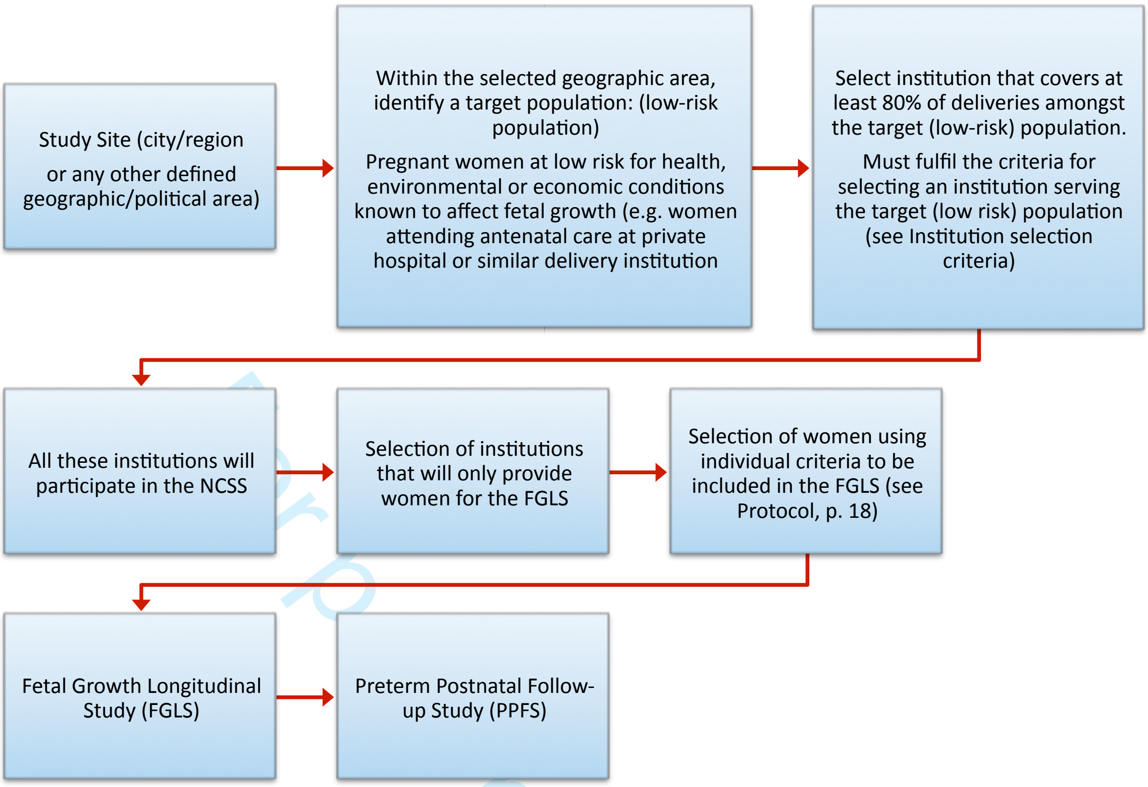

Institution Selection Criteria

a) LBW rate <10% and mean birth weight >3100g;

b) located at an altitude below 1600m;

c) perinatal mortality <20 per 1000 live birth;

d) mothers attending antenatal care in these institutions should plan to deliver in that or a similar hospital located in the same region;

e) >75% of mothers have attained an educational level greater than the locally defined cut-off point;

f) lack of known non-microbiological contamination such as pollution, radiation or any other toxic substances (this will be evaluated in collaboration with the WHO Department of Protection of the Human Environment);

Appendix I: The Pregnancy Physiology Pattern Prediction (4P) Study Details of observation measurement

Additional observations

Minimum dataset: In addition to blood pressure (that is currently recorded), temperature, respiratory rate, heart rate and oxygen saturation will be recorded at each ultrasound scan visit; this should only add 2 minutes to each visit.

Minimum intra-partum and post-partum dataset (hospital): All routinely collected intra-partum and post-partum measurements (blood pressure, temperature, respiratory rate, heart rate and oxygen saturation) will be added to the dataset.

Full dataset: Each participant will, if willing, be trained in the use of home monitoring equipment. The participants will then be asked to provide a daily dataset for the 2 weeks after delivery.

- Resting blood pressure will be recorded, but not displayed; readings will be downloaded into the research database at each scan visit.
- Resting pulse oximetry and heart rate will be recorded and directly transferred via the (bluetooth-connected) smartphone to the research database.
- Temperature will be taken and entered by the participant into the smartphone provided and automatically transferred to the research database.
- Automatic text reminders will be sent to the smartphone when datasets have not been received.

A research midwife will be employed to visit participants, reinforcing observation techniques. A complete dataset will be obtained during each visit. Two weeks after delivery, study personnel will arrange pick-up of the home monitoring equipment; a final dataset will be collected at this visit.

Blood pressure: When measuring blood pressure in the clinic or in the home, standardise the environment and provide a relaxed, temperate setting, with the woman quiet and seated, and her arm outstretched and supported. Blood pressure should be measured approximately 5 minutes after the woman has been seated and relaxed, with legs uncrossed. The arm should be supported at heart level. The correct cuff size should be used:

British Hypertension Society
Standard cuff Bladder 12-26 cm for the majority of adult arms
Large cuff Bladder 12-40 cm for obese arms
Small cuff Bladder 12-18 cm for lean adult arms

(Combined European Society/NICE/British Hypertension Society recommendations)

Blood pressures will be stored within the device and downloaded into the database at scan visits/home visits.

Temperature: Tympanic and oral temperature will be measured at each scan visit, using standard techniques. Self-measurement of temperature will be via the tympanic route only and will be entered into the smartphone by the participant. Midwife home visits will record both tympanic and oral temperature.

Pulse oximetry: Oxygen saturation and heart rate will be recorded at each scan visit using standard hospital equipment. For each self-observation set, oxygen saturation and heart rate will be relayed to the smartphone, and uploaded directly to the research database.

Respiratory rate: Respiratory rate will be manually counted for 1 minute at scan and home visits. At the end of each home observation set, respiration rate will be estimated using an accelerometer within the smartphone, with the smartphone applied to the chest by the participant sitting back in her seat quietly for 30 seconds.

For peer review only

Appendix J: The PEA POD Manual of Operations

The PEA POD uses a series of standard equations to estimate body composition. Those equations require a subject's weight (w), length (l) and volume (V). Weight and volume are measured by the machine itself. Length needs to be measured beforehand.

Warnings and cautions

The machine also requires a quiet and stable environment:

It must be on a flat floor (can be checked in System setup → inclinometer)
Not to be near a heater, air conditioning machine, fan or under direct sunlight
Not to be near a window, a door (especially one that could be opened during testing)
It is sensitive to low frequency noise
It must not be touched during a test
The room temperature (RT) should be between 20-28°C. If RT is out of range (by even 0.1°C), an alarm will go off and what you are doing will be lost. To prevent that if happening, ideally keep RT around 25°C (open windows and AC also limit the number of people in the room)
Moreover, RT should not vary by more than ±0.5°C during testing.
The test chamber temperature is maintained at 31°C
Room humidity 20-70%, variation during testing ±5%
Atmospheric pressure between 86-106kPa

The machine is designed for newborns and infants between 1 to 8kg (from birth to 5-6 months).
Do not turn the machine on if you are not using it.
The machine will need cleaning after each usage.
Also worth knowing, the temperature in the PEA POD room increases very quickly.

Working sequence

Turn PEA POD and air-conditioning on
Warm-up (2h)
Calibration:
Analyze hardware → Scale calibration * →Scale check → Autorun → Volume
*: scale calibration is to be performed every 2 weeks or if the PEA POD has been moved.
Test:
Volume → Enter patient's data → Scale → Volume measurement → Cleaning
Repeat as many times as the number of babies to measure
Print out results & Back-up
Log out
Turn machine off

Turning on/off the machine and warm up

Check the machine is on at the wall socket and at the back of the machine (main). The PEA POD and the PC will turn on together and the PEA POD software will launch automatically. The log-in window appears.

For peer review only - http://bmjopen.bmj.com/site/about/guidelines.xhtml

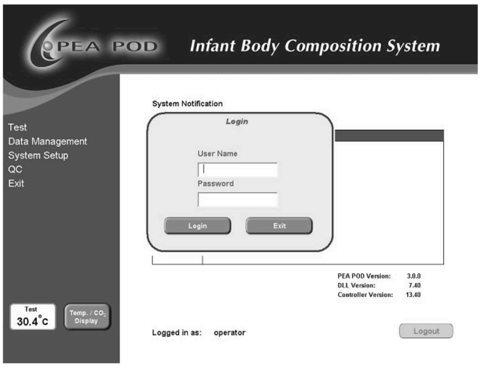

Log-in information:

User name: jiang

Password: peapod

(Data management password: admin)

When you turn the machine on, a system error message will be displayed 'Test Chamber Too Cool' → It will disappears when the test chamber has warmed up.

The machine needs 2 hours to warm-up. If tests are plans in the morning, the machine can be left on overnight. However, the running temperature of the PEA POD is 28.8°C, so you will need to turn the AC on or open a window in the morning before calibrating.

When the machine is turned on, the air circulation system starts. It will turn itself off after 10min of inactivity. The PEA POD will require an extra 5min to re-circulate air when becoming active.

On the day, if the machine is left inactive for a couple of hours, it is recommended to do a couple of Autoruns: QC → Autorun (Each takes about 10 min).

Do not turn the machine on if you are not using it.

To turn the machine off:

Log off and exit the PEA POD program (the PEA POD will stop)

Turn the PEA POD and the PC off → bottom right of keyboard, hold pressed for 3s

Turn everything off (main at the back and plug)

Operation

The interface of the machine is very user friendly. Follow the instructions on screen.
Remember to always execute the QC menu after the warm-up. Autoruns should be performed if the machine is left idle for a couple of hours.
Subject's behaviour and preparation:

The subject's hair should be flattened against their head using baby oil. In case the baby has a lot of hair or has curly hair, the baby should wear a cap, provided with the machine.

Calmer subjects make the experience more agreeable – testing after feeding results in calmer subjects. However, subject's behaviours (crying, movement) have been shown not to affect the results.

By default, the models used by the PEA POD are:

Body composition: Fomon
Thoracic gas volume: Stocks
Surface area artefact: Boyd

For peer review only

Menu titles

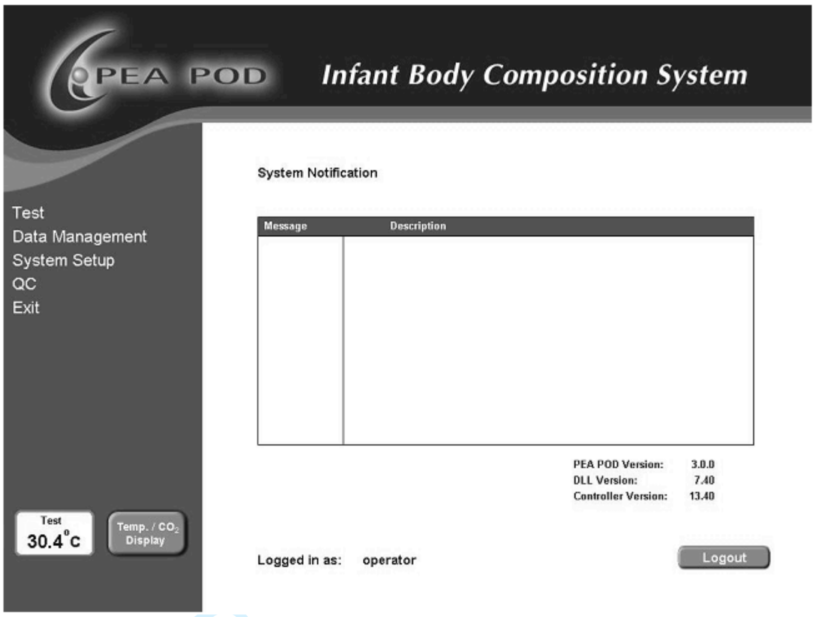

This is the main screen:

In the blue column on the right you will find:

- List of menus
- Environmental conditions (There can be 1 – like here – or 5 indicators)
- Error messages appears just underneath these indicators

In the middle of the screen:

- The system notification box where error message appears.
- Other information: Software versions and user's identity

On the bottom right:

- logout button

Test → Body Composition → Mass

Select this option when ready to carry out a test (after QC)

Data management → View subject results
→ Modify subject results
→ Delete test
→ Export result
→ Database backup

Database backup should be based on usage rather than regular time intervals

System setup → Test profile → Create
→ Remove
→ Density model → Create
→ Remove

Do not modify those

→ Scale checks → Noise and drift
→ Hysteresis
→ Inclinometer

No need to perform those operations

→ Facility
→ Automatic backup directory

QC → Analyze hardware
→ Calibrate scale * [4x]
→ Check scale ** [1x]
→ Autorun ** [6x]
→ Volume ** [3x]

* Only to be performed, if:
The PEA POD has been moved
If 2 weeks have elapsed since the last measurement

Remember:
To do run the QC menu everyday
** To record the QC in the log books

Exit

To be performed again if machine left inactive for a couple of hours

For peer review only

Maintenance schedule

After each subject: remove any waste (if applicable); clean scale tray, chamber tray and shields. Wipe with a cloth impregnated with cleaning solution, dry with clean cloth. According to Life Measurement, Inc. We shouldn't use isopropyl alcohol, solvents (like acetone), Virex and phenolic germicidal detergent solutions.

We can *a priori* use our regular wipes: Clinell® or PDI Sani-cloth® wipes.

There should be a clinical waste bin (yellow bin) just outside the PEA POD room (or somewhere along that corridor) where we can dispose of the scale lining, cleaning wipes and dirty nappies.

Do leave that bin outside to be emptied regularly.

Weekly: clean sides and top using cleaning solution and procedures specified in manual
Monthly: check pre-filter (replace if dirty) and clean test chamber (Zedong)
Bi-monthly: replace pre-filter (Zedong)

The pre-filter is situated at the back of the machine on the bottom left if you face the rear of the machine. They are behind a grid and they are blue. They have to be replaced if they appear to collect a lot of dirt.
The Brillianize spray is for the window only.

System errors

Several messages can appear:

Test chamber too cool
Ambient temperature out of range
Scale out of level
Test and reference chamber temperature too far apart
Volume sequence time limit **

➔ Contact customer service if persistent

Calibration valve pneumatic pressure out of specification
Test chamber door does not close
Door opened by door fail safe

➔ Contact customer service
➔ techsupport@lifemeasurement.com

** The volume sequence time limit means the time elapsed between the volume measurement test should not have any delays. Meaning, you need to stay at the machine and follow instructions as soon as they appear on the screen.
The measuring sequence is as follow:
V calibration → Patient data entry → Scale taring → Mass measurement → Volume measurement → Results
If 10min have elapsed between the end of calibration and beginning of V measurement, the alarm will go off and the whole measuring sequence of calibration is to be repeated.
Click 'cancel' → Test → Body Composition

For peer review only

Alarms

Alarm table – part I

Alarm	Activation criteria	Alarm response	Alarm silence period	Code	Alarm tone
High air temperature	3°C above set T or T>40°C	Audio, LED Screen Test chamber door opens Heater turned off	10min	101 - 109	4 rapid bursts, ½ sec pause, repeat
Air heating system failure	Over-T and/or failure of any system component including any one of the temperature sensors		2min	201 - 211	
Air recirculation system failure	Air flow out of specification		2min	301 & 302	
High carbon dioxide	Carbon dioxide level >0.45%		10min	401 & 402	
Power supply out of range	Voltage out of range		2min	501 - 503	
Remove subject from the scale	Failure to remove subject within 3min of measurement completion	Audio, LED Screen	NA	601	
Continue volume sequence	Failure to continue volume sequence within 10min of previous step		NA	701	

The last 2 alarms (not coloured) are activated by the lack of progression in the testing sequence.

For peer review only

Alarm table – part II

**Communication failure ***	**Loss of communication between GUI and electronics**	**Audio, LED Test chamber door opens Heater turned off**	**10min**	**801**	**3 chimes, 2sec pause, repeat**
**Redundant safety system**	**Controller stops executing code (crashes)**	**Audio, Test chamber door opens Heater turned off**	**None**	**901**	**1 chimes, 2sec pause, repeat**
	**Controller spontaneously reset itself**	**Audio**	**None**	**902**	**2 chimes, 2sec pause, repeat**
**Alarm**	**Activation criteria**	**Alarm response**	**Alarm silence period**	**Code**	**Alarm tone**

The sections that are coloured represent alarms that require the PEA POD to be switched off (keyboard) and then also at the main (back of the machine)

* This alarm require the PEA POD and the PC to be switched off independently

Responding to alarms

In case of one or multiples alarms, the following steps should be followed:

Recognize the occurrence of an alarm by appearance of one or more alarm messages on the screen, the blinking LED light on the top right corner of the touch pad area, and the audio alarm sound.

If the subject is on the scale tray or in the test chamber, remove the subject immediately from the PEA POD. Place the subject in a safe place before attending the alarm. Do not place the subject on the scale tray while attending to the alarm.

Silence the alarm by pressing the power button located in the bottom corner of the touch pad area for one quarter second or by clicking on the <SILENCE> button on the alarm message displayed on the screen. Note that Redundant Safety System alarms cannot be silenced.

Note the name of the alarm displayed, associated code, and tone (the tone is particularly important for Communication Failure alarms and Redundant Safety System alarms, as in those cases there will not be a software displayed message specifying an alarm name and code). Also note when and how the alarm occurred.

Shut down the PEA POD by pressing and holding the PEA POD power button for 3 sec and turning switch located on the back panel to the OFF position. In case of a Communication Failure alarm, the PEA POD and computer need to be shut down independently using the PEA POD power button and shutting down the computer using the start menu.

Contact LMI Customer Service and provide the name(s) of the alarm(s) that occurred, associated code(s), tone, and a description of when and how the alarm(s) occurred.

For peer review only - http://bmjopen.bmj.com/site/about/guidelines.xhtml

Results print-outs, export and backup

To avoid transcription error, we thought it would be easier if we print out 2 copies of the results:

- One to be stored in the corresponding folder for future reference in the PEA POD room
- One to be attached to the result sheet

10.1 Data exports and import into Excel

10.1.1 To Export data

The results are exported to a text file in a tab-delimited format. Further data analysis can then be performed using software programs (e.g. Excel...)

Data management →Export results

Enter password → Click OK

Select Results to Export:

The following filters can be applied: last name, gender, test date, body mass, ID_1, ID_2, ID_3 and/or ID_4. One you selected the filter(s) you want, click apply.

If you don't select any filter and click 'Apply', every results will be listed in the window.

If your search yields more than 200 results, a dialogue box will appear with the following options:

- Click 'Back' to further limit the number of records
- Click 'Preview' to populate the table (this may take some time)
- Click Export to export all records without previewing the data

Modifications to models can be implemented. If you scroll sideways the result list, you will find columns for the default models used. You can change them if you go to their scroll down menu (down arrow). The will not affect the database just the exported results.

Select the results you want to export → Click 'Next'

Name your file → Click 'Next'

The file will be saved in C:\PEAPOD Data\Export Results\

10.1.2 To transfer data

To transfer the data, exit the software and go to the above address or use the shortcut on the desktop. There are 2 USB port in the panel on the left side of the screen. Copy or move your file to a memory stick.

10.1.3 To import data into Excel

In Excel, go to Open.

Select your drive and folder. In the 'Files of type' at the bottom of the 'Open' dialog window, select 'All Files'.

Select your file → Click 'Open' → Click 'Next' → Click 'Next' → Click 'Finish'

The results will appear in an Excel spreadsheet, each column will be a field from the 'Enter Subject information' screen. Each test will occupy a line.

For peer review only

10.2 Database backup

This activity allows for backup of the contents of the database so that in case of a PEA POD malfunction, data can be restored. Database backup should be based on usage rather than regular time intervals.

Data management → Database Backup

You can modify the name of your file but it will always be appended by its creation date (dd-mm-yyyy). The file will be stored at C:\PEAPOD data\Database backup.

If the database is saved twice in the same day and the name is not changed, the latest one will replace the other.

Click ‘Next’ → Click ‘Finish’

10.3 Correction

If, for some reasons, you have entered wrong subject’s data, you can correct them. The computer will automatically update the results if affected.

Data management → Modify Subject Results → Log-in as administrator → Select Subject’s test (2 steps) → Modify subject’s information → click ‘Next’ → Click ‘Repeat Modify subject Results’ or ‘Finish’ to go back to the main screen.

Enter subject information page

During the automatic volume calibration, a screen will appear where you have to enter the subject’s details. At this point you can either retrieve information on a subject from a previous measurement or enter new details. If you enter as a new subject, a subject that is already in the system, the computer will ask you if you want to merge the 2 files or modify the latest one to differentiate it from the old one. The fields available are the following (* = mandatory).

Click next when finished

Noteworthy: at the conclusion of the volume measurement, results are displayed on screen. They can then be reviewed and some entries modified if incorrect. Proceeding to the next step saves the results and provides the option to print them out.

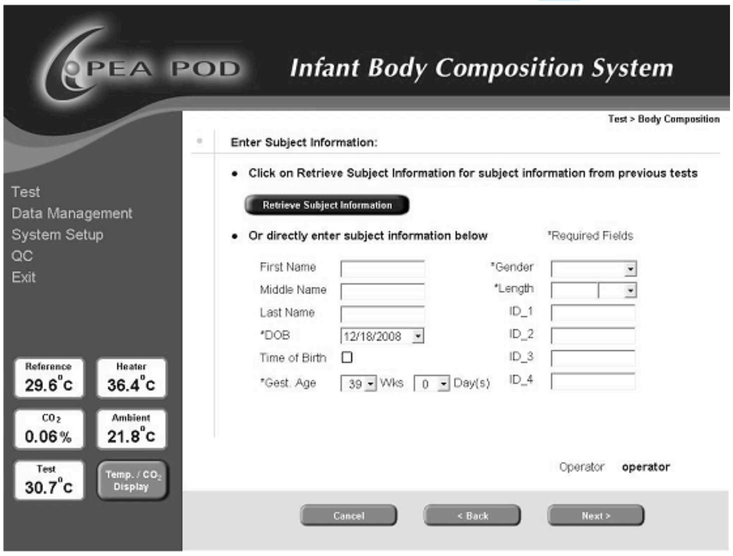

First name	FGLS
Middle name	
Last name	FGLS number – see list in the PEA POD room
* DOB	DD/MM/YYYY
Time of birth	The software only gives the option of 'finite' hour. So it was decided that: If a baby was born before half past the hour, we would enter as its time of birth the last complete hour and If a baby was born on or after half pas the hour, we would enter as its time of birth the next complete hour. For example, if a baby was born between 1pm and 1.29pm, time of birth is 1pm and if a baby was born between 1.30pm and 1.59pm, time of birth would be 2pm.
* Gest age	Gestational age at birth. Enter weeks and days. If any doubt, please refer to the FGLS list that should be in the room. On that list, the women are arranged by EDD (i.e. 40+0 weeks). Also there should be a wheel available in a folder.
* Gender	Male or Female
* Length	The length has to be measured prior to the test. There should be an infantometer in the PEA POD room. The software gives you the option of unit (cm or inches).
ID_1; ID_2; ID_3; ID_4	We are not using those fields for the moment. We might use them later on under the INTERBIO study to differentiate sub-population (IUGR, Macrosomia...)

12. Other information

12.1 How to refer to the PEA POD

Pediatric Air-displacement plethysmography

→ PEA POD (COSMED USA, Inc., Concord, CA, USA)

Model used: Fomon (or Butte), Software version: 3.1.0

12.2 Scale taring and volume calibration

Remember to check what non-clothing items the baby is wearing and use the duplicate props during volume calibration and scale taring.

Number of hospital tag, umbilical cord clamp, feeding tube, splint, canula...

If the baby had his/her length measured earlier, check which items to babies is wearing but do not undress baby just as yet as you need to execute volume calibration and scale taring. You will be provided with a PEA POD form that you or an anthropometrist should have completed with baby's length. Do not forget to record the information requested.

12.3 Number of people in the room for environment control

Remember to put the 'do not disturb' sign outside on the door

Limit the number of people in the room: 2 experimenters and parents (1 or 2)

If the AC is on, you should turn the A/C off if it is blowing directly on the PEA POD or if it causes the room temperature to fluctuate. If you turn it off during the QC measurements to have a more stable environment, then you should also turn it off during a body composition tests.

For peer review only

12.4 Images of the PEA POD machine

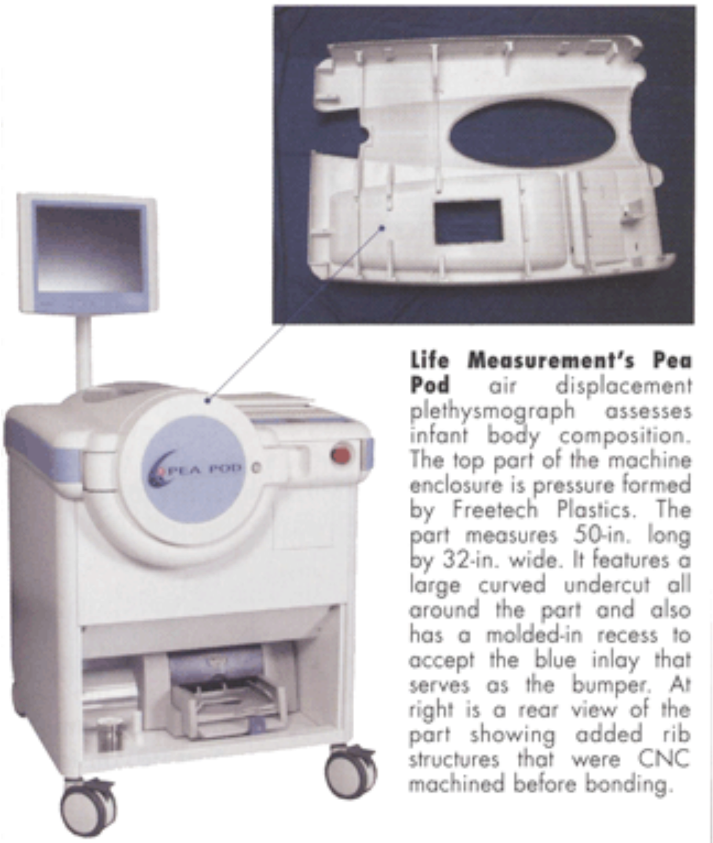

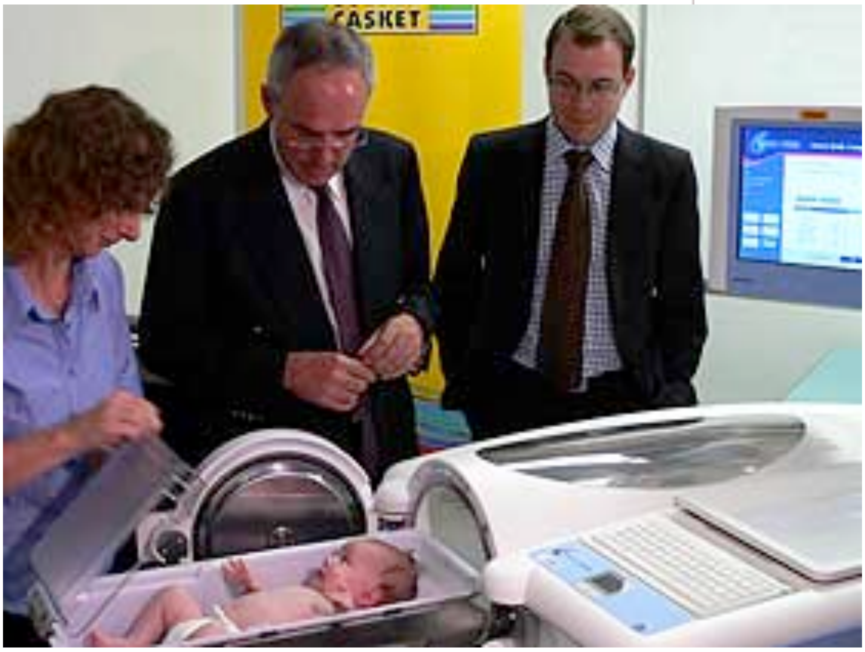

Appendix K: The INTERBIO-21st Study Protocol

The INTERBIO-21st Study

The Functional Classification of Abnormal Fetal and Neonatal Growth Phenotypes

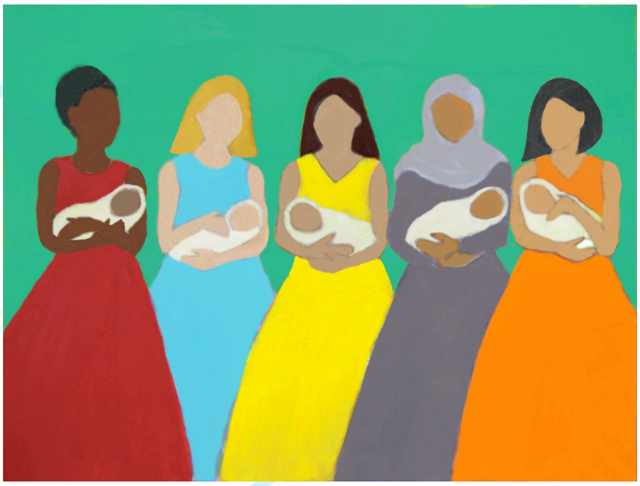

Study Protocol

The INTERBIO-21st Consortium

Oxford Maternal & Perinatal Health Institute (OMPHI)
Nuffield Department of Obstetrics & Gynaecology
University Department of Paediatrics
Wellcome Trust Centre for Human Genetics
and Oxford Tropical Medicine Network
University of Oxford, UK
Global Alliance to Prevent Prematurity & Stillbirth (GAPPS)
in association with:

Universidade Federal de Pelotas, Pelotas, Brazil; **The Aga Khan University Hospital**, Nairobi, Kenya; **Aga Khan University Medical Centre**, Karachi, Pakistan; **KEMRI-Coast Centre for Geographical Medicine & Research,** Kilifi, Kenya; **Shoklo Malaria Research Unit,** Mae Sot, Thailand; **University of Witwatersrand,** Johannesburg, South Africa; **John Radcliffe Hospital**, Oxford, UK.

Version 2, October 2012

For peer review only

Acknowledgements

This study is supported by a generous grant from the Bill & Melinda Gates Foundation to the University of Oxford, for which we are very grateful.

We acknowledge the enormous contribution made by our co-investigators and collaborating centres worldwide.

We would also like to thank the Health Authorities in Brazil, Kenya, Pakistan, South Africa, Thailand and the UK, who have facilitated the study by allowing participation of these sites as collaborating centres.

Credits

This study protocol is based on a first version produced between May 2010 and January 2011 principally by José Villar, Stephen Kennedy, Aris Papageorghiou and Ann Lambert (University of Oxford), with important contributions from Prof Cesar Victora (Federal University of Pelotas). Sections I-IX and the Appendices were revised in October 2012.

This protocol should be referenced as:
The INTERBIO-21st Consortium,
INTERBIO-21st Study Protocol, Oxford, October 2012.
(www.interbio21.org.uk)

For peer review only

Table of Contents

I. List of abbreviations used 4
II. Summary 5
III. Background 6
IV. **PROGRAMME I:** INTERBIO-Bank background 8
V. INTERBIO-21st Fetal Study 11
VI. INTERBIO-21st Neonatal Study 13
VII. Selection of cases and controls 15
VIII. Sample processing 17
IX. Selection of study centres 19
X. **PROGRAMME II:** Proof-of-concept study background 20
XI. Study design 21
XII. Sample size for epigenetic studies 23
XIII. Governance of study 25
XIV. Appendix I: Instructions for Recruiting Cases and Controls for NS 26
XV. Appendix II: Technical note on selection of controls 27
XVI. Appendix III: Definition of intrauterine growth restriction in field studies 29
XVII References 30

For peer review only

List of abbreviations used

**AGA**	Appropriate for Gestational Age
**BPD**	Biparietal Diameter
**BMI**	Body Mass Index
**CRL**	Crown Rump Length
**CI**	Confidence Interval
**DDT**	Dichloro-Diphenyl-Trichloroethane
**DSS**	Demographic Surveillance System
**FGLS**	Fetal Growth Longitudinal Study
**GWAS**	Genome Wide Association Studies
**HC**	Head Circumference
**IQTL**	Imprinted Quantitative Trait Loci
**IUGR**	Intrauterine Growth Restriction
**KDH**	Kilifi District Hospital
**LBW**	Low Birth Weight
**LMP**	Last Menstrual Period
**MeDIP-Chip**	Methylated-Cytosine DNA Immunoprecipitation-Microarray Chip
**MMN**	Multiple Micronutrient
**NICU**	Neonatal Intensive Care Unit
**NCSS**	Newborn Cross-sectional Study
**PPFS**	Preterm Postnatal Follow-up Study
**PCR**	Polymerase Chain Reaction
**RCT**	Randomised Control Trial
**RR**	Risk Ratio
**RT-PCR**	Real Time PCR
**SMRU**	Shoklo Malaria Research Unit.
**SGA**	Small for Gestational Age
**SNP**	Single Nucleotide Polymorphism

**INTERBIO-21st**	The INTERBIO-21st Study: The Functional Classification of Abnormal Fetal and Neonatal Growth Phenotypes
**INTERGROWTH-21st**	The INTERGROWTH-21st Project: The International Fetal and Newborn Growth Consortium for the 21st Century

For peer review only

SUMMARY

The INTERBIO-21st Study aims to evaluate newborn phenotypes so as to understand better the relationship between the causes of IUGR/SGA and preterm birth syndromes. It is based upon our hypothesis, presented in the initial INTERGROWTH-21st Project, that phenotypic subgroups other than those defined by birth weight and gestational age alone are needed to determine a newborn's nutritional status and assess the effectiveness of interventions to prevent and/or treat the effects of an adverse intrauterine environment. In effect, therefore, we are aiming to produce a more "functional" description of these syndromes.

The redefinition of newborn subgroups will arise from evaluating a combination of factors in pregnancies with normal and abnormal outcomes. These factors include maternal health; fetal growth patterns; growth patterns of fetal organs; newborn body composition and physiological function; micronutrient levels and data from epigenetic experiments. We will initially characterise normal genetic variability and normal variability across the epigenome in uncomplicated pregnancies, and compare these data to the variability observed in a sample of high-risk pregnancies. In a series of case-control studies, we will evaluate the effects of adverse environmental and nutritional factors (and other biomarkers), which possibly interact with genetic factors and the epigenome, on the sub-groups of IUGR/SGA and preterm birth.

The rigorous clinical and laboratory-based characterisation of newborn phenotypes and their different aetiologies in relation to morbidities, especially those that are common in resource-poor settings, should lead to better clinical management of pregnancies and newborn complications. This will contribute to the selection of more effective preventive interventions and screening strategies by improving their specificity.

Specifically, we shall:

PROGRAMME I: Create a unique biobank (INTERBIO-Bank) of maternal blood, maternal faeces and cord blood/placental samples from at least six populations with different risk profiles, including women at high risk for preterm delivery and IUGR/SGA because of malnutrition and/or infection. We shall follow a longitudinal and cross-sectional study design in two sub-studies. These samples will be used primarily to explore risk factors and biomarkers for the subgroups of IUGR/SGA and preterm delivery.

PROGRAMME II: Conduct, in the first of a series of experiments, a hypothesis-testing, proof-of-concept study comparing DNA methylation patterns and micronutrient status in term AGA and IUGR/SGA newborns drawn from the INTERBIO-Bank.

Figure 1: INTERBIO-21st Study Flow Diagram

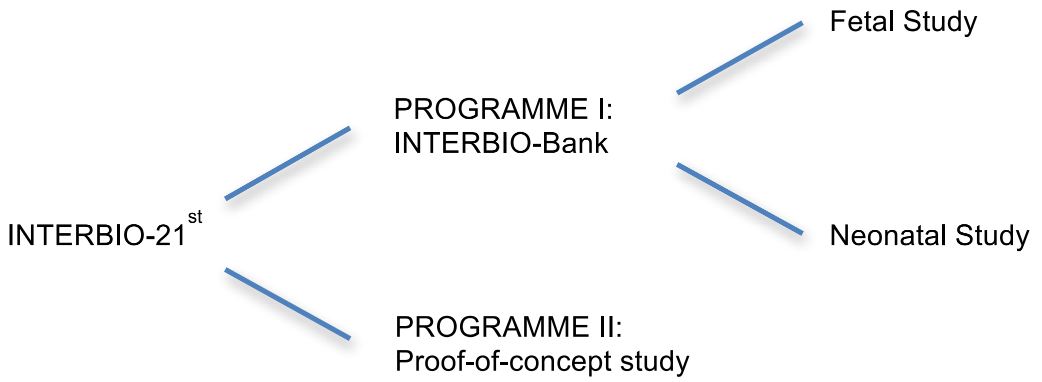

For peer review only - http://bmjopen.bmj.com/site/about/guidelines.xhtml

BACKGROUND

The INTERBIO-21st Study builds upon the International Fetal and Newborn Growth Consortium for the 21st Century (INTERGROWTH-21st), a unique, population-based project that is being conducted in eight different geographical locations in Brazil, China, India, Italy, Kenya, Oman, the UK and US. (www.intergrowth21.org.uk).

The primary objective of INTERGROWTH-21st is to develop new "prescriptive" standards, conceptually similar to the WHO Child Growth Standards, describing optimal fetal and preterm neonatal growth and newborn nutritional status, and to relate these to neonatal health risk. This objective is being achieved by implementing three studies involving detailed and highly standardised recording of maternal characteristics and anthropometry, pregnancy complications, exposure to pollutants, fetal growth, neonatal anthropometry and perinatal outcomes:

1. Fetal Growth Longitudinal Study (FGLS): ultrasound and clinical assessment of fetal growth every five weeks throughout pregnancy from <14 weeks, with accurate early pregnancy dating, in eight populations with optimal health, in defined geographical areas with low environmental risks. It will produce ultrasound and clinical Fetal Growth Standards.

2. Preterm Postnatal Follow-up Study (PPFS): follow-up of infants from the FGLS cohort born prematurely with regular anthropometry and nutritional evaluation to describe their postnatal growth pattern up to 2 years. It will produce Preterm Postnatal Growth Standards. All newborns from the complete cohort (FGLS and PPFS) will be seen at 1 and 2 years to evaluate health, nutrition and development.

3. Newborn Cross-Sectional Study (NCSS): anthropometric measures, neonatal morbidity and mortality, and pregnancy complications assessed in all newborns at each of the study centres over a 12 month period, i.e. all deliveries are being captured over 12 months from the same areas. It will produce Newborn Birth Weight for Gestational Age Standards.

The secondary objectives are:

d) **Clinical:** to develop a prediction model, based on multiple 2-dimensional (2D) ultrasound measurements, for estimating gestational age during mid-late pregnancy for use in populations of pregnant women without access to early/frequent antenatal care;

e) **Epidemiological:** to study in this multi-ethnic, population-based sample the determinants of LBW and its components (preterm delivery, impaired fetal growth and their subgroups) under current healthcare conditions, and

f) **Biological:** to acquire additional 3-D images to create an anatomical and growth databank of individual fetal organs as a unique source of biological information for future research.

The study populations from these geographically defined areas have no socio-economic constraints on growth; low morbidity and perinatal mortality, and adequate nutritional status. To be included, women must be non-smokers, with a normal pregnancy history, and without health problems likely to influence fetal growth or indicate a risk for pregnancy-related pathological conditions.

In **FGLS**, women are screened $<14^{+0}$ weeks at their first antenatal visit and followed-up with standard clinical and 2D ultrasound examinations every five weeks, i.e. up to six times during pregnancy. In **PPFS**, preterm infants ($> 26^{+0}$ but $< 38^{+0}$ weeks) born from this sample are being followed-up during their first 8 months of life with the same protocol and set of anthropometric measures used in the WHO Child Growth Study. Postnatal growth is being evaluated from both delivery and conception for comparison with the corresponding *in utero* measurements. All infants from FGLS and PPFS will also be seen at 1 and 2 years to evaluate health, nutrition and development.

In **NCSS**, all newborns at the study centres, born during a fixed 12 month period, have anthropometric measurements taken immediately after birth. Only babies born to women who meet the same inclusion criteria used in FGLS are being selected to construct the newborn standards. Birth weight and gestational age will also be related to neonatal morbidity and mortality outcomes to construct risk-related newborn weight for gestational age standards.

Standard quality control measures are being used, including adaptation of the ultrasound machines to ensure that blinded measurements are taken; a unique system of random evaluation and repetition of ultrasound measurements (from stored images) to monitor validity and reliability, and continuous real time assessment of all data collected. Anthropometric measures of all neonates are being monitored and standardised centrally. All data are entered and managed in an on-line system specifically developed for the study, including a means of transferring blinded data directly from the ultrasound equipment to the database. This allows initiation of data analysis soon after data collection is completed.

For peer review only - http://bmjopen.bmj.com/site/about/guidelines.xhtml

Figure 2: Three INTERGROWTH-21st cohorts

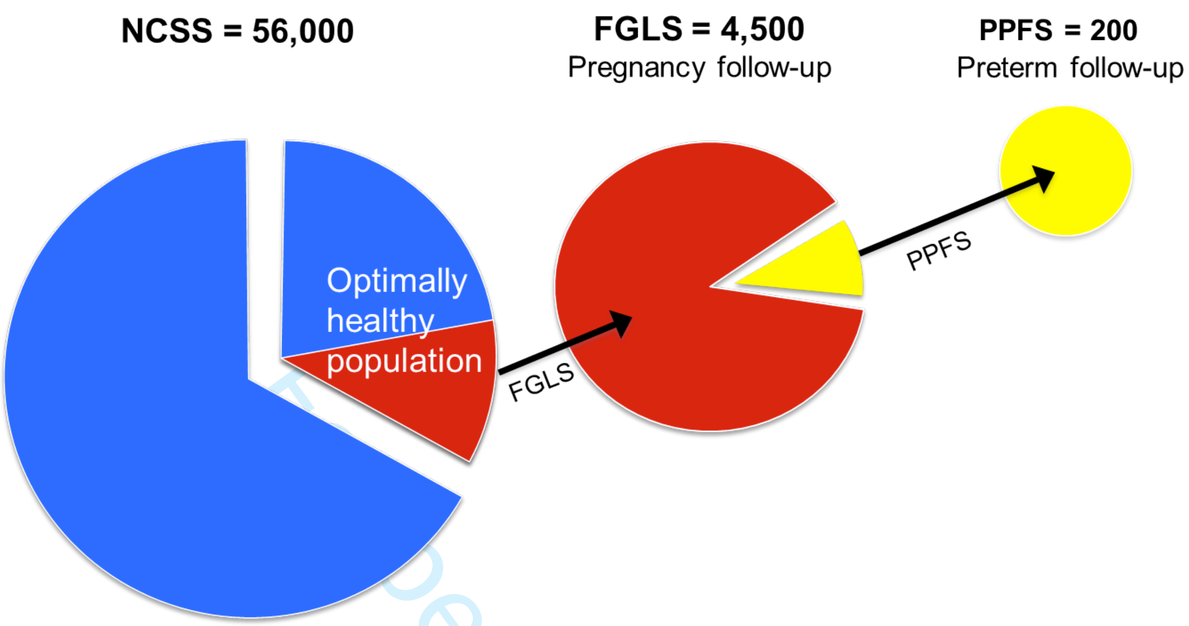

For peer review only

PROGRAMME I. INTERBIO-Bank

Create a biobank of maternal blood and cord blood/placental samples

Background

The aim is to establish a biobank (INTERBIO-Bank) of maternal blood, maternal faeces and cord blood/placental samples from healthy and complicated pregnancies to allow nutritional, epigenetic and other biomarker studies to be performed.

Collecting a heterogeneous group of cases will allow us to explore the wide range of aetiological factors (genetic, metabolic, vascular, autoimmune, infectious etc.) contributing to the development of complicated pregnancies that may present in the same way phenotypically (e.g. low gestational age), as well as the interactions between risk factors and outcomes. Ultimately, we aim to integrate all the pregnancy-related, clinical and biomarker data to improve the phenotypic characterisation of newborns, so as to facilitate the development of targeted interventions and screening strategies in pregnancy and early infant life. [c]

The pathways leading to pregnancy complications, e.g. preterm delivery, IUGR and SGA syndromes, are almost certainly controlled by multiple molecular, genetic, epigenetic and biochemical mechanisms. What is less clear is the relative contributions from risk factors such as infections, nutritional status and other environmental exposures, especially in resource-poor settings.

We hypothesise that:

1. There is more than one preterm delivery phenotype associated with inter-related pathways, i.e. the heterogeneous causes have different functional effects on the fetus/newborn.
2. Similarly, the IUGR/SGA phenotype has several intrauterine growth patterns, multiple causes (e.g. small maternal stature, poor maternal nutrition, infection, prematurity and utero-placental insufficiency), and neonatal and infant outcomes.
3. Hence, it is inappropriate to manage SGA and preterm newborns as single clinical entities, as usually occurs, based on the potentially false assumption that, *irrespective of the cause*, the adverse effects on the fetus and the clinical manifestations in the newborn are uniform;
4. These phenotypes will best be characterised by integrating measures of maternal health, fetal growth patterns, better estimation of gestational age and metabolic function, with biomarker data.

More rigorous clinical and laboratory-based characterisation of such phenotypic subgroups and their different aetiologies should lead to better clinical management of newborn complications and the development of more effective preventive interventions and screening strategies by improving their specificity. This is important because a lack of specificity of interventions tested in previous RCTs, particularly those to prevent preterm delivery, could have resulted in interventions that are actually effective in some phenotypic subgroups, being abandoned because they failed to show an overall protective effect.

A good example is the finding that calcium supplementation in low-risk women with low-calcium diets, significantly reduces the risk of pre-eclampsia (RR 0.48; 95% CI 0.33-0.69) but its impact on preterm birth (RR 0.81; 95% CI 0.64-1.03) borders on significance [1]. However, when the analysis was restricted to the four small RCTs including women at high risk of pre-eclampsia (n=568), there was a large and significant decrease in preterm birth (RR 0.45, 95% CI 0.24 to 0.83) [2]. Hence, it is possible that the magnitude of the effect of supplementation varies because the predominant preterm birth subgroups are different.

Similarly, although malaria infection clearly affects birth weight and gestational duration in epidemiological studies, a Cochrane systematic review of anti-malarial interventions in pregnancy showed that - among women in their 1st or 2nd pregnancies - treatment reduced anaemia, parasitaemia, placental malaria, perinatal deaths and low birth weight (RR 0.57; 95% CI 0.46-0.72), but had no effect on preterm births in the only trial assessing this outcome [2 3]. Thus, anti-malarial interventions may be effective in preventing only a subgroup of preterm births that is not seen when small trials use overall preterm rate as the primary outcome. Lastly, despite the considerable epidemiological evidence that gynaecological infections and bacterial vaginosis are associated with preterm birth, the results of several RCTs of antibiotic treatment of such infections have generally

[c] (Kramer MS, Victora CG Humana (2000); Barros FC, BMC Pregnancy and Childbirth (2010)

For peer review only - http://bmjopen.bmj.com/site/about/guidelines.xhtml

been disappointing [4 5]. However, it is possible that such treatments are still effective in reducing certain subgroups of preterm birth.

In addition, interventions that are phenotype-specific may, in the long-term, prevent the adverse metabolic and cardiovascular consequences of fetal malnutrition in adulthood. This general approach is of special relevance to resource-poor settings where targeting more homogeneous pregnancy and newborn sub-groups could considerably enhance the effectiveness of available resources.

The very thorough and highly standardised characterisation of antenatal events, using the same protocols in all the pregnancies will make this, to the best of our knowledge, the most comprehensive biobank in the world for nutritional, epigenetic and other biomarker studies in pregnancy.

Figure 3: INTERBIO 21st Fetal and Neonatal Studies: data and sample collection periods

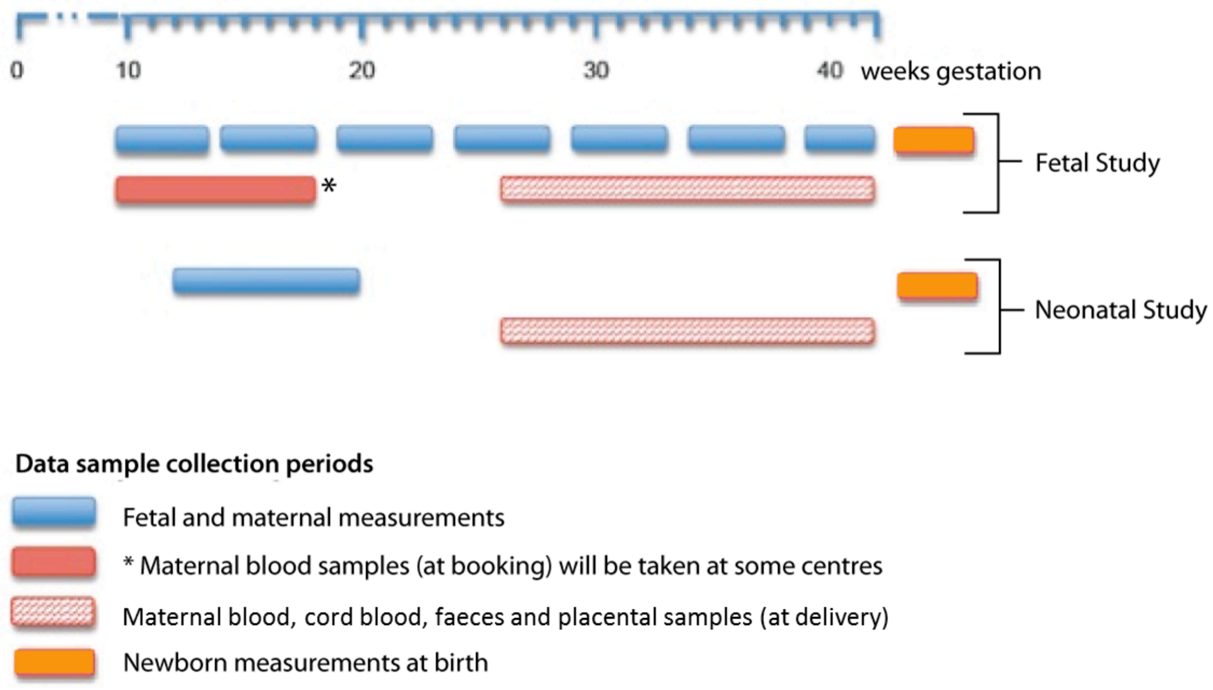

The biobank will be used for studies such as: genetics (SNP genotyping); epigenetics (DNA methylation, histone modification, imprinting, miRNA); expression analyses (mRNA and protein); micronutrient assays; immunohistochemistry; biomarker discovery and validation relating to outcomes such as preterm birth. Anonymised samples will also be made available to other biobanks via a process governed by the Biobank Management Group.

INTERBIO-Bank study design

We aim to collect and store maternal blood, maternal faeces and cord blood/placental samples (see Figure 3) to create a biobank from the following populations:

- 1) "Fetal Study" pregnancies in three centres currently in the INTERGROWTH-21st Project (Pelotas, Brazil; Nairobi, Kenya; Oxford, UK), supplemented by high-risk pregnancies in centres in resource-poor settings, monitored using the same protocol: **INTERBIO-21st Fetal Study**
- 2) "Neonatal Study" pregnancies in the same three centres (Pelotas, Brazil; Nairobi, Kenya; Oxford, UK), supplemented by high-risk pregnancies in centres in resource-poor settings, monitored using the same protocol: **INTERBIO-21st Neonatal Study**

The INTERBIO-21st Fetal Study will provide detailed phenotypic information based on fetal growth patterns and biological samples to investigate maternal/fetal nutritional status and maternal/placental/fetal biomarkers in pregnancies with optimal outcomes, as well as those complicated by a range of factors, including HIV, malaria, malnutrition and anaemia in resource-poor settings. In the field of DNA methylation in particular, this will be an important first step in describing normal variability in fetal/placental methylomes and how methylation signatures relate to both healthy and adverse clinical outcomes.

The INTERBIO-21st Neonatal Study will provide detailed newborn phenotypic information (including accurate gestational age at birth and neonatal morbidity) and biological samples for case-control studies of maternal/fetal nutritional and maternal/placental/fetal biomarkers in healthy pregnancies, as well as those complicated by a range of factors, including HIV, malaria, malnutrition and anaemia in resource-poor settings.

For peer review only

1) INTERBIO-21st Fetal Study: Collect and store maternal blood, maternal faeces and cord blood/placental samples from pregnancies in three INTERGROWTH-21st centres (n=500 per centre), supplemented by samples from high-risk populations monitored using the same protocols in centres in resource-poor settings (n=500 per centre).

In all centres, we plan to collect and store maternal blood, maternal faeces and cord blood/placental samples at delivery (in addition to the pregnancy and fetal growth data) from a total of 2,500 pregnancies (500 per centre). For details of blood, faecal and tissue sample collection see the INTERBIO-21st Operations Manual.

Detailed information will also be acquired about gestational age and fetal growth patterns starting at <14 weeks' gestation. This is of great relevance because of the recent evidence that fetal growth discrepancies, which can be detected by ultrasound as early as the 1st trimester, are associated with increased risks of preterm birth, low birth weight, and SGA at birth [6].

Inclusion criteria for INTERBIO-Bank

INTERGROWTH-21st centres that have already completed FGLS

Women from the entire population attending for antenatal care from <14 weeks' gestation, irrespective of their risk profile for adverse pregnancy/neonatal outcomes, should be recruited for INTERBIO-Bank. However, to participate, women must be at least 18 years old and their pregnancy must have been conceived naturally. Women who have a BMI over 35 must also be excluded from the study as their weight will be a barrier to accurate ultrasound scans. All other women are eligible.

New INTERBIO-21st centres

Women from the entire population attending for antenatal care from <14 weeks' gestation, irrespective of their risk profile for adverse pregnancy/neonatal outcomes, should be recruited for INTERBIO-Bank. However, to participate, women must be at least 18 years old and their pregnancy must have been conceived naturally. Women who have a BMI over 35 must also be excluded from the study as their weight will be a barrier to accurate ultrasound scans. All other women are eligible.

Estimation of gestational age at study entry

Gestational age at study entry will be estimated by ultrasound measurement of CRL <14 weeks. When LMP is available this should also be recorded. This estimation of gestational age by CRL takes into consideration that in a large proportion of very high risk pregnancies the LMP may not be known.

Fetal growth monitoring

After the first scan between 9^{+0} and 14^{+0} weeks, we will perform scans at ~5 weekly (±1 week) intervals. After the dating scan, 6 further visits (for fetal biometry) will be scheduled at ~5 weekly (± 1 week) intervals (i.e. 14-18, 19-23, 24-28, 29-33, 34-38 and 39-42 weeks). Seven measurements will be taken at each visit from 14^{+0} weeks onwards: Biparietal Diameter (BPD); Occipito-Frontal Diameter (OFD); Head Circumference (HC); Transverse abdominal diameter (TAD); Anterio-posterior abdominal diameter (APAD); Abdominal Circumference (AC) and Femur Length (FL). At each visit, the measurements will be obtained 3 times from 3 separately generated ultrasound images in a "blinded" fashion, and submitted electronically (with the associated images) to the Project Coordinating Unit. All the study centres will use equipment with similar characteristics. The staff will be appropriately trained following standardised procedures according to the corresponding FGLS Protocol and Ultrasound Operations Manual.

Pregnancy follow-up

Women in the study will receive standardised antenatal care (with some local variations) based on the recommended WHO package, part of which involves screening for conditions that emerge during pregnancy. All women recruited will be followed throughout pregnancy from the time of the first visit, irrespective of the pregnancy outcome.

Severe perinatal morbidity and mortality outcomes

We have decided to use an un-weighted composite outcome including at least one of the following conditions: stillbirth, neonatal death until hospital discharge of the newborn, newborn stay in Neonatal Intensive Care Unit (NICU) for ≥7 days or other severe neonatal complications. We believe this is a good proxy for adverse perinatal outcomes across countries. We have used it as a primary neonatal outcome in recent publications and it has been well accepted. Its only disadvantage is that it risks excluding, from the total number of early neonatal deaths, some cases amongst healthy, mostly term babies delivered vaginally who, after hospital discharge at 48 hours, develop severe complications or death up to 7 days post-natally without returning to the same hospital. However, missing these isolated cases is preferable to performing thousands of unnecessary home visits.

For peer review only - http://bmjopen.bmj.com/site/about/guidelines.xhtml

2) INTERBIO-21st Neonatal Study: Collect and store maternal blood, maternal faeces and cord blood/placental samples at birth from pregnancies in three INTERGROWTH-21st centres (200 newborns at <38+0 weeks' gestation plus 200 controls, and 200 IUGR/SGA plus 200 controls in each centre), supplemented by samples from high-risk pregnancies in resource-poor settings. For details of sample collection see INTERBIO-21st Operations Manual.

NCSS pregnancies in INTERGROWTH-21st are ideal, population-based cohorts for nutritional, epigenetic and other biomarker studies to study the causes of pregnancy complications and how they influence growth and development, principally for the reasons outlined in Box 1.

Box 1: Some unique characteristics of studies conducted using NCSS protocols

Geographically diverse populations

Large, population-based, sample size with severe morbidity and mortality outcomes

Early pregnancy dating by ultrasound provided by small number of standardised operators

Standardised methodology for maternal, newborn and infant follow-up anthropometric measures

Maternal morbidities during pregnancy captured prospectively

Environmental characterisation of the populations and individual participants

However, we recognise the need to enrich the collection of complicated pregnancies from populations with other risk factors that are especially relevant to the needs of developing countries. Therefore, we will supplement sample collection in the three INTERGROWTH-21st centres by also collecting samples from pregnancies from the general population in resource-poor settings where there is a high risk of fetal growth impairment and preterm delivery because of infection, malnutrition, poor socio-economic status and past adverse pregnancy outcomes. This strategy will increase the generation of cases from a relatively small population given the higher incidence of the conditions.

In these centres, we will collect and store samples from 800 pregnancies per centre:

> Maternal blood, maternal faeces, cord blood and placental samples will be collected from pregnancies (cases) that have delivered at <38+0 weeks gestation (n=200 per centre) or have resulted in IUGR/SGA newborns (n=200 per centre). Newborns that were born at <38+0 weeks' gestation and were IUGR/SGA will be included in both sets of cases as the case-control analysis will be carried out separately for each outcome.
>
> We will also collect the same samples from term AGA newborns (controls), i.e. non-IUGR, normal birth weight newborns at term, as a reference group (n=400 per centre, i.e. one control for each case).

All cases and controls are required to have had, reported in their medical records, an estimation of gestational age by ultrasound measurement of either CRL <14 weeks or HC <24 weeks. When LMP is available this should also be recorded. If the LMP is not available it should be recorded as such and ultrasound estimations will be used.

Because of the different populations in the centres selected, all analyses in this case-control strategy will be stratified by centre, and will only be pooled if there is no statistical evidence of heterogeneity**.**

Anthropometric measurements

All babies, i.e. all cases and controls, born during the study period will have weight, length and head circumference taken within 24 hours of delivery.

Standardised, electronic, digital, newborn weighing scales with a precision of 10g will be used and their calibration status will be checked twice a week; they will be replaced if they are faulty and cannot be repaired. We shall also provide all clinics with standardised infantometers for length (precision 0.1 cm) and tape measures for head circumference (precision 0.1 cm); these will be similarly calibrated and maintained. All anthropometrists will be trained centrally and monitored during the study following standard procedures by the Anthropometric Standardisation Unit; they in turn will train the nurses/midwives in how to apply the study's measurement protocol.

For peer review only - http://bmjopen.bmj.com/site/about/guidelines.xhtml

For a small subgroup, the following additional anthropometric measurements will be taken: arm circumference; thigh circumference; abdominal circumference and skinfold thickness, as well as neonatal body composition using air displacement plethysmography (PEA POD) in some centres.

Follow-up

All newborns during the study period, including those on NICU or special care, will be followed on a daily basis until hospital discharge to document severe morbidity and detect neonatal death. We will make strenuous efforts to coordinate and promote evidence-based care for the neonates born <38^{+0} weeks' gestation using materials developed as part of our best practice programme, by liaising with the lead neonatologist in each NICU before and during the study. We recognise that differences in practice will persist despite our best efforts, especially in resource-poor settings. However, we believe this is unavoidable in a very pragmatic study such as this, which is trying to reflect what happens on a daily basis in clinical practice. Furthermore, we will similarly make strenuous efforts to standardise the main protocols for feeding practices in each NICU before the study starts. During the routine site-visits by members of the Study Coordinating Unit and the Anthropometric Team we will monitor the implementation of the protocols.

Severe perinatal morbidity and mortality outcomes

We have decided to use an un-weighted composite outcome including at least one of the following conditions: stillbirth, neonatal death until hospital discharge of the newborn, newborn stay in NICU for ≥7 days or other severe neonatal complications. We have used such an outcome recently [7 8]; it requires limited standardisation of clinical diagnoses across hospitals and is well accepted as a marker in large, international, population-based studies of newborns that are severely ill.[d] It could be argued, however, that intrapartum stillbirth may not be related to fetal growth and should not be included in this index. We believe this is a valid point but as it will not be possible to separate those intrapartum deaths that are related to IUGR from those that are unrelated, we suggest keeping the index as it is. We believe this is a good proxy for adverse perinatal outcomes across countries.

On-line data management and statistical analysis

All clinical data will be entered into an on-line data management system specifically developed for the study. It includes a method for direct transfer of blinded data from the ultrasound machines to the database. This on-line system has the practical benefit of allowing on-going quality control, correction of errors or missing values and the initiation of data analysis soon after data collection is completed. It will be used for data management and monitoring all sub-studies, including patient recruitment and follow-up, and is based on the INTERGROWTH-21st Electronic Data Management System. The system permits all participants' data to be incorporated into the data files via the Internet as soon as they are available. Included within the system is a review process to ensure that all data are complete.

All sample related data will be entered separately into a data management system specifically developed for the study. The system allows samples to be tracked from the time of collection through processing, storage in the participating centres, and transport to a centralised facility. Each participant will have a unique identifier number, which will be used to link the clinical and sample databases. The number will also be used to barcode individual samples and aliquots. Quality control for this aspect of the study will be monitored by a team from GAPPS.

These systems will provide the Data Management Unit with a detailed daily record of patient enrolment and data entry, at both individual and institutional levels to monitor progress against the milestones listed in the protocol. Corresponding actions, such as telephone calls, web conferences and site visits will take place within a week of detecting a problem in a centre to ensure that appropriate corrective measures are taken.

---

[d] Others have also used these composite indices of neonatal morbidity (Hannah ME, Hannah WJ Kewson SA et al (2000); Wapner RJ, Sorokin Y, Thom EA (2006); Joseph KS, Fahey J, Platt R (2009)).

For peer review only - http://bmjopen.bmj.com/site/about/guidelines.xhtml

Selection of Cases and Controls All live and stillborn infants in the study hospitals during the data collection period, whether or not they survive until hospital discharge, will be screened. However, multiple births and post-term births (>42^{+0} weeks), will not be included.

Each newborn infant will fall into one of the four groups below:

Set	Infants born <38^{+0} weeks' gestation	Infants born IUGR/SGA	Description	Number of births at study site	Number to be included in the case-control study
A	Yes	No	Non-IUGR/SGA infants born <38^{+0} weeks	A	A (all)
B	No	Yes	IUGR/SGA infants born ≥38^{+0} weeks	B	B (all)
C	Yes	Yes	IUGR/SGA infants born <38^{+0} weeks	C	C (all)
D	No	No	Non-IUGR/SGA infants born ≥38^{+0} weeks	D	Sample = A+B+C

All mothers admitted for delivery (spontaneous or induced labour, or elective C-section) will be screened to check if they had gestational age estimated by CRL at <14 weeks or HC at <24 weeks. If not, they are not eligible for the study. If a mother had one or both of these two measurements, the screening form will be completed to collect the information required to classify her infant as: a) <38^{+0} weeks or ≥38^{+0} weeks, and b) IUGR/SGA or non-IUGR/SGA (based on the charts provided).

Operational definition of cases and controls in the maternity wards

To simplify the identification of cases and controls during screening, the following procedures will be used (see instructions in Appendix II):

First, gestational age will be assessed using CRL or HC. **Cases, born at <38^{+0} weeks, will be live or stillborn infants with gestational age assessed by an early ultrasound (either CRL at <14 weeks or HC at <24 weeks)**, regardless of whether or not they presented with IUGR/SGA at any time during pregnancy or at birth. These infants correspond to groups A and C in the table above.

Second, BW for gestational age will be assessed for infants born ≥38^{+0} weeks. **Cases, IUGR/SGA, will be live or stillbirths whose BW for gestational age is below the 10th centile of the INTERGROWTH-21st neonatal standard as defined on the form.** These infants correspond to group B in the table above. In the data analysis phase, infants from group C (IUGR/SGA infants born <38^{+0} weeks) will be added to those in group B so as to include all IUGR/SGA infants regardless of their gestational age at birth.

Third, the screening form will also identify **potential controls, that is, non-IUGR/SGA infants who were not born <38^{+0} weeks** (group D in the table above). The first potential control born after each case (either a case born <38^{+0} weeks or an IUGR/SGA case) in the same hospital[e] will be enrolled in the study as a control. After enrolling a case, a control must be recruited. If two cases are born in succession, the second case cannot be recruited and instead screening for a control continues. Once a case-control pair have been recruited and processed, sites then screen for another case.

At each site, 200 cases born <38^{+0} weeks and 200 IUGR/SGA cases will be recruited, along with 400 controls. If a site collects 200 cases born <38^{+0} weeks before it has collected 200 IUGR/SGA cases, it will stop recruiting cases born <38^{+0} weeks and their corresponding controls, and will continue recruiting IUGR/SGA cases until 200 (and their controls) have been recruited - and vice-versa, if the quota of 200 IUGR/SGA cases is collected before 200 cases born <38^{+0} weeks are enrolled.

[e] If there is more than one hospital at a given study site, and if presumed risk factors vary by hospital (e.g. one primarily attracts mothers of low socioeconomic status, and another attracts high income mothers), it may be necessary to weight the analyses to reproduce a control group that is representative of the study population; ignoring such differences may lead to overmatching.

For peer review only - http://bmjopen.bmj.com/site/about/guidelines.xhtml

Note that the only criteria for matching cases and controls are: a) hospital of birth and b) approximate date of birth (usually same day, sometimes the next day if there are no controls on the same day).

Figure 4: Neonatal Study Eligibility Flow Diagram

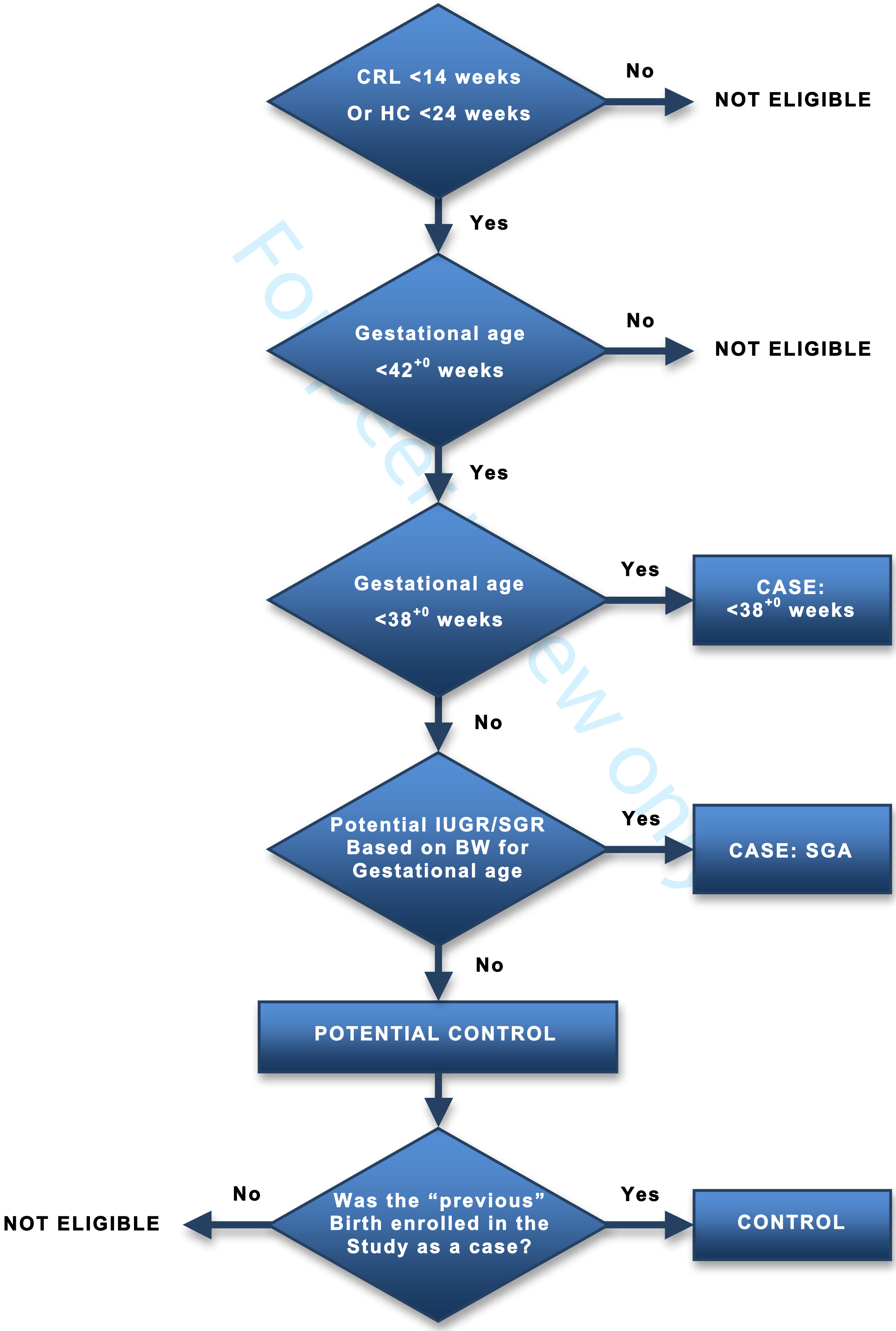

For peer review only - http://bmjopen.bmj.com/site/about/guidelines.xhtml

Definitions of cases and controls for the data analyses

Cases born <38^{+0} weeks' gestation for the data analyses will include all births at <38^{+0} weeks whether or not they present with IUGR/SGA (groups A and C).

IUGR/SGA cases for the data analyses will include the operational definition of IUGR/SGA cases (group B) plus those cases born <38^{+0} weeks who are also IUGR/SGA (group C); the latter were collected as a sub-set of cases born <38^{+0} weeks.

Infants in group C (IUGR/SGA infants born <38^{+0} weeks) will be included in both groups of cases, as the case-control analyses will be carried out separately for each outcome.

The table below provides the definition of controls for the analyses.

Controls for cases born <38^{+0} weeks will be a sample of live and stillborn infants born ≥38^{+0} weeks. In the statistical analyses, a proportion of term IUGR/SGA (xB) cases will be added to the operational controls (group D).

Controls for IUGR/SGA cases will be a sample of live and stillborn infants who are not IUGR/SGA at birth. In the analyses, they will include all operational controls (group D) plus a proportion of infants born <38^{+0} weeks who are not IUGR/SGA at birth(xA).

Table 2. Case-control comparisons in the data analyses.

Comparison	Cases	Controls	Comments
Infants born <38^{+0} weeks case-control study	A + C	D + xB	To reproduce the control population, set B (IUGR/SGA only) will be down-weighted by a factor x which is equal to the sampling fraction for set D, that is the proportion of all infants in the control pool who were included in the detailed study (cases).
IUGR/SGA case-control study	B + C	D + xA	As above, for set A (infants born <38^{+0} weeks only).

Subgroup analyses will include cases born <38^{+0} weeks, stratified according to: a) gestational age groups (the exact groupings will be decided based on the number of births each week of gestational age, so that there will be at least 100 cases in each sub-group) or b) by preterm phenotype, using the newly proposed INTERGROWTH-21st classification system. For IUGR/SGA, subgroup analyses will include stratification by: a) IUGR/SGA severity (<3rd, 3-5th, 6th-9th centiles) and b) gestational age.

Appendix II provides more detailed information on different strategies for selecting controls for case-control studies than we considered when planning the study, but some of these proposed strategies were not practical. As proposed above, INTERBIO-21st will adopt a traditional case-non-case design, and odds ratios will be used to estimate relative risks. This is based on the assumption that cases will be relatively rare, i.e. <10% of the overall number of births.

We estimate that the overall birth rate for infants born <38^{+0} weeks will be <10% and the overall IUGR/SGA rate will be <10-15%. However, by collecting data on all four sets (A, B, C and D), it will also be possible, with appropriate statistical weights in the analyses, to carry out case-base analyses using Poisson regression with robust variance, if the outcomes end up being more common (>10%).

Sample processing

The sample collection, processing and storage procedures will be performed in a standardised manner based on protocols described in detail in the INTERBIO-21st Operations Manual that has been developed with the assistance of the GAPPS team, and researchers at the Universities of Oxford [9] and Cambridge [10], and Johns Hopkins Bloomberg School of Public Health.

In brief, maternal and cord blood samples will be collected to store whole blood, plasma and the buffy coat for a wide range of purposes, including DNA extraction for genetic and epigenetic studies and micronutrient assays. Two placental biopsies will be taken for immunohistochemistry and DNA extraction and in RNA later for expression studies (if the sample is obtained <30 mins after delivery). In addition, we intend to collect and store samples for a number of future, as yet unspecified, biomarker assays relating to preterm delivery and fetal growth.

It is vitally important to ensure that samples are collected in a standardised way with adequate monitoring of quality control, principally because sample quality, quantity and handling can greatly influence the results of microarray and sequencing experiments [11].

For peer review only - http://bmjopen.bmj.com/site/about/guidelines.xhtml

The primary reason (aside from quality control) for ensuring that samples are collected, stored and processed in a uniform manner is to facilitate the anticipated interchange of data, in the future, with other biobanks. Standardising phenotypic definitions, sample collection methods and analyses fosters trans-national collaboration and networking [12]. We shall therefore also seek advice from groups such as the Public Population Project in Genomics (http://www.p3g.org), which promotes international harmonisation and collaboration in population genomics and biobanking by sharing research tools and expertise.

Faecal samples: We wish to collect a faecal sample from mothers, opportunistically at the time of delivery, for metabiomic studies. Although it has been suggested that we should also collect stool samples from infants every 6 months, we feel that this is a rather large-scale undertaking that is beyond our remit.

We certainly appreciate the importance of looking for maternal intestinal co-infections and microbiota. In fact, we published on this subject in 1989: in a prospective study of 14,914 pregnant Guatemalan women, the incidence of IUGR increased with the number of parasitic species detected [13].

Sample size

This is a great challenge in any field-study of this magnitude and even more difficult when exploring risk factors with relatively unknown degrees of association and prevalence in the population. The key issue is to reach a balance between logistical demands, including the need to maintain data quality in these populations, and power calculations especially for the planned epigenetic studies. Having said that, our co-investigators, Krina Zondervan and Cecilia Lindgren in the Wellcome Trust Centre for Human Genetics, Oxford, have considerable experience of conducting candidate gene and genome-wide association studies (GWAS) in related fields and the lessons learned over the last 15 years will be pertinent to the proposed studies.

To illustrate the point, Cardon & Zondervan reviewed how the complex interplay between genotype, phenotype, environmental factors and sample size affects the ability to detect disease susceptibility variants in population-based association studies [14]. They concluded that thousands of cases and controls are required to detect common variants with small effect sizes in such studies.

Three examples demonstrate the need to study large numbers to identify genes influencing quantitative traits involved in metabolic function, such as birth weight. Nearly 120,000 individuals were genotyped to identify three loci influencing anthropometric measures (waist circumference and waist-hip-ratio) of central obesity and fat distribution in a recently published meta-analysis of 16 GWAS, followed by large-scale replication testing [15]. Using similar methodology (GWAS meta-analysis followed by replication), over 90,000 individuals were genotyped to confirm that two loci are associated with BMI and to identify six additional loci [16] and, more recently, nearly 40,000 European individuals were genotyped in identifying variants in *ADCY5* and near *CCNI* associated with fetal growth and birth weight [17].

For the nested case-control studies, we are collecting samples from 2,000 controls; and 2,000 cases from pregnancies with adverse outcomes, e.g. delivery at <38^{+0} weeks' gestation, term IUGR/SGA. In addition, we have the potential to include 400 cases and 2100 controls from the FGLS population in the analysis, taking into consideration the possibility of selection bias in the selection of FGLS population controls.

It is very unlikely that fewer than these numbers will be needed to study the effects of adverse intra-uterine effects on epigenetic profiles, especially as there is emerging evidence from genome-wide epigenetic studies in animals that imprinted quantitative trait loci (iQTL) affect body weight and growth [18] and adult body composition [19] in much more complex and diverse patterns than previously assumed.

Selection of study centresWe aim to use the same rigorous processes to select the new sites for this extension as originally adopted in the selection of the current INTERGROWTH-21st centres. However, in this case, the selection criteria will inevitability involve finding a balance between obvious opportunities (e.g. having access to a malnourished pregnant population with a high prevalence of malaria/HIV) and the risks of working in a research naïve environment with limited existing access to antenatal care.

The criteria the INTERBIO-21st Steering Committee will use to select the centres will include factors such as: 1) existing research infrastructure and capacity; 2) existing maternity services, including antenatal ultrasound; 3) support of local health authorities; 4) previous experience in collecting biological samples; 5) geographical location to retain global coverage; 6) prevalence of key exposure variables, i.e. risk factors; 7) costs; 8) leveraged funding from other donors, and 9) need ideally for all samples in the proof-of-concept study to be analysed in a centralised facility.

For peer review only - http://bmjopen.bmj.com/site/about/guidelines.xhtml

Staged introduction of sample collection at likely study sites

Phase I

Shoklo Malaria Research Unit, Mae Sot, Thailand

KEMRI-Coast Centre for Geographical Medicine & Research, Kilifi, Kenya

John Radcliffe Hospital, Oxford, UK

Phase II

The Aga Khan University Hospital, Nairobi, Kenya

Aga Khan University Medical Centre, Karachi, Pakistan

University of Witwatersrand, Johannesburg, South Africa

Universidade Federal de Pelotas, Pelotas, Brazil

PROGRAMME II: Proof-of-concept study

Background

Understanding the gene-environmental interactions underlying the plasticity of the epigenome at certain times from fetal life to infancy will be crucial to developing interventions, particularly in pregnancy, that might correct or at least prevent the long-term, adverse consequences [20]. We believe that the key to doing so effectively is to recognise that phenotypes other than birth weight and gestational age alone are needed to determine the nutritional status of the newborn and assess the effectiveness of interventions.

The redefinition of newborn phenotypes will arise from evaluating a combination of factors in pregnancies with normal and abnormal outcomes. These include maternal health; fetal growth patterns measured using 2D ultrasound; growth patterns of individual fetal organs measured using 3D ultrasound; newborn body composition and physiological function; micronutrient levels and data from epigenetic experiments, which will initially characterise normal variability across the epigenome in uncomplicated pregnancy and then, in carefully designed nested case-control studies, evaluate the effects of adverse environmental and nutritional factors on the epigenome (and other biomarkers) in a pool of complicated and uncomplicated pregnancies.

General Objectives

The aim is to conduct a hypothesis-testing, proof-of-concept study comparing 500 normal birth weight and 500 term IUGR/SGA newborns (using both cord blood and placental samples) taken from the samples collected in the context of both the INTERBIO-21st Fetal and Newborn Studies. This will be the first in a series of experiments utilising samples collected for the INTERBIO-Bank.

We aim to assess DNA methylation patterns in ~100 imprinted genes previously implicated in fetal growth. Our hypothesis is that maternal micronutrient deficiency, particularly of folate and other methyl donor factors, results in impaired fetal growth, development and pregnancy outcomes, through altered DNA methylation.

We will therefore correlate these methylation patterns with pregnancy (clinical outcomes, fetal growth), nutritional (micronutrient assays), and neonatal (growth, development and body composition) data, which will allow us to:

1. Study the effects of environmental and nutritional factors on the epigenome;
2. Develop new phenotypic definitions of LBW and other adverse pregnancy outcomes

If validated, the results could inform knowledge-based actions to address underlying problems, such as poor nutrition and infection, leading to improved outcomes. The data will, in addition, serve to define normal variability in the epigenome and inform the design of future epigenome-wide studies, once the cost has fallen, as inevitably it will with technological advances.

In the long-term, we would also wish to correlate these epigenetic findings with single nucleotide polymorphism (SNP) genotyping data from a GWAS given the increasing evidence that epigenetic regulation is influenced by genetic factors and the recently published data implicating variants in *ADCY5* and near CCNI with fetal growth and birth weight [17].

For peer review only - http://bmjopen.bmj.com/site/about/guidelines.xhtml

Specific Objectives
We plan to study the methylation profiles of the ~100 imprinted genes that have to date been implicated in fetal growth, although the final list of candidate genes will be taken from our own systematic search of the literature, as well as existing databases, such as http://www.geneimprint.com and http://igc.otago.ac.nz.

Where possible, we will analyse cord blood and placental tissue separately to compare the methylation profiles of both tissues. The underlying rationale is as follows:

- There is increasing evidence that placental function and gene expression respond to, and are marked by, environmental insults. The placenta can therefore serve as a 'record of *in utero* exposure and pathology' [21]. Effects on the fetus almost certainly occur downstream of these events and so comparing the epigenetic profiles of both tissues in individual pregnancies may help to differentiate the various causes of IUGR/SGA and preterm delivery.
- Alterations in DNA methylation in humans appear to be tissue-specific:
    - Katari et al. (2009) have reported significantly different DNA methylation levels at specific CpG sites between cord blood and placenta [22].
    - Guo et al. (2008) have described similar findings in two imprinting clusters: the H19 promoter is unmethylated and IGF2 DMR2 hypomethylated in placenta. However, in cord blood, these two regions maintain the differential methylation status seen in most other tissues [23].
    - Yuen et al. (2009) have observed DNA methylation of the promoter in *TUSC3* and *WNT2* in placental, and not the associated fetal, tissues; within individual placentas, methylation was confined to trophoblastic chorionic villi, and not amnion, chorion, cord or decidua [24].

Study design

For this proof-of-concept study, we will randomly select 500 term IUGR/SGA cases from the INTERBIO-Bank. The 500 normal birth weight controls will be taken either from the population at least risk within the INTERBIO-21st Fetal Study or from the total with normal outcomes from the entire study population, and matched with the cases. A final decision will be made by the INTERBIO-21st Steering Committee.

Methods
We have given considerable thought to the best technological platform for assessing methylation profiles and we have consulted widely with leading experts in the scientific community and industry. There are a large number of different platforms available and many more being developed; in general, there is an inverse relationship between the cost of analysis and the resolution/coverage of the genomic region being studied. At this stage, however, we have decided to use Methylated-Cytosine DNA Immunoprecipitation-Microarray Chip (MeDIP-Chip) followed by bisulfite-(BS) PCR and high throughput sequencing for validation of differentially methylated loci [11].

The approach is well described in a recently published proof-of-concept study assessing whether 'DNA methylation in a subset of genomic loci may connect end-stage cardiomyopathy with different etiologies' [25]. In brief, these authors performed a preliminary analysis using MeDIP-Chip (Nimblegen, WI, US); validated differential methylation loci by BS-PCR and high throughput sequencing; identified three angiogenesis-related genetic loci that were differentially methylated with the BATMAN algorithm [26], and using quantitative RT-PCR, found that the expression of these genes differed significantly between cardiomyopathy hearts and normal controls.

However, we are aware that the samples may not be analysed for at least another two years by which time the technology is likely to have changed considerably, costs will have fallen and genome-wide profiling in large numbers of samples will be affordable. We are therefore in preliminary discussions with a number of companies, including Nanopore (Oxford, UK), http://www.nanoporetech.com, and Pacific Biosciences (Menlo Park, CA, US), http://www.pacificbiosciences.com, who may soon be able to offer high-throughput, single molecule sequencing [27]. Whichever platform is used, however, the intention ideally is to analyse all samples in a centralised facility; in fact, this applies to all the experiments proposed in the proof-of-concept study.

Specific experiments

Placenta v. cord blood methylation profiles: To the best of our knowledge, no study has compared the methylation profiles of the ~100 imprinted genes in placental tissue and cord blood. The outcomes of these comparisons will potentially shed light on the regulatory mechanisms and epigenetic profiles of adverse and healthy pregnancy outcomes.

For peer review only - http://bmjopen.bmj.com/site/about/guidelines.xhtml

Cases v. controls methylation profiles: The results of the placenta v. cord blood studies will help to determine which sample sets are compared in trying to identify the methylation profiles associated with adverse pregnancy and newborn outcomes. Comparisons will also be made between ethnic sub-groups. All the above experiments will be performed in duplicate with adequate quality control measures,

Sample pooling for methylation profiles: Pooling samples of 'healthy' controls to act as a reference standard for epigenetic studies has been proposed in the literature [11]. Given that FGLS provides an ideal opportunity to use samples from newborns whose intra-uterine growth has been optimal, we plan to explore this possibility with FGLS samples drawn from the three INTERGROWTH-21st centres. This might involve pooling samples collected both within and across these centres, although the experiments would need to be performed in India if samples are collected there.

Placental expression analyses: We will follow the same experimental design outlined in the Movassagh et al. (2010) study [26]. Quantitative real-time PCR will be performed for target genes, selected from the methylation studies, using validated Taqman Gene Expression Assay primers (Applied Biosystems, Foster City, CA) normalised against house-keeping gene data. In the long-term, we also plan to characterise global expression patterns in placental tissue using the new Illumina HT-12 v4 expression chip, for comparison between sub-groups and methylation profiles, as well as between normal and adverse pregnancy and newborn outcomes.

Nutritional status

To supplement the epigenetic studies above, we will also assess the nutritional status of the 500 cases and 500 controls selected for the proof-of-concept study, by measuring:

- Micronutrients in maternal blood at booking and cord blood at delivery
- Putative markers of methyl donation, e.g. S-adenosylmethionine (SAM) to S-adenosyl-homocysteine (SAH) ratio
- Neonatal body composition

The rationale for adding these measures is that they should facilitate the interpretation of the epigenetic data and the characterisation of specific sub-phenotypes, in particular IUGR and SGA.

Micronutrient assays: As with the epigenetic studies, there are a large number of technological platforms available to assay micronutrients and some controversy regarding the most appropriate ones to measure. The assessment of micronutrients in mother's blood is made even more complex by physiological alterations such as haemodilution and the hyperlipidaemic state of pregnancy [28 29].

We will therefore seek guidance from the Biomarker Group consisting of experts in the field before finalising the list of analytes and the methods to use. We will also draw heavily on the expertise of our collaborators at SMRU, Thailand who have considerable experience of assessing nutritional status in their populations. At present, based on unpublished data from their studies and our reading of the literature, the following analytes have been proposed as candidates to measure:

- Retinol Binding Protein (RBP)/Vitamin A
- Iodine (maternal) and TSH (newborn)
- Ferritin and Soluble Transferrin Receptor (sTfR) – markers of Fe deficiency
- Zinc protoporphyrin (ZnPP)
- Folate, thiamine, choline and zinc
- Vitamin D
- CRP and αGP

In Thailand, we will also measure Dichloro-Diphenyl-Trichloroethane (DDT) metabolite levels as DDT was used as an insecticide for malaria control in Northern Thailand until it was replaced by Deltamethrin in 2000. However, high serum DDT residues, which affect serum retinol levels and probably thiamine as well, are still detected in pregnant women living in the Mae La camp [30] We also plan to ask the local investigators to identify other possible chemical exposures to measure. Final decisions about which exposures to measure and where the samples will be analysed will be made by the Biomarker Group; however, we will ideally use centralised facilities.

Neonatal body composition: As part of our Wellcome Trust/EPSRC funded research program, we are already starting to measure neonatal body composition in: a) term normal birth weight, b) preterm and c) term IUGR/SGA infants enrolled in the UK component of FGLS and PPFS. To do so, we are using an infant-sized, air-displacement plethysmograph (PEA POD Infant Body Composition System, Life Measurement, Concord, CA, US). The study is being conducted so as to correlate fetal growth patterns with better measures than birth weight alone, i.e. the relative contributions of body fat, lean tissues and bone, all of which are key indicators of the adequacy of intra-uterine nutrition.

For peer review only - http://bmjopen.bmj.com/site/about/guidelines.xhtml

The PEA POD system compares well with the 4-compartment reference model, which is considered the best choice for assessing body composition in humans. In contrast, however, it is easy to perform; takes only a few minutes to complete; infant movement during the measurement is not a significant problem; the measurements can be repeated as frequently as needed, and the results are immediately available [31].

The system is now recognised as an established method to assess neonatal body composition in developed countries [32-34], and it is suggested that it may offer important insights into which fetal growth parameters most closely reflect the generalised nutritional state of neonates and infants [34]. However, there are no published data about its use in resource-poor settings as, to the best of our knowledge, the system has been installed in only one site in such a setting, as part of a collaboration between Jimma University, Ethiopia, and the Department of Human Nutrition, University of Copenhagen.

We now propose installing PEA POD systems in four of the centres in resource-poor settings to give a much more detailed assessment of nutritional status and growth than birth weight and gestational age alone.

Sample size for epigenetic studies

As discussed on page 18, it is extremely difficult to provide reliable power calculations at the moment for epigenetic studies: the field is too new and very few relevant studies have been conducted, especially in humans, to enable power calculations to be performed. It is also unclear at present to what extent it will be necessary to map DNA methylation at high resolution across the entire genome [35], which will inevitably influence the epigenotyping strategy and choice of platform, e.g. bisulfite sequencing or array-based technology. However, having said that, the sample size chosen matches that in the NIH National Standard for Normal Fetal Growth Study and we feel comfortable that it provides a reasonable compromise between cost, expediency and logistical demands.

The estimated samples sizes required to detect the effects of methylation status on adverse pregnancy outcomes are inevitably based on a range of assumptions, since the spectrum of methylation changes and their corresponding effect sizes are unknown. Table 1 shows the sample sizes required to detect differential methylation in cases vs. controls. The following assumptions are made:

- Methylation status is either on/off, and so the proportion of cases vs. controls with methylated status is analysed.
- Methylation proportion among controls of 0.2, with proportion in cases varying from 0.3-0.5, corresponds to an odds ratios (OR) of the effect of methylation status on outcome from 1.7-4.0.
- A significance threshold α of 5.0×10^{-4} (Bonferroni-corrected threshold for 100 candidate imprinted genes) vs. 5.0×10^{-7} (commonly applied genome-wide significance threshold in GWA studies [36]).
- Power of 80% vs. 90%
- Case: control ratio either 1:1 or 1:3

For peer review only

Table 1. Sample sizes* to detect differential methylation status between cases and controls

				Sample size for candidate gene study (α=5.0x10^{-4})		Sample size for genome-wide study (α=5.0x10^{-7})	
	methylation proportion among controls	methylation proportion among cases	OR (PAF)**	Ca:Co 1:1	Ca:Co 1:3	Ca:Co 1:1	Ca:Co 1:3
Power=80%	0.2	0.3	1.71 (0.12)	719	459	1313	913
		0.35	2.15 (0.19)	342	215	623	388
		0.4	2.67 (0.25)	204	127	370	260
		0.45	3.27 (0.32)	137	85	248	151
		0.5	4.00 (0.38)	99	61	179	125
Power=90%	0.2	0.3	1.71 (0.12)	847	559	1512	966
		0.35	2.15 (0.19)	412	263	716	451
		0.4	2.67 (0.25)	245	155	425	265
		0.45	3.27 (0.32)	164	103	284	175
		0.5	4.00 (0.38)	118	74	204	126

* Sample size for cases is given. Calculations include a continuity correction allowing for normal approximation of the binomial distribution.
** OR = odds ratio; PAF = population attributable fraction

Table 1 demonstrates the approximate power of the proposed experiments with 500 cases and 500 controls. However, for future experiments, based on conservative estimates (OR=2.2 and PAF=0.2), we will have considerable power to detect differences even for 90% power, given that we could have a 1:3 case: control ratio (i.e. 1,000 infants born at <38^{+0} weeks' gestation or 1,000 term IUGR/SGA newborns and at least 3,000 term, non-IUGR/SGA controls). Nevertheless, it is worth emphasizing that these are approximate calculations and that, in a study of this magnitude and complexity, logistical and budgetary considerations must inevitably play an important role in the selection of the sample size.

Data quality: Standardisation of the research staff, who will be responsible for obtaining the neonatal body composition data, represents a challenge. However, we will employ the same quality control measures that are now being used in FGLS and PPFS for the ultrasound and anthropometric data to ensure that the quality of the data is maintained.

Publications and Authorship

The policy regarding publications arising from the study is identical to that in place for the INTEGROWTH-21st Project as a whole and was approved at the first INTERBIO-21st Study Steering Committee.

For peer review only

Appendix I:

We are aiming to collect:
200 Cases <38^{+0} weeks, including ALL babies delivered at <36^{+0} weeks and 200 corresponding controls
200 SGA Cases, including ALL babies delivered with a birth weight <3rd percentile and 200 corresponding controls

Instructions for recruiting Cases and Controls for the Neonatal Study

Every day an INTERBIO-21st midwife/researcher will screen all women on the labour ward and those scheduled for a Caesarean section using the tablet computer provided. The tablet is programmed with an algorithm incorporated into a simple, user friendly application which, when completed, will select the correct proportion of cases and controls by gestational age and birth weight. This removes the need for the user to make a decision and keeps the user blind to who is recruited as a case or a control. The proportion of cases and controls eligible for the study by gestational age is as follows:

Gestational age (GA) or birth weight BW) for GA percentile (P)	% to be recruited	Case/Control
GA <36^{+0} weeks (up to and including 35^{+6})	100%	Preterm case
GA 36^{+0} to 36^{+6}	50%	Preterm case
GA 37^{+0} to 37^{+6}	5%	Preterm case
BW/GA <P3	100%	SGA case
BW/GA P3 to P9.9	50%	SGA case
GA 38^{+0} to 41^{+6} weeks and BW/GA ≥ P10	Will vary according to the number of cases recruited	Potential controls, who are to be sampled immediately after each case.

3rd percentile (P3):

Weeks	≥36^{+0}≤36^{+6}	≥37^{+0}≤37^{+6}	≥38^{+0}≤38^{+6}	≥39^{+0}≤39^{+6}	≥40^{+0}≤40^{+6}	≥41^{+0}≤41^{+6}
**Cut-off value**	2000g	2200g	2300g	2450g	2600g	2700g

10th percentile (P10):

Weeks	≥36^{+0}≤36^{+6}	≥37^{+0}≤37^{+6}	≥38^{+0}≤38^{+6}	≥39^{+0}≤39^{+6}	≥40^{+0}≤40^{+6}	≥41^{+0}≤41^{+6}
**Cut-off value**	2300g	2450g	2600g	2750g	2900g	3000g

The midwife/researcher using the tablet will approach and screen all women. For an eligible woman, he/she will ensure that consent has been acquired, then recruit the woman and collect biological samples. Some descriptive information, including age, parity and schooling will be collected on all women who are screened using the tablet, whether they are enrolled in the study or not. The midwife/researcher should aim to recruit as many women as possible each day given the circumstances on the labour ward and the capacity of the laboratory. The numbers of cases and controls recruited each day will be site specific.

For peer review only - http://bmjopen.bmj.com/site/about/guidelines.xhtml

Appendix II:

Technical note on selection of controls by Prof Cesar Victoria

Selection of appropriate controls in case-control studies is one of the most complex issues in epidemiological design, and also one in which recent progress has obliterated pre-existing ideas, in particular the notion that controls had to be "healthy" or "normal"[6]. There are currently two key concerns in the selection of controls. First, controls should represent the population from which the cases were selected. This will ensure internal validity of the study by avoiding selection bias. It is not required that controls should be healthy in all respects, because in the population where the cases came from there will be unhealthy subjects (for example, controls born at $<38^{+0}$ weeks' gestation may be IUGR). Second, control selection should be driven by the epidemiological measure of effect that one wishes to estimate. In aetiological research, the most appropriate measure of effect is the incidence density ratio (IDR), or rate ratio, which is equal to the ratio between the incidence rates in the exposed and unexposed groups. Nevertheless, it is not always possible to estimate the IDR directly in case-control designs, and feasibility considerations may lead to other approaches for selecting controls.

There are three main types of case-control studies, which differ according to the type of controls. If the outcome being studied is relatively rare (say, 10% or less), then the three types of controls produce similar results (see attached spreadsheet, "INTERBIO control selection.xls"). Nevertheless, delivery rates at <38 weeks' gestation could be above 10% in some study sites.

Birth study on infants born at $<38^{+0}$ weeks' gestation

Below are three potential methods for selecting controls for births $<38^{+0}$ weeks' gestation.

Case-concurrent design
Data from the Fetal Study allow adopting the case-concurrent method. If information on exposure (for example questionnaire-based exposure variables) is available for all mothers in the fetal growth study, there is no need to do a case-control analysis, because one will already have data on the whole cohort of pregnancies. The data can be analysed with standard cohort analyses (e.g. Cox regression) where the denominator is fetus-weeks-at-risk. If obtaining information on exposure for the whole cohort is too expensive (e.g. GWAS, single SNPs or some biomarkers) then one can do nested case-control analyses.[7] In this design, whenever a birth at $<38^{+0}$ weeks' gestation occurs, the next woman attending for antenatal care or ultrasound examination, with the same gestational age, would be selected as a matched control. This design has the advantage of estimating IDR directly, whether or not the outcome (birth at $<38^{+0}$ weeks' gestation) is common. The main disadvantage is that the study cases and controls would be restricted to women who comply with the entrance criteria for the study, and who attend antenatal care frequently. This approach may leave out many of the high-risk women who would not comply with these strict criteria, and as a consequence the study may miss out on important risk factors.

Case-non-case design
Non-case controls in the study will include infants born $\geq 38^{+0}$ weeks' gestation, regardless of whether or not they present IUGR. Both cases and controls will be selected in the Neonatal Study. Because there are many more potential controls than cases, controls will be sampled to improve the efficiency of the study, and to avoid carrying out expensive tests on all non-cases. A detailed discussion of the approach to selecting such controls is available in the body of this protocol. The case-non-case design is easy to explain to a wider audience than the other two designs discussed here, and it will provide an estimate of the odds ratios associated with specific exposures, which is a good estimate of the IDR when rates of delivery $<38^{+0}$ weeks' gestation is relatively low, but will overestimate the IDR if the delivery rate $<38^{+0}$ weeks is high (see attached spreadsheet, "INTERBIO control selection.xls"). Logistic regression is the method of choice for analyzing case-non-case designs.

Case-base design

---

[6] Olsen, J., Cesar, V., Ebrahim, S., Pearce, N. The idea of the healthy control is sick. Available at: http://www.ieaweb.org/index.php?option=com_content&view=article&id=62:the-idea-of-the-healthy-control-is-sick&catid=22:rapid-response&Itemid=54 [accessed 25/07/2011]

[7] For exposures that will also be collected in the Neonatal Study, it will be possible to carry out separate analyses in the Fetal and Neonatal studies, and compare their results; if results are similar, the validity of the findings will be enhanced.

© University of Oxford

For peer review only - http://bmjopen.bmj.com/site/about/guidelines.xhtml

In this design, controls are sampled from all pregnant women, including those who delivered <38^{+0} weeks' gestation. The latter women will therefore be included as both cases (all women with delivery at <38^{+0} weeks' gestation) and controls (a sample of these women, using the same sampling fraction as that of women with a delivery age >38^{+0} weeks). The case-base design estimates the prevalence ratio – it is important to remember that prevalence is obtained by dividing subjects with a given characteristic (for example, birth at <38^{+0} weeks' gestation) by the whole population, which includes all births. This justifies the inclusion of women with deliveries at <38^{+0} weeks' gestation in the control group as well. Prevalence ratios obtained from a case-base design tend to overestimate the IDR for births at <38^{+0} weeks' gestation (see attached spreadsheet, "INTERBIO control selection.xls"), particularly when the rate of delivery at <38^{+0} weeks' gestation is high. By collecting data on the four subgroups of births (A, B, C and D) as described in the body of this protocol, it is possible to use weighting to reproduce a case-base analysis. Analyses of case-base designs may be carried out using Poisson regression with robust variance.

IUGR study

Below are three potential methods for selecting controls for IUGR births. The same principles discussed above for the design for births at <38^{+0} weeks' gestation, also apply here, with a few modifications. Unlike births at <38^{+0} weeks' gestation - which would be defined equally in the fetal and neonatal studies – IUGR would have different definitions, as discussed below.

Case-concurrent design

In the Fetal Study with serial ultrasound measurements, it may be possible to identify the approximate time at which a fetus became growth-restricted, and use a case-concurrent design. Controls would be fetuses who are not growth-restricted at the time their corresponding cases start faltering. This design is a theoretical possibility, but in practice it may be hard to pinpoint the exact gestational age at which faltering started, and it would also be necessary to decide how to handle fetuses with temporary faltering followed by catch-up growth, and whose weight for gestational age goes back to the normal range. Therefore, although such studies are possible in theory, they are unlikely to be feasible. In addition, as mentioned above in the context of the case-concurrent design for deliveries at <38^{+0} weeks' gestation, the sample of women with frequent measurements in the Fetal Study is likely biases, and may exclude high-risk pregnancies which are less likely to attend antenatal care frequently.

Case-non-case design

Both for case-non-case and for case-base designs, the cases would include IUGR infants at birth, defined as BW/GA <10th centile. In the case-non-case design, controls would be a sample of all infants who do not present IUGR at birth. The measure of effect would be the odds ratio, which overestimates the IDR and the prevalence ratio when IUGR prevalence exceeds 10% (see attached spreadsheet, "INTERBIO control selection.xls"). Such high rates are common in some parts of the world such as South Asia and Central America.

Case-base design

IUGR at birth is a point prevalence measure, more specifically the proportion of all babies who are born with low weight for their gestational age. For example, 12% of all newborns in a population may present IUGR - note that the denominator of the prevalence measure includes births with and without IUGR. The case-base design directly estimates the prevalence ratio, because the control group includes a sample of all births, regardless of their gestational age at delivery or IUGR status. It may be argued that for IUGR the prevalence ratio is a better measure than the IDR, in particular given how hard it is to define the precise incidence and timing of IUGR onset (as discussed above under the case-concurrent design).

Conclusions

By collecting data on the four subgroups of births (A, B, C and D) as described in the body of this protocol, it is possible to carry out both case-non-case and case-base analyses in the Neonatal Study. We are proposing for logistical reasons that the Neonatal Study controls should be initially selected from non-cases and that the primary analyses should entail case-non-case comparisons. However, it is equally possible to analyse the data with a case-base comparison, by using statistical weighting to correct for the over-sampling of infants born <38^{+0} weeks' gestation and those who are IUGR, and therefore reproducing the whole population of births.

© University of Oxford

For peer review only - http://bmjopen.bmj.com/site/about/guidelines.xhtml

If information on exposures (e.g. through a questionnaire) is available for all births in the Neonatal Study, then it is possible to carry out a direct analysis of prevalence ratios, without the need for sampling controls. On the other hand, for exposures that are expensive to measure (e.g. lab tests) then sampling controls is a necessity.

Appendix III:

Definition of intrauterine growth restriction in field studies

A specific limitation of the anthropometric definition of IUGR is the fact that some small babies are biologically small, yet healthy. This could theoretically be overcome by incorporating putative biological or physical markers associated with IUGR/SGA to improve the definition, and in doing so, potentially separate those newborns that are biologically small (yet healthy) from those that are true IUGR/SGA. However, this may be a less relevant issue for the high-risk and undernourished populations we are planning to study where the proportion of the total IUGR/SGA population, that is composed of "healthy" small IUGR/SGA newborns is tiny, compared to a healthy well-nourished western population where biologically "small" babies can represent an important proportion of the IUGR population.

Furthermore, we believe there is not enough evidence presently that such markers can differentiate IUGR/SGA sub-groups sufficiently to justify their incorporation in the planned field studies, especially as these are taking place predominantly in developing countries. For example, it has been suggested that 1st trimester Doppler ultrasound has a role in distinguishing some of the etiologies of IUGR/SGA. In our opinion, even if these findings are eventually confirmed in a large-scale RCT and shown to be associated with a reduction in perinatal mortality, it would simply not be practical to introduce these additional measurements in the study sites we are planning to use and on the scale of our prospective data collection. Other biological markers that have been proposed as a way of characterising IUGR/SGA better are still being assessed and, therefore, one of the aims of the new study is to contribute to the evaluation of such markers in IUGR/SGA.

For peer review only

For peer review only - http://bmjopen.bmj.com/site/about/guidelines.xhtml

_ENREF_37_ENREF_41_ENREF_48_ENREF_49_ENREF_49_ENREF_56_ENREF_57_ENREF_58**References:**

1. Hofmeyr GJ, Atallah AN, Duley L. Calcium supplementation during pregnancy for preventing hypertensive disorders and related problems. *Cochrane Database Syst Rev* 2006;3:CD001059.

2. Barros FC, Bhutta ZA, Batra M, Hansen TN, Victora CG, Rubens CE. Global report on preterm birth and stillbirth (3 of 7): evidence for effectiveness of interventions. *BMC Pregnancy Childbirth* 2010;10 Suppl 1:S3.

3. Garner P, Gulmezoglu AM. Drugs for preventing malaria in pregnant women. *Cochrane Database Syst Rev* 2006(4):CD000169.

4. Simcox R, Sin WT, Seed PT, Briley A, Shennan AH. Prophylactic antibiotics for the prevention of preterm birth in women at risk: a meta-analysis. *Aust N Z J Obstet Gynaecol* 2007;47(5):368-77.

5. Swadpanich U, Lumbiganon P, Prasertcharoensook W, Laopaiboon M. Antenatal lower genital tract infection screening and treatment programs for preventing preterm delivery. *Cochrane Database Syst Rev* 2008(2):CD006178.

6. Mook-Kanamori DO, Steegers EA, Eilers PH, Raat H, Hofman A, Jaddoe VW. Risk factors and outcomes associated with first-trimester fetal growth restriction. *JAMA* 2010;303(6):527-34.

7. Villar J, Valladares E, Wojdyla D, Zavaleta N, Carroli G, Velazco A, et al. Caesarean delivery rates and pregnancy outcomes: the 2005 WHO global survey on maternal and perinatal health in Latin America. *Lancet* 2006;367(9525):1819-29.

8. Villar J, Abalos E, Carroli G, Giordano D, Wojdyla D, Piaggio G, et al. Heterogeneity of perinatal outcomes in the preterm delivery syndrome. *Obstet Gynecol* 2004;104(1):78-87.

9. Redman CW, Sargent IL. Circulating microparticles in normal pregnancy and pre-eclampsia. *Placenta* 2008;29 Suppl A:S73-7.

10. Pasupathy D, Dacey A, Cook E, Charnock-Jones DS, White IR, Smith GC. Study protocol. A prospective cohort study of unselected primiparous women: the pregnancy outcome prediction study. *BMC Pregnancy Childbirth* 2008;8:51.

11. Thu KL, Pikor LA, Kennett JY, Alvarez CE, Lam WL. Methylation analysis by DNA immunoprecipitation. *J Cell Physiol* 2009;222(3):522-31.

12. Burton PR, Hansell AL, Fortier I, Manolio TA, Khoury MJ, Little J, et al. Size matters: just how big is BIG?: Quantifying realistic sample size requirements for human genome epidemiology. *Int J Epidemiol* 2009;38(1):263-73.

13. Villar J, Klebanoff M, Kestler E. The effect on fetal growth of protozoan and helminthic infection during pregnancy. *Obstet Gynecol* 1989;74(6):915-20.

14. Zondervan KT, Cardon LR. The complex interplay among factors that influence allelic association. *Nat Rev Genet* 2004;5(2):89-100.

15. Lindgren CM, Heid IM, Randall JC, Lamina C, Steinthorsdottir V, Qi L, et al. Genome-wide association scan meta-analysis identifies three Loci influencing adiposity and fat distribution. *PLoS Genet* 2009;5(6):e1000508.

16. Willer CJ, Speliotes EK, Loos RJ, Li S, Lindgren CM, Heid IM, et al. Six new loci associated with body mass index highlight a neuronal influence on body weight regulation. *Nat Genet* 2009;41(1):25-34.

17. Freathy RM, Mook-Kanamori DO, Sovio U, Prokopenko I, Timpson NJ, Berry DJ, et al. Variants in ADCY5 and near CCNL1 are associated with fetal growth and birth weight. *Nat Genet* 2010.

© University of Oxford

For peer review only - http://bmjopen.bmj.com/site/about/guidelines.xhtml

18. Wolf JB, Cheverud JM, Roseman C, Hager R. Genome-wide analysis reveals a complex pattern of genomic imprinting in mice. *PLoS Genet* 2008;4(6):e1000091.

19. Cheverud JM, Hager R, Roseman C, Fawcett G, Wang B, Wolf JB. Genomic imprinting effects on adult body composition in mice. *Proc Natl Acad Sci U S A* 2008;105(11):4253-8.

20. Gluckman PD, Hanson MA, Bateson P, Beedle AS, Law CM, Bhutta ZA, et al. Towards a new developmental synthesis: adaptive developmental plasticity and human disease. *Lancet* 2009;373(9675):1654-7.

21. Maccani MA, Marsit CJ. Epigenetics in the placenta. *Am J Reprod Immunol* 2009;62(2):78-89.

22. Katari S, Turan N, Bibikova M, Erinle O, Chalian R, Foster M, et al. DNA methylation and gene expression differences in children conceived in vitro or in vivo. *Hum Mol Genet* 2009;18(20):3769-78.

23. Guo L, Choufani S, Ferreira J, Smith A, Chitayat D, Shuman C, et al. Altered gene expression and methylation of the human chromosome 11 imprinted region in small for gestational age (SGA) placentae. *Dev Biol* 2008;320(1):79-91.

24. Yuen RK, Avila L, Penaherrera MS, von Dadelszen P, Lefebvre L, Kobor MS, et al. Human placental-specific epipolymorphism and its association with adverse pregnancy outcomes. *PLoS One* 2009;4(10):e7389.

25. Movassagh M, Choy MK, Goddard M, Bennett MR, Down TA, Foo RS. Differential DNA methylation correlates with differential expression of angiogenic factors in human heart failure. *PLoS One* 2010;5(1):e8564.

26. Issa JP. CpG island methylator phenotype in cancer. *Nat Rev Cancer* 2004;4(12):988-93.

27. Howorka S, Cheley S, Bayley H. Sequence-specific detection of individual DNA strands using engineered nanopores. *Nat Biotechnol* 2001;19(7):636-9.

28. Seshadri S. Prevalence of micronutrient deficiency particularly of iron, zinc and folic acid in pregnant women in South East Asia. *Br J Nutr* 2001;85 Suppl 2:S87-92.

29. Black RE. Micronutrients in pregnancy. *Br J Nutr* 2001;85 Suppl 2:S193-7.

30. Stuetz W, McGready R, Cho T, Prapamontol T, Biesalski HK, Stepniewska K, et al. Relation of DDT residues to plasma retinol, alpha-tocopherol, and beta-carotene during pregnancy and malaria infection: a case-control study in Karen women in northern Thailand. *Sci Total Environ* 2006;363(1-3):78-86.

31. Ellis KJ, Yao M, Shypailo RJ, Urlando A, Wong WW, Heird WC. Body-composition assessment in infancy: air-displacement plethysmography compared with a reference 4-compartment model. *Am J Clin Nutr* 2007;85(1):90-5.

32. Eriksson B, Lof M, Forsum E. Body composition in full-term healthy infants measured with air displacement plethysmography at 1 and 12 weeks of age. *Acta Paediatr* 2010;99(4):563-8.

33. Carberry AE, Colditz PB, Lingwood BE. Body composition from birth to 4.5 months in infants born to non-obese women. *Pediatr Res* 2010.

34. Lee W, Balasubramaniam M, Deter RL, Hassan SS, Gotsch F, Kusanovic JP, et al. Fetal growth parameters and birth weight: their relationship to neonatal body composition. *Ultrasound Obstet Gynecol* 2009;33(4):441-6.

35. Bock C, Walter J, Paulsen M, Lengauer T. Inter-individual variation of DNA methylation and its implications for large-scale epigenome mapping. *Nucleic Acids Res* 2008;36(10):e55.

For peer review only - http://bmjopen.bmj.com/site/about/guidelines.xhtml

36. WTCCC. Genome-wide association study of 14,000 cases of seven common diseases and 3,000 shared controls. *Nature* 2007;447(7145):661-78.

For peer review only

For peer review only - http://bmjopen.bmj.com/site/about/guidelines.xhtml

STROBE Statement—Checklist of items that should be included in reports of *cohort studies*

Name of Study: The INTERGROWTH-21st Project International INTER-NDA standards for child development at two years of age: An International Prospective Population-based Study

Corresponding author: Michelle Fernandes, E: michelle.fernandes@wrh.ox.ac.uk

	Item No	Recommendation	Provided	Pg. No. references
**Title and abstract**	1	(*a*) Indicate the study's design with a commonly used term in the title or the abstract	✓	1
		(*b*) Provide in the abstract an informative and balanced summary of what was done and what was found	✓	1
**Introduction**				
Background/rationale	2	Explain the scientific background and rationale for the investigation being reported	✓	4-5
Objectives	3	State specific objectives, including any prespecified hypotheses	✓	1, 4-5
**Methods**				
Study design	4	Present key elements of study design early in the paper	✓	1, 4-5, 6
Setting	5	Describe the setting, locations, and relevant dates, including periods of recruitment, exposure, follow-up, and data collection	✓	1, 4-5, 6
Participants	6	(*a*) Give the eligibility criteria, and the sources and methods of selection of participants. Describe methods of follow-up	✓	1, 6
		(*b*) For matched studies, give matching criteria and number of exposed and unexposed	n/a	n/a
Variables	7	Clearly define all outcomes, exposures, predictors, potential confounders, and effect modifiers. Give diagnostic criteria, if applicable	✓	1, 7
Data sources/ measurement	8*	For each variable of interest, give sources of data and details of methods of assessment (measurement). Describe comparability of assessment methods if there is more than one group	✓	7, 8, 9
Bias	9	Describe any efforts to address potential sources of bias	✓	7, 8, 9
Study size	10	Explain how the study size was arrived at	✓	8
Quantitative variables	11	Explain how quantitative variables were handled in the analyses. If applicable, describe which groupings were chosen and why	✓	8, 9
Statistical methods	12	(*a*) Describe all statistical methods, including those used to control for confounding	✓	8, 9
		(*b*) Describe any methods used to examine subgroups and interactions	✓	8, 9
		(*c*) Explain how missing data were addressed	✓	8, 9
		(*d*) If applicable, explain how loss to follow-up was addressed	✓	8, 9
		(*e*) Describe any sensitivity analyses	✓	8, 9
**Results**				
Participants	13*	(a) Report numbers of individuals at each stage of study—eg numbers potentially eligible, examined for eligibility, confirmed eligible, included in the study, completing follow-up, and analysed	✓	Fig 2, pg 10

For peer review only - http://bmjopen.bmj.com/site/about/guidelines.xhtml

		(b) Give reasons for non-participation at each stage	✓	Fig 2, pg 10
		(c) Consider use of a flow diagram	✓	Fig. 2
Descriptive data	14*	(a) Give characteristics of study participants (eg demographic, clinical, social) and information on exposures and potential confounders	✓	Fig 2, pg 10
		(b) Indicate number of participants with missing data for each variable of interest	✓	Fig 2, pg 10
		(c) Summarise follow-up time (eg, average and total amount)	✓	Fig 2, pg 10
Outcome data	15*	Report numbers of outcome events or summary measures over time	✓	10
Main results	16	(*a*) Give unadjusted estimates and, if applicable, confounder-adjusted estimates and their precision (eg, 95% confidence interval). Make clear which confounders were adjusted for and why they were included	✓	10, 11
		(*b*) Report category boundaries when continuous variables were categorized	✓	10, 11
		(*c*) If relevant, consider translating estimates of relative risk into absolute risk for a meaningful time period	n/a	n/a
Other analyses	17	Report other analyses done—eg analyses of subgroups and interactions, and sensitivity analyses	✓	10, 11
**Discussion**				
Key results	18	Summarise key results with reference to study objectives	✓	12
Limitations	19	Discuss limitations of the study, taking into account sources of potential bias or imprecision. Discuss both direction and magnitude of any potential bias	✓	12, 13
Interpretation	20	Give a cautious overall interpretation of results considering objectives, limitations, multiplicity of analyses, results from similar studies, and other relevant evidence	✓	12-14
Generalisability	21	Discuss the generalisability (external validity) of the study results	✓	13
**Other information**				
Funding	22	Give the source of funding and the role of the funders for the present study and, if applicable, for the original study on which the present article is based	✓	15

*Give information separately for exposed and unexposed groups.

Note: An Explanation and Elaboration article discusses each checklist item and gives methodological background and published examples of transparent reporting. The STROBE checklist is best used in conjunction with this article (freely available on the Web sites of PLoS Medicine at http://www.plosmedicine.org/, Annals of Internal Medicine at http://www.annals.org/, and Epidemiology at http://www.epidem.com/). Information on the STROBE Initiative is available at http://www.strobe-statement.org.

For peer review only

BMJ Open

The INTERGROWTH-21st Project International INTER-NDA standards for child development at two years of age: An International Prospective Population-based Study

Journal:	*BMJ Open*
Manuscript ID	bmjopen-2019-035258.R1
Article Type:	Original research
Date Submitted by the Author:	28-Feb-2020

Complete List of Authors:	Fernandes, Michelle; University of Southampton, Faculty of Medicine, Department of Paediatrics; University of Oxford, Nuffield Department of Women's & Reproductive Health<br>Villar, José; University of Oxford, Nuffield Department of Women's & Reproductive Health; University of Oxford, Perinatal Health Institute, Green Templeton College<br>Stein, Alan; University of Oxford, Department of Psychiatry<br>Staines Urias, Eleonora; University of Oxford, Nuffield Department of Women's & Reproductive Health<br>Garza, Cutberto; Johns Hopkins University, Johns Hopkins Bloomberg School of Public Health<br>Victora, Cesar; Federal University of Pelotas, Postgraduate Program in Epidemiology<br>Barros, Fernando; Catholic University of Pelotas, Post-Graduate Program on Health and Behavior; Universidade Catolica de Pelotas, Post-Graduate Program in Health and Behavior<br>Bertino, Enrico; Universita degli Studi di Torino<br>Purwar, Manorama; Ketkar Hospital, Nagpur INTERGROWTH-21st Research Centre<br>Carvalho, Maria; Aga Khan University - Kenya<br>Giuliani, Francesca ; Ospedale Infantile Regina Margherita, Ospedale Infantile Regina Margherita-Sant'Anna Citta della Salute e della Scienza di Torino<br>Wulff, Katharina; University of Oxford, Nuffield Department of Clinical Neuroscience<br>Abubakar, Amina A.; KEMRI-Wellcome Trust Research Programme, Neurosciences Unit<br>Kihara, Michael; USIU, Department of Psychology<br>Cheikh Ismail, Leila; University of Sharjah, College of Health Sciences<br>Aranzeta, Luis; Centro de Tecnologıa e Innovacion<br>Albernaz, Elaine; Universidade Federal de Pelotas, Faculty of Medicine<br>Kunnawar, Naina; Ketkar Hospital Nagpur, Nagpur INTERGROWTH-21st Research Centre<br>Di Nicola, Paola; Ospedale Infantile Regina Margherita, Ospedale Infantile Regina Margherita-Sant'Anna Citta della Salute e della Scienza di Torino<br>Ochieng, Roseline; Aga Khan University - Kenya, Faculty of Health Sciences<br>Sandells, Tamsin; University of Oxford, Nuffield Department of Women's

	& Reproductive Health<br>Savini, Sandy; University of Oxford, Nuffield Department of Women's & Reproductive Health<br>Temple, Sophie; University of Oxford, Nuffield Department of Women's & Reproductive Health<br>Murray, Elizabeth; University of Oxford, Department of Psychiatry, Warneford Hospital<br>Ohuma, Eric O; University of Oxford Medical Sciences Division<br>Gravett, MG; University of Washington, Departments of Obstetrics and Gynecology and of Global Health<br>Pang, Ruyan; Peking University, School of Public Health<br>Jaffer, Yasmine; Ministry of Health, Muscat, Department of Family & Community Health<br>Noble, J. Alison; University of Oxford, Department of Engineering Science<br>Winsey, Adele; University of Oxford, Nuffield Department of Women's & Reproductive Health<br>Lambert, Ann; University of Oxford, Nuffield Department of Women's & Reproductive Health<br>Papageorghiou, Aris; University of Oxford, Nuffield Department of Women's & Reproductive Health; University of Oxford, Oxford Maternal & Perinatal Health Institute, Green Templeton College,<br>Bhutta, Zulfiqar; The Hospital for Sick Children , Centre for Global Child Health<br>Kennedy, Stephen; University of Oxford, Nuffield Department of Women's & Reproductive Health; University of Oxford, Oxford Maternal & Perinatal Health Institute, Green Templeton College,
**Primary Subject Heading**:	Paediatrics
Secondary Subject Heading:	Epidemiology, Global health
Keywords:	Community child health < PAEDIATRICS, Developmental neurology & neurodisability < PAEDIATRICS, PAEDIATRICS, EPIDEMIOLOGY

SCHOLARONE™
Manuscripts

I, the Submitting Author has the right to grant and does grant on behalf of all authors of the Work (as defined in the below author licence), an exclusive licence and/or a non-exclusive licence for contributions from authors who are: i) UK Crown employees; ii) where BMJ has agreed a CC-BY licence shall apply, and/or iii) in accordance with the terms applicable for US Federal Government officers or employees acting as part of their official duties; on a worldwide, perpetual, irrevocable, royalty-free basis to BMJ Publishing Group Ltd ("BMJ") its licensees and where the relevant Journal is co-owned by BMJ to the co-owners of the Journal, to publish the Work in this journal and any other BMJ products and to exploit all rights, as set out in our licence.

The Submitting Author accepts and understands that any supply made under these terms is made by BMJ to the Submitting Author unless you are acting as an employee on behalf of your employer or a postgraduate student of an affiliated institution which is paying any applicable article publishing charge ("APC") for Open Access articles. Where the Submitting Author wishes to make the Work available on an Open Access basis (and intends to pay the relevant APC), the terms of reuse of such Open Access shall be governed by a Creative Commons licence – details of these licences and which Creative Commons licence will apply to this Work are set out in our licence referred to above.

Other than as permitted in any relevant BMJ Author's Self Archiving Policies, I confirm this Work has not been accepted for publication elsewhere, is not being considered for publication elsewhere and does not duplicate material already published. I confirm all authors consent to publication of this Work and authorise the granting of this licence.

For peer review only

The INTERGROWTH-21st Project International INTER-NDA standards for child development at two years of age: An International Prospective Population-based Study

Short title: INTERGROWTH-21st Project INTER-NDA standards

**Authors' names**	Michelle Fernandes (0000-0002-0051-3389), José Villar, Alan Stein, Eleonora Staines Urias, Cutberto Garza, Cesar G. Victora, Fernando C. Barros, Enrico Bertino, Manorama Purwar, Maria Carvalho, Francesca Giuliani, Katharina Wulff, Amina Abubakar, Michael Kihara, Leila Cheikh Ismail, Luis Aranzeta, Elaine Albernaz, Naina Kunnawar, Paola Di Nicola, Roseline Ochieng, Tamsin Sandells, Sandy Savini, Sophie Temple, Elizabeth Murray, Eric O. Ohuma, Michael G. Gravett, Ruyan Pang, Yasmine A. Jaffer, J. Alison Noble, Adele Winsey, Ann Lambert, Aris T. Papageorghiou, Zulfiqar A. Bhutta, and Stephen H. Kennedy.
**Authors' names, positions, affiliations and addresses**	Michelle Fernandes, Honorary Research Fellow and NIHR Academic Clinical Fellow in Paediatrics, Nuffield Department of Women's & Reproductive Health, Women's Centre, John Radcliffe Hospital, University of Oxford, Oxford OX3 9DU, UK and Faculty of Medicine, Department of Paediatrics, University Hospitals Southampton, University of Southampton, Southampton SO16 6YD, UK.<br><br>José Villar, Professor, Nuffield Department of Women's & Reproductive Health, University of Oxford, Oxford, OX3 9DU, UK and Oxford Maternal & Perinatal Health Institute, Green Templeton College, University of Oxford, Oxford, OX2 6HG, UK.<br><br>Alan Stein, Professor and Head of Section of Child and Adolescent Psychiatry, Department of Psychiatry, Warneford Hospital, University of Oxford, Oxford, OX3 7JX, UK.<br><br>Eleonora Staines Urias, Senior Statistician, Nuffield Department of Women's & Reproductive Health, University of Oxford, Oxford, OX3 9DU, UK.<br><br>Cutberto Garza, Professor, Johns Hopkins Bloomberg School of Public Health, Baltimore, MD, 21205, USA.<br><br>Cesar G. Victora, Professor, Programa de Pós-Graduação em Epidemiologia, Universidade Federal de Pelotas, Pelotas, 96010-610, Brazil.<br><br>Fernando C. Barros, Professor, Programa de Pós-Graduação em Saúde e Comportamento, Universidade Católica de Pelotas, Pelotas, 96015-560, Brazil.<br><br>Enrico Bertino, Professor, Dipartimento di Scienze Pediatriche e dell' Adolescenza, SCDU Neonatologia, Universita di Torino, Torino, 10126, Italy.

For peer review only

Manorama Purwar, doctor, Nagpur INTERGROWTH-21st Research Centre, Ketkar Hospital, Nagpur, 440012, Maharashtra, India.

Maria Carvalho, doctor, Faculty of Health Sciences, Aga Khan University, Nairobi, Kenya.

Francesca Giuliani, doctor, Ospedale Infantile Regina Margherita-Sant'Anna Citta della Salute e della Scienza di Torino, Torino, 10126, Italy.

Katharina Wulff, Senior Research Fellow, Nuffield Department of Clinical Neuroscience, University of Oxford, Oxford OX3 9DU.

Amina Abubakar, Neurosciences Unit, KEMRI/Wellcome Trust Research Programme, PO Box 230-80108 Kilifi, Kenya.

Michael Kihara, Professor, Department of Psychology, United States International University, Africa.

Leila Cheikh Ismail, College of Health Sciences, University of Sharjah, Sharjah, United Arab Emirates.

Luis Aranzeta, Software developer, Centro de Tecnologıa e Innovacion, Ciudad de Mexico 11529, Mexico.

Elaine Albernaz, doctor, Faculty of Medicine, Universidade Federal de Pelotas, Pelotas, 96015-560, Brazil.

Naina Kunnawar, doctor, Nagpur INTERGROWTH-21st Research Centre, Ketkar Hospital, Nagpur, 440012, Maharashtra, India.

Paola Di Nicola, doctor, Ospedale Infantile Regina Margherita-Sant'Anna Citta della Salute e della Scienza di Torino, Torino, 10126, Italy.

Roseline Ochieng, doctor, Faculty of Health Sciences, Aga Khan University, Nairobi, Kenya.

Tamsin Sandells, research assistant, Nuffield Department of Women's & Reproductive Health, University of Oxford, Oxford, OX3 9DU, UK.

Sophie Temple, research assistant, Nuffield Department of Women's & Reproductive Health, University of Oxford, Oxford, OX3 9DU, UK.

Sandy Savini, research assistant, Nuffield Department of Women's & Reproductive Health, University of Oxford, Oxford, OX3 9DU, UK.

Elizabeth Murray, PhD student, Department of Psychiatry, Warneford Hospital, University of Oxford, Oxford, OX3 7JX, UK.

For peer review only

	Eric O. Ohuma, Senior Statistician, Nuffield Department of Women's & Reproductive Health, University of Oxford, Oxford, OX3 9DU, UK and Centre for Statistics in Medicine, Nuffield Department of Orthopaedics, Rheumatology & Musculoskeletal Sciences, University of Oxford, Oxford, OX3 7LD, UK.<br><br>Michael G. Gravett, Adjunct Professor, Departments of Obstetrics and Gynecology and of Global Health, University of Washington, Seattle, WA 98195-6460, USA.<br><br>Ruyan Pang, Professor, School of Public Health, Peking University, Beijing, 100191, China.<br><br>Yasmine A. Jaffer, Department of Family & Community Health, Ministry of Health, Muscat, Sultanate of Oman.<br><br>J. Alison Noble, Professor, Department of Engineering Science, University of Oxford, Oxford, OX1 3PJ, UK.<br><br>Adele Winsey, Nuffield Department of Women's & Reproductive Health, University of Oxford, Oxford, OX3 9DU, UK.<br><br>Ann Lambert, Nuffield Department of Women's & Reproductive Health, University of Oxford, Oxford, OX3 9DU, UK.<br><br>Aris T. Papageorghiou, Professor, Nuffield Department of Women's & Reproductive Health, University of Oxford, Oxford, OX3 9DU, UK and Oxford Maternal & Perinatal Health Institute, Green Templeton College, University of Oxford, Oxford, OX2 6HG, UK.<br><br>Zulfiqar A. Bhutta, Professor, Center for Global Child Health, Hospital for Sick Children, Toronto, M5G 2L3, Canada.<br><br>Stephen H. Kennedy, Professor and Head of Department, Nuffield Department of Women's & Reproductive Health, University of Oxford, Oxford, OX3 9DU, UK and Oxford Maternal & Perinatal Health Institute, Green Templeton College, University of Oxford, Oxford, OX2 6HG, UK.
**Corresponding author**	Correspondence to Michelle Fernandes, Nuffield Department of Women's & Reproductive Health, Women's Centre, John Radcliffe Hospital, University of Oxford, Oxford OX3 9DU, UK.<br><br>E: michelle.fernandes@wrh.ox.ac.uk; T:+44(0)1865222122
**Keywords**	Community child health, child development, paediatrics, epidemiology, neurodevelopment.

For peer review only

WORD COUNTS

Abstract: 299

Article Summary: 173

Main manuscript: 5016

For peer review only

For peer review only - http://bmjopen.bmj.com/site/about/guidelines.xhtml

ABSTRACT

Objectives

To describe the construction of the international INTER-NDA standards for child development at two years by reporting the cognitive, language, motor and behaviour outcomes in optimally healthy and nourished children in the INTERGROWTH-21st Project.

Design

Population-based cohort study, the INTERGROWTH-21st Project.

Setting

Brazil, India, Italy, Kenya and the UK.

Participants

1181 children prospectively recruited from early fetal life according to the prescriptive WHO approach, and confirmed to be at low risk of adverse perinatal and postnatal outcomes.

Primary Measures

Scaled INTER-NDA domain scores for cognition, language, fine and gross motor skills and behaviour; vision outcomes measured on the Cardiff tests; attentional problems and emotional reactivity measured on the respective subscales of the preschool Child Behaviour Checklist (CBCL); and the age of acquisition of the WHO gross motor milestones.

Results

Scaled INTER-NDA domain scores are presented as centiles, which were constructed according to the prescriptive WHO approach and excluded children born preterm and those with significant postnatal/neurological morbidity. For all domains, except negative behavior, higher scores reflect better outcomes and the threshold for normality was defined as ≥ 10th centile. For the INTER-NDA's cognitive, fine motor, gross motor, language and positive behaviour domains these are ≥38.5, ≥25.7, ≥51.7, ≥17.8, and ≥51.4, respectively. The threshold for normality for the INTER-NDA's negative behaviour domain is ≤50.0, i.e. ≤90th centile. At 22 to 30 months of age, the cohort overlapped with the WHO motor milestone centiles, showed low postnatal morbidity (<10%), and vision outcomes, attentional problems and emotional reactivity scores within the respective normative ranges.

Conclusions

From this large, healthy and well-nourished, international cohort, we have constructed, using the WHO prescriptive methodology, international INTER-NDA standards for child development at 2 years of age. Standards, rather than references, are recommended for population-level screening and the identification of children at risk of adverse outcomes.

For peer review only - http://bmjopen.bmj.com/site/about/guidelines.xhtml

ARTICLE SUMMARY

- The prescriptive WHO approach for developing biological standards was applied to a population-based sample of healthy and well-nourished children from Brazil, India, Italy, Kenya and the UK to construct the INTERGROWTH-21st Project neurodevelopmental assessment (INTER-NDA) standards for child development.
- Comprehensive health, growth and neurodevelopmental data were prospectively collected, from early pregnancy to 2 years post-birth, providing a unique opportunity to confirm the cohort's health and nutritional status and to control for multiple risk factors associated with sub-optimal child development.
- The INTER-NDA is a mixed-methodology, multi-dimensional, standardised measure of early child development, that can be administered rapidly, by non-specialists in high-, middle- and low-income settings.
- The INTER-NDA is a standardized screening assessment and does not provide a clinical diagnosis.
- The age range of the INTER-NDA is 22 to 30 months and limits its generalizability to other age groups.

For peer review only

INTRODUCTION

Approximately 250 million children under the age of five worldwide are at risk of not achieving their developmental potential.[1] Effective interventions are available but maximising their benefit at scale depends upon identifying those children at greatest need, preferably using standardised methodology.[2]

At present, a multiplicity of methods are used to measure neurodevelopment during early childhood (Supporting Information S1).[3 4] Many of these are administered by specialist staff and were developed using children from either high-income (HICs) or specific low- and middle-income countries (LMICs), each drawing their normative sample (often country- or region-specific) from the respective settings (Supporting information S1 and S2).[3 4] To our knowledge, none of these tools commonly used to measure neurodevelopment in early childhood, were based on children monitored from fetal life, and have adopted the prescriptive approach recommended by the World Health Organization (WHO) for the development of international biological standards during the construction of their norms.[5] Instead, references have been commonly used to assess the overall achievement of developmental skills and track progress over time in both, groups of children, and individuals. However, while references describe *how* children, in a specific setting and time, have attained certain milestones of interest, they do not describe how children, in all settings, *should* develop. The importance of this fundamental difference between references and standards was elegantly highlighted, in the context of skeletal growth in young children, by the WHO Multicentre Growth Reference Study (MGRS), which resulted in the construction of prescriptive international standards for monitoring child growth.[6] These WHO standards, describing optimal growth from early pregnancy to five years of age[7], are now widely employed in clinical practice and used to make comparisons across disparate populations.

The construction of international, prescriptive standards describing optimal neurodevelopment during early childhood is challenging not least because of the technical and logistical difficulties of implementing comprehensive early child developmental assessments across large international populations. To construct international *standards* of child development, in accordance with the WHO's prescriptive methodology,[5] four fundamental methodological principles must be fulfilled: (1) The normative sample should be selected using a "prescriptive" approach, which includes consideration of key factors known to be associated with poor developmental outcomes during early childhood (Supporting Information S2); (2) the conceptual framework *must* be population-based and international; (3) rigorous data management, standardisation and quality control procedures must be included, and (4) measurements must be complemented by independent assessments of specific functional and developmental domains (e.g. tests of vision) to confirm the prescriptive characteristics of the sample. This rigorous approach is important because the inclusion of inadequately nourished children, or those with mild neurodevelopmental disturbances (NDDs), in normative samples, can affect resultant thresholds. Moreover, the identification of children at risk of (even mild) NDDs is essential because there is evidence to show that very small developmental differences between individuals during early childhood can result in marked discrepancies in mental and physical health, educational attainment, and social and economic outcomes during later life.[8 9]

The INTERGROWTH-21st Project aimed to adopt this WHO prescriptive approach in constructing international standards for child development measured on a standardized, comprehensive assessment tool – the INTERGROWTH-21st Neurodevelopment Assessment (INTER-NDA) - at 2 years of age. Despite this circumscribed age range, by leveraging on the INTERGROWTH-21st Project's international cohort of mothers and children, recruited specifically to be optimally healthy and well-nourished throughout the duration of pregnancy and confirmed, during the infant follow-up component of the project, to be at low risk of adverse birth, health and growth outcomes at birth, 1 and 2 years of age, we were able to adopt the prescriptive approach and methods

For peer review only - http://bmjopen.bmj.com/site/about/guidelines.xhtml

recommended by the WHO MGRS in the construction of the INTER-NDA *standards* of child development at 2 years of age. In the present study, we analyzed cognitive, language, motor and behaviour outcomes at 2 years of age, measured on the INTER-NDA, for healthy and well-nourished children from the INTERGROWTH-21st Project study sites in Brazil, India, Italy, Kenya and the UK. We compared the vision, gross motor, attentional problems and emotional reactivity profiles, as well as growth and health outcomes, in these children to the corresponding norms for these independent measures.

For peer review only

METHODS

Study design and population

The INTERGROWTH-21st Project was a multi-centre, population-based study conducted between 2009 and 2016, in eight delimited geographical areas worldwide: the cities of Pelotas, Brazil; Turin, Italy; Muscat, Oman; Oxford, UK; Seattle, USA; Shunyi County, a suburban district of the Beijing municipality, China; the central area of the city of Nagpur, Maharashtra, India; and the Parklands suburb of Nairobi, Kenya. A geographical area was a complete city, or county, or part of a city with clear political or geographical limits, located at an altitude <1600m, with low-risk health indicators for perinatal morbidity and mortality, in which women receiving antenatal care had plans to give birth within the area, that had to be free or have low levels of major, known, non-microbiological contamination.[10] The primary aim of the INTERGROWTH-21st Project was to study growth, health, and development from early fetal life to two years of age in low-risk populations of mothers and children with optimal health and nutrition so as to produce prescriptive standards of fetal growth, newborn size and early child neurodevelopment to complement the existing WHO Child Growth Standards.

The INTERGROWTH-21st Project recruited pregnant women from the aforementioned populations, who met the individual entry criteria of health, nutrition, education, and socio-economic position (Supporting Information S3).[10] Standardised clinical care and neonatal feeding practices were implemented based on project protocols. The newborn cohort was followed up at birth, one and two years of age and evaluated for growth, nutrition, health, and the WHO gross motor milestones, using standardised methodology and rigorous quality control processes.[11] They constitute the Infant Follow-up Study (IFS) of the INTERGROWTH-21st Project. The baseline characteristics of the full cohort and follow-up methodology have been published elsewhere.[11] The project protocols are available at www.intergrowth21.org.uk.

Data collection and evaluation methods

All eligible children in five of the eight INTERGROWTH-21st Project study sites (the cities of Pelotas (Brazil); Turin (Italy); Oxford (UK); Nagpur (India) and the Parklands suburb of Nairobi (Kenya)), who had contributed data towards the construction of the international Fetal Growth and Newborn Growth Standards,[12 13] were invited to attend a comprehensive neurodevelopmental evaluation at the time of their second birthday. This age was selected as it was found to be the earliest at which: (i) neurodevelopment is not confounded by transient neurological syndromes of prematurity and (ii) conventionally used developmental instruments, such as the Bayley Scales of Infant Development (BSID), have been found to possess an acceptable level of medium and long term predictive validity.[14] The sites in China, Oman, and the USA did not participate because of logistical and administrative reasons, delays in the start of the study and/or staff availability, all unrelated to the IFS' main hypotheses (a comparison in the demographics, and health and growth outcomes between these sites has already been published).[11]

The evaluation consisted of (in order of administration): an assessment of vision (the Cardiff tests) an assessment of cognition, motor skills, language skills and behaviour (the INTER-NDA); caregiver reports of attentional problems and emotional reactivity (the corresponding subscales of the preschool Child Behaviour Checklist; CBCL); measurement of cortical auditory processing (to a novelty odd-ball paradigm on a wireless, gel-free electroencephalography system); measurement of infant sleep (using actigraphy) and an assessment of gross motor milestones (based on the WHO's checklist). Despite measuring cortical auditory processing and sleep in our cohort, a description of the methods and results relating to these technically complex outcomes are

beyond the scope of this paper. Moreover, as normative values for cortical auditory evoked response potentials and actigraphy data do not exist for children aged 2 years, the added value of these measures in confirming the healthy and well-nourished status of the cohort is uncertain. Information on the child's health and nutritional status, and anthropometric measurements (weight, length and head circumference), were also collected, at the 2-year visit, according to the INTERGROWTH-21st Project protocols.

A specially designed training program for the neurodevelopmental evaluation was implemented at all sites between 2012 and 2013.[15] Staff administering the assessments were aware of the project's general principles but not the specific hypotheses being tested. They were also unaware of individual children's scores from their own and other study sites.

Primary outcome measure: The INTERGROWTH-21st Neurodevelopment Assessment (INTER-NDA)

The INTER-NDA is a comprehensive, rapid assessment of cognition, (fine and gross) motor skills, language, and (positive and negative) behaviour for children aged 22 to 30 months (Supporting Information S4).[15] Its 37 items are administered in approximately 15 minutes using a combination of psychometric techniques (direct administration, concurrent observation and caregiver reports) to minimise risks of reporter and recall bias commonly encountered in caregiver interviews[3] while acknowledging that children might perform differently in artificial testing environments than in familiar settings. Children's performance on the INTER-NDA is scored across a spectrum of abilities, rather than on a pre-defined checklist and, therefore, affords a wider description of a child's faculties.[15] It has demonstrated strong agreement with the BSID, 3rd edition (BSID-III) (interclass correlation coefficients 0·75 to 0·88, p<0·001 for all domains with little to no bias on Bland Altman analysis); satisfactory internal consistency (Cronbach's alpha 0.56 to 0.81) and good unidimensionality across subscales (Comparative Fit Index = 0.90; Tuckler Lewis Index = 0 .94)[16]; and good levels of inter-rater (k=0·70; 95% CI: 0·47–0·88) and test re-test reliability (k=0·79; 95%CI: 0·48–0·96).[15]

The INTER-NDA is designed for use across socio-economic groups and populations. Its operation manual, standardisation protocol and forms are freely available at www.intergrowth21.org.uk. The kit consists of common household items encountered across the world. In all study sites, the INTER-NDA was translated into the local languages of the sites (Brazil: Brazilian Portuguese, India: Marathi; Italy: Italian; Kenya: Kiswahili), using the WHO Mental Health Initiative translation guidelines[17], which included processes of cultural customization, translation and back translation.

Other Outcome Measures of Neurodevelopment

To confirm the developmental normality of our cohort, we assessed specific functional and developmental outcomes of relevance by including three measurements independent of the INTER-NDA: (i) visual acuity and contrast sensitivity, measured on the Cardiff Tests;[18] (ii) attention problems and emotional reactivity measured on the respective subscales of the preschool CBCL,[19] and (iii) the age of achievement of six gross motor milestones measured on the WHO's checklist.[20]

The Cardiff Tests are validated and reliable measures of binocular vision in children that are not influenced by co-existing disturbances in language or cognition, and are independent of cultural biases. Their norms have been applied for clinical purposes.[18] The operational manual for their use in the INTERGROWTH-21st Project is available at https://www.intergrowth21.org.uk. Their administration takes 5 minutes. Visual acuity and contrast

For peer review only - http://bmjopen.bmj.com/site/about/guidelines.xhtml

sensitivity are measured in quick succession and taken together are a more robust measure of the integrity and functioning of the entire visual pathway than either test alone.[21]

The preschool version of the CBCL is a parent-rated questionnaire used worldwide as a diagnostic screen for behavioural and emotional problems in young children (https://aseba.org/translations/).[19] In the IFS of the INTERGROWTH-21st Project, mothers completed questions relating to the attentional problems and emotional reactivity CBCL scales.

The WHO Gross Motor milestones checklist consists of the normative windows of achievement for six gross motor milestones, developed from the WHO MGRS cohort between 4 and 24 months of age.[20] In the INTERGROWTH-21st Project, parents were asked to report the age when they first observed or "never observed" the milestones. The same information was collected from parents at the 1 and 2 year follow-up visits to evaluate the consistency of the reported dates.[11]

For peer review only

Data Management and Statistics

The INTERGROWTH-21st Project neurodevelopmental evaluation was supported by an electronic, tablet-based data collection and management system (the NeuroApp).[15] This contained the INTER-NDA and vision scoring forms, operation manuals, visual cues and integrated data quality checks to facilitate rapid collection of high-quality data and to ensure their secure upload to the project's centralised and site-based data-servers on which rigorous monthly checks were performed.[22]

For the INTER-NDA, two standardisation evaluations were carried out, in accordance with guidelines published in the World Bank's Toolkit for Examining Early Child Development,[3] to assess the ability of assessors to score and administer the INTER-NDA. During the first evaluation, assessors scored children's skills on the INTER-NDA from video recordings of four assessments performed by an expert assessor. Inter-rater and test-re test reliability were compared between assessors. At the second evaluation, an expert observed assessors performing three assessments each, and rated each assessor for their ability to administer the INTER-NDA correctly on a standardised protocol adherence checklist (Supporting Information S5). Protocol adherence scores were compared between assessors. The results of these evaluations are presented in Supporting Information S6.

The sample size considerations for this report have been previously published and depended on pragmatic considerations.[23] In summary, as the present report is the 2-year follow-up of the initial FGLS cohort of pregnant women, the total number of eligible children assessed at 2 years of age was therefore fixed. The initial sample size estimations (approximately 500 fetuses per site) focused on the precision and accuracy of the extreme centiles of the complete population, i.e. the 3rd or 97th centile because they correspond closely to ±2SD, and they are the recommended cut-offs of the WHO Child Growth Standards, which are used internationally to evaluate children of this age; however, in the present study, such estimations do not apply because of the different nature of the hypothesis.[23] In this component of the study, neurodevelopment was evaluated in an average of 261 children per site (1307 children total) at 2 years of age. This sample size was considered adequate to explore the predicted small site-specific differences. Post-hoc power calculations showed that the study was sufficiently powered to observe small differences among study sites (calculations for INTER-NDA domains with power >0.99) and small effect sizes for the between-group variances.[23] For example, for a between-group variance of 10% of the total variance and a two-tail alpha of 0.05, the power is 0.84.

Summary statistics were calculated for birth, neonatal and postnatal characteristics of children completing the neurodevelopmental evaluation and compared to those lost to follow-up. These characteristics include most

For peer review only - http://bmjopen.bmj.com/site/about/guidelines.xhtml

factors associated with poor child neurodevelopmental outcomes during the first two years of life (Supporting Information S2). The analytical and statistical strategy for the construction of the INTER-NDA centiles is presented in Figure 1. For all analyses, Stata 15 software was used (StataCorp. 2017, StataCorp LLC, College Station, TX).

Data from the participating sites were pooled, following the strategy recommended by WHO.[7] We have previously reported striking similarities in the distribution of the INTER-NDA domains among children from the five sites.[23] In summary, similar to the patterns observed in linear growth from fetal life to childhood, the variability in INTER-NDA scores between sites is far less (for most domains <10%) than the total variability between individuals within a study site, justifying pooling the data to construct international standards.[23 24]

Raw mean INTER-NDA domain scores (Supporting Information S7[16]) were calculated and their distributions explored. These showed important skewness and (particularly) kurtosis. As 30 INTER-NDA items were scored on a five-point scale, and six items were scored on a three-point scale, raw domain scores were converted to standardised scaled scores (Supporting Information S8).

To explore the low-risk profile of the cohort, centiles for visual acuity (measured in logMar) and contrast sensitivity (measured in contrast %) were determined and compared to the Cardiff Tests' established norms.[18] Attention problem and emotional reactivity subscale scores were calculated using ASEBA-web software, and compared to the CBCL's norms for Group 1 societies.[19] The proportion of children within the WHO motor development windows of achievement was estimated as previously described.[11]

In addition, after other exclusions (Figure 2), 28 of 1209 eligible children scored above the CBCL's 97th percentile threshold for clinical problems on the attentional problems and emotional reactivity CBCL subscales. We compared INTER-NDA centiles including and excluding this group (Supporting Information S9). As the INTER-NDA centiles were marginally lower on some domains when this group was included, we decided to exclude these children from the normative sample INTER-NDA sample in the construction of the INTER-NDA domain standards.

As no transformation was identified that suited all INTER-NDA domains, the Harrell-Davis distribution-free estimator was used to estimate pooled centiles from the standardised scaled scores.[25] This estimator weights the order statistics by the difference between two incomplete beta functions. INTER-NDA scaled domain scores were compared between boys and girls using the Wilcoxon rank-sum test.

Patient and public involvement

Patients were not involved in developing plans for the design of the study. Parents showed support for the study through high and sustained follow-up rates in all study sites. The INTERGROWTH-21st Project maintains contact with parents in the cohort through newsletters, webinars and blogs on its website, https://intergrowth21.tghn.org/ and through Twitter (@intergrowth21st).

For peer review only - http://bmjopen.bmj.com/site/about/guidelines.xhtml

RESULTS

The INTERGROWTH-21st Project Infant Follow-up Study: INTER-NDA normative cohort characteristics and overall health and nutrition at 2 years of age

Population

Of the 1758 eligible children enrolled in the five participating sites, 1339 (76%) were assessed at two years of age (Figure 2). After exclusions (including 54 children (3.1%) who were born at <37^{+0} weeks' gestation and 28 children who scored at the threshold for clinical problems on the attentional problems and/or emotional reactivity subscales of the CBCL), data from 1181 healthy children (67% of those eligible) were pooled to construct the international INTERGROWTH-21st Neurodevelopmental Standards. The study sites in Brazil, India, Italy, Kenya and the UK respectively contributed 147 (12.2%), 305 (25.2%), 296 (24.5%), 301 (24.9%) and 160 (13.2%) children to the normative INTER-NDA cohort. A detailed description of the prenatal, birth, postnatal morbidity, growth and nutritional characteristics of the cohort, during the first two years of life, has previously been published and is presented in Tables 1 and 2.[11] The comparison in socio-demographic, birth, health and growth characteristics between the five sites that contributed to the normative INTER-NDA cohort, and the three sites that did not, has also been previously published - no significant differences were observed between the two groups.[11]

The mean (± SD) age of both girls and boys at assessment was 24.8 (± 1.6) months. Eighty-nine percent of the neurodevelopmental measures were obtained between 22 and 24 months of age, and 99.9% between 22 and 30 months. The baseline prenatal, perinatal and neonatal characteristics were very similar across the five sites,[23] and with those children lost to follow-up (Table 1).

Health, growth and nutritional outcomes from birth to two years

The cohort's mean gestational age and weight at birth were 39.6 (± 1.2) weeks and 3.2 (± 0.4) kg, respectively.[11] The mean birth length and head circumference were 49.2 (±1.8) cms and 34.0(+1.3) cms respectively. Mean age at discharge from hospital, post-birth, was 3 (2-4) days. At hospital discharge, 89% of the cohort was exclusively breast-milk fed.[11] Exclusive breastfeeding was stopped at a median of 5 months (interquartile range, 3-6 months) and (any) breastfeeding stopped entirely at a median of 12 months (interquartile range, 6-18 months). Detailed information on the nutritional status of the cohort has been previously published.[11]

The overall postnatal morbidity of the cohort was low (Table 2): 9.4% of infants were hospitalised during the second year of life with a median hospital stay of 2 days (IQR 1, 3 days). The most frequently morbidities reported in outpatient clinics were exanthema/skin diseases, ≥3 episodes of fever lasting ≥3 days, and otitis media/lower-tract respiratory infections.[11 23] At two years of age, 92%, 90% and 91% of the cohort's length, weight and head circumference measures respectively were within the 3rd and 97th centiles of the WHO Child Growth Standards.[11]

Developmental profile of the cohort on independent measures of vision and development at 2 years

The visual acuity and contrast sensitivity centiles for our cohort are presented in Table 3. The cohort's 50th centile values for visual acuity and contrast sensitivity were 0.20 logMar and 1.5%, respectively. Both are within the Cardiff Tests' normative values for binocular visual acuity in children aged 24 to 30 months.[18] The visual acuity and contrast sensitivity values were identical for boys and girls across all centiles (Table 3) suggesting no biological variability in these outcomes between sexes.

For peer review only - http://bmjopen.bmj.com/site/about/guidelines.xhtml

The cohort's attentional problems and emotional reactivity scores at the 50th centile corresponded to CBCL T-scores of 53 and 50 respectively, i.e. the 62nd and <50th CBCL centiles. These values are below the CBCL's 93rd centile threshold for "borderline clinical problems".[19] For these CBCL subscales, 28 (2.1%) FGLS children scored above the CBCL's cut-off for clinical problems (>97th centile). These children were excluded from the INTER-NDA normative sample.

At two years of age, the cohort overlapped almost perfectly with the WHO motor milestones at the 50th, 3rd and 97th centiles of the range for healthy term infants.[11] For length and head circumference, the mean $\pm$ SD z-score was 0.0 ± 1.1 for both measures, and the respective medians were at the 49th and 50th percentiles of the WHO Child Growth Standards.[11] For weight, the mean $\pm$ SD z-score was 0.2 ± 1.1, and median was at the 58th percentile. A detailed description of these characteristics are presented elsewhere.[11]

International standards for the cognitive, motor, language and behavior domains of the INTER-NDA

The 3rd, 5th, 10th, 50th, 90th, 95th and 97th centiles for the INTER-NDA standardised (scaled) scores for cognition, language, motor, and behaviour domains for healthy, well-nourished two year-old children are presented, in Table 4, for the pooled cohort. For all INTER-NDA domains, except negative behavior, higher scores reflect better outcomes and the threshold for normality was defined as ≥10th centile. For negative behavior, where lower scores reflect better outcomes, the threshold for normality was defined as ≤90th centile. The thresholds of normality for the INTER-NDA's cognitive, fine motor, gross motor, language and positive behaviour domains are ≥38.5, ≥25.7, ≥51.7, ≥17.8, and ≥51.4 respectively. The threshold for normality for the INTER-NDA's negative behaviour domain is ≤50.0. To facilitate the easy and rapid implementation of these standards, in clinical, community and research settings, for the identification of children scoring ≤10th and ≤3rd centile on the INTER-NDA (≥90th and ≥97th centiles for negative behaviour) who would benefit from urgent and routine further assessment and/or specialist referral respectively, we have developed a neurodevelopmental chart that can be printed or downloaded (Figure 3).

INTER-NDA domain scores were similar between the cohort's male and female children (Supporting Information S10). There was a trend towards higher cognitive and language scores among girls, and higher negative behaviour scores among boys (Supporting Information S10); however, the clinical and developmental implications of these differences are unclear.

For peer review only

DISCUSSION

From this international, population-based cohort of optimally healthy and nourished children from Brazil, India, Italy, Kenya and the UK (Supporting Information S11 and S12); monitored from early pregnancy to two years of age, we have constructed international prescriptive standards for cognitive, language, motor and behavioural outcomes in two year-old children measured on a rapid, comprehensive assessment - the INTER-NDA. These centiles were constructed after excluding children born at <37 weeks gestation; those with significant/neurological morbidity, those whose mothers were known to have a mental health diagnoses during pregnancy and those who scored above the threshold for clinical attentional and emotional reactivity problems on the CBCL. We have confirmed the prenatal, perinatal, neonatal and postnatal healthy and well-nourished status of the normative INTER-NDA cohort using multiple measures during pregnancy, birth, 1 and 2 years of age; and have confirmed, at 2 years of age, its low-risk profile or adverse neurodevelopmental outcomes.[11] The threshold of normality for the INTER-NDA's cognitive, fine motor, gross motor, language and positive behaviour domains is ≥38.5, ≥25.7, ≥51.7, ≥17.8, and ≥51.4 respectively. The threshold for normality for the INTER-NDA's negative behaviour domain is ≤50.0. These centiles represent, to our knowledge, the first endeavour to construct standards for child developmental outcomes in this age group using the WHO prescriptive methodology *and* an international sampling frame. To facilitate the easy and rapid implementation of these standards in clinical, community and research settings for the identification of children at risk who would benefit from routine and urgent further assessment and specialist referral, respectively, we have developed a neurodevelopmental chart that can be printed or downloaded (Figure 3).

Strengths and limitations of this study

The strengths of the Infant Follow-up Study of the INTERGROWTH-21st Project include the population based cohort design; the use of the WHO recommended "prescriptive" approach; the international sampling frame; the inclusion of rigorous data management, standardization and quality control procedures and the incorporation of independent measurements of specific functional and developmental domains (vision, attentional problems, emotional reactivity and age of acquisition of key gross motor milestones) to confirm the satisfactory growth, health and development of our cohort was confirmed prior to the construction of these standards.[11] In addition, we used the INTER-NDA as the developmental measure of choice to construct these standards (Table 5). In Supporting Information S1 and S2, we present an overview of the normative samples and thresholds for NDDs of ten instruments commonly used to measure neurodevelopmental outcomes in two year-old children. Of these, two tools (the Guide for Monitoring Child Development, GMCD[26], [27] and the Caregiver-reported Early Developmental Instruments, CREDI[28]) fulfill some of the WHO-based methodological criteria for the construction of child developmental standards (GMCD: criteria 1, 3 and 4; and CREDI: criteria 2 and 4). The INTER-NDA fulfills 24 of the 26 criteria. Although a multi-dimensional assessment, is easy to implement and was designed for use across population groups in high-, middle- and low-income settings.[15] Despite an administration time of 15 minutes, it has demonstrated good to acceptable agreement with the BSID-III[16], and can be administered reliably, in the field, by trained non-specialists (Supporting Information S6).

The main limitation of our study was that the INTER-NDA is restricted to the 2-year age group. We selected 22 to 30 months as the time-point for the key developmental assessment of the entire study because developmental markers at this age have been found to be predictive of intelligence, school performance, adult nutrition and human capital in high-, middle- and low-income settings;[29-31] this age also corresponds to the end of Piaget's sensorimotor stage.[32] We acknowledge that, while some authors prefer a wider age range for population-

For peer review only - http://bmjopen.bmj.com/site/about/guidelines.xhtml

based child developmental surveillance,[3] the second birthday remains the earliest time point at which a holistic snapshot of a child's developmental repertoire can be captured reliably and parsimoniously at scale,[15] while still *within* 'the golden window of opportunity for neurodevelopment rescue' - the first 3 years of life - when interventions are evidenced to yield considerable benefits.[33] Conversely, some may argue that the 22 to 30 month age range is too broad in the context of the rapidly developing nervous system. By evaluating the performance of the INTER-NDA against the BSID-III in children aged 22 to 30 months[16], we have provided evidence that the INTER-NDA is a valid and reliable measure of child development in this age group. Nevertheless, the INTER-NDA is a standardized screening assessment and does not provide a clinical diagnosis. Therefore the possibility for misclassification must be considered when interpreting the findings. A further limitation is that three of the INTERGROWTH-21st Project study sites (China, Oman and the USA) did not participate in the neurodevelopmental evaluation. While the inclusion of these sites might have increased our overall sample size; as evidenced by the WHO MGRS study, the representation of every country is not necessary for the construction of biological standards because of the inherent prescriptive nature of the cohort.[34 35] Our findings, published in 2014 and earlier this year, confirmed that the growth and development of children across different ancestries, geographies and cultures are very similar from early pregnancy to 2 years of age, when environmental constraints on their health and nutrition are minimal, and justified the pooling of data across the five populations for the construction of international standards.[23 24] An additional limitation of our study is the exclusion of the detailed auditory and actigraphy data from the analyses.

To address the question as to what limits should be applied to determine thresholds of normal and non-normal development[12], we were guided by other neurodevelopmental tools using centile ranks to stratify NDD risk (Supporting Information S1). While many of these define sub-optimal development as below the 25th centile, we have presented evidence that most children in our cohort were developmentally normal for age. Therefore, we selected a lower threshold (≥10th centile) to define neurodevelopmental normality. Nevertheless, we acknowledge that, in clinical practice, risk-threshold determination may often take into consideration other factors such as parental concerns and resource allocation.[12]

Context of the study

Measuring neurodevelopmental milestones during early childhood at scale and comparing outcomes across populations are essential prerequisites for achieving the United Nations Sustainable Development Goal (UN SDG) 4.2 ("ensure that all girls and boys have access to quality early child development, care and pre-primary education so that they are ready for primary education"). The international INTER-NDA standards presented here contribute an important component to the care of young children: a unique clinical tool for use across all health-care systems (Table 5) to measure neurodevelopmental milestones and associated behaviours in two year-olds uniformly and at scale, and to identify children at risk of NDDs who would benefit from specialist referral and further investigation (Figure 3). It is hoped that these INTER-NDA standards, complementing our published standards for fetal growth and newborn size, and the WHO Child Growth Standards, will (i) contribute to the attainment of the early child development components of the UN SDGs and the WHO survive, thrive, and transform goals of the Global Strategy on Women's, Children's and Adolescents' Health; and (ii) provide a methodological template for the extension of the construction of child developmental standards to younger and older age groups.

For peer review only - http://bmjopen.bmj.com/site/about/guidelines.xhtml

Conclusion

From this international, population-based cohort of healthy and well-nourished children, confirmed to be at low-risk of adverse health, growth and developmental outcomes during the first two years of life, we have constructed the first international standards for cognition, language, motor skills and behavior at two years of age measured on the INTER-NDA. The use of standards to measure early child development is superior to references because of their prescriptive nature and universal applicability, in a manner similar to growth standards.

For peer review only

Acknowledgements

We gratefully acknowledge the Health Authorities in Pelotas, Brazil; Nagpur, India; Turin, Italy; Nairobi, Kenya, and Oxford, UK who facilitated this Project by permitting the participation of the respective study sites as collaborating centres. We are grateful to MedSciNet, UK, and Centro de Tecnología e Innovación, Mexico, for their support in the development, customisation and maintenance of the Project database and the NeuroApp, respectively. We are extremely grateful to the participating mothers and children in all the study sites, and to all those who contributed to the development of the INTERGROWTH-21st Project protocol.

Contributors

JV and SHK conceptualised and designed the INTERGROWTH-21st Project. MF, JV, SK, AS, LA, AA, JAN, MK, FG and KW designed and developed the FGLS neurodevelopmental follow-up and the INTERGROWTH-21st Neurodevelopment Assessment (INTER-NDA). JV, SK, CV, FB, ZB, CG, EB, AP, MG, PR, YJ, LCI and EB were responsible for the implementation all aspects of the INTERGROWTH-21st Project. MF, JC, SHK and LCI coordinated the implementation of the neurodevelopmental follow-up. MF, LCI, FB, EA, PdN, EB, FG, MP, NS, RO, MC, TS, ST, EM, AL, NK, SS and AW were responsible for site-based training and data collection. JV, SK, ESU, EO and MF were responsible for data management. MF, JV, ESU, EO and SHK had access to the Project's data; JV, ESU, MF, SHK and EO were responsible for the statistical analysis. MF and JV wrote the report with input from all the other authors. All authors reviewed and approved the final manuscript.

SHK and JV are responsible for the overall content as guarantors. The guarantors accept full responsibility for the work and/or the conduct of the study, had access to the data, and controlled the decision to publish. The corresponding author attests that all listed authors meet authorship criteria and that no others meeting the criteria have been omitted.

Funding

This work was supported by a grant from the Bill & Melinda Gates Foundation to the University of Oxford, Oxford, UK (Grant ID# 49038). MF is supported by an Academic Clinical Fellowship in Paediatrics from the National Institute for Health Research, UK to the University of Southampton. AP is supported by the National Institute for Health Research Oxford Biomedical Research Centre. The funders had no role in the study design, data collection and analysis, decision to publish or preparation of the manuscript.

Competing interests

None declared.

Ethics Approval

The INTERGROWTH-21st Project was approved by the research ethics committees of the Universidade Federal de Pelotas, Faculdade de Medicina comitê de ética em pesquisa (Ref: OF.051/09), the Indian Ministry of Health and Family Welfare and the Institutional Ethics Committee, Ketkar Hospital, Nagpur (Ref: 5/7/314/2008-RHN); Servizio Sanitario Nazionale – Regione Piemonte, Aziende Ospedaliere OIRM/S.Anna, Oridine Mauriziano di Torino, Comitato Etico Interaziendale (Ref: G9947/CEI/ C.27.2); the Aga Khan University Health Research Ethics Committee, Aga Khan University, Nairobi, Kenya (Ref: AKU- 09-106), and the Oxfordshire Research Ethics Committee 'C', United Kingdom (Ref: 08/H0606/139). Parents provided written, informed consent for their children.

For peer review only

Data Sharing Statement

Extra data is available by emailing *intergrowth21st@tghn.org*.

Open access

This is an Open Access article distributed in accordance with the Creative Commons Attribution Non Commercial (CC BY-NC 4.0) license, which permits others to distribute, remix, adapt, build upon this work non- commercially, and license their derivative works on different terms, provided the original work is properly cited and the use is non-commercial. See: http:// creativecommons.org/licenses/by-nc/4.0/

For peer review only

REFERENCES

1. Richter LM, Daelmans B, Lombardi J, et al. Investing in the foundation of sustainable development: pathways to scale up for early childhood development. The Lancet 2017;389(10064):103-18
2. Ertem IO, World Health Organization. Developmental difficulties in early childhood: prevention, early identification, assessment and intervention in low- and middle-income countries: a review. Geneva, Switzerland: World Health Organization, 2012:112
3. Fernald LCH, Kariger P, Engle P, et al. Examining Early Child Development in Low-Income Countries: A Toolkit for the Assessment of Children in the First Five Years of Life. Washington DC: The World Bank, 2009.
4. Fischer VJ, Morris J, Martines J. Developmental screening tools: feasibility of use at primary healthcare level in low-and middle-income settings. Journal of health, population, and nutrition 2014;32(2):314
5. Garza C, de Onis M. Rationale for developing a new international growth reference. Food & Nutrition Bulletin 2004;25(1):5S-14S
6. de Onis M, Garza C, Victora CG, et al. The WHO Multicentre Growth Reference Study: planning, study design, and methodology. Food Nutr Bull 2004;25(1 Suppl):S15-26
7. WHO Multicentre Growth Reference Study G, de Onis M. WHO Child Growth Standards based on length/height, weight and age. Acta Pædiatrica 2006;95:76-85
8. Moffitt TE, Arseneault L, Belsky D, et al. A gradient of childhood self-control predicts health, wealth, and public safety. Proceedings of the National Academy of Sciences 2011;108(7):2693-98
9. Caspi A, Moffitt TE, Newman DL, et al. Behavioral observations at age 3 years predict adult psychiatric disorders: Longitudinal evidence from a birth cohort. Archives of General Psychiatry 1996;53(11):1033-39
10. Villar J, Altman D, Purwar M, et al. The objectives, design and implementation of the INTERGROWTH-21st Project. BJOG: An International Journal of Obstetrics & Gynaecology 2013;120:9-26.
11. Villar J, Cheikh Ismail L, Staines Urias E, et al. The satisfactory growth and development at 2 years of age of the INTERGROWTH-21st Fetal Growth Standards cohort support its appropriateness for constructing international standards. American Journal of Obstetrics and Gynecology 2018;218(2, Supplement):S841-S54.e2
12. Papageorghiou AT, Ohuma EO, Altman DG, et al. International standards for fetal growth based on serial ultrasound measurements: the Fetal Growth Longitudinal Study of the INTERGROWTH-21st Project. Lancet 2014;384(9946):869-79
13. Villar J, Giuliani F, Bhutta ZA, et al. Postnatal growth standards for preterm infants: the Preterm Postnatal Follow-up Study of the INTERGROWTH-21st Project. Lancet Glob Health 2015;3(11):e681-91
14. Marlow N, Abbott J, Field D, et al. Report of a BAPM/RCPCH Working Group: Classification of health status at 2 years as a perinatal outcome. London, UK: British Association of Perinatal Medicine, 2008:23
15. Fernandes M, Stein A, Newton CRJ, et al. The INTERGROWTH-21st Project Neurodevelopment Package: A novel method for the multi-dimensional assessment of neurodevelopment in pre-school age children PLoS ONE 2014;9((11): e113360)
16. Murray E, Fernandes M, Newton CR, et al. Evaluation of the INTERGROWTH-21st Neurodevelopment Assessment (INTER-NDA) in 2 year-old children. PloS one 2018;13(2):e0193406
17. Harkness J, Pennell B, Villar A, et al. Translation procedures and translation assessment in the World Mental Health Survey Initiative. The WHO World Mental Health Surveys: Global Perspectives on the Epidemiology of Mental Disorders 2008:91-113
18. Adoh TO, Woodhouse JM. The Cardiff acuity test used for measuring visual acuity development in toddlers. Vision Research 1994;34(4):555-60
19. Achenbach TM, Rescorla LA. *Manual for the ASEBA Preschool Forms & Profiles*. Burlington, USA: University of Vermont, Research Center for Children, Youth, & Families, 2000
20. WHO MRSG, Onis Md. WHO motor development study: Windows of achievement for six gross motor development milestones. . Acta Pædiatrica 2006;S450:86-95
21. Adams RJ, Courage ML. Contrast sensitivity in the 24- and 36-month-olds as assessed with contrast sensitivity card procedure. Optometry & Vision Science 1993;70(2):97-101
22. Ohuma EO, Hoch L, Cosgrove C, et al. Managing data for the international, multicentre INTERGROWTH-21st Project. BJOG: An International Journal of Obstetrics & Gynaecology 2013;120:64-70
23. Villar J, Fernandes M, Purwar M, et al. Neurodevelopmental milestones and associated behaviours are similar among healthy children across diverse geographical locations. Nature Communications 2019;10(1):511
24. Villar J, Papageorghiou AT, Pang R, et al. The likeness of fetal growth and newborn size across non-isolated populations in the INTERGROWTH-21st Project: the Fetal Growth Longitudinal Study and Newborn Cross-Sectional Study. The Lancet Diabetes & Endocrinology 2014;2(10):781-92
25. Harrell FE, Davis C. A new distribution-free quantile estimator. Biometrika 1982;69(3):635-40
26. Ertem IO, Dogan DG, Gok CG, et al. A guide for monitoring child development in low-and middle-income countries. Pediatrics 2008;121(3):e581-e89
27. Ozturk Ertem I, Krishnamurthy V, Mulaudzi MC, et al. Validation of the International Guide for Monitoring Child Development demonstrates good sensitivity and specificity in four diverse countries. Acta Paediatrica 2019;108(6):1074-86
28. McCoy DC, Waldman M, Team CF, et al. Measuring early childhood development at a global scale: Evidence from the Caregiver-Reported Early Development Instruments. Early Childhood Research Quarterly 2018;45:58-68

For peer review only - http://bmjopen.bmj.com/site/about/guidelines.xhtml

29. Daniels MC, Adair LS. Growth in young Filipino children predicts schooling trajectories through high school. The Journal of nutrition 2004;134(6):1439-46
30. Feinstein L. Inequality in the early cognitive development of British children in the 1970 cohort. Economica 2003;70(277):73-97
31. Victora CG, Adair L, Fall C, et al. Maternal and child undernutrition: consequences for adult health and human capital. The lancet 2008;371(9609):340-57
32. Piaget J, Cook M. *The origins of intelligence in children*: International Universities Press New York, 1952.
33. Engle PL, Black MM, Behrman JR, et al. Strategies to avoid the loss of developmental potential in more than 200 million children in the developing world. The Lancet 2007;369(9557):229-42
34. de Onis M, Grarza C, Victora C, et al. The WHO multicentre growth reference study (MGRS): Rationale, planning, and implementation. Food and Nutrition Bulletin 2004;25
35. De Onis M, Garza C, Victora CG, et al. The WHO Multicentre Growth Reference Study: planning, study design, and methodology. Food and nutrition bulletin 2004;25:S15-S26

For peer review only

For peer review only - http://bmjopen.bmj.com/site/about/guidelines.xhtml

Table 1 Prenatal, perinatal and neonatal characteristics of children who completed the INTER-NDA in the INTERGROWTH-21st Project compared to those lost to follow-up.

**Prenatal, perinatal and neonatal characteristics**	**Children contributing to INTERGROWTH-21st international INTER-NDA standards (n=1209)**	**Children lost to follow-up (n=331)**
	Mean (SD) or number (%)	Mean (SD) or number (%)
Maternal age at recruitment, years	28.4 (3.8)	27.4 (4.3)
Maternal body-mass-index, kg/m^2	23.2 (3.0)	23.6 (2.8)
Multiple gestation	n=0 (0.0%)	n=0 (0.0%)
Chronic maternal illness	n=96 (8.1%)	n=26 (7.9)
Maternal infections (including HIV, rubella, syphilis, hepatitis B, CMV, toxoplasmosis, tuberculosis and malaria)	n=0 (0.0%)	n=1 (0.3)
Maternal haemoglobin (g/dL)	12.4 (1.0)	12.4 (1.0)
Maternal malignancy	n=0 (0.0%)	n=0 (0.0%)
Maternal substance abuse (including alcohol) and smoking	n=0 (0.0%)	n=0 (0.0%)
Maternal use of teratogenic drugs during pregnancy	n=628 (53.2)	n=222 (67.1%)
Maternal prenatal anxiety and depression/mental stress	n=0 (0.0%)	n=0 (0.0%)
Maternal preeclampsia and eclampsia	n=10 (0.9%)	n=4 (1.2%)
Placental structural anomalies	n=0 (0.0%)	n=0 (0.0%)
Fetal growth restriction	n=67 (5.7%)	n=14 (4.2)
Gestational age at delivery, weeks[a]	39.6 (1.2)	39.3 (1.5)
Birth weight, kg[a]	3.2 (0.4)	3.2 (0.5)
Birth length, cm[a]	49.2 (1.8)	49.0 (2.1)
Head circumference at birth, cm[a]	34.0 (1.2)	34.0 (1.3)
Apgar at 5 min[a]	9.5 (0.6)	9.6 (0.7)
Age at hospital discharge, days[b]	3.0 (2.0, 4.0)	2.0 (1.0, 3.0)
Boys[a]	n=564 (47.8)	n=160 (48.3)
Hyperbilirubinaemia[a]	n=49 (4.1)	n=18 (5.5)
Respiratory distress syndrome[a]	n=16 (1.4)	n=7 (2.1)
Transient tachypnoea of the newborn[a]	n=11 (0.9)	n=12 (3.6)
Exclusive breastfeeding at hospital discharge[a]	n=1097 (93.0)	n=300 (90.9)

INTER-NDA: The INTERGROWTH-21st Neurodevelopment Assessment
Data are mean (SD) or number (%) unless otherwise specified.
Missing data below 2% for all variables.
[a]Mean (SD)
[b]Median (interquartile range).
NICU: neonatal intensive care unit; ECD: early child development

For peer review only - http://bmjopen.bmj.com/site/about/guidelines.xhtml

Table 2 Postnatal morbidity between 1 and 2 years of age of children contributing to INTERGROWTH-21st international INTER-NDA standards.

**Morbidity between 1 and 2 years of life**	**Children contributing to INTERGROWTH-21st international INTER-NDA standards (n=1209)**
Hospitalised at least once	113 (9.4)
Total number of days hospitalised[a]	2 (1, 3)
Any prescription provided by a health care practitioner	712 (59.1)
Antibiotics (≥3 regimens)	142 (11.8)
Iron/folic acid/vitamin B12/other vitamins	194 (16.1)
Up-to-date with local vaccination policies	1136 (94.4)
Otitis media/Pneumonia/Bronchiolitis	88 (7.3)
Parasitosis/Diarrhoea/Vomiting	43 (3.6)
Exanthema/skin disease	150 (12.5)
Urinary tract infection/pyelonephritis	5 (0.4)
Fever ≥3 days (≥3 episodes)	134 (11.1)
Other infections requiring antibiotics	40 (3.3)
Asthma	13 (1.1)
Gastro-oesophageal reflux	3 (0.2)
Cow's milk protein allergy	8 (0.7)
Food allergies	13 (1.1)
Injury or trauma	27 (2.2)
Any condition requiring surgery	9 (0.7)

INTER-NDA: The INTERGROWTH-21st Neurodevelopment Assessment
Data are number (%) unless otherwise specified.
Missing data below 2% for all variables.
[a]Median (interquartile range).
ECD: early child development

For peer review only

Table 3 Visual acuity and contrast sensitivity centiles, measured on the Cardiff Tests, in the normative sample of the international INTER-NDA Standards.

	**Pooled Centiles (n=1209)**	**Girls (n=628)**	**Boys (n=581)**	**p-value**
**Visual Acuity (logMar)**				
**c10**	0.3	0.3	0.3	
**c25**	0.2	0.2	0.2	
**c50**	0.2	0.2	0.2	
**c75**	0.1	0.1	0.1	
**c90**	0.1	0.1	0.1	
Median (IQR)	0.2 (0.1, 0.2)	0.2 (0.1, 0.2)	0.2 (0.1, 0.2)	0.463
**Contrast Sensitivity (%)**				
**c10**	2.0	2.0	2.0	
**c25**	1.8	1.6	1.9	
**c50**	1.5	1.5	1.5	
**c75**	1.0	1.0	1.0	
**c90**	1.0	1.0	1.0	
Median (IQR)	1.5 (1.0, 2.0)	1.5 (1.0, 1.5)	1.5 (1.0, 2.0)	0.303

INTER-NDA: The INTERGROWTH-21st Neurodevelopment Assessment
*p value from Wilcoxon rank-sum test.
IQR: inter-quartile range

For peer review only

Table 4 The INTERGROWTH-21st Project International INTER-NDA standards for child development at two years of age.

INTER-NDA domain	Pooled Centiles (n=1181)						
	**c3**	**c10**	**c25**	**c50**	**c75**	**c90**	**c97**
**Cognitive[1]**	27.4	38.5	62.2	79.5	88.8	92.6	99.6
**Fine motor[1]**	17.5	25.7	74.2	91.4	100.0	100.0	100.0
**Gross motor[1]**	31.1	51.7	66.7	81.6	100.0	100.0	100.0
**Language[1]**	12.1	17.8	45.7	71.7	88.5	95.1	100.0
**Positive behaviour[1]**	37.8	51.4	70.0	90.0	100.0	100.0	100.0
**Negative behaviour[2]**	0.0	0.0	0.0	25.0	25.0	50.0	76.5

INTER-NDA: The INTERGROWTH-21st Neurodevelopment Assessment
[1]For these domains, higher scores reflect better outcomes
[2]For negative behaviour, lower scores reflect better outcomes

For peer review only

Table 5 Evaluation of the INTER-NDA against pre-established feasibility criteria for use of an early child development assessment in a low-middle income setting.

	**Does INTER-NDA fulfil the criteria?**	**Additional details**
**World Bank Toolkit for Examining ECD[1]**		
Psychometrically adequate, valid and reliable	Yes	ICCs 0.74 and 0.88 (p<0.001) between BSID-III and INTER-NDA for cognitive, language and motor domains; internal consistency 0.56-0.80[2]. Inter-rater reliability: k=0.70, 95% CI: 0.47–0.88); test-re test reliability: k=0.79, 95%CI: 0.48–0.96[3].
Balanced in terms of number of items at the lower end to avoid children with low scores	Yes	Age range of items: 6-36 months[3]
Enjoyable for children to take (e.g. interactive, colourful materials)	Yes	
Relatively easy to adapt to various cultures	Yes	Adapted via cultural customisation session during training and currently in use in 12 countries (Brazil, India, Italy, Kenya, Pakistan, Thailand, South Africa, Mexico, Grenada, Finland, Guatemala, Democratic Republic of Congo; www.inter-nda.com)
Easy to use in low-resource settings, e.g. not requiring much material	Yes	See Murray, Fernandes, Newton, et al., 2018 for image of kit[2]; cost <GBP 120.00; no fee per use; manuals and forms freely available at www.intergrowth21.org.uk
Not too difficult to obtain or too expensive	Yes	See above
Able to be used in a wide age range	Moderately narrow age range	22 to 30 months
**Fischer et al's feasibility criteria for use of developmental screening tools at primary healthcare level in low-middle income settings[4]**		
Results understood by health workers	Yes	Centiles
Reliable	Yes	See above
Valid	Yes	See above
Acceptable to caregivers	Yes	
Provides information that is relevant to primary care providers	Yes	Centiles
Information that can be used for referrals of early intervention	Yes	Centiles
Information that is useful for anticipatory guidance	Unknown	
Results understood by caregivers	Yes	
Staff members have the expertise to answer questions	Yes	Session on maternal questions and responses included in training package.
Access to application	Yes	Freely accessible at www.intergrowth-21.org.uk
Training involved	Yes	Time taken to train assessors in the INTER-NDA: 1 day for ≤3 assessors, 2 days for 3-5 assessors, 3 days for 5-10 assessors

For peer review only

How long it takes to administer the tool	15 minutes	
Cover multiple areas of child development	Yes	Cognition, language, fine and gross motor skills, and behaviour (positive, negative and global)[3]
Cost of the tool	Minimal	Cost of kit <GBP 120.00; no fee per use; manuals and assessment forms freely available at www.intergrowth21.org.uk. Tablet/phone based data collection application (INTER-NDA E-form) optional.
Minimal adaptation needed	Yes	Sessions on cultural customisation and translation included in training
Educational level of staff members	Secondary education	Results of comparison between field workers and specialists presented in Table S6 and in text
How many staff members to administer the tool	1	
Local norms available	International references available	Normative sample drawn from a prospectively recruited sample of 2 year-olds from Brazil, India, Italy, Kenya and the UK with confirmed optimal nutritional, health and developmental status during the first 1000 days of life.
Space	Minimal	Storage of kit and forms/table. See Murray, Fernandes, Newton, et al., 2018 for image of kit[2]

ICCs = interclass correlations.

[1]Fernald LCH, Kariger P, Engle P, Raikes A. Examining Early Child Development in Low-Income Countries: A Toolkit for the Assessment of Children in the First Five Years of Life. Washington DC: The World Bank, 2009.

[2]Murray E, Fernandes M, Newton CR, et al. Evaluation of the INTERGROWTH-21st Neurodevelopment Assessment (INTER-NDA) in 2 year-old children. *PloS One* 2018; 13: e0193406.

[3]Fernandes M, Stein A, Newton CRJ, et al. The INTERGROWTH-21st Project Neurodevelopment Package: A novel method for the multi-dimensional assessment of neurodevelopment in pre-school age children *PLoS One* 2014; **9**: e113360.

[4]Fischer VJ, Morris J, Martines J. Developmental screening tools: feasibility of use at primary healthcare level in low-and middle-income settings. *J Health Popul Nutr* 2014; **32**: 314.

For peer review only

Figure Titles, Legends and Captions

Figure 1
Title: Analytical and statistical strategy for the construction of the international INTERGROWTH-21st Project INTER-NDA Standards.

Legend: INTER-NDA: INTERGROWTH-21st Project Neurodevelopment Assessment

Figure 2

Title: Participant flow for the INTERGROWTH-21st Project Infant Follow-up Study cohort at two years of age.

Legend: FGLS: Fetal Growth Longitudinal Study; INTER-NDA: INTERGROWTH-21st Project Neurodevelopment Assessment

Figure 3
Title: The INTERGROWTH-21st Project International INTER-NDA standards for child development at two years of age.
Caption: INTER-NDA 3rd to 97th centile ranges for two year-old children are presented. These are based on scaled INTER-NDA standardized domain scores. Scores falling in the yellow zone correspond to scores between the 10th and 3rd centiles; scores in the orange zone correspond to scores <3rd centile. Clinical judgment should determine whether further developmental assessment is warranted for children with scores in the yellow and orange zones, and the urgency of such referrals.

For peer review only

For peer review only - http://bmjopen.bmj.com/site/about/guidelines.xhtml

For peer review only

For peer review only - http://bmjopen.bmj.com/site/about/guidelines.xhtml

Figure 1 Analytical and statistical strategy for the construction of the international INTERGROWTH-21st Project INTER-NDA Standards.

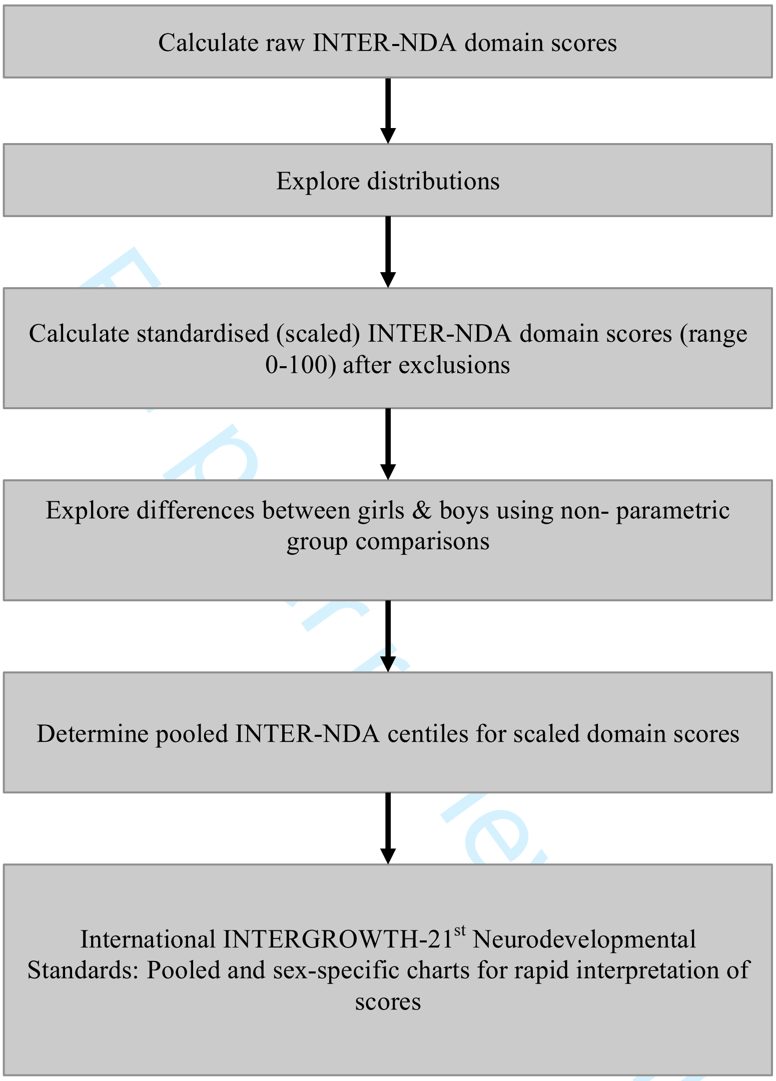

For peer review only - http://bmjopen.bmj.com/site/about/guidelines.xhtml

For peer review only

For peer review only - http://bmjopen.bmj.com/site/about/guidelines.xhtml

Figure 2 Participant flow for the INTERGROWTH-21st Project Infant Follow-up Study cohort at two years of age.

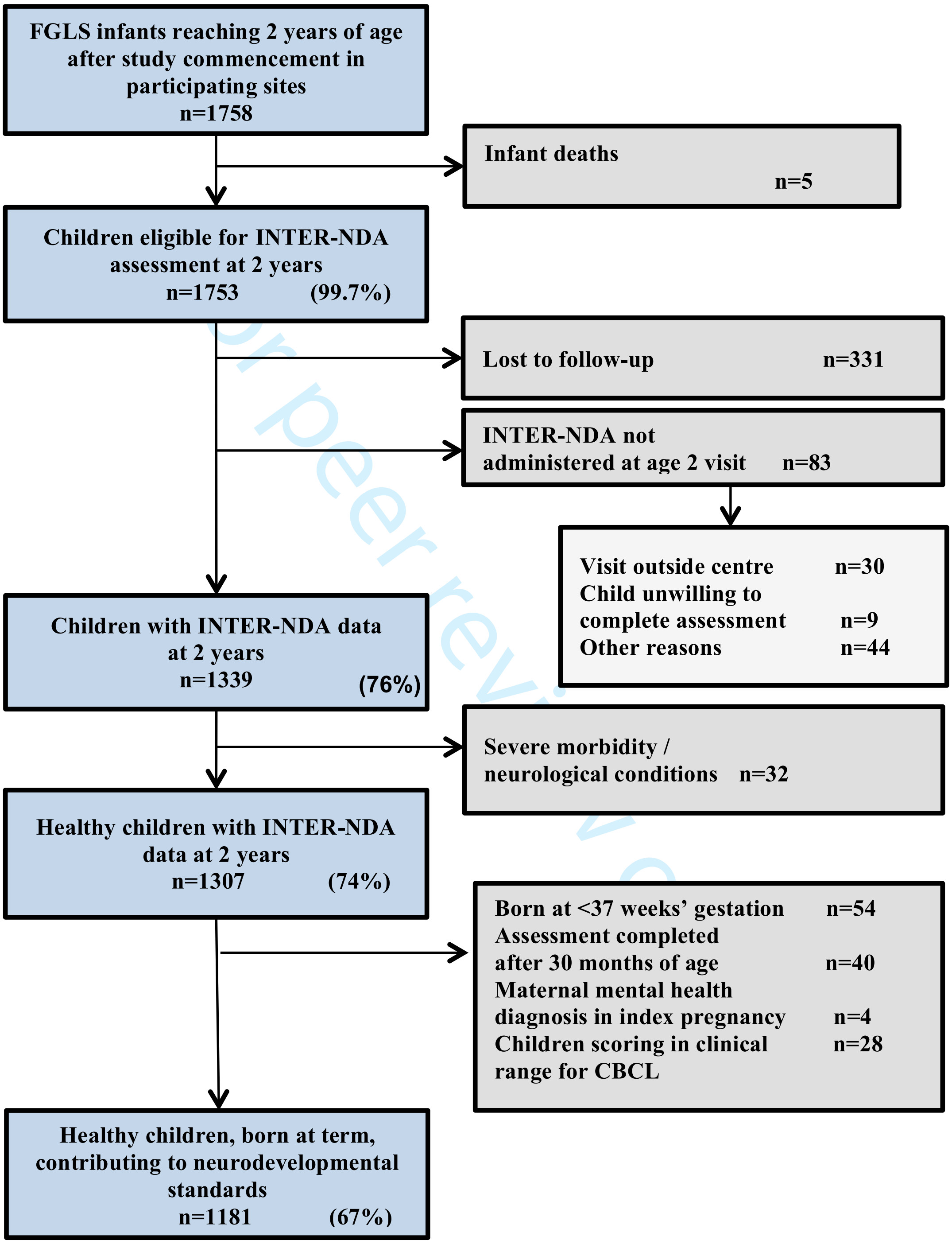

FGLS: Fetal Growth Longitudinal Study; INTER-NDA: INTERGROWTH-21st Project Neurodevelopment Assessment

For peer review only - http://bmjopen.bmj.com/site/about/guidelines.xhtml

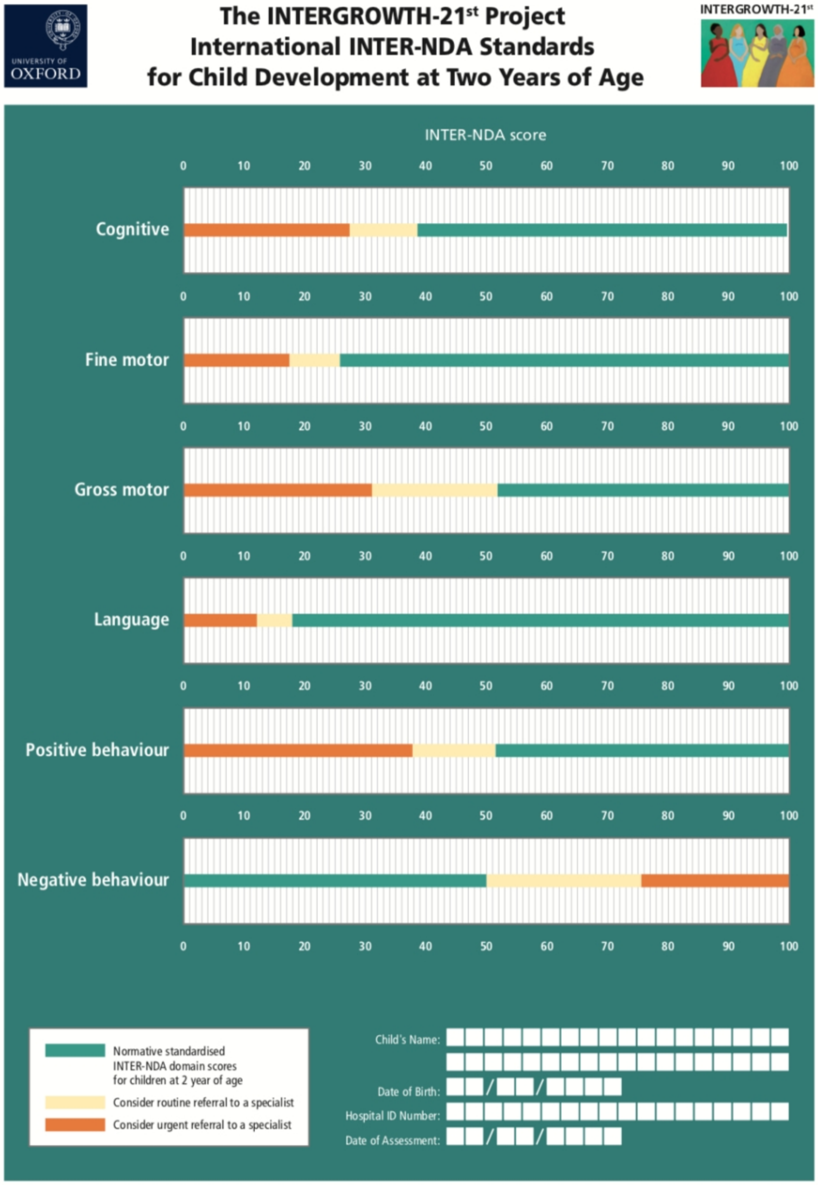

INTER-NDA 3rd to 97th centile ranges for two year-old children are presented. These are based on scaled INTER-NDA standardized domain scores. Scores falling in the yellow zone correspond to scores between the 10th and 3rd centiles; scores in the orange zone correspond to scores <3rd centile. Clinical judgment should determine whether further developmental assessment is warranted for children with scores in the yellow and orange zones, and the urgency of such referrals.

Supplementary Information

Supplementary Information S1 Characteristics of normative scales and thresholds used to identify neurodevelopmental impairment in commonly used developmental assessments for two year-old children.

Supplementary Information S2 Factors associated with poor neurodevelopmental outcomes in young children: Characteristics of the normative sample for the INTERGROWTH-21st Project International INTER-NDA standards, and for other neurodevelopmental tools evaluating two year-old children.

Supplementary Information S3 The INTERGROWTH-21st Fetal Growth Longitudinal Study (FGLS) individual participant entry criteria for mothers at the time of antenatal booking in the first trimester of pregnancy.

Supplementary Information S4 The INTERGROWTH-21st Neurodevelopment Assessment (INTER-NDA) data recording form.

Supplementary Information S5 The INTERGROWTH-21st Neurodevelopment Assessment (INTER-NDA) Protocol Adherence Checklist.

Supplementary Information S6 Results of the INTERGROWTH-21st Neurodevelopment Assessment (INTER-NDA) standardized evaluations between specialist and non-specialist assessors to (i) score and (ii) administer the INTER-NDA

Supplementary Information S7 The INTERGROWTH-21st Neurodevelopment Assessment (INTER-NDA) scoring system and interpretation of domain scores.

Supplementary Information S8 Formulae and tables for the conversion of raw scores to standardised (scaled) scores (range 0-100).

Supplementary Information S9 INTER-NDA domain scores centiles including and excluding children scoring above the CBCL threshold for clinical (97th centile) problems.

Supplementary Information S10 Comparison of INTERGROWTH-21st Neurodevelopment Assessment (INTER-NDA) domain scores between two year-old girls and boys

Supplementary Information S11 INTERGROWTH-21st Project Study Protocol (see separate file)

Supplementary Information S12 STROBE checklist (see separate file)

For peer review only

S1 Characteristics of normative scales and thresholds used to identify neurodevelopmental impairment in commonly used developmental assessments for two year-old children.

Tool	Subscales measured	Characteristics of normative sample	Cut-off score for neurodevelopmental delay	Details on scoring and interpretation of results
The Bayley Scales of Infant Development – III edition (BSID III) [a]*	Cognitive, expressive and receptive communication, fine and gross motor, adaptive behaviour, social emotional.	Country: USA<br>Year: January and October 2004<br>Sample Size: 24-month normative sample for cognitive, language and motor scales: 100 children (totally 1700 children aged 16 days through 43 months 15 days divided into 17 age groups of 100 children each). Normative sample for the social-emotional scale was based on 456 children and the adaptive behaviour scale was based on 1,350 children.<br>Sample characteristics: Sample selected to match the 2000 United States census.	Several criteria: **25% delay** in functioning when compared to same age peers; based on SD (**< -1 SD** i.e. cut-off thresholds of 85 for moderate impairment; **< -2 SD** i.e. cut-off thresholds of 70 for severe impairment) or **performance of a certain number of months below** the child's chronological age[1].	Scoring for every item is either 1 (credit) or 0 (no credit).<br><br>Scaled scores, composite scores, growth scores, centiles, & age-equivalents are obtained from raw scores.
The Malawi Developmental Assessment Tool (MDAT)[b]*	Gross motor, fine motor, language and social.	Country: Malawi<br>Year: June 2006 to July 2007<br>Sample size: 1426 normal healthy children aged 0 to 6 years<br>Sample characteristics: Those born preterm at <32 weeks' gestation, or with significant malnutrition using WHO criteria, medical problems or significant neurodisability were excluded.	**<25%** (upper limit of lowest quartile)	Plot children on MDAT normal reference ranges, corresponding to 25%, 50% and 75% of children passing each item.
The Griffiths Mental Development Scales[c]*	Locomotor, expressive and receptive language, personal-social, hand and eye coordination, performance, practical reasoning.	Country: UK and Ireland<br>Year: 1960s, revised in 2015<br>Sample size: 1026 children<br>Sample characteristics: National representative sample of children in UK; stratified according to geographical region and proportionate to the 1997 ONS population ratios[1].	Centiles and z -scores; **z score < -2 or SD< -2** indicates significant developmental delay on that subscale.	Raw scores are converted into z scores, developmental quotients and centile scores – these are used to obtain developmental age equivalents. A general quotient may also be obtained.
Pre-school version of Child Behavior Checklist (CBCL)[d]*	Problem and syndrome scales for emotionally reactive; anxious/depressed; somatic complaints; withdrawn; sleep problems; attention problems; aggressive behaviour.	Country: USA<br>Year: 1979, 2000<br>Sample size: Originally normed on 1728 US children.<br>Sample characteristics: -<br><br>Note: Multicultural norms available.	**>93rd centile** is abnormal (norms vary according to societies, and map onto the Diagnostic and Statistical Manual for Mental Disorders).<br><br>Any score that falls below the 93rd centile is considered normal, scores between the 93-97th centile are borderline clinical, and any score above the 97th centile is considered to be in the clinical range.	Different norms for different societies. The CBCL uses software to generate raw score; T score and centile score for each problem score as well as a total problem score, which ranges from 0 to 200. The standard scores are scaled so that 50 is average for the child's age and sex, with a standard deviation of 10 points. Higher scores indicate greater problems.
The Rapid Neurodevelopmental Assessment	Primitive reflexes, gross motor, fine motor, vision,	Country: Bangladesh<br>Year: 2010<br>Sample size: 81 children aged ≥3 to 24 months in urban (n = 47) and rural	**< -2 SD**: threshold for severe impairment;<br>**< -1 SD:** threshold for mild	For every item, the severity of functional limitations was determined as mild, moderate or severe limitation.

For peer review only

(RNDA)[e]*	hearing, speech, cognition, behaviour, and seizures.	(n = 34) community-based populations<br>Sample characteristics: 15% did not 'look' properly nourished, parental concerns regarding child development were expressed in 8% and for 50% at least one parent was illiterate.	impairment.<br>If low scores in >1 domain; the child is classified as having 'any' neurodevelopmental impairment.	
The Ages and Stages Questionnaire III edition (ASQ III)[f]	Communication, gross motor, fine motor, problem solving and personal-social.	Country: USA<br>Year: January 2004 and June 2008<br>Sample Size: 15,138 children (1,443 aged 24 months).<br>Sample characteristics: 76% of the sample had one or no known risk factor, 19% had 2 risk factors and 4% 3 or more risk factors. Risk factors were defined as extreme poverty, maternal age ≤19 years, maternal education <12th grade; involvement of child protective services with the family for abuse and/or neglect; medical risk, including prematurity; and infant's birth weight less than 3 pounds, 5 ounces.	**< -2SD**	Item scoring: 0 = not true, 1 = somewhat / sometimes true, or 2 = very true or often true of the child.
The Parents' Evaluation of Developmental Status (PEDS)[g]	General development.	Country: USA<br>Year: 1997<br>Sample size: 2823 families<br>Sample characteristics: Families from varying backgrounds, including SES and ethnicity.	Table for using scores to identify parental difficulties, non-significant concerns, one significant concern or two or more significant concerns by shading boxes based on scores. These are then used to select associated algorithms for further screening and/or referral.	Eight page booklet used to score the PEDS response form; an algorithm uses these scores to identify associated pathways for further screening and/or referral.
Caregiver-reported Early Developmental Instruments (CREDI)[h]	Long Form: Motor, cognitive, language, social-emotional and overall.<br><br>Short form: Overall development.	Countries: Brazil, Cambodia, Chile, Colombia, Ghana, Guatemala, India, Nepal, Philippines and USA<br>Year: 2017-2018<br>Sample size: 7807 children aged 0-35 months<br>Sample characteristics: Children with an "ideal home environment" defined through maternal educational attainment<br>(college or higher), and the number of activities done by adults with the child in the last 3 days (at least 4 out of the 6 MICS home stimulation activities); authors acknowledge that "although the data were representative for local populations in Brazil, Ghana, Tanzania, and Zambia, the overall sample is not representative of any country or a global population of children".	Raw scaled scores, norm referenced standardised scores, and z scores.	Uses the CREDI software package in R.
Denver Development Screening Test II (DDST II)[i]	Personal social, fine motor adaptive, language and gross motor.	Country: USA<br>Year: 1980s and 1990<br>Sample size: 2096 children<br>Sample characteristics: Children from Colorado; based on 1988, 1989 and then the 1990 US census population.	**Centile ranks** (25th, 50th, 75th, and 90th) are displayed as bar graphs and reflect the ages at which 25%, 50%, 75% and 90% of typically developing children in the standardisation sample completed the task.<br>Overall categories: Normal, and suspect.	Approximates a growth curve in its display of norms over time. The number of scores a child received below the normal expected range classifies the child as within normal, suspect, or delayed. If the child is suspect it is recommended that rescreening occur in 1-2 week.
Guide for Monitoring Child	Parental concerns, expressive language	Country: Turkey<br>Year: 1980s and 1990	<10th centile for any domain; if a child did not demonstrate ≥1 of the age-	Age at which >90% of the study sample performed each milestone was computed.

Development (GMCD)[j]	and communication, receptive language, relationship (social-emotional), play (social-emotional and cognitive) and self-help.	Sample size: 30 children in each age range<br>Sample characteristics: Sample selected as per WHO recommendations for a 'prescriptive sample' i.e. children were born healthy singletons with birth weight ≥2500 g and gestational age ≥37 weeks and had received preventive health care at 2 university-affiliated community well-child care clinics in Ankara from birth. The sample's growth was between the 5th and 95th centiles since birth; they had received and complied with the free iron prophylaxis available to children in Turkey (or had normal haemoglobin screens within 1 month of the study); were healthy, and growing normally, at their paediatric evaluation. They had not had "any health-related problems since birth apart from acute minor illnesses."	appropriate milestones, the GMCD interpretation was classified as "requires follow-up evaluation with or without intervention."	

[a]©Pearson. Bayley Scales of Infant and Toddler Development, Third Edition (Bayley-III) - Training. Available from: 98 http://www.pearsonclinical.co.uk
[b]Gladstone M, Lancaster GA, Umar E, Nyirenda M, Kayira E, van den Broek NR, Smyth RL. The Malawi Developmental Assessment Tool (MDAT): the creation, validation, and reliability of a tool to assess child development in rural African settings. *PLoS medicine* 2010; **7:** e1000273.
[c]Luiz D, Barnard A, Knoesen N, Kotras N, Horrocks S, McAlinden P, Challis D, O'Connell R. Griffiths Mental Development Scales—Extended Revised: Two to Eight Years: Administration Manual. Hogrefe, Oxford, UK 2006.
[d]Achenbach TM. Manual for the Child Behavior Checklist/4-18 and 1991 profile. University of Vermont, Department of Psychiatry 1991.
[e]Khan NZ, Muslima H, Begum D, Shilpi AB, Akhter S, Bilkis K, Begum N, Parveen M, Ferdous S, Morshed R, Batra M. Validation of rapid neurodevelopmental assessment instrument for under-two-year-old children in Bangladesh. *Pediatrics* 2010; **125:** e755-62.
[f]Squires J, Bricker DD, Twombly E. Ages & stages questionnaires. Baltimore, MD: Paul H. Brookes 2009.
[g]Glascoe FP. Collaborating with parents: Using Parents' Evaluation of Developmental Status to detect and address developmental and behavioral problems. Ellsworth & Vandermeer Press 1998.
[h]McCoy DC, Waldman M, Team CF, Fink G. Measuring early childhood development at a global scale: Evidence from the Caregiver-Reported Early Development Instruments. *Early Childhood Research Quarterly* 2018; **45:** 58-68.
[i]Frankenburg WK, Dodds J, Archer P, Shapiro H, Bresnick B. The Denver II: a major revision and restandardization of the Denver Developmental Screening Test. *Pediatrics* 1992; **89:** 91-7.
[j]Ertem IO, Dogan DG, Gok CG, Kizilates SU, Caliskan A, Atay G, Vatandas N, Karaaslan T, Baskan SG, Cicchetti DV. A guide for monitoring child development in low-and middle-income countries. *Pediatrics* 2008; **121:** e581-9.
[*]Candidate tools contributing to the development of the INTERGROWTH-21st Neurodevelopment Assessment (INTER-NDA).

For peer review only

S2 Factors associated with poor neurodevelopmental outcomes in young children[§#]: Characteristics of the normative sample for the INTERGROWTH-21st Project international INTER-NDA Standards, and for other neurodevelopmental tools evaluating two year-old children.

**Factors affecting child development**	**INTERGROWTH-21st Project sample characteristics (N=1209)**	**Characteristics of normative samples, reported and accessible in the public domain, for the following child developmental tools:**									
		**BSID III[a]**	**MDAT[b]**	**Griff-iths[c]**	**RNDA[d]**	**CBCL[e]**	**ASQ III[f]**	**PEDS[g]**	**CREDI[h]**	**DDST II[i]**	**GMCD[j]**
**PRENATAL FACTORS**											
**Genetic Factors:**											
Chromosomal abnormalities	Yes, n=0 (0.0%)	No	Yes^	No	No	No	Yes^	No	No	No	Yes^
Genetic syndromes	Yes, n=0 (0.0%)	No	No	No	No	No	No	No	No	No	No
Sporadic mutations	Yes, n=0 (0.0%)	No	No	No	No	No	No	No	No	No	No
Gene polymorphisms	Yes, n=0 (0.0%)	No	No	No	No	No	No	No	No	No	No
Trinucleotide repeat disorders	Yes, n=0 (0.0%)	No	No	No	No	No	No	No	No	No	No
Metabolic disorders	Yes, n=0 (0.0%)	No	No	No	No	No	No	No	No	No	No
**Intrauterine (materno-feto-placental) factors:**											
Chronic maternal illness	Yes, n=12 (0.9%)[1]	No	No	No	No	No	No	No	No	No	No
Maternal infections (including HIV, rubella, syphilis, hepatitis B, CMV, toxoplasmosis, tuberculosis and malaria)	Yes, n=0 (0.0%)	No	No	No	No	No	No	No	No	No	No
Maternal anaemia	Yes, mean Hb 12.5 (SD1.0)	No	No	No	No	No	No	No	No	No	No
Maternal malignancy	Yes, n=0 (0.0%)	No	No	No	No	No	No	No	No	No	No
Poor maternal nutrition (under- and overnutrition)	Yes, mean BMI 23.4 (SD 7.5)	No	No	No	No	No	No	No	No	No	No
Maternal substance abuse (including alcohol) and smoking	Yes, n=0 (0.0%)	No	No	No	No	No	No	No	No	No	No
Teratogenic drugs	Yes, n=0 (0.0%)	No	No	No	No	No	No	No	No	No	No
Toxins (lead, mercury, and arsenic)	No	No	No	No	No	No	No	No	No	No	No
Anxiety and depression/mental stress	Yes, n=0 (0.0%)	No	No	No	No	No	No	No	No	No	No
Preeclampsia and eclampsia	Yes, n=0 (0.0%)	No	No	No	No	No	No	No	No	No	No
Placental structural anomalies	Yes, n=0 (0.0%)	No	No	No	No	No	No	No	No	No	No
Liquor volume (oligo and polyhydramnios)	Yes, n=28 (2.3%)	No	No	No	No	No	No	No	No	No	No
Intrauterine infections (prolonged rupture of membranes and chorioamnionitis)	Yes, n=145 (11.9%)	No	No	No	No	No	No	No	No	No	No
Fetal behaviour	No	No	No	No	No	No	No	No	No	No	No
Fetal growth restriction	Yes, n=0 (0.0%)	No	No	No	No	No	No	No	No	No	Yes^
Multiple gestation	Yes, n=0 (0.0%)	No	No	No	No	No	Yes^	No	No	No	Yes
**External factors :**											
Maternal access to health care	Yes, n=1209	No	No	No	No	No	No	No	No	No	Yes

For peer review only

	(100.0%)										Yes
Maternal access to prenatal care	Yes, n=1209 (100.0%)	No	No	No	No	No	No	No	No	No	No
Exposure to radiation	No	No	No	No	No	No	No	No	No	No	No
Trauma	Yes, n=0 (0.0%)	No	No	No	No	No	No	No	No	No	Yes
**Socio-Maternal:**											
SES/Poverty	Yes	Yes	No	No	Yes	No	Yes	No	Yes	No	Yes
Hunger due to lack of money to buy food	Yes, n=0 (0.0%)	No	No	No	No	No	Yes	No	Yes	No	Yes^
Maternal education	Yes[2]	Yes	No	No	Yes	No	Yes	No	Yes	No	No
Paternal education	No	No	No	No	Yes	No	No	No	No	No	No
Maternal age at the time of delivery	Yes; mean=28.9 years (SD 3.8)	No	No	No	No	No	Yes	No	No	No	No
Involvement of social services/child protection agencies with family	No	No	No	No	No	No	Yes	No	No	No	No
Domestic violence or abuse	No	No	No	No	No	No	Yes	No	No	No	No
State of local/regional violence	Yes	No	No	No	No	No	Yes	No	No	No	No
Maternal employment in very physically demanding work during pregnancy	Yes, n=0 (0.0%)	No	No	No	No	No	No	No	No	No	No
Maternal occupation with risk of exposure to chemicals or toxic substances	Yes, n=0 (0.0%)	No	No	No	No	No	No	No	No	No	No
**BIRTH FACTORS**											
Prematurity (born at <37 weeks' gestation)	Yes, n=0 (0.0%)	No	Yes	No	No	No	Yes	No	No	No	Yes
Birth weight <2500 g	Yes, n=43 (3.5%)	No	No	No	No	No	Yes	No	No	No	Yes
Fetal distress prior to birth as adjudged by fetal heart rate monitoring and/or cord blood sampling	Yes, n=64 (5.3%)	No	No	No	No	No	No	No	No	No	Yes^
Resuscitation of newborn	Yes, n=36 (2.9%)	No	No	No	No	No	No	No	No	No	Yes^
**POSTNATAL FACTORS**											
**Maternal Factors:**											
Maternal mental health/maternal depression	Yes, n=0 (0.0%)	No	No	No	No	No	No	No	No	No	No
Maternal exposure to abuse and violence	Yes, n=0 (0.0%)	No	No	No	No	No	Yes	No	No	No	No
Parenting style: cognitive stimulation, caregiver sensitivity and responsiveness to the child, and caregiver affect (emotional warmth or rejection of child)	No	No	No	No	No	No	No	No	Yes	No	No
**Neonatal Factors:**											
Neonatal sepsis, including congenital and intracranial infections	Yes, n=7 (0.6%)	No	No	No	No	No	Yes^	No	No	No	Yes^
Neonatal seizures	Yes, n=0 (0.0%)	No	No	No	No	No	Yes^	No	No	No	Yes^

For peer review only

Prolonged ventilation	Yes, n=0 (0.0%)	No	No	No	No	No	Yes^	No	No	No	Yes^
Hypotension requiring inotropic support	Yes, n=0 (0.0%)	No	No	No	No	No	Yes^	No	No	No	Yes^
Intraventricular haemorrhage grade 2 or greater	Yes, n=0 (0.0%)	No	No	No	No	No	Yes^	No	No	No	Yes^
Hypoxic ischaemic encephalopathy	Yes, n=1 (0.1%)	No	No	No	No	No	Yes^	No	No	No	Yes^
Developmental abnormalities	Yes, n=7 (0.6%)	No	No	No	No	No	Yes^	No	No	No	Yes^
Cardiac diagnosis	Yes, n=1 (0.1%)[3]	No	No	No	No	No	Yes^	No	No	No	Yes^
Hyperbilirubinaemia, not requiring exchange transfusion	Yes, n=49 (4.0%)	No	No	No	No	No	Yes^	No	No	No	Yes^
Use of medications, including antibiotics, in neonatal period	Yes, n=141 (11.7%)	No	No	No	No	No	Yes^	No	No	No	Yes^
Breastfeeding at postnatal discharge from hospital	Yes, n=1123 (92.9%)	No	No	No	No	No	Yes^	No	No	No	Yes^
**Infant and Toddler Factors:**											
Adequate physical growth measured as per standardised protocols and growth charts	Yes, n=1209 (100.0%)	No	Yes	No	Yes	No	No	No	Yes	No	Yes
Breastfeeding and appropriate weaning	Yes[4]	No	No	No	No	No	No	No	No	No	Yes^
Micronutrient deficiencies, including iron, iodine and zinc	Yes, n=1 (0.1%)	No	No	No	No	No	Yes^	No	No	No	Yes
Severe infectious diseases (HIV, malaria, tuberculosis, meningitis)	Yes, n=0 (0.0%)	No	No	No	No	No	Yes	No	No	No	Yes
Cerebral palsy	Yes, n=0 (0.0%)	No	No	No	No	No	Yes^	No	No	No	Yes^
Neurological disorders	Yes, n=0 (0.0%)	No	No	No	No	No	Yes^	No	No	No	Yes^
Seizures	Yes, n=0 (0.0%)	No	No	No	No	No	Yes^	No	No	No	Yes^
Long-term health issues including metabolic, endocrinological and surgical conditions	Yes, n=0 (0.0%)	No	No	No	No	No	Yes^	No	No	No	Yes^
Exposure to environmental toxins such as lead, arsenic, manganese and pesticides	Yes, varies (<10%)[k]	No	No	No	No	No	No	No	No	No	No
Exposure to radiation	Yes, varies (<10%)[k]	No	No	No	No	No	No	No	No	No	No
Exposure to social adversity (e.g. neighbourhood crime)	Yes, varies (<10%)[k]	No	No	No	No	No	No	No	No	No	No
Trauma, including road traffic accidents and non-accidental injury	Yes, n=0 (0.0%)	No	Yes	No	No	No	Yes	No	No	No	No
Significant health conditions during the first 2 years of life, requiring prolonged hospitalisation	Yes, n=0 (0.0%)	No	Yes	No	No	No	Yes^	No	No	No	Yes^
Delayed acquisition of developmental milestones or neurosdisability as reported by parents/healthcare workers	Yes, n=0 (0.0%)	No	No	No	No	Yes	Yes^	No	Yes^	No	Yes^
Parental concerns	Yes, n=0 (0.0%)	No	No	No	No	Yes	Yes^	No	No	No	Yes^

BSID III: The Bayley Scales of Infant Development – III edition (BSID III)
MDAT: The Malawi Developmental Assessment Tool
Griffiths: The Griffiths Mental Development Scales
RNDA: The Rapid Neurodevelopmental Assessment

For peer review only

CBCL: Pre-school version of Child Behavior Checklist
ASQ III: The Ages and Stages Questionnaire III edition
PEDS: The Parents' Evaluation of Developmental Status
CREDI: Caregiver-reported Early Developmental Instruments
DDST II: Denver Development Screening Test II
GMCD: Guide for Monitoring Child Development

Yes: Factor in column 1 has been reported in published literature about the tool's normative sample, or has been stated, in published literature, to have been considered in the evaluation of the tool's normative sample
No: Factor in column 1 has not been reported in published literature about the tool's normative sample, or has not been stated, in published literature, to have been considered in the evaluation of the tool's normative sample
^Implied as stated as "healthy" and "low risk", specific descriptions of each predictor not stated.
[§] Walker SP, Wachs TD, Gardner JM, Lozoff B, Wasserman GA, Pollitt E, Carter JA, International Child Development Steering Group. Child development: risk factors for adverse outcomes in developing countries. *The lancet* 2007; **369:** 145-57.
[#] Fernandes M, Srinivasan K, Menezes G, Ramchandani PG. Prenatal depression, fetal neurobehavior, and infant temperament: Novel insights on early neurodevelopment from a socioeconomically disadvantaged Indian cohort. *Development and Psychopathology* 2018; **30:** 725-42.

[1]Chronic respiratory illness, including asthma n=4 (0·3%), endocrinological conditions including hypothyroidism n=7(0·6%), other n=1 (0·08%)
[2]Primary education n=62 (5·1%); Secondary education n=281 (23·2%); Professional/technical training n=198 (16·4%); University n=668 (55·3%)
[3]Patent ductus arteriosus
[4]Duration of exclusive breastfeeding: median 5.6 months (IQR 4.0, 6.0); Age of introduction of formula feeds: median 4.2 months (IQR 2.0, 6.0); age at introduction of first solids; median 6.0 months (IQR 5.5, 6.5)

[a]Pearson. Bayley Scales of Infant and Toddler Development, Third Edition (Bayley-III) - Training. Available from: 98 http://www.pearsonclinical.co.uk
[b]Gladstone M, Lancaster GA, Umar E, Nyirenda M, Kayira E, van den Broek NR, Smyth RL. The Malawi Developmental Assessment Tool (MDAT): the creation, validation, and reliability of a tool to assess child development in rural African settings. *PLoS medicine* 2010; **7:** e1000273.
[c]Luiz D, Barnard A, Knoesen N, Kotras N, Horrocks S, McAlinden P, Challis D, O'Connell R. Griffiths Mental Development Scales—Extended Revised: Two to Eight Years: Administration Manual. Hogrefe, Oxford, UK 2006.
[d]Achenbach TM. Manual for the Child Behavior Checklist/4-18 and 1991 profile. University of Vermont, Department of Psychiatry 1991.
[e]Khan NZ, Muslima H, Begum D, Shilpi AB, Akhter S, Bilkis K, Begum N, Parveen M, Ferdous S, Morshed R, Batra M. Validation of rapid neurodevelopmental assessment instrument for under-two-year-old children in Bangladesh. *Pediatrics* 2010; **125:** e755-62.
[f]Squires J, Bricker DD, Twombly E. Ages & stages questionnaires. Baltimore, MD: Paul H. Brookes 2009.
[g]Glascoe FP. Collaborating with parents: Using Parents' Evaluation of Developmental Status to detect and address developmental and behavioral problems. Ellsworth & Vandermeer Press 1998.
[h]McCoy DC, Waldman M, Team CF, Fink G. Measuring early childhood development at a global scale: Evidence from the Caregiver-Reported Early Development Instruments. *Early Childhood Research Quarterly* 2018; **45:** 58-68.
[i]Frankenburg WK, Dodds J, Archer P, Shapiro H, Bresnick B. The Denver II: a major revision and restandardization of the Denver Developmental Screening Test. *Pediatrics* 1992; **89:** 91-7.
[j]Ertem IO, Dogan DG, Gok CG, Kizilates SU, Caliskan A, Atay G, Vatandas N, Karaaslan T, Baskan SG, Cicchetti DV. A guide for monitoring child development in low-and middle-income countries. *Pediatrics* 2008; **121:** e581-9.
[k]Eskenazi B, Bradman A, Finkton D, Purwar M, Noble JA, Pang R, Burnham O, Cheikh Ismail L, Farhi F, Barros FC, Lambert A. A rapid questionnaire assessment of environmental exposures to pregnant women in the INTERGROWTH-21st Project. *BJOG: An International Journal of Obstetrics & Gynaecology* 2013; **120:** 129-38.

For peer review only

S3 The INTERGROWTH-21st Fetal Growth Longitudinal Study (FGLS) individual participant entry criteria for mothers at the time of antenatal booking in the first trimester of pregnancy

**Maternal eligibility criteria at booking (<14 weeks of gestation)**	
1.	Aged ≥18 and <35 years.
2.	Body mass index ≥18.5 and <30 kg/m^2.
3.	Height ≥153 cm.
4.	Singleton pregnancy.
5.	A known last menstrual period with regular cycles (defined as 28 ± 4 days) without hormonal contraceptive use, or breastfeeding in the 2 months before pregnancy.
6.	Natural conception.
7.	No relevant past medical history, with no need for long- term medication (including fertility treatment and over- the- counter medicines, but excluding routine iron, folate, calcium, iodine or multivitamin supplements).
8.	No evidence of socio- economic constraints likely to impede fetal growth identified using local definitions of social risk.
9.	No use of tobacco or recreational drugs such as cannabis in the 3 months before or after becoming pregnant.
10.	No heavy alcohol use (defined as >5 units (50 ml pure alcohol) per week) since becoming pregnant.
11.	No more than one miscarriage in the two previous consecutive pregnancies.
12.	No previous baby delivered preterm (<37^{+0} weeks of gestation) or with a birthweight <2500 g or >4500 g.
13.	No previous neonatal or fetal death, previous baby with any congenital malformations, and no evidence in present pregnancy of congenital disease or fetal anomaly.
14.	No previous pregnancy affected by pre- eclampsia/eclampsia, HELLP syndrome or a related pregnancy- associated condition.
15.	No clinically significant atypical red cell alloantibodies.
16.	Negative urinalysis.
17.	Systolic blood pressure <140 mmHg and diastolic blood pressure <90 mmHg.
18.	No diagnosis or treatment for anaemia during this pregnancy (haemoglobin levels will be monitored throughout pregnancy).
19.	No clinical evidence of any other sexually transmitted diseases, including syphilis and clinical trichomoniasis.
20.	Not in an occupation with risk of exposure to chemicals or toxic substances, or very physically demanding activity to be evaluated by local standards. Also women should not be conducting vigorous or contact sports, such as scuba diving or similar activities.

For peer review only

S4 The INTERGROWTH-21st Neurodevelopment Assessment (INTER-NDA) data recording form

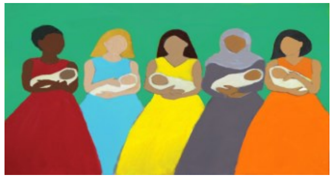

The INTERGROWTH-21st Neurodevelopment Assessment INTER-NDA

Name of child: ______________________ Date of birth: __ /__ / _____ Date of assessment: __ /__ / _____

Name of assessor: ____________________

No.	Item	Observed Performance				
1	**Builds a tower of 5 cubes**<br>*(trials=3, demonstration=3)*	5 cubes	3-4 cubes	2 cubes	No attempt	Unable to assess
2	**Names 4 colours when asked to do so**<br>*(trials=1, demonstration=0)*	Names 4 colours	Names 3 colours	Names 1 or 2 colours	Does not name any colour	Unable to assess
3	**Matches 3 cubes of same colours when requested to do so**<br>(trials=1, demonstration=1 of one colour)	Matches 3 colours	Matches 2 colours	Matches 1 colour	Does not match any colour	Unable to assess
4	**Hands the examiner one cube when asked to do so (Examiner says "Please give me one cube" & keeps palm open for 5 seconds after child has handed over 1 cube)**<br>*(trials=1, demonstration=0)*	Hands only one block within 5 seconds	Hands only one block in more than 5 seconds	Hands two or more blocks	Does not hand any block / No attempt	Unable to assess
5	**Puts the spoon in the cup when asked to do so**<br>*(trials=5, demonstration=0)*	Puts the spoon in cup in ≤3 trials	Puts the spoon in cup in 4-5 trials	Takes the spoon or the cup but does not complete action	No attempt	Unable to assess

For peer review only

6	**Matches shapes on board**<br>*(trials=5, demonstration=partial – removal only)*	All shapes in ≤3 trials	All shapes with repeated demonstration i.e. 4-5 trials	One or two shapes in 4-5 trials	No attempt	Unable to assess
7	**Matches shapes on rotated board**<br>*(trials=5, demonstration=partial – removal only)*	All shapes in ≤3 trials	All shapes with repeated demonstration i.e. 4-5 trials	One or two shapes in 4-5 trials	No attempt	Unable to assess
8	**Points correctly when asked "Where is the door/entrance to the room?"**<br>*(trials=5, demonstration=0)*	Identifies door correctly in ≤3 trials	Identifies door correctly in 4-5 trials	Attempts, but does not identify door	No attempt	Unable to assess
9	**Puts a raisin precisely inside a small opening in a bottle**<br>*(trials=1, demonstration=1, test both hands)*	Precise release of raisin into bottle with each hand	Clumsy release, raisin falls out of bottle with one or more hand	Attempts but unsuccessful release with one or more hand	No attempt	Unable to assess
10	**Drinks water from cup/bottle/sippy cup when placed in front of child**<br>*(trials=1, demonstration=0; maternal recall if observation not possible)*	Drinks water from cup/sippy cup without spilling	Drinks clumsily & spills	Attempts but unsuccessful	No attempt	Unable to assess
11	**Looks towards an object located across the room when pointed at by the examiner**<br>(trials=5)	Looks or points at object in ≤3 trials	Looks or points at object in 4-5 trials	Looks at the wrong object, or attempts but cannot identify object	No attempt	Unable to assess
12	**Pretends to drink from a toy cup when placed in front of him/her**<br>*(trials=2, demonstration=1 if not spontaneous on first attempt)*	Spontaneously	After 1 demonstration	Partial attempt after 1 demonstration	No attempt	Unable to assess
13	**Able to make a cup of tea with the toy tea set when requested by examiner (Examiner says "Can you make a cup of tea?")**<br>*(trials=2, demonstration=1 if not spontaneous on first attempt)*	Spontaneously, with pouring motion	After 1 demonstration	Partial attempt after 1 demonstration	No attempt	Unable to assess

For peer review only

14	**Feeds doll when requested to (Examiner says "Can you give the dolly some tea?")** *(trials=2, demonstration=1 if not spontaneous on first attempt)*	Spontaneously	After 1 demonstration	Partial attempt after 1 demonstration	No attempt	Unable to assess
15	**Imitates straight horizontal scribble** *(trials=5, demonstration=5)*	≤3 trials	4-5 trials; with difficulty	Attempts (hold crayon)	Cannot hold crayon	Unable to assess
16	**Identifies glitter bracelet under correct washcloth** *(trials=5, demonstration=0, test both sides)*	Finds bracelet correctly in ≤2 trails on both sides	Find bracelet correctly in 3 trials or on one side only	Find bracelet correctly in 4-5 trials or on one side only	Does not find bracelet or no attempt	Unable to assess
17	**Correctly identifies object groups using plurals** *(concurrent observation)*	Uses 5 plurals	Uses 3-4 plurals	Uses 1-2 plurals	Does not use any plurals	Unable to assess
18	**Asks for toilet by gesture or verbally** *(maternal recall)*	Always	Occasionally	Partial (only for bowel movement)	Never	Unable to assess
19	**Runs** *(maternal recall)*	Runs steadily	Attempts	Walks only	Walks with support	Unable to assess
20	**Throws a ball very near** *(trials=1, demonstration=1; test both hands)*	Good release	Unsteady release	Attempts	No attempt	Unable to assess
21	**Kicks ball** *(maternal recall)*	Kicks ball with knee flexed	Runs after ball & attempts kicking it	Walks and touches ball with foot	No attempt	Unable to assess
22	**Climbs upstairs holding rail, 2 feet/stair or in adult fashion** *(maternal recall)*	Climbs stairs alone steadily	Climbs stairs alone unsteadily	Climbs stairs with help (uses railing, holds adult's hand)	No attempt	Unable to assess
23	**Uses 2-4 syllable babble such as dada, mama but not specifically to anything or any person** *(concurrent observation)*	Spontaneously	Mimics	1 syllabe babble e.g. ba, ma, da	None	Unable to assess
24	**Use two words together** *(concurrent observation)*	Two words, appropriate use	Two words, inappropriate use	One word, appropriate use	No attempt	Unable to assess

25	**Indicates by gesture to say no**<br>*(concurrent observation or maternal recall)*	Indicates verbally or by definite gesture all the time	Indicates verbally or by definite gesture some of the time	Attempts, but incomplete indication	No attempt	Unable to assess
26	**Use of a pronoun e.g. me, my, she, he, it, I**<br>*(concurrent observation)*	≥1 pronoun in correct context	≥1 pronoun, incorrect use	Use of proper names but not pronouns	No use	Unable to assess
27	**How many words does the child use during the assessment other than mama/dada**<br>*(concurrent observation)*	≥8 words	6-7 words	4-5 words	≤3 words	Unable to assess
28	**How many sentences of 3 words or more does the child use during the assessment?**<br>*(concurrent observation)*	≥2	1	≥1 two word utterance	None	Unable to assess
29	**In how many instances does the child follow on a topic of conversation providing new information?**<br>*(concurrent observation)*	At least one, using ≥ 2 words, proving correct information	At least one, uses single words, provides correct information	Uses any number of words, provides incorrect information	Does not follow up on conversations	Unable to assess
30	**Combines word and gesture when asked**<br>*(Do not demonstrate, trials=3, use different example if mother says child does not know the one you are asking)*	Combines word and gesture completely and appropriately	Combines word and gesture completely but inappropriately	Combines word and gesture incompletely and inappropriately	Does not combine a word an gesture	Unable to assess

What is the child's native (first) language? ____________________
What is the language in which the assessment is being conducted in? ____________________
Does the child speak/understand any languages other than his/her native (first) language? ____________________

For peer review only

How often were the following behaviours in the child during the assessment?				
31	**Positive Affect**	Never or rarely	Some of the time	Most of the time
32	**Exploration**	Never or rarely	Some of the time	Most of the time
33	**Ease of engagement**	Never or rarely	Some of the time	Most of the time
34	**Cooperativeness**	Never or rarely	Some of the time	Most of the time
35	**Adaptability to change**	Never or rarely	Some of the time	Most of the time
36	**Distractibility**	Never or rarely	Some of the time	Most of the time
37	**Negative Affect**	Never or rarely	Some of the time	Most of the time

For peer review only

S5 The INTERGROWTH-21st Neurodevelopment Assessment (INTER-NDA) Protocol Adherence Checklist

	The INTERGROWTH-21st Project<br>Neurodevelopment Assessment Package<br>**Protocol Adherence Checklist**	**NDA-PAC**<br>**Page 2 of 2**

**Participant ID No.**	__ __ - __ __ __ __ __	**Researcher code: Expert**	__ __
**Child Date of Birth**	D D M M Y Y	**Researcher code: Assessor**	__ __
**Visit Date**	D D M M Y Y		

Please rate the performance of the assessor in adhering to the INTERGROWTH-21st Neurodevelopment Assessment Protocol during the administration of the following items. For the purpose of this exercise, complete adherence is defined as adherence to all aspects of the protocol as stated in the operation manual of the INTER-NDA; partial adherence is defined as adherence to half or more aspects, but not all aspects, of the protocol as stated in the operation manual of the INTER-NDA and limited adherence is defined as adherence to less than half of the aspects of the protocol as stated in the operation manual of the INTER-NDA as adjudged by the expert assessor.

**IV. The INTER-NDA**	Complete adherence	Partial adherence	Limited adherence
1. Item 1 - uses 5 red cubes, 3 trials, demonstration before each trial			
2. Item 2 - 4 cubes of different colours placed in a line, assessor does not name colours, 1 trial			
3. Item 3 - 1 demonstration, 1 trial, conceptually correct scoring (stacking & pointing accepted)			
4. Item 4 - holds hand out for 5 seconds			
5. Item 5 - correct placement of objects - handles facing child, cup & spoon not adjacent, 5 trials			
6. Item 6 - 5 trials, no demonstration, shapes near child, holds board, puts shapes back discretely			
7. Item 7 - 5 trials, no demonstration, shapes near child, rotates board on table, holds board			
8. Item 9 - 1 demonstration, 1 trial, tests both hands			
9. Item 11 - 5 trials, no demonstration, relatively slow pointing movement so child can follow			
10. Item 12 - allows time for self-symbolic play before suggestion & demonstration			
11. Item 13 - 2 trials, demonstrate if not spontaneous, involve mother if necessary			
12. Item 14 - 2 trials, demonstrate if not spontaneous, involve mother if necessary			
13. Item 15 - 5 trials, demonstration before each trial, start at upper part of paper			
14. Item 16 - 5 trials, displaces bracelet relatively slowly, tests both sides			
15. Item 20 - 1 demonstration, 1 trial, tests both hands			
16. Item 30 - uses opportunities during assessment, uses culturally appropriate combinations, informs mothers about purpose & rationale of item			
17. Assessor's interaction with the child (good verbal & nonverbal communication, sets child at ease, builds rapport, accomodates needs of the child)			
18. Assessor's interaction with the mother/caregiver (explains tests, builds rapport, involves mother in testing providing instructions to prevent compromising test)			

Name of expert researcher	
Signature	

S6 Results of the INTERGROWTH-21st Neurodevelopment Assessment (INTER-NDA) standardized evaluations between specialist and non-specialist assessors to (a) administer and (b) score the INTER-NDA

S6 (a) Comparisons in protocol adherence scores for the INTER-NDA between non-specialist and specialist assessors.

	**Median (95% CI)**		**Comparison between groups**
	**Non-specialist assessors** (n=4)	**Specialist assessors** (n=3)	
**INTER-NDA Protocol Adherence Score (Total Range: 18 – 54)**	53.0 (43.4 – 53.6)	52.0 (46.2 – 54.2)	U=27.5, p=0.8
**INTER-NDA Protocol Adherence %**	98.0 (80.4 – 99.2)	96.3 (85.5 – 99.3)	U=30.5, p=1.0

For peer review only

S6 (b) Comparisons in INTER-NDA domain scores between non-specialist and specialist assessors.

		Neurodevelopment scores (n=23)					
		**Cognition**	**Language**	**Gross motor**	**Fine motor**	**Positive behavior**	**Negative behavior**
		Mean (SD)	***Mean (SD)***	***Mean (SD)***	***Mean (SD)***	***Mean (SD)***	***Mean (SD)***
**Video/Child 1**	**Field workers** (n=10)	21.1 (2.0)[a]	17.7 (3.7)	4.6 (0.9)	4.6 (0.9)	14.9 (0.3)	2.4 (0.7)
	**Healthcare professionals** (n=13)	24.2 (2.2)[a]	17.1 (3.1)	5.1 (0.8)	5.1 (1.0)	14.5 (0.9)	2.3 (0.5)
**Video/Child 2**	**Field workers** (n=10)	19.4 (1.7)	31.4 (2.7)	3.2 (0.4)	4.0[§]	12.7 (1.3)	3.8 (0.4)
	**Healthcare professionals** (n=13)	19.5 (1.4)	30.9 (3.1)	5.5 (2.6)	4.5 (1.0)	12.5 (1.3)	3.9 (0.5)
**Video/Child 3**	**Field workers** (n=10)	21.9 (2.0)	25.0 (2.9)	4.0 (0.9)	7.6 (0.7)	12.4 (1.3)	3.3 (0.5)
	**Healthcare professionals** (n=13)	22.6 (1.9)	26.8 (5.3)	3.9 (0.8)	5.7 (1.4)	11.0 (1.6)	3.9 (0.7)

[a]t=-3.2, p<0.001. [§]As score for this group is identical for all test subjects within this group no SD has been reported

For peer review only

S7 The INTERGROWTH-21st Neurodevelopment Assessment (INTER-NDA) scoring system and interpretation of domain scores.

**INTER-NDA domain**	Number of items contributing to domain	Constituent item numbers	Method of domain estimation	Interpretation of score
Cognitive	13	1,2,4,5,6,7,8, 11,12,13,14,16,18	Mean of constituent item scores	Higher score reflects better performance
Fine motor	4	9,10,15,20	Mean of constituent item scores	Higher score reflects better performance
Gross motor	3	19,21,22	Mean of constituent item scores	Higher score reflects better performance
Language	12	3,5,8,17,23,24,25,26,27,28,29,30	Mean of constituent item scores	Higher score reflects better performance
Positive behaviour	5	31,32,33,34,35	Mean of constituent item scores	Higher score reflects better performance
Negative behaviour	2	36,37	Mean of constituent item scores	Lower score reflects better performance

For peer review only

S8 Formulae and tables for the conversion of raw scores to standardised (scaled) scores (range 0-100)

Domain	Min - Max	Scaling formula
Cognitive, fine motor, gross motor and language	1 – 4	((x – 1) / 3)) * 100
Positive and negative behaviour	1 – 3	((x – 1) / 2)) * 100

Domain conversion table (selected values) for cognitive, motor (fine and gross), and language domains	
**Raw mean score**	**Scaled mean score**
1.00	0.0
1.25	8.3
1.50	16.7
1.75	25.0
2.00	33.3
2.25	41.7
2.50	50.0
2.75	58.3
3.00	66.7
3.25	75.0
3.50	83.3
3.75	91.7
4.00	100.0

For peer review only

Domain conversion table (selected values) for positive and negative	
**Raw mean score**	**Scaled mean score**
1.0	0.0
1.2	10.0
1.4	20.0
1.6	30.0
1.8	40.0

2.0	50.0
2.2	60.0
2.4	70.0
2.6	80.0
2.8	90.0
3.0	100.0

For peer review only

Supplementary Information S9 INTER-NDA domain scores centiles including and excluding children scoring above the CBCL threshold for clinical (97th centile) problems.

INTER-NDA domain	Pooled Centiles for children including children scoring above the 97th CBCL centiles for attentional problems and/or emotional reactivity (n=1209)							Pooled Centiles for children excluding children scoring above the 97th CBCL centiles for attentional problems and/or emotional reactivity (n=1181)						
	**c3**	**c10**	**c25**	**c50**	**c75**	**c90**	**c97**	**c3**	**c10**	**c25**	**c50**	**c75**	**c90**	**c97**
**Cognitive**[1]	27.3	38.5	60.9	79.2	88.2	92.6	99.6	27.4	38.5	62.2	79.5	88.8	92.6	99.6
**Fine motor**[1]	17.4	25.2	71.5	90.4	100.0	100.0	100.0	17.5	25.7	74.2	91.4	100.0	100.0	100.0
**Gross motor**[1]	31.9	51.6	66.7	81.5	100.0	100.0	100.0	31.1	51.7	66.7	81.6	100.0	100.0	100.0
**Language**[1]	12.1	17.0	44.6	70.7	88.0	94.9	100.0	12.1	17.8	45.7	71.7	88.5	95.1	100.0
**Positive behaviour**[1]	32.8	50.3	69.7	90.0	100.0	100.0	100.0	37.8	51.4	70.0	90.0	100.0	100.0	100.0
**Negative behaviour**[2]	0.0	0.0	0.0	25.0	25.1	50.1	83.0	0.0	0.0	0.0	25.0	25.0	50.0	76.5

INTER-NDA: The INTERGROWTH-21st Neurodevelopment Assessment
[1]For these domains, higher scores reflect better outcomes
[2]For negative behaviour, lower scores reflect better outcomes

For peer review only

S10 Comparison of INTERGROWTH-21st Neurodevelopment Assessment (INTER-NDA) domain scores between two year-old girls and boys

	Centiles for girls (n=617)							Centiles for boys (n=564)							Girls (n=617)	Boys (n=564)	
**INTER-NDA Domain**	**c3**	**c10**	**c25**	**c50**	**c75**	**c90**	**c97**	**c3**	**c10**	**c25**	**c50**	**c75**	**c90**	**c97**	**Median (IQR)**	**Median (IQR)**	**p-value***
**Cognitive**[1]	27.2	38.9	64.3	81.7	89.7	93.0	99.5	27.0	38.2	58.3	76.9	86.8	92.6	99.2	82.1 (64.1, 89.7)	76.9 (59.0, 87.2)	0.001
**Fine motor**[1]	18.7	28.3	74.6	91.6	100.0	100.0	100.0	17.6	25.3	68.1	86.0	100.0	100.0	100.0	91.7 (75.0, 100.0)	83.3 (66.7, 100.0)	0.062
**Gross motor**[1]	27.6	52.7	66.7	83.1	100.0	100.0	100.0	32.4	52.5	66.7	79.6	100.0	100.0	100.0	83.3 (66.7, 100.0)	77.8 (66.7, 100.0)	0.318
**Language**[1]	13.9	22.1	49.5	75.7	90.1	97.1	100.0	11.6	16.8	40.0	65.6	85.2	93.9	100.0	75.8 (50.0, 90.0)	66.7 (38.9, 86.1)	<0.001
**Positive behaviour**[1]	34.1	51.7	70.4	90.0	100.0	100.0	100.0	39.5	52.4	68.2	90.0	100.0	100.0	100.0	90.0 (70.0, 100.0)	90.0 (70.0, 100.0)	0.100
**Negative behaviour**[2]	0.0	0.0	0.0	16.1	25.0	50.0	75.6	0.0	0.0	0.0	25.0	29.4	50.6	83.6	25.0 (0.0, 25.0)	25.0 (0.0, 25.0)	<0.001

INTER-NDA: The INTERGROWTH-21st Neurodevelopment Assessment
*p value from Wilcoxon rank-sum test
[1]For these domains, higher scores reflect better outcomes
[2]For negative behaviour, lower scores reflect better outcomes
IQR: inter-quartile range

For peer review only

The International Fetal and Newborn Growth Standards for the 21st Century

INTERGROWTH-21st

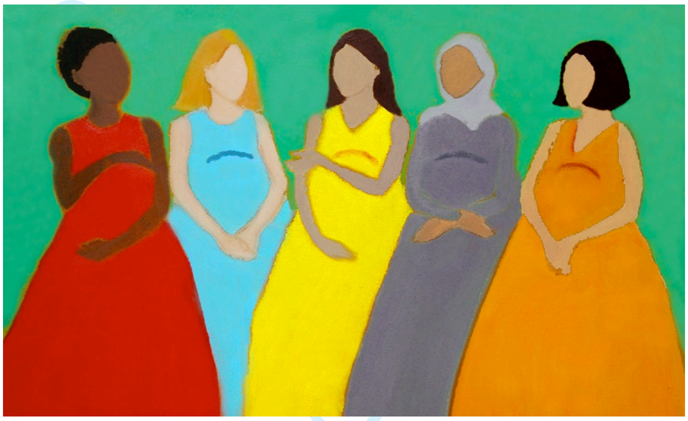

Study Protocol

International Fetal and Newborn Growth Consortium

The Aga Khan University Hospital, Kenya; **Aga Khan University Medical Centre**, Pakistan; **Beijing Obstetrics & Gynaecology Hospital,** China; **Ketkar Nursing Home**, India; **Universidade Federal de Pelotas**, Brazil; **Ministry of Health**, Sultanate of Oman; **University of Oxford**, UK; **Department of Nutrition for Health & Development, WHO**, Switzerland; **Università di Torino**, Italy, **University of Washington**, USA

UNIVERSITY OF OXFORD

v.6 November 2012

For peer review only

Nuffield Department of Obstetrics & Gynaecology, University of Oxford, UK (Professor José Villar; Professor Stephen Kennedy; Dr Aris Papageorghiou);

Universidade Federal de Pelotas, Pelotas, Brazil (Professor Fernando Barros);

Ketkar Nursing Home, Nagpur, India (Dr. Manorama Purwar);

Department of Paediatrics & Child Health, Aga Khan University Medical Centre, Karachi, Pakistan (Professor Zulfiqar Bhutta);

Beijing Obstetrics & Gynaecology Hospital, Maternal and Child Health Center, Capital Medical University, Beijing, China (Professor Pang Ruyan);

Ministry of Health, Muscat, Sultanate of Oman (Dr. Yasmin Jaffer);

The Aga Khan University Hospital, Nairobi; Kenya (Dr. Maria Carvalho);

Department of Obstetrics & Gynaecology, University of Washington, USA (Professor Mike Gravett)

Department of Paediatrics, Università di Torino, Turin, Italy (Professor Enrico Bertino)

For peer review only

This protocol should be referenced as: International Fetal and Newborn Growth Consortium. The International Fetal and Newborn Growth Standards for the 21st Century (INTERGROWTH-21st) Study Protocol, v.6 2012, www.intergrowth21.org.uk

Acknowledgements

This study is supported by a generous grant from the Bill & Melinda Gates Foundation to the University of Oxford, for which we are very grateful.

We would also like to thank the Health Authorities in Brazil, China, India, Italy, Kenya, Oman, the USA and the University of Oxford, whose funding will facilitate the participation of these study sites as collaborating centres.

For peer review only

Table of Contents

I. Brief Summary 5

II. Governance of Study 6

III. Credits 8

IV. List of Abbreviations Used 9

V. Executive Summary 10

VI. Goals and Objectives 13

VII. Project Design and Implementation 14

VIII. Monitoring, Evaluation, and Dissemination 39

IX. Optimizing Public Health Outcomes and Intellectual Property Plans to Achieve Global Access 40

X. Organizational Capacity and Management Plan 42

XI. Citations 44

XII. Appendices 46

Appendix A: Data Collection Forms 46

Appendix B: Data Flow Models 47

Appendix C: Ultrasound Methods And Quality Control 50

Appendix D: Newborn, Preterm and Maternal Anthropometric Techniques, Equipment and Standardization and Data Quality Assurance and Control Plan. 66

Appendix E: Data Management: the Electronic System 67

Appendix F: Policy and determination of responsibilities for INTERGROWTH-21st 68

Appendix G: Management Structure 73

Appendix H: Selection of Study Sites Flow 74

Appendix I: The Pregnancy Physiology Pattern Prediction (4P) Study: Details of observation measurement 76

Appendix J: The PEA POD Manual of Operations 77

Appendix K: The INTERBIO-21st Study Protocol 89

For peer review only

I. Brief Summary

Charitable Purpose:

To develop new "prescriptive" standards describing normal fetal growth and newborn nutritional status in eight geographically diverse populations, and to relate these standards to neonatal health risk. The worldwide use of these tools should improve infants' health and nutritional status.

Project Description:

The project aims to develop scientifically robust clinical tools to assess fetal growth and the nutritional status of newborn infants, as adjuncts to the recently produced WHO charts for children aged 0 to 5. These will be incorporated into national and international maternal and neonatal programs, and be used to monitor and evaluate maternal wellbeing, infant health and nutrition at a population level.

To achieve these objectives, primary data will be collected on a population-based sample of healthy pregnant women. The tools will describe how fetuses and newborns *should* grow in *all* countries rather than the more limited objective of past growth references which describe how they have grown at specific times and locations. They will allow for evidence-based evaluation of nutritional status at birth and measurement of the impact of preventive and treatment interventions in the community.

For peer review only

II. Governance of Study

Steering Committee:
- Zulfiqar Bhutta - Chair (Husein Laljee Dewraj Professor and Chairman, Department of Paediatrics and Child Health, Aga Khan University Medical Centre, Karachi, Pakistan)
- José Villar (Professor of Perinatal Medicine, Nuffield Department of Obstetrics & Gynaecology, University of Oxford) Principal investigator
- Stephen Kennedy (Head of Department & Professor of Reproductive Medicine/Honorary Consultant, Nuffield Department of Obstetrics & Gynaecology, University of Oxford)
- Aris Papageorghiou (Consultant/Honorary Senior Lecturer in Obstetrics and Fetal Medicine, St George's, University of London and Senior Fellow in Fetal Medicine, Nuffield Department of Obstetrics & Gynaecology, University of Oxford)
- Alison Noble (Professor of Engineering Science, Institute of Biomedical Engineering, University of Oxford)
- Doug Altman (Director, Centre for Statistics in Medicine, University of Oxford)
- Cesar G. Victora (Professor of Epidemiology, Federal University of Pelotas, Brazil)
- Cameron Chumlea (Fels Professor Departments of Community Health & Pediatrics, Wright State University School of Medicine, Ohio, USA)
- Juan A Rivera (Director of the Centre for Research in Nutrition and Health, The National Institute of Public Health, Professor of Nutrition in the School of Public Health in Mexico)
- Fiona Burton (Counsellor and Lay Member, Oxford)

Executive Committee:
- Doug Altman
- Zulfiqar Bhutta
- Alison Noble
- Aris Papageorghiou
- Stephen Kennedy
- José Villar

Principal Investigators
- Fernando Barros (Brazil)
- Pang Ruyan (China)
- Alejandro Velasco (Cuba)
- Manorama Purwar/Mangala Ketkar (India)
- Tulia Todros/Enrico Bertino (Italy)
- Maria Cavarlho/William Stones (Kenya)
- Ali Jaffar Mohammed/Dr Yasmin Jaffar (Oman)
- Stephen Kennedy (UK)
- Manjiri Dighe/Michael Gravett (USA)

Scientific Advisory Committee
- M K Bhan (Secretary, Dept of Biotechnology, Government of India, New Delhi, India)
- Cutberto Garza (Provost and Dean of Faculties, Boston College, USA)
- Shahida Zaidi (Professor of Obstetrics & Gynaecology, Vice President of International Federation of Gynaecology & Obstetrics, Geneva)
- Ana Langer (Maternal Health Task Force at the Harvard School of Public Health)
- Michael Katz (ex-March of Dimes Senior Vice President for Research and Global Programs, Carpentier Professor Emeritus of Pediatrics and Professor Emeritus of Public Health, Columbia University, New York, USA).
- Peter Rothwell (Division of Clinical Neurology, University of Oxford)

For peer review only

Coordinating Unit
- José Villar (Principal Investigator)
- Stephen Kennedy (Study Director)
- Leila Cheikh Ismail (Project Leader)
- Ann Lambert (Project Administrator)
- Melissa Shorten (Project Secretary)

Ultrasound Group
- Aris Papageorghiou
- Shaida Zaidi
- Laurant Salomon
- Ippokratis Sarris/Cristos Ioannou (UK)
- Adriane Mitidieri (Brazil)
- Sunita Sohoni (India)
- Zhang Yong/Wu Qingqing (China)
- Megha Venkatraman/Waleeda Al-Zadjali (Oman)
- Jyoti Shah (Kenya)
- Manjire Dighe (USA)
- Manuela Oberto (Italy)

Anthropometry Group
- Leila Cheikh Ismail
- Wm. Cameron Chumlea
- Adelheid Onyango
- Zulfiqar Bhutta
- Manuel Sanchez Luna
- Susan Lloyd (UK)
- Claudia Rossi (Italy)
- Fatima Al Rahbi (Oman)
- Lui Hong/Shen Yingjie (China)
- Naina Kunnawar (India)
- Manjiri Dighe (USA)
- Denise Mota (Brazil)
- Roseline Ochieng (Kenya)

Neonatal Group
- Zulfiqar Bhutta
- Ricardo Uauy
- Enrico Bertino (Italy)
- Elaine Albernaz (Brazil)
- Kenny McCormick (UK)
- Bashir Bhat (Oman)
- Roseline Ochieng (Kenya)
- Vikram Rajan (India)
- Maneesh Batra (USA)
- Pang Ruyan (China)

Data Management and Analysis Group
- Doug Altman
- Laima Juodvirsiene
- Fenella Roseman (UK)
- Marlos Domingues (Brazil)
- Naina Kunnawar (India)
- Paolo Gilli (Italy)
- Wang Jun Hua/ Wu Minghui (China)
- Hamood Al-Jabri (Oman)
- Norah Musee (Kenya)
- Sarah Waller (USA)

For peer review only

III. Credits

This protocol was prepared by José Villar, Stephen Kennedy, Paul Chamberlain, Douglas Altman and Alison Noble (University of Oxford) and Mercedes de Onis (WHO Nutrition). These authors produced a first version, between October 2006 and May 2007, based conceptually on the published WHO Multicentre Growth Reference Study (MGRS) protocol. The following people provided comments that were considered for inclusion in the initial version: Daniel Giordano, Mario Merialdi, Guillermo Carroli, as well as several participants at a WHO meeting held in June 2007 where this first version was presented by José Villar, the Principal Investigator. We thank them for their contributions. José Villar had discussions with Daniel Giordano about data collection forms and models for data management, based on available documents and previous studies; some components have been retained in the present study and his input is much appreciated.

Further important contributions were made by Cesar Victora, Fernando Barros, Michael Kramer and Robert Platt to expanded versions of the initial document that eventually constituted the grant application to the Gates Foundation. They also made important contributions to the responses provided by the applicants to the reviewers' comments. The reviewers and staff at the Gates Foundation made considerable contributions and raised a number of important issues, all of which have been incorporated into this version. We are very grateful to all the contributors and in particular to Dr Ellen Piwoz. Finally, members of the study's Steering Committee and potential investigators contributed during the preparatory phase of the study to the final version of this protocol.

During 2008 until February 2009, Jan van den Broeck made contributions to the anthropometric, ultrasound and anthropometric quality control strategies of the study design after the original protocol was approved by the Gates Foundation. Where these suggestions have been retained, appropriate credits are provided in the text.

Overall, this protocol is intended to extend the concept of the MGRS protocol to cover most aspects of growth during fetal and newborn life. Therefore, this document has been developed to comply as much as possible with the WHO MGRS' rationale and procedures. We thank all the investigators and the members of the WHO MGRS Coordinating Unit for allowing us to consult their study material and to use an electronic version of their document during the early preparation of our protocol.

All the individuals mentioned above, have been invited to join the Study team at various stages and in different capacities. We still welcome contributions from those few who have not yet joined us.

For peer review only

DISCLAIMER: WHO has not formally reviewed the INTERGROWTH-21st study protocol. Therefore, the technical advice being provided to INTERGROWTH-21st by WHO staff does not constitute endorsement of, or support for, the research project by WHO.

IV. List of Abbreviations Used

**AC**	Advisory Committee
**AC**	Abdominal Circumference
**ADQU**	Anthropometric Quality Control Unit
**APAD**	Antero-posterior abdominal diameter
**BMI**	Body Mass Index
**BPD**	Biparietal Diameter
**CRL**	Crown Rump Length
**CU**	Coordinating Unit
**DMU**	Data Management Unit
**EC**	Executive Committee
**FGLS**	Fetal Growth Longitudinal Study
**FL**	Femur Length
**IDAMIS**	INTERGROWTH-21st Electronic Data Management System
**IUGR**	Intrauterine Growth Restriction
**HC**	Head Circumference
**LBW**	Low Birth Weight
**LMP**	Last Menstrual Period
**MGRS**	WHO Multicentre Growth Reference Study
**NCSS**	Newborn Cross-sectional Study
**NDOG**	Nuffield Department of Obstetrics and Gynaecology
**NICU**	Neonatal Intensive Care Unit
**OFD**	Occipito-Frontal Diameter
**OMPHI**	Oxford Maternal and Perinatal Health Institute
**PPFS**	Preterm Postnatal Follow-up Study
**PRC**	Publications Review Committee
**SC**	Steering Committee
**SLE**	Systemic Lupus Erythematosus
**TAD**	Transverse abdominal diameter

For peer review only

V. Executive Summary

At least 60% of the 4 million neonatal deaths that occur worldwide every year are associated with low birth weight (LBW), caused by intrauterine growth restriction (IUGR), preterm delivery, and genetic/chromosomal abnormalities (1), demonstrating that under-nutrition is already a leading health problem at birth. Accurate assessment of fetal growth and gestational age for timely identification and management of growth restriction, risk assessment of undernourished newborns, and monitoring LBW trends are therefore public health priorities, especially in developing countries where 98% of all neonatal deaths occur. The long-term health implications of these conditions are now well recognized for both developed and developing countries.

Traditionally, fetal nutritional status has been assessed by measurement of uterine height in pregnancy, which can be used in first level screening but needs more precision for suspected IUGR. Birth weight, as the final point of intrauterine growth, is also used but does not adequately describe the fetal growth process. Furthermore, the newborn standards presently recommended by WHO are based on a population of births from California, USA, in the 1970s and are unlikely to be a suitable international reference in the 21st century (2). This is a particular problem for preterm babies, that have different growth patterns to fetuses still *in utero*, and for whom there are no high quality postnatal growth standards, i.e. using the prescriptive approach of the new WHO infant and child growth standards (3).

In the developed world and in urban areas in most developing countries, especially if complications are suspected, fetal growth is assessed by comparing ultrasound measurements of fetal size with reference percentiles obtained from fetuses whose growth was assumed to be normal. Unfortunately, most of the ultrasound charts of size by gestational age were obtained from very small populations of fetuses in the USA or Europe, and may not be appropriate for use internationally.

These limitations have generated concern regarding the value of ultrasound in routine clinical care particularly late in pregnancy, as well as when newborn standards are used to determine the health status of populations or monitor trends and progress in reducing under-nutrition. For example, the WHO Expert Committee on "Physical Status: the use and interpretation of anthropometry" (1995) recommended the development of international standards for both fetal and newborn growth, as well as child growth (4). The latter were recently produced by WHO and are being implemented worldwide; the former are still in the research priority agenda (5). We now propose in this project to extend the WHO work by constructing a new set of international **Fetal and Newborn Growth Standards**. It will be the first, population based, large, multi-ethnic, longitudinal, fetal growth standard based on early assessment of gestational age.

Goals and Objectives

The goal is to develop new "prescriptive" standards describing normal fetal and preterm neonatal growth over time and newborn nutritional status, and to relate these to neonatal health risk.

The primary objective is to produce a set of international **Fetal and Newborn Growth Standards** (fetal growth, birth weight for gestational age and postnatal growth of preterm infants) for practical applications in clinical use and for monitoring trends in populations. We will relate the new newborn standards to neonatal morbidity and mortality to identify levels of perinatal risk.

The secondary objectives are:

a) **Clinical:** to develop a prediction model, based on multiple 2-dimensional (2D) ultrasound measurements, for estimating gestational age during mid-late pregnancy for use in populations of pregnant women without access to early/frequent antenatal care;
b) **Epidemiological:** to investigate in this multi-ethnic, population based sample the determinants of LBW and its components (preterm delivery, impaired fetal growth) under current healthcare conditions, and

For peer review only - http://bmjopen.bmj.com/site/about/guidelines.xhtml

c) **Biological:** to acquire additional 3-dimensional (3D) images to create an anatomical and growth databank of individual fetal organs as a unique source of biological information for future research.

Project Design and Implementation

The primary objective has 3 components (Activities 1-3):

1. **Fetal Growth Longitudinal Study (FGLS)** from <14^{+0} weeks gestational age to birth to monitor and measure fetal growth clinically (Symphyseal-fundal-height) and by ultrasound in a healthy population.
2. **Preterm Postnatal Follow-up Study (PPFS)** of preterm infants (>26^{+0} but <37^{+0} weeks) in the FGLS to describe their postnatal growth pattern.
3. **Newborn Cross-sectional Study (NCSS)** of all newborns at the study centres over 12 months, obtaining anthropometric measures and neonatal morbidity and mortality rates.

Eight geographically diverse populations will participate, covering North and South America, Europe, Africa, Western Asia and the Indian Subcontinent. Each will contribute a population-based sample of healthy pregnant women expected to provide full growth potential to their fetuses, making it possible to produce prescriptive standards, similar to the WHO Child Growth Standards. Participating sites will contribute to all the components of the project.

The study populations obtained from these geographically defined areas (e.g. city or county) should have no socio-economic constraints on growth, low morbidity and perinatal mortality and adequate nutritional status. To be included, women should be non-smokers, with a normal pregnancy history, and without health problems likely to influence fetal growth or indicate a risk for pregnancy-related pathological conditions. In **FGLS**, women will be screened before 14^{+0} weeks at the time of their first antenatal visit and followed-up with standard clinical and 2D ultrasound examinations every five weeks, i.e. up to six times during pregnancy. In **PPFS**, preterm infants (>26^{+0} but <37^{+0} weeks) born from this sample will be followed-up during the first 8 months of life with the same protocol and set of anthropometric measures that were used in the WHO Child Growth Study. Postnatal growth will be evaluated from both delivery and conception for comparison with the corresponding *in utero* measurements. In the **NCSS** study, all newborns at the study centres, born during a fixed (e.g. 12 month) period, will have anthropometric measurements taken immediately after birth. Only babies born to women who meet the same inclusion criteria used in FGLS will be selected to construct the newborn standards. Birth weight and gestational age will also be related to neonatal morbidity and mortality outcomes to construct risk-related newborn weight for gestational age standards. Approximately 5,000 pregnancies will be included in FGLS allowing for ~ 350 preterm infants to be included in PPFS. We expect ~500 women will have to be recruited at each study site to achieve the required number of participants. In NCSS, ~50,000 newborns will be recruited, of whom we expect close to 75% will be eligible for the standards yielding ~1,800 cases with severe newborn outcomes.

Standard quality control measures will be included, as well as a unique system of random evaluation and repetition of ultrasound measurements (from stored images) to monitor validity and reliability, and continuous real time assessment of all data collected. A limited number of experienced obstetric ultrasonographers and neonatal anthropometrists will form the research teams at each study site. Neonatal anthropometric measures will be monitored and standardized centrally. All data will be entered and managed in an on-line data management system specifically developed for the study, including a system for direct transfer of blinded data from the ultrasound equipment to the database. This on-line system allows the initiation of data analysis soon after data collection is completed. Results from all populations will be pooled (if biologically relevant differences are not observed between sites) to construct the curves for international applications using the same analytical strategy as in the WHO Child Growth Standards. We expect that extensive secondary analyses will be conducted from these data that will make major contributions to science and clinical practice in the near future (Objectives II and III) and for many years to come. This study involves minimal risk to participants, no greater than at routine antenatal or clinical

For peer review only - http://bmjopen.bmj.com/site/about/guidelines.xhtml

examinations. Ethical approval will be sought at the appropriate levels, informed consent obtained and confidentiality guaranteed.

Monitoring, Evaluation and Dissemination

The project will run from July 2008 to December 2014. The project's implementation will be monitored following standardized practices implemented in previous large multi-centre studies by WHO and the Perinatal Research Network, according to the set of milestones described in the proposal. It is expected that by 2014: a) The newly developed fetal growth standards will be incorporated into all obstetric ultrasound equipment produced worldwide; b) Preterm infants' growth will be evaluated using the new postnatal growth curve, and c) Newborns will be assessed using the new, international birth weight for gestational age standards. Intermediate milestones are: a) Start recruitment by April 2009; b) Complete fetal sample by March 2011; c) Complete follow-up by February 2012; d) Have data set ready for analysis by July of 2012, and e) Present final standards by the end of 2013. The dissemination of the fetal, postnatal and newborn growth standards will utilize the same channels as the roll-out of the WHO Child Growth Standards and will be coordinated by the Maternal Health Task Force at the Harvard School of Public Health.

Optimizing Public Health Outcomes

The new fetal and newborn standards when incorporated into health services will be unique tools for evidence-based estimation of under-nutrition at birth, maternal and perinatal programme monitoring and the clinical care of newborns worldwide, specifically in developing countries where most of these health problem exist. When fully implemented, the growth charts we propose to develop should benefit all pregnancies throughout the world because fetal growth is currently evaluated using clearly limited tools. This should provide major clinical and economic benefits. The preterm and newborn charts should be of greatest benefit in developing countries where most morbidity and mortality amongst newborns (especially growth restricted and preterm babies) occur.

For peer review only

VI. Goals and Objectives

A. Goals and Attributable Benefits

The **goal** is to develop new "prescriptive" standards describing normal fetal and preterm neonatal growth and newborn nutritional status, and relate these to perinatal health risk.

The anticipated **attributable benefits** of this project are that by 2014:

a) The newly developed fetal growth standards will be incorporated into all obstetric ultrasound equipment produced worldwide.
b) Preterm infants' growth will be evaluated using the new postnatal growth curves
c) All newborns worldwide will be assessed using the new, risk related, international birth weight for gestational age standards.

B. Objectives

Primary objective:

I. To produce a set of international **Fetal and Newborn Growth Standards** (fetal growth, birth weight for gestational age and postnatal growth of preterm infants) for practical applications in clinical use and for monitoring trends in populations. The newborn standards will then be related to perinatal morbidity and mortality to identify levels of risk and target interventions.

Secondary objectives:

II. To develop a prediction model, based on multiple 2D ultrasound measurements, for estimating gestational age during mid-late pregnancy for use in populations of pregnant women without access to early/frequent antenatal care.
III. To determine in this multi-ethnic sample the determinants of LBW, as well as associated conditions, e.g. preterm delivery and impaired fetal growth and their subgroups, under current healthcare conditions.
IV. To acquire additional 3D images to create an anatomical and growth databank of individual fetal organs as a unique source of biological information for future research.

To the best of our knowledge, this project is unique because of its comprehensiveness and scale (never attempted before). It has all the characteristics recommended in the literature for constructing ideal standards for international use (6, 7): it is prescriptive, longitudinal, population-based, and gestational age will be established by ultrasound early in pregnancy. We will be able to explore associated research questions, such as possible ethnic differences in fetal/newborn growth, risk factors for LBW and its subgroups. We will also be able to predict gestational age in women with poor antenatal care and simultaneously provide a set of practical tools, which can be introduced into maternal and newborn care worldwide in a short space of time.

There is considerable demand from maternal and neonatal services to WHO and professional organizations for the standards that this study will produce (5). Hence, we have designed this project in consultation with experts in the field, as well as other research groups and institutions, in such a way that it could easily complement other yet to be developed studies, thereby reducing the risk for duplication.

For peer review only

VII. Project Design and Implementation

Background

In April 2006, WHO released the WHO Child Growth Standards for children aged 0 and 5 which were generated by the WHO Multicentre Growth Reference Study (MGRS) (8). Two characteristics made the MGRS unique and unprecedented as a study in its field: 1) The study included populations from several countries (Brazil, Ghana, India, Norway, Oman and the USA) and 2) A prescriptive approach was used to select the study populations, i.e. only children from populations with minimal environmental constraints on growth were included. This was achieved by recruiting children of affluent and educated parents, because high education and family income have been identified as the environmental variables most likely to be associated with optimal child growth. In addition, chronic illness, failure to adhere to MGRS feeding recommendations and maternal smoking were used as exclusion criteria.

By virtue of these characteristics, the MGRS provided the strong scientific foundations for developing standards that indicate how children should grow, as opposed to previous studies that simply described actual patterns of growth at a particular time and place. Consequently, the WHO Child Growth Standards (8) are now being used worldwide to judge children's growth because they demonstrate how healthy children grow in an environment which allows them to achieve their full growth potential.

The proposed study extends the MGRS into fetal and neonatal life. It will be based on the same prescriptive approach and international representation will be ensured by including populations from several countries. The design, implementation and conduct of the study, and dissemination of the results, as well as their incorporation into clinical practice guidelines and health care policy, will build on what has been achieved with the WHO Child Growth Standards, ensuring continuity between the development and implementation at country level of pre- and postnatal growth standards.

Conceptual Issues

There are important conceptual differences between growth references and standards. References, traditionally regarded as descriptive, are used for comparing different populations, while standards are prescriptive, implying a value judgment of optimal growth to be followed by individual pregnancies. A number of developed, as well as some developing countries have their own national standards for obstetric and neonatal use. In general, it is accepted that there is some variation in growth patterns among children of different racial or ethnic groups; however, these variations are relatively small compared to the large, worldwide variation in growth related to health, nutrition and socio-economic status. Therefore, the WHO Expert Committee on Physical Status (4) argued that "for this reason, a common reference has the advantage of uniform application allowing international comparisons without losing the usefulness for local application". Such advantages outweigh the disadvantage of not taking into account racial and ethnic variations, if actually observed. There are also practical reasons for not developing local growth standards for each country: 1) Many populations in less developed areas experience growth deficits as a result of poor health and nutrition, so that a local reference will have less screening value for the detection of health and growth disorders; 2) Significant secular changes in growth status within a relatively short period of time may render local standards less useful for clinical screening, and 3) Proper reference development is not a task that can be done easily or repeatedly, and it is very expensive to develop local standards.

In addition, as this is a longitudinal study, we will be able to study both fetal size over time (most commonly referred to as growth charts, i.e. centiles of *size* conditional on age) and actual fetal growth, expressed by the rate of growth between time periods (velocity charts).

For peer review only - http://bmjopen.bmj.com/site/about/guidelines.xhtml

Rationale for International Population Based Standards

We believe that the current use of multiple local charts presents sufficient problems to warrant preparing simple, international standards. However, measuring fetuses and newborns from a single, ethnically homogeneous community would be inappropriate, as it would fail to reflect any worldwide variation in growth. Using multiple populations from several countries would enhance the diversity in the biological characteristics - such as parental size and maternal weight gain during pregnancy - as well as the socio-cultural factors influencing fetal growth. This approach would also be politically more acceptable than constructing the standards based on data obtained from a single country.

Furthermore, there have been suggestions that the so called "customized" birth weight standards including ultrasound-derived curves of intrauterine weight (based on estimated fetal weight) are better predictors of perinatal mortality than population-based birth weight standards similar to those we plan to produce in the newborn component of the study. We do not agree with this concept as the maternal variables included in "customized" charts, such as ethnicity, parity and maternal weight are not physiological characteristics, they are intermediate variables. There is now strong evidence, using perinatal mortality as the substantive outcome, that the benefit in prediction is not a consequence of the "customization" by maternal characteristics but rather an effect of the higher birth weight distribution obtained from ultrasound derived fetal weight charts. Therefore, these adjusted charts will identify more preterm infants as growth restricted and therefore predict mortality better because of a higher cut-off and not because they fit fetal growth patterns better (9). The proposed population-based newborn standards from a well nourished, healthy population should produce results similar to those obtained by the "customized" charts, and in addition will be related to perinatal morbidity and mortality in the same study population.

Introducing the New Standards to Pregnancy and Newborn Care

Antenatal care consists mostly of a series of screening tests of varied complexity, implemented at different levels of care that, as a group, contribute to evaluating the overall status of each pregnancy. Objective assessments of fetal growth deviations can play a major role in prenatal care, neonatal care and outcome-based research. We are not proposing to recommend routine serial ultrasound examinations for all pregnant women in either developed or developing countries (see (10) and NICE Recommendations 2008); rather that, when they have to be used because of a clinical condition, they are interpreted correctly. Our study will produce three, new, scientifically valid tools for use at different levels of care to complement other tools already in use. The new standards will facilitate the correct interpretation of ultrasound scanning at levels of care where it is already widely used e.g. referral and most urban hospitals in developing countries. It is anticipated therefore that the new charts will have a major impact on overall care: for example, they are likely to result in fewer unnecessary interventions, such as Caesarean sections. The newborn standards will be used at all levels of delivery including rural areas; the preterm standards will be used in NICUs and similar levels up to primary health care. As an additional contribution to primary health care, we will produce new, multi-ethnic, uterine fundal height charts. We believe that such a comprehensive approach is the most effective format of care for pregnancy and newborns in developing countries.

Overall Study Strategy

Our overall strategy has been to adopt a very pragmatic approach to the study. In other words, we have tried to incorporate the study procedures into routine clinical care, so as to inconvenience the mothers as little as possible and avoid disrupting service delivery. An additional advantage of this strategy is that it increases the likelihood that the study's recommendations will be implemented and introduced into real life settings.

Nevertheless, during the preparation and implementation of the study, there will be many instances when a clear-cut answer to a clinical problem will not exist. We plan, therefore, to adopt a policy of asking the Principal Investigators and other members of the Steering Committee to make decisions in such cases. This policy will broaden our knowledge base and create a genuine feeling of team membership, which is so important in carrying out complex, multi-centre studies. The decision-making process will be supported by an

For peer review only - http://bmjopen.bmj.com/site/about/guidelines.xhtml

independent Advisory Group, which will be asked to resolve strategic conflicts. Ultimately, however, responsibility for finalizing the Study Protocol and the Operations Manual rests with the Executive Committee.

Selection of Study Sites and Population Framework:

There are two levels in the selection process of the study population: a) the cluster level, i.e. geographical region (e.g. city or part of a city) and within this geographical area, the selection of health institutions where women attend for antenatal and delivery care and b) the individual level, i.e. women or newborns that have the characteristics to be eligible for each of the sub- studies. We will discuss here the latter strategy, as it is common to all sub-studies and leave the former for each section on the specific sub-studies.

It is not necessary and would be unrealistic, especially in developing countries, for all institutions in a study area to fulfill all selection criteria. The basic characteristic for an institution to be considered is to serve a low risk population for impaired fetal growth. The concept is that such target populations, from which the individual participants will be drawn, should have no or be at very low risk of health, environmental or socio-economic constrains on fetal and newborn growth. Definitions will be locally adapted, for identifying socioeconomic characteristics associated with unconstrained growth in these populations. In principle, these will include markers of household income, housing tenure, education, occupation and employment status using locally selected cut-off points. These variables have been recently identified as explanatory factors in the relationship between birth weight and ethnicity (11).

Specifically, from each study site we will identify health institutions that serve populations with the above characteristics. We will conduct first a census of all hospitals where deliveries take place that are classified under local definitions as "private" or "corporation" hospitals or serving the upper socio-economic sector of the selected region. This is very important for developing countries sites. In developed countries, potential centres should also serve a general population, which will mostly have low-risk characteristics. We should concentrate on large institutions (>1000 deliveries a year), as it is important to involve only a small number of hospitals. Thus, from this census we will select those hospitals that cover at least 80% of all deliveries in the target population. However, it is recognized that some sites will not have large private hospitals and so local adaptations will have to be made.

The selected hospitals will need to agree to collaborate with the University of Oxford (Study Coordinating Unit) and provide evidence of being able to implement the study protocol; in particular, being able to coordinate referrals for ultrasound scans and having a policy of confirming gestational age by early ultrasound examination in all pregnant women. They will also need to show, using the latest data available, that the population they serve is actually of low risk as defined by: a) LBW rate <10% and mean birth weight >3100g; b) located at an altitude below 1,600m; c) perinatal mortality <20 per 1000 live births; d) mothers attending antenatal care in these institutions should plan to deliver in that or a similar hospital located in the same region; e) >75% of mothers have attained an educational level greater than the locally defined cut-off point; and f) lack of known non-microbiological contamination such as pollution, radiation or any other toxic substances (this will be evaluated in collaboration with the WHO Department of Protection of the Human Environment).

From this pool of eligible institutions that have the potential to provide the required sample size for ALL components of the study (target population), the Executive Committee and its advisors, in collaboration with the local study team, will identify the final list of units that will contribute to all the different sub-studies according to their capacity to provide the sample size needed, logistics, availability of equipment and population distribution. Individual participants will be selected from such a target population following the individual criteria listed below (see Section 1.1). Sampling for the fetal growth study will consist of all women that agree to participate and meet the selection criteria from the given date for initiation of the study until the sample is reached (500 pregnancies). For the preterm study we will take all preterm babies, with the inclusion criteria, from this cohort and for the newborn study we will take all newborns delivered at these institutions during the 12 month study period.

For peer review only - http://bmjopen.bmj.com/site/about/guidelines.xhtml

A. Objective I (Activities 1 – 3)
The primary objective of the international, multi-centre study will be achieved by three Activities:

1. Fetal Growth Longitudinal Study (FGLS) from <14^{+0} weeks gestation to birth, to develop the Fetal Growth Standards;
2. Preterm Postnatal Follow-up Study (PPFS) of preterm infants (>26^{+0} but <37^{+0} weeks) in the FGLS, to develop the Preterm Postnatal Growth Standards;
3. Newborn Cross-Sectional Study (NCSS) of all neonates born over 12 months in the participating centres including anthropometric measures and indicators of perinatal morbidity/mortality to develop the Newborn Birth Weight for Gestational Age Standards.

Both longitudinal and cross-sectional data will be collected at eight geographically diverse study sites, covering North and South America, Europe, Africa, Eastern, Western Asia and the Indian subcontinent, providing subpopulations from 5 major ethnic groups worldwide. The study sites have been selected from a pool of approximately 15 institutions already identified on the basis of a proven record of submitting high-quality data to clinical studies (10, 12-14). They also a) serve populations with a socio-economic status that does not restrict fetal growth (ensuring that the curves reflect the true growth potential of fetuses, without constraints related to maternal nutrition or any other social or environmental factors); b) are located at an altitude below 1,600m, and c) have a perinatal mortality rate <20/1000, a preterm delivery rate less than 15% of the target population.

1. FGLS: to develop the Fetal Growth Standards (Activity 1)
Limitations of existing intrauterine growth charts: There are three important issues relating to the construction of fetal growth standards that, in general, were not considered when the ones presently in use were produced (4). The issues are a) **longitudinal approach**: To create a standard that reflects patterns of healthy fetal growth, serial size measurements at different stages of pregnancy are required. This provides estimates of both the amount of variability in measures among fetuses in a population, as well as the amount of variability within individuals from their own average growth trajectory (15). Some early charts were built using a single measurement from each fetus based on the assumption that cross-sectional measurements from different fetuses can be used to estimate longitudinal patterns of growth. Other authors collected multiple measurements per fetus but analyzed the data in a cross-sectional manner, resulting in an underestimation of the true variance. The present study will collect and analyze data longitudinally. b) **Small sample size**: Most intrauterine standards are based on small sample sizes, which may lead to unstable estimates of percentiles, in particular at the extremes of the distribution. The extreme centiles are those that matter for the identification of growth deviations. c) **Lack of a prescriptive approach**: In the past, references have often been built based on existing practices or from specific research units' their final aim was to have population representation in particular place and time (population representativeness). This is a descriptive approach. We believe this strategy is incorrect as the underlying population often does not possess the best health and/or nutrition. For example, the observed secular increase in birth weight could reflect the tendency towards an increase in maternal body mass index (BMI) and obesity observed in most countries and not necessarily better health status of the pregnant population. Similarly, the very high Caesarean section rates in some populations (12) can affect birth weight patterns as reflected by the increase in preterm deliveries in these populations. Therefore, the proposed new standards will be built on the basis of recommended health practices (i.e. prescriptive approach): that is, defining the target population as well-nourished, healthy (both before and during pregnancy), non-smoking, no extreme ages, adequate education and socio-economic status. The sample to be used for the new standards should be representative of this "prescriptive" population, rather than the general population.

1.1 FGLS Individual Entry Criteria
Introduction: How to select a "healthy population" or its counterpart, the risk factors associated with fetal growth restriction, is open to considerable debate. The most important

For peer review only - http://bmjopen.bmj.com/site/about/guidelines.xhtml

factors to consider when selecting a healthy population with no obvious risk factors for IUGR or over-growth, at the first antenatal care visit early in pregnancy, are: a) achieving a balance between strict criteria for risk and external validity of the study population and b) the logistics of screening for factors that are not part of routine care or for which consensus is lacking about their effect on fetal growth.

We have reviewed the extensive literature on this topic which has tried to separate out the risk factors for preterm delivery and IUGR (16) particularly in low and middle income countries, as well as data from our own large-scale studies which have systematically explored the associations between risk factors and perinatal outcomes. In defining the eligibility criteria for the FGLS, one could exclude women with every possible risk factor for poor pregnancy outcome. However, we believe it would be preferable, in the initial screening process, to identify factors that are proven to be related to pregnancy outcomes, specifically those that are recommended as ways of identifying women who would benefit from low-risk antenatal care.

The approach is based upon our overview of the evidence of the effectiveness of antenatal care (17), which was recently up-dated (Abalos, *WHO* 2008). Similar patterns of care are recommended in the NICE/UK Clinical Guideline (2003) and by WHO for the antenatal booking visit. We will try to ensure that the approach is implemented in the participating centres in a standardized way.

All women attending the first antenatal care visit in the selected institutions with an initial evaluation of gestational age $<14^{+0}$ weeks will be screened at study entry based upon the criteria listed below. These women define a population that is likely to need only routine antenatal care in pregnancy, i.e. it is a group of clinically healthy women who can follow basic antenatal care models. Some variables have clear thresholds (e.g. urinary culture positive or negative); for others with less clear values (e.g. maternal height), we have selected a cut-off of 153 cm from the WHO Global Survey, a very large, global, multi-ethnic Maternal & Perinatal Health Study - for a detailed description of one of the 3 regions studied, see (18). This cut-off is supported by corresponding results from a number of other international studies (19, 20)

For peer review only

Individual Characteristics:

Recruited women must have the following characteristics at booking:

a) aged ≥18 and <35 years;
b) BMI ≥18.5 and <30 kg/m^2;
c) height ≥ 153 cm;
d) singleton pregnancy;
e) a known LMP with regular cycles (defined as 28 days ±4 days) without hormonal contraceptive use, or breastfeeding in the 2 months before pregnancy;
f) natural conception
g) no relevant past medical history (refer to screening form), with no need for long-term medication (including fertility treatment and over-the-counter medicines, but excluding routine iron, folate, calcium, iodine or multivitamin supplements);
h) no evidence of socio-economic constraints likely to impede fetal growth identified using local definitions of social risk;
i) no use of tobacco or recreational drugs such as cannabis in the 3 months before or after becoming pregnant;
j) no heavy alcohol use (defined as > 5 units (50ml pure alcohol) per week) since becoming pregnant;
k) no more than one miscarriage in the 2 previous consecutive pregnancies;
l) no previous baby delivered pre-term (<37 weeks) or with a birth weight <2500g or >4500g;
m) no previous neonatal or fetal death, previous baby with any congenital malformations, and no evidence in present pregnancy of congenital disease or fetal anomaly;
n) no previous pregnancy affected by pre-eclampsia/eclampsia, HELLP syndrome or a related pregnancy-associated condition;
o) no clinically significant atypical red cell alloantibodies;
p) negative urinalysis;
q) systolic blood pressure <140 mmHg and diastolic blood pressure < 90 mmHg;
r) no diagnosis or treatment for anaemia during this pregnancy (Hb levels wil be monitored throughout pregnancy)
s) no clinical evidence of any other sexually transmitted diseases, including syphilis and clinical Trichomoniasis;
t) not in an occupation with risk of exposure to chemicals or toxic substances, or very physically demanding activity to be evaluated by local standards. Also women should not be conducting vigorous or contact sports, as well as scuba diving or similar activities

For peer review only

Estimation of gestational age at study entry: Clearly, establishing a very precise determination of gestational age is vitally important for constructing these growth standards (in addition to clinical management) and we are prepared to screen large numbers of women, if necessary, to obtain the ideal population.

There are three possibilities for gestational age estimation early in pregnancy: dating by a) LMP alone; b) early ultrasound alone or c) LMP and ultrasound. The implications of these different methods on research findings have recently been discussed (22). Dating by LMP and ultrasound is clearly an assessment of different parameters. Although it has been suggested that before 14^{+0} weeks of gestation ultrasound may be better by an average of 2-3 days in predicting the date of delivery, in clinical practice, both are often used in combination. Based on the 2004 Birth Cohort from the Brazilian Centre, we anticipate that 7% of women will not have a reliable LMP. In other studies in this socio-economic group, the figure may be as high as 20%.

Taking all these factors into consideration, we have decided not to base gestational age solely on LMP; rather we shall use a standard gynecological definition of LMP as the first level in calculating the gestational age. Among women with a certain LMP and regular periods who are 9^{+0} to 14^{+0} weeks pregnant, we will corroborate the gestational age with an ultrasound CRL measurement, using an internationally recognized and validated chart (23). If the difference in gestational age estimation by CRL and LMP is ≤ 7 days we will consider the LMP to be reliable and take it as the true biological date. It is acknowledged that we may exclude a number of women who might otherwise have been eligible if only ultrasound were used, but the aim is to define a population with as exact a gestational age at entry as possible.

1.2 Nutritional adequacy during pregnancy

In the WHO Child Growth Study, it was assumed from the outset that children recruited from affluent populations consumed adequate complementary foods. This proved to be the case when we analyzed their complementary diets (24). In the present study, we shall adopt the same concept but in addition will develop general nutritional guidelines for pregnant/lactating women, suitable for local use, based on the best available evidence, e.g. NICE recommended, UK Food Standards Agency advice http://www.eatwell.gov.uk/agesand Stages/pregnancy/whenyrpregnanct/ for promotion amongst the participating mothers and care providers.

Routine nutritional supplements, e.g. protein or energy, will not be given because a) it is not a component of the recommended antenatal care package (10) and b) we are only planning to recruit women with adequate nutritional status. Iron-folate supplementation, will be prescribed if necessary for anemia during/after pregnancy but given routinely only if such a policy is in place in the institution. A similar position will be taken with calcium supplementation for the prevention of pre-eclampsia and preterm delivery if such a policy is in place in the hospital.

Women will be asked to indicate if they are taking nutritional supplements and this information will be recorded in the data collection form. It is not practical in a study of this size to measure adherence in any other way or to obtain individual intakes, e.g. 24 hr recall, considering the poor reliability of such instruments for individual assessment.

1.3 Pregnancy follow-up

Women in FGLS will receive standardized antenatal care (with some local variations) based on the recommended WHO package, part of which involves screening for conditions that emerge during pregnancy.

All women recruited will be followed throughout pregnancy from the time of the first visit, irrespective of the pregnancy outcome. As a general principle, the number of exclusions will be as small as possible. They are likely to be confined to fetuses with congenital abnormalities (based on a final evaluation at birth); multiple pregnancies that were not identified at recruitment; mothers diagnosed with catastrophic or very severe medical conditions which were not present at recruitment (e.g. cancer, HIV), and those with pregnancy related conditions requiring hospital admission (e.g. eclampsia or severe pre-eclampsia). Hospital admission *per se* is not a reason for exclusion: women admitted simply for "observation" will still contribute data to the fetal growth standards unless they develop one of the conditions listed above. Most sites are malaria free ecozones; thus, intermittent preventive treatment is not routinely recommended although insecticide-treated mosquito nets may be used. In other potential sites malaria is prevalent. We will adhere to local protocols and exclude from incorporation in the fetal growth standards any woman with evidence of malaria infection during the pregnancy.

For the sake of clarity, we will produce during the preparatory phase of the study, in collaboration with the Steering Committee and experts in the field, a very detailed list of those conditions, which would result in a pregnancy being excluded from the preparation of the fetal growth standards. In any case, if any exclusion is needed it will be done only during the data analysis period and local investigators will complete the follow-up of all enrolled women irrespective or any follow-up experience. Data from preterm deliveries, even in the absence of one of the exclusion criteria, will not contribute to the fetal growth standards.

For peer review only - http://bmjopen.bmj.com/site/about/guidelines.xhtml

We also expect that INTERGROWTH-21st will have fewer follow-up problems than randomized trials as it does not involve an intervention; a well-educated population will be recruited, and women and their families will like some components of the study such as ultrasound pictures or close monitoring of preterm infants. Nevertheless, to ensure that the loss to follow-up remains below our predicted <5% level, we will maintain very close contact with participants; remind them about imminent visits and ultimately conduct home visits if necessary.

1.4 FGLS 2D Ultrasound Measurements

Justification: In preparing the protocol it became clear that more than 6 ultrasound examinations after the dating scan would present unwanted logistical problems and inconvenience to mothers. It was also evident that a minimum growth change has to occur between visits, to be reliably measured by ultrasound, considering the errors in the equipment and observers. Thus, even if velocity growth by unit of time (e.g. one week) for certain parameters is high during some gestational periods, the actual change may not be reliably measured. We have considered alternative spacing between measurements such as longer intervals early in pregnancy (e.g. 8 weeks) and shorter ones in later pregnancy (e.g. 4 weeks). There is evidence, however, that measures such as BPD and femur length continue to increase fairly linearly until 34 weeks without a clear period of very high velocity growth. The logistics of coordinating multiple antenatal visits at different time intervals in a large sample mitigate against adopting variable timings. Finally, as women will be recruited more or less randomly at gestational ages between 9^{+0} to 14^{+0} weeks, we will have a spread of visits throughout pregnancy, which is also advantageous in logistical terms. In a previous report, for example the Guatemalan study, 21% of participants had one scan, 50% had two scans, 24% had 3 scans, and 5% had 4 or 5 scans at varying gestational ages (25). In the Western Australia study, participants were recruited at 16-18 weeks and scanned again at approximately 24, 28, 34 and 38 weeks (26) which we believe is less satisfactory than our schedule. We have explored further this question in a large Scandinavian data set. For 5 measures, in a fixed interval of 7 weeks between measures, they did not detect any problems in modeling growth. Therefore, after the first scan between 9^{+0} to 14^{+0} weeks, we will perform scans at ~5 weekly (±1 week) intervals, i.e. 14-18, 19-23, 24-28, 29-33, 34-38 and 39-42 weeks. Thus, a woman could have scans at 10, 15, 20, 25, 30, 35 and 40 weeks or 11, 16, 21, 26, 31, 36 and 41 weeks depending upon the gestational age at recruitment and duration of pregnancy. This has the advantage of providing greater coverage of the complete gestational period and easier scheduling of the scans.

Schedule: After the dating scan, 6 further visits (for fetal biometry) will be scheduled at ~5 weekly (± 1 week) intervals (i.e. 14-18, 19-23, 24-28, 29-33, 34-38 and 39-42 weeks). Seven measurements will be taken at each visit from 14 weeks onwards: Biparietal Diameter (BPD); Occipito-Frontal Diameter (OFD); Head Circumference (HC); Transverse abdominal diameter (TAD); Anterio-posterior abdominal diameter (APAD); Abdominal Circumference (AC) and Femur Length (FL)

At each visit, the measurements will be obtained 3 times from 3 separately generated ultrasound images in a “blinded” fashion, and submitted electronically (with the associated images) to the Coordinating Unit. The BPD, OFD, HC, TAD, APAD, AC and FL images should fill at least 30% of the monitor screen. The last recorded value of each fetal measurement will be revealed after submission for clinical purposes, as per local protocols. Standardized images will be obtained for all measurements based on internationally accepted protocols (27).

For example, the BPD will be measured from the outer-outer edges of the parietal bones in a cross-sectional view of the fetal head at the level of the ventricles. The measurement should be obtained from an image with the midline echo as close as possible to the horizontal plane with the angle of insonation of the ultrasound beam at 90°. The image should be oval containing a centrally positioned midline echo interrupted in its anterior third by the cavum septum pellucidum. The anterior walls of the lateral ventricles should be centrally placed around the midline echo and the choroid should be visible in the posterior horn in the distal hemisphere.

For peer review only - http://bmjopen.bmj.com/site/about/guidelines.xhtml

The CRL to gestational age conversion will be performed using the charts developed by Robinson and Fleming (23), which remain the most widely accepted reference for early gestational age estimation. This chart is, in any case, based in a cohort of women with known gestational estimation by LMP. This is a circular argument that limits the use of ultrasound alone for the estimation of gestational age. We are aware that an ultrasound expert group in the UK is reviewing the use of these charts. We are, therefore, prepared to consider changing to a new reference if one becomes available.

We also prefer not to include participants in whom fetal size is discrepant from LMP. If we use ultrasound alone to estimate gestational age and the measurement is erroneous, that error becomes built into the growth chart. This seems another circular argument that we must try to avoid. Furthermore, the ultrasound estimation of gestational age has the limitation that all fetuses with a given CRL value will have the same gestational age estimation. This lack of biological variability is a major limitation for evaluating growth. A very interesting discussion on this point can be seen in (28).

In short, we will only consider for recruitment women with a certain LMP and regular periods; from these, we will only select those women with a CRL that is equivalent to their LMP, based on the Robinson and Fleming charts. This population is at the lowest risk of negative pregnancy outcomes (29).

Additional measurements: There is a unique opportunity to complement the study by taking additional measurements, e.g. BPD using the scanner's ellipse facility; trans-cerebellar diameter; humerus, radius/ulna, tibia/fibula and fetal foot length, and Doppler measurements of the umbilical cord artery, middle cerebral artery and maternal uterine artery. However, we are concerned that introducing extra tasks could reduce the overall quality of the seven main measurements and inconvenience the participants. Therefore, the feasibility of taking additional measurements will be evaluated in consultation with each centre before a final decision is made. In general, only some centres are expected to participate in these additional studies, which are mostly descriptive and exploratory with smaller sample sizes than in the main study.

Variability of measurements: We intend to explore several factors that might explain variability, e.g. parity, BMI, gestational age, position/presentation of the fetus, liquor volume, gender and number of measurements. We shall also introduce a comprehensive system for evaluating intra- and inter-observer variability using the 2D and 3D measures, as well as a random sample of retaken measures by an external ultrasonographer in each centre.

Equipment: All the study centres will use equipment with the same characteristics (determined by an independent group), including a state-of-the-art scanner. The staff will be appropriately trained following standardized procedures. The study will provide the equipment free to all the centres in developing countries; centres in developed countries will be expected to provide equivalent equipment themselves, as their contribution to the study. It is very important that ultrasound probes provide an extremely high quality grey scale image. We like to stress that the results of the study will be in the public domain and all manufacturers will be able to use the results free of charge. A detailed description of the methodology for ultrasound measures is presented in Appendix C.

For peer review only - http://bmjopen.bmj.com/site/about/guidelines.xhtml

1.5 FGLS Quality Control Measures

Quality control measures will include a unique system of random evaluation and repetition of ultrasound measurements and continuous real time assessment of all data collected. A limited number of experienced obstetric ultrasonographers and neonatal anthropometrists will form the research teams at each study site. They will be provided with standardized equipment and an operations manual that will describe all measurement techniques, protocols and procedures for training and clinical use in all the centres. The ultrasonographers will receive specific training under the supervision of qualified instructors. Intra-observer and inter-observer measurement errors will be assessed during the training course before initiating the study.

Both the examiner and the instructor will scan each fetus briefly. Each examiner will obtain two images of each fetal anatomical parameter under study. Differences between these measurements will be expressed as the percentage of the measurement obtained from the technically better image. Percentage differences will be used to take into account the increase in the dimensions of the fetal anatomical parameters with advancing gestational age. Percentage differences for each examiner will be averaged and the mean values compared to zero and to the instructor's measurements by t-test. In addition, measurement error will be evaluated by defining a maximum acceptable discrepancy, e.g. 5%, and requiring that 95% of differences lie within that value. This will provide information on the accuracy of individual measurements. The same model of ultrasound machine will be used at all sites, and the equipment will be serviced periodically by specialized technicians as per the manufacturer's recommendations.

1.6 FGLS 3-Dimensional Ultrasound Volumes

3-Dimensional (3D) ultrasound technology, a relatively new scanning technique in obstetrics, offers exciting possibilities to assess the fetus. The technique is based upon volume acquisition using specially designed probes with later, off-line review and "3D reconstruction" on a computer. This allows examination of multiple planes of the imaged structure from 3 different right angles (top-to-bottom; front-to-back and side-to-side). Additionally, a variety of techniques for measuring fetal organ and limb volumes have been described. The multiplanar display allows a clearer and more comprehensive assessment of fetal anatomy, and volume calculation produces more accurate estimates of fetal size and the impact of growth restriction on specific fetal organs (30).

The primary purpose of the 3D component in this study is quality control, i.e. to provide volumetric data to assess the quality of the 2D measures (BPD, OFD, HC, TAD, APAD, AC and FL). Hence, the 3D volumes will be the head (at the level of the BPD), the abdomen (at the level of the AC) and the femur. The task of controlling quality with these data will carried out by an independent group. The technique of 3D volume acquisition is similar for all structures. Initially, the structure to be imaged is identified during the last of the three 2D scans taken. Once imaged correctly, the volume acquisition facility is engaged and the volume obtained over a brief (10-15 seconds) interval and stored in digital form on computer for later analysis, which will be performed in Oxford by the Noble research group after the stored images have been transferred there electronically.

1.7 Criteria for excluding participants from preparation of fetal growth standards

Women in FGLS will receive standardized antenatal care (with some local variations) based on the recommended packages, part of which involves screening for conditions that emerge during pregnancy and is presently used in the hospitals. All women recruited will be followed throughout pregnancy from the time of the first visit irrespective of the pregnancy outcome, except for those with a late diagnosis of multiple pregnancy. However, we do not expect to include all pregnancies in the preparation of the fetal growth standards as some of them may develop conditions that can affect fetal growth. As a general principle, the number of exclusions will be as small as possible. They are likely to be confined to fetuses with congenital abnormalities (based on a final evaluation at birth); severe IUGR requiring early delivery; multiple pregnancies that were not identified at recruitment; mothers diagnosed with catastrophic or very severe medical conditions which were not present at recruitment (e.g. cancer, HIV), those with pregnancy related conditions requiring hospital admission (e.g. eclampsia, placental abruption or severe pre-eclampsia) or those with acute conditions such

For peer review only - http://bmjopen.bmj.com/site/about/guidelines.xhtml

as an episode of malaria infection. Hospital admission *per se* is not a reason for exclusion: women admitted simply for observation will still contribute data to the fetal growth standards unless they develop one of the conditions listed above.

For the sake of clarity, we will produce during the preparatory phase of the study, in collaboration with the Steering Committee and experts in the field, a very detailed list of those conditions, which would result in a pregnancy being excluded from the preparation of the fetal growth standards. Data from preterm deliveries, in the absence of one of the exclusion criteria, will contribute to the fetal growth standards until the time of delivery.

1.8 Symphyseal-fundal height charts

We will produce, in addition to the ultrasound charts, new data on symphyseal-fundal height measurements to update the charts we first produced 30 years ago based on a single sample (31). To produce one, standardized, well-validated, multi-ethnic chart using this selected population to replace several used in primary health care units and hospitals around the world would be extremely useful. We expect that these measures be taken at the same time that the ultrasound 2D measures following the same schedule by specifically trained staff.

1.9 PEA POD measurements

For the Oxford site only, we will include an additional anthropometric measurement of body composition from FGLS pregnancies. Body weight alone can be very misleading; the weight scale cannot tell the difference between an ounce of fat and an ounce of muscle. Body composition estimation provides a measure of the amount of fat mass and the fat-free mass in the body and is now recognized as the most accurate method of measuring infant growth and nutritional status. It is now widely recognised that the accurate assessment and tracking of body composition in the critical period immediately following birth can provide key information in both clinical and research settings. Body composition information can be used to help monitor and evaluate infant growth patterns, optimise nutritional interventions, obtain important feedback during drug treatments, and optimise discharge criteria. Previously, obtaining reliable infant body composition data has been difficult, with available methods limited by problems with accuracy, practicality, invasiveness, and safety. Air Displacement Plethysmography has solved all of these issues by offering accurate and precise measurements of infant body fat and fat-free mass quickly, safely, and comfortably. This technique has now become a vital tool in evaluating infant growth and nutritional status and it has been widely applied in a large number of studies of newborns and infants throughout the world as it is quick and entirely safe.

Over many thousands of applications in infants the use of the PEA POD has not proven to be distressing at all to infants or their parents. In the unlikely event that a child were to be distressed after being placed in the PEA POD (or if for any reason a parent became distressed) then the procedure would be immediately terminated. A further attempt would be made only when appropriate i.e. the child was calm and with consent from the parent.

We will use the PEA POD, which is very similar to a standard neonatal incubator for determining percent fat and fat-free mass in infants. The simple, 7-minute test consists of measuring the subject's mass (weight) using a very precise electronic scale, and volume, which is determined by air displacement plethysmography while the infant lies inside the PEA POD chamber. From these two measurements, the infant's body composition is calculated. For the PEA POD manual of operations see Appendix J.

1.10 1 and 2 year follow-up

All infants born to mothers in FGLS will be followed up at the ages of 1 and 2 (± 2 weeks) to evaluate their postnatal growth. The same methods used to measure the head circumference, weight and length at birth will be employed in the follow-up of infants in this cohort. These will allow us to monitor the growth and health of all infants for whom we have accurate, ultrasound-derived, intrauterine growth patterns and detailed information about pregnancy and perinatal events.

Four additional, non-invasive and painless measures of development will also be taken at the 2 year follow-up using standard protocols as defined by the authors of the respective tests: 1) Assessments of motor skills, cognition, speech and behaviour using a tool

For peer review only - http://bmjopen.bmj.com/site/about/guidelines.xhtml

consisting of the Rapid Neurodevelopmental Assessment of Children (32) and components from the Malawi Development Assessment Tool and the Bayley Scales of Infant Development; 2) Visual acuity and contrast sensitivity measurements using the Cardiff Tests (33); 3) Cortical auditory processing assessments to an auditory novelty, odd-ball paradigm using wireless and gel-free electroencephalography (34), and 4) sleep-wake measures using actigraphy (35).

These measurements will allow us to monitor the growth, health and development of all infants for whom we have detailed ultrasound-derived, intrauterine growth patterns and detailed information about pregnancy and perinatal events.

The additional developmental measurements at the 2 year follow-up will be as follows:

**Functional Domain**	**Functional construct assessed**	**Measure**
Development	Motor skills, cognition, speech and behaviour	The INTERGROWTH-21st Neurodevelopmental Assessment of Children
Vision	Visual acuity and contrast sensitivity	Cardiff tests
Hearing	Auditory processing to a novelty, odd-ball paradigm	Cortical evoked response potentials using electroencephalography
Sleep	Sleep efficiency	Actigraphy

At the 1 and 2 year follow-up appointments of the infants, we will measure maternal weight. We will contact women and their GPs by letter about these appointments and invite them to attend. If they are unable to attend, we will ask them if we can instead arrange a home visit, a telephone interview or if they can complete the questionnaire by post or email.

1.11 Biological samples

Oxford FGLS extension (INTERBIO-21st Fetal & Infant Growth Study)

From 2011 to 2014, we will collect and store maternal blood (12ml) at booking, a urine sample (5ml) at each follow-up ultrasound appointment, as well as maternal faeces (5ml, only if the mother has opened her bowels) and cord blood (12ml)/placental (9x 5mg) samples at delivery (in addition to the pregnancy and fetal growth data) from a total of 1,000 pregnancies. The samples will be used for nutritional and epigenetic studies into fetal growth. The INTERBIO-21st Fetal & Infant Growth Study will continue to monitor fetal growth and the growth and development of the infants using the same protocols as the FGLS component of INTERGROWTH-21st http://www.medscinet.net/intergrowth/protocol.aspx http://www.medscinet.net/Interbio/protocol.aspx?lang=1 (See Appendix K).

Oxford NCSS extension (INTERBIO-21st Neonatal & Infant Growth Study)

From 2011 to 2014, we will collect and store maternal blood (12ml) at delivery, as well as maternal faeces (5ml, only if the mother has opened her bowels) and cord blood (12ml)/placental (9x 5mg) samples at delivery (in addition to the pregnancy data) from a total of 10,000 pregnancies. The INTERBIO-21st Neonatal & Infant Growth Study will continue to monitor the growth and development of the infants using the same protocols as the NCSS component of INTERGROWTH-21st http://www.medscinet.net/intergrowth/protocol.aspx. http://www.medscinet.net/Interbio/protocol.aspx?lang=1 (See Appendix K).

Epigenetic studies in INTERGROWTH-21st participants

Use of Guthrie cards: We will perform the same analyses (i.e. nutritional and epigenetic) on DNA extracted from routinely collected blood spots (stored as Guthrie cards and no longer required for clinical purposes) obtained from the cohort of 5,941 babies born at the John Radcliffe Hospital who participated in the original INTERGROWTH-21st Study. The Guthrie cards will be barcoded, linked anonymously to the clinical data collected from the INTERGROWTH-21st participants and then unlinked.

For peer review only - http://bmjopen.bmj.com/site/about/guidelines.xhtml

Discarded 1st trimester Down's screening samples

From 2011 to 2014, we will collect and store all discarded 1st trimester samples that were used to screen Oxford patients for Down's Syndrome (approximately 7,000/year). The results of the analysis of these samples will be linked to INTERBIO-21st Fetal and Neonatal & Infant Growth Study data, as well as anonymised, routinely collected pregnancy outcome data from all other women. As serum samples from other hospital trusts are also routinely sent to the John Radcliffe Hospital for Down's screening, we will: a) store these samples; b) link them to anonymised, routinely collected pregnancy outcome data from all women delivering at the Royal Berkshire Hospital, Reading; Milton Keynes Hospital, and Buckinghamshire Healthcare NHS Trust (approximately 21,000/year), and c) similarly use them in future studies to test novel biomarkers predictive of adverse pregnancy outcomes.

Sample use

All the samples described above will be used for a range of biochemical, nutritional, genetic and epigenetic studies into placental function and fetal growth. Any unused samples in the future may be donated to the Oxford Radcliffe Biobank for studies into the causes of pregnancy-related problems http://wyvern.ndcls.ox.ac.uk/orb/.

1.11 The Pregnancy Physiology Pattern Prediction (4P) Study

An urgent need to develop an evidence-based, national, Modified Obstetric Early Warning Score (MEOWS) was highlighted in the two most recent Confidential Enquiries into Maternal Deaths in the UK. An essential prerequisite to developing such an early warning system is knowledge of the normal distributions of physiological data (blood pressure, temperature, respiratory rate, heart rate and oxygen saturation) in "low-risk" pregnant women. The 4P Study aims to obtain these longitudinal data, from <14 weeks' gestation to 2 weeks after delivery, from women participating in the Oxford arm of the INTERBIO-21st Fetal & Infant Growth Study. For details of observation measurement in the 4P Study, see Appendix I.

Outcomes

- A national early warning system will be developed for all gestational ages and the immediate post-partum period.
- An NHS web-based programme will be developed, allowing age, parity and gestational-age specific MEOWS chart to be created for individual mothers on admission to hospital.
- An automated, multi-parameter, age, parity and gestational-age specific alerting system will be created for use in monitored areas and hospitals with an EPR system.
- Fetal size will be correlated with maternal physiological values.

2. PPFS: to develop the Preterm Postnatal Growth Standards (Activity 2)

2.1 Follow-up of preterm babies in FGLS and INTERBIO-21st Fetal & Infant Growth Study

All preterm newborns (>26^{+0}<37^{+0} weeks) from the FGLS and INTERBIO-21st Fetal & Infant Growth Study cohorts will be followed-up for 8 months after delivery and at 15, 18 and 21 months to evaluate postnatal growth. The same anthropometric measurement techniques to measure the head circumference, weight and length will be used to follow-up infants in these cohorts. At 15, 18 and 21 months, we will use the same data collection forms that are used to follow-up these infants at 1 year. These measurements will allow us to monitor the growth and health of all infants for whom we have detailed ultrasound-derived, intrauterine growth patterns and detailed information about pregnancy and perinatal events.

In addition, we will assess the motor development of the preterm cohort every month from 4 months of age to 8 months of age using the motor development assessment questionnaire, which is part of the 1 year assessment. This consists of two parts: 1) a questionnaire for the parents about the age their child achieved some gross motor milestones, and 2) a questionnaire completed by the Measuring Team reporting whether or not the child demonstrated those milestones during the visit.

At 2 years of age, the same anthropometric measurements (head circumference, weight and length) will be taken. In addition, we will also measure developmental outcomes in the

For peer review only - http://bmjopen.bmj.com/site/about/guidelines.xhtml

children. These measurements will include a test of hearing (cortical auditory processing); a test of visual acuity and contrast sensitivity; an assessment of the child's cognitive, language, motor and behavioural development, and an assessment of sleep. Section 1.9 details the techniques used to measure these developmental outcomes.

These measurements will allow us to monitor the growth, health and development of all infants for whom we have detailed ultrasound-derived, intrauterine growth patterns and detailed information about pregnancy and perinatal events.

We plan not to include preterm newborns ≤26 weeks (only ~3% of all preterm newborns) because of the very high morbidity/mortality in this group and need for very special intensive care. A fixed follow-up period was chosen to simplify organization of the study and reduce loss to follow-up. Nevertheless, an analysis based on the time from conception (corrected age) will be performed to compare preterm babies with their *in utero* counterparts conceived around the same time. This length of follow-up should minimize a) inconvenience to the mothers; b) the need for home visits, and c) the study's cost and complexity. An analysis based on the time from conception (corrected age) will be performed to compare preterm babies with their *in utero* counterparts conceived around the same time. Although we want to produce growth charts for the first 6 months of life, we have extended the follow-up period to 8 months for these infants to avoid the so-called "right-edge" effect in the construction of the growth standards.

Exploratory sub-group analyses will also be conducted, e.g. very preterm infants <32 weeks (~20% of all preterm newborns) and induced for medical reasons vs. spontaneous preterm deliveries. However, to be included in the PPFS growth charts, newborns have to meet the criteria decided a priori. A detailed protocol describing clinical conditions for inclusion in the final growth curves has been prepared in consultation with expert neonatologists before any data analysis is undertaken.

This protocol will focus on definitions that can be made operational across institutions. Newborns will be excluded retrospectively if they have severe medical morbidities likely to impact upon growth and congenital malformations. (See also section 2.4).

Clearly, for some subgroups of very preterm infants this will represent only an exploratory analysis; however, it will still be done because of the unique opportunity to study continuous fetal-preterm growth. It is expected that many babies, especially those born <32 weeks, will be managed in some form of neonatal intensive care unit. Measurements of these babies will be taken using the study protocol, but in accordance with the clinical status of the infant and the unit's protocols. It is clear that to ensure that "clinically stable" babies are comparable across different NICUs, standardization and continuous monitoring are needed. Ultimately, in such an acute clinical care setting, the final judgment will depend on the attending doctor; realistically, this is how the standards will be used in practice anyway.

2.2 PPFS Anthropometric Measurements

The anthropometric measurements will include weight, length and head circumference. The three measurements (and a standard clinical evaluation) will be taken every 2 weeks during the first 8 weeks, and then every 4 weeks until 8 postnatal months, using essentially the same methodology and operating manual employed in the WHO Child Growth Study (6). The only difference will be that in the proposed study all measures, interviews and clinical evaluations will be conducted at a special follow-up clinic in the corresponding hospital. Routine home visits are not planned, except for those mothers who do not comply with the protocol's scheduled visits. It is expected that the preterm babies will have a maximum of 11 follow-up visits over 8 months, but this number may be reduced for some as complications, including death, are expected especially in the very preterm subgroup. Abdominal circumference will not be included as it is not used in routine neonatal practice and respiratory movements in these tiny newborns make the measurements unreliable.

Standardized, electronic, digital, newborn weighing scales will be used and serviced regularly; they will be replaced if they are faulty and cannot be repaired. All anthropometrists in the study will be trained centrally; they in turn will train the nurses/midwives in how to use the scales. We will follow the same procedures used in MGRS to certify all staff responsible for taking the measurements, as was the case in our own previous randomized trials. During routine measurement sessions, two anthropometrists will independently take duplicate

For peer review only - http://bmjopen.bmj.com/site/about/guidelines.xhtml

measurements. Maximum allowable differences between duplicate measurement values will be checked to detect outliers and trigger immediate re-measurement if necessary.

2.3 PPFS Quality Control Measures

An Anthropometric Data Quality Unit, organized on similar lines to the Ultrasound Quality Control Unit, will be created. Its role will be to conduct, following the WHO MGRS's training procedures, the standardization, monitoring, site visits, and retraining when necessary of all staff responsible for anthropometric measurements (MGRS Manual of Operation, WHO Geneva). Using the on-line data management system, samples of measurements will be obtained to evaluate variability and consistency within each site and across sites. The same protocol will be followed for measures at birth for all newborns in the fetal and newborn studies. For the preterm follow-up we will use the MGRS protocol but include only weight, length and head circumference. We will assign 2 staff at each centre to take all newborn (and postnatal follow-up) anthropometric measurements. This will permit replication of the WHO MGRS measurement standardization protocol (6).

As stated above, the standardization of all anthropometric measurements is a central element and challenge of this protocol. The measurers (or lead measurer from each site) will be brought together at a central location for two initial training meetings to be conducted by experienced staff from the WHO-MGRS team with the assistance of the MGRS anthropometry video. This video will be used for local training as well as for on-going standardization activities. The study will provide all sites with a new infantometer(s) with digital reading for infant length measures. For birth weight, hospitals will use the balances they are currently using but they will be calibrated daily. Other equipment will be calibrated at least once a week during data collection. The overall sample will be also be monitored daily, using the on-line data entry system, for unexpected values and outliers so that measurements can then be repeated within a few days, if appropriate.

2.4 Infant Morbidity During Follow-Up

For the Preterm Follow-up Study, we will use an indicator of morbidity at entry and an extended version for the follow-up itself. The protocol states (see section 2.1) that the study will exclude newborns ≤26^{+0} weeks (only ~3% of all preterm newborns) because of the very high morbidity/mortality in this group and need for very special intensive care. A detailed protocol describing clinical conditions, "minimum criteria", for inclusion in the final growth curves will be prepared a priori before any data analysis is undertaken. Babies that do not meet the “minimum criteria” will nevertheless be followed up to form a subgroup for comparative analysis with the “healthy” preterm infants. Efforts will therefore be made to standardize the major components of infant care throughout the participating institutions. The protocol will focus on definitions that can be made operational across institutions. Criteria are likely to include any morbidity severe enough to have a negative impact on growth, feeding or the feasibility of measuring the infants at the required times.

2.5 Infant Feeding Practices

We expect newborn feeding to be protocol driven and based on a) the recent WHO recommendations (Edmond and Bahl "Optimal feeding of low-birth-weight infants" WHO, 2006) and b) standard NICU practice for clinical status. Descriptive data, collected in an adapted version of the WHO MGRS form, will serve to document that the centres conformed to recommended feeding patterns and clinical practice. The comparison with intrauterine charts is an important, biological secondary objective, but the main objective of the PPFS is to describe the postpartum growth that can be expected, or aspired, to by "healthy preterm newborns".

It is anticipated that all the sites will adhere to internationally recommended standards for feeding preterm infants; see review (36). The recommended first choice for LBW infants is breastfeeding or mother's own expressed milk. This is associated with the lowest incidence of infection and necrotizing enterocolitis, and best outcomes in terms of neurodevelopment. Where that is not possible, the next best choice is donor human milk and, in third place, preterm infant formula (Edmond and Bahl "Optimal feeding of low-birth-weight infants" WHO, 2006).

For peer review only - http://bmjopen.bmj.com/site/about/guidelines.xhtml

3 NCSS: to develop Newborn Birth Weight for Gestational Age Standards (Activity 3)

3.1 NCSS Individual Entry Criteria

The Newborn Cross-Sectional Study will include a) all babies born to mothers in FGLS and b) all other babies born at the study centres during a fixed 12 month period. The vast majority of babies will have had their gestational age confirmed by an early ultrasound because all the hospitals included in INTERGROWTH-21st sites should already have a policy of checking gestational age with an early dating scan. Measures will be taken during the study period to reinforce this policy to ensure that all newborns have their gestational age at birth corroborated by ultrasound scan estimation early in pregnancy. The strategy of including the complete newborn population will allow us to study a large number of LBW infants and subgroups under a wide range of conditions that can affect these outcomes. We will also be able to compare the total populations across centres in terms of socio-demographics and pregnancy outcomes to confirm that the populations contributing to the new standards have the required characteristics.

3.1.2 Constructing the Standards

For construction of the birth weight for gestational age standards, babies born to mothers without health problems likely to influence fetal growth will be selected from the total number of births at each centre. However, the total population of newborns during the same study period will be used to explore Objective III (determinants of LBW and its components, preterm delivery and impaired fetal growth, under current healthcare conditions). In addition, we plan to use an outcome-based approach, based on recent recommendations.[a]

3.2 NCSS Anthropometric Measurements

All babies born during the study period will have these anthropometric measures taken within 24 hours of delivery: weight, length and head circumference. To select those newborns eligible for the NCSS (newborn standards), we will use the same criteria as for the fetal growth study.

Standardized, electronic, digital, newborn weighing scales with a precision of 10 grams will be used and their calibration status will be checked daily; they will be replaced if they are faulty and cannot be repaired. We shall also provide all clinics with standardized infantometers for length (precision 0.1 cm) and tape measures for head circumference (precision 0.1 cm); these will be similarly calibrated and maintained. All anthropometrists will be trained centrally and monitored during the study following standard procedures by the Anthropometric Standardization Unit; they in turn will train the nurses/midwives in how to apply the study's measurement protocol. We will follow the same procedures used in MGRS to certify before and during the study all staff responsible for taking the measurements, as was the case in our own previous randomized trials. As in MGRS, routine data collection will comprise duplicate measurements of weight, length and head circumference taken independently by two anthropometrists. They will check maximum allowable differences between their replicate measurement values immediately the measurements. These maximum allowable differences are approximately twice the Technical Error of Measurement of a well-trained observer (e.g. 0.7 cm for length and 0.5 cm for head circumference). If the maximum allowable difference is exceeded, the anthropometrists will immediately go on taking other duplicate measurements until the difference is acceptable. Replicate measurement values will be averaged before analysis to increase accuracy further.

3.3 NCSS Quality Control Measures

The same quality control measures and standardization strategy (adapted from the WHO Child Growth Study protocol) (7) which are described above for PPFS will be employed for the three measurements to be taken for the whole study population in NCSS. A special Anthropometric Data Quality Control Unit, independent of the Study Coordinating Unit, will

[a] Joseph KS, Fahey J, Platt R et al. (2009) An outcome based approach for the creation of fetal growth standards: do singletons and twins need separate standards? Am J Epidemiol 169:5:616-624

For peer review only - http://bmjopen.bmj.com/site/about/guidelines.xhtml

be responsible for the monitoring and quality control of these measurements during the entire study.

3.4 Follow-up of the NCSS

All newborns during the study period, including those on NICU or special care, will be followed on a daily basis until hospital discharge to detect neonatal death and document severe morbidity. We will make strenuous efforts to coordinate and promote evidence-based care for the preterm neonates using materials developed as part of the WHO best practice programme, by liaising with the lead neonatologist in each NICU before and during the study. We recognize that differences in practice will persist despite our best efforts. However, we believe this is unavoidable in a very pragmatic study such as this, which is trying to reflect what happens on a daily basis in clinical practice. Furthermore, we will similarly make strenuous efforts to standardize the main protocols for feeding practices in each NICU before the study starts. During the routine site-visits by members of the Study Coordinating Unit and the Anthropometric Team we will monitor the implementation of the protocols.

3.5 Biological Samples

We have debated the issue of anonymized genetic/epigenetic data at great length. In principle, we would not wish to miss an opportunity to put together what would be a unique databank. However, we are very concerned about a) the effect that a request to collect DNA might have on recruitment rates; b) ethical questions that might be raised by IRBs; c) the additional time required to obtain informed consent, and d) the extra cost of processing the samples. Having said this, it nonetheless remains an important issue. Therefore, we shall bring together a group of international experts and potential academic partners, to discuss the advantages and disadvantages of collecting biological samples for a specific hypothesis relating to fetal growth and/or preterm delivery. The final decision will depend largely on whether the study has sufficient power to answer a specific question, relating for example to the effect of a genetic polymorphism on a pregnancy outcome. After much discussion, it was finally decided to collect biological samples from FGLS and NCSS pregnancies from 2011 onwards. For details of biological sample collection refer to Section 1.11 and Appendix K.

3.6 Severe Perinatal Morbidity and Mortality Outcomes

Birth weight for gestational age standards will be related to indicators of perinatal outcome to establish risk levels associated with different growth patterns. The ‘ideal’ outcome is perinatal mortality, but its anticipated infrequent occurrence in this low risk population makes it unrealistic to have a sample large enough for the necessary number of events across the gestational age distribution (perinatal mortality is expected to be close to 1% in these populations). We have therefore decided to use an un-weighted composite outcome including at least one of the following conditions: stillbirth, neonatal death until hospital discharge of the newborn, newborn stay in NICU for ≥7 days or other severe neonatal complications. We have used such an outcome recently (12, 13); it requires limited standardization of clinical diagnoses across hospitals and is well accepted as a marker in large, international, population based studies of newborns that are severely ill.[b] It could be argued, however, that intrapartum stillbirth may not be related to fetal growth and should not be included in this index. We believe this is a valid point but as it will not be possible to separate those intrapartum deaths that are related to IUGR from those that are unrelated, we suggest keeping the index as it is. As we expect a very low fetal death rate, it should not have a major impact on the observed rates.

We believe this is a good proxy for adverse perinatal outcomes across countries. We have used it as a primary neonatal outcome in recent publications and it has been well accepted. Its only disadvantage is that it risks excluding from the total number of early neonatal deaths some cases amongst healthy, mostly term babies delivered vaginally who, after hospital discharge at 48 hours, develop severe complications or death up to 7 days

[b] Others have also used these composite indices of neonatal morbidity (Hannah ME, Hannah WJ Kewson SA et al (2000); Wapner RJ, Sorokin Y, Thom EA (2006); Joseph KS, Fahey J, Platt R (2009)).

For peer review only - http://bmjopen.bmj.com/site/about/guidelines.xhtml

post-natally without returning to the same hospital. However, missing these isolated cases is preferable to performing thousands of unnecessary home visits.

Data from our population-based studies in some of the sites indicate that the incidence of this outcome is close to 5%. We will confirm this estimate during the preparatory phase of the study in the study centres. We will also reach consensus with the investigators and consultants on the few very severe clinical conditions to be included in the composite outcome in addition to perinatal death and ≥7 days NICU stay.

4. On-line Data Management and Statistical Analysis

All data will be entered into an on-line data management system specifically developed for the study; it will include a system for direct transfer of blinded data from the ultrasound machines to the database. This on-line system has the practical benefit of allowing on-going quality control, correction of errors or missing values and the initiation of data analysis soon after data collection is completed. We have previously used such a system in very large multi-centre studies (12) and randomized clinical trials in developing countries, and we are confident that there will be few problems in adapting this concept to the proposed study. The system will be field tested during 2008 using the data flow model shown in Appendix B.

This system will be used for data management and monitoring all sub-studies, including patient recruitment and follow-up, and is based on the INTERGROWTH-21st Electronic Data Management System (IDAMIS). The system permits all participants' data to be incorporated contemporaneously into the data files via the Internet. Included within the system is a review process to ensure that all data are complete, and that the research team is notified about imminent patient visits and expected delivery dates. At an institutional level, each centre will have its own progress enrolment charts and weekly recruitment targets. The system will also provide the Data Coordinating Unit with a detailed daily record of patient enrolment and data entry, at both individual and institutional levels to monitor progress against the milestones listed in the protocol. Corresponding actions, such as telephone calls, web conferences or site visits will take place within a week of detecting a problem in a centre to ensure that appropriate corrective measures are introduced. Lastly, the system will allow the database to be analyzed as soon the data collection period has ended.

Data from all sites will be pooled to construct the curves for international applications using the WHO Child Growth Study analytical strategy (8). Data analysis will be conducted following the same strategy used in the construction of the WHO MGRS curves during Years 3-5. It will be coordinated by the Statistical Director (D. Altman), consultant statistician/epidemiologist and the team's senior investigators. Extensive secondary analyses of these data will be conducted including the development of velocity standards, thereby contributing in a major way to answering questions relating to clinical practice and public health now and for years to come.

The appropriateness of pooling data from all sites will be assessed by comparisons of site means, standard deviations and the 3rd and 97th centiles to the pooled values of primary data. Consistent differences ≥ 0.5 SD between the mean values of individual sites and the pooled sample will be used as a pre-set trigger for considering whether to adjust by site for purposes of pooling data. This has to be supported by site-specific consistent differences across primary measures. For the WHO-MGRS it was concluded that data from all sites were remarkably similar and thus should be pooled (8). This is the strongest biological argument to date and will be the basis for the conceptualization of our analysis.

The planned approach will be based on the experience of the WHO-MGRS adapted to the fetal growth parameters (37); we shall follow the same exploratory strategy used in this study. We are aware that these analyses may not be automatically applicable, but we feel comfortable that the considerable experience of this team of statisticians in carrying out a similar study will enable them to select a final model that is fit for purpose. The methods do not require similar intervals. We are not making any assumptions about the shapes of the individual curves, except that they will be smooth.

For the physical growth standards, a class growth distribution that can fit the data for all measurements will be explored with an appropriate smoothing technique to generate centiles. Candidate distributions (e.g. Box-Cox t, modulus-exponential-normal, Box-Cox-power-exponential, Johnson's S_U or Johnson's S_B) will be compared on the basis of goodness of fit for each age group using the following diagnostic tools: (a) Log-likelihood

For peer review only - http://bmjopen.bmj.com/site/about/guidelines.xhtml

comparisons, (b) Q-Q plots (i.e. comparing empirical and distributional centiles), and (c) Comparisons of observed percentages that occur below estimated centiles against expected values. Once the most appropriate distribution(s) for our data is (are) identified, smoothing techniques will be tested against the same diagnostic criteria applied to the selection of distributions such as polynomial smoothing, the Box-Cox t distribution that smoothes using natural splines and a variation of it with the Box-Cox-power-exponential distribution. Sir David Cox (University of Oxford Statistics Department) who advised us on another pregnancy-based study, will be invited to contribute to the selection process.

It could be argued that only parous women should be included in the construction of the standards. Furthermore, it would reduce the incidence of LBW babies amongst the mothers recruited as nulliparous women are known to be at higher risk of LBW babies. However, it is also the case that long-term outcomes in newborns born to parous versus nulliparous women have similar overall morbidity and mortality rates. Overall, we believe that the advantages of recruiting solely parous women are outweighed by the loss in external validity and credibility in doing so. Therefore, we intend including all women regardless of parity but we will try to recruit similar proportions across the study sites. We will also explore in the analysis if, in this sample of healthy women, parity is an important effect modifier for fetal growth, but we do not expect to produce parity-specific fetal growth standards. In addition, based on the same biological principles, sex-specific charts should be constructed for fetuses as they have been for newborns. However, we do not plan to separate fetuses by sex as this is not a routine practice and it is unlikely that it will became one in the future. Conversely, separate sex-specific charts will be developed to monitor the postnatal growth of preterm babies.

5. Sample Sizes for Objective I (Activities 1 - 3)

General principles: The precision of growth chart centiles is determined by several factors, of which the most important is sample size. Other factors are also relevant, including study design (cross-sectional vs. longitudinal), the timing of measurements, and the method of curve-fitting. Many criteria can be used to estimate sample size, but three are considered critical here, namely the precision of: a) a given centile at a particular gestational age; b) the logistics of selecting and following-up a large cohort, and c) the rate of the expected perinatal outcome for Activity 3, in Objective I. (The outcomes to be used in Objectives II-III are either continuous variables or far more prevalent than severe neonatal morbidity/mortality, i.e. a preterm delivery rate of 9-10%, and therefore sample size limitations are of less concern for these parts of the study). Sample sizes have been estimated taking each of these criteria into account. The numbers quoted below are combined figures for all study sites.

It is possible that the WHO-MGRS findings of similar infant growth patterns amongst different ethnic groups may not apply to fetal growth, even though recent data for US white/black newborns support the concept of pathological rather than physiological factors being responsible for observed variations (16). We question why fetuses should grow differently to infants or be affected differently by environmental influences, or why biologically different growth patterns should exist across ethnic groups. Nevertheless, we will make all practical efforts to obtain the largest possible sample size per ethnic group to allow separate evaluation in case the alternative hypothesis (different fetal growth by ethnic group) is observed. Even if this is the case for some groups it is very unlikely that all ethnic groups will have biologically relevant different growth patterns.

FGLS: To obtain complete data from 4,000 pregnancies at 8 study sites, 500 mothers will have to be enrolled at each centre. Data from previous years will be used at each study site to estimate the expected number of eligible women available and the recruitment rates. We expect that ~75% of the total pregnant population at the sites selected will be eligible for this cohort, although local investigators will be strongly advised to be conservative in their estimates of compliance rates and to allow for attrition due to other reasons. The 4,000 women should provide close to 800 cases of each of the 5 general ethnic groups. Sample size calculations (15), based on a width of the 95% CI of the 5th birth weight percentile of 2.5% at term (2,700g), demonstrate that 600 women per ethnic group should be adequate, after excluding complicated pregnancies and women lost to follow-up. This sample size is larger than most previous studies and adequate, we believe, to produce reliable curves.

For peer review only - http://bmjopen.bmj.com/site/about/guidelines.xhtml

However, we will not produce ethnic-specific standards because such pregnant populations are rarely representative of a unique ethnic group, particularly in urban areas in developing and developed countries. It would therefore be impractical to alternate between standards based on each patient's ethnic classification, even if this could be done. We will not be able to estimate outcomes across groups with any degree of power but that is not an objective of this study. These numbers should also fulfill the requirements for PPFS of ~360 preterm infants (assuming a preterm rate of 9% based on our previous study involving similar populations (12)).

We estimated that fewer than 5% of women will be lost to follow up (the figure is usually ~ 3% in our large trials). We also adjusted for the fact that 10-15% of women will be excluded from the preparation of the fetal growth standards because they will have developed problems severe enough to have affected fetal growth.

PPFS: A cohort of ~360 preterm babies will be recruited from FGLS for this study. It is expected that the gestational age distribution will provide 310 babies >30 weeks gestation. Only the subgroup of babies free of major clinical problems will be used to create the standards. We recognize that the sample size calculation here is influenced by logistic issues and the availability of newborns from FGLS rather than statistical calculations. However, it is still large by preterm study standards and we shall have very detailed follow-up data. We consider that the possibility of having a full set of fetal and newborn growth patterns from a cohort of preterm newborns is a biological priority even if we shall not have the power to explore other subgroup analyses such as gestational age sub-groups or early postnatal morbidity. Postnatal growth from healthy populations has been shown to be similar among ethnic groups (38, 39).

NCSS: The sample size calculations for this component of the study depend mostly on the number of preterm newborns needed at the lower end of the gestational age distribution and the perinatal outcome selected. A fixed data collection period (12 months) is planned for all hospitals but it is clear that some variability will have to be accepted considering the size of each institution and the population characteristics. From the pool of all newborns, we will select the ones born to eligible women using the FGLS criteria listed above. We expect the refusal rate to be minimal. We will aim for a total of 50,000 newborns with very detailed information which will provide, based on data from similar institution (40), a sample of ~75% eligible babies for the newborn charts, including ~1,800 with a severe outcome using our established criteria. Of these 1,800, we expect that 70% (about 1,200) will be between 26 and 36 completed weeks of gestation.

Missing values: We consider that women without newborn data cannot contribute to any of the three studies. Fetal deaths and newborns with congenital malformation will be excluded. All women should have at least 2 follow-up values after the dating scan to be included in the fetal growth study.

B. Objective II: Activities 4 - 6

Objective II will be achieved by the following three activities:

1) Preparation of computer files of 2D ultrasound measures for 3-week gestational windows between 20-34 weeks;

2) Development of analytical strategies for the construction of predictive models to estimate gestational age during mid-late pregnancy and

3) Completion of prediction model to estimate gestational age during mid-late pregnancy based on several ultrasound measures obtained at a single visit. Ultrasound-based gestational age estimation in infrequent attendees or women with limited access to care is usually based on a single ultrasound measure (e.g. BPD), but the estimates have large errors and wide confidence intervals. We are planning to develop equations that use several ultrasound measures obtained at a single visit. If we achieve this, it will be a major contribution to the care of high-risk women attending hospitals on only one or two antenatal visits late in pregnancy.

Statistical strategies for the production of the prediction models include multiple regression analysis using gestational age at delivery as the gold standard (dependent variable), then recalculating the expected gestational age at different time points. The independent variables or predictors will be the basic ultrasound measures at a given

For peer review only - http://bmjopen.bmj.com/site/about/guidelines.xhtml

gestational age window, as well as other clinical parameters that are available to the staff during the antenatal care.

C. Objective III: Determinants of LBW and its components, preterm delivery and impaired fetal growth, under current healthcare conditions (Activity 7)

The complex interactions between risk factors, clinical presentations and underlying biological processes are poorly understood in relation to adverse perinatal outcomes, especially LBW which is such a heterogeneous entity. This has been a major limitation in preventing preterm delivery and impaired fetal growth. We therefore aim to explore risk factors and perinatal outcomes for LBW (<2,500g), preterm delivery and impaired fetal growth in the entire NCSS study population of 50,000 newborns. For the identification of risk factors, data collected during routine and specialist antenatal care from ALL women delivering in the institutions over a fixed period will be included in the study. A summary antenatal and delivery form will be introduced into these institutions, which will conform with a) the new WHO model of antenatal care for basic routine care (10) and b) local protocols for special cases, standardized by us from previous trials in pre-eclampsia, hypertension, urinary tract conditions, and intra- and post-partum care. An important conceptual issue is that we do not aim to detect any new, unexplored risk factors. Rather, we plan to determine how risk factors, that are routinely recorded during standard antenatal care, are distributed in the preterm/IUGR cases and their subgroups across these populations, in view of the considerable heterogeneity in risks and outcomes within these two conditions.

We plan to investigate the determinants of preterm delivery and IUGR in sub-groups from clinical, routine laboratory, demographic and socio-economic variables obtained from all women attending the study centres without any exclusion (as opposed to FGLS which aims to produce standards from a sample of selected, healthy women). It would, of course, be very interesting to collect more detailed information about other variables or test biomarkers of, for example, infection. The question, as always, is when to stop adding more variables to an already complex study. One alternative is to add ancillary studies to selected centres that are interested in collaborative research with other groups. It would certainly not be impossible for us to coordinate the collection, storage and testing of such samples (as our group has experience of collecting thousands of samples for similar studies) but funding is not available for such an activity at this point.

We will apply standard statistical strategies (e.g. logistic regression analysis) using the ~4,500 preterm newborns or IUGRs from the total NCSS study population to study these relationships compared to term babies, and we shall explore some recently proposed analytical strategies as well (41). We will consider subgroups of preterm deliveries, e.g. induced vs. spontaneous deliveries; premature rupture of membranes; pre-eclampsia related vs. unexplained impaired fetal growth, as well other pathological and physiological conditions (42, 43). The adjusted ORs and confidence intervals for these risk factors as related to the different subgroups will be estimated and biologically plausible interactions will be explored. The association between subgroups of newborns and perinatal outcomes will be also examined. We also intend to explore several factors that might explain variability in fetal growth, e.g. parity, BMI, gestational age, position/presentation of the fetus, liquor volume, gender and number of measurements. We shall also introduce a comprehensive system for evaluating intra- and inter-observer variability using the 2D and 3D measures, as well as a random sample of retaken measures by an external ultrasonographer in each centre.

D. Objective IV: Acquisition of additional 3D images to create an anatomical and growth databank of individual fetal organs (Activities 8 - 9)

Activity 8: 3D Data Collection during FGLS

Data collection for this activity will be conducted immediately after the 2D measurements have been taken for FGLS. The 3D volumetric images of the head, abdomen, and femur will be acquired using the methods described in Section 1.6. We expect that the provider of the equipment will provide technical assistance to optimize data collection.

The primary purpose of the 3D component is to provide volumetric data to assess the quality of the 2D measures (BPD, OFD, HC, TAD, APAD, AC and FL). The secondary objectives are a) to evaluate currently used ultrasound measures of fetal volume with the

For peer review only - http://bmjopen.bmj.com/site/about/guidelines.xhtml

expectation that some of them can contribute to the improvement of the presently used estimation of fetal weight and b) to establish a unique "biological databank" for future research. Additional 3D volumes of the chest (at the level of the 4-chamber view of the heart) and the humerus will be taken specifically for these purposes in some centres.

The secondary objective a) is very interesting from a clinical point of view as most of the ultrasound-derived equations for estimation of fetal weight have low predictability and were developed using data collected from small studies with less sophisticated ultrasound equipment than is now available. Thus, the feasibility of combining 2D and 3D measures will be explored including the possibility of doing so across gestational ages. We expect to evaluate the additional benefits that could be obtained by adding 3D values in a sub-sample of the study.

Other uses of the 3D data are planned: for example, the accuracy of fetal weight estimation based on 2D measures is known to be poor in clinical practice. It is expected that 3D data will improve the formulae that are still in use despite being produced over 20 years ago. Furthermore, the likelihood of low-cost, portable 3D equipment being available in the future raises the possibility of managing high-risk pregnancies in rural or distant regions by sending the images to a referral centre rather than the mother herself. We plan to take the final decision regarding the amount of 3D data to be collected and the number of participating sites after conducting a pilot study assessing the human resources available at the centres, the need for additional training, the time that 3D scanning adds to each visit, equipment considerations and the feasibility of transferring the very large datasets to the Coordinating Unit in Oxford. Issues we shall explore in the pilot study include: a) difficulties obtaining individual organ volumes (44); b) the need to obtain at least two good volume measures from most of the areas of interest which may require 20-30 minutes additional scanning; c) the need for the audit staff to know the values of the calculated volumes and the way the operator arrived at each volume which could introduce considerable bias, and d) how to save and transmit the volumetric data reliably given the risks of a hard drive crashing or being corrupted. In any case, it is unlikely that we shall record more than the 5 volumes listed above at each visit, and it is also worth mentioning that the Gates Foundation has not funded the 3D component of the study.

We have debated the issue of adding more ultrasound measures (i.e. we have a unique opportunity that may not be repeated again) with the Ultrasound Advisory Group and other experts in the field, as we are concerned that the potential list of additional 2D/3D measures is long. As is the case in selecting which data to collect in any large study, one has to balance the enthusiasm of the researchers against pragmatism. We have chosen to concentrate on taking a few measures well (and more than once) in the time available during each scanning session rather than risk the possibility that additional measurements (including some not used in routine clinical practice) will be taken hurriedly. Of course, it will be possible to take further 2D measurements retrospectively for research purposes, if needed, from the acquired 3D volumes.

Activity 9: Preparation of 3D Files and Exploratory Analyses

There are two goals of the 3D component of this project. The first is to understand better the clinical advantages of making manual and automated fetal measurements on 3D v. 2D data. This should lead to a clearer understanding of IUGR and the relationship between different growth patterns and fetal/neonatal outcomes, as well as the impact of delayed growth in individual fetal organs and health care problems in adult life (e.g. an increased risk of osteoporosis). The second goal is to derive statistical models of the fetus or parts of the fetus to provide informative visualizations of nominal shapes and growth data at different gestational ages.

One possible approach will be to build parts model of the fetus, and to deform it to different instances of data acquired at the same gestational age. This will not be a simple task and will require theoretical and technical advances in biomedical image analysis. The research will be led by A.Noble, Professor of Engineering Science, University of Oxford, and forms the basis of a separate grant application to a UK agency, the UK Engineering and Physical Sciences Research Council. The aims of Activity 9 are simply therefore to prepare the 3D images and commence these exploratory analyses. Examples of what is currently possible include studying the relationship between a) brain growth and neonatal/infant

For peer review only - http://bmjopen.bmj.com/site/about/guidelines.xhtml

neurodevelopment (45) and b) fetal bone growth and neonatal/infant bone density to assess the origins of osteoporosis (46).

Ethical Issues

The study will comply with the International Ethical Guidelines for Biomedical Research Involving Human Subjects. Ethical safeguards will include:

Ethical approval at international, national and local levels: The study protocol will be submitted to the relevant UK ethics committee in keeping with the University of Oxford's responsibilities as the study sponsor, as well as the appropriate national bodies of the countries included in the study (if required) and all local ethics committees. Ultrasound and anthropometric research involves minimal, if any, risk to the mother and fetus and is part of routine care in these institutions.

Individual informed consent: Written informed consent will be obtained from all women enrolled in the study after the study objectives and procedures have been described in detail. The information will be provided in written form (translated into all the required languages) and orally by the researchers themselves based on a standard template, which can be referred to whenever needed. Women will only be recruited if it is clear they understand what the research entails and once they have had sufficient time (at least 48 hours) to consider whether to participate or not. All information collected in these studies will be made available to the clinicians responsible for the women's care; however, the 2D ultrasound measurements will only be made available after the data have been submitted on-line (see 1.2 above).

Discontinuation: Women who have agreed to participate in FGLS may decide to leave the study at any time without adversely affecting their care in any way.

Confidentiality: All phenotypic and imaging data will be available for clinical use at each centre. For research purposes, privacy rules will be maintained in line with the *Health Insurance Portability and Accountability Act of 1996* (HIPAA). All individuals will be de-identified to ensure confidentiality. At the Coordinating Unit, all phenotypic and imaging data will therefore be stored, transmitted and analyzed anonymously.

Data sharing for clinical care: All the clinical data we plan to collect are being obtained anyway as part of routine clinical practice in these institutions; the data will therefore will be available to the providers at all times. Our proposed policy with regard to the ultrasound measures is as follows: a) the gestational age estimate will be incorporated into the medical records; b) an ultrasound examination for structural congenital malformations will be performed at the time of the 3rd scan, if this conforms with local practice, and the results will be incorporated into the medical records; c) as far as 2D ultrasound measurements are concerned, after the blinded values have been submitted electronically to the dataset, the last measurement will be provided for clinical use, and d) no 3D data will be provided for clinical use.

Finally, there are concerns about the misuse of ultrasound for sex selection in places where it may be used (especially if the practice is illegal). We shall therefore be very vigilant in regions where this practice is known to occur and monitor sex ratios in the sample continuously.

E. Challenges

Implementing this project presents major challenges for the research team. However, our experience in conducting large-scale studies in pregnancy means that we are well prepared to face the challenges, some of which may be difficult to resolve:

Recruitment: We do not anticipate capacity problems in recruiting a large number of women (4,000) from 8 centres, but it remains unclear what proportion of women in each centre will actually be eligible for FGLS given the inclusion criteria. We anticipate an average eligibility of 75%, and expect the centres to devise local strategies to maximize that number.

Follow-up: We do not anticipate problems retaining pregnant women during follow-up but we do recognize that such a large follow-up study requires a commitment from participants and staff. In addition, the planning and coordination of the ultrasound scans with only one ultrasound machine available per centre will require considerable organization in the hospitals that should not be underestimated. Follow-up of the newborns presents the following challenges: a) difficulties measuring very small infants, especially those in an

For peer review only - http://bmjopen.bmj.com/site/about/guidelines.xhtml

incubator; b) morbidities associated with prematurity, which may modify the measures to be taken; c) mortality affecting the final sample size, which explains why we intend focusing on healthy preterms, and d) follow-up after discharge is a problem in any such study, but we expect that adherence to the protocol will be very good for the preterm babies given the parents' concerns for their health.

Data quality: Standardization of a large number of health professionals is always a major challenge for which we are prepared. Coordinating travel arrangements between centres to monitor data quality will need to be well organized.

Data analysis: Constructing growth curves is a sophisticated task for which several statistical methods are available; the experience gained in the WHO study is a major asset to overcome this challenge.

Sample size: Selecting an adequately powered sample size for studies like this one is always a compromise between statisticians' estimates, logistics and cost. We are concerned that we should not over-extend our study by attempting to answer too many questions. Ideally, we should have a sample size to create preterm standards with stable outer centiles and birth weight for gestational age standards with sufficient newborns with severe outcomes across the gestational age distribution. We would also like to explore ethnic-specific growth, in the event that multiple ethnic differences emerge from the data. However, based on the findings of the WHO Child Growth Study, we consider it unlikely that each ethnic group will have its own distinct growth pattern. Nevertheless, we shall explore this question by including women from each of the 5 ethnic groups: Whites, Latin Americans, African blacks, Orientals and Indians.

Motivation: The final challenge, but perhaps the most important, is to maintain motivation of the local investigators and data collection teams throughout the project, as they are the people who can guarantee the quality of the study.

Resources: Finally, we appreciate that advocating the introduction of new fetal growth standards into clinical practice in developing countries could be criticized on the grounds that it will require already under resourced institutions to purchase relatively expensive ultrasound equipment. We accept the possibility that some institutions will purchase ultrasound equipment based on the results of this study, despite our recommendation that serial ultrasound measurements are not necessary for the evaluation of fetal growth during routine antenatal care (10). However, many district level and referral hospitals in developing countries already have ultrasound machines and when they are used to evaluate high risk pregnancies and/or women with uncertain gestational age, the results may be misinterpreted or misused as the charts available are often inadequate. Therefore, much of this project is orientated towards more effective use of equipment that is already in such institutions.

Interestingly, the expensive and very sophisticated 3D imaging system being investigated in this study, for the first time on this scale, has potential to benefit high-risk pregnancies in rural areas and isolated communities. The rationale is that it will become feasible to transfer images from peripheral clinics to specialized centres for reconstruction and interpretation as the cost of the equipment falls and it becomes more portable. Such a strategy would reduce the transfer of large numbers of women unnecessarily as occurs at present and contribute to the decentralization of antenatal care services worldwide.

F: Regional distribution of Study Sites:

We have recruited study sites in the following regions of the world: Europe (UK); North America (USA); Central and South America (Brazil); Africa (Kenya); Arab World (Oman); Asia (China) and the India subcontinent (India).

G. Intervention Effectiveness for Impaired Fetal Growth

We ourselves have explored the question of what to do after IUGR is diagnosed and we have published a series of review papers critically evaluating RCT interventions for IUGR (47-50) and another group's recent update (51). There are not many interventions for treating (as opposed to preventing) IUGR, other than planned elective delivery, to which use of ultrasound contributes greatly in developing countries. This explains why referral to an adequate level of perinatal care is so important an option. Our study therefore focuses on helping clinicians to detect impaired fetal growth accurately, which should avoid incorrect diagnoses and, thereby, iatrogenic preterm births. The misuse of this technology is one of

For peer review only - http://bmjopen.bmj.com/site/about/guidelines.xhtml

the factors responsible for unnecessary medical interventions and why some treatments or interventions are shown to be ineffective, i.e. treatment of fetuses that may not have required it in the first place. Furthermore, in the light of information contained in the Lancet 2008 Nutrition Series and other similar recent reports, we will consider the possibility of implementing nested case-control studies to obtain information related to maternal body composition, metabolism and micronutrient status, or growth factors and birth outcomes. We will explore them in detail as this is a unique opportunity for such studies.

We think that interventions can only be effective if they focus on the factor responsible for the growth restriction. IUGR is a very heterogeneous condition, as we have shown, and it is unlikely that a "silver bullet" will resolve all IUGR (or preterm deliveries), even if correctly detected. Overall, we believe that accurate early identification, avoiding false positive cases, and appropriate referral to adequate levels of obstetric and newborn care will have a great impact on neonatal mortality and resource allocation in developing countries.

For peer review only

VIII. Monitoring, Evaluation, and Dissemination

A. Monitoring and Evaluation

Data collection will be monitored using routine procedures and protocols employed by our network in several similarly sized, multi-centre studies. The production and evaluation of specific standards will undergo a methodical and rigorous peer review process through the expert consultation mechanisms used in previous projects: the WHO Child Growth Study and the perinatal multi-centre studies conducted by our network. Monitoring the implementation process at the study sites will follow principles established in implementing previous, similarly complex, studies carried out by Consortium members. This will include site visits and on-going quality control measures (see Section IV).

B. Dissemination of Results

The generous participation of hundreds of scientists in all aspects of dissemination was the key to the success of the WHO Child Growth Study and we will therefore rely on their already established system. So, involving the Maternal Health Task Force at the Harvard School of Public Health in disseminating the results locally will be our principal strategy; this includes local and regional meetings and symposia. We will then focus on the local and global professional societies that are an integral part of the application, as well as health authorities in the participating countries and regional health institutions. Finally, a formal dissemination strategy similar to the one used by the WHO Child Growth Study will be implemented, including partnerships with other key players in the international field such as UNICEF, other UN agencies, NGOs and government institutions.

For peer review only

IX. Optimizing Public Health Outcomes and Intellectual Property Plans to Achieve Global Access

It is expected that these international standards will be used to assess the growth of fetuses and newborns throughout the world. The basic assumption behind the proposed standards is that fetal growth is optimal in healthy populations. Therefore, the proposed curves will constitute "optimal" standards. Widespread uptake of these fetal curves will be ensured by ultrasound equipment manufacturers incorporating them into their systems. The potential uses are:

1) Population assessment to a) provide a reference for comparison of the means (or medians), standard deviations, and trajectories of the population means (or medians) for any given sample, b) estimate the proportions of fetuses-newborns below a given cut-off in a sample, and c) standardize for gestational age and sex differences between samples, thereby allowing comparisons of prevalence rates below a given cut-off across samples.

2) Individual assessment to a) screen for fetal growth on a single occasion to identify fetuses with excess or deficient growth leading to appropriate diagnostic and/or therapeutic interventions (including delivery), and b) provide a clinical tool for assessing the efficacy of treatments or helping to choose time of delivery, particularly in severely growth restricted fetuses. Hence, the primary use of the curves at an individual level will be to identify unacceptable deviations as early as possible to prevent severe deficits and excesses, although they will need to be introduced into clinical practice with appropriate care and auditing. We expect to link with, and build upon, WHO's global efforts to disseminate the child growth charts. The large network of proposed partners and consortium members should prove, with the help of industry, to be a very effective method for disseminating the results at both clinical and public health levels.

A. Intellectual Property (IP) Plan.

	Yes	*No*
1. Is the proposed research likely to lead to any patentable or commercially exploitable results?		X
The study will provide each centre with a sophisticated (commercially available) 3D ultrasound machine, specially adapted for the needs of the study, as well as technical expertise for data transfer and storage of 2D and 3D data. The proposed research is unlikely to lead to patentable results. Ultrasound machine manufacturers throughout the world will be encouraged as part of the Dissemination and Implementation Plan to incorporate the new standards into their machines from which they may derive commercial benefit.		

2. Will the proposed project, either at its inception or at a foreseeable future point, depend on the use of technologies, materials, or other inventions that may conflict with goals of global access in terms of either cost or availability in the developing world?		X

3. Is the proposed project and related IP subject to any agreements (e.g., licenses, collaborations, research or funding agreements or any other form of agreement) with commercial, academic, or other organizations, including other funding entities, subgrantees or subcontractors?		X

4. Does your organization plan to assume responsibility for maturation, production, and dissemination of the innovation itself?	X	

For peer review only - http://bmjopen.bmj.com/site/about/guidelines.xhtml

B. Commitment to Sharing Data and Materials

It is highly unlikely that patentable IP will be generated by this project. The growth standard and related analyses represent the new evidence-based knowledge that will be generated. These will be widely disseminated, especially in open access publications, and shared with research and clinical communities worldwide, as well as the appropriate national and international agencies.

The Fetal Growth Standards charts will be made available to all manufacturers of ultrasound machines. Data from each centre will be provided to the local investigators with the understanding that the primary and secondary objectives of this study can only be explored with the pooled data set and can not be replicated using local data. Other locally relevant issues can be analyzed by individual centres.

The study Steering Committee (SC) and Executive Committee (EC) will also, after the main tasks are completed, engage in negotiations with other *bona fide* researchers for access to the data set to allow scientific and public health relevant questions to be explored. The SC and the EC have developed a set of rules and procedures for the evaluation of such requests in keeping with the commitment of the present investigators to ensure the widest possible utilization of the data (see Appendix F).

C. How will we bridge the new data to the existing data?

We aim to produce the following new standards:

1. **Fetal growth by ultrasound:** Based on discussions with ultrasound manufacturers and the known limitations of existing charts, we anticipate that the newly developed fetal growth charts will replace all charts that are currently incorporated into ultrasound machines.

2. **Postnatal growth for preterm babies:** Based on discussions with a number of pediatricians in different countries and WHO information, we anticipate that the postnatal growth charts for preterm babies will be incorporated into routine clinical practice without much difficulty because there is a lack of well-developed charts for this specific purpose. We believe that the international community is looking forward to such charts being developed.

3. **Birth weight for gestational age standards:** It is likely that the new, international, risk-related, birth weight for gestational age standards will be widely adopted following implementation of the new fetal growth and preterm postnatal growth standards. However, we acknowledge that this process may take longer because other standards are already being used (even though their limitations are recognized). We shall therefore work closely with the Maternal Health Task Force at the Harvard School of Public Health to follow the strategy they are using for the infant growth standards.

We do not anticipate any conceptual limitations to the future use of the standards, particularly in the light of the statement in the recently published Lancet "Maternal and Child Undernutrition" series that international fetal and newborn growth standards need to be developed (5). Nevertheless, it is clear that whenever efforts are being made to change clinical practice (even if the need to do so is recognized), common barriers and resistance to change will be encountered. We feel that our experience in introducing the MGRS standards will be invaluable for this task although, in theory at least, introducing the ultrasound standards should present fewer problems as they will be built into the equipment and we hope they will be endorsed by the International Society of Ultrasound in Obstetrics & Gynecology and other professional associations responsible for recommending new technology into clinical practice.

Rolling out the fundal-height charts and the newborn and preterm growth standards will use the same procedures as WHO did when it rolled out its Child Growth Standards. We shall coordinate these processes from the start of INTERGROWTH-21st by liaising closely with both the Maternal Health Task Force at the Harvard School of Public Health and the leading manufacturers of ultrasound machines. The network already developed by the team will serve as a means of rolling out the standards we propose developing in INTERGROWTH-21st.

For peer review only - http://bmjopen.bmj.com/site/about/guidelines.xhtml

X. Organizational Capacity and Management Plan

A. Organizational Capacity and Facilities

History: The University of Oxford enjoys an international reputation as a world-class centre of excellence in research and teaching. It employs over 7,800 academic, research and support staff across a wide range of academic disciplines. The Medical Sciences Division, within which the Nuffield Department of Obstetrics & Gynaecology (NDOG) is located, is one of the major centres for clinical and basic biomedical research in Europe, with more than 2,200 staff and 800 postgraduate students. It achieved top scores in both the 1996 and 2001 UK HEFCE research assessment exercises, and fosters the highest possible standards in research, teaching and patient care. The annual grant income from external sources for the Medical Sciences Division is approximately $200M.

Experience: The Consortium members (University of Oxford and collaborating centres) have extensive experience in conducting multi-centre studies and constitute perhaps the largest research network in the world in the field of women's and perinatal health. NDOG has coordinated large-scale genetic epidemiology studies across multiple international sites, involving data collection from thousands of individuals in the ENDOGENE Study. Office space within its existing facilities at the John Radcliffe Hospital, Oxford, will be provided to house the Coordinating Unit (CU) staff, as well as the necessary IT support and administrative assistance. Sites have been chosen on the understanding that they have the necessary infrastructure to conduct the study (e.g. IT and capacity for ultrasound research). Finally, the professional societies recruited to help coordinate implementation and dissemination of the results are among the leading groups in the world in the field of obstetrics.

B. Management and Staffing Plan

Management: The study will be coordinated and managed by NDOG (University of Oxford), where the CU will be located (see Appendix F). Four units responsible for day-to-day monitoring of quality control and data collection (2D Ultrasound Data Quality Unit; Anthropometric Data Quality Control Unit; Data Management Unit and 3D Ultrasound Data Quality Unit) will report directly to the CU. All data, except the 3D data, will be centralized at the Data Management Unit.

The Steering Committee (SC) consists of representatives of Consortium members and the lead investigator from each of the study centres. It has also external members to provide support in areas related to the study. The SC will meet twice a year to review progress, ensure uniformity of data collection from the study sites, and discuss any substantive issues that arise. Any adaptations to the protocol or technical variations required at individual sites because of local needs will be reviewed and approved by the SC.

The Executive Committee (EC) will monitor the progress of the study on a regular basis and resolve substantive issues arising from implementation of the study. The Study Coordinator will be ex-officio member of the EC. The EC will make the final selection of study sites; exclude a site if data collection standards are not sufficiently high, and make final decisions about sample selection and analytical issues related to construction of the standards.

The AC, consisting of internationally recognized experts in ultrasound, obstetrics, statistics, nutrition, fetal development, anthropometrics, epidemiology and biology, will provide technical advice to the CU, EC and SC. The study will be indemnified by the University of Oxford that will carry liability for any adverse outcomes arising from breaches of research protocol.

Study coordination:

The **Principal Investigator** (José Villar) will be responsible for all scientific and technical components of the project. The **Project Director** (Stephen Kennedy) will be the focal person in NDOG responsible for overall study management. The **Project Leader** (Leila Cheikh Ismail), will be responsible for day-to-day implementation of the study, coordinating and monitoring of all activities as well as training, monitoring and evaluation. A **Statistical**

For peer review only - http://bmjopen.bmj.com/site/about/guidelines.xhtml

Director (Doug Altman) will advise on sampling strategies, sample sizes and all statistical methods, in particular those for the construction of the new standards. He will be assisted in Years 1-5 by a **Data Management Supervisor** (Stephen Ash), who will coordinate all aspects of data management between the Data Management Unit (DMU), the CU in Oxford and the other data quality units. This will include the preparation of data sets, quality control strategies and data files for use by the team of statisticians. This strategy for data management and analysis has been extremely efficient in previous, large multi-centre studies conducted by us as it allows for direct contact between the data collection centres and the coordinating unit. In Years 3-5, Dr Altman will also be assisted by a **Senior Statistician** to develop, test and implement computer programs to construct the new growth standards.

There will be a **Senior Technical Coordinator** (Aris Papageorghiou, St George's, London) of the ultrasound component of the study, who will have responsibility for overall coordination of the ultrasound measurements, including quality control and standardization. He will be assisted by an **External Ultrasound Expert** (Laurant Salomon), acting as a consultant and a **Clinical Research Fellow** (Caroline Knight) to oversee the quality of a random sample of ultrasound measurements during the data collection period. The CU team will be completed by a **Secretary** (Melissa Shorten) responsible for secretarial support to the CU, a **Project Administrator** (Ann Lambert) in charge of all aspects of transferring funds to the study centres, equipment, meetings, travel and coordination between the CU, DMU and study centres, and a **Research Associate** (Isabelle Wilson) responsible for piloting protocol and monitoring its implementation.

For peer review only

XI. Citations

1. Lawn JE, Cousens S, Zupan J. 4 million neonatal deaths: when? Where? Why? Lancet 2005;365:891-900.
2. Williams RL, Creasy RK, Cunningham GC, Hawes WE, Norris FD, Tashiro M. Fetal growth and perinatal viability in California. Obstet Gynecol 1982;59:624-32.
3. Rao SC, Tompkins J. Growth curves for preterm infants. Early Hum Dev 2007;83:643-51.
4. de Onis M, Habicht JP. Anthropometric reference data for international use: recommendations from a World Health Organization Expert Committee. Am J Clin Nutr 1996;64:650-8.
5. Black RE, Allen LH, Bhutta ZA, Caulfield LE, de Onis M, Ezzati M et al. Maternal and child undernutrition: global and regional exposures and health consequences. Lancet 2008;371:243-60.
6. de Onis M, Onyango AW, Van den Broeck J, Chumlea WC, Martorell R. Measurement and standardization protocols for anthropometry used in the construction of a new international growth reference. Food Nutr Bull 2004;25:S27-36.
7. Kramer MS, Platt RW, Wen SW, Joseph KS, Allen A, Abrahamowicz M et al. A new and improved population-based Canadian reference for birth weight for gestational age. Pediatrics 2001;108:E35.
8. de Onis M, Garza C, Onyango AW, Martorell R. WHO Child Growth Standards. Acta Paediatr 2006;450:1-101.
9. Zhang X, Platt RW, Cnattingius S, Joseph KS, Kramer MS. The use of customised versus population-based birthweight standards in predicting perinatal mortality. BJOG 2007;114:474-7.
10. Villar J, Ba'aqeel H, Piaggio G, Lumbiganon P, Miguel Belizan J, Farnot U et al. WHO antenatal care randomised trial for the evaluation of a new model of routine antenatal care. Lancet 2001;357:1551-64.
11. Kelly Y, Panico L, Bartley M, Marmot M, Nazroo J, Sacker A. Why does birthweight vary among ethnic groups in the UK? Findings from the Millennium Cohort Study. J Public Health 2008;epub Jul 21.
12. Villar J, Valladares E, Wojdyla D, Zavaleta N, Carroli G, Velazco A et al. Caesarean delivery rates and pregnancy outcomes: the 2005 WHO global survey on maternal and perinatal health in Latin America. Lancet 2006;367:1819-29.
13. Villar J, Abdel-Aleem H, Merialdi M, Mathai M, Ali MM, Zavaleta N et al. World Health Organization randomized trial of calcium supplementation among low calcium intake pregnant women. Am J Obstet Gynecol 2006;194:639-49.
14. Gulmezoglu AM, Villar J, Ngoc NT, Piaggio G, Carroli G, Adetoro L et al. WHO multicentre randomised trial of misoprostol in the management of the third stage of labour. Lancet 2001;358:689-95.
15. Royston P. Constructing time-specific reference ranges. Stat Med 1991;10:675-90.
16. Kramer M, Victora CG. Low birth weight and perinatal mortality. In: Nutrition and Health in Developing Countries. Totowa, NJ: Humana Press, 2001:57-69.
17. Carroli G, Rooney C, Villar J. How effective is antenatal care in preventing maternal mortality and serious morbidity? An overview of the evidence. Paediatr Perinat Epidemiol 2001;15 Suppl 1:1-42.
18. Villar J, Carroli G, Zavaleta N, Donner A, Wojdyla D, Faundes A et al. Maternal and neonatal individual risks and benefits associated with caesarean delivery: multicentre prospective study. BMJ 2007;335:1025.
19. Merchant KM, Villar J. Maternal height and newborn size relative to risk of intrapartum caesarean delivery and perinatal distress. BJOG 2001;108:689-96
20. Goedhart G, van Eijsden M, van der Wal M, Bonsel G. Ethnic differences in term birthweight: the role of constitutional and environmental factors. Paediat Perinat Epidemiol 22:4:360-368
21. Mortensen LH, Diderichsen F, Arntzen A, Gissler M, Cnattingius S, Schnor O et al. Social inequality in fetal growth: a comparative study of Denmark, Finland, Norway and Sweden in the period 1981-2000. J Epidemiol Community Health 2008;62:325-31.

For peer review only - http://bmjopen.bmj.com/site/about/guidelines.xhtml

22. Lynch CD, Zhang J. The research implications of the selection of a gestational age estimation method. Paediatr Perinat Epidemiol 2007;21 Suppl 2:86-96.
23. Robinson HP, Fleming JE. A critical evaluation of sonar "crown-rump length" measurements. Br J Obstet Gynaecol 1975;82:702-10.
24. Group MGRS, de Onis M. Complementary feeding in the WHO Multicentre Growth Reference Study. Acta Paediatr Suppl 2006;450:27-37.
25. Neufeld LM, Haas JD, Grajeda R, Martorell R. Ultrasound measurement of fetal size in rural Guatemala. Int J Gynaecol Obstet 2004;84:220-8.
26. Blake KV, Gurrin LC, Beilin LJ, Stanley FJ, Kendall GE, Landau LI et al. Prenatal ultrasound biometry related to subsequent blood pressure in childhood. J Epidemiol Community Health 2002;56:713-8.
27. British Ultrasoud Society. Fetal Measurements Document, 2008.
28. Verburg BO, Steegers EAP, De Ridder M, Snijders RJM, Smith E, Hofman A, Moll, HA, Jaddoe VWV, Wittema JCM. New charts for ultrasound dating of pregnancy and assessment of fetal growth: longitudinal data from a population-based cohort study. Ultrasound Obstet Gynae:31:388
29. Thorsell M, Kaijser M, Almstrom H, Andolf E. Expected day of delivery from ultrasound dating versus last menstrual period--obstetric outcome when dates mismatch. BJOG 2008;115:585-9.
30. Platt LD. Three-dimensional ultrasound, 2000. Ultrasound Obstet Gynecol 2000;16:295-8.
31. Belizan JM, Villar J, Nardin JC, Malamud J, De Vicurna LS. Diagnosis of intrauterine growth retardation by a simple clinical method: measurement of uterine height. Am J Obstet Gynecol 1978;131:643-6.
32. Khan NZ, Muslima H, Begum D, Shilpi AB, Akhter S, Bilkis K, et al. Validation of Rapid Neurodevelopmental Assessment Instrument for Under-Two-Year-Old Children in Bangladesh. Pediatrics. 2010; **125**(4): e755-e62.
33. Adoh TO, Woodhouse JM. The Cardiff acuity test used for measuring visual acuity development in toddlers. Vision Research. 1994; **34**(4): 555-60.
34. Kihara M, Hogan AM, Newton CR, Garrashi HH, Neville BR, de Haan M. Auditory and visual novelty processing in normally-developing Kenyan children. Clinical Neurophysiology. 2010; **121**(4): 564-76.
35. So K, Michael Adamson T, Horne RSC. The use of actigraphy for assessment of the development of sleep/wake patterns in infants during the first 12 months of life. Journal of Sleep Research. 2007; **16**(2): 181-7.
36. McGuire W, Henderson G, Fowlie PW. Feeding the preterm infant. BMJ 2004;329:1227
37. Borghi E, de Onis M, Garza C, Van den Broeck J, Frongillo EA, Grummer-Strawn L et al. Construction of the World Health Organization child growth standards: selection of methods for attained growth curves. Stat Med 2006;25:247-65.
38. Bhandari N, Bahl R, Taneja S, de Onis M, Bhan MK. Growth performance of affluent Indian children is similar to that in developed countries. Bull World Health Organ 2002;80:189-95.
39. Owusu WB, Lartey A, de Onis M, Onyango AW, Frongillo EA. Factors associated with unconstrained growth among affluent Ghanaian children. Acta Paed 2004;93:1115-9.
40. Villar J, Abalos E, Carroli G, Giordano D, Wojdyla D, Piaggio G et al. Heterogeneity of perinatal outcomes in the preterm delivery syndrome. Obstet Gynecol 2004;104:78-87.
41. Kramer MS, Ananth CV, Platt RW, Joseph KS. US Black vs White disparities in foetal growth: physiological or pathological? Int J Epidemiol 2006;35:1187-95.
42. Zhang J, Villar J, Sun W, Merialdi M, Abdel-Aleem H, Mathai M et al. Blood pressure dynamics during pregnancy and spontaneous preterm birth. Am J Obstet Gynecol 2007;197:162 e1-6.
43. Villar J, Carroli G, Wojdyla D, Abalos E, Giordano D, Ba'aqeel H et al. Preeclampsia, gestational hypertension and intrauterine growth restriction, related or independent conditions? Am J Obstet Gynecol 2006;194:921-31.
44. Chang CH, Yu CH, Ko HC, Chen CL, Chang FM. Predicting fetal growth restriction with liver volume by three-dimensional ultrasound: efficacy evaluation. Ultrasound Med Biol 2006;32:13-7.

For peer review only - http://bmjopen.bmj.com/site/about/guidelines.xhtml

45. Huang H, Zhang J, Wakana S, Zhang W, Ren T, Richards LJ et al. White and gray matter development in human fetal, newborn and pediatric brains. Neuroimage 2006;33:27-
46. Cooper C. Epidemiology of osteoporotic fracture: looking to the future. Rheumatology (Oxford) 2005;44 Suppl 4:iv36-iv40.
47. Villar J, Gulmezoglu AM, de Onis M. Nutritional and antimicrobial interventions to prevent preterm birth: an overview of randomized controlled trials. Obstet Gynecol Surv 1998;53:575-85.
48. Gulmezoglu M, de Onis M, Villar J. Effectiveness of interventions to prevent or treat impaired fetal growth. Obstet Gynecol Surv 1997;52:139-49.
49. de Onis M, Villar J, Gulmezoglu M. Nutritional interventions to prevent intrauterine growth retardation: evidence from randomized controlled trials. Eur J Clin Nutr 1998;52 Suppl 1:S83-93.
50. Kulier R, de Onis M, Gulmezoglu AM, Villar J. Nutritional interventions for the prevention of maternal morbidity. Int J Gynaecol Obstet 1998;63:231-46.
51. Iams JD, Romero R, Culhane JF, Goldenberg RL. Primary, secondary, and tertiary interventions to reduce the morbidity and mortality of preterm birth. Lancet 2008;371:164-75.

XII. Appendices

Appendix A: Data Collection Forms
Three different types of data recording forms will be used:

FGLS: 1) Screening: Interview
2) Screening: Ultrasound Dating
3) Maternal Study Entry
4) Ultrasound Follow-up
5) Pregnancy Follow-up
6) Pregnancy and Delivery (including newborn anthropometrics)
7) Referral/Admission
8) Adverse Events
9) Fetal and Neonatal Abnormality

PPFS: 1) Preterm Follow-up
2) Preterm End of Study
3) Preterm Referral/Admission

NCSS: 1) Pregnancy and Delivery (including newborn anthropometrics) - same as FGLS

It should be emphasized that all forms are as short as possible to improve compliance. Therefore, all questions have been carefully evaluated to ensure that they serve at least one of the following purposes: a) eligibility (e.g. socio-economic status); b) sample description (demographic and environmental variables, etc.); c) exclusion criteria (major illnesses, etc.); d) standardization of results across centres; e) future use of references (vitamin/mineral supplements), or f) to assess possible selection biases.

For peer review only

Appendix B: Data Flow Models
FETAL GROWTH LONGITUDINAL STUDY DATA FLOW

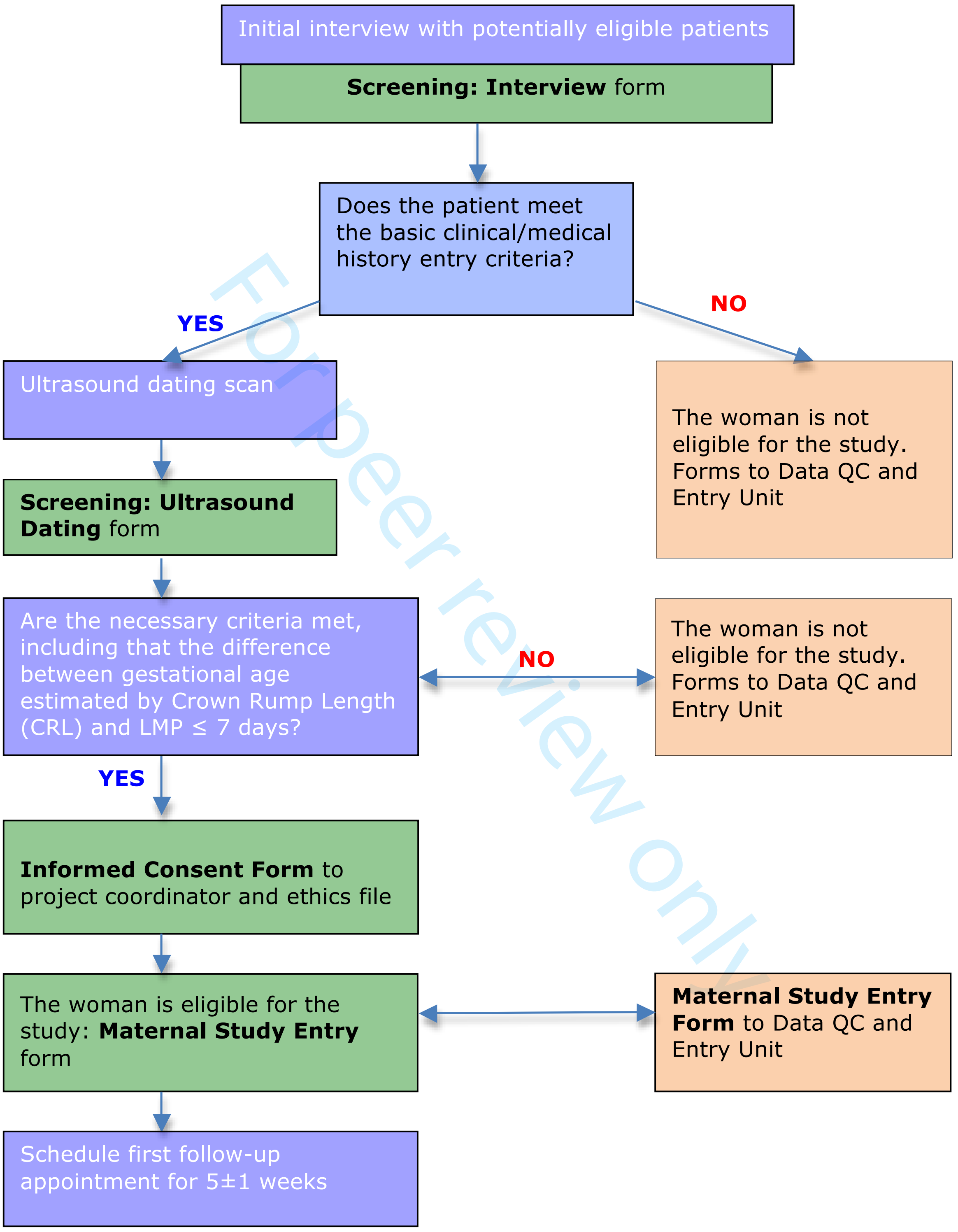

FETAL GROWTH LONGITUDINAL STUDY DATA FLOW (CONT.)

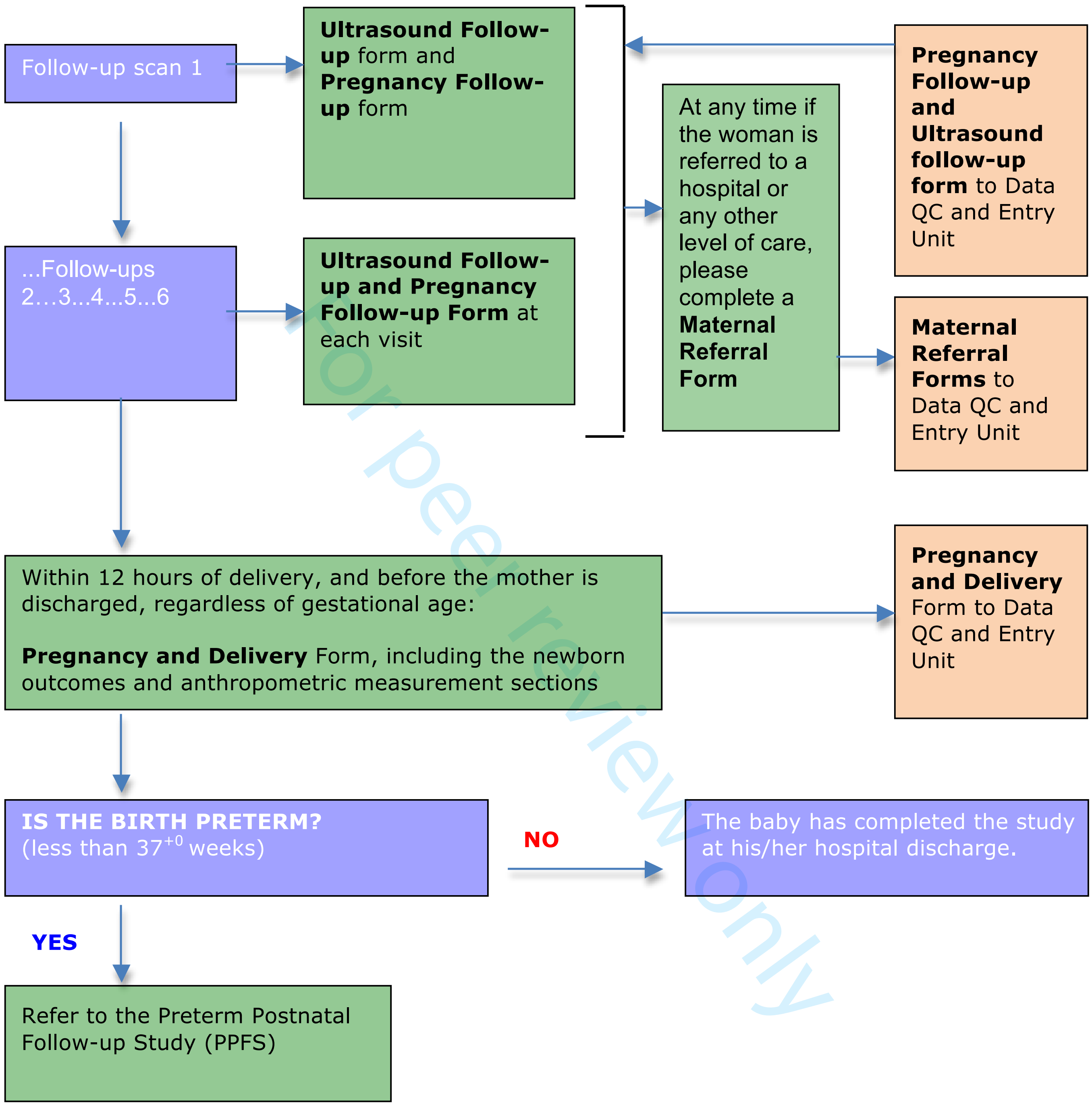

PRETERM POSTNATAL FOLLOW-UP STUDY DATA FLOW

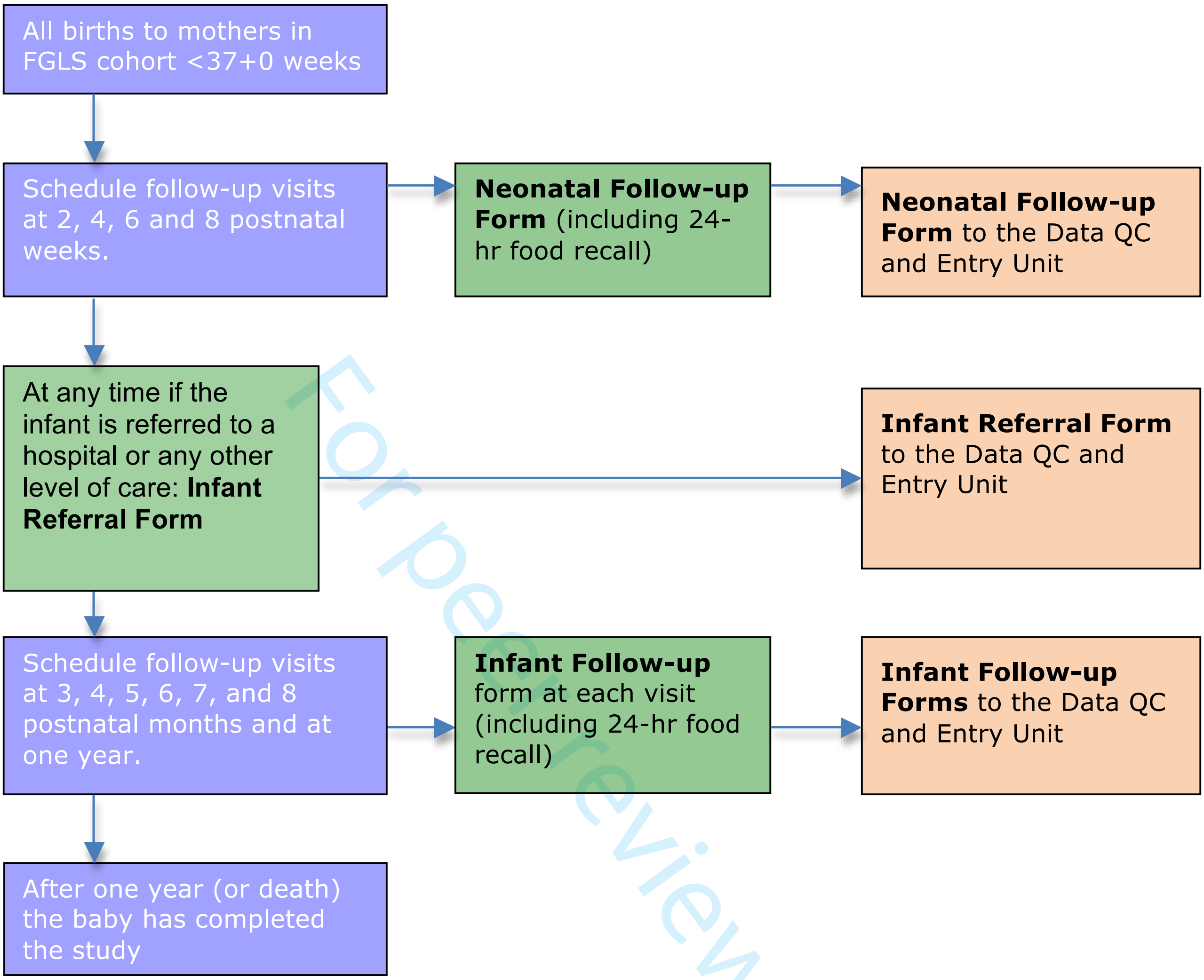

For peer review only

Appendix C: Ultrasound Methods And Quality Control

Acknowledgements: The first version of the ultrasound protocol was prepared between 18th June and 10th July 2008 by Aris Papageorghiou and Laurent Salomon, with valuable input from Jan van den Broeck. Aris Papageorghiou revised the protocol based on feedback from the INTERGROWTH-21st Executive Committee and Paul Chamberlain. Further amendments were then made based on a series of exchanges between Aris Papageorghiou and Laurent Salomon, Jan van den Broeck (quality control), Paul Chamberlain, José Villar and Stephen Kennedy. The protocol was finalized after extensive discussion at the initial meeting of the Steering Committee, which took place in Oxford on 15-17th September 2008, and finally approved in the Steering Committee Meeting in Oxford, 25-27th March 2009.

1. Background

A central plank of INTERGROWTH-21st is the creation of fetal growth charts during the FGLS part of the study. The correct use of ultrasound is essential to ensure that accurate, reproducible and applicable results are obtained. This document sets out all the ultrasound requirements for the study, including measurements, training, equipment and quality control.

2.1 Initial ultrasound examination and dating:

All mothers will have a trans-abdominal ultrasound scan between 9^{+0} to 14^{+0} weeks. This will be performed to rule out ectopic pregnancy, missed abortion and multiple gestations and to estimate gestational age (GA).

For the purposes of the study, gestational age will be calculated by LMP and confirmed by fetal crown-rump length (CRL). In order to allow accurate assessment of gestation the inclusion criteria used were:

- Certain LMP
- Regular 26-30 day menstrual cycles in the last 3 months;
- No hormonal contraception use, pregnancy or breastfeeding in the 3 months preceding the LMP.
- As embryonic growth may be different in pregnancies from assisted reproductive techniques[2,3] only those with spontaneous conception were included
- The CRL will be plotted on well-established charts [4,5]. If the difference in gestational age estimation by CRL and LMP is ≤ 7 days we will consider the LMP to be reliable and take it as the true biological date. Women where the difference in gestational age between LMP and CRL is greater than 7 days will not be eligible.

2.2 Subsequent ultrasound examinations

Following the initial scan, women will have up to six scheduled scans at ~5 weekly (±1 week) intervals until delivery. When rescanning does not occur within the allotted time, the women will be asked to attend at their next scheduled study appointment.

Essential documentation

At all examinations after dating the following will be documented:

1. Fetal presentation (cephalic, Breech, Transverse, Oblique)
2. Placental localization (fundal, high anterior, high posterior, high right lateral, high left lateral, low anterior, low posterior, low right lateral, low left lateral).
3. Amniotic fluid volume (polyhydramnios, increased, normal, reduced, oligohydramnios, anhydramnios).

For peer review only - http://bmjopen.bmj.com/site/about/guidelines.xhtml

Essential measurements (see* 3. Methodology of ultrasound and definitions*)

At all examinations after dating the following measurements will be taken:

1. Biparietal diameter (BPD)
2. Occipito-Frontal Diameter (OFD)
3. Head circumference (HC) using the ellipse facility
4. Transverse abdominal diameter (TAD)
5. Antero-posterior abdominal diameter (APAD)
6. Abdominal circumference (AC) using the ellipse facility
7. Femur length (FL)

Optional measurements

This study presents a unique opportunity to obtain other measurements of fetal biometry. However, the introduction of extra tasks could reduce the overall quality of the seven main measurements and inconvenience the participants. Therefore, the feasibility of taking additional measurements will be evaluated in consultation with each centre before a final decision is made and only some centres are expected to participate in these additional studies. In descending order of importance:

1. Humerus
2. Radius / Ulna
3. Tibia / Fibula

2.5 3-Dimensional Ultrasound Volumes (3D ultrasound)

Essential 3D measurements

The objective of the 3D component is to provide volumetric data to assess the quality of the 2D measurements (BPD, OFD, HC, AC, FL). Hence, the 3D volumes that we obtain will be of the:

(1) head (capture 3D volume with 2D view at the level of the BPD)
(2) abdomen (volume at the level of the AC)
(3) femur (volume in the same view as measuring the FL)

Optional 3D measurements

In order to establish a unique “biological databank” for future research additional 3D volumes of other organs, e.g. the heart at the level of the 4-chamber view of the heart; placental volumes, can be taken specifically for these purposes. These will be considered ancillary studies and will be dependent upon local capacity and the approval of the Steering Committee.

We plan to take the final decision regarding the amount of 3D data to be collected and the number of participating sites for these ancillary studies after conducting a pilot study assessing the human resources available at the centres, the need for additional training, time that 3D scanning adds to each visit, equipment considerations and the feasibility of transferring the very large datasets to the Coordinating Unit in Oxford.

Issues that we will explore in the pilot study include:

a) difficulties obtaining liver, brain and fetal cerebellar volumes
b) the need to obtain at least two good volume measures from the areas of interest, which may require 20-30 mins additional scanning
c) the temptation to divert attention away from standard measurements to more “exciting new technology” which will be popular with women. For these purposes the surface rendering function will be disabled.
d) how to save and transmit the volumetric data reliably given the risks of a hard drive crashing or being corrupted.
e) With advancing gestation (especially in the third trimester, it is likely that we will not be able to record many of the 5 volumes listed above accurately (i.e. brain and abdomen).

For peer review only

3. Methodology of ultrasound and definitions

The first visit (dating scan) will be between 9^{+0} and 14^{+0} completed weeks. The aim of this scan is to confirm fetal viability, and to exclude multiple pregnancy or major fetal abnormality. Fetal CRL is measured at this visit. In women who meet the inclusion criteria and where the discrepancy between gestational age by LMP and CRL is ≤7 days, subsequent visits (for fetal biometry) will be scheduled at approximately 5 weekly (± 1 week) intervals (i.e. 14-18, 19-23, 24-28, 29-33, and 34-38 weeks and 39-42 weeks).

A full morphological evaluation will be conducted at 19-23 weeks following standard practice at each centre. Fetuses diagnosed with any minor abnormalities will be managed as per local clinical guidelines. If the clinical decision is to continue with the pregnancy the mother will remain in the study. Study follow-up for these cases will be identical, but eventual exclusion at the time of data analysis will be carried out.

Fetuses with major abnormalities that may affect morphometric measurements will be excluded from further study. All infants will receive anthropometrical assessment after delivery

3.1 Basic characteristics

1. Transabdominal ultrasound
2. Lateral recumbent position.
3. Essential ultrasound measurements are obtained at all visits
    - Biparietal diameter (BPD)
    - Occipito-Frontal Diameter (OFD)
    - Calculated Head circumference (HC) using the ellipse facility
    - Transverse abdominal diameter (TAD)
    - Anterio-posterior abdominal diameter (APAD)
    - Calculated abdominal circumference (AC) using the ellipse facility
    - Femur length (FL)
4. Measurements are obtained 3 times from 3 separately generated ultrasound images of each structure in a blinded fashion (no measurement visible) and submitted electronically.
5. Following this the final set of measurements are revealed for clinical management purposes as per local protocols.
6. In each view a single 3D volume is stored for quality control purposes.
7. Ultrasound equipment provides measurements to tenth of a millimetre.
8. Data are submitted electronically to the web-based data set
9. Depending on the availability of appropriate technology, associated images are also submitted electronically to the Coordinating Unit. If real time submission of images is not available these will be down-loaded onto a memory card and submitted via email.
10. Ultrasound images of CRL, BPD, OFD, HC, AC and FL must fill at least 30% of the monitor screen.

3.2 Measurement techniques for compulsory measurements [4-10]

Crown – Rump Length (CRL) – only at the dating scan (9^{+0} to 14^{+0} weeks)

Obtaining the image

- A mid-sagittal section of the fetus should be obtained
- The fetus should be horizontal (at 90° to the angle of insonation)
- The fetus should be in a neutral position (not hyperextended or flexed)

Magnification

- The image must fill at least 30% of the monitor screen.

For peer review only - http://bmjopen.bmj.com/site/about/guidelines.xhtml

Caliper placement

- The intersection of the calipers should be placed on the outer borders of the head and rump.

Biparietal Diameter

Obtaining the image

- A cross-sectional view of the fetal head at the level of the thalami
- As close as possible to the horizontal with the angle of insonation as close as possible to 90°
- Oval shape
- Symmetrical
- Centrally positioned, continuous midline echo (falx cerebri) broken anteriorly at one third of its length by the cavum septum pellucidum
- The thalami should be located symmetrically on each side of the midline falx.

Magnification

- The cross section of the fetal head must fill at least 30% of the monitor.

Caliper placement

- The intersection of the calipers should be placed on the outer border of the parietal bones ('outer to outer') at the widest part of the skull.

Occipito-frontal diameter (OFD) and Head Circumference (HC)

Obtaining the image and magnification: obtained from the same still image as the BPD.

Caliper placement

The intersection of the calipers should be placed on the outer border of the occipital and frontal edges of the skull at the point of the midline ('outer to outer') across the longest part of the skull. The HC will be calculated from the BPD and OFD measurements using the ellipse facility (using the formula HC = π(BPD + OFD)/2).

Abdominal Circumference

Obtaining the image

- Transverse section of the fetal abdomen as close as possible to circular
- Umbilical vein in its anterior third
- Stomach bubble visible
- Kidneys and bladder not visible

Magnification

- The cross section of the fetal abdomen must fill at least 30% of the monitor screen.

Caliper placement

- The anterior-posterior abdominal diameter (APAD) and transverse abdominal diameters (TAD) are measured.
- To measure the APAD the intersection of the calipers is placed on the outer borders of the body outline from the posterior aspect (skin covering the spine) to the anterior abdominal wall.
- To measure the TAD the intersection of the calipers is placed on the outer borders of the body outline at 90° to the APAD, across the abdomen at the widest point.
- The AC will be calculated from the APAD and TAD measurements using the ellipse facility (based on the formula AC = π(APAD + TAD)/2).

Femur Length Technique

Obtaining the image

- To be imaged as close as possible to the horizontal plane.
- Angle of insonation of the ultrasound beam is 90°.

For peer review only

- The full length of the bone is visualised
- Not obscured by shadowing from adjacent bony parts.

Magnification

- The cross section of the fetal abdomen must fill at least 30% of the monitor.

Caliper placement

- The intersection of the calipers is placed on the outer borders of the edges of the femoral bone 'outer to outer').
- The trochanter is not to be measured.

3.3 Techniques for optional measurements:
The humerus, radius, ulna, tibia and fibula can all be measured from 14^{+0} weeks onwards. Similar to the guidelines of femur length measurement, the longest length of each bone is measured with the bone at as close as possible to 90° to the ultrasound beam. The humerus is measured from upper to lower lateral margins. In the forearm the ulna is distinguished from the radius by its longer proximal length (both radius and ulna end at the same level distally). In the lower leg the lateral bone is the fibula with the tibia lying medially to it. All long bones are to be measured along their longest lengths from upper to lower lateral margins. The bone measured should fill at least 30% of the width of the screen. **Only one side (the anterior or most easily accessible limb) should be measured.**

3.4 Inability to take measurements
The fetal position may on occasion be so persistently unfavourable as not to allow measurements according to the guidelines above.

CRL: Accurate measurement of CRL is of obvious importance in ensuring that a woman is eligible to take part in the study. If despite repeated attempts it is not possible to obtain a CRL measurement that allows confirmation of gestational age the woman is not eligible for the study (in the same way she will not be eligible for the study if the gestation calculated from CRL and LMP is discrepant by more than 7 days).

Essential measurements: With the exception of CRL, every effort should be made to obtain the best possible measurements taking into account the definitions above. This may require allowing the woman to go and return for the scan at a later date/time (within one week). Measurements should not be taken if it remains impossible to obtain a good quality image to allow accurate measurement this should not be taken. The next appointment should be kept as scheduled.

Optional measurements: The same principle applies to the optional measurements: these should not be taken if it is impossible to obtain a good quality image.

4. Equipment (based on RCR recommendations)[13]:
In all applications of ultrasound, three things are of prime importance: image clarity, resolution and the ability to differentiate tissue structures. Hence, although it is accepted that the best possible images may not be obtained from all patients at all times, the equipment chosen must be capable of visualizing tissue structures in the overwhelming majority of patients with different body sizes and shapes to a level sufficient to meet the exacting needs of the study.

The specifications below are the minimum requirements for the study, but it is important to recognize that image clarity and resolution may differ between manufacturers despite machines having similar specifications. It is therefore essential that candidate machines are tested before a final choice is made to assess their quality and to determine operator preferences. It is envisaged that this will be done at the Study Coordinating Unit (Oxford) or the Ultrasound Coordinating Unit (St George¹s, London).

For peer review only - http://bmjopen.bmj.com/site/about/guidelines.xhtml

We realize that there is a potential trade-off between quality and cost and, therefore, both these factors need to be considered in a balanced way in making a decision.

Study specific considerations

1. All ultrasound equipment to be used will be standardized
   - Uniform probes
   - Uniform factory presets ("study preset")
2. Commercially available high quality real-time ultrasound scanner.
3. Less than 2 years old.
4. Transabdominal probes suitable for scanning throughout pregnancy.
5. Facility for on-line transfer of measurements and associated images.
6. Facility to "blind" measurements from examiner until after data transfer.
7. Facility to "unblind" the final measurement to allow clinical use.
8. Equipment will be serviced periodically by specialized technicians as per the manufacturer's recommendations.

Operation/ Display

General requirements:

- Operating from a single phase standard domestic 240V, 13A, 50 Hz mains supply (110V in Cuba)
- Large single high resolution non-interlaced monitor (min.15")
- Display examination details
  Basic patient ID
  date & time
  examination centre
  selected probe
  acoustic power setting
  mechanical and thermal indices and other image processing and Doppler information to be shown in all display modes on all video/disk/hardcopy outputs
- Floating keyboard and monitor with easy to move console
- Single and dual display of the modes should be available simultaneously
- High definition variable size display magnification
- Digital Processing Channels Beamformer
- Digital display memory
- Large Dynamic Range
- Automated 'Tissue Specific' pre-sets
- Automated 'Tissue Specific' signal processing
- Ability to store reasonable number of images
  The number of images that can be stored must be stated.
- Large Cineloop
- Extended signal processing facilities
- Customizable pre-sets and calculations, for individual users and for different types of applications, for all modes
- Automated 'INTERGROWTH-21st Study" pre-sets
- Full DICOM-3 image and Cineloop transfer activated
- Hard Disk storage
- CD rewriter or DVD rewriter for backup of presets, configuration and archiving of studies in DICOM format
- DVD ±R(W) Drive
- > 100GB on board archive
- S video or composite video out (ideally all of the following: RGB, composite, S video, VGA and DVI)
- Ability to save images / cineloops in jpeg / avi format to external media for teaching purposes and ideally the ability to store images / cine loops to USB key
- On screen measurement of distance, area and circumference

For peer review only - http://bmjopen.bmj.com/site/about/guidelines.xhtml

**OPTIONAL**	**3D / 4D capability**

(using ellipse or tracing methods), where a minimum of four simultaneous distance measurements or one area/ circumference measurement is required

- High definition B-mode
  - Controls of transmit power, receive gain (including TGC) and dynamic compression
  - Dynamic transmit and receive focusing with user selection of focal zones
  - Configurable spatial/temporal averaging
  - Measurement tools such as distance, area, circumference and 2D-area (ellipse or tracing)

Scanheads

- The scanner should have, at least 2 active transducer ports from which one can be selected from the control panel.
- Broad band 2D probe which must be suitable for examinations at 9-14 weeks, 21-23 week and third trimester growth scans and with a penetration suitable for a wide variety of patients.
  E.g. Broadband 3-6 MHz and broadband 5-9 MHz probes or a single broadband probe of 3-9 MHz

Accessories

- Thermal printer capable of printing onto continuous standard thermal paper
- DVD player/recorder

General Configuration

- The unit should be compact and provide reasonable portability.

Safety

- The unit should meet or exceed performance and safety requirements of UL 544, CSA C22.2 and IEC 60601-1 and be CE marked

Maintenance

- In order to ensure the equipment is maintained in proper working order, the basic maintenance requirements are listed below. Suppliers are required to specify the services they will provide and their costs:
  - Clear instructions on the regular maintenance that should be carried out by the user
  - If the Unit needs regular maintenance, the frequency with which any parts need replacing
  - Engineer call-out response time of 48 hours following initial assessment/advice from the hospital's maintenance team
  - Options available if the equipment is out of action for an extended period
  - Details of any maintenance contract(s) available to cover all normal device deterioration and emergency repairs
- The maintenance support should be such that there is automatic supply upgrade of new firmware

For peer review only

- 3D Broadband 3-6 MHz or 3D broadband 5-9 MHz probes
- Spatial Temporal Image Correlation (STIC) – option – capture full fetal heart cycle in real time using volumetric transducer
- Surface rendering **disabled**
- Volume Contrast Imaging
- Specific software for post-processing stored 3D volumes.

HD Flow imaging (bidirectional Power Doppler), Tissue Doppler, Spectral Doppler, Colour velocity mapping and Power Doppler mapping

- Controls of power, gain, velocity range, baseline shift, low pass filter and compression
- Control for display time base
- Manual and automatic spectral analysis and waveform index estimator
- Volume flow measurement, User defined calculations
- Good sensitivity, temporal and velocity resolutions
- Acceptable Range gate registration accuracy, Gate duration, Beam width, Penetration depth, Velocity, Direction indication, Directional discrimination, Velocity estimation accuracy, High-pass filter, Waveform index estimation accuracy, Volume flow estimation accuracy
- Acceptable Colour direction indication, Lowest detectable velocity, Highest detectable velocity, Image spatial resolution (axial, lateral and slice thickness), Temporal resolution, Velocity resolution, Tissue colour suppression, Angle dependence of colour, Registration colour/B-scan image, Penetration depth

Further Notes

- Any non-compliance with the specification must be clearly identified together with any alternative or additional features.
- A decision may be taken to lease the selected device. If so, a tendering exercise will be undertaken to choose an appropriate leasing company.
- The availability of any training including courses offered / recommended by the manufacturer / supplier should be stated, together with any associated costs that may be incurred. Preference will be given to manufacturers / suppliers offering free fault finding, preventative maintenance, and first line maintenance training courses to two members of each participating centre's maintenance team.
- The cost of any contracts available for the regular service and/or maintenance of the devices should be separately stated.
- Prices should include any discounts or special offers available, including those of any ex-demonstration units that may be available, but these should be separately detailed.
- The terms of guarantee must be stated.

5. Quality Control

Quality assurance and control of the Ultrasound component of INTERGROWTH-21st will be the remit of an Ultrasound Quality Unit (USQU) (see section 6 below).

In order to ensure ongoing quality control we will employ rigorous tools for training, assessment and certification under the supervision of qualified instructors. Intra-observer and inter-observer measurement errors will be assessed during the training course before initiating the study.

Ongoing quality control and adherence to protocols will be performed. This will include quality assessment of images, random evaluation and repetition of ultrasound measurements, and assessment of collected data. It is envisaged that a limited number of

For peer review only - http://bmjopen.bmj.com/site/about/guidelines.xhtml

experienced obstetric ultrasonographers will form the research teams at each study site. They will be provided with standardized equipment and the ultrasound protocol describing all measurement techniques, protocols and procedures for training and clinical use.

5.1 Initial training and standardization of all study 2D-ultrasonographers against an international lead ultrasonographer, and assessment of local conditions

5.1.1 Initial training, assessment and standardization of ultrasonographers

It is recognized that the local ultrasonographers will already have a high standard of training. Therefore, the goals of initial training are:

- To ensure all ultrasonographers are familiar with equipment to be used in the study, including the ultrasound machine, the SOPs, the automated recording of ultrasound measurement values, software and entry of data.
- Ensuring standardization by training, assessment and certification. The schedule for this is as follows:
  1. Individual and group theoretical training (Coordinating Unit)
  2. Hands-on ultrasound training (Coordinating Unit)
  3. Submission of a log of 10 measurements of each parameter. These measurements will be repeated three times from three different images (to assess intra-observer variability), and repeated offline by the external sonographer (to assess inter-observer variability):
  4. Measurements should, on average, score >75% of the max score (i.e. 4 and 3 respectively, see below (5.2.1)
  5. No more that one out of the ten repeated measurement by the same operator should vary of more than two standard deviations (SD) of the measurement error of each given parameter at the given GA.
  6. No more than two out of the ten repeated measurements by a different operator should vary by more than two SD (proxy for random error) and there should not be a systematic bias of more than 0.5 SD.
  7. If these criteria are not fulfilled, certification will not be given.
  8. If the criteria are fulfilled certification of competence will be given prior to the start of the study

5.1.2 Pilot test-retest study

Similar to the anthropometry component of the study, quality control of ultrasound measurements will be primarily based on the comparison of repeat measurements by the same or different observers. Routine data collection will be guided by a system of maximum allowable differences between replicates. However, in contrast to anthropometry, for ultrasound measurements not much is known about what the maximum allowable differences should be for the different fetal measures at different stages of pregnancy. It is not known how exactly fetal size and GA influence measurement error in ultrasound. In addition, it is not clear how much measurement error in ultrasound is influenced by expertise and how expert-level and non-expert-level measurement errors compare. A pilot test-test study is therefore proposed to determine the Technical Errors of Measurement at both levels of expertise and trends to bias (in comparison with expert), specifically for:

- CRL between 9^{+0}-14^{+0} weeks
- BPD, HC, FL, AC in the different GA intervals that will be used in the study:14-18, 19-23, 24-28, 29-33, 34-38 and 39-42 weeks

This pilot study will be carried out in the unit at Oxford. It will involve one or several other obstetricians who regularly do ultrasound measurements in the same hospital. All observers should be familiar with the measurement protocol of the study and should have received at least one training session by the LU prior to the start of the pilot study. The same equipment must be used as the one that will be used in the main study. For each of the seven GA

For peer review only

intervals (from 9^{+0}-14^{+0} to 39-42 weeks) a number of 10 or 15 women will be enrolled over a 2-month period (November – December 2008).

It is proposed that each woman undergoes 4 independent assessments in a random order, two assessments by the LU and two by another ultrasonographer in the hospital. Each assessment will consist of an ultrasound session during which all the measurements needed for the GA interval will be performed in a random sequence. For each measurement a number of metadata (factors potentially modifying measurement error) could be collected so that the pilot study of itself becomes more publishable. All observers should ideally be blinded to the measurement values they obtain by automatic transfer of values to the online system.

This pilot study will yield GA-specific Technical Errors of Measurement and other statistics of inter- and intra-observer reliability and bias. These, in turn, will be used to define the maximum allowable differences for each fetal measure at each age that will be used to: (1) evaluate success of initial training (2) identify the need for re-measurement in the routine data collection, and (3) interpret data quality statistics from the standardization exercises and the random re-measurements.

5.1.3 Assessing local factors: the initial site visit

Prior to the start of the study, an initial site visit will be performed by a member of the USQU. The aims are to ensure:

- Preparation of the local data quality control activities, which will involve
    - Identification of a local supervisor of ultrasound data quality control activities. This will often be the ultrasonographer with the best data quality statistics and closest in performance to the LU during the initial LU visit. The person will have good organizational and team working skills and be competent in using the required software. The local supervisor should be instructed and trained to do the following tasks:
        - Conduct refresher sessions every two months, and document observer performances
        - Conduct data quality control re-measurements on a random sample of routine measurements
        - Observe as many routine measurements as possible performed by all site ultrasonographers
        - Extract and analyze, in collaboration with a LU, data from routine standardization sessions and from random re-measurements done at the site.
        - Liaise with LU about problems with ultrasonographers, SOPs and data quality statistics
    - Identification of a local data management coordinator who will liaise with the overall study coordinator.

5.2 Quality control of measurements in the FGLS: Monitoring and feedback of performances of ultrasonographers and identification of needs for retraining

5.2.1 Qualitative quality control

All measurements taken by ultrasonographers will be qualitatively controlled and scored by the Research Fellow.

For peer review only - http://bmjopen.bmj.com/site/about/guidelines.xhtml

Scores will be given according to the described scheme by *Salomon et al*[11]. Each specific criterion scores one point; thus, the maximum score is 6 points for BPD/HC; 6 points for AC; and 4 points for FL.
Any ultrasonographer with more that 10% of images rejected in a given period will have his/her certification withdrawn and will undergo re-training.

Objective scoring system for still images (modified from Salomon et al[11]*)*

BPD/ OFD/ HC	*AC*	*FL*
Symmetrical plane	Circular plane	Both ends of the bone clearly visible
Plane showing thalami	Image shows the stomach bubble	<45◦ angle to the horizontal
Cavum septum pellucidum 1/3 along midline echo	Image shows umbilical vein along 1/3 of the abdomen	Femoral plane occupying at least 30% of the total image size
Cerebellum not visible	Kidneys not visible	Calipers placed correctly
Fetal head occupies at least 30% of the total image size	Abdomen occupies at least 30% of the total image size	
Calipers and dotted ellipse placed correctly	Calipers and dotted ellipse placed correctly	

In case of clearly incorrect caliper placement, images will be rejected and measurement excluded. If the image scores half or less of the maximum score (i.e. 3 points or 2 points, respectively), measurements will also be excluded.

5.2.2 Quantitative quality control: Intra-observer reliability

Intra-observer reliability will be prospectively assessed based on the three concealed measurements taken routinely. No more that **one out of the ten** repeated measurement by the same operator should vary by more than two SD of the given parameter at the given GA.

If this is not fulfilled, certification is cancelled and the sonographer identified as requiring re-training. Feedback and discussion between USQU and site supervisors will follow in order to highlight the need for re-training and to carry out re-training.

5.2.3 Quantitative quality control: Random re-measurement on 2D images

In order to assess correct caliper placement, a random sample of 10% of all measurements will be re-measured on still images by the Clinical Research Fellow / LU. This will allow production and evaluation of inter-observer reliability and bias (against the LU) for each observer based on the random re-measurements. No more than **one out of the ten** repeated measurement by a different operator should vary by more than two SD and there should not be a systematic bias of more than 0.5 SD.

If this is not fulfilled, certification is cancelled and the sonographer identified as requiring re-training. Feedback and discussion between USQU and site supervisors will follow in order to highlight the need for re-training and to carry out re-training.

5.2.4 Quantitative quality control: Random use of 3D images to re-measure planes

In order to assess correct image acquisition, a random sample of 5% of all 3D volumes will be used to acquire the ideal measurement plane by the Clinical Research Fellow / LU. This will allow evaluation of inter-observer reliability and bias (against the LU) for each observer based on the random re-measurements. No more than **two out of the ten** repeated measurement by a different operator should vary by more than two SD (proxy for random error) and there should not be a systematic bias of more than 0.5 SD.

For peer review only - http://bmjopen.bmj.com/site/about/guidelines.xhtml

If this is not fulfilled, certification is cancelled and the sonographer identified as requiring re-training. Feedback and discussion between USQU and site supervisors will follow in order to highlight the need for re-training and to carry out re-training.

5.2.5 Site standardization exercises.

Local test-retest exercises involving all site observers are an opportunity for refreshing the understanding of the protocol, better focus on the protocol, re-training and better comparison of observers as each observer measures the same subjects, which is not the case for routine measurements and for the random quality control re-measurements.

- Organize periodic production and evaluation of data quality statistics for each observer based on the measurements taken during the standardization sessions
- Feedback and discussion between USQU and site supervisors about data quality statistics from the standardization sessions; identification of need for re-training; organize and carry out re-training
- These exercises will be done on 10 patients every 6 months, or more frequently depending on UQDU recommendations

5.3 Optimizing the data systems to accommodate the needs for quality control

The data entry system should allow incorporating measurement values and data from quality control and standardization exercises and be able to identify these as such.

- The data system could select a random sample of non-supervisor routine measurements for QC re-measurement. Ultrasonographers will be unaware which measurements will be re-measured when they do their measurements.
- Data extraction routine for monitoring of data quality statistics of entire study, sites, and individual ultrasonographers
- Establishment of a research dataset on determinants of ultrasound data quality, to be extracted from the main database.
    - Possible factors causing variability in ultrasound measurements with a given type of instrument may include, among others:
        - Parity
        - BMI
        - GA, size
        - Presentation / position of fetus
        - Sex
        - Liquor volume
        - Order of measurements
        - Observer factors
        - Measurement setting factors

For peer review only

5.4 Reporting, analysis and publication on ultrasound data quality

5.4.1 Central reporting

The USQU will produce 3-monthly data quality statistics based on standardization sessions, QC re-measurements and routine replicates. This may include, as appropriate the production of statistics and trend plots of:

- Intra-observer reliability of individual ultrasonographers
- Inter-observer reliability of individual ultrasonographers against LU
- Site bias against LU
- Site intra-observer reliability
- Site inter-observer reliability against supervisor
- Comparison among sites
- Proportion of failed checks on maximum allowable differences and on range checks

B. Publication plan

- Publications on ultrasonographic training and data quality will be integrated into the study's overall publication policy
- Possible topics include:
    - Paper on ultrasound data quality in INTERGROWTH 21st
    - Research questions using the research dataset on ultrasound data quality
        - Determinants of observer bias in ultrasonography
        - Determinants of observer reliability in ultrasonography
        - Differences between concealed and revealed measurements
        - Difference between 2D and 3D measurements
        - Differences in data quality statistics obtained from training sessions, routine data collection, quality control, and standardization sessions

For peer review only

Figure: Summary of quality control

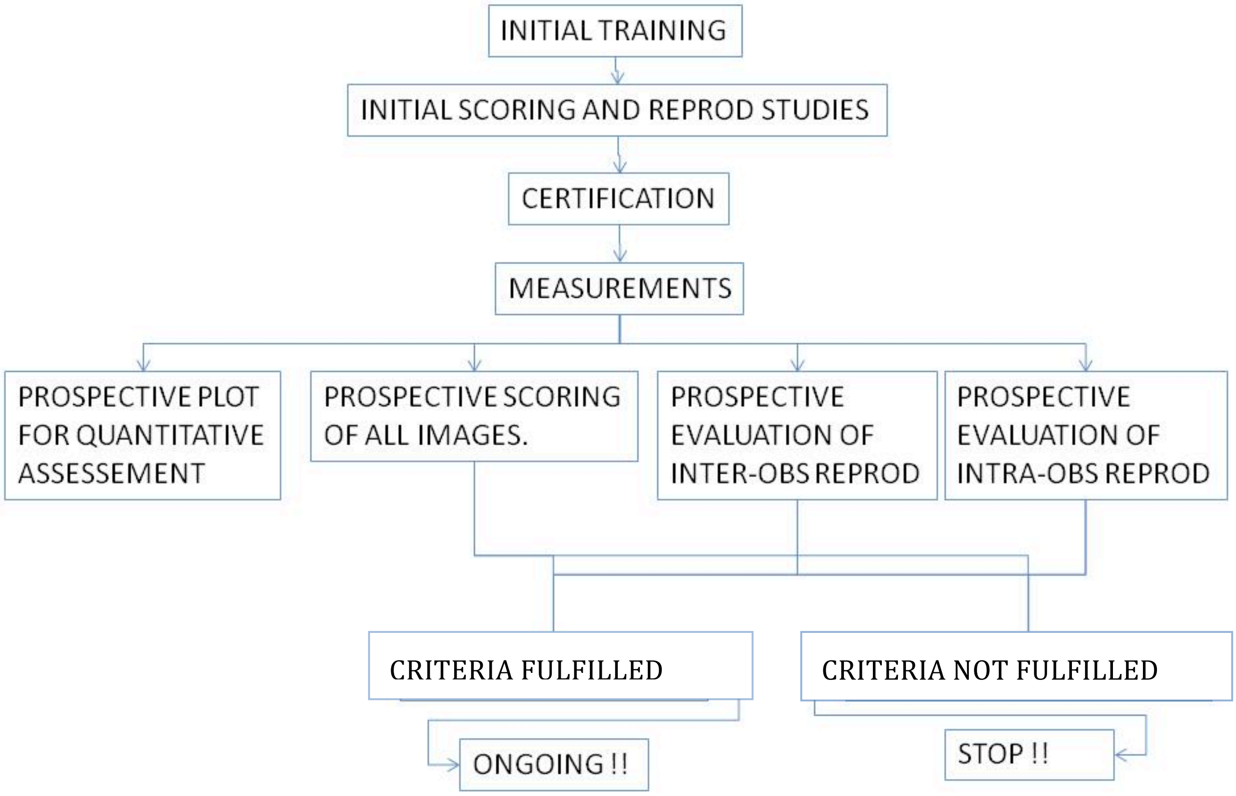

6. Ultrasound Data Quality Unit
Quality assurance and control of the Ultrasound component of INTERGROWTH-21st will be the remit of a Ultrasound Quality Unit (USQU). USQU will be coordinated by Aris Papageorghiou who will be assisted by External Ultrasound Experts (Laurent J Salomon and Shaida Zaidi) and a Clinical Research Fellow (Study protocol p.34). This unit will coordinate with the anthropometric quality control unit and both will follow similar protocols.

6.1 Proposed activities of the USQU
The USQU will be responsible for:

1. Development of standard operating procedures (SOP)
2. Initial training, assessment and certification of ultrasonographers
3. Standardizing all study 2D-ultrasonographers against a lead ultrasonographer (LU)
4. Monitoring and feedback of on-site standardization levels and performances of individual ultrasonographers; identification of needs for retraining
    a. Quality control of routine measurements
    b. Random sample of replicate measurements
    c. Site standardization exercises
5. Optimizing the data system to accommodate the needs for quality control
6. Reporting, analysis and publication on ultrasound data quality
    a. Central reporting
    b. Establishment of an analysis dataset on ultrasound data quality, to be extracted from the main database
    c. Publication plan

For peer review only - http://bmjopen.bmj.com/site/about/guidelines.xhtml

6.2 Ultrasound protocol and standard operating procedures (SOP).

This SOP includes procedural guidelines on:

- Patient selection
- Preparation of subject for measurement
- Sequence of measures and replicates; blinding to previous measurement values
- Measurement technique
  - Positioning of subject
  - Handling of caliper, identification of planes and landmarks, marking distances, recording and transfer of results
  - Entry of measurement metadata
- How to deal with special fetal positions and other unusual circumstances; circumstances under which one should delay or forego the assessment or part of the assessment;
- Release of measurement values and GA calculations for clinical purposes
- Preparation of measurement setting and instruments; linkage with computer and database
- Ultrasound device and accessories: maintenance, transport, storage, calibration checks

6.3 Unanticipated changes to this protocol / SOP

Despite all efforts, it is likely to be impossible to anticipate all possible eventualities in a study of this size. Therefore, changes to this protocol will be possible. These will be proposed to the Steering Committee by the USQU, and incorporated if approved.

For peer review only

References

1. The International Fetal and Newborn Growth Consortium for the 21st Century INTERGROWTH-21st Study Protocol. Oxford University, 2008

2. Cetin I, Cozzi V, Antonazzo P. Fetal development after assisted reproduction - a review. Placenta. 2003 Oct;24 Suppl B:S104-13

3. Jacob S, Moley KH. Gametes and embryo epigenetic reprogramming affect developmental outcome: implication for assisted reproductive technologies. Pediatr Res. 2005 Sep;58(3):437-46

4. Robinson HP. Sonar measurement of fetal crown-rump length as means of assessing maturity in first trimester of pregnancy. Br Med J. 1973 Oct 6;4(5883):28-31

5. Robinson HP, Fleming JE. A critical evaluation of sonar "crown-rump length" measurements. Br J Obstet Gynaecol 1975;82:702-10.

6. British Medical Ultrasound Society. Fetal size and dating: Charts recommended for clinical obstetric practice. February 2007 (Revised: January 2008)

7. Chitty LS, Altman DG, Henderson A, Campbell S. Charts of fetal size: 2. Head measurements. Br J Obstet Gynaecol 1994; 101: 35–43.

8. Chitty LS, Altman DG, Henderson A, Campbell S. Charts of fetal size: 3. Abdominal measurements. Br J Obstet Gynaecol 1994; 101: 125–131.

9. Chitty LS, Altman DG, Henderson A, Campbell S. Charts of fetal size: 4. Femur length. Br J Obstet Gynaecol 1994; 101:132–135.

10. Snijders RJM, Nicolaides KH. Fetal biometry at 14–40 weeks' gestation. Ultrasound Obstet Gynecol 1994; 4: 34–48.

11. Salomon LJ,. Bernard JP,. Duyme M, Doris B, Mas N, Ville Y. Feasibility and reproducibility of an image-scoring method for quality control of fetal biometry in the second trimester. Ultrasound Obstet Gynecol 2006; 27: 34–40

12. International Society of Ultrasound in Obstetrics & Gynecology Education Committee. Sonographic examination of the fetal central nervous system: guidelines for performing the 'basic examination' and the 'fetal neurosonogram'. Ultrasound Obstet Gynecol. 2007 Jan;29(1):109-16. Links

13. Royal College of Radiologists: Standards for ultrasound Equipment (2005)

For peer review only

Appendix D: Newborn, Preterm and Maternal Anthropometric Techniques, Equipment and Standardization and Data Quality Assurance and Control Plan.

Please see the Anthropometric Handbook.

For peer review only

Appendix E: Data Management: the Electronic System

The data management process will be performed electronically, simultaneously with data collection, at each study site. Data entry will be implemented on a customized web-based system developed for the study. The system automatically carries out range and consistency checks for immediate correction and reviews percentages of missing/unknown values for each variable. This technology also allows a real-time audit of each site by a set of pre-defined reports included in the system (recruitment, data inconsistencies, etc) run periodically by the Project Data Manager. Additional modules for internal/external messenger, hosting of study documentation (protocol, guidelines, etc) and user administrator make up an integral data management solution for multicentre studies.

The INTERGROWTH-21st website homepage will consist of an open part and a part protected by username and password. The open part will be used for publication of information, news etc. The protected part will contain the data entry application, a reporting module, an alert module, a descriptive statistics module, and an administration module. We are looking into the possibility of linking the application to the external ultrasound data sources.

Ultrasound measurements obtained during the follow-up visits will be electronically transferred from the ultrasound equipment to the web-based system to avoid transcription errors using a database format, agreed between ultrasound equipment manufacturer and system development group. Routine analyses will be carried out regularly for each variable to check digit preference and unusual frequencies of answers that may reflect poor understanding of the instructions. After the information is electronically transferred, the last measured will be made available to the attending staff for clinical use. The application includes an automated user and event logging system.

Data collected will be evaluated by the Data Management Unit for further quality control analyses and compliance with the study protocol, including the analysis of eligibility criteria, the timing of visits and drop-out rates. Measurements for the study taken from the equipment will have a facility to blind all fetal measurement values until they are submitted for the study. Following submission the last value will be printed for use in clinical care.

All other clinical and anthropometric data will be collected initially in a paper form entered onto the on-line system at each local institution by specially trained data management staff. Data should be entered into the system within one week of being collected facilitating the retrieval of possible missing data or other inconsistencies.

For peer review only

Appendix F: Policy and determination of responsibilities for INTERGROWTH-21st

1. Study Coordination and Management

The study's coordination and management, including data management and analyses, are the primary responsibility of the Oxford Maternal & Perinatal Health Institute (OMPHI) within the Nuffield Department of Obstetrics & Gynaecology, University of Oxford, which will act as the study Coordinating Unit (CU). The Maternal Health Task Force at the Harvard School of Public Health will collaborate with OMPHI in the study's overall implementation.

The study coordination and management will include the following activities:

1.1 Study Preparation

Coordination activities:

- Develop protocol with collaborators
- Conduct site visits for selection of study sites
- Selection of committee members and assign tasks to study committees
- Organize collaborators' meetings
- Establish communication procedures between sites and CU
- Administer funding base for coordination activities and study implementation
- Keep donor agencies informed about progress of study

Study materials:

- Prepare data collection forms and supporting documents
- Prepare operation manuals
- Prepare measurement and standardization protocols including Quality Assurance
- Select and purchase equipment
- Prepare data collection forms and other study materials as required

Data processing and management systems:

- Conduct feasibility study
- Negotiate and implement an electronic data management system
- Set-up system for on-line data entry and validation
- Define electronic monitoring reports to be produced
- Pilot data collection systems and monitoring reports

Statistical analysis:

- Develop statistical analysis plan

1.2 Study Implementation

Coordination activities:

- Assist study sites in local adaptation of study materials
- Submit protocol to Ethics Committees
- Distribute data collection forms and other study materials to centres
- Develop standard set(s) of slides for presentations about the study
- Establish training procedures for research staff

1.3 Study Conduct

Coordination activities:

- Coordinate study's day-to-day activities
- Monitor study progress through site visits and on-going review of data entry
- Communicate with local investigators; supporting units; Steering Committee (SC) and Advisory Committee; and other collaborators (e.g. UN agencies, donors)
- Prepare electronic study newsletter

For peer review only - http://bmjopen.bmj.com/site/about/guidelines.xhtml

- Convene regular meetings of staff at CU
- Organize coordination meetings of local Principal Investigators; Steering Committee; site visits; exchange of relevant staff between sites, etc.

Data management and statistical analysis:

- Monitor data collection procedures at study sites and regularly advise SC about all data received and processed
- Validate data and produce queries about inconsistent and/or apparently incorrect data
- Send data queries to study sites so that errors are corrected at collection phase
- Update master file using corrections from validation checks and/or answers to queries
- Evaluate electronic monitoring reports by study site based on recruitment rates, drop-outs, data completeness, quality assurance checks, etc.
- Maintain communication channels between study sites and supporting units relating to queries
- Inform SC whenever a study site requires retraining in data collection or is failing to respond to queries and implement corresponding actions
- Conduct interim statistical analyses of data in accordance with analysis plan approved by SC
- Conduct final statistical analyses of data in accordance with plan approved by SC
- In collaboration with SC, coordinate preparation of articles for publication in peer-reviewed, scientific journals etc.

2. Study Committees

The following Committees will oversee the implementation of the study:

2.1 Steering Committee (SC)

The SC will consist of the following:

- Professional staff from CU and its supporting units
- Principal Investigators (one per site)
- Selected senior scientists
- Representatives from Department of Nutrition and Department of Environmental Health, and other related Institutions as considered appropriate

The SC will meet regularly by conference call or face-to-face meetings to review the study's progress and discuss substantive issues that arise from the study's implementation and conduct. Its role is to make managerial decisions that affect the conduct of the study; to ensure the comparability of data from the different sites, and to resolve problems that arise in running this complex project.

The SC will have an **Executive Committee (EC)** that will meet frequently to review study progress and problems, protocol divergences, and other substantive issues that may arise from the study's implementation. The EC will have overall administrative responsibility for the study's implementation. All changes or alterations to the protocol or issues related to the technical, financial or administrative conduct of the study must be approved by the EC. This Committee will also select the study sites; approve the continuing participation of the sites selected, and determine which data are to be included in the pooled, international data set. Every effort will be made to achieve consensus for all decisions but where this is not possible resolution will be by simple majority with a quorum of at least 5 members. The EC membership will be JV, SK, AN, DA, MdO, ZB, AP, the SC Chair and a Principal Investigator selected by his/her peers. The Project Manager will be an ex-officio member.

For peer review only

2.2 Advisory Committee (AC)

- The AC will consist of senior scientists in the areas of Medicine, Epidemiology and Statistics, Obstetrics and Gynaecology, Paediatrics and Human Biology. The task of the AC will be to provide overall and strategic advice to the SC.

3. Data Management

Electronic data management will be contracted to Medscinet, a private company that has provided similar services in previous multicentre studies we have conducted. It has extensive experience of conducting large multicentre trials and observational studies, and it is recognized as a leading research organization in reproductive health. Although based in Sweden, the company has a UK base in the Department of Women's Health, St Thomas' Hospital, London. It will provide a full on-line data management system, as well as supervision and training support to the participating sites for the purposes of data entry, cleaning, file preparation, and study monitoring. All participating sites will be required to adhere to the study's data management system, which will be available on the study web page. We have successfully used this system in two previous multicentre studies involving sites included in INTERGROWTH-21st.

4. Ancillary Studies

Proposals for ancillary studies from one or more local Principal Investigators will be considered as long as a) they do not result in alterations to the main protocol and b) they do not impact adversely on subject recruitment and/or participation in the main study. All ancillary studies and/or additional data collection must be submitted to the EC for written approval before implementation. A letter of intent must be submitted to the CU at the conceptual stage of the ancillary study. If approved by the EC, the local Principal Investigator(s) will be invited to submit a detailed proposal that should include the study's rationale, objective, methodology, expected outcomes and budget.

5. Site Monitoring

Study progress will be monitored by:

a) Monthly reports for each study site produced by the CU using data from the electronic data bank, which will be shared with the study sites and EC members.

b) Visits (the frequency as required by the progress of the study) to all study sites to ensure that the study is uniformly implemented (the timing to be decided by the CU in coordination with the EC and Anthropometric Quality Control Unit (ADQU). The activities to be performed during each visit include, but are not restricted to:

- Meeting site Principal Investigator and members of study staff
- Review of study procedures, use of data forms and related documents
- Observation of study personnel carrying out specific procedures
- Particular attention will be paid to standardization of data collection (e.g., adherence to study protocol, and measurement/standardization protocols)
- Review of coverage and participation rates, including characteristics of participants and non-participants, and reasons for loss to follow-up
- Observation of data validation procedures
- Physical walk-through of certain procedures (e.g., screening to determine subject eligibility or taking of informed consent)
- Conversations with key support personnel to assess their practice with regard to data collection
- Inspection of study facilities and subject study files

For peer review only - http://bmjopen.bmj.com/site/about/guidelines.xhtml

c) Exchange visits by field supervisors, regional and global meetings of field directors to facilitate standardization of procedures across sites.

6. Dissemination of Results, Data ownership, Publications and Authorship

6.1 Dissemination of Results

The results of the study will be disseminated as widely as possible, in publications and the mass media. Activities will include:

- Publication in peer-reviewed journals of all papers reporting primary and secondary outcomes based on the pooled international data set
- Publication of papers and editorials in regional journals that are published in local languages, such as Bulletin of PAHO (Spanish), Chinese, French or African Journals
- Electronic study newsletter
- Publication in periodicals from other collaborating institutions.
- Presentations at local, regional and international meetings
- Publication of a book including all collected information (e.g., growth curves) and detailed methodological issues unlikely to be included in reports to peer-reviewed journals. The Executive Committee in coordination with Local investigators will take responsibility for this task.

6.2 Data Ownership

All data derived from the INTERGROWTH-21st multicentre project will be the property of the Oxford Maternal and Perinatal Health Institute (OMPHI) at the University of Oxford. Individual investigators will share the ownership of their site-specific data sets with OMPHI. After publication of the growth curves and the main papers related to the secondary objectives, the pooled data set will enter the public domain at a time to be determined by the SC. The anonymity of all study participants will be ensured.

6.3 Publications and Authorship

All papers reporting the new growth charts (Primary Objective, components 1-3), based on the pooled international data set (the growth curves) will be published under corporate authorship (International Fetal and Newborn Growth Consortium) The address for correspondence will be the Oxford Maternal & Perinatal Health Institute (OMPHI), Nuffield Department of Obstetrics & Gynaecology, University of Oxford, John Radcliffe Hospital, Oxford OX3 9DU, UK.

For these publications, the names of the members of all committees and study units will be listed at the end of the paper (see Lancet 2006;367:1819 for an example of the format) plus their individual contributions to the study (e.g., protocol development, study coordination, data management, data analysis, manuscript preparation, etc) as required by the individual journal.

Sites participating in the multicentre project will be listed alphabetically by country; the local Principal Investigator will be responsible for selecting the authors within each country. It is understood that authorship, within each site, will be offered to those who have made a substantial contribution to the study. Within each site, the order of the author's names will be the responsibility of the local Principal Investigator. The SC will provide a list of people to be mentioned in the standard Acknowledgments, once it has been agreed what type of support will be acknowledged. All papers arising from the study should include such a list of acknowledgements. The University of Oxford will give certificates of collaboration to doctors, nurses and local staff who contributed to the study but whose names cannot appear in publications.

For peer review only - http://bmjopen.bmj.com/site/about/guidelines.xhtml

For all other papers arising from the study (except the main growth charts described above), including those relating to secondary objectives, a modified system of corporative authorship will be used, in which SC members will be listed by name according to an agreed rotation based on personal interests and leadership in the preparation of the paper. These names would be followed by "for the International Fetal and Newborn Growth Consortium" or similar statement as agreed by the SC. The authorship order will rotate based on a system agreed by the SC, i.e. initial lottery and rotation thereafter or some other system.

The study will have a **Publications Review Committee (PRC)** consisting of the EC members, a Local Principal Investigator and one or two *ad hoc* Senior Scientists invited according to specific needs. The PRC will be responsible for:

- Preparation of a list of expected publications on the primary and secondary outcomes of the study, with the authorship strategy for each set according to the rules above which are to be approved by the SC.
- Setting-up writing committees for the primary outcome, corporate authorship papers and a review mechanism by the SC of the final drafts that are submitted for publication.
- Reviewing and approving all papers sent for publication with the goal of maintaining internal consistency of material and methods, as well as authorship policies.
- Reviewing and approving all site-specific analyses proposed by local Principal Investigators.

The following more general rules will apply:

- Individual sites will not be allowed to publish analyses of country-specific data or subsets of sites that, in the EC's assessment, have the potential to be misconstrued as standard data for particular nations or population groups.

- However, individual sites and groups of sites are encouraged to publish other analyses of data based on their site-specific data. These analyses must be cleared by the EC before preparation. The order of author's names in publications based on site-specific analyses will be the responsibility of the local Principal Investigator. The EC will provide the Acknowledgments and funding sources, in a standard format.

- The CU is encouraged to publish methodological and conceptual papers that describe the methods and procedures used in the study. The order of authors' names for these publications will follow the modified corporative authorship model including the SC as well as the CU members that lead the preparation of the papers. The CU will provide the Acknowledgments in a standard format.

- Scientists are encouraged to present the study at scientific conferences and meetings. When scientists are invited to international or regional meetings to present the study, the CU should be informed and prior written approval is required with details of the type, venue and organizers of the meeting. The CU will keep an archive of all materials presented at meetings and make them available at the study's web page.

Press enquiries will be honoured unless there are some operational or scientific reasons for withholding information. Requests for interim results or other details arising during the study which if honoured are likely to have an adverse effect on the study, will be denied. Only one individual will be authorized to interact with the press (the local Principal Investigator or public relations officer of his/her institution) at every site in coordination with the CU. In the multicentre study context, the EC will respond to queries concerning the overall study design or results. The SC will define the type of queries that may be answered locally and those that must be referred for response to the EC. Publicity concerning study results in preparation will be avoided.

For peer review only - http://bmjopen.bmj.com/site/about/guidelines.xhtml

Appendix G: Management Structure

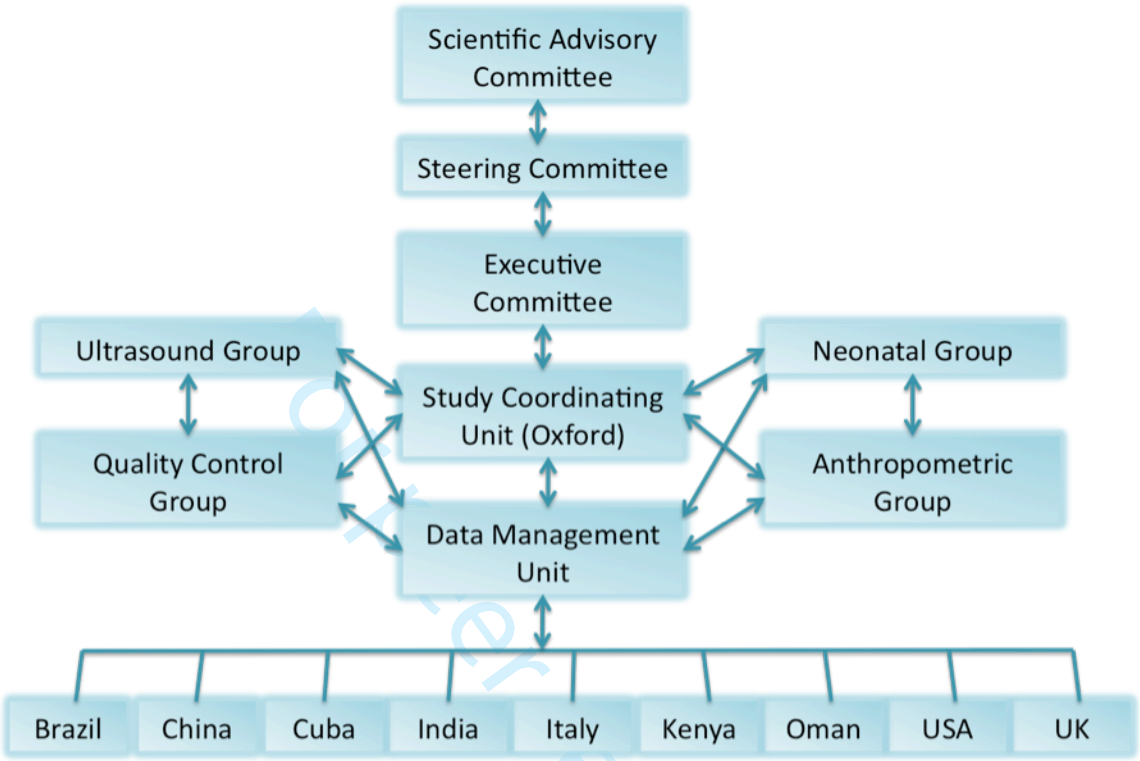

For peer review only

Appendix H: Selection of Study Sites Flow

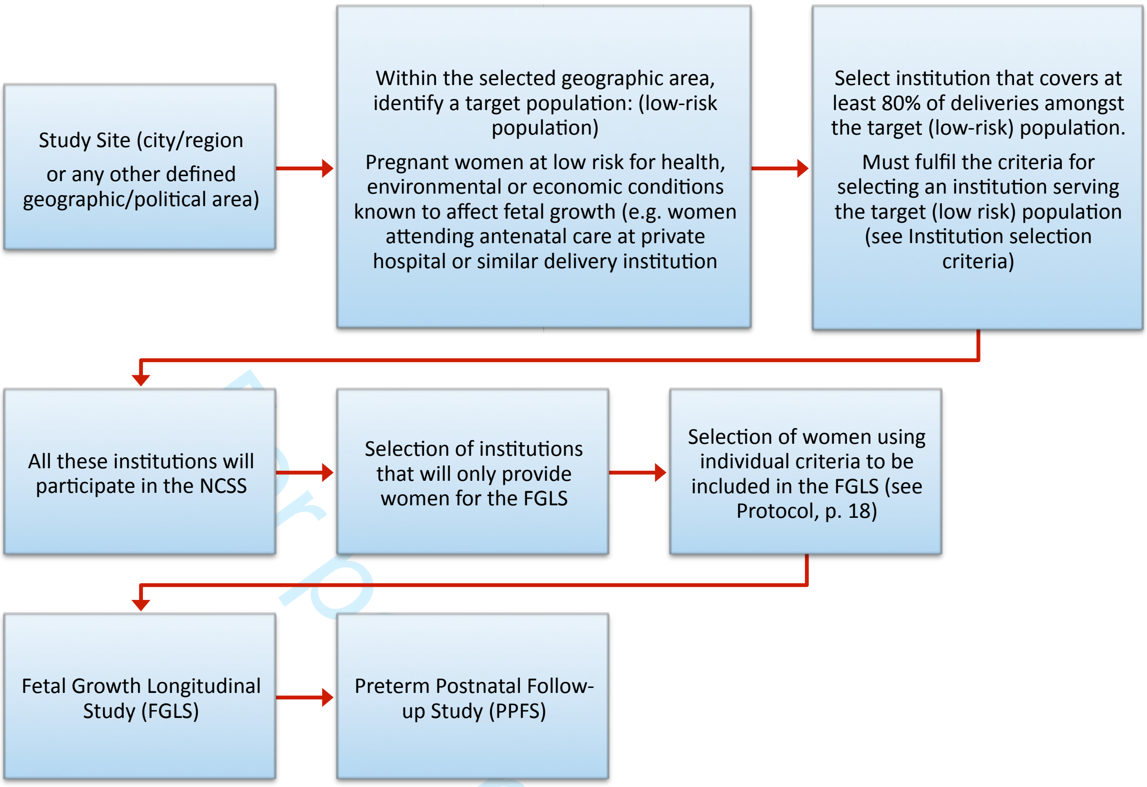

Institution Selection Criteria

a) LBW rate <10% and mean birth weight >3100g;

b) located at an altitude below 1600m;

c) perinatal mortality <20 per 1000 live birth;

d) mothers attending antenatal care in these institutions should plan to deliver in that or a similar hospital located in the same region;

e) >75% of mothers have attained an educational level greater than the locally defined cut-off point;

f) lack of known non-microbiological contamination such as pollution, radiation or any other toxic substances (this will be evaluated in collaboration with the WHO Department of Protection of the Human Environment);

For peer review only

Appendix I: The Pregnancy Physiology Pattern Prediction (4P) Study Details of observation measurement

Additional observations

Minimum dataset: In addition to blood pressure (that is currently recorded), temperature, respiratory rate, heart rate and oxygen saturation will be recorded at each ultrasound scan visit; this should only add 2 minutes to each visit.

Minimum intra-partum and post-partum dataset (hospital): All routinely collected intra-partum and post-partum measurements (blood pressure, temperature, respiratory rate, heart rate and oxygen saturation) will be added to the dataset.

Full dataset: Each participant will, if willing, be trained in the use of home monitoring equipment. The participants will then be asked to provide a daily dataset for the 2 weeks after delivery.

- Resting blood pressure will be recorded, but not displayed; readings will be downloaded into the research database at each scan visit.
- Resting pulse oximetry and heart rate will be recorded and directly transferred via the (bluetooth-connected) smartphone to the research database.
- Temperature will be taken and entered by the participant into the smartphone provided and automatically transferred to the research database.
- Automatic text reminders will be sent to the smartphone when datasets have not been received.

A research midwife will be employed to visit participants, reinforcing observation techniques. A complete dataset will be obtained during each visit. Two weeks after delivery, study personnel will arrange pick-up of the home monitoring equipment; a final dataset will be collected at this visit.

Blood pressure: When measuring blood pressure in the clinic or in the home, standardise the environment and provide a relaxed, temperate setting, with the woman quiet and seated, and her arm outstretched and supported. Blood pressure should be measured approximately 5 minutes after the woman has been seated and relaxed, with legs uncrossed. The arm should be supported at heart level. The correct cuff size should be used:

British Hypertension Society
Standard cuff Bladder 12-26 cm for the majority of adult arms
Large cuff Bladder 12-40 cm for obese arms
Small cuff Bladder 12-18 cm for lean adult arms

(Combined European Society/NICE/British Hypertension Society recommendations)

Blood pressures will be stored within the device and downloaded into the database at scan visits/home visits.

Temperature: Tympanic and oral temperature will be measured at each scan visit, using standard techniques. Self-measurement of temperature will be via the tympanic route only and will be entered into the smartphone by the participant. Midwife home visits will record both tympanic and oral temperature.

Pulse oximetry: Oxygen saturation and heart rate will be recorded at each scan visit using standard hospital equipment. For each self-observation set, oxygen saturation and heart rate will be relayed to the smartphone, and uploaded directly to the research database.

Respiratory rate: Respiratory rate will be manually counted for 1 minute at scan and home visits. At the end of each home observation set, respiration rate will be estimated using an accelerometer within the smartphone, with the smartphone applied to the chest by the participant sitting back in her seat quietly for 30 seconds.

For peer review only - http://bmjopen.bmj.com/site/about/guidelines.xhtml

Appendix J: The PEA POD Manual of Operations

The PEA POD uses a series of standard equations to estimate body composition. Those equations require a subject's weight (w), length (l) and volume (V). Weight and volume are measured by the machine itself. Length needs to be measured beforehand.

Warnings and cautions

The machine also requires a quiet and stable environment:

It must be on a flat floor (can be checked in System setup → inclinometer)
Not to be near a heater, air conditioning machine, fan or under direct sunlight
Not to be near a window, a door (especially one that could be opened during testing)
It is sensitive to low frequency noise
It must not be touched during a test
The room temperature (RT) should be between 20-28°C. If RT is out of range (by even 0.1°C), an alarm will go off and what you are doing will be lost. To prevent that if happening, ideally keep RT around 25°C (open windows and AC also limit the number of people in the room)
Moreover, RT should not vary by more than ±0.5°C during testing.
The test chamber temperature is maintained at 31°C
Room humidity 20-70%, variation during testing ±5%
Atmospheric pressure between 86-106kPa

The machine is designed for newborns and infants between 1 to 8kg (from birth to 5-6 months).
Do not turn the machine on if you are not using it.
The machine will need cleaning after each usage.
Also worth knowing, the temperature in the PEA POD room increases very quickly.

Working sequence

Turn PEA POD and air-conditioning on
Warm-up (2h)
Calibration:
Analyze hardware → Scale calibration * →Scale check → Autorun → Volume
*: scale calibration is to be performed every 2 weeks or if the PEA POD has been moved.
Test:
Volume → Enter patient's data → Scale → Volume measurement → Cleaning
Repeat as many times as the number of babies to measure
Print out results & Back-up
Log out
Turn machine off

Turning on/off the machine and warm up

Check the machine is on at the wall socket and at the back of the machine (main). The PEA POD and the PC will turn on together and the PEA POD software will launch automatically. The log-in window appears.

For peer review only - http://bmjopen.bmj.com/site/about/guidelines.xhtml

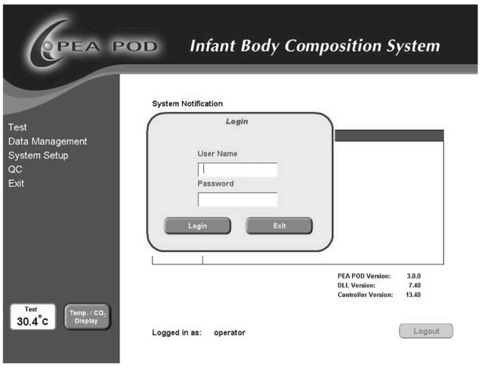

<u>Log-in information</u>:

User name: jiang

Password: peapod

(Data management password: admin)

When you turn the machine on, a system error message will be displayed 'Test Chamber Too Cool' → It will disappears when the test chamber has warmed up.

The machine needs 2 hours to warm-up. If tests are plans in the morning, the machine can be left on overnight. However, the running temperature of the PEA POD is 28.8°C, so you will need to turn the AC on or open a window in the morning before calibrating.

When the machine is turned on, the air circulation system starts. It will turn itself off after 10min of inactivity. The PEA POD will require an extra 5min to re-circulate air when becoming active.

On the day, if the machine is left inactive for a couple of hours, it is recommended to do a couple of Autoruns: QC → Autorun (Each takes about 10 min).

Do not turn the machine on if you are not using it.

To turn the machine off:

Log off and exit the PEA POD program (the PEA POD will stop)

Turn the PEA POD and the PC off → bottom right of keyboard, hold pressed for 3s

Turn everything off (main at the back and plug)

Operation

The interface of the machine is very user friendly. Follow the instructions on screen.
Remember to always execute the QC menu after the warm-up. Autoruns should be performed if the machine is left idle for a couple of hours.
Subject's behaviour and preparation:

The subject's hair should be flattened against their head using baby oil. In case the baby has a lot of hair or has curly hair, the baby should wear a cap, provided with the machine.

Calmer subjects make the experience more agreeable – testing after feeding results in calmer subjects. However, subject's behaviours (crying, movement) have been shown not to affect the results.

By default, the models used by the PEA POD are:

Body composition: Fomon
Thoracic gas volume: Stocks
Surface area artefact: Boyd

For peer review only

Menu titles

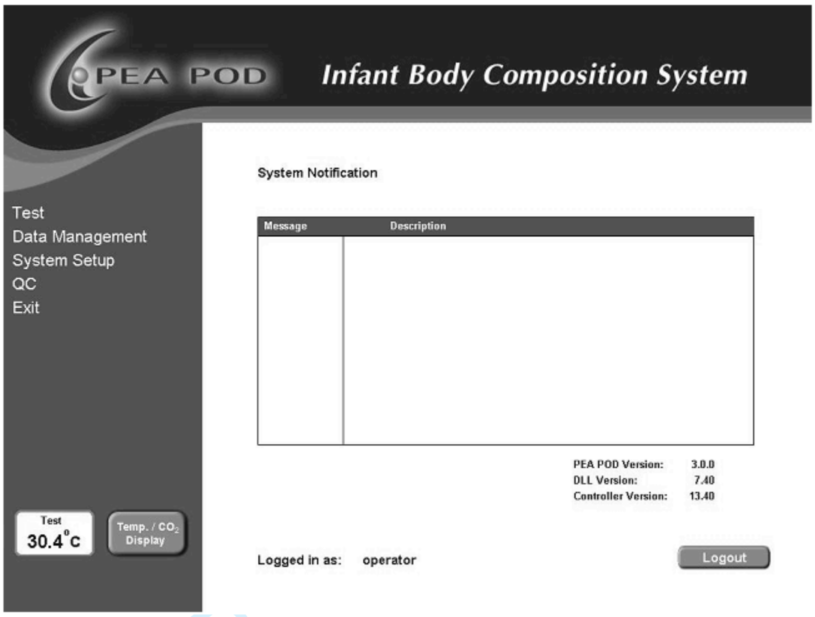

This is the main screen:
In the blue column on the right you will find:
- List of menus
- Environmental conditions (There can be 1 – like here – or 5 indicators)
- Error messages appears just underneath these indicators

In the middle of the screen:
- The system notification box where error message appears.
- Other information: Software versions and user's identity

On the bottom right:
- logout button

Test → **Body Composition** → **Mass**

Select this option when ready to carry out a test (after QC)

Data management → **View subject results**
→ **Modify subject results**
→ **Delete test**
→ **Export result**
→ **Database backup**

Database backup should be based on usage rather than regular time intervals

System setup → **Test profile** → **Create**
→ **Remove**
→ **Density model** → **Create**
→ **Remove**

Do not modify those

→ **Scale checks** → **Noise and drift**
→ **Hysteresis**
→ **Inclinometer**
→ **Facility**
→ **Automatic backup directory**

No need to perform those operations

QC → Analyze hardware
→ Calibrate scale * [4x]
→ Check scale ** [1x]
→ Autorun ** [6x]
→ Volume ** [3x]

* Only to be performed, if:
The PEA POD has been moved
If 2 weeks have elapsed since the last measurement

Remember:
To do run the QC menu everyday
** To record the QC in the log books

Exit

To be performed again if machine left inactive for a couple of hours

Maintenance schedule

After each subject: remove any waste (if applicable); clean scale tray, chamber tray and shields. Wipe with a cloth impregnated with cleaning solution, dry with clean cloth. According to Life Measurement, Inc. We shouldn't use isopropyl alcohol, solvents (like acetone), Virex and phenolic germicidal detergent solutions.

We can *a priori* use our regular wipes: Clinell® or PDI Sani-cloth® wipes.

There should be a clinical waste bin (yellow bin) just outside the PEA POD room (or somewhere along that corridor) where we can dispose of the scale lining, cleaning wipes and dirty nappies.

For peer review only

Do leave that bin outside to be emptied regularly.

- Weekly: clean sides and top using cleaning solution and procedures specified in manual
- Monthly: check pre-filter (replace if dirty) and clean test chamber (Zedong)
- Bi-monthly: replace pre-filter (Zedong)

The pre-filter is situated at the back of the machine on the bottom left if you face the rear of the machine. They are behind a grid and they are blue. They have to be replaced if they appear to collect a lot of dirt.

The Brillianize spray is for the window only.

System errors

Several messages can appear:

- Test chamber too cool
- Ambient temperature out of range
- Scale out of level
- Test and reference chamber temperature too far apart
- Volume sequence time limit **

➔ Contact customer service if persistent

- Calibration valve pneumatic pressure out of specification
- Test chamber door does not close
- Door opened by door fail safe

➔ Contact customer service

➔ techsupport@lifemeasurement.com

** The volume sequence time limit means the time elapsed between the volume measurement test should not have any delays. Meaning, you need to stay at the machine and follow instructions as soon as they appear on the screen.

The measuring sequence is as follow:

V calibration → Patient data entry → Scale taring → Mass measurement → Volume measurement → Results

If 10min have elapsed between the end of calibration and beginning of V measurement, the alarm will go off and the whole measuring sequence of calibration is to be repeated.

Click ‘cancel’ → Test → Body Composition

For peer review only

Alarms

Alarm table – part I

Alarm	Activation criteria	Alarm response	Alarm silence period	Code	Alarm tone
High air temperature	3°C above set T or T>40°C	Audio, LED Screen Test chamber door opens Heater turned off	10min	101 - 109	4 rapid bursts, ½ sec pause, repeat
Air heating system failure	Over-T and/or failure of any system component including any one of the temperature sensors		2min	201 - 211	
Air recirculation system failure	Air flow out of specification		2min	301 & 302	
High carbon dioxide	Carbon dioxide level >0.45%		10min	401 & 402	
Power supply out of range	Voltage out of range		2min	501 - 503	
Remove subject from the scale	Failure to remove subject within 3min of measurement completion	Audio, LED Screen	NA	601	
Continue volume sequence	Failure to continue volume sequence within 10min of previous step		NA	701	

The last 2 alarms (not coloured) are activated by the lack of progression in the testing sequence.

review only

Alarm table – part II

**Communication failure ***	**Loss of communication between GUI and electronics**	**Audio, LED Test chamber door opens Heater turned off**	**10min**	**801**	**3 chimes, 2sec pause, repeat**
**Redundant safety system**	**Controller stops executing code (crashes)**	**Audio, Test chamber door opens Heater turned off**	**None**	**901**	**1 chimes, 2sec pause, repeat**
	**Controller spontaneously reset itself**	**Audio**	**None**	**902**	**2 chimes, 2sec pause, repeat**
**Alarm**	**Activation criteria**	**Alarm response**	**Alarm silence period**	**Code**	**Alarm tone**

The sections that are coloured represent alarms that require the PEA POD to be switched off (keyboard) and then also at the main (back of the machine)

* This alarm require the PEA POD and the PC to be switched off independently

Responding to alarms

In case of one or multiples alarms, the following steps should be followed:

Recognize the occurrence of an alarm by appearance of one or more alarm messages on the screen, the blinking LED light on the top right corner of the touch pad area, and the audio alarm sound.

If the subject is on the scale tray or in the test chamber, remove the subject immediately from the PEA POD. Place the subject in a safe place before attending the alarm. Do not place the subject on the scale tray while attending to the alarm.

Silence the alarm by pressing the power button located in the bottom corner of the touch pad area for one quarter second or by clicking on the <SILENCE> button on the alarm message displayed on the screen. Note that Redundant Safety System alarms cannot be silenced.

Note the name of the alarm displayed, associated code, and tone (the tone is particularly important for Communication Failure alarms and Redundant Safety System alarms, as in those cases there will not be a software displayed message specifying an alarm name and code). Also note when and how the alarm occurred.

Shut down the PEA POD by pressing and holding the PEA POD power button for 3 sec and turning switch located on the back panel to the OFF position. In case of a Communication Failure alarm, the PEA POD and computer need to be shut down independently using the PEA POD power button and shutting down the computer using the start menu.

Contact LMI Customer Service and provide the name(s) of the alarm(s) that occurred, associated code(s), tone, and a description of when and how the alarm(s) occurred.

For peer review only - http://bmjopen.bmj.com/site/about/guidelines.xhtml

Results print-outs, export and backup

To avoid transcription error, we thought it would be easier if we print out 2 copies of the results:

One to be stored in the corresponding folder for future reference in the PEA POD room

One to be attached to the result sheet

10.1 Data exports and import into Excel

10.1.1 To Export data

The results are exported to a text file in a tab-delimited format. Further data analysis can then be performed using software programs (e.g. Excel...)

Data management →Export results

Enter password → Click OK

Select Results to Export:

The following filters can be applied: last name, gender, test date, body mass, ID_1, ID_2, ID_3 and/or ID_4. One you selected the filter(s) you want, click apply.

If you don't select any filter and click 'Apply', every results will be listed in the window.

If your search yields more than 200 results, a dialogue box will appear with the following options:

Click 'Back' to further limit the number of records

Click 'Preview' to populate the table (this may take some time)

Click Export to export all records without previewing the data

Modifications to models can be implemented. If you scroll sideways the result list, you will find columns for the default models used. You can change them if you go to their scroll down menu (down arrow). The will not affect the database just the exported results.

Select the results you want to export → Click 'Next'

Name your file → Click 'Next'

The file will be saved in C:\PEAPOD Data\Export Results\

10.1.2 To transfer data

To transfer the data, exit the software and go to the above address or use the shortcut on the desktop. There are 2 USB port in the panel on the left side of the screen. Copy or move your file to a memory stick.

10.1.3 To import data into Excel

In Excel, go to Open.

Select your drive and folder. In the 'Files of type' at the bottom of the 'Open' dialog window, select 'All Files'.

Select your file → Click 'Open' → Click 'Next' → Click 'Next' → Click 'Finish'

The results will appear in an Excel spreadsheet, each column will be a field from the 'Enter Subject information' screen. Each test will occupy a line.

For peer review only - http://bmjopen.bmj.com/site/about/guidelines.xhtml

10.2 Database backup

This activity allows for backup of the contents of the database so that in case of a PEA POD malfunction, data can be restored. Database backup should be based on usage rather than regular time intervals.

Data management → Database Backup

You can modify the name of your file but it will always be appended by its creation date (dd-mm-yyyy). The file will be stored at C:\PEAPOD data\Database backup.

If the database is saved twice in the same day and the name is not changed, the latest one will replace the other.

Click ‘Next’ → Click ‘Finish’

10.3 Correction

If, for some reasons, you have entered wrong subject’s data, you can correct them. The computer will automatically update the results if affected.

Data management → Modify Subject Results → Log-in as administrator → Select Subject’s test (2 steps) → Modify subject’s information → click ‘Next’ → Click ‘Repeat Modify subject Results’ or ‘Finish’ to go back to the main screen.

Enter subject information page

During the automatic volume calibration, a screen will appear where you have to enter the subject’s details. At this point you can either retrieve information on a subject from a previous measurement or enter new details. If you enter as a new subject, a subject that is already in the system, the computer will ask you if you want to merge the 2 files or modify the latest one to differentiate it from the old one. The fields available are the following (* = mandatory).

Click next when finished

Noteworthy: at the conclusion of the volume measurement, results are displayed on screen. They can then be reviewed and some entries modified if incorrect. Proceeding to the next step saves the results and provides the option to print them out.

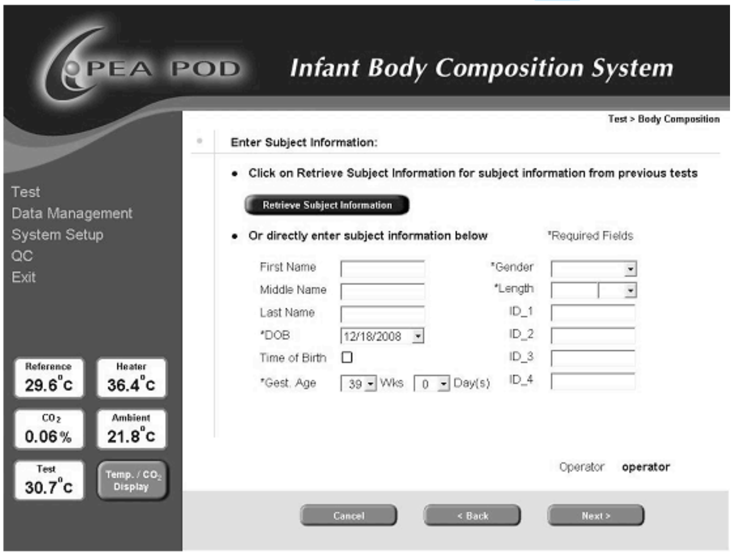

For Peer Review

First name	FGLS
Middle name	
Last name	FGLS number – see list in the PEA POD room
* DOB	DD/MM/YYYY
Time of birth	The software only gives the option of 'finite' hour. So it was decided that:<br>If a baby was born before half past the hour, we would enter as its time of birth the last complete hour and<br>If a baby was born on or after half pas the hour, we would enter as its time of birth the next complete hour.<br>For example, if a baby was born between 1pm and 1.29pm, time of birth is 1pm and if a baby was born between 1.30pm and 1.59pm, time of birth would be 2pm.
* Gest age	Gestational age at birth. Enter weeks and days. If any doubt, please refer to the FGLS list that should be in the room. On that list, the women are arranged by EDD (i.e. 40+0 weeks). Also there should be a wheel available in a folder.
* Gender	Male or Female
* Length	The length has to be measured prior to the test. There should be an infantometer in the PEA POD room. The software gives you the option of unit (cm or inches).
ID_1; ID_2; ID_3; ID_4	We are not using those fields for the moment. We might use them later on under the INTERBIO study to differentiate sub-population (IUGR, Macrosomia...)

12. Other information

12.1 How to refer to the PEA POD

Pediatric Air-displacement plethysmography

→ PEA POD (COSMED USA, Inc., Concord, CA, USA)

Model used: Fomon (or Butte), Software version: 3.1.0

12.2 Scale taring and volume calibration

Remember to check what non-clothing items the baby is wearing and use the duplicate props during volume calibration and scale taring.

Number of hospital tag, umbilical cord clamp, feeding tube, splint, canula...

If the baby had his/her length measured earlier, check which items to babies is wearing but do not undress baby just as yet as you need to execute volume calibration and scale taring. You will be provided with a PEA POD form that you or an anthropometrist should have completed with baby's length. Do not forget to record the information requested.

12.3 Number of people in the room for environment control

Remember to put the 'do not disturb' sign outside on the door

Limit the number of people in the room: 2 experimenters and parents (1 or 2)

If the AC is on, you should turn the A/C off if it is blowing directly on the PEA POD or if it causes the room temperature to fluctuate. If you turn it off during the QC measurements to have a more stable environment, then you should also turn it off during a body composition tests.

For peer review only

12.4 Images of the PEA POD machine

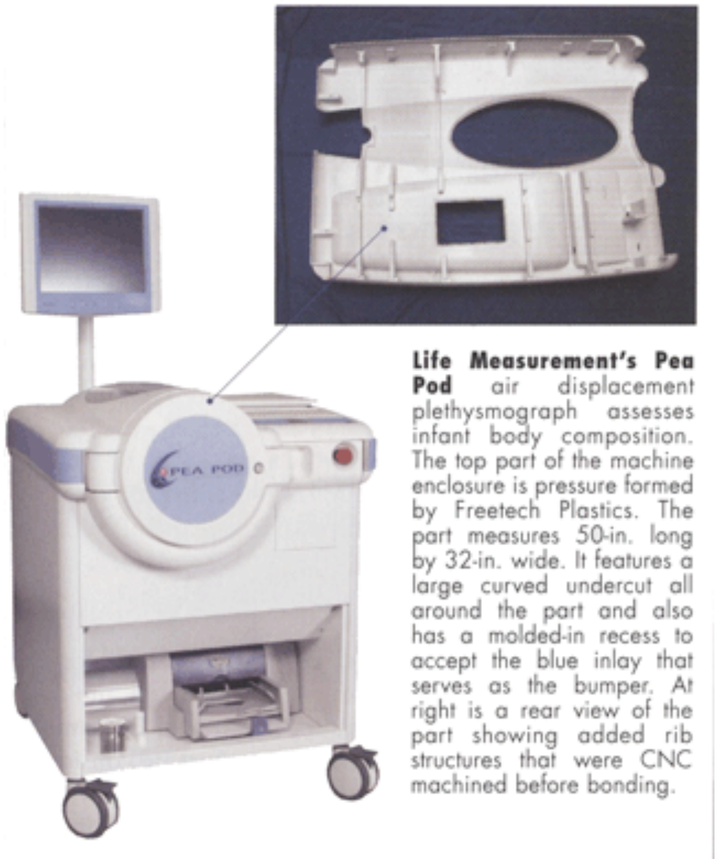

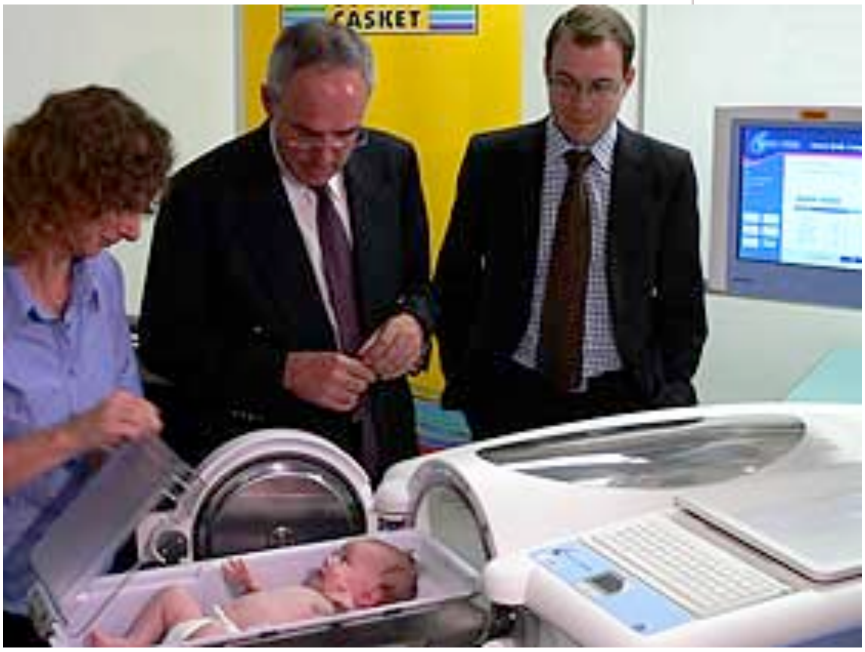

Appendix K: The INTERBIO-21st Study Protocol

The INTERBIO-21st Study

The Functional Classification of Abnormal Fetal and Neonatal Growth Phenotypes

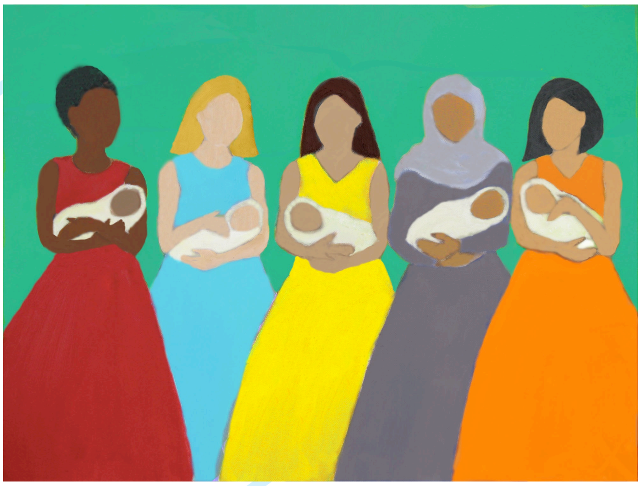

Study Protocol

The INTERBIO-21st Consortium

Oxford Maternal & Perinatal Health Institute (OMPHI)
Nuffield Department of Obstetrics & Gynaecology
University Department of Paediatrics
Wellcome Trust Centre for Human Genetics
and Oxford Tropical Medicine Network
University of Oxford, UK
Global Alliance to Prevent Prematurity & Stillbirth (GAPPS)
in association with:

Universidade Federal de Pelotas, Pelotas, Brazil; **The Aga Khan University Hospital**, Nairobi, Kenya; **Aga Khan University Medical Centre**, Karachi, Pakistan; **KEMRI-Coast Centre for Geographical Medicine & Research,** Kilifi, Kenya; **Shoklo Malaria Research Unit,** Mae Sot, Thailand; **University of Witwatersrand,** Johannesburg, South Africa; **John Radcliffe Hospital**, Oxford, UK.

Version 2, October 2012

For peer review only - http://bmjopen.bmj.com/site/about/guidelines.xhtml

Acknowledgements

This study is supported by a generous grant from the Bill & Melinda Gates Foundation to the University of Oxford, for which we are very grateful.

We acknowledge the enormous contribution made by our co-investigators and collaborating centres worldwide.

We would also like to thank the Health Authorities in Brazil, Kenya, Pakistan, South Africa, Thailand and the UK, who have facilitated the study by allowing participation of these sites as collaborating centres.

Credits

This study protocol is based on a first version produced between May 2010 and January 2011 principally by José Villar, Stephen Kennedy, Aris Papageorghiou and Ann Lambert (University of Oxford), with important contributions from Prof Cesar Victora (Federal University of Pelotas). Sections I-IX and the Appendices were revised in October 2012.

This protocol should be referenced as:
The INTERBIO-21st Consortium,
INTERBIO-21st Study Protocol, Oxford, October 2012.
(www.interbio21.org.uk)

For peer review only

Table of Contents

I.	List of abbreviations used	4
II.	Summary	5
III.	Background	6
IV.	**PROGRAMME I:** INTERBIO-Bank background	8
V.	INTERBIO-21st Fetal Study	11
VI.	INTERBIO-21st Neonatal Study	13
VII.	Selection of cases and controls	15
VIII.	Sample processing	17
IX.	Selection of study centres	19
X.	**PROGRAMME II:** Proof-of-concept study background	20
XI.	Study design	21
XII.	Sample size for epigenetic studies	23
XIII.	Governance of study	25
XIV.	Appendix I: Instructions for Recruiting Cases and Controls for NS	26
XV.	Appendix II: Technical note on selection of controls	27
XVI.	Appendix III: Definition of intrauterine growth restriction in field studies	29
XVII	References	30

For peer review only

List of abbreviations used

**AGA**	Appropriate for Gestational Age
**BPD**	Biparietal Diameter
**BMI**	Body Mass Index
**CRL**	Crown Rump Length
**CI**	Confidence Interval
**DDT**	Dichloro-Diphenyl-Trichloroethane
**DSS**	Demographic Surveillance System
**FGLS**	Fetal Growth Longitudinal Study
**GWAS**	Genome Wide Association Studies
**HC**	Head Circumference
**IQTL**	Imprinted Quantitative Trait Loci
**IUGR**	Intrauterine Growth Restriction
**KDH**	Kilifi District Hospital
**LBW**	Low Birth Weight
**LMP**	Last Menstrual Period
**MeDIP-Chip**	Methylated-Cytosine DNA Immunoprecipitation-Microarray Chip
**MMN**	Multiple Micronutrient
**NICU**	Neonatal Intensive Care Unit
**NCSS**	Newborn Cross-sectional Study
**PPFS**	Preterm Postnatal Follow-up Study
**PCR**	Polymerase Chain Reaction
**RCT**	Randomised Control Trial
**RR**	Risk Ratio
**RT-PCR**	Real Time PCR
**SMRU**	Shoklo Malaria Research Unit.
**SGA**	Small for Gestational Age
**SNP**	Single Nucleotide Polymorphism

**INTERBIO-21st**	The INTERBIO-21st Study: The Functional Classification of Abnormal Fetal and Neonatal Growth Phenotypes
**INTERGROWTH-21st**	The INTERGROWTH-21st Project: The International Fetal and Newborn Growth Consortium for the 21st Century

For peer review only

SUMMARY

The INTERBIO-21st Study aims to evaluate newborn phenotypes so as to understand better the relationship between the causes of IUGR/SGA and preterm birth syndromes. It is based upon our hypothesis, presented in the initial INTERGROWTH-21st Project, that phenotypic subgroups other than those defined by birth weight and gestational age alone are needed to determine a newborn's nutritional status and assess the effectiveness of interventions to prevent and/or treat the effects of an adverse intrauterine environment. In effect, therefore, we are aiming to produce a more "functional" description of these syndromes.

The redefinition of newborn subgroups will arise from evaluating a combination of factors in pregnancies with normal and abnormal outcomes. These factors include maternal health; fetal growth patterns; growth patterns of fetal organs; newborn body composition and physiological function; micronutrient levels and data from epigenetic experiments. We will initially characterise normal genetic variability and normal variability across the epigenome in uncomplicated pregnancies, and compare these data to the variability observed in a sample of high-risk pregnancies. In a series of case-control studies, we will evaluate the effects of adverse environmental and nutritional factors (and other biomarkers), which possibly interact with genetic factors and the epigenome, on the sub-groups of IUGR/SGA and preterm birth.

The rigorous clinical and laboratory-based characterisation of newborn phenotypes and their different aetiologies in relation to morbidities, especially those that are common in resource-poor settings, should lead to better clinical management of pregnancies and newborn complications. This will contribute to the selection of more effective preventive interventions and screening strategies by improving their specificity.

Specifically, we shall:

PROGRAMME I: Create a unique biobank (INTERBIO-Bank) of maternal blood, maternal faeces and cord blood/placental samples from at least six populations with different risk profiles, including women at high risk for preterm delivery and IUGR/SGA because of malnutrition and/or infection. We shall follow a longitudinal and cross-sectional study design in two sub-studies. These samples will be used primarily to explore risk factors and biomarkers for the subgroups of IUGR/SGA and preterm delivery.

PROGRAMME II: Conduct, in the first of a series of experiments, a hypothesis-testing, proof-of-concept study comparing DNA methylation patterns and micronutrient status in term AGA and IUGR/SGA newborns drawn from the INTERBIO-Bank.

Figure 1: INTERBIO-21st Study Flow Diagram

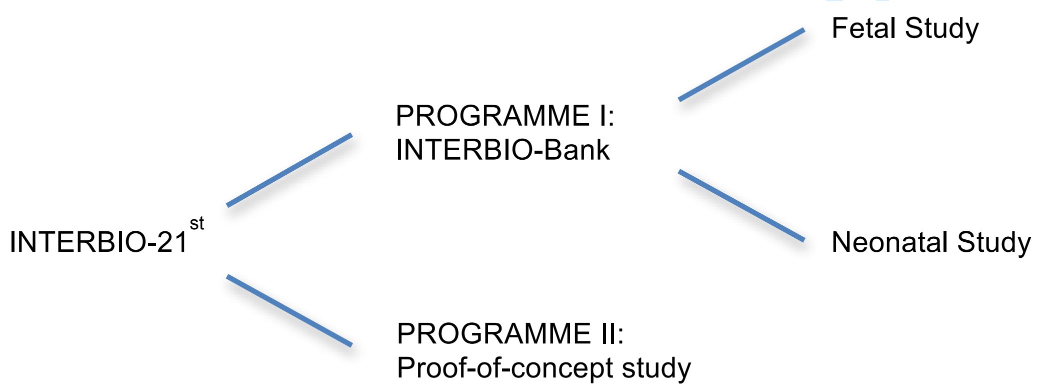

For peer review only

BACKGROUND

The INTERBIO-21st Study builds upon the International Fetal and Newborn Growth Consortium for the 21st Century (INTERGROWTH-21st), a unique, population-based project that is being conducted in eight different geographical locations in Brazil, China, India, Italy, Kenya, Oman, the UK and US. (www.intergrowth21.org.uk).

The primary objective of INTERGROWTH-21st is to develop new "prescriptive" standards, conceptually similar to the WHO Child Growth Standards, describing optimal fetal and preterm neonatal growth and newborn nutritional status, and to relate these to neonatal health risk. This objective is being achieved by implementing three studies involving detailed and highly standardised recording of maternal characteristics and anthropometry, pregnancy complications, exposure to pollutants, fetal growth, neonatal anthropometry and perinatal outcomes:

1. Fetal Growth Longitudinal Study (FGLS): ultrasound and clinical assessment of fetal growth every five weeks throughout pregnancy from <14 weeks, with accurate early pregnancy dating, in eight populations with optimal health, in defined geographical areas with low environmental risks. It will produce ultrasound and clinical Fetal Growth Standards.

2. Preterm Postnatal Follow-up Study (PPFS): follow-up of infants from the FGLS cohort born prematurely with regular anthropometry and nutritional evaluation to describe their postnatal growth pattern up to 2 years. It will produce Preterm Postnatal Growth Standards. All newborns from the complete cohort (FGLS and PPFS) will be seen at 1 and 2 years to evaluate health, nutrition and development.

3. Newborn Cross-Sectional Study (NCSS): anthropometric measures, neonatal morbidity and mortality, and pregnancy complications assessed in all newborns at each of the study centres over a 12 month period, i.e. all deliveries are being captured over 12 months from the same areas. It will produce Newborn Birth Weight for Gestational Age Standards.

The secondary objectives are:

d) **Clinical:** to develop a prediction model, based on multiple 2-dimensional (2D) ultrasound measurements, for estimating gestational age during mid-late pregnancy for use in populations of pregnant women without access to early/frequent antenatal care;

e) **Epidemiological:** to study in this multi-ethnic, population-based sample the determinants of LBW and its components (preterm delivery, impaired fetal growth and their subgroups) under current healthcare conditions, and

f) **Biological:** to acquire additional 3-D images to create an anatomical and growth databank of individual fetal organs as a unique source of biological information for future research.

The study populations from these geographically defined areas have no socio-economic constraints on growth; low morbidity and perinatal mortality, and adequate nutritional status. To be included, women must be non-smokers, with a normal pregnancy history, and without health problems likely to influence fetal growth or indicate a risk for pregnancy-related pathological conditions.

In **FGLS**, women are screened <14^{+0} weeks at their first antenatal visit and followed-up with standard clinical and 2D ultrasound examinations every five weeks, i.e. up to six times during pregnancy. In **PPFS**, preterm infants (> 26^{+0} but < 38^{+0} weeks) born from this sample are being followed-up during their first 8 months of life with the same protocol and set of anthropometric measures used in the WHO Child Growth Study. Postnatal growth is being evaluated from both delivery and conception for comparison with the corresponding *in utero* measurements. All infants from FGLS and PPFS will also be seen at 1 and 2 years to evaluate health, nutrition and development.

In **NCSS**, all newborns at the study centres, born during a fixed 12 month period, have anthropometric measurements taken immediately after birth. Only babies born to women who meet the same inclusion criteria used in FGLS are being selected to construct the newborn standards. Birth weight and gestational age will also be related to neonatal morbidity and mortality outcomes to construct risk-related newborn weight for gestational age standards.

Standard quality control measures are being used, including adaptation of the ultrasound machines to ensure that blinded measurements are taken; a unique system of random evaluation and repetition of ultrasound measurements (from stored images) to monitor validity and reliability, and continuous real time assessment of all data collected. Anthropometric measures of all neonates are being monitored and standardised centrally. All data are entered and managed in an on-line system specifically developed for the study, including a means of transferring blinded data directly from the ultrasound equipment to the database. This allows initiation of data analysis soon after data collection is completed.

For peer review only - http://bmjopen.bmj.com/site/about/guidelines.xhtml

Figure 2: Three INTERGROWTH-21st cohorts

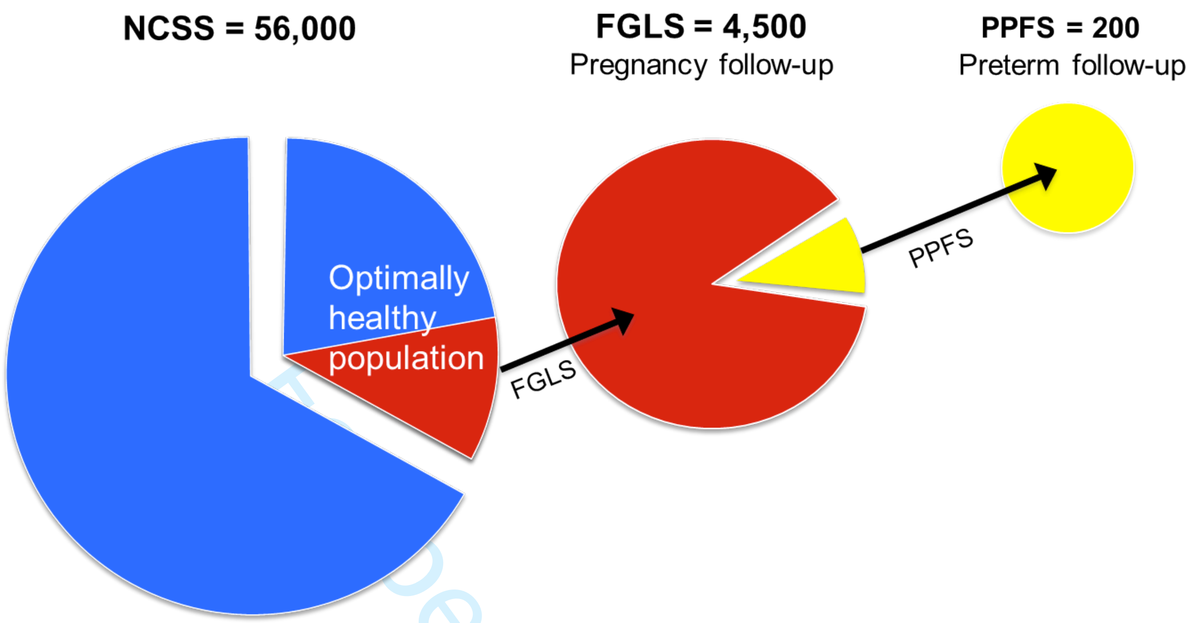

For peer review only

PROGRAMME I. INTERBIO-Bank

Create a biobank of maternal blood and cord blood/placental samples

Background

The aim is to establish a biobank (INTERBIO-Bank) of maternal blood, maternal faeces and cord blood/placental samples from healthy and complicated pregnancies to allow nutritional, epigenetic and other biomarker studies to be performed.

Collecting a heterogeneous group of cases will allow us to explore the wide range of aetiological factors (genetic, metabolic, vascular, autoimmune, infectious etc.) contributing to the development of complicated pregnancies that may present in the same way phenotypically (e.g. low gestational age), as well as the interactions between risk factors and outcomes. Ultimately, we aim to integrate all the pregnancy-related, clinical and biomarker data to improve the phenotypic characterisation of newborns, so as to facilitate the development of targeted interventions and screening strategies in pregnancy and early infant life.[c]

The pathways leading to pregnancy complications, e.g. preterm delivery, IUGR and SGA syndromes, are almost certainly controlled by multiple molecular, genetic, epigenetic and biochemical mechanisms. What is less clear is the relative contributions from risk factors such as infections, nutritional status and other environmental exposures, especially in resource-poor settings.

We hypothesise that:

1. There is more than one preterm delivery phenotype associated with inter-related pathways, i.e. the heterogeneous causes have different functional effects on the fetus/newborn.
2. Similarly, the IUGR/SGA phenotype has several intrauterine growth patterns, multiple causes (e.g. small maternal stature, poor maternal nutrition, infection, prematurity and utero-placental insufficiency), and neonatal and infant outcomes.
3. Hence, it is inappropriate to manage SGA and preterm newborns as single clinical entities, as usually occurs, based on the potentially false assumption that, *irrespective of the cause*, the adverse effects on the fetus and the clinical manifestations in the newborn are uniform;
4. These phenotypes will best be characterised by integrating measures of maternal health, fetal growth patterns, better estimation of gestational age and metabolic function, with biomarker data.

More rigorous clinical and laboratory-based characterisation of such phenotypic subgroups and their different aetiologies should lead to better clinical management of newborn complications and the development of more effective preventive interventions and screening strategies by improving their specificity. This is important because a lack of specificity of interventions tested in previous RCTs, particularly those to prevent preterm delivery, could have resulted in interventions that are actually effective in some phenotypic subgroups, being abandoned because they failed to show an overall protective effect.

A good example is the finding that calcium supplementation in low-risk women with low-calcium diets, significantly reduces the risk of pre-eclampsia (RR 0.48; 95% CI 0.33-0.69) but its impact on preterm birth (RR 0.81; 95% CI 0.64-1.03) borders on significance [1]. However, when the analysis was restricted to the four small RCTs including women at high risk of pre-eclampsia (n=568), there was a large and significant decrease in preterm birth (RR 0.45, 95% CI 0.24 to 0.83) [2]. Hence, it is possible that the magnitude of the effect of supplementation varies because the predominant preterm birth subgroups are different.

Similarly, although malaria infection clearly affects birth weight and gestational duration in epidemiological studies, a Cochrane systematic review of anti-malarial interventions in pregnancy showed that - among women in their 1st or 2nd pregnancies - treatment reduced anaemia, parasitaemia, placental malaria, perinatal deaths and low birth weight (RR 0.57; 95% CI 0.46-0.72), but had no effect on preterm births in the only trial assessing this outcome [2 3]. Thus, anti-malarial interventions may be effective in preventing only a subgroup of preterm births that is not seen when small trials use overall preterm rate as the primary outcome. Lastly, despite the considerable epidemiological evidence that gynaecological infections and bacterial vaginosis are associated with preterm birth, the results of several RCTs of antibiotic treatment of such infections have generally

[c] (Kramer MS, Victora CG Humana (2000); Barros FC, BMC Pregnancy and Childbirth (2010)

For peer review only - http://bmjopen.bmj.com/site/about/guidelines.xhtml

been disappointing [4 5]. However, it is possible that such treatments are still effective in reducing certain subgroups of preterm birth.

In addition, interventions that are phenotype-specific may, in the long-term, prevent the adverse metabolic and cardiovascular consequences of fetal malnutrition in adulthood. This general approach is of special relevance to resource-poor settings where targeting more homogeneous pregnancy and newborn sub-groups could considerably enhance the effectiveness of available resources.

The very thorough and highly standardised characterisation of antenatal events, using the same protocols in all the pregnancies will make this, to the best of our knowledge, the most comprehensive biobank in the world for nutritional, epigenetic and other biomarker studies in pregnancy.

Figure 3: INTERBIO 21st Fetal and Neonatal Studies: data and sample collection periods

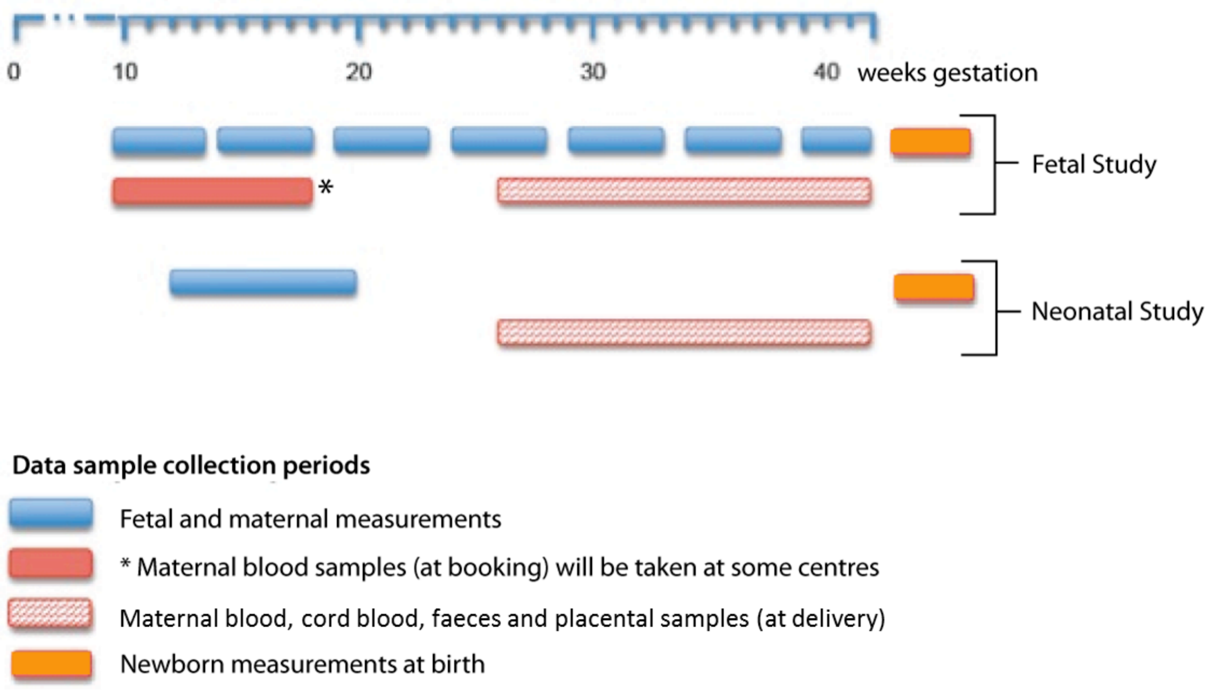

The biobank will be used for studies such as: genetics (SNP genotyping); epigenetics (DNA methylation, histone modification, imprinting, miRNA); expression analyses (mRNA and protein); micronutrient assays; immunohistochemistry; biomarker discovery and validation relating to outcomes such as preterm birth. Anonymised samples will also be made available to other biobanks via a process governed by the Biobank Management Group.

INTERBIO-Bank study design

We aim to collect and store maternal blood, maternal faeces and cord blood/placental samples (see Figure 3) to create a biobank from the following populations:

- 1) "Fetal Study" pregnancies in three centres currently in the INTERGROWTH-21st Project (Pelotas, Brazil; Nairobi, Kenya; Oxford, UK), supplemented by high-risk pregnancies in centres in resource-poor settings, monitored using the same protocol: **INTERBIO-21st Fetal Study**
- 2) "Neonatal Study" pregnancies in the same three centres (Pelotas, Brazil; Nairobi, Kenya; Oxford, UK), supplemented by high-risk pregnancies in centres in resource-poor settings, monitored using the same protocol: **INTERBIO-21st Neonatal Study**

The INTERBIO-21st Fetal Study will provide detailed phenotypic information based on fetal growth patterns and biological samples to investigate maternal/fetal nutritional status and maternal/placental/fetal biomarkers in pregnancies with optimal outcomes, as well as those complicated by a range of factors, including HIV, malaria, malnutrition and anaemia in resource-poor settings. In the field of DNA methylation in particular, this will be an important first step in describing normal variability in fetal/placental methylomes and how methylation signatures relate to both healthy and adverse clinical outcomes.

The INTERBIO-21st Neonatal Study will provide detailed newborn phenotypic information (including accurate gestational age at birth and neonatal morbidity) and biological samples for case-control studies of maternal/fetal nutritional and maternal/placental/fetal biomarkers in healthy pregnancies, as well as those complicated by a range of factors, including HIV, malaria, malnutrition and anaemia in resource-poor settings.

For peer review only

1) INTERBIO-21st Fetal Study: Collect and store maternal blood, maternal faeces and cord blood/placental samples from pregnancies in three INTERGROWTH-21st centres (n=500 per centre), supplemented by samples from high-risk populations monitored using the same protocols in centres in resource-poor settings (n=500 per centre).

In all centres, we plan to collect and store maternal blood, maternal faeces and cord blood/placental samples at delivery (in addition to the pregnancy and fetal growth data) from a total of 2,500 pregnancies (500 per centre). For details of blood, faecal and tissue sample collection see the INTERBIO-21st Operations Manual.

Detailed information will also be acquired about gestational age and fetal growth patterns starting at <14 weeks' gestation. This is of great relevance because of the recent evidence that fetal growth discrepancies, which can be detected by ultrasound as early as the 1st trimester, are associated with increased risks of preterm birth, low birth weight, and SGA at birth [6].

Inclusion criteria for INTERBIO-Bank
INTERGROWTH-21st centres that have already completed FGLS

Women from the entire population attending for antenatal care from <14 weeks' gestation, irrespective of their risk profile for adverse pregnancy/neonatal outcomes, should be recruited for INTERBIO-Bank. However, to participate, women must be at least 18 years old and their pregnancy must have been conceived naturally. Women who have a BMI over 35 must also be excluded from the study as their weight will be a barrier to accurate ultrasound scans. All other women are eligible.

New INTERBIO-21st centres

Women from the entire population attending for antenatal care from <14 weeks' gestation, irrespective of their risk profile for adverse pregnancy/neonatal outcomes, should be recruited for INTERBIO-Bank. However, to participate, women must be at least 18 years old and their pregnancy must have been conceived naturally. Women who have a BMI over 35 must also be excluded from the study as their weight will be a barrier to accurate ultrasound scans. All other women are eligible.

Estimation of gestational age at study entry
Gestational age at study entry will be estimated by ultrasound measurement of CRL <14 weeks. When LMP is available this should also be recorded. This estimation of gestational age by CRL takes into consideration that in a large proportion of very high risk pregnancies the LMP may not be known.

Fetal growth monitoring
After the first scan between 9^{+0} and 14^{+0} weeks, we will perform scans at ~5 weekly (±1 week) intervals. After the dating scan, 6 further visits (for fetal biometry) will be scheduled at ~5 weekly (± 1 week) intervals (i.e. 14-18, 19-23, 24-28, 29-33, 34-38 and 39-42 weeks). Seven measurements will be taken at each visit from 14^{+0} weeks onwards: Biparietal Diameter (BPD); Occipito-Frontal Diameter (OFD); Head Circumference (HC); Transverse abdominal diameter (TAD); Anterio-posterior abdominal diameter (APAD); Abdominal Circumference (AC) and Femur Length (FL). At each visit, the measurements will be obtained 3 times from 3 separately generated ultrasound images in a "blinded" fashion, and submitted electronically (with the associated images) to the Project Coordinating Unit. All the study centres will use equipment with similar characteristics. The staff will be appropriately trained following standardised procedures according to the corresponding FGLS Protocol and Ultrasound Operations Manual.

Pregnancy follow-up
Women in the study will receive standardised antenatal care (with some local variations) based on the recommended WHO package, part of which involves screening for conditions that emerge during pregnancy. All women recruited will be followed throughout pregnancy from the time of the first visit, irrespective of the pregnancy outcome.

Severe perinatal morbidity and mortality outcomes
We have decided to use an un-weighted composite outcome including at least one of the following conditions: stillbirth, neonatal death until hospital discharge of the newborn, newborn stay in Neonatal Intensive Care Unit (NICU) for ≥7 days or other severe neonatal complications. We believe this is a good proxy for adverse perinatal outcomes across countries. We have used it as a primary neonatal outcome in recent publications and it has been well accepted. Its only disadvantage is that it risks excluding, from the total number of early neonatal deaths, some cases amongst healthy, mostly term babies delivered vaginally who, after hospital discharge at 48 hours, develop severe complications or death up to 7 days post-natally without returning to the same hospital. However, missing these isolated cases is preferable to performing thousands of unnecessary home visits.

For peer review only - http://bmjopen.bmj.com/site/about/guidelines.xhtml

2) INTERBIO-21st Neonatal Study: Collect and store maternal blood, maternal faeces and cord blood/placental samples at birth from pregnancies in three INTERGROWTH-21st centres (200 newborns at <38^{+0} weeks' gestation plus 200 controls, and 200 IUGR/SGA plus 200 controls in each centre), supplemented by samples from high-risk pregnancies in resource-poor settings. For details of sample collection see INTERBIO-21st Operations Manual.

NCSS pregnancies in INTERGROWTH-21st are ideal, population-based cohorts for nutritional, epigenetic and other biomarker studies to study the causes of pregnancy complications and how they influence growth and development, principally for the reasons outlined in Box 1.

Box 1: Some unique characteristics of studies conducted using NCSS protocols

Geographically diverse populations

Large, population-based, sample size with severe morbidity and mortality outcomes

Early pregnancy dating by ultrasound provided by small number of standardised operators

Standardised methodology for maternal, newborn and infant follow-up anthropometric measures

Maternal morbidities during pregnancy captured prospectively

Environmental characterisation of the populations and individual participants

However, we recognise the need to enrich the collection of complicated pregnancies from populations with other risk factors that are especially relevant to the needs of developing countries. Therefore, we will supplement sample collection in the three INTERGROWTH-21st centres by also collecting samples from pregnancies from the general population in resource-poor settings where there is a high risk of fetal growth impairment and preterm delivery because of infection, malnutrition, poor socio-economic status and past adverse pregnancy outcomes. This strategy will increase the generation of cases from a relatively small population given the higher incidence of the conditions.

In these centres, we will collect and store samples from 800 pregnancies per centre:

Maternal blood, maternal faeces, cord blood and placental samples will be collected from pregnancies (cases) that have delivered at <38^{+0} weeks gestation (n=200 per centre) or have resulted in IUGR/SGA newborns (n=200 per centre). Newborns that were born at <38^{+0} weeks' gestation and were IUGR/SGA will be included in both sets of cases as the case-control analysis will be carried out separately for each outcome.

We will also collect the same samples from term AGA newborns (controls), i.e. non-IUGR, normal birth weight newborns at term, as a reference group (n=400 per centre, i.e. one control for each case).

All cases and controls are required to have had, reported in their medical records, an estimation of gestational age by ultrasound measurement of either CRL <14 weeks or HC <24 weeks. When LMP is available this should also be recorded. If the LMP is not available it should be recorded as such and ultrasound estimations will be used.

Because of the different populations in the centres selected, all analyses in this case-control strategy will be stratified by centre, and will only be pooled if there is no statistical evidence of heterogeneity.

Anthropometric measurements

All babies, i.e. all cases and controls, born during the study period will have weight, length and head circumference taken within 24 hours of delivery.

Standardised, electronic, digital, newborn weighing scales with a precision of 10g will be used and their calibration status will be checked twice a week; they will be replaced if they are faulty and cannot be repaired. We shall also provide all clinics with standardised infantometers for length (precision 0.1 cm) and tape measures for head circumference (precision 0.1 cm); these will be similarly calibrated and maintained. All anthropometrists will be trained centrally and monitored during the study following standard procedures by the Anthropometric Standardisation Unit; they in turn will train the nurses/midwives in how to apply the study's measurement protocol.

For peer review only - http://bmjopen.bmj.com/site/about/guidelines.xhtml

For a small subgroup, the following additional anthropometric measurements will be taken: arm circumference; thigh circumference; abdominal circumference and skinfold thickness, as well as neonatal body composition using air displacement plethysmography (PEA POD) in some centres.

Follow-up
All newborns during the study period, including those on NICU or special care, will be followed on a daily basis until hospital discharge to document severe morbidity and detect neonatal death. We will make strenuous efforts to coordinate and promote evidence-based care for the neonates born <38^{+0} weeks' gestation using materials developed as part of our best practice programme, by liaising with the lead neonatologist in each NICU before and during the study. We recognise that differences in practice will persist despite our best efforts, especially in resource-poor settings. However, we believe this is unavoidable in a very pragmatic study such as this, which is trying to reflect what happens on a daily basis in clinical practice. Furthermore, we will similarly make strenuous efforts to standardise the main protocols for feeding practices in each NICU before the study starts. During the routine site-visits by members of the Study Coordinating Unit and the Anthropometric Team we will monitor the implementation of the protocols.

Severe perinatal morbidity and mortality outcomes
We have decided to use an un-weighted composite outcome including at least one of the following conditions: stillbirth, neonatal death until hospital discharge of the newborn, newborn stay in NICU for ≥7 days or other severe neonatal complications. We have used such an outcome recently [7 8]; it requires limited standardisation of clinical diagnoses across hospitals and is well accepted as a marker in large, international, population-based studies of newborns that are severely ill.[d] It could be argued, however, that intrapartum stillbirth may not be related to fetal growth and should not be included in this index. We believe this is a valid point but as it will not be possible to separate those intrapartum deaths that are related to IUGR from those that are unrelated, we suggest keeping the index as it is. We believe this is a good proxy for adverse perinatal outcomes across countries.

On-line data management and statistical analysis
All clinical data will be entered into an on-line data management system specifically developed for the study. It includes a method for direct transfer of blinded data from the ultrasound machines to the database. This on-line system has the practical benefit of allowing on-going quality control, correction of errors or missing values and the initiation of data analysis soon after data collection is completed. It will be used for data management and monitoring all sub-studies, including patient recruitment and follow-up, and is based on the INTERGROWTH-21st Electronic Data Management System. The system permits all participants' data to be incorporated into the data files via the Internet as soon as they are available. Included within the system is a review process to ensure that all data are complete.

All sample related data will be entered separately into a data management system specifically developed for the study. The system allows samples to be tracked from the time of collection through processing, storage in the participating centres, and transport to a centralised facility. Each participant will have a unique identifier number, which will be used to link the clinical and sample databases. The number will also be used to barcode individual samples and aliquots. Quality control for this aspect of the study will be monitored by a team from GAPPS.

These systems will provide the Data Management Unit with a detailed daily record of patient enrolment and data entry, at both individual and institutional levels to monitor progress against the milestones listed in the protocol. Corresponding actions, such as telephone calls, web conferences and site visits will take place within a week of detecting a problem in a centre to ensure that appropriate corrective measures are taken.

[d] Others have also used these composite indices of neonatal morbidity (Hannah ME, Hannah WJ Kewson SA et al (2000); Wapner RJ, Sorokin Y, Thom EA (2006); Joseph KS, Fahey J, Platt R (2009)).

For peer review only - http://bmjopen.bmj.com/site/about/guidelines.xhtml

Selection of Cases and Controls All live and stillborn infants in the study hospitals during the data collection period, whether or not they survive until hospital discharge, will be screened. However, multiple births and post-term births (>42^{+0} weeks), will not be included.

Each newborn infant will fall into one of the four groups below:

Set	Infants born <38^{+0} weeks' gestation	Infants born IUGR/SGA	Description	Number of births at study site	Number to be included in the case-control study
A	Yes	No	Non-IUGR/SGA infants born <38^{+0} weeks	A	A (all)
B	No	Yes	IUGR/SGA infants born ≥38^{+0} weeks	B	B (all)
C	Yes	Yes	IUGR/SGA infants born <38^{+0} weeks	C	C (all)
D	No	No	Non-IUGR/SGA infants born ≥38^{+0} weeks	D	Sample = A+B+C

All mothers admitted for delivery (spontaneous or induced labour, or elective C-section) will be screened to check if they had gestational age estimated by CRL at <14 weeks or HC at <24 weeks. If not, they are not eligible for the study. If a mother had one or both of these two measurements, the screening form will be completed to collect the information required to classify her infant as: a) <38^{+0} weeks or ≥38^{+0} weeks, and b) IUGR/SGA or non-IUGR/SGA (based on the charts provided).

Operational definition of cases and controls in the maternity wards

To simplify the identification of cases and controls during screening, the following procedures will be used (see instructions in Appendix II):

First, gestational age will be assessed using CRL or HC. **Cases, born at <38^{+0} weeks, will be live or stillborn infants with gestational age assessed by an early ultrasound (either CRL at <14 weeks or HC at <24 weeks)**, regardless of whether or not they presented with IUGR/SGA at any time during pregnancy or at birth. These infants correspond to groups A and C in the table above.

Second, BW for gestational age will be assessed for infants born ≥38^{+0} weeks. **Cases, IUGR/SGA, will be live or stillbirths whose BW for gestational age is below the 10th centile of the INTERGROWTH-21st neonatal standard as defined on the form.** These infants correspond to group B in the table above. In the data analysis phase, infants from group C (IUGR/SGA infants born <38^{+0} weeks) will be added to those in group B so as to include all IUGR/SGA infants regardless of their gestational age at birth.

Third, the screening form will also identify **potential controls, that is, non-IUGR/SGA infants who were not born <38^{+0} weeks** (group D in the table above). The first potential control born after each case (either a case born <38^{+0} weeks or an IUGR/SGA case) in the same hospital[e] will be enrolled in the study as a control. After enrolling a case, a control must be recruited. If two cases are born in succession, the second case cannot be recruited and instead screening for a control continues. Once a case-control pair have been recruited and processed, sites then screen for another case.

At each site, 200 cases born <38^{+0} weeks and 200 IUGR/SGA cases will be recruited, along with 400 controls. If a site collects 200 cases born <38^{+0} weeks before it has collected 200 IUGR/SGA cases, it will stop recruiting cases born <38^{+0} weeks and their corresponding controls, and will continue recruiting IUGR/SGA cases until 200 (and their controls) have been recruited - and vice-versa, if the quota of 200 IUGR/SGA cases is collected before 200 cases born <38^{+0} weeks are enrolled.

[e] If there is more than one hospital at a given study site, and if presumed risk factors vary by hospital (e.g. one primarily attracts mothers of low socioeconomic status, and another attracts high income mothers), it may be necessary to weight the analyses to reproduce a control group that is representative of the study population; ignoring such differences may lead to overmatching.

For peer review only - http://bmjopen.bmj.com/site/about/guidelines.xhtml

Note that the only criteria for matching cases and controls are: a) hospital of birth and b) approximate date of birth (usually same day, sometimes the next day if there are no controls on the same day).

Figure 4: Neonatal Study Eligibility Flow Diagram

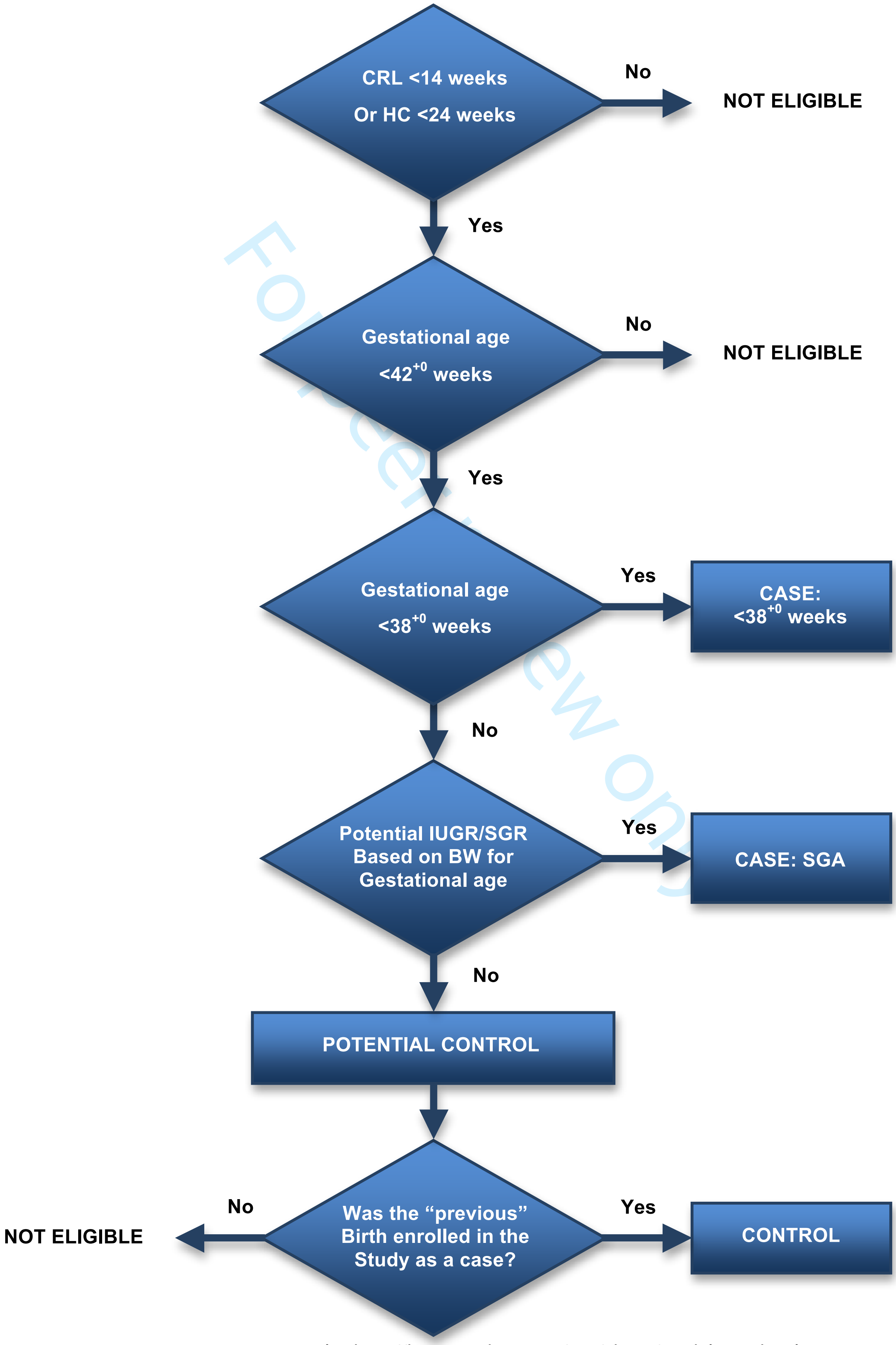

For peer review only - http://bmjopen.bmj.com/site/about/guidelines.xhtml

Definitions of cases and controls for the data analyses

Cases born <38^{+0} weeks' gestation for the data analyses will include all births at <38^{+0} weeks whether or not they present with IUGR/SGA (groups A and C).

IUGR/SGA cases for the data analyses will include the operational definition of IUGR/SGA cases (group B) plus those cases born <38^{+0} weeks who are also IUGR/SGA (group C); the latter were collected as a sub-set of cases born <38^{+0} weeks.

Infants in group C (IUGR/SGA infants born <38^{+0} weeks) will be included in both groups of cases, as the case-control analyses will be carried out separately for each outcome.

The table below provides the definition of controls for the analyses.

Controls for cases born <38^{+0} weeks will be a sample of live and stillborn infants born ≥38^{+0} weeks. In the statistical analyses, a proportion of term IUGR/SGA (xB) cases will be added to the operational controls (group D).

Controls for IUGR/SGA cases will be a sample of live and stillborn infants who are not IUGR/SGA at birth. In the analyses, they will include all operational controls (group D) plus a proportion of infants born <38^{+0} weeks who are not IUGR/SGA at birth(xA).

Table 2. Case-control comparisons in the data analyses.

Comparison	Cases	Controls	Comments
Infants born <38^{+0} weeks case-control study	A + C	D + xB	To reproduce the control population, set B (IUGR/SGA only) will be down-weighted by a factor x which is equal to the sampling fraction for set D, that is the proportion of all infants in the control pool who were included in the detailed study (cases).
IUGR/SGA case-control study	B + C	D + xA	As above, for set A (infants born <38^{+0} weeks only).

Subgroup analyses will include cases born <38^{+0} weeks, stratified according to: a) gestational age groups (the exact groupings will be decided based on the number of births each week of gestational age, so that there will be at least 100 cases in each sub-group) or b) by preterm phenotype, using the newly proposed INTERGROWTH-21st classification system. For IUGR/SGA, subgroup analyses will include stratification by: a) IUGR/SGA severity (<3rd, 3-5th, 6th-9th centiles) and b) gestational age.

Appendix II provides more detailed information on different strategies for selecting controls for case-control studies than we considered when planning the study, but some of these proposed strategies were not practical. As proposed above, INTERBIO-21st will adopt a traditional case-non-case design, and odds ratios will be used to estimate relative risks. This is based on the assumption that cases will be relatively rare, i.e. <10% of the overall number of births.

We estimate that the overall birth rate for infants born <38^{+0} weeks will be <10% and the overall IUGR/SGA rate will be <10-15%. However, by collecting data on all four sets (A, B, C and D), it will also be possible, with appropriate statistical weights in the analyses, to carry out case-base analyses using Poisson regression with robust variance, if the outcomes end up being more common (>10%).

Sample processing

The sample collection, processing and storage procedures will be performed in a standardised manner based on protocols described in detail in the INTERBIO-21st Operations Manual that has been developed with the assistance of the GAPPS team, and researchers at the Universities of Oxford [9] and Cambridge [10], and Johns Hopkins Bloomberg School of Public Health.

In brief, maternal and cord blood samples will be collected to store whole blood, plasma and the buffy coat for a wide range of purposes, including DNA extraction for genetic and epigenetic studies and micronutrient assays. Two placental biopsies will be taken for immunohistochemistry and DNA extraction and in RNA later for expression studies (if the sample is obtained <30 mins after delivery). In addition, we intend to collect and store samples for a number of future, as yet unspecified, biomarker assays relating to preterm delivery and fetal growth.

It is vitally important to ensure that samples are collected in a standardised way with adequate monitoring of quality control, principally because sample quality, quantity and handling can greatly influence the results of microarray and sequencing experiments [11].

For peer review only - http://bmjopen.bmj.com/site/about/guidelines.xhtml

The primary reason (aside from quality control) for ensuring that samples are collected, stored and processed in a uniform manner is to facilitate the anticipated interchange of data, in the future, with other biobanks. Standardising phenotypic definitions, sample collection methods and analyses fosters trans-national collaboration and networking [12]. We shall therefore also seek advice from groups such as the Public Population Project in Genomics (http://www.p3g.org), which promotes international harmonisation and collaboration in population genomics and biobanking by sharing research tools and expertise.

Faecal samples: We wish to collect a faecal sample from mothers, opportunistically at the time of delivery, for metabiomic studies. Although it has been suggested that we should also collect stool samples from infants every 6 months, we feel that this is a rather large-scale undertaking that is beyond our remit.

We certainly appreciate the importance of looking for maternal intestinal co-infections and microbiota. In fact, we published on this subject in 1989: in a prospective study of 14,914 pregnant Guatemalan women, the incidence of IUGR increased with the number of parasitic species detected [13].

Sample size

This is a great challenge in any field-study of this magnitude and even more difficult when exploring risk factors with relatively unknown degrees of association and prevalence in the population. The key issue is to reach a balance between logistical demands, including the need to maintain data quality in these populations, and power calculations especially for the planned epigenetic studies. Having said that, our co-investigators, Krina Zondervan and Cecilia Lindgren in the Wellcome Trust Centre for Human Genetics, Oxford, have considerable experience of conducting candidate gene and genome-wide association studies (GWAS) in related fields and the lessons learned over the last 15 years will be pertinent to the proposed studies.

To illustrate the point, Cardon & Zondervan reviewed how the complex interplay between genotype, phenotype, environmental factors and sample size affects the ability to detect disease susceptibility variants in population-based association studies [14]. They concluded that thousands of cases and controls are required to detect common variants with small effect sizes in such studies.

Three examples demonstrate the need to study large numbers to identify genes influencing quantitative traits involved in metabolic function, such as birth weight. Nearly 120,000 individuals were genotyped to identify three loci influencing anthropometric measures (waist circumference and waist-hip-ratio) of central obesity and fat distribution in a recently published meta-analysis of 16 GWAS, followed by large-scale replication testing [15]. Using similar methodology (GWAS meta-analysis followed by replication), over 90,000 individuals were genotyped to confirm that two loci are associated with BMI and to identify six additional loci [16] and, more recently, nearly 40,000 European individuals were genotyped in identifying variants in *ADCY5* and near *CCNI* associated with fetal growth and birth weight [17].

For the nested case-control studies, we are collecting samples from 2,000 controls; and 2,000 cases from pregnancies with adverse outcomes, e.g. delivery at $<38^{+0}$ weeks' gestation, term IUGR/SGA. In addition, we have the potential to include 400 cases and 2100 controls from the FGLS population in the analysis, taking into consideration the possibility of selection bias in the selection of FGLS population controls.

It is very unlikely that fewer than these numbers will be needed to study the effects of adverse intra-uterine effects on epigenetic profiles, especially as there is emerging evidence from genome-wide epigenetic studies in animals that imprinted quantitative trait loci (iQTL) affect body weight and growth [18] and adult body composition [19] in much more complex and diverse patterns than previously assumed.

Selection of study centresWe aim to use the same rigorous processes to select the new sites for this extension as originally adopted in the selection of the current INTERGROWTH-21st centres. However, in this case, the selection criteria will inevitability involve finding a balance between obvious opportunities (e.g. having access to a malnourished pregnant population with a high prevalence of malaria/HIV) and the risks of working in a research naïve environment with limited existing access to antenatal care.

The criteria the INTERBIO-21st Steering Committee will use to select the centres will include factors such as: 1) existing research infrastructure and capacity; 2) existing maternity services, including antenatal ultrasound; 3) support of local health authorities; 4) previous experience in collecting biological samples; 5) geographical location to retain global coverage; 6) prevalence of key exposure variables, i.e. risk factors; 7) costs; 8) leveraged funding from other donors, and 9) need ideally for all samples in the proof-of-concept study to be analysed in a centralised facility.

For peer review only - http://bmjopen.bmj.com/site/about/guidelines.xhtml

Staged introduction of sample collection at likely study sites

Phase I

Shoklo Malaria Research Unit, Mae Sot, Thailand

KEMRI-Coast Centre for Geographical Medicine & Research, Kilifi, Kenya

John Radcliffe Hospital, Oxford, UK

Phase II

The Aga Khan University Hospital, Nairobi, Kenya

Aga Khan University Medical Centre, Karachi, Pakistan

University of Witwatersrand, Johannesburg, South Africa

Universidade Federal de Pelotas, Pelotas, Brazil

PROGRAMME II: Proof-of-concept study

Background

Understanding the gene-environmental interactions underlying the plasticity of the epigenome at certain times from fetal life to infancy will be crucial to developing interventions, particularly in pregnancy, that might correct or at least prevent the long-term, adverse consequences [20]. We believe that the key to doing so effectively is to recognise that phenotypes other than birth weight and gestational age alone are needed to determine the nutritional status of the newborn and assess the effectiveness of interventions.

The redefinition of newborn phenotypes will arise from evaluating a combination of factors in pregnancies with normal and abnormal outcomes. These include maternal health; fetal growth patterns measured using 2D ultrasound; growth patterns of individual fetal organs measured using 3D ultrasound; newborn body composition and physiological function; micronutrient levels and data from epigenetic experiments, which will initially characterise normal variability across the epigenome in uncomplicated pregnancy and then, in carefully designed nested case-control studies, evaluate the effects of adverse environmental and nutritional factors on the epigenome (and other biomarkers) in a pool of complicated and uncomplicated pregnancies.

General Objectives

The aim is to conduct a hypothesis-testing, proof-of-concept study comparing 500 normal birth weight and 500 term IUGR/SGA newborns (using both cord blood and placental samples) taken from the samples collected in the context of both the INTERBIO-21st Fetal and Newborn Studies. This will be the first in a series of experiments utilising samples collected for the INTERBIO-Bank.

We aim to assess DNA methylation patterns in ~100 imprinted genes previously implicated in fetal growth. Our hypothesis is that maternal micronutrient deficiency, particularly of folate and other methyl donor factors, results in impaired fetal growth, development and pregnancy outcomes, through altered DNA methylation.

We will therefore correlate these methylation patterns with pregnancy (clinical outcomes, fetal growth), nutritional (micronutrient assays), and neonatal (growth, development and body composition) data, which will allow us to:

1. Study the effects of environmental and nutritional factors on the epigenome;
2. Develop new phenotypic definitions of LBW and other adverse pregnancy outcomes

If validated, the results could inform knowledge-based actions to address underlying problems, such as poor nutrition and infection, leading to improved outcomes. The data will, in addition, serve to define normal variability in the epigenome and inform the design of future epigenome-wide studies, once the cost has fallen, as inevitably it will with technological advances.

In the long-term, we would also wish to correlate these epigenetic findings with single nucleotide polymorphism (SNP) genotyping data from a GWAS given the increasing evidence that epigenetic regulation is influenced by genetic factors and the recently published data implicating variants in *ADCY5* and near CCNI with fetal growth and birth weight [17].

For peer review only - http://bmjopen.bmj.com/site/about/guidelines.xhtml

Specific Objectives

We plan to study the methylation profiles of the ~100 imprinted genes that have to date been implicated in fetal growth, although the final list of candidate genes will be taken from our own systematic search of the literature, as well as existing databases, such as http://www.geneimprint.com and http://igc.otago.ac.nz.

Where possible, we will analyse cord blood and placental tissue separately to compare the methylation profiles of both tissues. The underlying rationale is as follows:

- There is increasing evidence that placental function and gene expression respond to, and are marked by, environmental insults. The placenta can therefore serve as a 'record of *in utero* exposure and pathology' [21]. Effects on the fetus almost certainly occur downstream of these events and so comparing the epigenetic profiles of both tissues in individual pregnancies may help to differentiate the various causes of IUGR/SGA and preterm delivery.
- Alterations in DNA methylation in humans appear to be tissue-specific:
    - Katari et al. (2009) have reported significantly different DNA methylation levels at specific CpG sites between cord blood and placenta [22].
    - Guo et al. (2008) have described similar findings in two imprinting clusters: the H19 promoter is unmethylated and IGF2 DMR2 hypomethylated in placenta. However, in cord blood, these two regions maintain the differential methylation status seen in most other tissues [23].
    - Yuen et al. (2009) have observed DNA methylation of the promoter in *TUSC3* and *WNT2* in placental, and not the associated fetal, tissues; within individual placentas, methylation was confined to trophoblastic chorionic villi, and not amnion, chorion, cord or decidua [24].

Study design

For this proof-of-concept study, we will randomly select 500 term IUGR/SGA cases from the INTERBIO-Bank. The 500 normal birth weight controls will be taken either from the population at least risk within the INTERBIO-21st Fetal Study or from the total with normal outcomes from the entire study population, and matched with the cases. A final decision will be made by the INTERBIO-21st Steering Committee.

Methods

We have given considerable thought to the best technological platform for assessing methylation profiles and we have consulted widely with leading experts in the scientific community and industry. There are a large number of different platforms available and many more being developed; in general, there is an inverse relationship between the cost of analysis and the resolution/coverage of the genomic region being studied. At this stage, however, we have decided to use Methylated-Cytosine DNA Immunoprecipitation-Microarray Chip (MeDIP-Chip) followed by bisulfite-(BS) PCR and high throughput sequencing for validation of differentially methylated loci [11].

The approach is well described in a recently published proof-of-concept study assessing whether 'DNA methylation in a subset of genomic loci may connect end-stage cardiomyopathy with different etiologies' [25]. In brief, these authors performed a preliminary analysis using MeDIP-Chip (Nimblegen, WI, US); validated differential methylation loci by BS-PCR and high throughput sequencing; identified three angiogenesis-related genetic loci that were differentially methylated with the BATMAN algorithm [26], and using quantitative RT-PCR, found that the expression of these genes differed significantly between cardiomyopathy hearts and normal controls.

However, we are aware that the samples may not be analysed for at least another two years by which time the technology is likely to have changed considerably, costs will have fallen and genome-wide profiling in large numbers of samples will be affordable. We are therefore in preliminary discussions with a number of companies, including Nanopore (Oxford, UK), http://www.nanoporetech.com, and Pacific Biosciences (Menlo Park, CA, US), http://www.pacificbiosciences.com, who may soon be able to offer high-throughput, single molecule sequencing [27]. Whichever platform is used, however, the intention ideally is to analyse all samples in a centralised facility; in fact, this applies to all the experiments proposed in the proof-of-concept study.

Specific experiments

Placenta v. cord blood methylation profiles: To the best of our knowledge, no study has compared the methylation profiles of the ~100 imprinted genes in placental tissue and cord blood. The outcomes of these comparisons will potentially shed light on the regulatory mechanisms and epigenetic profiles of adverse and healthy pregnancy outcomes.

For peer review only - http://bmjopen.bmj.com/site/about/guidelines.xhtml

Cases v. controls methylation profiles: The results of the placenta v. cord blood studies will help to determine which sample sets are compared in trying to identify the methylation profiles associated with adverse pregnancy and newborn outcomes. Comparisons will also be made between ethnic sub-groups. All the above experiments will be performed in duplicate with adequate quality control measures,

Sample pooling for methylation profiles: Pooling samples of 'healthy' controls to act as a reference standard for epigenetic studies has been proposed in the literature [11]. Given that FGLS provides an ideal opportunity to use samples from newborns whose intra-uterine growth has been optimal, we plan to explore this possibility with FGLS samples drawn from the three INTERGROWTH-21st centres. This might involve pooling samples collected both within and across these centres, although the experiments would need to be performed in India if samples are collected there.

Placental expression analyses: We will follow the same experimental design outlined in the Movassagh et al. (2010) study [26]. Quantitative real-time PCR will be performed for target genes, selected from the methylation studies, using validated Taqman Gene Expression Assay primers (Applied Biosystems, Foster City, CA) normalised against house-keeping gene data. In the long-term, we also plan to characterise global expression patterns in placental tissue using the new Illumina HT-12 v4 expression chip, for comparison between sub-groups and methylation profiles, as well as between normal and adverse pregnancy and newborn outcomes.

Nutritional status

To supplement the epigenetic studies above, we will also assess the nutritional status of the 500 cases and 500 controls selected for the proof-of-concept study, by measuring:

- Micronutrients in maternal blood at booking and cord blood at delivery
- Putative markers of methyl donation, e.g. S-adenosylmethionine (SAM) to S-adenosyl-homocysteine (SAH) ratio
- Neonatal body composition

The rationale for adding these measures is that they should facilitate the interpretation of the epigenetic data and the characterisation of specific sub-phenotypes, in particular IUGR and SGA.

Micronutrient assays: As with the epigenetic studies, there are a large number of technological platforms available to assay micronutrients and some controversy regarding the most appropriate ones to measure. The assessment of micronutrients in mother's blood is made even more complex by physiological alterations such as haemodilution and the hyperlipidaemic state of pregnancy [28 29].

We will therefore seek guidance from the Biomarker Group consisting of experts in the field before finalising the list of analytes and the methods to use. We will also draw heavily on the expertise of our collaborators at SMRU, Thailand who have considerable experience of assessing nutritional status in their populations. At present, based on unpublished data from their studies and our reading of the literature, the following analytes have been proposed as candidates to measure:

- Retinol Binding Protein (RBP)/Vitamin A
- Iodine (maternal) and TSH (newborn)
- Ferritin and Soluble Transferrin Receptor (sTfR) – markers of Fe deficiency
- Zinc protoporphyrin (ZnPP)
- Folate, thiamine, choline and zinc
- Vitamin D
- CRP and αGP

In Thailand, we will also measure Dichloro-Diphenyl-Trichloroethane (DDT) metabolite levels as DDT was used as an insecticide for malaria control in Northern Thailand until it was replaced by Deltamethrin in 2000. However, high serum DDT residues, which affect serum retinol levels and probably thiamine as well, are still detected in pregnant women living in the Mae La camp [30] We also plan to ask the local investigators to identify other possible chemical exposures to measure. Final decisions about which exposures to measure and where the samples will be analysed will be made by the Biomarker Group; however, we will ideally use centralised facilities.

Neonatal body composition: As part of our Wellcome Trust/EPSRC funded research program, we are already starting to measure neonatal body composition in: a) term normal birth weight, b) preterm and c) term IUGR/SGA infants enrolled in the UK component of FGLS and PPFS. To do so, we are using an infant-sized, air-displacement plethysmograph (PEA POD Infant Body Composition System, Life Measurement, Concord, CA, US). The study is being conducted so as to correlate fetal growth patterns with better measures than birth weight alone, i.e. the relative contributions of body fat, lean tissues and bone, all of which are key indicators of the adequacy of intra-uterine nutrition.

For peer review only - http://bmjopen.bmj.com/site/about/guidelines.xhtml

The PEA POD system compares well with the 4-compartment reference model, which is considered the best choice for assessing body composition in humans. In contrast, however, it is easy to perform; takes only a few minutes to complete; infant movement during the measurement is not a significant problem; the measurements can be repeated as frequently as needed, and the results are immediately available [31].

The system is now recognised as an established method to assess neonatal body composition in developed countries [32-34], and it is suggested that it may offer important insights into which fetal growth parameters most closely reflect the generalised nutritional state of neonates and infants [34]. However, there are no published data about its use in resource-poor settings as, to the best of our knowledge, the system has been installed in only one site in such a setting, as part of a collaboration between Jimma University, Ethiopia, and the Department of Human Nutrition, University of Copenhagen.

We now propose installing PEA POD systems in four of the centres in resource-poor settings to give a much more detailed assessment of nutritional status and growth than birth weight and gestational age alone.

Sample size for epigenetic studies

As discussed on page 18, it is extremely difficult to provide reliable power calculations at the moment for epigenetic studies: the field is too new and very few relevant studies have been conducted, especially in humans, to enable power calculations to be performed. It is also unclear at present to what extent it will be necessary to map DNA methylation at high resolution across the entire genome [35], which will inevitably influence the epigenotyping strategy and choice of platform, e.g. bisulfite sequencing or array-based technology. However, having said that, the sample size chosen matches that in the NIH National Standard for Normal Fetal Growth Study and we feel comfortable that it provides a reasonable compromise between cost, expediency and logistical demands.

The estimated samples sizes required to detect the effects of methylation status on adverse pregnancy outcomes are inevitably based on a range of assumptions, since the spectrum of methylation changes and their corresponding effect sizes are unknown. Table 1 shows the sample sizes required to detect differential methylation in cases vs. controls. The following assumptions are made:

- Methylation status is either on/off, and so the proportion of cases vs. controls with methylated status is analysed.
- Methylation proportion among controls of 0.2, with proportion in cases varying from 0.3-0.5, corresponds to an odds ratios (OR) of the effect of methylation status on outcome from 1.7-4.0.
- A significance threshold α of 5.0×10^{-4} (Bonferroni-corrected threshold for 100 candidate imprinted genes) vs. 5.0×10^{-7} (commonly applied genome-wide significance threshold in GWA studies [36]).
- Power of 80% vs. 90%
- Case: control ratio either 1:1 or 1:3

For peer review only - http://bmjopen.bmj.com/site/about/guidelines.xhtml

Table 1. Sample sizes* to detect differential methylation status between cases and controls

				Sample size for candidate gene study (α=5.0x10^{-4})		Sample size for genome-wide study (α=5.0x10^{-7})	
	methylation proportion among controls	methylation proportion among cases	OR (PAF)**	Ca:Co 1:1	Ca:Co 1:3	Ca:Co 1:1	Ca:Co 1:3
Power=80%	0.2	0.3	1.71 (0.12)	719	459	1313	913
		0.35	2.15 (0.19)	342	215	623	388
		0.4	2.67 (0.25)	204	127	370	260
		0.45	3.27 (0.32)	137	85	248	151
		0.5	4.00 (0.38)	99	61	179	125
Power=90%	0.2	0.3	1.71 (0.12)	847	559	1512	966
		0.35	2.15 (0.19)	412	263	716	451
		0.4	2.67 (0.25)	245	155	425	265
		0.45	3.27 (0.32)	164	103	284	175
		0.5	4.00 (0.38)	118	74	204	126

* Sample size for cases is given. Calculations include a continuity correction allowing for normal approximation of the binomial distribution.
** OR = odds ratio; PAF = population attributable fraction

Table 1 demonstrates the approximate power of the proposed experiments with 500 cases and 500 controls. However, for future experiments, based on conservative estimates (OR=2.2 and PAF=0.2), we will have considerable power to detect differences even for 90% power, given that we could have a 1:3 case: control ratio (i.e. 1,000 infants born at <38^{+0} weeks' gestation or 1,000 term IUGR/SGA newborns and at least 3,000 term, non-IUGR/SGA controls). Nevertheless, it is worth emphasizing that these are approximate calculations and that, in a study of this magnitude and complexity, logistical and budgetary considerations must inevitably play an important role in the selection of the sample size.

Data quality: Standardisation of the research staff, who will be responsible for obtaining the neonatal body composition data, represents a challenge. However, we will employ the same quality control measures that are now being used in FGLS and PPFS for the ultrasound and anthropometric data to ensure that the quality of the data is maintained.

Publications and Authorship

The policy regarding publications arising from the study is identical to that in place for the INTEGROWTH-21st Project as a whole and was approved at the first INTERBIO-21st Study Steering Committee.

For peer review only

STROBE Statement—Checklist of items that should be included in reports of *cohort studies*

Name of Study: The INTERGROWTH-21st Project International INTER-NDA standards for child development at two years of age: An International Prospective Population-based Study

Corresponding author: Michelle Fernandes, E: michelle.fernandes@wrh.ox.ac.uk

	Item No	Recommendation	Provided	Pg. No. references
**Title and abstract**	1	(*a*) Indicate the study's design with a commonly used term in the title or the abstract	✓	1
		(*b*) Provide in the abstract an informative and balanced summary of what was done and what was found	✓	1
**Introduction**				
Background/rationale	2	Explain the scientific background and rationale for the investigation being reported	✓	4-5
Objectives	3	State specific objectives, including any prespecified hypotheses	✓	1, 4-5
**Methods**				
Study design	4	Present key elements of study design early in the paper	✓	1, 4-5, 6
Setting	5	Describe the setting, locations, and relevant dates, including periods of recruitment, exposure, follow-up, and data collection	✓	1, 4-5, 6
Participants	6	(*a*) Give the eligibility criteria, and the sources and methods of selection of participants. Describe methods of follow-up	✓	1, 6
		(*b*) For matched studies, give matching criteria and number of exposed and unexposed	n/a	n/a
Variables	7	Clearly define all outcomes, exposures, predictors, potential confounders, and effect modifiers. Give diagnostic criteria, if applicable	✓	1, 7
Data sources/ measurement	8*	For each variable of interest, give sources of data and details of methods of assessment (measurement). Describe comparability of assessment methods if there is more than one group	✓	7, 8, 9
Bias	9	Describe any efforts to address potential sources of bias	✓	7, 8, 9
Study size	10	Explain how the study size was arrived at	✓	8
Quantitative variables	11	Explain how quantitative variables were handled in the analyses. If applicable, describe which groupings were chosen and why	✓	8, 9
Statistical methods	12	(*a*) Describe all statistical methods, including those used to control for confounding	✓	8, 9
		(*b*) Describe any methods used to examine subgroups and interactions	✓	8, 9
		(*c*) Explain how missing data were addressed	✓	8, 9
		(*d*) If applicable, explain how loss to follow-up was addressed	✓	8, 9
		(*e*) Describe any sensitivity analyses	✓	8, 9
**Results**				
Participants	13*	(a) Report numbers of individuals at each stage of study—eg numbers potentially eligible, examined for eligibility, confirmed eligible, included in the study, completing follow-up, and analysed	✓	Fig 2, pg 10

For peer review only - http://bmjopen.bmj.com/site/about/guidelines.xhtml

		(b) Give reasons for non-participation at each stage	✓	Fig 2, pg 10
		(c) Consider use of a flow diagram	✓	Fig. 2
Descriptive data	14*	(a) Give characteristics of study participants (eg demographic, clinical, social) and information on exposures and potential confounders	✓	Fig 2, pg 10
		(b) Indicate number of participants with missing data for each variable of interest	✓	Fig 2, pg 10
		(c) Summarise follow-up time (eg, average and total amount)	✓	Fig 2, pg 10
Outcome data	15*	Report numbers of outcome events or summary measures over time	✓	10
Main results	16	(*a*) Give unadjusted estimates and, if applicable, confounder-adjusted estimates and their precision (eg, 95% confidence interval). Make clear which confounders were adjusted for and why they were included	✓	10, 11
		(*b*) Report category boundaries when continuous variables were categorized	✓	10, 11
		(*c*) If relevant, consider translating estimates of relative risk into absolute risk for a meaningful time period	n/a	n/a
Other analyses	17	Report other analyses done—eg analyses of subgroups and interactions, and sensitivity analyses	✓	10, 11
**Discussion**				
Key results	18	Summarise key results with reference to study objectives	✓	12
Limitations	19	Discuss limitations of the study, taking into account sources of potential bias or imprecision. Discuss both direction and magnitude of any potential bias	✓	12, 13
Interpretation	20	Give a cautious overall interpretation of results considering objectives, limitations, multiplicity of analyses, results from similar studies, and other relevant evidence	✓	12-14
Generalisability	21	Discuss the generalisability (external validity) of the study results	✓	13
**Other information**				
Funding	22	Give the source of funding and the role of the funders for the present study and, if applicable, for the original study on which the present article is based	✓	15

*Give information separately for exposed and unexposed groups.

Note: An Explanation and Elaboration article discusses each checklist item and gives methodological background and published examples of transparent reporting. The STROBE checklist is best used in conjunction with this article (freely available on the Web sites of PLoS Medicine at http://www.plosmedicine.org/, Annals of Internal Medicine at http://www.annals.org/, and Epidemiology at http://www.epidem.com/). Information on the STROBE Initiative is available at http://www.strobe-statement.org.

For peer review only - http://bmjopen.bmj.com/site/about/guidelines.xhtml
